# Study of the Metabolite Changes in *Ganoderma lucidum* under Pineapple Leaf Residue Stress via LC-MS/MS Coupled with a Non-Targeted Metabolomics Approach

**DOI:** 10.3390/metabo13040487

**Published:** 2023-03-28

**Authors:** Yijun Liu, Yangyang Qian, Chunyu Wang, Yingying He, Chuxing Zhu, Gang Chen, Lijing Lin, Yuliang Chen

**Affiliations:** 1 State Key Laboratory of Pulp and Paper Engineering, College of Light Industry and Engineering, South China University of Technology, Guangzhou 510640, China; 2 Hainan Key Laboratory of Storage & Processing of Fruits and Vegetables, Agricultural Products Processing Research Institute, Chinese Academy of Tropical Agricultural Sciences, Zhanjiang 524001, China; 3 Key Laboratory of Tropical Crop Products Processing of Ministry of Agriculture and Rural Affairs, Agricultural Products Processing Research Institute, Chinese Academy of Tropical Agricultural Sciences, Zhanjiang 524001, China; 4 College of Tea (Pu’er), West Yunnan University of Applied Sciences, Pu’er 665000, China

**Keywords:** pineapple leaf residue, *G. lucidum*, non-targeted metabolomics, metabolic pathways

## Abstract

The effects of fermentation metabolites of *G. lucidum* under different pineapple leaf residue additions were separated and identified using liquid chromatography coupled with tandem mass spectrometry (LC-MS/MS). The mass spectra showed that the metabolites had good response values only in the positive ion mode, and 3019 metabolites with significant differences, mainly distributed in 95 metabolic pathways, were identified. The multivariate analyses, including the principal component analysis (PCA), orthogonal least squares discriminant analysis (OPLS-DA), and volcano plots (VP), revealed that the *G. lucidum* metabolites exhibited significant differences (*p* < 0.05) and were well clustered under various pineapple leaf residue additions, featuring 494–545 upregulated and 998–1043 downregulated metabolites. The differential metabolic pathway analysis proved that two metabolic pathways related to the biosynthesis of amino acids and ABC transporters were particularly significant under the addition of pineapple leaf residue, where amino acids such as histidine and lysine were upregulated in contrast to downregulated tyrosine, valine, L-alanine, and L-asparagine. These study results are considered instrumental in substantiating the application of pineapple leaf residue in the cultivation of *G. lucidum* and improving its utilization rate and added value.

## 1. Introduction

Pineapples are a popular tropical fruit. The main pineapple cultivation areas in China are Guangdong, Hainan, Guangxi, and Yunnan provinces. Nearly 80% of the pineapple produced in China goes to the domestic market as fresh fruit [1]. After pineapple fruit harvesting, its by-products (such as pineapple roots, stems, and leaves) are mostly crushed and returned to farmland, producing a large amount of waste and losing valuable ingredients [2,3]. This study focuses on pineapple leaf residue obtained from pineapple stalks and leaves left after harvesting through fiber extraction and other related processes. Pineapple leaf residue is rich in amino acids, pineapple protease, and other characteristic nutrients [4,5]. It has good intake and digestion properties, satisfactory physical effectiveness [6], promotes enzymatic digestion of proteins in feed [7,8], improves growth performance and nutrient digestibility in pigs and sheep, and reduces the amount and cost of concentrate feed [9]. In addition, pineapple leaf residue is rich in organic matter, such as carbohydrates, which can improve the gas production rate and pool capacity during traditional biogas fermentation when used for biogas preparation [10]. While it is exploited as a potential biomass for butanol production and the yield of butanol is 0.16 g/g [11], among other characteristics, its application is promising. However, increasing the added value and utilization rate of pineapple leaf residue is still problematic and requires further research efforts.

Of particular interest is the option of using pineapple leaf residue to promote the growth of *Ganoderma lingzhi*, which is a well-known medicinal and food fungus in China. Its growth requires nutritional components such as carbon, nitrogen, inorganic salts, and vitamins [12]. Carbon is a crucial component, providing the energy for mycelial growth but also participating in the formation of vitamins, proteins, and amino acids in the mycelium [13]. Nitrogen is also mandatory for the growth of the mycelium of *G. lucidum*, which mainly absorbs nitrogenous compounds in the reduced state, such as amino acids, proteins, urea, ammonia, ammonium salts, and nitrates [14]. Relevant research results show that heat treatment, ultrasonic treatment, NO concentration, nitrogen source concentration, and other factors can affect the expression of related functional genes during the growth of *G. lucidum*, resulting in significant differences in metabolites. The value of pH regulates the secretion of triterpenoids, intracellular and extracellular polysaccharides, mycelium proteins, and other fermentation metabolites and their antioxidant activities [15]. NO can regulate the expression of genes in the mevalonate pathway and promote the increase in triterpenoid content in *G. lucidum* [16]. Manganese ions can improve the activity of manganese peroxidases, such as chavicol and palmitoylethanolamide, in the *G. lucidum* fruiting body [17]. Ultrasonic treatment induced significant changes in the mycelium metabolism and transcription levels [18]. High temperature stress leads to the upregulation and downregulation of genes related to protein assembly, transportation, degradation, energy metabolism, etc. After heat stress, the *G. lucidum* fruiting body activates protein assembly and other processes, which promote a significant increase in polysaccharide yield [19]. The concentration of the nitrogen source can affect the expression of the AreA transcription factor in *G. lucidum*, which plays an important role in the growth, secondary metabolism, and resistance to cell wall stress of *G. lucidum* [20].

Pineapple leaf residue is rich in fast carbon sources, such as sucrose, as well as amino acids, vitamins, and trace metal elements, such as calcium and magnesium [21,22], which can be used as a nutritional additive to promote the growth of *G. lucidum* mycelium and increase its protein content. The annual production of pineapple leaf residue in China is vast. For example, over 200,000 tons are produced annually for the prefecture of the Guangdong province of China. This local pineapple industry would benefit by three million USD if the added value of pineapple leaf residue could be raised by 15 USD/ton using the above method. The types and contents of nitrogen sources and carbon sources had an influence on the growth of *G. lucidum*. Pineapple leaf residue contained fast carbon sources (including glucose) and fast nitrogen sources (including amino acids) needed for the rapid growth of *G. lucidum*. Compared with traditional cultivation substrates, the metabolic path of *G. lucidum* would change. Therefore, the content of stimulating nutrients would also change with different added amounts of pineapple leaf residue, which would lead to changes in the types and contents of metabolites of *G. lucidum*. Since the mechanism of changes in the metabolic pathways related to the application of pineapple leaf residue to the regulation of *G. lucidum* fermentation is not clear, this study analyzed the changes in metabolites of *G. lucidum* under pineapple leaf residue stress based on non-targeted metabolomics to substantiate the feasibility of the proposed application of pineapple leaf residue.

## 2. Materials and Methods

### 2.1. Preparation of Fermentation Substrates

Pineapple leaf residue was obtained by machine separation. The standard susceptible corn (maize) variety Yuenong No. 9 used in this study was purchased from the market. The pineapple leaf residue and corn were mixed at ratios of 1:7 (p1), 1:5 (p2), 1:3 (p3), and 1:1 (p4), where the blank group was p0 and the water content was adjusted to 55–60%. After bagging, sterilization, and cooling, the *G. lucidum* strain was inserted under aseptic conditions and cultured in dark light at 24–28 °C for 30 days. After the fermenting mycelium grew all over the bag, it was removed, dried at −40 °C, and crushed to obtain the fermentation substrate.

### 2.2. Extraction of Metabolites

A 30 mg sample was added to 300 μL of cold acetonitrile, shaken and extracted for 30 min, centrifuged at 15,403× *g*, and 4 °C for 10 min, and 100 μL was removed and concentrated to dryness by vacuum centrifugation at 37 °C. The residue was dissolved in 100 μL acetonitrile and centrifuged at 15,403× *g* and 4 °C for 10 min, and the supernatant was removed for detection.

### 2.3. Detection Conditions for LC-MS/MS

The parameters were based on the study of Wu et al. [23,24,25] with appropriate modifications. The ultrahigh-performance liquid chromatograph (UHPLC) (Nexera UHPLC LC-30A, Shimadzu, Japan) was equipped with a Waters BEH C18 (150 × 2.1 mm, 2.5 µm, Shimadzu, Japan) column. Ten microliters of sample were passed through the column at 40 °C. Mobile phase A was eluted at 0.3 mL/min for 2 min, followed by mobile phase B at 0.3 mL/min. Mobile phase A consisted of 5% acetonitrile and 95% formic acid at a 0.1% concentration, and mobile phase B consisted of 95% acetonitrile and 5% formic acid at a 0.1% concentration.

A mass spectrometer (TripleTOF5600+, AB SCIEX™, Framingham, MA, USA) provided electrospray ionization (ESI) positive and negative ion detection modes. ESI source conditions were as follows: ion source gas 1 50, ion source gas 2 50, curtain gas (CUR) 25, a source temperature of 500 °C (positive ions) and 450 °C (negative ions), an ion spray voltage floating (ISVF) of 5500 V (positive ions) and 4400 V (negative ions), a TOF MS scan range of 100–1200 Da, and a product ion scan range of 50–1000 Da. The TOF MS scan accumulation time was 0.2 s, and the product ion scan accumulation time was 0.01 s. Secondary mass spectra were acquired using information-dependent acquisition (IDA) and high sensitivity mode; delustering potential (DP) was ± 60 V, and collision energy was 35 ± 15 eV.

### 2.4. Database Search and Data Analysis

The raw data obtained by LC-MS/MS were converted to ABF format by the Analysis Base File Converter software. The ABF format files were imported into the MS-DIAL 4.70 software for pre-processing [26], including peak extraction, noise removal, deconvolution, peak alignment, and exporting the 3D data matrix in CSV format.

The extracted peak information was compared with the database, and a complete database search was performed for the three repositories: Mass Bank, Respect, and GNPS. The three-dimensional matrix included sample information, retention time, mass-to-nucleus ratio, and mass spectral response intensity (peak area). The peak detection parameters had a minimum peak height of 1000 amplitude and a mass slice width of 0.1 Da. The alignment parameters were set as follows: a retention time tolerance of 0.05 min and an MS1 tolerance of 0.015 Da. The identification setting envisaged accurate mass tolerances (MS1 and MS2) of 0.01 Da and 0.05 Da, respectively, and an identification score cut-off of 80.

The identified metabolites were annotated by the Kegg (Kyoto encyclopedia of genes and genomes) database (https://www.genome.jp/kegg/pathway.html, accessed on 9 June 2022). SIMCA (14.1, Sartorius Lab Instruments GmbH & Co. KG, Goettingen, Germany) was used for principal component analysis, s-plot drawing, and VIP value calculation. OriginPro (2021, OriginLab Corporation, Northampton, UK) was used to make heat maps. Photoshop (2022, Adobe Systems Incorporated, San Jose, CA, USA) was used to combine pictures.

## 3. Results and Discussion

### 3.1. Total Ionogram Analysis of Samples with LC-MS/MS Detection, PCA, and PLS-DA Analysis

The total ion flow plots of p0 are shown in Figure S1A,B. Figure S1A,B show that the sample had high response values in the positive ion mode and low response values in the negative ion mode, so the metabolite data obtained in the positive ion mode were chosen for analysis in this study. The mass spectra of the metabolites and other information are presented in Supplementary Materials Table S1.

**Figure 1:**
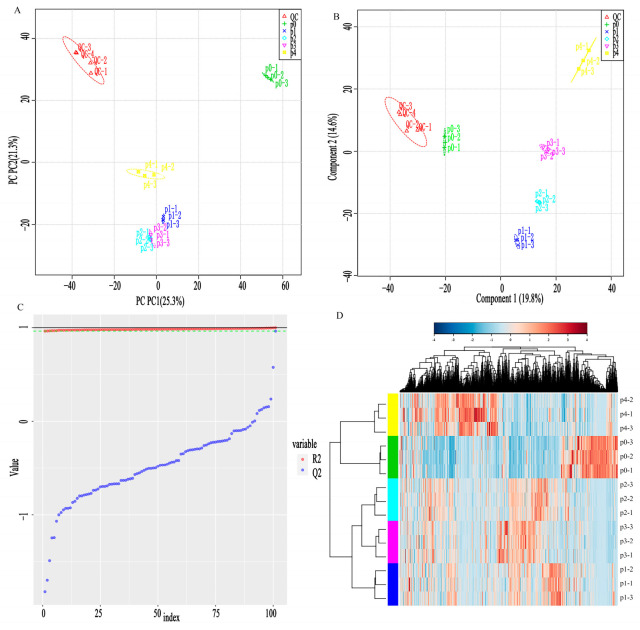
(**A**–**D**) shows the PCA plot, OPLS-DA plot, OPLS-DA model alignment test plot, and differential metabolite cluster analysis of pineapple leaf residues, respectively.

Figure 1A–D show the PCA plot, PLS-DA plot, PLS-DA model substitution test plot, and agglomerate hierarchical clustering of different metabolites for the samples obtained with various pineapple leaf residue additions, respectively. QC was the quality control sample, and the better aggregation in graphs (A) and (B) indicated an excellent instrument testing status. Figure 1 features an overlap between p2 and p3 in the PCA model, and five samples in the OPLS-DA model of Figure 1B were better distinguished from each other and had good clustering. Figure 1C represents the alignment test plot of the OPLS-DA model, where R^2^ is the prediction rate of the grouping and Q^2^ is the accuracy of the model prediction (R^2^ = 0.9752 and Q^2^ = 0.6942 in this study). The data indicated that the model had a reliable explanatory degree and predictive ability [27]. The rows and columns in Figure 1D represent the samples and metabolites, respectively. The metabolite content is shown with different colors, where p1 and p4 have the most significant difference. The more intense red color of p4 and p0 corresponds to a larger difference in metabolite differential concentrations.

### 3.2. Screening for Differential Metabolites between Samples

**Figure 2:**
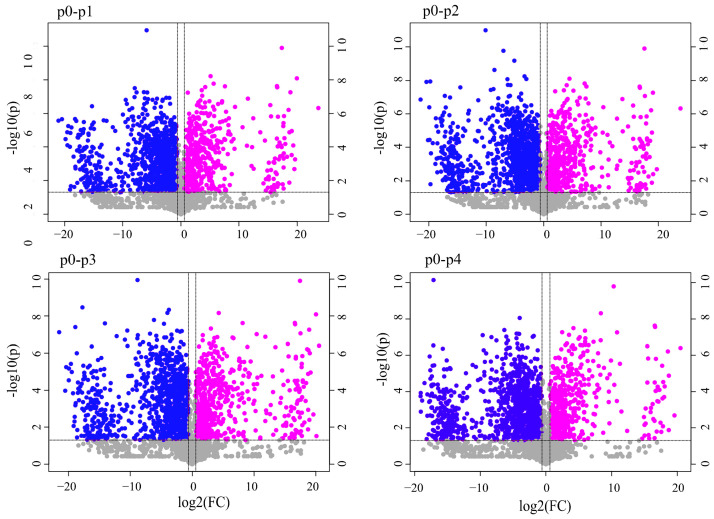
Volcanoes of differential metabolites with different pineapple leaf residue addition stresses.

Volcano plots were used to visualize the differences in metabolite content between the two groups of samples and the statistical significance of the differences [28]. The horizontal coordinate of the volcano plot was the ploidy level (logarithmic transformation with a base of 2), and the vertical coordinate was the statistical test of the difference (negative logarithmic transformation with a base of 10). In general, ploidy level is a term referring to the number of chromosome sets in somatic cells of the diplophase (2 n) or gametophytic cells of the haplophase (1 n). The larger the absolute value of the horizontal coordinate, the greater the difference in the ploidy expression between the two samples. The larger the value of the vertical coordinate, the more significant the difference in expression and the more reliable the screened differential substances. Such an approach has been widely used in the metabolite mining of fruits and vegetables, Chinese herbal medicines, etc. [28,29,30]. Each dot in the graph indicates a metabolite. Significantly upregulated and downregulated metabolites are indicated by pink and blue dots, respectively. As shown in Figure 2, a total of 1492 differential metabolites were present between p0 and p1 in the positive ion mode, and 494 were significantly upregulated in expression, including 3-(5,7-dimethoxy-4-oxochromen-2-yl)propanoic acid (454), the iodinated analog of Makalu (450), 3-[(3R,5S,10R,13R,14S,17R)-5,14-dihydroxy-10,13-dimethyl-3-[(2R,3R,4S,5S,6R)-3,4,5-trihydroxy-6-(hydroxymethyl)oxan-2-yl]oxy-2,3,4, 6,7,8,9,11,12,15,16,17-dodecahydro-1H-cyclopenta[a]phenanthren-17-yl]-2H-furan-5-one (2637), khivorin (2191), methazolamide (436), etc. There were 998 significantly downregulated expressions, including those of diphenyl phosphate (403), cyclo(tyrosyl-prolyl) (2506), 1-(2-(1H-indol-3-yl) ethyl-thiourea (583), and strophanthidin (2420). A total of 1609 differential metabolites were present between p0 and p2. Of them, 545 were significantly upregulated in expression, including 3-(5,7-dimethoxy-4-oxochromen-2-yl) propanoic acid (454), khivorin (2191), isocucurbitacin B (2503), huperzine A (57), gossypetin (1689), etc. The other 1064 significantly downregulated expressions included diphenyl phosphate (403), cyclo(tyrosyl-prolyl) (2506), 1-(2-(1H-indol-3-yl) ethyl) thiourea (583), buxtamine (2624), and strophanthidin (2420). A total of 1614 differential metabolites were present between p0 and p3. Of them, 575 were significantly upregulated, including uncarine c (1344), 4-(4-methoxyphenyl)-2-oxo-2H-chromen-7-yl 2-(4-methylphenylsulfonamido)-2- phenylacetate (834), the iodinated analog of makaluvone (450), and khivorin (2191). A total of 1039 significantly downregulated expressions were identified, including pendimethalin (735), strophanthidin (2420), buxtamine (2624), cyclo(tyrosyl-prolyl) (2506), diphenyl phosphate (403), etc. A total of 1575 differential metabolites were present between p0 and p4. A total of 532 were significantly upregulated in expression, including uncarine c (1344), khivorin (2191), gossypetin (1689), neoandrographolide (2125), butin (1687), phosphatidylinositol (2829), etc. A total of 1043 was significantly downregulated in expression, including cyclo(tyrosyl-prolyl) (2506), buxtamine (2624), (Z)-methyl 4-((9-(furan-2-yl)-3,7-dioxo-3,7,8,9-tetrahydro-2H-furo [2,3-f] chromen-2-ylidene) methyl) benzoate (740), 3-(1,2-dihydroxypropyl)-1,6,8-trihydroxyanthracene-9,10-dione (352), epicatechin (306), diphenyl phosphate (403), etc.

### 3.3. Differential Metabolite KEGG Metabolic Pathway Analysis

**Figure 3:**
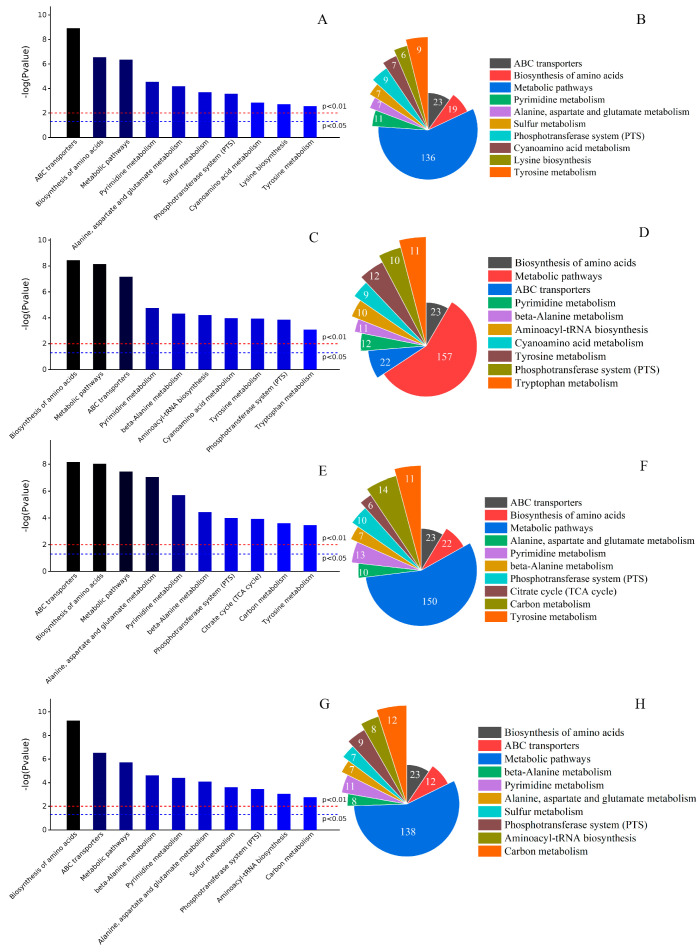
KEGG metabolic pathway enrichment histograms for p0 vs. p1 (**A**), p0 vs. p2 (**C**), p0 vs. p3 (**E**), and p0 vs. p4 (**G**). Top 10 metabolic pathway species for p0 vs. p1 (**B**), p0 vs. p2 (**D**), p0 vs. p3 (**F**), and p0 vs. p4 (**H**).

To explore the regulatory role of pineapple leaf residue on *G. lucidum*, this study enriched the KEGG database for differential metabolites into shaped pathways and identified 3019 significantly different metabolites mainly distributed in 95 metabolic pathways, among which p0 vs. p1, p0 vs. p2, p0 vs. p3, and p0 vs. p4 KEGG metabolic pathways were 87, 93, 88, and 89, respectively. The enrichment histogram of the top ten metabolic pathways and the number of metabolite species are shown in Figure 3. According to Figure 3, the metabolic pathways affecting the fermentation of *G. lucidum* under different pineapple leaf residue additions were as follows: biosynthesis of amino acids, ABC transporters, metabolic pathways, β-alanine metabolism, pyrimidine metabolism, alanine, aspartate and glutamate metabolism, sulfur metabolism, phosphotransferase system (PTS), aminoacyl-tRNA biosynthesis, carbon metabolism, cyan amino acid metabolism, lysine biosynthesis, tyrosine metabolism, and citrate cycle (TCA cycle). The top three metabolic pathways were the biosynthesis of amino acids, ABC transporters, and metabolic pathways. Among the top three metabolic pathways. The -log (*p* value) and the number of species for p0 vs. p1 were (6.56, 19), (8.93, 23), and (6.35, 136), respectively. For p0 vs. p2, these were (8.44, 23), (7.16, 22), and (8.14, 157), respectively. For p0 vs. p3, these were (8.03, 22), (8.17, 23), and (7.46, 150), respectively. For p0 vs. p4, the respective parameters were (9.26, 23), (6.53, 20), and (5.73, 138).

In addition, it can be seen in Figure 3 that various additions of pineapple leaf residue differentially regulated the metabolic pathways of *G. lucidum* fermentation, which were closely related to protein synthesis and nucleotide synthesis. The synthesis of amino acids was mainly related to the citric acid cycle pathway, the glycolysis cycle pathway, the pentose phosphate pathway, and the amino acid decomposition pathway [31,32,33,34]. From Figure 3, the biosynthesis of amino acids and ABC transporter metabolic pathways associated with protein synthesis were particularly significant. At the same time, the metabolic pathways related to amino acid metabolism (including cyan amino acid metabolism, lysine biosynthesis, tyrosine metabolism, the citric acid cycle, and so on) were also active, which showed that pineapple leaf residue affected not only amino acid biosynthesis but also many metabolic pathways related to amino acid metabolism.

### 3.4. Differential Metabolite Heatmap Analysis of the Biosynthesis of Amino Acids and ABC Transporters

**Figure 4:**
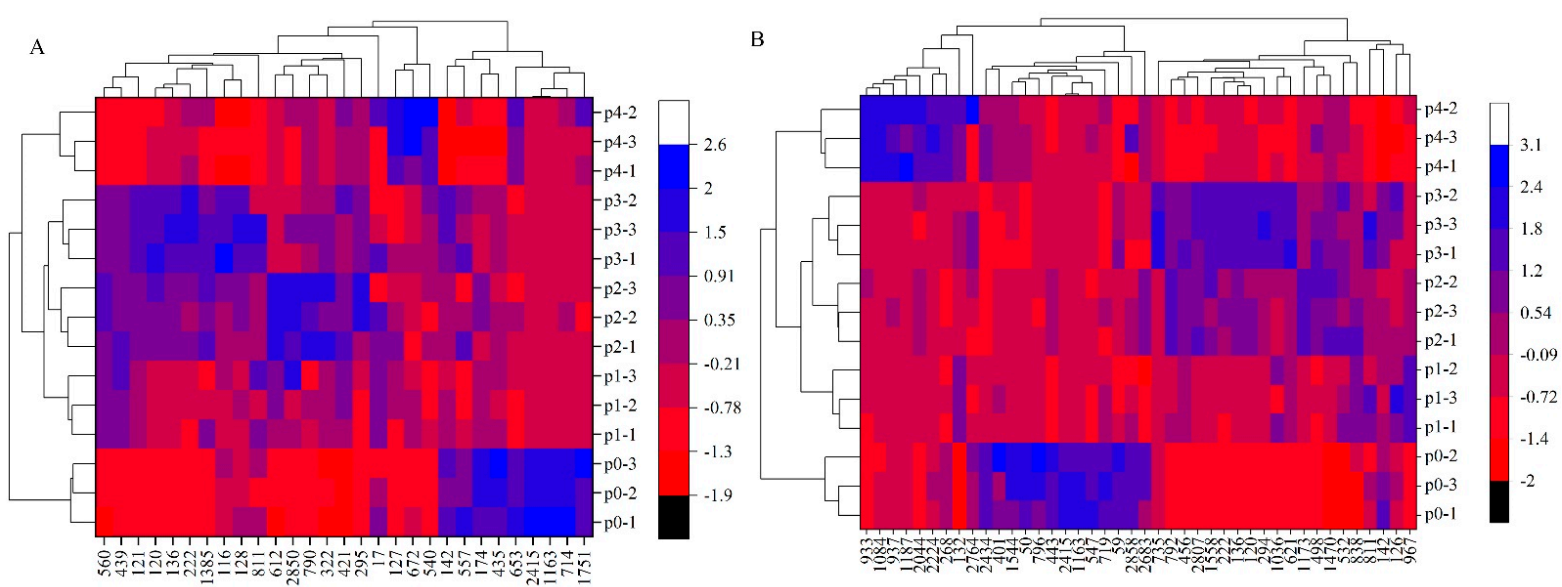
Thermographic analysis of differential metabolites in the metabolic pathways of (**A**) amino acid biosynthesis and (**B**) ABC transporter biosynthesis.

Differential metabolites in the biosynthesis of amino acids and ABC transporters metabolic pathways of *G. lucidum* under stress with different pineapple leaf residue additions are shown in Figure 4A,B, respectively. As seen in Figure 4A, compared with p0, 29 differential metabolites in the biosynthesis of amino acids, L-aspartic acid (2415), L-histidine (1163), isopropylmalic acid (714), d-ribose 5 phosphate (435), and 2,6-diaminopimelic acid (174), exhibited an overall upregulation trend. The metabolites L-citrulline (127), 3-dehydroshikimate (672), and citric acid (540) showed an overall downregulation trend. It could be inferred that pineapple leaf residue promoted the transformation of related metabolites in the TCA cycle into amino acids, such as glutamic acid and histidine. The pineapple leaf residue addition was between p1 and p3, and the overall downregulation trend of metabolites such as isocitric acid (560), o-succinylhomoserine (439), and l-asparagine (121), was quite pronounced. As seen in Figure 4B, compared to p0, among the 42 differential metabolites in ABC transporters, raffinose (401), melatonin (1544), spermidine (50), deoxyuridine (796), sn-glycerol 3-phosphate (443), L-aspartic acid (2415), L-histidine (1163), mannitol (547), 3,4-dihydrocoumarin (719), thiamine (59), and sorbitol (2858) showed an overall upregulation trend. Meanwhile, 13-(2-(4-(2-methoxyphenyl) piperazin-1-yl)-2-oxoethyl)-7,8-dihydroindolo [2’,3’:3,4] pyrido[2,1-b] quinazolin-5(13H)-one (993), N-(2- furylmethyl)-9H-purin-6-amine (1084), N-(1,3-dihydroxy-2-methylpropan-2-yl)-3-methyl-4-oxo-2-phenyl-4H-chromene-8-carboxamide (937), denudatine (1187), and seven other compounds showed downregulation in p4. Such compounds as pendimethalin (735), loureirin b (792), quebrachitol (456), eschscholtzxanthin (2807), and glyasperin c (1558) showed a downregulation trend between p1 and p3. Previous research by Deng et al. showed that the gas production of methanogens and other microorganisms decreased with the increase in pineapple leaf residue, which interfered with the metabolism of methane and other microorganisms and affected the yield rate and yield [10]. Carbon sources (glucose, galactose, rhamnose, xylose, and mannose) and nitrogen sources (yeast powder, yeast extract, and peptone) affected the apparent viscosity, exopolysaccharide yield, and corresponding sugar content of *G. lucidum* fermentation broth [14]. Therefore, it could be inferred that the contents of the rapid nitrogen source, carbon source, and trace metal elements in the culture medium were different due to the different added amount of pineapple leaf residue, and the related metabolic pathways affecting the growth process of *G. lucidum* showed a significant upregulation and downregulation trend from the related metabolites.

### 3.5. Metabolic Pathway Analysis of the Biosynthesis of Amino Acids

**Figure 5:**
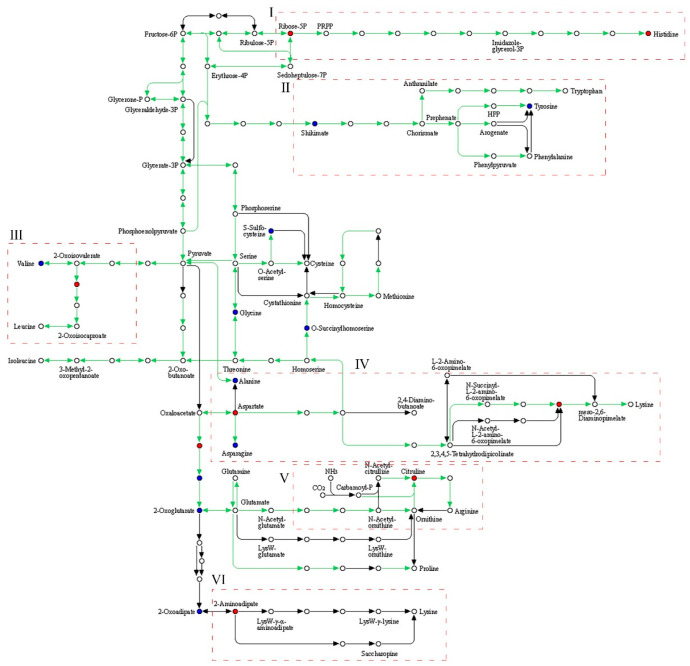
Biosynthesis of amino acids metabolic pathway (p0 vs. p3). Red and green dots represent the upregulation and downregulation of metabolites, respectively. I, II, III, IV, V, and VI represent the histidine metabolic pathway, tyrosine metabolic pathway, valine metabolic pathway, lysine metabolic pathway, arginine metabolic pathway and lysine metabolic pathway.

As shown in Figure 4, the overall upregulation trend of metabolites such as L-aspartic acid (2415), L-histidine (1163), isopropylmalic acid (714), d-ribose 5-phosphate (435), and 2,6-diaminopimelic acid (174) was evident. The metabolic pathway of biosynthesis of amino acids in *G. lucidum* under pineapple leaf residue stress is shown in Figure 5 (p0 vs. p3 metabolic pathway). The metabolic pathway analysis of the biosynthesis of amino acids is shown in Figure 5. As shown in Figure 5 I, pineapple leaf residue induced Sedoheptulose-7P to be transformed into Ribose-5p (435), which was transformed into ribose-5-phosphate-1-pyrophosphate (PRPP) under the action of phosphorylase, and finally Histine (1163) was generated under the action of a series of enzymes while the metabolites ribose-5p and histidine were upregulated. As shown in Figure 5 II, shikimate (421) was transformed into chorismate under the action of related enzymes, and tryptophane was generated under the action of prephenate, aminotransferase, and a series of enzymes. With the downregulation of shikimate, the metabolic pathway of tyrosine biosynthesis was weakened, and the expression of tryptophan was downregulated. As seen in Figure 5 III, pineapple leaf residue stimulated the metabolism pathway of leucine biosynthesis, induced 2-oxosovererate to be transformed into 2-isopropylmalic acid (811), and generated 2-oxosocaproate under the action of a series of enzymes, thus the leucine and valine expressions were upregulated and downregulated, respectively. As shown in Figure 5 IV, the expression of L-aspartate (2415) was promoted, the lysine biosynthesis metabolic pathway was active, and the expression of lysine was upregulated, while the expression of L-alanine (136) and l-asparagine (121) was downregulated by stimulation of pineapple leaf residue in *G. lucidum* fermentation. As seen in Figure 5 V, with the participation of pineapple leaf residue, the metabolic pathway of arginine biosynthesis was active during the growth of *G. lucidum* mycelium, and there were many ways to promote the upregulation of citrulline (127) expression. Carbamoyl-P was transformed into N-Acetylcitrulline with the participation of CO_2_ and NH_3_, and then into citrulline with the help of related enzymes. 2-Oxoglutatarate was transformed into N-acetyl-omitine with the participation of various enzymes and glutamate, and citruline was finally generated. According to Figure 5 VI, with the addition of pineapple leaf residue, the metabolic pathway of lysine biosynthesis was active during the growth of *G. lucidum* mycelium. Under the action of related enzymes, 2-oxoadipate (322) was transformed into 2-aminoadipate (653), the expression of 2-oxoadipate was downregulated, and the expression of 2-aminoadipate was upregulated. 2-aminooadpate was transformed into lys2-r-a-aminooadjpate, and after a series of changes, LysW-r-Lysine was produced, which promoted the synthesis of lysine, or transformed into saccharopin under the action of related enzymes to promote the synthesis of lysine.

With the development of microbiology and other disciplines, much progress has been made in the research, development, and application of amino acids, and the application fields have covered the markets of animal feed, health food, dietary supplements, pharmaceutical products, artificial sweeteners, and cosmetics. Food and animal feed were the main application fields of amino acids in China. Protein is an essential nutrient in the process of animal growth and development and plays an important biological function in the process of life activities. Amino acids are the basic building blocks of protein. With the vigorous development of national animal husbandry, the gap in protein-related feed was increasing, and amino acids such as lysine and histidine were essential amino acids for animal growth and development as well as being very scarce in animal feeding. Amino acids are closely related to animal metabolism, not only promoting animal growth but also improving feed utilization [35]. Among them, lysine not only participated in protein synthesis (including skeletal muscle, peptide hormones, plasma proteins, enzymes, and other key proteins), lipid metabolism, ketone body metabolism, and glucose production, but also regulated the expression of some genes in intestinal cells, which had an impact on the absorption and metabolism of other amino acids and further affected the absorption and metabolism of proteins. In the process of feeding animals, lysine could promote animal growth, improve meat quality, improve immunity, improve reproductive performance, and so on [36,37,38]. Histidine also plays an important role in protein synthesis, nervous system regulation, cell pH stability, and body health in animals [39,40]. In this study, pineapple leaf residue was applied to the cultivation of *G. lucidum*. During the growth of *G. lucidum*, pineapple leaf residue polysaccharide, fiber, and other components were transformed into nutrients needed for its growth and metabolism, and at the same time, amino acids and other metabolites were secreted. Pineapple leaf residue contained a variety of trace metal elements, fast-acting carbon sources, and nitrogen sources to regulate the amino acid metabolism pathway of *G. lucidum*. It promoted the secretion of lysine, histidine, and other amino acids, which provided an important new path for the application of pineapple leaf residue.

## 4. Conclusions

The results showed that the metabolites of *G. lucidum* significantly differed at various levels of pineapple leaf residue stress, which interfered with 95 metabolic pathways during the growth of *G. lucidum*, among which the amino acid biosynthesis and ABC transporter protein metabolic pathways were the most affected. This study revealed the molecular mechanism of the response of *G. lucidum* to pineapple leaf residue stress, whereas such amino acids as histidine and lysine were upregulated, in contrast to tyrosine, valine, L-alanine, and L-asparagine, which were downregulated. This study provided basic data for substantiating the use of pineapple leaf residue and improving its added value. Verification of the role of related metabolites in the stress process is planned for the follow-up study.

## Data Availability

Not applicable.

## Supplementary Materials

The following supporting information can be downloaded at: https://www.mdpi.com/article/10.3390/metabo13040487/s1, Figure S1: Total ion flow diagram in positive ion mode (**A**) and negative ion mode (**B**) of LC-MS/MS.; Table S1: Identification information of metabolite in *G.lucidum* under pineapple leaf residue stress.

## References

[1] Liu C.H., He H., He X.G., Shao X.H., Kuang S.Z., Xiao W.Q. (2021). Pineapple cultivar structure and extrension of new idependent breeding pineapple cultivars in China. China Trop. Agric..

[2] Van Tran T., Nguyen D.T.C., Nguyen T.T.T., Nguyen D.H., Alhassan M., Jalil A., Nabgan W., Lee T. (2023). A critical review on pineapple (Ananas comosus) wastes for water treatment, challenges and future prospects towards circular economy. Sci. Total. Environ..

[3] Zhang Y., Ou Z.Q., Deng Y.G., Cui Z.D., Li L., Zhang J., Wang Y.Q., Wei L.J., Liu S.L. (2019). Development and experiments of type 1JHB-150 pineapple leaf shattering and returning machine. J. Agric. Mech. Res..

[4] Zhou W., Ye C., Geng L., Chen G., Wang X., Chen W., Sa R., Zhang J., Zhang X. (2021). Purification and characterization of bromelain from pineapple (*Ananas comosus L*.) peel waste. J. Food Sci..

[5] Ramli A.N.M., Manas N.H.A., Hamid A.A.A., Hamid H.A., Illias R.M. (2018). Comparative structural analysis of fruit and stem bromelain from Ananas comosus. Food Chem..

[6] Liu C., Asano S., Ogata H., Ito S., Nakase T., Takeda S., Miyoshi K., Numata Y., Takahashi K., Kajikawa H. (2021). Digestive, fermentative, and physical properties of pineapple residue as a feed for cattle. Anim. Sci. J..

[7] Zhao Y.Y., Fu B.M., Wang Y., Ye C. (2020). Pharmacological activity of pineapple protease and its application in livestock and poultry diseases. Anim. Breed. Feed..

[8] Bahri S., Hadati K.S., Satrimafitrah P. (2021). Production of protein hydrolysate from tofu dregs using the crude extract of bromelain from pineapple core (Ananas comosus l). J. Physics: Conf. Ser..

[9] Wu Z.M., Peng S., Wang Z., Yang K., Qiu X.Y., Wu P.X., Xu Y.W., Wang J.L., Yin F.Q. (2022). Evaluation of *Lactic acid bacteria* and cellulase on silage fermentation quality and fermentation in vitro of pineapple residue. Chin. J. Anim. Nutr..

[10] Deng Y.G., Wang J.L., Sun W.S., Jiao J., Zheng Y., Wang G. (2010). Effect of dry matter concentration on dry anaerobic fermentation for biogas production in pineapple leaf residues. Anhui Agric. Sci..

[11] Sajjanshetty R., Kulkarni N.S., Shankar K., Jayalakshmi S., Sreeramulu K. (2021). Enhanced production and in-situ removal of butanol during the fermentation of lignocellulosic hydrolysate of pineapple leaves. Ind. Crop. Prod..

[12] Ling G.W., Jia C.Y., Ze Y.S., Qi W., Fang Z., Zhi H.J., Cong L.X., Fan R.P., Zhong A.L., Li N. (2022). Ganoderic acids-rich ethanol extract from *Ganoderma lucidum* protects against alcoholic liver injury and modulates intestinal microbiota in mice with excessive alcohol intake. Curr. Res. Food Sci..

[13] Wu T., Liu X., Wang T., Tian L., Qiu H., Ge F., Zhu J., Shi L., Jiang A., Yu H. (2022). Heme Oxygenase/Carbon Monoxide Participates in the Regulation of *Ganoderma lucidum* Heat-Stress Response, Ganoderic Acid Biosynthesis, and Cell-Wall Integrity. Int. J. Mol. Sci..

[14] Liu L., Feng J., Gao K., Zhou S., Yan M., Tang C., Zhou J., Liu Y., Zhang J. (2022). Influence of carbon and nitrogen sources on structural features and immunomodulatory activity of exopolysaccharides from Ganoderma lucidum. Process. Biochem..

[15] Tang C.W., Zhang J.S., Liu Y.F., Tang Y., Wang P., Feng J. (2023). Effects of pH on metabolites and antioxidant activities of *Ganoderma lingzhi* liquid fermentation. Mycosystema.

[16] Gu L., Zhong X., Lian D., Zheng Y., Wang H., Liu X. (2017). Triterpenoid biosynthesis and the transcriptional response elicited by nitric oxide in submerged fermenting Ganoderma lucidum. Process. Biochem..

[17] Zhang B., Zhou J., Li Q., Gan B., Peng W., Zhang X., Tan W., Jiang L., Li X. (2019). Manganese affects the growth and metabolism of *Ganoderma lucidum* based on LC-MS analysis. PeerJ.

[18] Sun L., Liu L.-P., Wang Y.-Z., Yang L., Zhang C., Yue M.-X., Dabbour M., Mintah B.K., Wang L. (2021). Effect of ultrasonication on the metabolome and transcriptome profile changes in the fermentation of Ganoderma lucidum. Microbiol. Res..

[19] Ma X., Wang P., Zhou S., Sun Y., Liu N., Li X., Hou Y. (2015). De novo transcriptome sequencing and comprehensive analysis of the drought-responsive genes in the desert plant Cynanchum komarovii. BMC Genom..

[20] Zhu J., Song S.Q., Yue S.N., Lian L.D., Zhao M.W. (2020). Biological function of the transcription factor AreA in *Ganoderma lingzhi*. Mycosystema.

[21] Ahmed O.H., Husni M.H.A., Anuar A.R., Hanafi M.M. (2003). Production of Humic Acid from Pineapple Leaf Residue. J. Sustain. Agric..

[22] Ahmed O.H., Husni A.M., Anuar R.A., Hanafi M.M. (2003). Alternative means of recycling pineapple leaf residues. Fruits.

[23] Wu S.L., Tang M., Zhang X.M., Tang J. (2022). Analysis of metabolites change from flowering to withering of Rhododendron delavayi based on LC-MS/MS. Guihaia.

[24] Li Z.N., Liu C., Wu Q.F., Wang F., Chen H.P., Liu Y.P. (2022). Metabolomics analysis of eucommiae cortex obtained by different processing and drying methods based on ultra-performance liquid chromatography-tandem mass spectrometry. J. Instrum. Anal..

[25] Ji B.C., Li Z.G., Huang Y.C., Zhou W.X., Yang Y., Du T. (2022). Comparative analysis of metabolomes in fruits of different mulberry varieties based on LC-MS technology. Acta Sericologica Sin..

[26] Tsugawa H., Cajka T., Kind T., Ma Y., Higgins B., Ikeda K., Kanazawa M., VanderGheynst J., Fiehn O., Arita M. (2015). MS-DIAL: Data-independent MS/MS deconvolution for comprehensive metabolome analysis. Nat. Methods.

[27] Yi-Jun L., Yang-Yang Q., Bing S., Yang-Yang L., Xing-Hao T., Hong-Jun O., Yahui L., Ge T., Zi-Wei Y., Fei C. (2021). Effects of four drying methods on Ganoderma lucidum volatile organic compounds analyzed via headspace solid-phase microextraction and comprehensive two-dimensional chromatography-time-of-flight mass spectrometry. Microchem. J..

[28] Yang J.B., Wang Q., Wang X.T., Chen X.L., Wang Y., Gao H.Y., Song Y.F., Wei F., Ma S.C. (2022). Difference analysis of chemical composition of polygoni multiflori radix and polygoni multiflori radix preparata based on plant metabonomics. Chin. J. Pharmacovigil..

[29] Huang R.S., Zhan R.J., Chen B.H., Wang Y., Liao B.Y., Zhang Q.W., Li C.N. (2022). Comparative study on the quality of mulberry leaf vegetable from different mulberry varieties based on metabolomics. Acta Sericologica Sin..

[30] Gu Y., Zang P., Li J., Yan Y., Wang J. (2022). Plasma metabolomics in a deep vein thrombosis rat model based on ultra-high performance liquid chromatography-electrostatic field orbitrap high resolution mass spectrometry. Chin. J. Chromatogr..

[31] Xiong Y., Zhang F.-L., Li J.-R., Peng P.-Z., Liu B., Zhao L.-N. (2021). Ganoderma lucidum protease hydrolyzate on lipid metabolism and gut microbiota in high-fat diet fed rats. Food Biosci..

[32] Lv X.-C., Wu Q., Cao Y.-J., Lin Y.-C., Guo W.-L., Rao P.-F., Zhang Y.-Y., Chen Y.-T., Ai L.-Z., Ni L. (2022). Ganoderic acid A from *Ganoderma lucidum* protects against alcoholic liver injury through ameliorating the lipid metabolism and modulating the intestinal microbial composition. Food Funct..

[33] Viroel F.J.M., Laurino L.F., Caetano L.A., Jozala A.F., Spim S.R.V., Pickler T.B., Sercundes M.K., Gomes M.C., Hataka A., Grotto D. (2022). *Ganoderma lucidum* Modulates Glucose, Lipid Peroxidation and Hepatic Metabolism in Streptozotocin-Induced Diabetic Pregnant Rats. Antioxidants.

[34] Li H., Liu J., Hou Z., Luo X., Lin J., Jiang N., Hou L., Ma L., Li C., Qu S. (2022). Activation of mycelial defense mechanisms in the oyster mushroom Pleurotus ostreatus induced by Tyrophagus putrescentiae. Food Res. Int..

[35] Kato M., Urabe S., Matsuzawa S., Kato A., Fukazawa M., Hiyama E., Kurii A., Mikami N., Kitajima Y., Hida M. (2022). High-volume pre-dilution on-line hemodiafiltration is the adequate blood purification method from the viewpoint of amino acid nutrition. Ren. Replace. Ther..

[36] Li J.L., Guo C.Z., Wang S.J., Wang J.B., Sui S.S. (2020). Research advance on biological activities and application of lysine. China Feed..

[37] Zhang Y.M. (2022). Effects of Dietary Lysine Supplementation on Wool Production and Gut Microbes of *Angora Rabbits*. Master’s Thesis.

[38] An S.H., Kang H.-K., Kong C. (2022). Standardized ileal digestible lysine requirements of 21–28 days old male broilers. Anim. Feed. Sci. Technol..

[39] Baldi G., Soglia F., Laghi L., Meluzzi A., Petracci M. (2020). The role of histidine dipeptides on postmortem acidification of broiler muscles with different energy metabolism. Poult. Sci..

[40] Kasiga T., White B.M., Bruce T.J., Brown M.L. (2020). Effect of fish meal replacement with CarinataBrassica carinatain low animal protein diets of rainbow troutOncorhynchus mykiss (Walbaum) on trypsin activity, protein and amino acid digestibility and bioavailability. Aquac. Res..

