# Supplementary material for: Study of the Metabolite Changes in Ganoderma lucidum under Pineapple Leaf Residue Stress via LC-MS/MS Coupled with a Non-Targeted Metabolomics Approach
Source: Metabolites. 2023 Mar 28;13(4):487. doi: 10.3390/metabo13040487 (PMC10144527; doi:10.3390/metabo13040487)
Supplement: Supplementary file 1 [file metabolites-13-00487-s001.zip › metabolites-2213186-supplementary.pdf]

Study of the metabolite changes in *Ganoderma lucidum* under pineapple leaf residue stress via LC-MS/MS coupled with a non-targeted metabolomics approach

Yijun Liu [1,2,3†], Yangyang Qian[1,4†], Chunyu Wang[1], Yingying He[1], Chuxing Zhu[1], Gang Chen [1,*], Lijing Lin[2,3] and Yuliang Chen[2,3]

[1] State Key Laboratory of Pulp and Paper Engineering, College of Light Industry and Engineering, South China University of Technology, Guangzhou 510640, China;
[2] Hainan Key Laboratory of Storage & Processing of Fruits and Vegetables, Agricultural Products Processing Research Institute, Chinese Academy of Tropical Agricultural Sciences, Zhanjiang 524001, China;
[3] Key Laboratory of Tropical Crop Products Processing of Ministry of Agriculture and Rural Affairs, Agricultural Products Processing Research Institute, Chinese Academy of Tropical Agricultural Sciences, Zhanjiang 524001, China;
[4] College of Tea (Pu'er), West Yunnan University of Applied Sciences, Pu'er 665000, China.
† These authors contributed equally to this work.
* Correspondence: papercg@scut.edu.cn.

Table S1. Identification information of metabolite in *G.lucidum* under pineapple leaf residue stress

	Average Rt(min)	Average Mz	Metabolite name	Adduct type	Reference m/z	Formula	Ontology	INCHIKEY	SMILES
1	0.161	259.96069	N-acetyl-O-methyltyrosine	[M+H]+	259.96497	C12H15NO4	Phenylalanine and derivatives	HRUASHPCEMXOMR-UHFFFAOYSA-N	O=C(O)C(N=C(O)C)CC1=CC=C(OC)C=C1
2	0.168	204.9659	PYROGALLIN	[M+H]+	204.96191	C11H8O4	Tropolones	OLSYHLURTGEIBH-UHFFFAOYSA-N	O=C1C(O)=CC=CC=2C=CC(O)=C(O)C12
3	0.172	283.26163	(9Z)-9-octadecenoic acid	[M+H]+	283.26001	C18H34O2	Long-chain fatty acids	ZQPPMHVWECSIRJ-KTKRTIGZSA-N	O=C(O)CCCCCCCC=CCCCCCCCC
4	0.194	111.03909	Butyric acid	[M+H]+	111.04	C4H8O2	Straight chain fatty acids	FERIUCNNQQJTOY-UHFFFAOYSA-N	O=C(O)CCC
5	0.322	118.08617	Betaine	[M+H]+	118.08626	C5H11NO2	Alpha amino acids	KWIUHFFTVRNATP-UHFFFAOYSA-N	C[N+](C)(C)CC(=O)[O-]
6	0.333	191.96115	Clopyralid	[M+H]+	191.96136	C6H3Cl2NO2	Pyridinecarboxylic acids	HUBANNPOLNYSAD-UHFFFAOYSA-N	C1=CC(Cl)=NC(=C1Cl)C(=O)O
7	0.34	271.26318	Heptadecanoic acid	[M+H]+	271.26315	C17H34O2	Long-chain fatty acids	KEMQGTRYUADPNZ-UHFFFAOYSA-N	O=C(O)CCCCCCCCCCCCCCCC
8	0.352	186.01845	O-PHOSPHO-L-SERINE	[M+H]+	186.02	C3H8NO6P	Alpha amino acids	BZQFBWGGLXLEPQ-UHFFFAOYSA-N	O=C(O)C(N)COP(=O)(O)O
9	0.367	171.03514	2-(2,4-dioxo-3,4-dihydropyrimidin-1(2H)-yl)acetic acid	[M+H]+	171.03999	C6H6N2O4	Alpha amino acids and derivatives	ZFNQFXDDQAEAFI-UHFFFAOYSA-N	O=C1C=CN(C(=O)N1)CC(=O)O
10	0.4	176.01422	1-Methylsulfinylbutenyl isothiocyante	[M+H]+	176.01984	C6H9NOS2	Glucosinolate breakdown metabolites	QKGJFQMGPDVOQE-UHFFFAOYNA-N	CS(=O)C=CCCN=C=S
11	0.428	163.05971	3-Hydroxy-3-Methylglutaric acid	[M+H]+	163.0601	C6H10O5	Hydroxy fatty acids	NPOAOTPXWNWTSH-UHFFFAOYSA-N	CC(O)(CC(O)=O)CC(O)=O
12	0.438	199.17021	3,7-dimethyl-6-octenyl acetate	[M+H]+	199.17	C12H22O2	Fatty alcohol esters	JOZKFWLRHCDGJA-LLVKDONJSA-N	O=C(OCCC(C)CCC=C(C)C)C
13	0.439	96.96992	Phosphoric acid	[M-H]-	96.96962	H3O4P	Non-metal phosphates	NBIIXXVUZAFLBC-UHFFFAOYSA-N	O=P(O)(O)O
14	0.442	285.27829	stearic acid	[M+H]+	285.28	C18H36O2	Long-chain fatty acids	QIQXTHQIDYTFRH-UHFFFAOYSA-N	O=C(O)CCCCCCCCCCCCCCCCC
15	0.464	391.28308	Di(2-ethylhexyl)phthalate (DEHP)	[M+H]+	391.28427	C24H38O4	Benzoic acid esters	BJQHLKABXJIVAM-UHFFFAOYNA-N	CCCCC(CC)COC(C1=CC=CC=C1C(=O)OCC(CC)CCCC)=O
16	0.482	109.02829	p-Benzoquinone	[M+H]+	109.0284	C6H4O2	P-benzoquinones	AZQWKYJCGOJGHM-UHFFFAOYSA-N	O=C1C=CC(=O)C=C1
17	0.487	201.99016	Cysteine-S-sulfate	[M+H]+	201.98384	C3H7NO5S2	S-sulfo-L-cysteines	NOKPBJYHPHHWAN-REOHCLBHSA-N	C([C=H](C(=O)O)N)SS(=O)(=O)O
18	0.508	132.10263	Isoleucine (not validated)	[M+H]+	132.1028	C6H13NO2	Amino acids	AGPKZVBTJJNPAG-UHFFFAOYNA-N	O=C(O)C(N)C(C)CC
19	0.509	102.12761	Hexylamine	[M+H]+	102.12772	C6H15N	Monoalkylamines	BMVXCPBXGZKUPN-UHFFFAOYSA-N	CCCCCCN
20	0.542	186.01791	O-Phosphoserine	[M+H]+	186.0162	C3H8NO6P	L-alpha-amino acids	BZQFBWGGLXLEPQ-UHFFFAOYNA-N	O=C(O)C(N)COP(=O)(O)O
21	0.553	232.16878	Alpha-pyrrolidinovalerophenone	[M+H]+	232.17	C15H21NO	Alkyl-phenylketones	YDIIDRWHPFMLGR-UHFFFAOYSA-N	O=C(C=1C=CC=CC1)C(N2CCCC2)CCC
22	0.563	297.99988	(E)-2-(hydroxymethyl)-3-(3-oxo-5-propan-2-yl-4,5,6,7-tetrahydro-1H-2-benzofuran-4-yl)prop-2-enoic acid	[M+H]+	298.00226	C15H20O5	Terpene lactones	YWASZTJGPFRWMW-BJMVGYQFSA-N	O=C(O)C(=CC1C=2C(=O)OCC2CCC1C(C)C)CO
23	0.597	155.14288	Linalool	[M+H]+	155.14304	C10H18O	Acyclic monoterpenoids	CDOSHBSSFJOMGT-UHFFFAOYNA-N	OC(C=C)(C)CCC=C(C)C
24	0.606	113.06019	1,2-Cyclohexanedione	[M+H]+	113.05971	C6H8O2	Cyclic ketones	OILAIQUEIWYQPH-UHFFFAOYSA-N	O=C1C(=O)CCCC1
25	0.696	168.02489	Quinolinic Acid	[M+H]+	168.02502	C7H5NO4	Pyridinecarboxylic acids	GJAWHXHKYYXBSV-UHFFFAOYSA-N	O=C(O)C1=NC=CC=C1C(=O)O
26	0.7	255.93947	Triclopyr	[M+H]+	255.93295	C7H4Cl3NO3	Polyhalopyridines	REEQLXCGVXDJSQ-UHFFFAOYSA-N	OC(=O)COC1=C(Cl)C=C(Cl)C(Cl)=N1
27	0.701	303.02585	4-hydroxy-3-[(4-hydroxy-6-methyl-2-oxopyran-3-yl)methyl]-6-methylpyran-2-one	[M+H]+	303.02655	C13H12O6	Pyranones and derivatives	DVLLGHPCJPEUHA-UHFFFAOYSA-N	O=C1OC(=CC(O)=C1CC=2C(=O)OC(=CC2O)C)C
28	0.704	251.16005	Nardosinone	[M+H]+	251.16	C15H22O3	Sesquiterpenoids	KXGHHSIMRWPVQM-JWFUOXDNSA-N	O=C1C2=CCCC(C)C2(C)C3C(OOC3(C)C)C1
29	0.704	148.94887	Trifluoromethanesulfonic acid	[M-H]-	148.95258	CHF3O3S	PFSA	ITMCEJHCFYSIIV-UHFFFAOYSA-N	O=S(=O)(O)C(F)(F)F
30	0.705	278.94257	5-Chloro-perfluoropentanoic acid	[M-H]-	278.94647	C5HClF8O2	PFSA	VQKMJXAMQVPHHB-UHFFFAOYSA-N	O=C(O)C(F)(F)C(F)(F)C(F)(F)C(F)(F)Cl
31	0.707	147.97292	PFSM-perfluoroalkyl_sulfonamide	[M-H]-	147.96855	CH2F3NO2S	PFSA	KAKQVSNHTBLJCH-UHFFFAOYSA-N	O=S(=O)(N)C(F)(F)F
32	0.709	370.94284	PFCA-pentafluorosulfide	[M-H]-	370.94168	C5HF13O2S	PFSA	NDDSIRSIIHKJNN-UHFFFAOYSA-N	O=C(O)C(F)(F)C(F)(F)C(F)(F)C(F)(F)S(F)(F)(F)(F)F
33	0.71	226.05573	Synephrine hydrochloride	[M+H]+	226.06	C9H14ClNO2	1-hydroxy-2-unsubstituted benzenoids	COTCEGYSNTWJQV-UHFFFAOYSA-N	Cl.OC1=CC=C(C=C1)C(O)CNC
34	0.711	385.87479	[4-acetyloxy-2,5-dihydroxy-6-(hydroxymethyl)oxan-3-yl] (E)-3-(4-hydroxyphenyl)prop-2-enoate	[M+H]+	385.87271	C17H20O9	Coumaric acid esters	QXRDTLAXUJONPD-QPJJXVBHSA-N	O=C(OC1C(O)OC(CO)C(O)C1OC(=O)C)C=CC2=CC=C(O)C=C2
35	0.711	236.03975	Carnitine (Dl) Hydrochloride	[M+H]+	236.04503	C7H16ClNO3	Carnitines	JXXCENBLGFBQJM-UHFFFAOYSA-N	Cl.O=C([O-])CC(O)C[N+](C)(C)C
36	0.714	189.02379	6,7-dihydroxybenzofuran-3(2H)-one	[M+H]+	189.02	C8H6O4	Benzofurans	ZDHCVQNIRWDINX-UHFFFAOYSA-N	O=C1C2=CC=C(O)C(O)=C2OC1
37	0.714	308.25049	Pentadecanoyl Ethanolamide	[M+2H]2+	308.25	C17H35NO2	N-acylethanolamines	UHPXXKLAACDXGS-UHFFFAOYSA-N	O=C(NCCO)CCCCCCCCCCCCCC
38	0.715	231.03406	3,4-Dimethoxycinnamic Acid	[M+H]+	231.03662	C11H12O4	Coumaric acids and derivatives	HJBWJAPEBGSQPR-GQCTYLIASA-N	O=C(O)C=CC1=CC=C(OC)C(OC)=C1
39	0.716	284.01968	Procymidone	[M+H]+	284.02396	C13H11Cl2NO2	Phenylpiperidines	QXJKBPAVAHBARF-UHFFFAOYNA-N	CC12CC2(C)C(N(C3=CC(=CC(=C3)Cl)Cl)C1=O)=O
40	0.717	247.02939	columbianetin	[M+H]+	247.03241	C14H14O4	Angular furanocoumarins	YRAQEMCYCSSHJG-UHFFFAOYSA-N	O=C1OC2=C(C=C1)C=CC=3OC(CC32)C(O)(C)C
41	0.718	233.03185	MUCIC ACID	[M+H]+	233.02678	C6H10O8	Glucuronic acid derivatives	DSLZVSRJTYRBFB-DUHBMQHGSA-N	O=C(O)C(O)C(O)C(O)C(O)C(=O)O

42	0.721	283.02527	1,3,5,8-Tetrahydroxyxanthone	[M+H]+	283.01999	C13H8O6	Xanthones	MPXAWSABMVLIBU-UHFFFAOYSA-N	O=C1C=2C(O)=CC(O)=CC2OC=3C(O)=CC=C(O)C13
43	0.722	123.03981	2-methylcyclohexa-2,5-diene-1,4-dione	[M+H]+	123.04	C7H6O2	P-benzoquinones	VTWDKFNVVLAELH-UHFFFAOYSA-N	O=C1C=CC(=O)C(=C1)C
44	0.722	165.03516	Allicin + 2H (not validated, isomer of 328)	[M+H]+	165.03999	C6H12OS2	Allicin derivatives	XLVMWOFYTFNDKM-UHFFFAOYNA-N	O=S(CCC)SC=CC
45	0.722	360.93964	PFSA-unsaturated	[M-H]-	360.93979	C6HF11O3S	PFSA	WNQXBYFTFVMUCU-UHFFFAOYSA-N	O=S(=O)(O)C(F)(F)C(F)(F)C(F)(F)C(F)(F)C(F)=C(F)F
46	0.722	212.03979	PHOSPHOCREATINE	[M+H]+	212.03999	C4H10N3O5P	Alpha amino acids and derivatives	DRBBFCLWYRJSJZ-UHFFFAOYSA-N	O=C(O)CN(C(=NP(=O)(O)O)N)C
47	0.723	143.04996	5-(hydroxymethyl)pyrimidine-2,4-diol	[M+H]+	143.05	C5H6N2O3	Pyrimidones	JDBGXEHEIRGOBU-UHFFFAOYSA-N	OC1=NC=C(C(O)=N1)CO
48	0.723	125.03749	N,N-Dimethylsulfamide	[M+H]+	125.03793	C2H8N2O2S	Sulfuric acid diamides	QMHAHUAQAJVBIW-UHFFFAOYSA-N	CN(C)S(N)(=O)=O
49	0.724	167.01352	3-hydroxy-4-methoxy-2,3-dihydropyran-6-one	[M+H]+	167.01662	C6H8O4	Dihydropyranones	PBEXSRJMCVDBFK-UHFFFAOYSA-N	O=C1OCC(O)C(OC)=C1
50	0.724	146.1647	Spermidine	[M+H]+	146.16518	C7H19N3	Dialkylamines	ATHGHQPFGPMSJY-UHFFFAOYSA-N	NCCCCNCCCN
51	0.725	267.01202	Bauerine C	[M+H]+	267.01599	C12H8Cl2N2O	Beta carbolines	JFESWTBLTSUPGK-UHFFFAOYSA-N	O=C1NC=CC=2C3=CC=C(Cl)C(Cl)=C3N(C12)C
52	0.726	329.98038	(E)-N-[2-hydroxy-2-(4-hydroxyphenyl)ethyl]-3-(4-hydroxy-3-methoxyphenyl)prop-2-enamide	[M+H]+	329.98386	C18H19NO5	Hydroxycinnamic acids and derivatives	VJSCHQMOTSXAKB-YCRREMRBSA-N	OC(=NCC(O)C1=CC=C(O)C=C1)C=CC2=CC=C(O)C(OC)=C2
53	0.727	103.04988	(R)-4-aminoisoxazolidin-3-one	[M+2H]2+	103.05	C3H6N2O2	Alpha amino acids and derivatives	DYDCUQKUCUHJBH-UWTATZPHSA-N	O=C1NOCC1N
54	0.728	356.97989	4-[1-(1,3-benzodioxol-5-yl)propan-2-yl]-4,5-dimethoxy-2-prop-2-enylcyclohexa-2,5-dien-1-one	[M+H]+	356.98035	C21H24O5	Bicyclic monoterpenoids	SMOHLDSEWHACKE-UHFFFAOYSA-N	O=C1C=C(OC)C(OC)(C=C1CC=C)C(C)CC2=CC=C3OCOC3=C2
55	0.728	160.97531	OPFC-perfluoroalkyl_sulfate	[M-H]-	160.97256	C2H4F2O4S	PFSA	LAGMPONGXCPPKO-UHFFFAOYSA-N	O=S(=O)(O)OCC(F)F
56	0.728	221.04977	Pesticide2_Thidiazuron_C9H8N4OS_1-Phenyl-3-(1,2,3-thiadiazol-5-yl)urea	[M+H]+	221.049	C9H8N4OS	N-phenylureas	HFCYZXMHUIHAQI-UHFFFAOYSA-N	OC(=NC=1C=CC=CC1)NC=2SN=NC2
57	0.729	281.10614	Huperzine A	[M+H]+	281.10507	C15H18N2O	Quinolones and derivatives	ZRJBHWIHUMBLCN-UHFFFAOYSA-N	OC=1N=C2C(=CC1)C3(N)C(=CC)C(C=C(C)C3)C2
58	0.73	280.99149	methyl 5,6-diacetyloxy-10-hydroxy-2,4b,7,7,10a,12a-hexamethyl-12-methylidene-1,4,8-trioxo-4a,5,6,6a,9,10,10b,11-octahydronaphtho[1,2-	[M+H]+	280.99219	C30H40O11	Oxosteroids	FFXYBQSGDXRLHS-UHFFFAOYSA-N	O=C(OC1C(OC(=O)C)C2(C)C3C(=O)OC(C(=O)OC)(C(=O)C3(C(=C)CC2C4(C)C(O)CC(=O)C(C)(C)C14)C)C)C
59	0.732	265.11029	Thiamine	[M+H]+	265.11121	C12H17N4OS	Thiamines	JZRWCGZRTZMZEH-UHFFFAOYSA-N	CC1=C(CCO)SC=[N+]1CC1=CN=C(C)NC1=N
60	0.741	189.13409	Homoarginine	[M+H]+	189.1346	C7H16N4O2	Alpha amino acids	QUOGESRFPZDMMT-UHFFFAOYNA-N	O=C(O)C(N)CCCCNC(=N)N
61	0.743	188.17538	N8-Acetylspermidine	[M+H]+	188.17574	C9H21N3O	Carboximidic acids	FONIWJIDLJEJTL-UHFFFAOYSA-N	OC(=NCCCCNCCCN)C
62	0.744	329.07806	8-(2-hydroxy-1-methoxy-3-methylbut-3-enyl)-7-methoxychromen-2-one	[M+H]+	329.07858	C16H18O5	Coumarins and derivatives	DBPWCIPDCMVUFT-UHFFFAOYSA-N	O=C1OC2=C(C=C1)C=CC(OC)=C2C(OC)C(O)C(=C)C
63	0.744	133.09662	Ornithine	[M+H]+	133.09715	C5H12N2O2	Alpha amino acids	AHLPHDHHMVZTML-UHFFFAOYSA-N	O=C(O)C(N)CCCN
64	0.745	359.04245	3-O-acetylpadmatin	[M-H]-	359.04004	C18H16O8	7-O-methylated flavonoids	WWYQJKVSCKMFCP-MSOLQXFVSA-N	O=C(OC1C(=O)C2=C(O)C=C(OC)C=C2OC1C3=CC=C(O)C(O)=C3)C
65	0.748	311.06683	Cefditoren pivoxil	[M+H]+	311.06699	C25H28N6O7S3	Cephalosporins	AFZFFLVORLEPPO-OUOWMNRXSA-N	O=C(OCOC(=O)C(C)(C)C)C1=C(C=CC=2SC=NC2C)CSC3N1C(=O)C3NC(=O)C(=NOC)C=4N=C(SC4)N
66	0.75	352.09363	feruloyltyramine	[M+H]+	352.09457	C18H19NO4	Methoxyphenols	NPNNKDMSXVRADT-WEVVVXLNSA-N	OC(=NCCC1=CC=C(O)C=C1)C=CC2=CC=C(O)C(OC)=C2
67	0.758	175.11835	Arginine	[M+H]+	175.119	C6H14N4O2	Arginine and derivatives	ODKSFYDXXFIFQN-UHFFFAOYSA-N	O=C(O)C(N)CCCN=C(N)N
68	0.758	371.04456	Norstictic acid	[M-H]-	371.03799	C18H12O9	Diarylethers	IEVVSJFLBYOUCJ-UHFFFAOYSA-N	O=CC1=C(O)C=C(C=2C(=O)OC3=C(OC12)C4=C(C(=O)OC4O)C(O)=C3C)C
69	0.774	189.1604	NEPSILON,NEPSILON,NEPSILON-TRIMETHYLLYSINE	[M+H]+	189.16	C9H20N2O2	Alpha amino acids	MXNRLFUSFKVQSK-UHFFFAOYSA-N	O=C([O-])C(N)CCCC[N+](C)(C)C
70	0.778	353.20703	Hydroxyprogesterone	[M+H]+	353.2052	C21H30O3	Gluco/mineralocorticoids, progestogins and	DBPWSSGDRRHUNT-UHFFFAOYSA-N	O=C1C=C2CCC3C(CCC4(C)C3CCC4(O)C(=O)C)C2(C)CC1
71	0.783	341.02631	Pesticide4_Pyrimethanil_C12H13N3_2-Pyrimidinamine, 4,6-dimethyl-	[M-H]-	341.026	C6H9N3	Aminopyrimidines and derivatives	IDQNBVFPZMCDDN-UHFFFAOYSA-N	N=C1N=C(C=C(N1)C)C
72	0.786	429.23834	Irbesartan	[M+H]+	429.23975	C25H28N6O	Biphenyls and derivatives	YOSHYTLCDANDAN-UHFFFAOYSA-N	CCCCC1=NC2(CCCC2)C(N1CC3=CC=C(C=C3)C4=CC=CC=C4C5=NNN=N5)=O
73	0.787	469.13022	Galapagin	[M-H]-	469.129	C20H24O10	Phenolic glycosides	NJRLDXGREKHXGV-IVABAYMNSA-N	O=C(OCC1OC(OC=2C(=C(O)C=3C(=O)C=C(OC3C2C)C)C)C(O)C(O)C1O)C
74	0.787	257.06497	ONONETIN	[M-H]-	257.06015	C15H14O4	Stilbenes	XHBZOAYMBBUURD-UHFFFAOYSA-N	O=C(C1=CC=C(O)C=C1O)CC2=CC=C(OC)C=C2
75	0.789	426.98535	(Perfluoroheptyl)ethanoic acid	[M-H]-	426.98209	C9H3F15O2	PFSA	WSEQBIVQCARAMW-UHFFFAOYSA-N	O=C(O)CC(F)(F)C(F)(F)C(F)(F)C(F)(F)C(F)(F)C(F)(F)C(F)(F)F
76	0.79	266.15857	"Mirtazapine (Remeron, Avanza)"	[M+H]+	266.16501	C17H19N3	Piperazinoazepines	RONZAEMNMFQXRA-UHFFFAOYSA-N	N=1C=CC=C2C1N3CCN(C)CC3C=4C=CC=CC4C2
77	0.79	173.10349	L-(+)-Arginine	[M-H]-	173.1044	C6H14N4O2	L-alpha-amino acids	ODKSFYDXXFIFQN-BYPYZUCNSA-N	N[C=H](CCCNC(N)=N)C(O)=O
78	0.794	740.6745	Glyceryl trimyristate	[M+H]+	740.677	C45H86O6	Triacylglycerols	DUXYWXYOBMKGIN-UHFFFAOYSA-N	O=C(OCC(OC(=O)CCCCCCCCCCCCC)COC(=O)CCCCCCCCCCCCC)CCCCCCCCCCCC
79	0.795	319.01706	Medicagol	[M+H]+	319.01999	C16H8O6	Coumestans	URMVEUAWRUQHON-UHFFFAOYSA-N	O=C1OC=2C=C(O)C=CC2C=3OC4=CC=5OCOC5C=C4C13
80	0.796	307.14911	Vincanidine	[M-H]-	307.1452	C19H20N2O2	Alkaloids	JDOFCMASVRMYJU-UHFFFAOYNA-N	CC=C1CN2CCC34C2CC1C(C=O)=C3NC1=C4C=CC=C1O
81	0.797	216.01375	2-amino-3-prop-2-enylsulfinylpropanoic acid	[M+H]+	216.00912	C6H11NO3S	Alpha amino acids	XUHLIQGRKRUKPH-UHFFFAOYSA-N	O=C(O)C(N)CS(=O)CC=C
82	0.797	292.96927	Daidzein	[M+H]+	292.96872	C15H10O4	Isoflavones	ZQSIJRDFPHDXIC-UHFFFAOYSA-N	O=C1C(=COC2=CC(O)=CC=C12)C=3C=CC(O)=CC3
83	0.797	320.03976	Epalrestat	[M+H]+	320.04099	C15H13NO3S2	Alpha amino acids and derivatives	CHNUOJQWGUIOLD-NFZZJPOKSA-N	O=C(O)CN1C(=S)SC(=CC(=CC=2C=CC=CC2)C)C1=O
84	0.797	420.95001	Fipronil-sulfide	[M+H]+	420.95108	C12H4Cl2F6N4S	Phenylpyrazoles	FQXWEKADCSXYOC-UHFFFAOYSA-N	NC1=C(SC(F)(F)F)C(=NN1C1=C(Cl)C=C(C=C1Cl)C(F)(F)F)C#N

85	0.797	387.18439	Fiscalin B_130006	[M+H]+	387.18201	C23H22N4O2	Quinazolines	DNPSVQYQTPYSPH-VQTJNVASSA-N	O=C1NC(C2=NC=3C=CC=CC3C(=O)N2C1CC4=CNC=5C=CC=CC54)C(C)C
86	0.797	197.10014	Gramine	[M+H]+	197.10001	C11H14N2	3-alkylindoles	OCDGBSUVYYYVKQZ-UHFFFAOYSA-N	C=1C=CC2=C(C1)NC=C2CN(C)C
87	0.797	425.14035	Icariside F2	[M+H]+	425.14001	C18H26O10	O-glycosyl compounds	NJMQSVWMCODQIP-FQXXIRCGSA-N	OCC1(O)COC(OCC2OC(OCC=3C=CC=CC3)C(O)C(O)C2O)C1O
88	0.797	463.09662	Rutarin	[M+H]+	463.09546	C20H24O10	Coumarin glycosides	JWWFVRMFYKPZNE-VVIWCBLHSA-N	O=C1OC=2C(OC3OC(CO)C(O)C(O)C3O)=C4OC(CC4=CC2C=C1)C(O)(C)C
89	0.798	128.95871	PFCA-Cl	[M-H]-	128.95604	C2HClF2O2	PFSA	OAWAZQITIZDJRB-UHFFFAOYSA-N	O=C(O)C(F)(F)Cl
90	0.8	193.03453	DL-Isocitric acid	[M+H]+	193.03429	C6H8O7	Tricarboxylic acids and derivatives	ODBLHEXUDAPZAU-UHFFFAOYNA-N	OC(C(CC(O)=O)C(O)=O)C(O)=O
91	0.803	253.02815	Thiacloprid	[M+H]+	253.03091	C10H9ClN4S	2-halopyridines	HOKKPVIRMVDYPB-GXDHUFHOSA-N	ClC1=NC=C(CN2CCSC2=NC#N)C=C1
92	0.804	290.84595	dehydrocurvularin	[M+H]+	290.85229	C16H18O5	Macrolides and analogues	AVIRMQMUBGNCKS-GQCTYLIASA-N	O=C1C=CCCCC(OC(=O)CC=2C=C(O)C=C(O)C12)C
93	0.805	168.07693	N(PAI)-METHYL-L-HISTIDINE	[M-H]-	168.077	C7H11N3O2	Histidine and derivatives	BRMWTNUJHUMWMS-UHFFFAOYSA-N	O=C(O)C(N)CC=1N=CN(C1)C
94	0.808	375.12671	Anhydrohaloxindole A	[M+H]+	375.12601	C21H21ClN2O	Isoindolones	ZHHYANZIARLNFJ-FWKFCYAVSA-N	[C-]#[N+]C1C2=C3C(=O)NC4=CC=CC(=C43)C(C)(C)C2CC(C1)C1(C=C
95	0.809	100.0397	1-AMINOCYCLOPROPANE-1-CARBOXYLATE	[M-H]-	100.04	C4H7NO2	Alpha amino acids	PAJPWUMXBYXFCZ-UHFFFAOYSA-N	O=C(O)C1(N)CC1
96	0.815	224.07703	Flufenacet OXA	[M-H]-	224.07285	C11H12FNO3	Alpha amino acids and derivatives	FFKNXXCOXIZLJD-UHFFFAOYSA-N	CC(C)N(C(=O)C(O)=O)C1=CC=C(F)C=C1
97	0.823	154.06143	Histidine	[M-H]-	154.06219	C6H9N3O2	Histidine and derivatives	HNDVDQJCIGZPNO-UHFFFAOYNA-N	O=C(O)C(N)CC1=CN=CN1
98	0.832	449.06415	Archangelicine	[M+H]+	449.06046	C24H26O7	Angular furanocoumarins	RVGGCRQPGKFZDS-AKRYRNCVSA-N	O=C1OC2=C(C=C1)C=CC=3OC(C(OC(=O)C(=CC)C)C32)C(OC(=O)C(=CC)C)(C)C
99	0.832	333.06744	methyl 2-{7-hydroxy-11-methyl-2,9-dioxo-12,15-dioxatetracyclo[8.4.1.0?,??.0?,?]pentadeca-3,5,7-trien-13-yl}acetate	[M+H]+	333.06555	C17H16O7	Naphthopyranones	IHXLRPOTFALLKF-UHFFFAOYSA-N	O=C(OC)CC1OC(C)C23OC3(C(=O)C=4C=CC=C(O)C4C2=O)C1
100	0.833	254.01785	2-(2-bromophenyl)-4,4-dimethyl-4,5-dihydrooxazole	[M+H]+	254.01801	C11H12BrNO	Bromobenzenes	XLCYXINIUJNTPF-UHFFFAOYSA-N	BrC=1C=CC=CC1C2=NC(C)(C)CO2
101	0.833	827.2616	Pentasaccharides (Hex-Hex-Hex-Hex-Hex)	[M-H]-	827.26562	C30H52O26	Sugars	FTNIPWXXIGNQQF-UHFFFAOYNA-N	OCC5OC(OC1C(O)C(O)C(OC1(CO))OC2C(O)C(O)C(OC2(CO))OC3C(O)C(O)C(OC3(CO))OC4C(O)C(O)C(O)OC4(CO))C(O)C(O)C5(O)
102	0.833	200.0679	Willardiine	[M+H]+	200.06659	C7H9N3O4	L-alpha-amino acids	FACUYWPMDKTVFU-UHFFFAOYNA-N	O=C(O)C(N)CN1C=CC(O)=NC1=O
103	0.835	336.13953	Linezolide	[M-H]-	336.13651	C16H20FN3O4	Phenylmorpholines	TYZROVQLWOKYKF-UHFFFAOYNA-N	O=C1OC(CN=C(O)C)CN1C2=CC=C(C(F)=C2)N3CCOCC3
104	0.835	299.11566	rhodioloside	[M-H]-	299.11362	C14H20O7	O-glycosyl compounds	ILRCGYURZSFMEG-RKQHYHRCSA-N	OC1=CC=C(C=C1)CCOC2OC(CO)C(O)C(O)C2O
105	0.835	285.10019	Salicin	[M-H]-	285.09796	C13H18O7	Phenolic glycosides	NGFMICBWJRZIBI-UHFFFAOYNA-N	OCC=1C=CC=CC1OC2OC(CO)C(O)C(O)C2O
106	0.836	465.03827	4,9-dimethoxy-7-(perfluorobutyl)-5H-furo[3,2-g]chromen-5-one	[M+H]+	465.04001	C17H9F9O5	Furanochromones	BVUOXVSHTGNMRR-UHFFFAOYSA-N	O=C1C=C(OC=2C(OC)=C3OC=CC3=C(OC)C12)C(F)(F)C(F)(F)C(F)(F)C(F)(F)F
107	0.836	447.15421	Benzyl alcohol + Hex-Pen	[M-H]-	447.1517	C18H26O10	Alkyl benzyl hexosides	NJMQSVWMCODQIP-UHFFFAOYNA-N	OCC3(O)(COC(OCC2OC(OCC1=CC=CC=C1)C(O)C(O)C2(O))C3(O))
108	0.838	241.09131	Lapachol	[M-H]-	241.089	C15H14O3	Vitamin K compounds	CIEYTVIYYGTCCI-UHFFFAOYSA-N	O=C1C(O)=C(C(=O)C=2C=CC=CC12)CC=C(C)C
109	0.841	449.168	Cratoxyarborenone E	[M+H]+	449.16705	C24H26O6	4-prenylated xanthones	CJBZJZGULKATPY-UHFFFAOYSA-N	O=C1C2=CC(O)=C(OC)C(=C2OC=3C1=C(O)C=C(O)C3CC=C(C)C)CC=C(C)C
110	0.842	201.01534	esculetin	[M+H]+	201.01582	C9H6O4	6,7-dihydroxycoumarins	ILEDWLMCKZNDJK-UHFFFAOYSA-N	O=C1OC=2C=C(O)C(O)=CC2C=C1
111	0.843	337.16953	(2R,3R,4S,5S,6R)-2-[(2E)-4-ethenyl-2,5-dimethylhexa-2,5-dienoxy]-6-(hydroxymethyl)oxane-3,4,5-triol	[M+H]+	337.16882	C16H26O6	Fatty acyl glycosides of mono- and disaccharides	FOWZPEXPOVXJSN-MIYBPCGNSA-N	OCC1OC(OCC(=CC(C=C)C(=C)C)C)C(O)C(O)C1O
112	0.844	282.04477	DCOIT	[M+H]+	282.04807	C11H17Cl2NOS	Aryl chlorides	PORQOHRXAJJKGK-UHFFFAOYSA-N	CCCCCCCCN1C(C(=C(Cl)S1)Cl)=O
113	0.845	867.23413	(3S,4R)-7-hydroxy-3-[(3S,4R)-7-hydroxy-4-(4-hydroxyphenyl)-2-oxo-5-[(2S,3R,4S,5S,6R)-3,4,5-trihydroxy-6-(hydroxymethyl)oxan-2-yl]oxy-3,4-	[M+H]+	867.23419	C42H42O20	Coumarin glycosides	AYJLVEAOIKOWIP-DUKVQSBLSA-N	O=C1OC2=CC(O)=CC(OC3OC(CO)C(O)C(O)C3O)=C2C(C4=CC=C(O)C=C4)C1C5C(=O)OC6=CC(O)=CC(OC7OC(CO)C(O)C(O)C7O)=C6C5C8
114	0.845	284.09711	Letrozol	[M-H]-	284.09418	C17H11N5	Diphenylmethanes	HPJKCIUCZWXJDR-UHFFFAOYSA-N	N#CC1=CC=C(C=C1)C(C2=CC=C(C#N)C=C2)N3N=CN=C3
115	0.845	104.03451	Serine	[M-H]-	104.03532	C3H7NO3	Serine and derivatives	MTCFGRXMJLQNBG-UHFFFAOYNA-N	O=C(O)C(N)CO
116	0.845	118.05007	Threonine	[M-H]-	118.05096	C4H9NO3	L-alpha-amino acids	AYFVYJQAPQTCCC-UHFFFAOYNA-N	O=C(O)C(N)C(O)C
117	0.846	380.09332	(4-chlorophenyl)(7,8-dimethoxy-3-methyl-2,3-dihydro-1H-benzo[d]azepin-4-yl)methanone	[M+H]+	380.10001	C20H20ClNO3	Benzazepines	XWOLWJSHOPYCNG-UHFFFAOYSA-N	O=C(C1=CC=C(Cl)C=C1)C2=CC=3C=C(OC)C(OC)=CC3CCN2C
118	0.848	319.16055	Fluvoxamine	[M+H]+	319.16278	C15H21F3N2O2	Trifluoromethylbenzenes	CJOFXWAVKWHTFT-UHFFFAOYSA-N	COCCCCC(C1=CC=C(C=C1)C(F)(F)F)=NOCCN
119	0.848	145.06253	Glutamine (D)	[M-H]-	145.06186	C5H10N2O3	D-alpha-amino acids	ZDXPYRJPNDTMRX-GSVOUGTGSA-N	O=C(O)C(N)CCC(=N)O
120	0.848	74.02409	GLYCINE	[M-H]-	74.0242	C2H5NO2	Alpha amino acids	DHMQDGOQFOQNFH-UHFFFAOYSA-N	O=C(O)CN
121	0.848	131.04536	L-ASPARAGINE	[M-H]-	131.04601	C4H8N2O3	Asparagine and derivatives	DCXYFEDJOCDNAF-UHFFFAOYSA-N	O=C(O)C(N)CC(=O)N
122	0.851	129.06555	5,6-Dihydro-5-methyluracil	[M+H]+	129.06586	C5H8N2O2	Hydropyrimidines	NBAKTGXDIBVZOO-UHFFFAOYNA-N	OC=1N=C(O)C(C)CN1
123	0.852	443.13913	Diferuloyl glycerol	[M-H]-	443.1347	C23H24O9	Ferulic acid and derivatives	PATJZXBBUQEFOY-UHFFFAOYSA-N	O=C(OCC(O)COC(=O)C=CC=1C=CC(O)=C(OC)C=1)C=CC=2C=CC(O)=C(OC)C=2
124	0.852	216.09775	Securinine	[M-H]-	216.103	C13H15NO2	Indolizidines	SWZMSZQQJRKFBP-WZRBSPASSA-N	O=C1OC23C(C=CC(N4CCCCC42)C3)=C1
125	0.852	221.04175	Syringic acid	[M+Na]+	221.03999	C9H10O5	Gallic acid and derivatives	JMSVCTWVEWCHDZ-UHFFFAOYSA-N	O=C(O)C1=CC(OC)=C(O)C(OC)=C1
126	0.853	244.09224	Cytidine	[M+H]+	244.0928	C9H13N3O5	Pyrimidine nucleosides	UHDGCWIWMRVCDJ-XVFCMESISA-N	c1cn([C=H]2[C=H]([C=H]([C=H](CO)O2)O)O)c(nc1=N)O
127	0.853	193.0294	L-citrulline	[M+H]+	193.02893	C6H13N3O3	L-alpha-amino acids	RHGKLRLOHDJJDR-BYPYZUCNSA-N	O=C(O)C(N)CCCNC(=N)O

128	0.854	189.0867	"LL-2,6-DIAMINOHEPTANEDIOATE"	[M-H]-	189.088	C7H14N2O4	Alpha amino acids	GMKMEZVLHJARHF-UHFFFAOYSA-N	O=C(O)C(N)CCCC(N)C(=O)O
129	0.854	307.11316	5,13,15-trihydroxy-9-methyl-10-oxabicyclo[10.4.0]hexadeca-1(12),13,15-triene-3,11-dione	[M-H]-	307.11871	C16H20O6	Macrolides and analogues	TXFYYWHRTDECTR-UHFFFAOYSA-N	O=C1OC(C)CCCC(O)CC(=O)CC=2C=C(O)C=C(O)C12
130	0.854	457.16702	7-O-Ethylmorroniside	[M+H]+	457.16901	C19H30O11	O-glycosyl compounds	IRKFOLIBBQDADK-LEYKIWRXSA-N	O=C(OC)C1=COC(OC2OC(CO)C(O)C(O)C2O)C3C(OC(OCC)CC13)C
131	0.854	293.09772	cyclopenin	[M-H]-	293.09317	C17H14N2O3	1,4-benzodiazepines	APLKWZASYUZSBL-PBHICJAKSA-N	O=C1C=2C=CC=CC2N=C(O)C3(OC3C=4C=CC=CC4)N1C
132	0.854	365.10464	Melibiose	[M+Na]+	365.10544	C12H22O11	O-glycosyl compounds	DLRVVLDZNNYCBX-ABXHMFFYSA-N	OCC1OC(OCC2OC(O)C(O)C(O)C2O)C(O)C(O)C1O
133	0.854	250.09183	Muramic acid	[M-H]-	250.09323	C9H17NO7	Sugar acids and derivatives	MSFSPUZXLOGKHJ-UHFFFAOYNA-N	O=C(O)C(OC1C(O)C(OC(O)C1N)CO)C
134	0.854	229.08705	Xanthyletin	[M+H]+	229.086	C14H12O3	Linear pyranocoumarins	QOTBQNVNUBKJMS-UHFFFAOYSA-N	O=C1OC=2C=C3OC(C=CC3=CC2C=C1)(C)C
135	0.855	385.12561	[(3aS,4S,5S,6E,10Z,11aR)-6-formyl-5-hydroxy-10-(hydroxymethyl)-3-methylidene-2-oxo-3a,4,5,8,9,11a-hexahydrocyclodeca[b]furan-4-yl] 2-	[M+H]+	385.12576	C19H22O7	Germacranolides and derivatives	HQPSNGXRJBMMAL-JVWKVRNCSA-N	O=CC1=CCCC(=CC2OC(=O)C(=C)C2C(OC(=O)C(=C)C)C1O)CO
136	0.855	88.03969	L-ALANINE	[M-H]-	88.0399	C3H7NO2	Alanine and derivatives	QNAYBMKLOCPYGJ-UWTATZPHSA-N	O=C(O)C(N)C
137	0.855	164.03775	Methioninesulfoxide	[M-H]-	164.03868	C5H11NO3S	Alpha amino acids	QEFRNWWLZKMPFJ-UHFFFAOYNA-N	O=C(O)C(N)CCS(=O)C
138	0.856	220.08148	N-Acetylglucosamine	[M-H]-	220.08266	C8H15NO6	N-acyl-alpha-hexosamines	OVRNDRQMDRJTHS-UHFFFAOYNA-N	OC(=NC1C(O)OC(CO)C(O)C1O)C
139	0.857	172.07118	Metronidazole	[M+H]+	172.07167	C6H9N3O3	Nitroimidazoles	VAOCPAMSLUNLGC-UHFFFAOYSA-N	O=N(=O)C1=CN=C(N1CCO)C
140	0.858	527.15503	Flavone base + 3O, 2MeO, O-guaiacylglycerol	[M+H]+	527.15509	C27H26O11	Flavone O-glycosides	WXNJNHFYIWEHIL-UHFFFAOYNA-N	O=C1C=C(OC2=CC(O)=CC(O)=C12)C=4C=C(OC)C(OC(CO)C(O)C=3C=CC(O)=C(OC)C=3)=C(OC)C=4
141	0.858	321.12939	Indole-3-acetyl-L-phenylalanine	[M-H]-	321.12448	C19H18N2O3	Amino acids	BUGQHORRADGONS-UHFFFAOYNA-N	OC(CC1=CNC2=CC=CC=C12)=NC(CC1=CC=CC=C1)C(O)=O
142	0.858	106.04941	L-Serine	[M+H]+	106.04987	C3H7NO3	Serine and derivatives	MTCFGRXMJLQNBG-REOHCLBHSA-N	C([C=H](C(=O)O)N)O
143	0.859	312.16492	Xanthinol	[M+H]+	312.16663	C13H21N5O4	Xanthines	DSFGXPJYDCSWTA-UHFFFAOYNA-N	O=C1C2=C(N=CN2CC(O)CN(C)CCO)N(C(=O)N1C)C
144	0.86	156.04173	2-amino-4-hydroxypyrimidine-5-carboxylic acid	[M+H]+	156.03999	C5H5N3O3	Pyrimidinecarboxylic acids	GBADBFAXAWZGHM-UHFFFAOYSA-N	O=C(O)C1=CN=C(N=C1O)N
145	0.86	368.11847	2-Chloro-N6-cyclopentyladenosine	[M-H]-	368.1131	C15H20ClN5O4	Purine nucleosides	XSMYYYQVWPZWIZ-UHFFFAOYNA-N	ClC=1N=C(NC2CCCC2)C=3N=CN(C3N1)C4OC(CO)C(O)C4O
146	0.86	147.07611	Glutamine	[M+H]+	147.07642	C5H10N2O3	Alpha amino acids	ZDXPYRJPNDTMRX-UHFFFAOYNA-N	NC(CCC(O)=N)C(O)=O
147	0.861	190.07031	N-ACETYL-DL-GLUTAMIC ACID	[M+H]+	190.07001	C7H11NO5	Glutamic acid and derivatives	RFMMMVDNIPUKGG-YFKPBYRVSA-N	O=C(O)CCC(NC(=O)C)C(=O)O
148	0.862	412.1445	(S,R)-Nosapine	[M-H]-	412.14017	C22H23NO7	Phthalide isoquinolines	AKNNEGZIBPJZJG-MSOLQXFVSA-N	O=C1OC(C2=CC=C(OC)C(OC)=C12)C3C4=C(OC)C=5OCOC5C=C4CCN3C
149	0.862	132.02942	Aspartic acid	[M-H]-	132.03023	C4H7NO4	Aspartic acid and derivatives	CKLJMWTZIZZHCS-UHFFFAOYNA-N	O=C(O)CC(N)C(=O)O
150	0.862	298.10217	Erythraline	[M+H]+	298.10001	C18H19NO3	Erythrinanes	TVOFUERNMZTYRM-UHFFFAOYSA-N	O1C=2C=C3C(=CC2OC1)C45C(C=CC(OC)C4)=CCN5CC3
151	0.863	265.09296	5,9-dihydroxy-5,7,7-trimethyl-4,5a,6,8,8a,9-hexahydro-1H-azuleno[5,6-c]furan-3-one	[M-H]-	265.09396	C15H22O4	Terpene lactones	UUZWMJQAEYBHAO-UHFFFAOYSA-N	O=C1OCC2=C1CC(O)(C)C3CC(C)(C)CC3C2O
152	0.864	110.05975	4-Aminophenol	[M+H]+	110.06004	C6H7NO	Aniline and substituted anilines	PLIKAWJENQZMHA-UHFFFAOYSA-N	c1cc(ccc1N)O
153	0.865	85.04746	3-Amino-1,2,4-triazole	[M+H]+	85.05087	C2H4N4	Triazoles	KLSJWNVTNUYHDU-UHFFFAOYSA-N	C1=NC(=N)NN1
154	0.867	517.13977	5,7-dihydroxy-6-[3,4,5-trihydroxy-6-(hydroxymethyl)oxan-2-yl]-8-[3,4,5-trihydroxy-6-(hydroxymethyl)oxan-2-yl]oxychromen-2-one	[M-H]-	517.13855	C21H26O15	Coumarin glycosides	YRVGYJMSCQFOCA-UHFFFAOYSA-N	O=C1OC=2C(OC3OC(CO)C(O)C(O)C3O)=C(O)C(=C(O)C2C=C1)C4OC(CO)C(O)C(O)C4O
155	0.867	173.09402	N-alpha-Acetyl-L-ornithine	[M-H]-	173.09317	C7H14N2O3	N-acyl-L-alpha-amino acids	JRLGPAXAGHMNOL-LURJTMIESA-N	CC(O)=N[C=H](CCCN)C(O)=O
156	0.868	222.09625	N-Acetyl-D-glucosamine	[M+H]+	222.09721	C8H15NO6	N-acyl-alpha-hexosamines	OVRNDRQMDRJTHS-RTRLPJTCSA-N	CC(=N[C=H]1[C=H]([C=H]([C=H](CO)OC1O)O)O)O
157	0.868	168.06749	Norepinephrine	[M-H]-	168.06662	C8H11NO3	Catechols	SFLSHLFXELFNJZ-UHFFFAOYNA-N	OC1=CC=C(C=C1O)C(O)CN
158	0.869	527.20825	[(1aR,1bS,2S,5aS,6R,6aS)-5a-hydroxy-1a-methyl-2-[(2S,3R,4S,5S,6R)-3,4,5-trihydroxy-6-(hydroxymethyl)oxan-2-yl]oxy-1b,2,6,6a-	[M-H]-	527.21338	C25H36O12	Terpene glycosides	PRWWZXHOHQRKDQ-SVDQWWJBSA-N	O=C(OC1C2OC2(C)C3C(OC=CC13O)OC4OC(CO)C(O)C(O)C4O)C(=CCC(O)(C=C)C)C
159	0.869	399.07019	2-amino-5-[2-[[(3S)-2,3-dihydroxy-2-[(1S)-1-hydroxyethyl]butanoyl]oxymethyl]anilino]-5-oxopentanoic acid	[M+H]+	399.06964	C18H26N2O8	Glutamine and derivatives	NFINSJKQOBJWEB-WAQLSPKVSA-N	O=C(O)C(N)CCC(O)=NC=1C=CC=CC1COC(=O)C(O)(C(O)C)C(O)C
160	0.869	369.18729	Gardnerine	[M-H]-	369.18198	C20H24N2O2	Alkaloids	RBALEJFQJCAPLN-UHFFFAOYNA-N	COC1=CC=C2C(NC3=C2CC2C(CO)C4CC3N2CC4=CC)=C1
161	0.869	383.16586	Hydroxygardnutine	[M-H]-	383.16125	C20H22N2O3	Alkaloids	OUONWRIEOZGMSQ-UHFFFAOYNA-N	COC1=CC=C2C(NC3=C2C2OCC4C2N2CC(=CCO)C4CC32)=C1
162	0.869	329.10806	Pesticide4_Halofenozide_C18H19ClN2O2_N'-Benzoyl-4-chloro-N'-(2-methyl-2-propanyl)benzohydrazide	[M-H]-	329.10699	C18H19ClN2O2	4-halobenzoic acids and derivatives	CNKHSLKYRMDDNQ-UHFFFAOYSA-N	O=C(NN(C(=O)C=1C=CC=CC1)C(C)(C)C)C2=CC=C(Cl)C=C2
163	0.87	477.14401	Isorhamnetin 3-galactoside	[M-H]-	477.14212	C22H22O12	Flavonoid-3-O-glycosides	CQLRUIIRRZYHHS-UHFFFAOYSA-N	O=C1C(OC2OC(CO)C(O)C(O)C2O)=C(OC=3C=C(O)C=C(O)C13)C=4C=CC(O)=C(OC)C4
164	0.871	263.17041	Oxysophocarpine	[M+H]+	263.17001	C15H22N2O2	Matrine alkaloids	QMGGMESMCJCABO-LHDUFFHYSA-N	O=C1C=CCC2N1CC3CCC[N+]4([O-])CCCC2C34
165	0.871	440.17328	Quinadoline B_130007	[M+H]+	440.173	C25H21N5O3	Pyridopyrimidines	GACSRZKCZGCRJX-PDODLRMBSA-N	O=C1C=2C=CC=CC2N=C3N1C4C(=O)NC3C5(C=6C=CC=CC6N7C(=O)C8N(CCC8)C75)C4
166	0.872	173.09155	Glycyl-L-proline	[M+H]+	173.09207	C7H12N2O3	Dipeptides	KZNQNBZMBZJQJO-YFKPBYRVSA-N	NCC(=O)N1CCC[C=H]1C(O)=O
167	0.872	292.14835	PiperlotineC	[M+H]+	292.14999	C16H21NO4	Cinnamic acids and derivatives	TYFKYDTUEMTUNY-VOTSOKGWSA-N	O=C(C=CC1=CC(OC)=C(OC)C(OC)=C1)N2CCCC2
168	0.872	309.13043	Toddalolactone	[M+H]+	309.13	C16H20O6	Coumarins and derivatives	GLWPLQBQHWYKRK-CYBMUJFWSA-N	O=C1OC2=CC(OC)=C(C(OC)=C2C=C1)CC(O)C(O)(C)C
169	0.873	234.09596	1-Isothiocyanato-8-(methylsulfinyl)-octane	[M+H]+	234.09808	C10H19NOS2	Sulfoxides	BCRXKWOQVFKZAG-UHFFFAOYNA-N	CS(=O)CCCCCCCCN=C=S
170	0.873	146.09209	4-Guanidinobutyric acid	[M+H]+	146.09241	C5H11N3O2	Gamma amino acids and derivatives	TUHVEAJXIMEOSA-UHFFFAOYSA-N	O=C(O)CCCNC(=N)N

171	0.874	283.10229	Glycitein	[M-H]-	283.10272	C16H12O5	Isoflavones	DXYUAIFZCFRPTH-UHFFFAOYSA-N	O=C1C(=COC2=CC(O)=C(OC)C=C12)C=3C=CC(O)=CC3
172	0.875	537.16602	Selaginpulvilin K	[M-H]-	537.16901	C36H26O5	Fluorenes	UTTATNZIWSODLT-UHFFFAOYSA-N	O=CC1=CC=C2C=3C=CC(O)=CC3C(C4=CC=C(OC)C=C4)(C5=CC=C(OC)C=C5)C2=C1C#CC6=CC=C(O)C=C6
173	0.875	349.13431	Torsemide (Demadex)	[M+H]+	349.133	C16H20N4O3S	Pyridinesulfonamides	NGBFQHCMQULJNZ-UHFFFAOYSA-N	O=C(NC(C)C)NS(=O)(=O)C=1C=NC=CC1NC2=CC=CC(=C2)C
174	0.876	191.10188	2,6-diaminopimelic acid	[M+H]+	191.10265	C7H14N2O4	Alpha amino acids	GMKMEZVLHJARHF-UHFFFAOYSA-N	C(CC(C(=O)O)N)CC(C(=O)O)N
175	0.876	289.18607	4-((1R,3S,5r,7r)-5,7-dimethyl-1,3-diazaadamantan-2-yl)-2-methoxyphenol	[M+H]+	289.19	C17H24N2O2	Methoxyphenols	LATQZYGIAROYHX-UHFFFAOYSA-N	OC1=CC=C(C=C1OC)C2N3CC4(C)CN2CC(C)(C3)C4
176	0.877	346.05377	2'-DEOXYGUANOSINE 5'-MONOPHOSPHATE	[M-H]-	346.05499	C10H14N5O7P	Purine 2'-deoxyribonucleoside	LTFMZDNNPPEQNG-UHFFFAOYSA-N	O=C1N=C(N)NC2=C1N=CN2C3OC(COP(=O)(O)O)C(O)C3
177	0.877	346.12225	Omeprazole (Prilosec)	[M+H]+	346.12201	C17H19N3O3S	Sulfinylbenzimidazoles	SUBDBMMJDZJVOS-UHFFFAOYSA-N	O=S(C1=NC=2C=C(OC)C=CC2N1)CC3=NC=C(C(OC)=C3C)C
178	0.877	288.11932	OPHTHALMIC ACID	[M-H]-	288.12	C11H19N3O6	Peptoid-peptide hybrids	SPLUHYDIQKURQW-UHFFFAOYSA-N	O=C(O)CN(C(=O)CCC(N)C(=O)O)C(=O)C(N)CC
179	0.877	138.05437	Trigonelline	[M+H]+	138.05499	C7H7NO2	Alkaloids and derivatives	WWNNZCOKKKDOPX-UHFFFAOYSA-N	O=C([O-])C1=CC=C[N+](=C1)C
180	0.878	398.1691	Lipothiazole A	[M+H]+	398.17401	C18H27N3O5S	Peptides	XSLDIFFYKBQCGW-VFNNOXKTSA-N	O=C(O)C(N=C(O)CN=C(O)C=CC=1N=C(SC1)CCCCCCC)CO
181	0.878	247.175	Santonin	[M+H]+	247.17221	C15H18O3	Eudesmanolides, secoeudesmanolides, and	XJHDMGJURBVLLE-BOCCBSBMSA-N	O=C1C=CC2(C(=C1C)C3OC(=O)C(C)C3CC2)C
182	0.879	273.19333	d-estradiol	[M+H]+	273.19	C18H24O2	Estrogens and derivatives	VOXZDWNPVJITMN-ZBRFXRBCSA-N	OC1=CC=C2C(=C1)CCC3C2CCC4(C)C(O)CCC34
183	0.883	231.09755	(+)-Dihydrokavain	[M-H]-	231.10266	C14H16O3	Kavalactones	VOOYTQRREPYRIW-LBPRGKRZSA-N	O=C1OC(CC(OC)=C1)CCC=2C=CC=CC2
184	0.883	353.15369	1,4a-dimethyl-9-oxo-7-propan-2-yl-3,4,10,10a-tetrahydro-2H-phenanthrene-1-carboxylic acid	[M+H]+	353.15134	C20H26O3	Diterpenoids	MSWJSDLNPCSSNW-UHFFFAOYSA-N	O=C(O)C1(C)CCCC2(C3=CC=C(C=C3C(=O)CC12)C(C)C)C
185	0.883	179.05479	9-Fluorenone	[M-H]-	179.05023	C13H8O	Fluorenes	YLQWCDOCJODRMT-UHFFFAOYSA-N	O=C1C=2C=CC=CC2C=3C=CC=CC13
186	0.883	195.05017	Gluconate	[M-H]-	195.05103	C6H12O7	Medium-chain hydroxy acids and derivatives	RGHNJXZEOKUKBD-SQOUGZDYSA-N	OC[C=H](O)[C=H](O)[C=H](O)[C=H](O)C(O)=O
187	0.883	329.1813	Guaiacin	[M+H]+	329.17999	C20H24O4	Aryltetralin lignans	TZAAYUCUPIYQBR-JGRMJRGVSA-N	OC1=CC=C(C=C1OC)C2C3=CC(O)=C(OC)C=C3CC(C)C2C
188	0.883	187.10748	NALPHA-ACETYL-L-LYSINE	[M-H]-	187.108	C8H16N2O3	N-acyl-alpha amino acids	VEYYWZRYIYDQJM-ZETCQYMHSA-N	O=C(NC(C(=O)O)CCCCN)C
189	0.884	459.13342	(-)-Podophyllotoxin	[M-H]-	459.12967	C22H22O8	Lignols	YJGVMLPVUAXIQN-UHFFFAOYNA-N	COC1=CC(=CC(OC)=C1OC)C1C2C(COC2=O)C(O)C2=C1C=C1OCOC1=C2
190	0.884	428.21259	2-amino-3-[[(6E,9E)-5-hydroxy-8-methoxy-5,9,13,14-tetramethyl-2-oxo-1-oxacyclotetradeca-6,9-dien-4-yl]sulfanyl]propanoic acid	[M-H]-	428.21121	C21H35NO6S	Macrolides and analogues	XBLHNZRXAORYAC-KHQWPVHISA-N	O=C(O)C(N)CSC1CC(=O)OC(C)C(C)CCC=C(C)C(OC)C=CC1(O)C
191	0.884	253.05511	7,2'-Dihydroxyflavone	[M-H]-	253.05063	C15H10O4	Flavones	NUGPQONICGTVNA-UHFFFAOYSA-N	O=C1C=C(OC2=CC(O)=CC=C12)C=3C=CC=CC3O
192	0.885	311.09625	Sappanone A Dimethyl Ether	[M-H]-	311.09595	C18H16O5	Homoisoflavonoids	VCNDHJFYRZQMHX-KPKJPENVSA-N	O=C1C2=CC=C(OC)C=C2OCC1=CC3=CC=C(OC)C(O)=C3
193	0.886	113.02359	FT-OH	[M-H]-	113.02197	C3H5F3O	PFSA	HDBGBTNNPRCVND-UHFFFAOYSA-N	FC(F)(F)CCO
194	0.886	112.0864	Histamine	[M+H]+	112.08692	C5H9N3	2-arylethylamines	NTYJJOPFIAHURM-UHFFFAOYSA-N	N1=CNC(=C1)CCN
195	0.886	124.03897	Isonicotinic acid	[M+H]+	124.03931	C6H5NO2	Pyridinecarboxylic acids	TWBYWOBDOCUKOW-UHFFFAOYSA-N	OC(=O)C1=CC=NC=C1
196	0.886	465.10007	Myricitrin	[M+H]+	465.10001	C21H20O12	Flavonoid-3-O-glycosides	DCYOADKBABEMIQ-OWMUPTOHSA-N	O=C1C(OC2OC(C)C(O)C(O)C2O)=C(OC=3C=C(O)C=C(O)C13)C=4C=C(O)C(O)=C(O)C4
197	0.886	186.10512	Norharman	[M+H]+	186.10257	C11H8N2	Beta carbolines	AIFRHYZBTHREPW-UHFFFAOYSA-N	N=1C=CC2=C(C1)NC=3C=CC=CC32
198	0.887	310.11353	N2,N2-Dimethylguanosine	[M-H]-	310.11569	C12H17N5O5	Purine nucleosides	RSPURTUNRHNVGF-UHFFFAOYNA-N	OC1=NC(=NC2=C1N=CN2C3OC(CO)C(O)C3O)N(C)C
199	0.887	245.11237	palythine	[M+H]+	245.10899	C10H16N2O5	Alpha amino acids and derivatives	KYCBIRYKYQCBFO-UHFFFAOYSA-N	O=C(O)CN=C1C(OC)=C(N)CC(O)(CO)C1
200	0.888	212.05486	(+-)-Baclofen	[M-H]-	212.04839	C10H12ClNO2	Gamma amino acids and derivatives	KPYSYYIEGFHWSV-UHFFFAOYNA-N	NCC(CC(O)=O)C1=CC=C(Cl)C=C1
201	0.888	144.10252	(2R)-6-methylpiperidine-2-carboxylic acid	[M+H]+	144.10001	C7H13NO2	L-alpha-amino acids	IHLDCUQUFBWSJU-PRJDIBJQSA-N	O=C(O)C1NC(C)CCC1
202	0.888	363.14069	Gibberellin A8	[M-H]-	363.14493	C19H24O7	C19-gibberellin 6-carboxylic acids	WZRRJZYYGOOHRC-UQJCXHNCSA-N	C[C=]12[C=H]3[C=H](C(O)=O)[C=]45CC(=C)[C=](O)(C4)CC[C=H]5[C=]3(C[C=H](O)[C=H]1O)OC2=O
203	0.888	134.08087	N-Methylthreonine	[M+H]+	134.08118	C5H11NO3	L-alpha-amino acids	CCAIIPMIAFGKSI-UHFFFAOYNA-N	O=C(O)C(NC)C(O)C
204	0.888	283.06604	Xanthosine (Not validated)	[M-H]-	283.0679	C10H12N4O6	Nucleosides	UBORTCNDUKBEOP-UHFFFAOYNA-N	OC=1N=C(O)C=2N=CN(C=2(N=1))C3OC(CO)C(O)C3(O)
205	0.889	290.12292	Arbutin	[M+H]+	290.12341	C12H16O7	Phenolic glycosides	BJRNKVDFDLYUGJ-RMPHRYRLSA-N	OC1=CC=C(OC2OC(CO)C(O)C(O)C2O)C=C1
206	0.889	315.08136	Eriodictyol 7,3'-dimethyl ether	[M-H]-	315.07901	C17H16O6	7-O-methylated flavonoids	QZJVBGCZOLNWHW-AWEZNQCLSA-N	O=C1C2=C(O)C=C(OC)C=C2OC(C3=CC=C(O)C(OC)=C3)C1
207	0.889	322.11252	Pesticide4_Flutolanil_C17H16F3NO2_Benzamide, N-[3-(1-methylethoxy)phenyl]-2-(trifluoromethyl)-	[M-H]-	322.10599	C17H16F3NO2	Benzanilides	PTCGDEVVHUXTMP-UHFFFAOYSA-N	FC(F)(F)C=1C=CC=CC1C(O)=NC2=CC=CC(OC(C)C)=C2
208	0.889	226.10651	triacanthine	[M+H]+	226.11	C10H13N5	6-aminopurines	BEPGTHDUUROBHM-UHFFFAOYSA-N	N1=CN=C2C1=C(N=CN2CC=C(C)C)N
209	0.891	352.15793	9-(beta-d-ribofuranosyl)zeatin	[M+H]+	352.16	C15H21N5O5	Purine nucleosides	GOSWTRUMMSCNCW-GPTCUSJASA-N	OCC(=CCNC1=NC=NC2=C1N=CN2C3OC(CO)C(O)C3O)C
210	0.891	479.23477	Dehydrolucidenic acid A, 20(21)-	[M+H]+	479.23999	C27H36O6	11-oxosteroids	APMICFQMLNFXRZ-UHFFFAOYSA-N	O=C(O)CCC(=C)C1CC(=O)C2(C3=C(C(=O)CC12C)C4(C)CCC(=O)C(C)(C)C4CC3O)C
211	0.892	409.18039	2-(5-methoxy-4,8,8-trimethyl-2-oxo-2,8,9,10-tetrahydropyrano[2,3-f]chromen-3-yl)-N-(pyridin-2-yl)acetamide	[M+H]+	409.17999	C23H24N2O5	Angular pyranocoumarins	XBYUUOMUOFZSBJ-UHFFFAOYSA-N	O=C1OC2=C(C(OC)=CC=3OC(C)(C)CCC32)C(=C1CC(=O)NC4=NC=CC=C4)C
212	0.892	331.1601	Cinchonine hydrochloride	[M+H]+	331.16	C19H23ClN2O	Cinchona alkaloids	IMUHWLVEEVGMBC-PAAXXVDQSA-N	Cl.OC(C=1C=CN=C2C=CC=CC21)C3N4CCC(C3)C(C=C)C4
213	0.892	410.16357	FucGlcNAcGA	[M+H]+	410.16568	C16H27NO11	N-acyl-alpha-hexosamines	ZMHFWGFDQOCSHY-GEHFNBAWSA-N	C[C=H]1O[C=H](O[C=H]2[C=H](O)[C=H](CO)O[C=H](OCC=O)[C=H]2N=C(C)O)[C=H](O)[C=H](O)[C=H]1O

214	0.892	249.10724	Glu-Thr	[M+H]+	249.10811	C9H16N2O6	Dipeptides	JSIQVRIXMINMTA-UHFFFAOYNA-N	O=C(O)CCC(N)C(O)=NC(C(=O)O)C(O)C
215	0.892	337.15906	Lotusine	[M+H]+	337.16	C19H24NO3+	Benzylisoquinolines	ZKTMLINFIQCERN-QGZVFWFLSA-O	OC1=CC=C(C=C1)CC2C3=CC(OC)=C(O)C=C3CC[N+]2(C)C
216	0.892	235.11823	Mepirizole	[M+H]+	235.11896	C11H14N4O2	Alkyl aryl ethers	RHAXSHUQNIEUEY-UHFFFAOYSA-N	N=1C(=NC(=CC1OC)C)N2N=C(C=C2OC)C
217	0.892	303.2019	Quercetin	[M+H]+	303.20657	C15H10O7	Flavonols	REFJWTPEDVJJIY-UHFFFAOYSA-N	O=C1C(O)=C(OC=2C=C(O)C=C(O)C12)C=3C=CC(O)=C(O)C3
218	0.892	457.11777	Raltitrexed	[M-H]-	457.11874	C21H22N4O6S	Glutamic acid and derivatives	IVTVGDXNLFLDRM-UHFFFAOYNA-N	O=C(O)CCC(NC(=O)C=1SC(=CC1)N(C)CC=2C=CC3=NC(=NC(O)=C3C2)C)C(=O)O
219	0.893	372.12805	(1r,3R,4s,5S)-4-{[(2E)-3-(3,4-Dihydroxyphenyl)-2-propenoyl]oxy}-1,3,5-trihydroxycyclohexanecarboxylic acid	[M+H]+	372.12891	C16H18O9	Quinic acids and derivatives	GYFFKZTYYAFCTR-UCEFVAKBSA-N	O=C(O)C1(O)CC(O)C(OC(=O)C=CC2=CC=C(O)C(O)=C2)C(O)C1
220	0.893	271.12787	3-(4-hydroxy-5-oxo-3-phenyl-2H-furan-2-yl)propanoic acid	[M+H]+	271.12769	C13H12O5	Butenolides	PPZVSYXNLXFYAD-UHFFFAOYSA-N	O=C(O)CCC1OC(=O)C(O)=C1C=2C=CC=CC2
221	0.893	291.15372	Androsterone	[M+H]+	291.15656	C19H30O2	Androgens and derivatives	QGXBDMJGAMFCBF-HLUDHZFRSA-N	O=C1CCC2C3CCC4CC(O)CCC4(C)C3CCC12C
222	0.893	116.07078	L-VALINE	[M-H]-	116.071	C5H11NO2	Valine and derivatives	KZSNJWFQEVHDMF-UHFFFAOYSA-N	O=C(O)C(N)C(C)C
223	0.893	251.1671	Lychnopholic acid	[M+H]+	251.16901	C15H22O3	Sesquiterpenoids	LMJMJHGFJXZGDE-WKMLJHOTSA-N	O=C(O)C1=CCCC(=C)C2CC(C)(C)C2C(O)C1
224	0.893	341.0383	Pesticide2_Thiophanate-methyl_C12H14N4O4S2_	[M-H]-	341.039	C12H14N4O4S2	Benzene and substituted derivatives	QGHREAKMXXNCOA-UHFFFAOYSA-N	O=C(OC)NC(=S)NC=1C=CC=CC1NC(=S)NC(=O)OC
225	0.893	168.06474	Pyridoxal	[M+H]+	168.065	C8H9NO3	Pyridoxals and derivatives	RADKZDMFGJYCBB-UHFFFAOYSA-N	O=CC1=C(O)C(=NC=C1CO)C
226	0.894	231.13382	Kainic Acid	[M+H]+	231.13393	C10H15NO4	Kainoids	VLSMHEGGTFMBBZ-OOZYFLPDSA-N	O=C(O)CC1C(NCC1C(=C)C)C(=O)O
227	0.894	380.16791	Kasugamycin	[M+H]+	380.16635	C14H25N3O9	Aminocyclitol glycosides	PVTHJAPFENJVNC-UQTMRZPGSA-N	O=C(O)C(=NC1CC(N)C(OC1C)OC2C(O)C(O)C(O)C(O)C2O)N
228	0.894	257.19839	Sparteine	[M+H]+	257.20001	C15H26N2	Sparteine, lupanine, and related alkaloids	SLRCCWJSBJZJBV-ZQDZILKHSA-N	N12CCCCC1C3CN4CCCCC4C(C2)C3
229	0.894	335.14423	Thymol-beta-D-glucoside	[M+H]+	335.14648	C16H24O6	Terpene glycosides	GKQGIQVSMCHAFX-IBEHDNSVSA-N	OCC1OC(OC2=CC(=CC=C2C(C)C)C)C(O)C(O)C1O
230	0.895	490.19086	[(2R,3S,4S,5S)-4-hydroxy-2,5-bis(hydroxymethyl)-5-[(2R,3R,4S,5S,6R)-3,4,5-trihydroxy-6-(hydroxymethyl)oxan-2-yl]oxyoxolan-3-yl] (E)-3-phenylprop-2-	[M+H]+	490.19189	C21H28O12	Cinnamic acid esters	AKARZXADDNXORH-WQVLWSBKSA-N	O=C(OC1C(O)C(OC2OC(CO)C(O)C(O)C2O)(OC1CO)CO)C=CC=3C=CC=CC3
231	0.895	278.12198	3-methoxy-6-methyl-6,7-dihydro-5h-dibenzo[c,e]azepin-2-ol	[M+H]+	278.12	C16H17NO2	Benzazepines	GJHXLOJRUGWNKP-UHFFFAOYSA-N	OC=1C=C2C=3C=CC=CC3CN(C)CC2=CC1OC
232	0.895	286.17496	Dendrobine	[M+H]+	286.17001	C16H23NO2	Indoles and derivatives	YCWDWJWRRMHVMZ-JSYFJIKASA-N	O=C1OC23C4N(C)CC5CCC(C13C2C(C)C)C45C
233	0.895	362.08304	Fenoxaprop-P-ethyl	[M+H]+	362.07898	C18H16ClNO5	2-phenoxypropionic acid esters	PQKBPHSEKWERTG-UHFFFAOYNA-N	O=C(OCC)C(OC1=CC=C(OC2=NC=3C=CC(Cl)=CC3O2)C=C1)C
234	0.896	202.10716	(E)-1-(4-methylquinazolin-2(1H)-ylidene)guanidine	[M+H]+	202.11	C10H11N5	Quinazolinamines	ONQKSKDLYYDFLL-UHFFFAOYSA-N	N=C(N=C1N=C(C=2C=CC=CC2N1)C)N
235	0.896	392.16351	(S)-methyl 2-(3-(6,7-dimethoxy-4-oxoquinazolin-3(4H)-yl)propanamido)-3-methylbutanoate	[M+H]+	392.16	C19H25N3O6	Hybrid peptides	VLJIRKJQXBCCFT-KRWDZBQOSA-N	O=C(OC)C(NC(=O)CCN1C=NC=2C=C(OC)C(OC)=CC2C1=O)C(C)C
236	0.896	312.19	17-hydroxy-3-oxo-, (17??)-19-Norpregna-5(10),9(11)-diene-21-nitrile	[M+H]+	312.19	C20H25NO2	Oxosteroids	WUQMVSOZWZOUJR-FUMNGEBKSA-N	N#CCC1(O)CCC2C3C(=CCC21C)C4=C(CC(=O)CC4)CC3
237	0.896	579.17517	5,7-dihydroxy-2-(4-hydroxyphenyl)-3-[(2S,3R,4S,5S,6R)-3,4,5-trihydroxy-6-[[(2S,3R,4S,5S)-3,4,5-trihydroxyoxan-2-yl]oxymethyl]oxan-2-yl]oxychromen-	[M-H]-	579.17218	C26H28O15	Flavonoid-3-O-glycosides	YJPZWZRYHLEDNA-KSPKLRDJSA-N	O=C1C(OC2OC(COC3OCC(O)C(O)C3O)C(O)C(O)C2O)=C(OC=4C=C(O)C=C(O)C14)C=5C=CC(O)=CC5
238	0.897	554.20581	1-((S)-2-((S)-2-(2-hydroxy-4-oxoquinazolin-3(4H)-yl)-3-(1H-indol-3-yl)propanamido)propanoyl)piperidine-4-carboxylic acid	[M+H]+	554.20001	C28H29N5O6	N-acyl-alpha amino acids and derivatives	NXJYJHDDQUZYBZ-HJPURHCSSA-N	O=C(O)C1CCN(C(=O)C(NC(=O)C(N2C(=O)C=3C=CC=CC3N=C2O)CC4=CNC=5C=CC=CC54)C)CC1
239	0.897	185.12793	2-hydroxy-1-isopentyl-4-methyl-1H-imidazol-5(4H)-one	[M+H]+	185.13	C9H16N2O2	Hydantoins	CEHHIMFMAIPKGU-UHFFFAOYSA-N	O=C1N(C(O)=NC1C)CCC(C)C
240	0.897	365.15567	GlcNAcThrNAc	[M+H]+	365.15546	C14H24N2O9	N-acyl-alpha-hexosamines	CUSVKSYUKVCGOL-UYHBOPCKSA-N	CC(O)=N[C=H](CO)[C=H](O[C=H]1O[C=H](CO)[C=H](O)[C=H](O)[C=H]1N=C(C)O)C=O
241	0.897	385.25507	Ibutilide fumarate	[M+H]+	385.25201	C44H76N4O10S2	Phenylbutylamines	PCIOHQNIRPWFMV-WXXKFALUSA-N	O=C(O)C=CC(=O)O.O=S(=O)(NC1=CC=C(C=C1)C(O)CCCN(CC)CCCCCCC)C.O=S(=O)(NC1=CC=C(C=C1)C(O)CCCN(CC)CCCCCCC)C
242	0.897	263.10114	N6-benzyl-7H-purine-2,6-diamine	[M+H]+	263.10001	C12H12N6	6-alkylaminopurines	XSSREOLCRTWEPI-UHFFFAOYSA-N	N1=CNC2=C1N=C(N=C2NCC=3C=CC=CC3)N
243	0.897	279.1254	Pesticide4_Oxadixyl_C14H18N2O4_	[M+H]+	279.134	C14H18N2O4	Anilides	UWVQIROCRJWDKL-UHFFFAOYSA-N	O=C1OCCN1N(C(=O)COC)C=2C(=CC=CC2C)C
244	0.897	288.10703	Rutaecarpine	[M+H]+	288.10999	C18H13N3O	Beta carbolines	ACVGWSKVRYFWRP-UHFFFAOYSA-N	O=C1C=2C=CC=CC2N=C3C=4NC=5C=CC=CC5C4CCN13
245	0.898	472.22598	[(2R,3S,4S,5R,6R)-3,4,5-trihydroxy-6-[2-(4-hydroxyphenyl)ethoxy]oxan-2-yl]methyl 2-(2-oxo-1,3-dihydroindol-3-yl)acetate	[M+H]+	472.22751	C24H27NO9	Saccharolipids	YOTVUKXKJYRTAR-NAJYVHOLSA-N	O=C(OCC1OC(OCCC2=CC=C(O)C=C2)C(O)C(O)C1O)CC3C(O)=NC=4C=CC=CC43
246	0.898	257.07693	1-Methylpseudouridine	[M-H]-	257.07791	C10H14N2O6	Nucleoside and nucleotide analogues	UVBYMVOUBXYSFV-UHFFFAOYNA-N	O=C1N=C(O)C(=CN1C)C2OC(CO)C(O)C2O
247	0.898	204.12234	Acetylcarnitine	[M]+	204.12248	C9H18NO4	Acyl carnitines	RDHQFKQIGNGIED-UHFFFAOYNA-O	O=C(OC(CC(=O)O)C[N+](C)(C)C)C
248	0.898	347.14291	albocycline	[M+H]+	347.1416	C18H28O4	Macrolides and analogues	BYWWNDLILWPPJP-REXWONOSSA-N	O=C1OC(C)C(C)CCC=C(C)C(OC)C=CC(O)(C=C1)C
249	0.898	354.17535	Dihydrozeatin riboside	[M+H]+	354.17719	C15H23N5O5	Purine nucleosides	DBVVQDGIJAUEAZ-YXYADJKSSA-N	CC(CO)CCNC1=C2N=CN([C=H]3O[C=H](CO)[C=H](O)[C=H]3O)C2=NC=N1
250	0.898	319.14841	Erianin	[M+H]+	319.14999	C18H22O5	Stilbenes	UXDFUVFNIAJEGM-UHFFFAOYSA-N	OC1=CC(=CC=C1OC)CCC2=CC(OC)=C(OC)C(OC)=C2
251	0.899	241.1541	(4R,4aS,6R)-6-isopropenyl-4,4a-dimethyl-4,4a,5,6,7,8-hexahydro-2(3H)-naphthalenone	[M+H]+	241.16	C15H22O	Eremophilane, 8,9-secoeremophilane and	WTOYNNBCKUYIKC-JMSVASOKSA-N	O=C1C=C2CCC(C(=C)C)CC2(C)C(C)C1
252	0.899	371.18024	2-(furan-3-yl)-7,8-dihydroxy-6a,7,10b-trimethyl-octahydro-1H-naphtho[2,1-c]pyran-4-one	[M+H]+	371.17999	C20H28O5	Diterpene lactones	RBEATXBLUJTDQZ-UHFFFAOYSA-N	O=C1OC(C2=COC=C2)CC3(C)C1CCC4(C)C3CCC(O)C4(O)C
253	0.899	185.05571	2,3-Dihydroxybiphenyl	[M-H]-	185.06081	C12H10O2	Biphenyls and derivatives	YKOQAAJBYBTSBS-UHFFFAOYSA-N	C1=CC=C(C=C1)C=2C=CC=C(C2O)O
254	0.899	623.20239	3-[(2S,3R,4S,5S,6R)-4,5-dihydroxy-6-(hydroxymethyl)-3-[(2S,3R,4S,5R,6R)-3,4,5-trihydroxy-6-(hydroxymethyl)oxan-2-yl]oxyoxan-2-yl]oxy-5-hydroxy-2-	[M-H]-	623.20544	C28H32O16	Flavonoid-3-O-glycosides	PNBMEXOTFKNHLQ-PVZBESAUSA-N	O=C1C(OC2OC(CO)C(O)C(O)C2OC3OC(CO)C(O)C(O)C3O)=C(OC4=CC(OC)=CC(O)=C14)C=5C=CC(O)=CC5
255	0.899	299.15945	Auraptene	[M+H]+	299.16	C19H22O3	Terpene lactones	RSDDHGSKLOSQFK-RVDMUPIBSA-N	O=C1OC=2C=C(OCC=C(C)CCC=C(C)C)C=CC2C=C1
256	0.899	251.1013	Dehydroeffusol	[M+H]+	251.10001	C17H14O2	Phenanthrols	GSSPKCPIRDPBQE-UHFFFAOYSA-N	OC=1C=C(C=C)C=2C(=CC=C3C2C=CC(O)=C3C)C1

257	0.899	304.14941	evodiamine	[M+H]+	304.14999	C19H17N3O	Beta carbolines	TXDUTHBFYKGSAH-UHFFFAOYSA-N	O=C1C=2C=CC=CC2N(C)C3C=4NC=5C=CC=CC5C4CCN13
258	0.899	347.17996	Isorosmanol	[M+H]+	347.17999	C20H26O5	Diterpene lactones	UXVPWKDITRJELA-CLWJZODNSA-N	O=C1OC2C3=CC(=C(O)C(O)=C3C14CCCC(C)(C)C4C2O)C(C)C
259	0.899	397.15878	Myrislignan	[M+H]+	397.16	C21H26O6	Lignans, neolignans and related compounds	ULZFTGWWPHYLGI-UHFFFAOYSA-N	OC1=CC=C(C=C1OC)C(O)C(OC2=C(OC)C=C(C=C2OC)CC=C)C
260	0.899	189.12294	N-epsilon-Acetyllysine	[M+H]+	189.12337	C8H16N2O3	D-alpha-amino acids	DTERQYGMUDWYAZ-UHFFFAOYNA-N	O=C(O)C(N)CCCCN=C(O)C
261	0.899	237.12297	Pesticide4_Cymoxanil_C7H10N4O3_(2E)-2-Cyano-N-(ethylcarbamoyl)-2-(methoxyimino)acetamide	[M+H]+	237.123	C7H10N4O3	Oxime ethers	XERJKGMBORTKEO-VZUCSPMQSA-N	N#CC(=NOC)C(O)=NC(O)=NCC
262	0.899	209.12801	Pilocarpine	[M+H]+	209.12801	C11H16N2O2	Alkaloids and derivatives	QCHFTSOMWOSFHM-UHFFFAOYSA-N	O=C1OCC(CC2=CN=CN2C)C1CC
263	0.9	355.19733	14-deoxy-11,12-dihydroandrographolide	[M+H]+	355.20001	C20H28O4	Diterpene lactones	GQPOXOPEDHRPQT-CRBRZBHVSA-N	O=C1OCC(C=CC2C(=C)CCC3C(C)(CO)C(O)CCC23C)=C1
264	0.9	327.15308	Acepromazine	[M+H]+	327.15256	C19H22N2OS	Phenothiazines	NOSIYYJFMPDDSA-UHFFFAOYSA-N	CN(C)CCCN1C2=CC=CC=C2SC2=C1C=C(C=C2)C(C)=O
265	0.9	152.05614	Guanine	[M+H]+	152.05669	C5H5N5O	Purines and purine derivatives	UYTPUPDQBNUYGX-UHFFFAOYSA-N	c1nc2c([nH]1)[nH]c(=N)nc2O
266	0.9	292.13849	N-Fructosyl isoleucine	[M-H]-	292.1391	C12H23NO7	N-Fructosyl amino acids	VYGRYVGDPYFVCA-UHFFFAOYNA-N	O=C(O)C(NCC1(O)(OC(CO)C(O)C1(O)))C(C)CC
267	0.901	317.17029	[6]-Gingerol	[M+H]+	317.17001	C17H26O4	Gingerols	NLDDIKRKFXEWBK-AWEZNQCLSA-N	O=C(CCC1=CC=C(O)C(OC)=C1)CC(O)CCCCC
268	0.901	251.07591	2'-Deoxyinosine	[M-H]-	251.07858	C10H12N4O4	Purine 2'-deoxyribonucleosides	VGONTNSXDCQUGY-RRKCRQDMSA-N	OC[C=H]1O[C=H](C[C=H]1O)N1C=NC2=C1N=CN=C2O
269	0.901	256.09259	2'-O-Methylcytidine	[M-H]-	256.0939	C10H15N3O5	Pyrimidine nucleosides	RFCQJGFZUQFYRF-UHFFFAOYNA-N	N=C1N=C(O)N(C=C1)C2OC(CO)C(O)C2OC
270	0.901	237.08633	3,4-dihydro-2,2-dimethyl-indeno[1,2-b]-pyran-5(2H)-one	[M+H]+	237.08501	C14H14O2	Indenes and isoindenes	ICNOFNFXLJFTLZ-UHFFFAOYSA-N	O=C1C=2C=CC=CC2C=3OC(C)(C)CCC13
271	0.901	442.19034	butyrolactone i	[M+H]+	442.186	C24H24O7	Fatty acid esters	NGOLMNWQNHWEKU-XMMPIXPASA-N	O=C1OC(C(=O)OC)(C(=C1O)C=2C=CC(O)=CC2)CC3=CC=C(O)C(=C3)CC=C(C)C
272	0.901	260.15964	Isoleucylglutamine	[M+H]+	260.16	C11H21N3O4	Dipeptides	CNPNWGHRMBQHBZ-UHFFFAOYNA-N	O=C(O)C(NC(=O)C(N)C(C)CC)CCC(=O)N
273	0.901	188.05731	N-Acetylglutamic acid	[M-H]-	188.05644	C7H11NO5	Glutamic acid and derivatives	RFMMMVDNIPUKGG-UHFFFAOYNA-N	O=C(O)CCC(N=C(O)C)C(=O)O
274	0.901	194.11696	Pesticide3_Isoprocarb_C11H15NO2_2-Isopropylphenyl methylcarbamate	[M+H]+	194.117	C11H15NO2	Cumenes	QBSJMKIUCUGGNG-UHFFFAOYSA-N	OC(=NC)OC=1C=CC=CC1C(C)C
275	0.901	303.03775	Thymidine-3',5'-cyclic monophosphate sodium salt	[M-H]-	303.03876	C10H13N2O7P	Pyrimidones	QSJFDOVQWZVUQG-UHFFFAOYNA-N	CC1=CN(C2CC3OP(O)(=O)OCC3O2)C(=O)NC1=O
276	0.902	382.16867	Azelastine	[M+H]+	382.16806	C22H24ClN3O	Phthalazinones	MBUVEWMHONZEQD-UHFFFAOYNA-N	CN1CCCC(CC1)N2C(C3=CC=CC=C3C(CC4=CC=C(C=C4)Cl)=N2)=O
277	0.902	627.14978	Baimaside	[M+H]+	627.15002	C27H30O17	Flavonoid-3-O-glycosides	RDUAJIJVNHKTQC-UJECXLDQSA-N	O=C1C(OC2OC(CO)C(O)C(O)C2OC3OC(CO)C(O)C(O)C3O)=C(OC=4C=C(O)C=C(O)C14)C=5C=CC(O)=C(O)C5
278	0.902	255.09207	Chrysin	[M+H]+	255.09	C15H10O4	Flavones	RTIXKCRFFJGDFG-UHFFFAOYSA-N	O=C1C=C(OC=2C=C(O)C=C(O)C12)C=3C=CC=CC3
279	0.902	300.10641	Erysovine	[M+H]+	300.10001	C18H21NO3	Erythrinanes	IHPMURIXWRKEKD-UHFFFAOYSA-N	OC=1C=C2C(=CC1OC)CCN3CC=C4C=CC(OC)CC423
280	0.902	164.07068	Phenylalanine (Not validated)	[M-H]-	164.0708	C9H11NO2	Amino acids	COLNVLDHVKWLRT-UHFFFAOYNA-N	O=C(O)C(N)CC1=CC=CC=C1
281	0.903	454.19165	3,7,21-trihydroxy-17,17-dimethyl-14-(3-methylbut-2-en-1-yl)-10,12,16-trioxapentacyclo[11.8.0.0?,??.0?,?.0??,??]henicosa-	[M+H]+	454.186	C25H24O7	8-prenylated isoflavanones	DQNIDSLDXGTEPL-UHFFFAOYSA-N	O=C1C2=C(O)C=3C=CC(OC3C(=C2OC4OC5=CC(O)=CC=C5C14O)CC=C(C)C)(C)C
282	0.903	271.09088	Alloimperatorin	[M+H]+	271.09	C16H14O4	8-hydroxypsoralens	KDXVVZMYSLWJMA-UHFFFAOYSA-N	O=C1OC=2C(O)=C3OC=CC3=C(C2C=C1)CC=C(C)C
283	0.903	415.16873	Diltiazem HCl (Tiazac)	[M+H]+	415.16901	C22H27ClN2O4S	Benzothiazepines	HDRXZJPWHTXQRI-BHDTVMLSSA-N	Cl.O=C(OC1C(=O)N(C=2C=CC=CC2SC1C3=CC=C(OC)C=C3)CCN(C)C)C
284	0.903	131.03433	ETHYLMALONIC ACID	[M-H]-	131.034	C5H8O4	Branched fatty acids	UKFXDFUAPNAMPJ-UHFFFAOYSA-N	O=C(O)C(C(=O)O)CC
285	0.903	400.21652	Mepacrine	[M+H]+	400.21503	C23H30ClN3O	Acridines	GPKJTRJOBQGKQK-UHFFFAOYNA-N	CCN(CC)CCCC(C)N=C1C2=C(NC3=C1C=C(OC)C=C3)C=C(Cl)C=C2
286	0.903	243.06123	Uridine (Not validated)	[M-H]-	243.0616	C9H12N2O6	Nucleosides	DRTQHJPVMGBUCF-UHFFFAOYNA-N	O=C1C=CN(C(=O)N1)C2OC(CO)C(O)C2(O)
287	0.904	400.18069	colchicine	[M+H]+	400.17999	C22H25NO6	Tropones	IAKHMKGGTNLKSZ-INIZCTEOSA-N	O=C1C=C2C(=CC=C1OC)C3=C(OC)C(OC)=C(OC)C=C3CCC2NC(=O)C
288	0.904	316.18454	Nonivamide	[M+H]+	316.17999	C17H27NO3	Methoxyphenols	RGOVYLWUIBMPGK-UHFFFAOYSA-N	O=C(NCC1=CC=C(O)C(OC)=C1)CCCCCCCC
289	0.906	343.15097	[2-[4-methyl-2-(2-methylpropanoyloxy)phenyl]oxiran-2-yl]methyl 2-methylpropanoate	[M+H]+	343.15158	C18H24O5	Phenol esters	OLARKEMZPWGFJU-UHFFFAOYSA-N	O=C(OC1=CC(=CC=C1C2(OC2)COC(=O)C(C)C)C)C(C)C
290	0.906	237.06332	4,8-dihydroxy-7-(hydroxymethyl)-6-methoxy-3,4-dihydro-2H-naphthalen-1-one	[M-H]-	237.064	C12H14O5	Tetralins	NXSUIALRPVXVTA-UHFFFAOYSA-N	O=C1C2=C(O)C(=C(OC)C=C2C(O)CC1)CO
291	0.906	274.09143	cordycepin	[M+H]+	274.09106	C10H13N5O3	Purine 3'-deoxyribonucleosides	OFEZSBMBBKLLBJ-BAJZRUMYSA-N	OCC1OC(N2C=NC=3C(=NC=NC32)N)C(O)C1
292	0.906	227.11038	N-Methylcytisine	[M+H]+	227.11	C12H16N2O	Cytisine and derivatives	CULUKMPMGVXCEI-VHSXEESVSA-N	O=C1C=CC=C2N1CC3CN(C)CC2C3
293	0.907	136.07518	2-Phenylacetamide	[M+H]+	136.07568	C8H9NO	Phenylacetamides	LSBDFXRDZJMBSC-UHFFFAOYSA-N	N=C(O)CC=1C=CC=CC1
294	0.907	284.09805	Guanosine	[M+H]+	284.09894	C10H13N5O5	Purine nucleosides	NYHBQMYGNKIUIF-UUOKFMHZSA-N	C([C=H]1[C=H]([C=H]([C=H](n2cnc3c2[nH]c(=N)nc3O)O1)O)O)O
295	0.907	220.0806	O-SUCCINYL-L-HOMOSERINE	[M+H]+	220.08	C8H13NO6	L-alpha-amino acids	GNISQJGXJIDKDJ-YFKPBYRVSA-N	O=C(O)CCC(=O)OCCC(N)C(=O)O
296	0.907	389.20026	quassin	[M+H]+	389.20001	C22H28O6	Quassinoids	IOSXSVZRTUWBHC-DJFCUZIKSA-N	O=C1OC2CC3C(C=C(OC)C(=O)C3(C)C4C(=O)C(OC)=C(C)C(C1)C24C)C
297	0.908	302.08572	(E)-4-(4-methoxybenzylidene)-3-phenylisoxazol-5(4H)-one	[M+H]+	302.07999	C17H13NO3	Anisoles	HGIIHVJCDPYWBJ-RVDMUPIBSA-N	O=C1ON=C(C=2C=CC=CC2)C1=CC3=CC=C(OC)C=C3
298	0.908	164.07002	Ethionine	[M+H]+	164.07397	C6H13NO2S	L-alpha-amino acids	GGLZPLKKBSSKCX-YFKPBYRVSA-N	CCSCC[C=H](N)C(O)=O
299	0.908	224.112	N-2-Fluorenylacetamide	[M+H]+	224.10699	C15H13NO	Fluorenes	CZIHNRWJTSTCEX-UHFFFAOYSA-N	OC(=NC1=CC=C2C=3C=CC=CC3CC2=C1)C

300	0.908	161.10692	Tryptamine	[M+H]+	161.10699	C10H12N2	Tryptamines and derivatives	APJYDQYYACXCRM-UHFFFAOYSA-N	NCCC1=CNC=2C=CC=CC21
301	0.909	525.1203	(2S,3R,4aS,12bR)-2,3,4a,8-tetrahydroxy-12b-(5-hydroxy-6-methyloxan-2-yl)oxy-3-methyl-2,4-dihydrobenzo[a]anthracene-1,7,12-trione	[M+H]+	525.11572	C25H26O10	Anthraquinone glycosides	CKZNKYSWWCSICZ-GTJIABFSSA-N	O=C1C=2C=CC3(O)CC(O)(C)C(O)C(=O)C3(OC4OC(C)C(O)CC4)C2C(=O)C=5C=CC=C(O)C15
302	0.909	421.17996	15-Acetoxyeremantholide B	[M+H]+	421.17999	C22H28O8	Furofurans	UNFGCPCUYMXWDG-OOQPEQBFSA-N	O=C(OCC1=CC2OC(=O)C3(C)C2C(OC3(O)C(C)CC)CC4(OC1=CC4=O)C)C
303	0.909	409.10858	5,7-dihydroxy-2-(4-hydroxy-3-methoxyphenyl)-6-(3-methylbut-2-enyl)-2,3-dihydrochromen-4-one	[M+H]+	409.1048	C21H22O6	6-prenylated flavanones	GWVGVAPNTKLROZ-UHFFFAOYSA-N	O=C1C2=C(O)C(=C(O)C=C2OC(C3=CC=C(O)C(OC)=C3)C1)CC=C(C)C
304	0.909	133.01364	D-(+)-Malic acid	[M-H]-	133.01425	C4H6O5	Beta hydroxy acids and derivatives	BJEPYKJPYRNKOW-UWTATZPHSA-N	O[C=H](CC(O)=O)C(O)=O
305	0.909	305.06287	Flavanol base + 5O	[M-H]-	305.06491	C15H14O7	Flavanol O-glycosides	XMOCLSLCDHWDHP-UHFFFAOYNA-N	OC3=CC(O)=C2C(OC(C1=CC(O)=C(O)C(O)=C1)C(O)C2)=C3
306	0.91	291.08328	Epicatechin	[M+H]+	291.08401	C15H14O6	Catechins	PFTAWBLQPZVEMU-UKRRQHHQSA-N	OC=1C=C(O)C2=C(OC(C3=CC=C(O)C(O)=C3)C(O)C2)C1
307	0.91	327.11667	Eucalyptin	[M+H]+	327.116	C19H18O5	7-O-methylated flavonoids	NHMMAMIRMITGRD-UHFFFAOYSA-N	O=C1C=C(OC2=C1C(O)=C(C(OC)=C2C)C)C=3C=CC(OC)=CC3
308	0.91	137.04549	Hypoxanthine	[M+H]+	137.04579	C5H4N4O	Hypoxanthines	FDGQSTZJBFJUBT-UHFFFAOYSA-N	c1nc2c([nH]1)ncnc2O
309	0.91	328.13754	N-Fructosyl phenylalanine	[M+H]+	328.13879	C15H21NO7	N-Fructosyl amino acids	FAVRCIXPIVJIPN-UHFFFAOYNA-N	O=C(O)C(NCC1(O)(OC(CO)C(O)C1(O)))CC2=CC=CC=C2
310	0.911	173.05659	1-naphthalenecarboxaldehyde, 2-hydroxy	[M+H]+	173.05971	C11H8O2	Naphthols and derivatives	NTCCNERMXRIPTR-UHFFFAOYSA-N	C1=CC=C2C(=C1)C=CC(=C2C=O)O
311	0.911	115.00303	Maleic acid	[M-H]-	115.00368	C4H4O4	Dicarboxylic acids and derivatives	VZCYOOQTPOCHFL-UPHRSURJSA-N	OC(=O)\C=C/C(O)=O
312	0.911	245.185	N-L-Leucyl-L-leucine	[M+H]+	245.18597	C12H24N2O3	Peptides	LCPYQJIKPJDLLB-UHFFFAOYSA-N	O=C(O)C(NC(=O)C(N)CC(C)C)CC(C)C
313	0.912	452.22025	(3,5,14-trihydroxy-13-methyl-17-(5-oxo-2,5-dihydrofuran-3-yl)hexadecahydro-1H-cyclopenta[a]phenanthren-10-yl)methyl nitrate	[M+H]+	452.22	C23H33NO8	Cardenolides and derivatives	HCEPYGIKCDFDPJ-UHFFFAOYSA-N	O=C1OCC(=C1)C2CCC3(O)C4CCC5(O)CC(O)CCC5(CO[N+](=O)[O-])C4CCC23C
314	0.912	132.10138	Isoleucine	[M+H]+	132.1019	C6H13NO2	Isoleucine and derivatives	AGPKZVBTJJNPAG-UHFFFAOYNA-N	CCC(C)C(N)C(O)=O
315	0.912	585.30957	lappaconitine	[M+H]+	585.31	C32H44N2O8	Lappaconitine-type diterpenoid alkaloids	NWBWCXBPKTTZNQ-GSAAXPBRSA-N	O=C(OC12CN(CC)C3C4CC1C3(C(OC)CC2)C5CC6C(OC)CC4(O)C5(O)C6OC)C=7C=CC=CC7NC(=O)C
316	0.912	163.02548	Lumazine	[M-H]-	163.02615	C6H4N4O2	Pteridines and derivatives	UYEUUXMDVNYCAM-UHFFFAOYSA-N	OC=1N=C(O)C2=NC=CN=C2N1
317	0.912	154.04941	Mesalazine	[M+H]+	154.04987	C7H7NO3	Aminobenzoic acids	KBOPZPXVLCULAV-UHFFFAOYSA-N	C1=CC(=C(C=C1N)C(=O)O)O
318	0.912	303.08398	N-Acetylaspartylglutamic acid	[M-H]-	303.08337	C11H16N2O8	Dipeptides	OPVPGKGADVGKTG-UHFFFAOYNA-N	O=C(O)CCC(N=C(O)C(N=C(O)C)CC(=O)O)C(=O)O
319	0.913	220.11786	Pantothenic acid	[M+H]+	220.11795	C9H17NO5	Secondary alcohols	GHOKWGTUZJEAQD-UHFFFAOYSA-N	CC(C)(CO)C(C(=NCCC(=O)O)O)O
320	0.913	153.07565	Ribitol	[M+H]+	153.07574	C5H12O5	Sugar alcohols	HEBKCHPVOIAQTA-ZXFHETKHNA-N	OC[C=H](O)[C=H](O)[C=H](O)CO
321	0.914	370.20917	allocryptopine	[M+H]+	370.21036	C21H23NO5	Protopine alkaloids	HYBRYAPKQCZIAE-UHFFFAOYSA-N	O=C1C2=CC=3OCOC3C=C2CCN(C)CC4=C(OC)C(OC)=CC=C4C1
322	0.915	159.02898	2-OXOADIPATE	[M-H]-	159.02901	C6H8O5	Medium-chain keto acids and derivatives	FGSBNBBHOZHUBO-UHFFFAOYSA-N	O=C(O)C(=O)CCCC(=O)O
323	0.916	495.10931	Flavonol base + 4O, 1MeO, O-Hex	[M+H]+	495.11292	C22H22O13	Flavonol O-glycosides	AFCDXKGLUDDXCK-UHFFFAOYNA-N	O=C2C(O)=C(OC=3C=C(OC1OC(CO)C(O)C(O)C1(O))C(OC)=C(O)C2=3)C4=CC=C(O)C(O)=C4
324	0.916	301.10318	Haematoxylin	[M-H]-	301.10031	C16H14O6	1-benzopyrans	WZUVPPKBWHMQCE-UHFFFAOYSA-N	OC1=CC=C2C(OCC3(O)CC4=CC(O)=C(O)C=C4C23)=C1O
325	0.916	335.05991	Pachyrrhizin	[M-H]-	335.05612	C19H12O6	Isoflav-3-enones	PENSQRMNZZWMGV-UHFFFAOYSA-N	O=C1OC2=CC=3OC=CC3C=C2C=C1C=4C=C5OCOC5=CC4OC
326	0.918	204.08644	7-Methylsulfenylheptyl isothiocyanate	[M+H]+	204.08752	C9H17NS2	Glucosinolate breakdown metabolites	LDIRGNDMTOGVRB-UHFFFAOYSA-N	CSCCCCCCCN=C=S
327	0.918	103.03886	ACETOACETATE	[M+H]+	103.04	C4H6O3	Short-chain keto acids and derivatives	WDJHALXBUFZDSR-UHFFFAOYSA-N	O=C(O)CC(=O)C
328	0.918	117.05432	Ketoisovaleric acid	[M+H]+	117.05462	C5H8O3	Short-chain keto acids and derivatives	QHKABHOOEWYVLI-UHFFFAOYSA-N	O=C(O)C(=O)C(C)C
329	0.918	273.03693	Phenylbenzimidazole sulfonic acid	[M-H]-	273.03394	C13H10N2O3S	Phenylbenzimidazoles	UVCJGUGAGLDPAA-UHFFFAOYSA-N	C1=CC=C(C=C1)C2=NC=3C=CC(=CC3N2)S(O)(=O)=O
330	0.918	523.10327	Protopseudohypericin	[M+H]+	523.09998	C30H18O9	Perylenequinones	QFAPJWSQKUFHAP-UHFFFAOYSA-N	O=C1C2=C(O)C=C(C=C2C3=C4C=5C=C(C=C(O)C5C(=O)C6=C(O)C=C(O)C(C=7C(O)=CC(O)=C1C73)=C64)CO)C
331	0.921	425.10352	[3-hydroxy-3-methyl-1-(7-oxofuro[3,2-g]chromen-9-yl)oxybutan-2-yl] (Z)-2-methylbut-2-enoate	[M+H]+	425.10196	C21H22O7	Psoralens	VJVXQSFKTVUEFY-XGICHPGQSA-N	O=C1OC2=C(OCC(OC(=O)C(=CC)C)C(O)(C)C)C=3OC=CC3C=C2C=C1
332	0.922	406.10947	8-(azepan-1-ium-1-ylmethyl)-3-(4-chlorophenyl)-4-oxo-4H-chromen-7-olate	[M+H]+	406.10999	C22H22ClNO3	Isoflavones	LUXRPLCOGZWQEC-UHFFFAOYSA-N	O=C1C(=COC=2C1=CC=C([O-])C2C[NH+]3CCCCCC3)C=4C=CC(Cl)=CC4
333	0.922	285.10693	8-hydroxy-5,8a-dimethyl-3-methylidene-3a,4,4a,8,9,9a-hexahydrobenzo[f][1]benzofuran-2,7-dione	[M+H]+	285.10971	C15H18O4	Eudesmanolides, secoeudesmanolides, and	XAFJIIZCMNHLRS-UHFFFAOYSA-N	O=C1OC2CC3(C)C(O)C(=O)C=C(C)C3CC2C1=C
334	0.923	453.09949	[(2R,3S,4S,5R,6S)-6-(3,4-dihydroxybenzoyl)oxy-3,4,5-trihydroxyoxan-2-yl]methyl 3,4-dihydroxybenzoate	[M+H]+	453.10275	C20H20O12	p-Hydroxybenzoic acid alkyl esters	VNQNUULCMPXFER-XDCWJTEKSA-N	O=C(OCC1OC(OC(=O)C2=CC=C(O)C(O)=C2)C(O)C(O)C1O)C3=CC=C(O)C(O)=C3
335	0.924	369.18414	Gibberellin A4&A7	[M+H]+	369.18405	C19H22O5	C19-gibberellin 6-carboxylic acids	SEEGHKWOBVVBTQ-UKJRIFTCSA-N	O=C(O)C1C2C3(OC(=O)C2(C)C(O)C=C3)C4CCC5C(=C)CC14C5
336	0.927	103.00303	MALONATE	[M-H]-	103.003	C3H4O4	Dicarboxylic acids and derivatives	OFOBLEOULBTSOW-UHFFFAOYSA-N	O=C(O)CC(=O)O
337	0.929	222.01904	5-Aminonaphthalene-2-sulfonic acid	[M-H]-	222.02304	C10H9NO3S	2-naphthalene sulfonates	UWPJYQYRSWYIGZ-UHFFFAOYSA-N	C1=CC2=CC(=CC=C2C(=C1)N)S(O)(=O)=O
338	0.933	209.02934	GALACTARATE	[M-H]-	209.03	C6H10O8	Glucuronic acid derivatives	DSLZVSRJTYRBFB-UHFFFAOYSA-N	O=C(O)C(O)C(O)C(O)C(O)C(=O)O
339	0.934	400.14264	9-[[3-methyl-3-[(4-methyl-5-oxo-2H-furan-2-yl)methyl]oxiran-2-yl]methoxy]furo[3,2-g]chromen-7-one	[M+H]+	400.13907	C21H18O7	Psoralens	CISFAPDYRQPRCZ-UHFFFAOYSA-N	O=C1OC2=C(OCC3OC3(C)CC4OC(=O)C(=C4)C)C=5OC=CC5C=C2C=C1
340	0.935	573.1394	methyl 8,11,15,20,22-pentahydroxy-24-methyl-13,18,27-trioxo-6-oxaheptacyclo[15.10.2.0?,??.0?,??.0?,??.0?,??.0??,??]nonacosa-	[M+H]+	573.13916	C31H24O11	Xanthones	KIWTVJVARRPBPR-UHFFFAOYSA-N	O=C1C=2C(O)=C3C(=CC2OC4(C(=O)OC)C1=C(O)CCC4O)CC56C=CC3C(=O)C6=C(O)C=7C(O)=CC(=CC7C5=O)C
341	0.935	351.08566	Pinastric acid	[M-H]-	351.086	C20H16O6	Anisoles	KXQKSBAGVQMQSN-FBMGVBCBSA-N	O=C1OC(C(O)=C1C=2C=CC(OC)=CC2)=C(C(=O)OC)C=3C=CC=CC3
342	0.936	202.06985	2-((2H-pyrazolo[3,4-d]pyrimidin-4-yl)amino)ethanol	[M+H]+	202.07001	C7H9N5O	Pyrazolo[3,4-d]pyrimidines	LCOTUPXIQXPIPA-UHFFFAOYSA-N	OCCNC1=NC=NC2=NNC=C21

343	0.936	409.07272	4',5,7-trihydroxy-3,6-dimethoxyflavone	[M+H]+	409.07047	C17H14O7	6-O-methylated flavonoids	DDNPCXHBFYJXBJ-UHFFFAOYSA-N	O=C1C(OC)=C(OC2=CC(O)=C(OC)C(O)=C12)C=3C=CC(O)=CC3
344	0.939	149.06329	p-Coumaraldehyde	[M+H]+	149.05971	C9H8O2	Cinnamaldehydes	CJXMVKYNVIGQBS-OWOJBTEDSA-N	OC1=CC=C(\C=C\C=O)C=C1
345	0.94	545.13092	(3S,5R,8S)-2-[(benzoyloxy)methyl]-8-methyl-3-{[(2S,3R,4S,5S,6R)-3,4,5-trihydroxy-6-(hydroxymethyl)oxan-2-yl]oxy}-9,10-	[M+H]+	545.13232	C23H28O13S	Terpene glycosides	IRBLIRNSGBXQFB-HJOPEMAASA-N	O=C(OCC12C3OC4(C)CC(O3)(C2CC41OC5OC(CO)C(O)C(O)C5O)S(=O)(=O)O)C=6C=CC=CC6
346	0.94	351.06812	bergenin	[M+H]+	351.07001	C14H16O9	Gallic acid and derivatives	YWJXCIXBAKGUKZ-HJJNZUOJSA-N	O=C1OC2C(O)C(O)C(OC2C3=C(O)C(OC)=C(O)C=C13)CO
347	0.941	574.15588	3-(3,4-dimethoxyphenyl)-5-hydroxy-10-(3-oxo-3,5,6,7-tetrahydropyrido[3,2,1-ij]quinolin-2-yl)-9,10-dihydropyrano[2,3-	[M+H]+	574.15002	C32H25NO8	Pyranoisoflavonoids	PDSXCCOQUZAXNS-UHFFFAOYSA-N	O=C1OC2=CC(O)=C3C(=O)C(=COC3=C2C(C4=CC=5C=CC=C6C5N(C4=O)CCC6)C1)C=7C=CC(OC)=C(OC)C7
348	0.941	515.13336	7-hydroxy-2-(4-hydroxy-3,5-dimethoxyphenyl)-5-[(2S,3R,4S,5S,6R)-3,4,5-trihydroxy-6-(hydroxymethyl)oxan-2-yl]oxychromen-4-one	[M+H]+	515.13647	C23H24O12	Flavonoid O-glycosides	FLSOTPIEFVBPBU-LDBVRRDLSA-N	O=C1C=C(OC2=CC(O)=CC(OC3OC(CO)C(O)C(O)C3O)=C12)C=4C=C(OC)C(O)=C(OC)C4
349	0.942	412.09091	3-(2,3-dihydrobenzo[b][1,4]dioxin-6-yl)-8-((dimethylamino)methyl)-7-hydroxy-4H-chromen-4-one hydrochloride	[M+H]+	412.09	C20H20ClNO5	Isoflavones	ZWPFIFVTTMMGQP-UHFFFAOYSA-N	Cl.O=C1C(=COC=2C1=CC=C(O)C2CN(C)C)C=3C=CC=4OCCOC4C3
350	0.942	349.09314	methyl 2-(4a,6,10a-trihydroxy-1-methyl-5,10-dioxo-3,4-dihydro-1H-benzo[g]isochromen-3-yl)acetate	[M-H]-	349.0929	C17H18O8	Naphthopyranones	HILBWEPXZDMOAE-UHFFFAOYSA-N	O=C(OC)CC1OC(C)C2(O)C(=O)C=3C=CC=C(O)C3C(=O)C2(O)C1
351	0.943	307.07803	(4E,6E)-2-(hydroxymethyl)-3-(3-methoxy-3-oxopropyl)deca-4,6-dienoic acid	[M+H]+	307.07788	C15H24O5	Medium-chain fatty acids	PVHUKALHRBPQNM-BSWSSELBSA-N	O=C(O)C(CO)C(C=CC=CCCC)CCC(=O)OC
352	0.943	331.06177	3-(1,2-dihydroxypropyl)-1,6,8-trihydroxyanthracene-9,10-dione	[M+H]+	331.06299	C17H14O7	Hydroxyanthraquinones	BMKQPAJRQUUYGC-UHFFFAOYSA-N	O=C1C=2C=C(O)C=C(O)C2C(=O)C3=C(O)C=C(C=C13)C(O)C(O)C
353	0.943	347.073	asterric acid	[M-H]-	347.07321	C17H16O8	Diphenylethers	XOKVHFNTYHPEHN-UHFFFAOYSA-N	O=C(O)C1=C(O)C=C(C=C1OC=2C(OC)=CC(O)=CC2C(=O)OC)C
354	0.943	149.0098	Tartaric acid	[M-H]-	149.00916	C4H6O6	Sugar acids and derivatives	FEWJPZIEWOKRBE-UHFFFAOYNA-N	O=C(O)C(O)C(O)C(=O)O
355	0.944	205.09776	Tryptophan	[M+H]+	205.0975	C11H12N2O2	Indole and derivatives	QIVBCDIJIAJPQS-UHFFFAOYNA-N	O=C(O)C(N)CC2=CNC=1C=CC=CC=12
356	0.945	543.12946	(2R)-8-[(2R,3S)-5,7-dihydroxy-2-(4-hydroxyphenyl)-4-oxo-2,3-dihydrochromen-3-yl]-5,7-dihydroxy-2-(4-hydroxyphenyl)-2,3-dihydrochromen-	[M+H]+	543.12854	C30H22O10	Biflavonoids and polyflavonoids	MXEIKUWMKSYEII-XRHWURSXSA-N	O=C1C2=C(O)C=C(O)C(=C2OC(C3=CC=C(O)C=C3)C1)C4C(=O)C=5C(O)=CC(O)=CC5OC4C6=CC=C(O)C=C6
357	0.946	189.15919	Pesticide9_Propamocarb_C9H20N2O2_Propyl [3-(dimethylamino)propyl]carbamate	[M+H]+	189.159	C9H20N2O2	Carbamate esters	WZZLDXDUQPOXNW-UHFFFAOYSA-N	OC(=NCCCN(C)C)OCCC
358	0.947	234.09686	1-isothiocyanato-8-methanesulfinyloctane	[M+H]+	234.099	C10H19NOS2	Glucosinolate breakdown metabolites	BCRXKWOQVFKZAG-UHFFFAOYNA-N	O=S(C)CCCCCCCCN=C=S
359	0.947	88.03909	Vinyl Carbamate	[M+H]+	88.03931	C3H5NO2	Carbamate esters	LVLANIHJQRZTPY-UHFFFAOYSA-N	N=C(O)OC=C
360	0.949	385.12595	[(2S,3R,4S,5S,6R)-3,4,5-trihydroxy-6-(hydroxymethyl)oxan-2-yl] (2E,6E)-8-hydroxy-2,6-dimethylocta-2,6-dienoate	[M+H]+	385.12592	C16H26O8	Terpene glycosides	QZIOPOIUZGGXKI-KOYXEPTDSA-N	O=C(OC1OC(CO)C(O)C(O)C1O)C(=CCCC(=CCO)C)C
361	0.95	220.08052	1-Isothiocyanato-7-(methylsulfinyl)-heptane	[M+H]+	220.08243	C9H17NOS2	Sulfoxides	OGYHCBGORZWBPH-UHFFFAOYNA-N	CS(=O)CCCCCCCN=C=S
362	0.95	377.08466	4-hydroxy-5-[(2S,3R,4S,5S,6R)-3,4,5-trihydroxy-6-(hydroxymethyl)oxan-2-yl]oxy-1H-benzo[f][2]benzofuran-3-one	[M-H]-	377.0878	C18H18O9	Phenolic glycosides	SGQJDTLFQCDZGS-UQEKQVNISA-N	O=C1OCC2=CC=3C=CC=C(OC4OC(CO)C(O)C(O)C4O)C3C(O)=C12
363	0.95	353.08609	4-methylumbelliferyl glucuronide	[M+H]+	353.0867	C16H16O9	Coumarin glycosides	ARQXEQLMMNGFDU-JHZZJYKESA-N	CC1=CC(=O)OC2=C1C=CC(O[C=H]1O[C=H]([C=H](O)[C=H](O)[C=H]1O)C(O)=O)=C2
364	0.955	381.08005	?-Gentiobiose	[M+H]+	381.07938	C12H22O11	O-glycosyl compounds	DLRVVLDZNNYCBX-CQUJWQHSSA-N	OCC1OC(OCC2OC(O)C(O)C(O)C2O)C(O)C(O)C1O
365	0.955	100.07543	N-Methyl-2-pyrrolidone	[M+H]+	100.07569	C5H9NO	N-alkylpyrrolidines	SECXISVLQFMRJM-UHFFFAOYSA-N	CN1CCCC1=O
366	0.956	128.07033	Isoguvacine	[M+H]+	128.0706	C6H9NO2	Hydropyridines	KRVDMABBKYMBHG-UHFFFAOYSA-N	OC(=O)C1=CCNCC1
367	0.956	130.04941	L-5-Oxoproline	[M+H]+	130.04987	C5H7NO3	Alpha amino acids and derivatives	ODHCTXKNWHHXJC-VKHMYHEASA-N	OC(=O)[C=H]1CCC(O)=N1
368	0.958	103.00397	Malonic acid	[M-H]-	103.00368	C3H4O4	Dicarboxylic acids and derivatives	OFOBLEOULBTSOW-UHFFFAOYSA-N	O=C(O)CC(=O)O
369	0.958	405.15991	N-methyl-N-(2-(pyridin-2-yl)ethyl)-2-(2,3,5-trimethyl-7-oxo-7H-furo[3,2-g]chromen-6-yl)acetamide	[M+H]+	405.16	C24H24N2O4	Psoralens	CHZCSOQKQNCACE-UHFFFAOYSA-N	O=C1OC=2C=C3OC(=C(C3=CC2C(=C1CC(=O)N(C)CCC4=NC=CC=C4)C)C)C
370	0.959	355.08621	7,9-dimethoxy-6-(4-methoxyphenyl)-[1,3]dioxolo[4,5-g]chromen-8-one	[M-H]-	355.08234	C19H16O7	5-O-methylated flavonoids	LWUQVHKGVXRUPD-UHFFFAOYSA-N	O=C1C(OC)=C(OC=2C=C3OCOC3=C(OC)C21)C=4C=CC(OC)=CC4
371	0.959	432.16943	Beta-Peltatin	[M+H]+	432.16528	C22H22O8	Lignan lactones	HLBPOYVRLSXWJJ-PDSMFRHLSA-N	O=C1OCC2CC3=C(O)C=4OCOC4C=C3C(C5=CC(OC)=C(OC)C(OC)=C5)C12
372	0.959	383.07373	irigenin	[M+Na]+	383.07001	C18H16O8	3'-hydroxy,4'-methoxyisoflavonoids	TUGWPJJTQNLKCL-UHFFFAOYSA-N	O=C1C(=COC2=CC(O)=C(OC)C(O)=C12)C=3C=C(O)C(OC)=C(OC)C3
373	0.961	235.04451	1-[3-hydroxy-2-(2-hydroxypropan-2-yl)-2,3-dihydro-1-benzofuran-5-yl]ethanone	[M-H]-	235.03888	C13H16O4	Acetophenones	XFIALOBGPJCAMB-UHFFFAOYSA-N	O=C(C1=CC=C2OC(C(O)C2=C1)C(O)(C)C)C
374	0.961	102.05458	1-Aminocyclopropane-1-carboxylic acid	[M+H]+	102.05495	C4H7NO2	Alpha amino acids	PAJPWUMXBYXFCZ-UHFFFAOYSA-N	NC1(CC1)C(O)=O
375	0.961	116.03393	Maleamic acid	[M+H]+	116.03422	C4H5NO3	Straight chain fatty acids	FSQQTNAZHBEJLS-UHFFFAOYSA-N	O=C(O)C=CC(=N)O
376	0.961	199.063	Monuron	[M+H]+	199.06326	C9H11ClN2O	N-phenylureas	BMLIZLVNXIYGCK-UHFFFAOYSA-N	CN(C)C(=O)NC1=CC=C(Cl)C=C1
377	0.962	217.04756	2-Benzyl-4-chlorophenol	[M-H]-	217.04257	C13H11ClO	Diphenylmethanes	NCKMMSIFQUPKCK-UHFFFAOYSA-N	C1=CC=C(C=C1)CC2=CC(=CC=C2O)Cl
378	0.962	418.15298	5,6-dihydroxy-9,12,13-trimethyl-14-(2-methylpropyl)-2H,5H,6H,7H,8H,13H,13aH,14H,15H,16H,16bH-oxacyclododeca[3,2-e]isoindole-	[M+H]+	418.1524	C24H35NO5	Isoindoles	MPMJLHQXZNLSCP-UHFFFAOYSA-N	O=C1OC23C(O)=NC(CC(C)C)C3C(C(=CC2C=C(C)CCC(=O)C(O)CC1)C)C
379	0.964	209.02921	D-SACCHARIC ACID	[M-H]-	209.03	C6H10O8	Glucuronic acid derivatives	DSLZVSRJTYRBFB-LLEIAEIESA-N	O=C(O)C(O)C(O)C(O)C(O)C(=O)O
380	0.966	353.0741	6:3+6O fatty acyl hexoside	[M-H]-	353.07169	C12H18O12	Fatty acyl hexosides	DVMKRMXSGLOXJZ-UHFFFAOYNA-N	O=C2OC(C(O)=C2(OC1OC(CO)C(O)C(O)C1(O)))C(O)C(O)O
381	0.967	343.14117	Abietin	[M+H]+	343.14001	C16H22O8	Phenolic glycosides	SFLMUHDGSQZDOW-FAOXUISGSA-N	OCC=CC1=CC=C(OC2OC(CO)C(O)C(O)C2O)C(OC)=C1
382	0.967	511.10333	Fissinolide	[M-H]-	511.09772	C29H36O8	Limonoids	YOTCKRFNSMJTGD-PSWUJVJRSA-N	O=C(OC1C2C(=O)C(C)(C3C(=C4CC(=O)OC(C5=COC=C5)C4(C)CC3)C2)C(CC(=O)OC)C1(C)C)C
383	0.967	117.07365	Glycine-Betaine	[M+H]+	117.07843	C5H11NO2	Alpha amino acids	KWIUHFFTVRNATP-UHFFFAOYSA-N	C[N+](C)(C)CC([O-])=O
384	0.968	599.11938	[(2S,3R,4R,5R,6S)-2-[2-(3,4-dihydroxyphenyl)-5,7-dihydroxy-4-oxochromen-3-yl]oxy-4,5-dihydroxy-6-methyloxan-3-yl] 3,4,5-trihydroxybenzoate	[M-H]-	599.11926	C28H24O15	Flavonoid-3-O-glycosides	KTTNFIOZYNBKEY-YIOJSCHVSA-N	O=C(OC1C(O)C(O)C(OC1OC=2C(=O)C=3C(O)=CC(O)=CC3OC2C=4C=CC(O)=C(O)C4)C)C5=CC(O)=C(O)C(O)=C5
385	0.968	321.12735	Pangamic Acid Sodium	[M+H]+	321.12683	C10H18NNaO8	Alpha amino acid esters	MKVDFFROCUECAJ-CKDSBIORSA-M	[Na+].O=C([O-])C(O)C(O)C(O)C(O)COC(=O)CN(C)C

386	0.968	258.1091	sn-Glycero-3-phosphocholine	[M]+	258.10956	C8H21NO6P	Glycerophosphocholines	SUHOQUVVVLNYQR-MRVPVSSYSA-O	C[N+](C)(C)CCOP(O)(=O)OC[C=H](O)CO
387	0.969	388.14377	methyl 3-(4-hydroxyphenyl)-2-[(4-hydroxyphenyl)methyl]-4-methoxy-5-oxofuran-2-carboxylate	[M+H]+	388.13907	C20H18O7	Fatty acid esters	DYMYDSWEIIXYNX-UHFFFAOYSA-N	O=C1OC(C(=O)OC)(C(C=2C=CC(O)=CC2)=C1OC)CC3=CC=C(O)C=C3
388	0.97	80.04919	Pyridine	[M+H]+	80.04948	C5H5N	Pyridines and derivatives	JUJWROOIHBZHMG-UHFFFAOYSA-N	C1=CC=NC=C1
389	0.971	212.05444	Imidacloprid-urea	[M+H]+	212.05852	C9H10ClN3O	2-halopyridines	ADWTYURAFSWNSU-UHFFFAOYSA-N	OC1=NCCN1CC1=CN=C(Cl)C=C1
390	0.974	367.06216	Dracorhodin perchlorate	[M+H]+	367.06	C17H15ClO7	5-O-methylated flavonoids	KRTYZFUODYMZPG-UHFFFAOYSA-N	[O-][Cl+3]([O-])([O-])[O-].OC=1C=C2[O+]=C(C=CC2=C(OC)C1C)C=3C=CC=CC3
391	0.975	649.21802	(S)-2-(((S)-7-acetamido-1,2,3-trimethoxy-9-oxo-5,6,7,9-tetrahydrobenzo[a]heptalen-10-yl)amino)-N-(benzo[d]thiazol-2-yl)-4-	[M+H]+	649.21997	C33H36N4O6S2	Methionine and derivatives	RUFIGEPXTYTFFK-DHLKQENFSA-N	O=C1C=C2C(=CC=C1NC(C(=O)NC3=NC=4C=CC=CC4S3)CCSC)C5=C(OC)C(OC)=C(OC)C=C5CCC2NC(=O)C
392	0.976	282.08313	Guanosine (Not validated)	[M-H]-	282.0834	C10H13N5O5	Nucleosides	NYHBQMYGNKIUIF-UHFFFAOYNA-N	O=C3NC(=NC1=C3(N=CN1C2OC(CO)C(O)C2(O)))N
393	0.977	300.0481	2-Chloroadenosine	[M-H]-	300.05051	C10H12ClN5O4	Purine nucleosides	BIXYYZIIJIXVFW-UHFFFAOYNA-N	ClC=1N=C(N)C=2N=CN(C2N1)C3OC(CO)C(O)C3O
394	0.977	214.03406	3-Chlorotyrosine	[M-H]-	214.02765	C9H10ClNO3	Tyrosine and derivatives	ACWBBAGYTKWBCD-UHFFFAOYNA-N	O=C(O)C(N)CC1=CC=C(O)C(Cl)=C1
395	0.977	218.10289	Pesticide4_Pymetrozine_C10H11N5O_(E)-4,5-Dihydro-6-methyl-4-[(3-pyridinylmethylene)amino]-1,2,4-triazin-3(2H)-one	[M+H]+	218.103	C10H11N5O	1,2,4-triazines	QHMTXANCGGJZRX-WUXMJOGZSA-N	OC1=NN=C(C)CN1N=CC=2C=NC=CC2
396	0.977	292.07083	PFSM-ammonio	[M-H]-	292.07101	[C8H16F3N2O4S]+	PFSA	ODUUUNLAMNNKMD-UHFFFAOYSA-O	O=C(O)C[N+](C)(C)CCCNS(=O)(=O)C(F)(F)F
397	0.98	332.15433	Deoxysappanone B 7,3’-Dimethyl Ether	[M+H]+	332.14923	C18H18O5	Homoisoflavanones	OFAYBXSZQCUARK-UHFFFAOYSA-N	O=C1C2=CC=C(OC)C=C2OCC1CC3=CC=C(O)C(OC)=C3
398	0.981	343.12183	2-alpha-Mannobiose	[M+H]+	343.12347	C12H22O11	O-glycosyl compounds	HIWPGCMGAMJNRG-UHFFFAOYNA-N	OCC1OC(O)C(OC2OC(CO)C(O)C(O)C2O)C(O)C1O
399	0.986	395.05588	bisdethiobis(methylthio)gliotoxin	[M+H]+	395.0564	C15H20N2O4S2	Alpha amino acids and derivatives	OVBAGMZLGLXSBN-UOVKNHIHSA-N	O=C1N(C)C(SC)(C(=O)N2C3C(=CC=CC3O)CC12SC)CO
400	0.986	325.11331	Disaccharide(Hex-Hex)	[M+H]+	325.11371	C12H22O11	Sugars	PVXPPJIGRGXGCY-UHFFFAOYNA-N	OCC2OC(OCC1OC(O)(CO)C(O)C1(O))C(O)C(O)C2(O)
401	0.986	505.17514	Raffinose	[M+H]+	505.1763	C18H32O16	Oligosaccharides	MUPFEKGTMRGPLJ-UHFFFAOYNA-N	OCC1OC(OCC2OC(OC3(OC(CO)C(O)C3O)CO)C(O)C(O)C2O)C(O)C(O)C1O
402	0.988	277.17801	NP1EC (TENTATIVE)	[M-H]-	277.18091	C17H26O3	Phenoxyacetic acid derivatives	NISAHDHKGPWBEM-UHFFFAOYSA-N	CCCCCCCCCC1=CC=C(C=C1)OCC(=O)O
403	0.989	249.03647	Diphenyl phosphate	[M-H]-	249.03223	C12H11O4P	Aryl phosphodiesters	ASMQGLCHMVWBQR-UHFFFAOYSA-N	C1=CC=C(C=C1)OP(O)(=O)OC2=CC=CC=C2
404	0.99	227.07306	4-methoxy-9H-xanthen-9-one	[M+H]+	227.071	C14H10O3	Xanthones	SYEHHMRXODULBY-UHFFFAOYSA-N	O=C1C=2C=CC=CC2OC=3C(OC)=CC=CC13
405	0.99	319.04898	Flavanone base + 6O	[M-H]-	319.0459	C15H12O8	Flavanone O-glycosides	KJXSIXMJHKAJOD-UHFFFAOYNA-N	O=C2C3=C(O)C=C(O)C=C3(OC(C1=CC(O)=C(O)C(O)=C1)C2(O))
406	0.991	148.02434	3-Hydroxyaspartic acid	[M-H]-	148.02515	C4H7NO5	Aspartic acid and derivatives	YYLQUHNPNCGKJQ-UHFFFAOYNA-N	O=C(O)C(O)C(N)C(=O)O
407	0.991	263.01627	4:2 Fluorotelomer alcohol	[M-H]-	263.01239	C6H5F9O	PFSA	JCMNMOBHVPONLD-UHFFFAOYSA-N	FC(F)(F)C(F)(F)C(F)(F)C(F)(F)CCO
408	0.991	305.13574	5,9-dihydroxy-7-(hydroxymethyl)-5,7-dimethyl-4,5a,6,8,8a,9-hexahydro-1H-azuleno[5,6-c]furan-3-one	[M+H]+	305.13593	C15H22O5	Terpene lactones	DBKIEMOKQWYZOA-UHFFFAOYSA-N	O=C1OCC2=C1CC(O)(C)C3CC(C)(CO)CC3C2O
409	0.992	177.09456	Canavanine	[M+H]+	177.09821	C5H12N4O3	L-alpha-amino acids	FSBIGDSBMBYOPN-VKHMYHEASA-N	O=C(O)C(N)CCONC(=N)N
410	0.994	306.1171	2-methyl-3-[(2S,3R,4S,5S,6R)-3,4,5-trihydroxy-6-(hydroxymethyl)oxan-2-yl]oxypyran-4-one	[M+H]+	306.11835	C12H16O8	O-glycosyl compounds	YGSIRXHFAUFUEJ-GPTQDWHKSA-N	O=C1C=COC(=C1OC2OC(CO)C(O)C(O)C2O)C
411	0.994	275.01581	6-PHOSPHOGLUCONIC ACID	[M-H]-	275.017	C6H13O10P	Monosaccharide phosphates	BIRSGZKFKXLSJQ-UHFFFAOYSA-N	O=C(O)C(O)C(O)C(O)C(O)COP(=O)(O)O
412	0.994	199.96814	Cysteine S-sulfate	[M-H]-	199.96928	C3H7NO5S2	S-sulfo-L-cysteines	NOKPBJYHPHHWAN-REOHCLBHSA-N	N[C=H](CSS(O)(=O)=O)C(O)=O
413	0.995	323.0274	URIDINE-5-MONOPHOSPHATE	[M-H]-	323.02802	C9H13N2O9P	Pyrimidine ribonucleoside	DJJCXFVJDGTHFX-UHFFFAOYSA-N	O=C1C=CN(C(=O)N1)C2OC(COP(=O)(O)O)C(O)C2O
414	0.996	333.05783	(2S,3S)-3,5,7-trihydroxy-6-methyl-2-(3,4,5-trihydroxyphenyl)-2,3-dihydrochromen-4-one	[M-H]-	333.06158	C16H14O8	Epigallocatechins	NZRLODDQFPZTPM-CVEARBPZSA-N	O=C1C2=C(O)C(=C(O)C=C2OC(C3=CC(O)=C(O)C(O)=C3)C1O)C
415	0.996	553.11426	Biflavonoid-flavone base + 3O and flavanone base + 2O + 1MeO	[M-H]-	553.11218	C31H22O10	Biflavonoids	XMFILYNCNQOLOA-CYLJNIGPNA-N	O=C1C=C(OC2=C1C(O)=CC(O)=C2C3=CC(=CC=C3(O))C4OC=5C=C(OC)C=C(O)C=5(C(=O)C4))C6=CC=C(O)C=C6
416	0.997	167.99599	Cysteic Acid	[M-H]-	167.99722	C3H7NO5S	Alpha amino acids	XVOYSCVBGLVSOL-UHFFFAOYNA-N	O=C(O)C(N)CS(=O)(=O)O
417	0.997	413.10254	Deacetylasperulosidic acid	[M+H]+	413.10001	C16H22O11	Iridoid O-glycosides	ZVXWFPTVHBWJOU-YYFGDFGFSA-N	O=C(O)C1=COC(OC2OC(CO)C(O)C(O)C2O)C3C(=CC(O)C13)CO
418	0.997	317.0563	Myricetin	[M-H]-	317.05789	C15H10O8	Flavonols	IKMDFBPHZNJCSN-UHFFFAOYSA-N	O=C1C(O)=C(OC=2C=C(O)C=C(O)C12)C=3C=C(O)C(O)=C(O)C3
419	0.998	171.00551	Glycerophosphate(2)	[M-H]-	171.00639	C3H9O6P	Glycerophosphates	AWUCVROLDVIAJX-GSVOUGTGSA-N	OC[C=H](O)COP(O)(O)=O
420	0.999	233.0287	(2R)-5-methoxy-2-methyl-2,3,8,9-tetrahydrofuro[2,3-h]chromen-4-one	[M-H]-	233.02577	C13H14O4	Chromones	BCRRDBKZXSRMHL-SSDOTTSWSA-N	O=C1C2=C(OC)C=C3OCCC3=C2OC(C)C1
421	0.999	192.08609	Shikimic Acid	[M+H]+	192.08664	C7H10O5	Shikimic acids and derivatves	JXOHGGNKMLTUBP-HSUXUTPPSA-N	O=C(O)C1=CC(O)C(O)C(O)C1
422	1.002	259.02078	D-Mannose-6-phosphate	[M-H]-	259.02243	C6H13O9P	Hexose phosphates	NBSCHQHZLSJFNQ-QTVWNMPRSA-N	OC1O[C=H](COP(O)(O)=O)[C=H](O)[C=H](O)[C=H]1O
423	1.002	585.14142	Neomangiferin	[M+H]+	585.14001	C25H28O16	Xanthones	VUWOVGXVRYBSGI-IRXABLMPSA-N	O=C1C2=CC(OC3OC(CO)C(O)C(O)C3O)=C(O)C=C2OC4=CC(O)=C(C(O)=C14)C5OC(CO)C(O)C(O)C5O
424	1.003	274.0907	Pentose + Proline	[M+H]+	274.0932	C11H15NO7	Amino acids	VMOJEEZNLOLYBG-UHFFFAOYNA-N	O=C(O)C1N(C(=O)CC1)CC2(O)(OC(CO)C(O)C2(O))
425	1.003	273.02631	Simonyellin	[M-H]-	273.03	C14H10O6	Isocoumarins and derivatives	GJSCVPZGPZIBMU-UHFFFAOYSA-N	O=C1OC=C2C(OC)=C(O)C(=O)C3=C2C1=C(O)C=C3C
426	1.005	183.08548	D-Sorbitol	[M+H]+	183.08632	C6H14O6	Sugar alcohols	FBPFZTCFMRRESA-FBXFSONDNA-N	OC[C=H](O)[C=H](O)[C=H](O)[C=H](O)CO
427	1.006	393.07874	Hexsose + C5H10O3, 1phosphate	[M-H]-	393.07819	C11H23O13P	Organic acids	HJZBAIMJDQCPCL-UHFFFAOYNA-N	C(C1C(C(C(C(O1)OC(COP(=O)(O)O)C(C(CO)O)O)O)O)O)O
428	1.006	279.04669	Piscidic Acid	[M+H]+	279.04749	C11H12O7	Phenylpropanoic acids	TUODPMGCCJSJRH-UHFFFAOYSA-N	O=C(O)C(O)C(O)(C(=O)O)CC1=CC=C(O)C=C1

429	1.007	241.01073	33FTA	[M-H]-	241.0105	C6H5F7O2	Medium-chain fatty acids	ISFKSWMQWIRDNC-UHFFFAOYSA-N	OC(=O)CCC(F)(F)C(F)(F)C(F)(F)F
430	1.007	261.03696	D-Sorbitol 6-phosphate	[M-H]-	261.03809	C6H15O9P	Monosaccharide phosphates	GACTWZZMVMUKNG-SLPGGIOYSA-N	OC[C=H](O)[C=H](O)[C=H](O)[C=H](O)COP(O)(O)=O
431	1.007	373.17044	Phillygenin	[M+H]+	373.17001	C21H24O6	Furanoid lignans	CPJKKWDCUOOTEW-YJPXFSGGSA-N	OC1=CC=C(C=C1OC)C2OCC3C(OCC23)C4=CC=C(OC)C(OC)=C4
432	1.008	337.12189	Pesticide6_Fenbuconazole_C19H17ClN4_4-(4-Chlorophenyl)-2-phenyl-2-(1H-1,2,4-triazol-1-ylmethyl)butanenitrile	[M+H]+	337.121	C19H17ClN4	Linear 1,3-diarylpropanoids	RQDJADAKIFFEKQ-UHFFFAOYSA-N	N#CC(C=1C=CC=CC1)(CN2N=CN=C2)CCC3=CC=C(Cl)C=C3
433	1.009	603.15314	[(2R,3S,4S,5R,6S)-6-[2,3-dihydroxy-4-[(E)-3-(4-hydroxyphenyl)prop-2-enoyl]phenoxy]-3,4,5-trihydroxyoxan-2-yl]methyl (E)-3-(4-	[M+H]+	603.14728	C30H28O12	Flavonoid O-glycosides	MXLIDYXKRCYRPL-SVQBVAIGSA-N	O=C(OCC1OC(OC2=CC=C(C(=O)C=CC3=CC=C(O)C=C3)C(O)=C2O)C(O)C(O)C1O)C=CC4=CC=C(O)C=C4
434	1.009	123.05582	2-Acetylpyrazine	[M+H]+	123.0553	C6H6N2O	Aryl alkyl ketones	DBZAKQWXICEWNW-UHFFFAOYSA-N	CC(=O)c1cnccn1
435	1.009	229.01082	D-RIBOSE 5-PHOSPHATE	[M-H]-	229.011	C5H11O8P	Pentose phosphates	PPQRONHOSHZGFQ-UHFFFAOYSA-N	O=CC(O)C(O)C(O)COP(=O)(O)O
436	1.009	237.01511	Methazolamide	[M+H]+	237.013	C5H8N4O3S2	Thiadiazole sulfonamides	FLOSMHQXBMRNHR-UHFFFAOYSA-N	O=C(N=C1SC(=NN1C)S(=O)(=O)N)C
437	1.011	207.01566	2-Naphthalene sulfonic acid	[M-H]-	207.01215	C10H8O3S	2-naphthalene sulfonates	KVBGVZZKJNLNJU-UHFFFAOYSA-N	C1=CC=C2C=C(C=CC2=C1)S(O)(=O)=O
438	1.011	241.03622	4,6,8-trihydroxy-7-methoxy-3-methyl-3,4-dihydroisochromen-1-one	[M+H]+	241.03632	C11H12O6	Hydroxybenzoic acid derivatives	ZLLQQKITWRWKTD-UHFFFAOYSA-N	O=C1OC(C)C(O)C2=CC(O)=C(OC)C(O)=C12
439	1.011	218.06564	O-Succinylhomoserine	[M-H]-	218.06702	C8H13NO6	L-alpha-amino acids	GNISQJGXJIDKDJ-UHFFFAOYNA-N	O=C(O)CCC(=O)OCCC(N)C(=O)O
440	1.011	275.04526	Ursinoic Acid	[M-H]-	275.04794	C15H16O5	2,2-dimethyl-1-benzopyrans	XUXBOSSCMLZGTA-UHFFFAOYSA-N	O=C(O)CC(=O)C1=CC=C(OC)C=2C=CC(OC21)(C)C
441	1.014	765.20166	5-hydroxy-3-[(2S,3R,4R,5S)-3-hydroxy-5-(hydroxymethyl)-4-[(2S,3R,4S,5S,6R)-3,4,5-trihydroxy-6-(hydroxymethyl)oxan-2-yl]oxyoxolan-2-	[M+H]+	765.20203	C32H38O19	Flavonoid-7-O-glycosides	LKVIDBLGZHMPCV-HLAATANNSA-N	O=C1C(OC2OC(CO)C(OC3OC(CO)C(O)C(O)C3O)C2O)=C(OC4=CC(OC5OC(C)C(O)C(O)C5O)=CC(O)=C14)C=6C=CC(O)=CC6
442	1.014	329.00366	Furosemide	[M-H]-	329.00046	C12H11ClN2O5S	Aminobenzenesulfonamides	ZZUFCTLCJUWOSV-UHFFFAOYSA-N	C=1C=C(CNC2=CC(=C(C=C2C(=O)O)S(N)(=O)=O)Cl)OC1
443	1.016	173.02065	sn-Glycerol 3-phosphate	[M+H]+	173.02095	C3H9O6P	Glycerophosphates	AWUCVROLDVIAJX-GSVOUGTGSA-N	OC[C=H](O)COP(O)(O)=O
444	1.018	236.11203	N-(9-oxodecyl)acetamide	[M+H]+	236.11226	C12H23NO2	Acetamides	JRJBWKJICGFLDP-UHFFFAOYSA-N	O=C(C)CCCCCCCCN=C(O)C
445	1.018	257.00549	PFCA-H	[M-H]-	257.0054	C6H5F7O3	PFSA	NYXWTWRATMQVGT-UHFFFAOYSA-N	O=C(O)COCC(F)(F)C(F)(F)C(F)(F)F
446	1.019	94.98025	Methanesulfonate	[M-H]-	94.98083	CH4O3S	Organosulfonic acids	AFVFQIVMOAPDHO-UHFFFAOYSA-N	CS(O)(=O)=O
447	1.02	407.16507	(2R,3R,4S,6R)-2-((5-hydroxy-2,2-dimethyl-3,4-dihydro-2H-benzo[h]chromen-6-yl)oxy)-6-(hydroxymethyl)tetrahydro-2H-pyran-3,4,5-triol	[M+H]+	407.16599	C21H26O8	Phenolic glycosides	WVILKFILAZPSRT-MEAUJJKLSA-N	OC1=C(OC2OC(CO)C(O)C(O)C2O)C=3C=CC=CC3C=4OC(C)(C)CCC14
448	1.02	183.00534	2,4-Dinitrophenol	[M-H]-	183.00475	C6H4N2O5	Dinitrophenols	UFBJCMHMOXMLKC-UHFFFAOYSA-N	C1=CC(=C(C=C1N(=O)=O)N(=O)=O)O
449	1.02	218.03207	Ornidazole	[M-H]-	218.0338	C7H10ClN3O3	Nitroimidazoles	IPWKIXLWTCNBKN-UHFFFAOYNA-N	O=N(=O)C1=CN=C(N1CC(O)CCl)C
450	1.021	329.00134	iodinated analogue of makaluvone	[M+H]+	329	[C11H10IN2O2]+	Pyrrolo[4,3,2-de]quinolines	NMSZMSNHNYDJLD-UHFFFAOYSA-O	O=C1C(=O)C2=C3C(=CN2C)CC[NH2+]C3=C1I
451	1.021	140.99435	Phosphonoacetic acid	[M+H]+	140.99474	C2H5O5P	Organic phosphonic acids	XUYJLQHKOGNDPB-UHFFFAOYSA-N	O=C(O)CP(=O)(O)O
452	1.023	239.03094	Sphondin	[M+H]+	239.03046	C12H8O4	Angular furanocoumarins	DLCJNIBLOSKIQW-UHFFFAOYSA-N	O=C1OC=2C(C=C1)=CC(OC)=C3OC=CC32
453	1.027	338.98691	"FRUCTOSE 1,6-BIPHOSPHATE"	[M-H]-	338.98801	C6H14O12P2	Hexose phosphates	XPYBSIWDXQFNMH-UYFOZJQFSA-N	O=C(COP(=O)(O)O)C(O)C(O)C(O)COP(=O)(O)O
454	1.031	277.05878	3-(5,7-dimethoxy-4-oxochromen-2-yl)propanoic acid	[M-H]-	277.0603	C14H14O6	Chromones	AKJIPZYHXYQVJT-UHFFFAOYSA-N	O=C(O)CCC=1OC2=CC(OC)=CC(OC)=C2C(=O)C1
455	1.033	319.04214	Disulfiram	[M+H]+	319.04099	C10H20N2S4	Thiuram disulfides	AUZONCFQVSMFAP-UHFFFAOYSA-N	S=C(SSC(=S)N(CC)CC)N(CC)CC
456	1.033	233.04124	Quebrachitol	[M+H]+	233.04219	C7H14O6	Cyclohexanols	DSCFFEYYQKSRSV-UHFFFAOYSA-N	OC1C(O)C(O)C(OC)C(O)C1O
457	1.035	608.08636	Uridine-5-diphosphoacetylgalactosamine	[M+H]+	608.08887	C17H27N3O17P2	Pyrimidine nucleotide sugars	LFTYTUAZOPRMMI-UHFFFAOYNA-N	O=C1N=C(O)C=CN1C2OC(COP(=O)(O)OP(=O)(O)OC3OC(CO)C(O)C(O)C3N=C(O)C)C(O)C2O
458	1.036	535.03558	UDP-xylose	[M-H]-	535.03717	C14H22N2O16P2	Pyrimidine ribonucleoside	DQQDLYVHOTZLOR-OCIMBMBZSA-N	O[C=H]1[C=H](COP(O)(=O)OP(O)(=O)O[C=H]2OC[C=H](O)[C=H](O)[C=H]2O)O[C=H]([C=H]1O)N1C=CC(O)=NC1=O
459	1.039	166.97424	PHOSPHO(ENOL)PYRUVIC ACID	[M-H]-	166.97501	C3H5O6P	Phosphate esters	DTBNBXWJWCWCIK-UHFFFAOYSA-N	O=C(O)C(OP(=O)(O)O)=C
460	1.04	463.04724	4-Hydroxyglucobrassicin	[M-H]-	463.04868	C16H20N2O10S2	Alkylglucosinolates	CSMYCLLHRFFFLG-UHFFFAOYNA-N	O=S(=O)(O)ON=C(SC1OC(CO)C(O)C(O)C1O)CC2=CNC=3C=CC=C(O)C32
461	1.045	585.13806	7-[(2S,3R,4R,5R,6S)-3,4,5-trihydroxy-6-methyloxan-2-yl]oxy-3-[4-[(2S,3R,4R,5R,6S)-3,4,5-trihydroxy-6-methyloxan-2-yl]oxyphenyl]chromen-4-	[M+H]+	585.1369	C27H30O12	Isoflavonoid O-glycosides	RIXHOIPNPVCEHZ-WIYOTWCASA-N	O=C1C(=COC2=CC(OC3OC(C)C(O)C(O)C3O)=CC=C12)C=4C=CC(OC5OC(C)C(O)C(O)C5O)=CC4
462	1.045	203.14989	Asymmetric dimethylarginine	[M+H]+	203.15025	C8H18N4O2	Arginine and derivatives	YDGMGEXADBMOMJ-LURJTMIESA-N	CN(C)C(=N)NCCC[C=H](C(=O)O)N
463	1.045	412.96481	Perfluorooctanoic acid	[M-H]-	412.96643	C8HF15O2	Perfluoroalkyl carboxylic acid and	SNGREZUHAYWORS-UHFFFAOYSA-N	C(C(C(C(C(C(C(C(F)(F)F)(F)F)(F)F)(F)F)(F)F)(F)F)(F)F)(=O)O
464	1.046	176.01884	1-Isothiocyanato-4-(methylsulfinyl)-butane	[M-H]-	176.02094	C6H11NOS2	Glucosinolate breakdown metabolites	SUVMJBTUFCVSAD-UHFFFAOYNA-N	CS(=O)CCCCN=C=S
465	1.047	517.01044	2-(3,4-dihydroxyphenyl)-5,8-dihydroxy-7-methoxy-3-[(2S,3R,4R,5R,6S)-3,4,5-trihydroxy-6-methyloxan-2-yl]oxychromen-4-one	[M+H]+	517.01416	C22H22O12	Flavonoid-3-O-glycosides	PTUBUBVQXWVBPI-QJCKQCIOSA-N	O=C1C(OC2OC(C)C(O)C(O)C2O)=C(OC3=C(O)C(OC)=CC(O)=C13)C=4C=CC(O)=C(O)C4
466	1.047	259.02002	Carbmazepine	[M+H]+	259.0192	C15H12N2O	Dibenzazepines	FFGPTBGBLSHEPO-UHFFFAOYSA-N	N=C(O)N1C=2C=CC=CC2C=CC=3C=CC=CC31
467	1.048	266.9931	Dichlorophen	[M-H]-	266.9985	C13H10Cl2O2	Diphenylmethanes	MDNWOSOZYLHTCG-UHFFFAOYSA-N	C=1C=C(C(CC2=CC(=CC=C2O)Cl)=CC1Cl)O
468	1.05	149.02304	Phthalic anhydride	[M+H]+	149.02332	C8H4O3	Phthalic anhydrides	LGRFSURHDFAFJT-UHFFFAOYSA-N	O=C1OC(=O)C2=CC=CC=C12
469	1.05	189.1597	Propamocarb	[M+H]+	189.15976	C9H20N2O2	Carbamate esters	WZZLDXDUQPOXNW-UHFFFAOYSA-N	CCCOC(O)=NCCCN(C)C
470	1.05	325.04221	Uridine 5'-monophosphate	[M+H]+	325.04315	C9H13N2O9P	Pyrimidine ribonucleoside	DJJCXFVJDGTHFX-UHFFFAOYNA-N	O=C1N=C(O)C=CN1C2OC(COP(=O)(O)O)C(O)C2O
471	1.052	146.11703	Acetylcholine	[M+H]+	146.117	C7H16NO2	Acyl cholines	OIPILFWXSMYKGL-UHFFFAOYSA-N	CC(=O)OCC[N+](C)(C)C

472	1.053	357.02502	(E)-1,4-bis(4-hydroxy-3-methoxyphenyl)-2,3-dimethylbut-2-ene-1,4-dione	[M+H]+	357.02454	C20H20O6	Lignans, neolignans and related compounds	ZTNYRNGTBAGYIZ-VAWYXSNFSA-N	O=C(C1=CC=C(O)C(OC)=C1)C(=C(C(=O)C2=CC=C(O)C(OC)=C2)C)C
473	1.053	225.01572	Xanthotoxol	[M+H]+	225.01601	C11H6O4	8-hydroxypsoralens	JWVYQQGERKEAHW-UHFFFAOYSA-N	O=C1OC2=C(O)C=3OC=CC3C=C2C=C1
474	1.074	398.07434	Dysidin	[M+H]+	398.069	C16H22Cl3NO4	N-substituted carboxylic acid imides	PXUALOWHEHOKSO-SOXOQJGTSA-N	O=C(C=C(OC)CC(C)C(Cl)(Cl)Cl)N1C(=O)C=C(OC)C1C(C)C
475	1.083	405.00891	Uridine 5'-diphosphate	[M+H]+	405.00946	C9H14N2O12P2	Pyrimidine ribonucleoside	XCCTYIAWTASOJW-XVFCMESISA-N	O[C=H]1[C=H](COP(O)(=O)OP(O)(O)=O)O[C=H]([C=H]1O)N1C=CC(O)=NC1=O
476	1.086	307.04114	Techtochrysin	[M+H]+	307.04001	C16H12O4	7-O-methylated flavonoids	IRZVHDLBAYNPCT-UHFFFAOYSA-N	O=C1C=C(OC=2C=C(OC)C=C(O)C12)C=3C=CC=CC3
477	1.086	565.04608	UDP-D-glucose	[M-H]-	565.04773	C15H24N2O17P2	Pyrimidine nucleotide sugars	HSCJRCZFDFQWRP-JZMIEXBBSA-N	OC[C=H]1O[C=H](OP(O)(=O)OP(O)(=O)OC[C=H]2O[C=H]([C=H](O)[C=H]2O)N2C=CC(O)=NC2=O)[C=H](O)[C=H](O)[C=H]1O
478	1.088	265.13943	9-hydroxyparthenolide	[M+H]+	265.14001	C15H20O4	Germacranolides and derivatives	RZUCCKARTVHQBW-CVZYAWEWSA-N	O=C1OC2C3OC3(C)CCC=C(C)C(O)CC2C1=C
479	1.088	213.02667	Juarezic Acid	[M+H]+	213.03123	C11H10O2	Styrenes	FEIQOMCWGDNMHM-KBXRYBNXSA-N	O=C(O)C=CC=CC=1C=CC=CC1
480	1.092	185.03188	Glutamine (L)	[M+H]+	185.0323	C5H10N2O3	L-alpha-amino acids	ZDXPYRJPNDTMRX-VKHMYHEASA-N	O=C(O)C(N)CCC(=N)O
481	1.094	113.05945	1,4-Cyclohexanedione	[M+H]+	113.0597	C6H8O2	Cyclic ketones	DCZFGQYXRKMVFG-UHFFFAOYSA-N	O=C1CCC(=O)CC1
482	1.095	606.07379	Uridine 5'-diphospho-N-acetylglucosamine	[M-H]-	606.07428	C17H27N3O17P2	Pyrimidine nucleotide sugars	LFTYTUAZOPRMMI-CFRASDGPSA-N	CC(O)=N[C=H]1[C=H](OP(O)(=O)OP(O)(=O)OC[C=H]2O[C=H]([C=H](O)[C=H]2O)N2C=CC(O)=NC2=O)O[C=H](CO)[C=H](O)[C=H]1
483	1.096	608.0863	Uridine-5-diphosphoacetylglucosamine	[M+H]+	608.08887	C17H27N3O17P2	Pyrimidine nucleotide sugars	LFTYTUAZOPRMMI-UHFFFAOYNA-N	O=C1N=C(O)C=CN1C2OC(COP(=O)(O)OP(=O)(O)OC3OC(CO)C(O)C(O)C3N=C(O)C)C(O)C2O
484	1.1	413.09427	2-amino-5-[2-[[2,3-dihydroxy-2-(1-hydroxyethyl)butanoyl]oxymethyl]-4-hydroxyanilino]-5-oxopentanoic acid	[M-H]-	413.0943	C18H26N2O9	Glutamine and derivatives	JMRJLJOBHHWYIV-UHFFFAOYSA-N	O=C(O)C(N)CCC(=O)NC1=CC=C(O)C=C1COC(=O)C(O)(C(O)C)C(O)C
485	1.1	127.03925	Dihydro-4,4-dimethyl-2,3-furandione	[M-H]-	127.04007	C6H8O3	Gamma butyrolactones	HRTOQFBQOFIFEE-UHFFFAOYSA-N	O=C1OCC(C1=O)(C)C
486	1.101	132.02939	D-ASPARTATE	[M-H]-	132.03	C4H7NO4	Aspartic acid and derivatives	CKLJMWTZIZZHCS-UHFFFAOYSA-N	O=C(O)CC(N)C(=O)O
487	1.102	356.08932	Balsalazide	[M-H]-	356.08881	C17H15N3O6	Beta amino acids and derivatives	KONZVQJABTUMFX-UHFFFAOYSA-N	O=C(O)C1=CC(=NNC2=CC=C(C=C2)C(=O)NCCC(=O)O)C=CC1=O
488	1.105	422.1037	(Z)-2-(2-methoxybenzylidene)-9-(pyridin-3-yl)-8,9-dihydro-2H-furo[2,3-f]chromene-3,7-dione	[M+H]+	422.10001	C24H17NO5	Aurone flavonoids	FEIHRQSTXZRGQD-JAIQZWGSSA-N	O=C1OC2=CC=C3C(=O)C(OC3=C2C(C=4C=NC=CC4)C1)=CC=5C=CC=CC5OC
489	1.105	164.09171	N,N-dimethyl-7H-purin-6-amine	[M+H]+	164.09	C7H9N5	6-alkylaminopurines	BVIAOQMSVZHOJM-UHFFFAOYSA-N	N1=CN=C(C=2NC=NC12)N(C)C
490	1.106	189.1602	"NEPSILON,NEPSILON,NEPSILON-TRIMETHYLLYSINE"	[M+H]+	189.16	C9H20N2O2	Alpha amino acids	MXNRLFUSFKVQSK-UHFFFAOYSA-N	O=C([O-])C(N)CCCC[N+](C)(C)C
491	1.106	291.11734	_120168	[M+H]+	291.12201	C16H18O5	Diphenylethers	WIOKWEJDRXNVSH-UHFFFAOYSA-N	OC=1C=C(OC)C=C(C1OC=2C=C(C=C(OC)C2O)C)C
492	1.106	723.19165	dimethyl 3,3'-(2,5-dihydroxy-3,6-dioxocyclohexa-1,4-diene-1,4-diyl)bis(3-(3-methoxy-4-(2-methoxy-2-oxoethoxy)phenyl)propanoate)	[M+H]+	723.19	C34H36O16	Dalbergiones	CIHGIKFGHBFZGP-UHFFFAOYSA-N	O=C(OC)COC1=CC=C(C=C1OC)C(C=2C(=O)C(O)=C(C(=O)C2O)C(C3=CC=C(OCC(=O)OC)C(OC)=C3)CC(=O)OC)CC(=O)OC
493	1.108	146.11807	DEOXYCARNITINE	[M+H]+	146.12	C7H15NO2	Straight chain fatty acids	JHPNVNIEXXLNTR-UHFFFAOYSA-N	O=C([O-])CCC[N+](C)(C)C
494	1.108	382.08154	Metsulfuron-methyl	[M+H]+	382.08157	C14H15N5O6S	Benzoic acid esters	RSMUVYRMZCOLBH-UHFFFAOYSA-N	COC(=O)C1=CC=CC=C1S(=O)(=O)N=C(O)N=C1NC(OC)=NC(C)=N1
495	1.108	139.05795	Salicylic Acid	[M+H]+	139.05215	C26H44O9	Medium-chain fatty acids	MINDHVHHQZYEEK-HBBNESRFSA-N	O=C(O)CCCCCCCCOC(=O)C=C(C)CC1OCC(CC2OC2C(C)C(O)C)C(O)C1O
496	1.109	161.09158	Ala-Ala	[M+H]+	161.09207	C6H12N2O3	Dipeptides	DEFJQIDDEAULHB-UHFFFAOYNA-N	O=C(O)C(N=C(O)C(N)C)C
497	1.111	337.10931	4'-hydroxy-2',4,6'-trimethoxychalcone	[M+H]+	337.10999	C18H18O5	Cinnamylphenols	FQZORWOCAANBNC-RMKNXTFCSA-N	O=C(C=CC1=CC=C(OC)C=C1)C=2C(OC)=CC(O)=CC2OC
498	1.111	266.08737	ADENOSINE	[M-H]-	266.08899	C10H13N5O4	Purine nucleosides	OIRDTQYFTABQOQ-UHFFFAOYSA-N	OCC1OC(N2C=NC=3C(=NC=NC32)N)C(O)C1O
499	1.111	624.13531	Quercetin-3,4'-O-di-beta-glucopyranoside	[M-2H]2-	624.13318	C27H30O17	Flavonoid-3-O-glycosides	RPVIQWDFJPYNJM-DEFKTLOSSA-N	OC[C=H]1O[C=H](OC2=C(O)C=C(C=C2)C2=C(O[C=H]3O[C=H](CO)[C=H](O)[C=H](O)[C=H]3O)C(=O)C3=C(O)C=C(O)C=C3O2)[C=H]
500	1.113	546.20062	4-oxo-8-propyl-1,2,3,4-tetrahydrocyclopenta[c]chromen-7-yl 2-(4-methylphenylsulfonamido)-3-phenylpropanoate	[M+H]+	546.20001	C31H31NO6S	Phenylalanine and derivatives	BPKLDKRUGJQLGK-UHFFFAOYSA-N	O=C1OC=2C=C(OC(=O)C(NS(=O)(=O)C3=CC=C(C=C3)C)CC=4C=CC=CC4)C(=CC2C5=C1CCC5)CCC
501	1.113	246.09019	Salsolidine	[M+H]+	246.0891	C12H17NO2	Tetrahydroisoquinolines	HMYJLVDKPJHJCF-UHFFFAOYSA-N	O(C=1C=C2C(=CC1OC)C(NCC2)C)C
502	1.115	149.00819	"(R,R)-TARTARIC ACID"	[M-H]-	149.009	C4H6O6	Sugar acids and derivatives	FEWJPZIEWOKRBE-UHFFFAOYSA-N	O=C(O)C(O)C(O)C(=O)O
503	1.116	267.10773	1-(3-carboxypropyl)-3,7-dimethylxanthine	[M+H]+	267.1088	C11H14N4O4	Xanthines	WKASGTGXOGALBG-UHFFFAOYSA-N	CN1C=NC2=C1C(N(CCCC(=O)O)C(N2C)=O)=O
504	1.117	294.08142	1-cyclopropyl-6,7-difluoro-1,4-dihydro-8-methoxy-4-oxo-3-Quinoline-3-carboxylic acid	[M-H]-	294.07999	C14H11F2NO4	Quinoline carboxylic acids	WQJZXSSAMGZVTM-UHFFFAOYSA-N	O=C(O)C1=CN(C2=C(OC)C(F)=C(F)C=C2C1=O)C3CC3
505	1.117	191.10196	DL-alpha,epsilon-Diaminopimelic acid	[M+H]+	191.10263	C7H14N2O4	Alpha amino acids	GMKMEZVLHJARHF-UHFFFAOYNA-N	NC(CCCC(N)C(O)=O)C(O)=O
506	1.117	543.13031	Mometasone furoate	[M+H]+	543.13098	C27H30Cl2O6	Gluco/mineralocorticoids, progestogins and	WOFMFGQZHJDGCX-RRDYVFDESA-N	O=C1C=CC2(C(=C1)CCC3C4CC(C)C(OC(=O)C=5OC=CC5)(C(=O)CCl)C4(C)CC(O)C32Cl)C
507	1.118	223.07399	Pesticide4_Acetamiprid_C10H11ClN4_(1E)-N-[(6-chloropyridin-3-yl)methyl]-N'-cyano-N-methylethanimidamide	[M+H]+	223.07401	C10H11ClN4	2-halopyridines	WCXDHFDTOYPNIE-RIYZIHGNSA-N	N#CN=C(N(C)CC1=CN=C(Cl)C=C1)C
508	1.119	273.08069	6,7-bis(hydroxymethyl)-4,9a-dimethyl-2,7,8,9-tetrahydro-1H-benzo[7]annulen-3-one	[M+H]+	273.08112	C15H22O3	Cyclohexenones	DPBOXKYWJVXMAW-UHFFFAOYSA-N	O=C1C(=C2C=C(CO)C(CO)CCC2(C)CC1)C
509	1.119	90.05459	beta-Alanine	[M+H]+	90.05495	C3H7NO2	Beta amino acids and derivatives	UCMIRNVEIXFBKS-UHFFFAOYSA-N	O=C(O)CCN
510	1.119	145.06117	L-GLUTAMINE	[M-H]-	145.061	C5H10N2O3	Alpha amino acids	ZDXPYRJPNDTMRX-UHFFFAOYSA-N	O=C(O)C(N)CCC(=O)N
511	1.12	165.04128	3-Methylxanthine	[M-H]-	165.04179	C6H6N4O2	Xanthines	GMSNIKWWOQHZGF-UHFFFAOYSA-N	CN1C2=C(N=CN2)C(O)=NC1=O
512	1.121	137.02353	4-HYDROXYBENZOATE	[M-H]-	137.024	C7H6O3	Hydroxybenzoic acid derivatives	FJKROLUGYXJWQN-UHFFFAOYSA-N	O=C(O)C1=CC=C(O)C=C1
513	1.122	209.0675	1,3,7-Trimethyluric acid	[M-H]-	209.06801	C8H10N4O3	Xanthines	BYXCFUMGEBZDDI-UHFFFAOYSA-N	O=C1C2=C(N=C(O)N2C)N(C(=O)N1C)C
514	1.122	382.1347	2-[[1-(4-nitrobenzoyl)pyrrolidine-2-carbonyl]amino]benzoic acid	[M-H]-	382.13391	C19H17N3O6	Acylaminobenzoic acid and derivatives	IXQCQOLLSMIABK-UHFFFAOYSA-N	O=C(O)C=1C=CC=CC1N=C(O)C2N(C(=O)C3=CC=C(C=C3)N(=O)=O)CCC2

515	1.123	504.18906	1,1'-azanediylbis(3-methyl-2H-naphtho[1,2,3-de]quinoline-2,7(3H)-dione) triacetate	[M+H]+	504.19	C40H33N3O10	Anthracenes	VWAIGFCJFBXWBL-UHFFFAOYSA-N	O=C(O)C.O=C(O)C.O=C(O)C.O=C1C=2C=CC=CC2C3=C(NC=4C(=O)N(C=5C=CC=C6C(=O)C=7C=CC=CC7C4C65)C)C(=O)N(C=8C=CC=C1C8
516	1.123	251.05205	Perseitol	[M+H]+	251.0528	C7H16O7	Sugar alcohols	OXQKEKGBFMQTML-UHFFFAOYSA-N	OCC(O)C(O)C(O)C(O)C(O)CO
517	1.124	483.10959	Parishin E	[M+H]+	483.10999	C19H24O13	Phenolic glycosides	XAIUTKHLNZBMEG-UHFFFAOYSA-N	O=C(O)CC(O)(C(=O)O)CC(=O)OCC1=CC=C(OC2OC(CO)C(O)C(O)C2O)C=C1
518	1.125	367.13229	(3E,12E)-3,12-dimethyl-8-methylidene-6,18-dioxatricyclo[14.2.1.0?,?]nonadeca-3,12,16(19)-triene-7,17-dione	[M+H]+	367.13062	C20H24O4	Diterpene lactones	KTYZKXFERQUCPX-SPUCVAIDSA-N	O=C1OC2C=C1CCC=C(C)CCC3C(=C)C(=O)OC3C=C(C)C2
519	1.125	305.08261	5,7-dimethoxy-2-phenyl-4H-chromen-4-one	[M+H]+	305.07999	C17H14O4	7-O-methylated flavonoids	JRFZSUMZAUHNSL-UHFFFAOYSA-N	O=C1C=C(OC2=CC(OC)=CC(OC)=C12)C=3C=CC=CC3
520	1.125	257.08453	Flavan-3-ols + 3O	[M+H]+	257.082	C15H14O5	Flavanol O-glycosides	RSYUFYQTACJFML-DDOUFSBJNA-N	OC1=CC=C(C=C1)C3OC=2C=C(O)C=C(O)C=2CC3(O)
521	1.125	321.05811	fuzhuanin A	[M+H]+	321.05899	C15H12O8	1-benzopyrans	QCFAYXITEXFSLB-QMTHXVAHSA-N	O=C(O)C=1OC(=O)C=C(C1)C2OC=3C=C(O)C=C(O)C3CC2O
522	1.125	616.20166	JACAGLABROSIDE B	[M+2H]2+	616.20245	C30H30O13	Saccharolipids	AIFVFVGRWAMEBM-GSQIWVKLSA-N	O=C1C=CC(O)(C=C1)CC(=O)OCC2OC(OC(=O)CC3(O)C=CC(=O)C=C3)C(OC(=O)CC=4C=CC=CC4)C(O)C2O
523	1.127	146.09229	4-Guanidinobutanoic acid	[M+H]+	146.09239	C5H11N3O2	Gamma amino acids and derivatives	TUHVEAJXIMEOSA-UHFFFAOYSA-N	C(CC(=O)O)CNC(=N)N
524	1.127	221.04111	4-hydroxy-3,5-dimethoxybenzoic acid	[M+Na]+	221.03999	C9H10O5	Gallic acid and derivatives	JMSVCTWVEWCHDZ-UHFFFAOYSA-N	O=C(O)C1=CC(OC)=C(O)C(OC)=C1
525	1.128	445.1189	baicalin	[M-H]-	445.12244	C21H18O11	Flavonoid-7-O-glucuronides	IKIIZLYTISPENI-ZFORQUDYSA-N	O=C(O)C1OC(OC=2C=C3OC(=CC(=O)C3=C(O)C2O)C=4C=CC=CC4)C(O)C(O)C1O
526	1.128	112.05007	Cytosine	[M+H]+	112.05054	C4H5N3O	Pyrimidones	OPTASPLRGRRNAP-UHFFFAOYSA-N	c1cnc([nH]c1=N)O
527	1.128	604.06958	Guanosine 5'-diphosphate-D-mannose	[M-H]-	604.06989	C16H25N5O16P2	Purine nucleotide sugars	MVMSCBBUIHUTGJ-GDJBGNAASA-N	OC[C=H]1O[C=H](OP(O)(=O)OP(O)(=O)OC[C=H]2O[C=H]([C=H](O)[C=H]2O)N2C=NC3=C2NC(=N)N=C3O)[C=H](O)[C=H](O)[C=H]1
528	1.128	193.07103	Methyl-beta-galactopyranoside	[M-H]-	193.07176	C7H14O6	O-glycosyl compounds	HOVAGTYPODGVJG-UHFFFAOYNA-N	OCC1OC(OC)C(O)C(O)C1O
529	1.13	522.20026	(2S,3S)-3-hydroxy-2-((S)-2-((S)-2-(2-hydroxy-4-oxoquinazolin-3(4H)-yl)-3-(1H-indol-3-yl)propanamido)propanamido)butanoic acid	[M+H]+	522.20001	C26H27N5O7	Oligopeptides	JZZOGGANLSAZTC-IMSILIBVSA-N	O=C(O)C(NC(=O)C(NC(=O)C(N1C(=O)C=2C=CC=CC2N=C1O)CC3=CNC=4C=CC=CC43)C)C(O)C
530	1.131	380.09595	Atalaphylline	[M+H]+	380.09744	C23H25NO4	Acridones	GLXYKTASIIUSRC-UHFFFAOYSA-N	O=C1C=2C=CC=C(O)C2NC3=C1C(O)=C(C(O)=C3CC=C(C)C)CC=C(C)C
531	1.132	462.17926	Morphine_3_Glucuronide	[M+H]+	462.17587	C23H27NO9	Morphinans	WAEXKFONHRHFBZ-UHFFFAOYNA-N	CN1CCC23C4OC5=C(OC6OC(C(O)C(O)C6O)C(O)=O)C=CC(CC1C2C=CC4O)=C35
532	1.132	151.06015	Xylitol	[M-H]-	151.0612	C5H12O5	Sugar alcohols	HEBKCHPVOIAQTA-UHFFFAOYNA-N	OCC(O)C(O)C(O)CO
533	1.133	253.0808	Osthenol	[M+H]+	253.08	C14H14O3	7-hydroxycoumarins	RAKJVIPCCGXHHS-UHFFFAOYSA-N	O=C1OC2=C(C=C1)C=CC(O)=C2CC=C(C)C
534	1.133	219.02779	THEOBROMINE	[M+H]+	219.02788	C7H8N4O2	Xanthines	YAPQBXQYLJRXSA-UHFFFAOYSA-N	O=C1N=C(O)C2=C(N=CN2C)N1C
535	1.133	203.05363	Theophylline	[M+H]+	203.05	C7H8N4O2	Xanthines	ZFXYFBGIUFBOJW-UHFFFAOYSA-N	O=C1C=2NC=NC2N(C(=O)N1C)C
536	1.133	149.04483	Xylose	[M-H]-	149.04555	C5H10O5	Pentoses	PYMYPHUHKUWMLA-VPENINKCSA-N	O=CC(O)C(O)C(O)CO
537	1.134	400.14685	2,6-dihydroxy-3-mesityl-5-(1,2,3,4-tetrahydroisoquinolin-1-yl)pyrimidin-4(3H)-one	[M+H]+	400.14999	C22H23N3O3	Tetrahydroisoquinolines	IOIBVYOJSLAKRI-UHFFFAOYSA-N	O=C1C(=C(O)N=C(O)N1C=2C(=CC(=CC2C)C)C)C3NCCC=4C=CC=CC43
538	1.135	221.06551	Ethyl-beta-glucuronide	[M-H]-	221.06668	C8H14O7	O-glucuronides	IWJBVMJWSPZNJH-UHFFFAOYNA-N	O=C(O)C1OC(OCC)C(O)C(O)C1O
539	1.136	274.1871	4-hydroxy-2-octylquinoline 1-oxide:Series 4 HAQ C8	[M+H]+	274.18201	C17H23NO2	4-hydroxy-2-alkylquinolines	UKKYWOFQJOMYIL-UHFFFAOYSA-N	O=N=1C=2C=CC=CC2C(O)=CC1CCCCCCCC
540	1.136	193.02975	Citric acid	[M+H]+	193.03	C6H8O7	Tricarboxylic acids and derivatives	KRKNYBCHXYNGOX-UHFFFAOYSA-N	O=C(O)CC(O)(C(=O)O)CC(=O)O
541	1.136	310.11194	N-Acetylneuraminic acid	[M+H]+	310.11325	C11H19NO9	N-acylneuraminic acids	SQVRNKJHWKZAKO-PFQGKNLYSA-N	CC(=N[C=H]1[C=H](C[C=](C(=O)O)(O)O[C=H]1[C=H]([C=H](CO)O)O)O)O
542	1.137	541.14294	[6-[3,4-dihydroxy-2,5-bis(hydroxymethyl)oxolan-2-yl]oxy-3,4,5-trihydroxyoxan-2-yl]methyl (E)-3-(4-hydroxy-3-methoxyphenyl)prop-2-enoate	[M+H]+	541.14386	C22H30O14	Coumaric acids and derivatives	XMBZZLUIFFOAHR-HWKANZROSA-N	O=C(OCC1OC(OC2(OC(CO)C(O)C2O)CO)C(O)C(O)C1O)C=CC3=CC=C(O)C(OC)=C3
543	1.137	305.13281	Octyl gallate	[M+H]+	305.13	C15H22O5	Galloyl esters	NRPKURNSADTHLJ-UHFFFAOYSA-N	O=C(OCCCCCCCC)C1=CC(O)=C(O)C(O)=C1
544	1.138	509.12595	Malvidin-3-O-beta-D-galactoside	[M-H]-	509.12952	C23H25O12	Anthocyanidin-3-O-glycosides	PXUQTDZNOHRWLI-XSEKTIEYSA-O	COC1=CC(=CC(OC)=C1O)C1=C(O[C=H]2O[C=H](CO)[C=H](O)[C=H](O)[C=H]2O)C=C2C(O)=CC(O)=CC2=[O+]1
545	1.138	361.04913	Vulpinic Acid	[M+H]+	361.04727	C19H14O5	Butenolides	OMZRMXULWNMRAE-BMRADRMJSA-N	O=C1OC(C(O)=C1C=2C=CC=CC2)=C(C(=O)OC)C=3C=CC=CC3
546	1.139	348.06934	ADENOSINE 5'-MONOPHOSPHATE	[M+H]+	348.07001	C10H14N5O7P	Purine ribonucleoside monophosphates	UDMBCSSLTHHNCD-UHFFFAOYSA-N	O=P(O)(O)OCC1OC(N2C=NC=3C(=NC=NC32)N)C(O)C1O
547	1.139	181.07068	Mannitol	[M-H]-	181.07176	C6H14O6	Sugar alcohols	FBPFZTCFMRRESA-UHFFFAOYNA-N	OCC(O)C(O)C(O)C(O)CO
548	1.14	235.12791	Asarylaldehyde	[M+H]+	235.12737	C10H12O4	Benzoyl derivatives	IAJBQAYHSQIQRE-UHFFFAOYSA-N	O=CC1=CC(OC)=C(OC)C=C1OC
549	1.14	222.02815	quinmerac	[M+H]+	222.03163	C11H8ClNO2	Quinoline carboxylic acids	ALZOLUNSQWINIR-UHFFFAOYSA-N	CC1=CC=2C=CC(=C(C2N=C1)C(=O)O)Cl
550	1.141	193.07028	Quinic acid	[M+H]+	193.07001	C7H12O6	Quinic acids and derivatives	AAWZDTNXLSGCEK-TUNDHVGDSA-N	O=C(O)C1(O)CC(O)C(O)C(O)C1
551	1.142	307.07846	(-)-Gallocatechin (GC)	[M+H]+	307.082	C15H14O7	Epigallocatechins	XMOCLSLCDHWDHP-DOMZBBRYSA-N	OC=1C=C(O)C2=C(OC(C3=CC(O)=C(O)C(O)=C3)C(O)C2)C1
552	1.142	151.03467	2-Mercaptobenzimidazole	[M+H]+	151.03244	C7H6N2S	Benzimidazoles	YHMYGUUIMTVXNW-UHFFFAOYSA-N	C1=CC=C2C(=C1)N=C(N2)S
553	1.143	411.08875	[3-(4-hydroxy-3-methoxybenzoyl)-2,3-dimethyloxiran-2-yl]-(4-hydroxy-3-methoxyphenyl)methanone	[M+H]+	411.08405	C20H20O7	Dibenzylbutane lignans	ZDJWTWORZULMCQ-UHFFFAOYSA-N	O=C(C1=CC=C(O)C(OC)=C1)C2(OC2(C(=O)C3=CC=C(O)C(OC)=C3)C)C
554	1.143	446.15009	2-((13-allyl-8-oxo-8H-benzo[c]indolo[3,2,1-ij][1,5]naphthyridin-12-yl)oxy)-N-cyclopropylacetamide	[M+H]+	446.14999	C26H21N3O3	Indolonaphthyridine alkaloids	BAVIFLQGTGNVOP-UHFFFAOYSA-N	O=C(NC1CC1)COC2=CC=C3C(C=4C=CN=C5C=6C=CC=CC6C(=O)N3C54)=C2CC=C
555	1.144	277.0318	3,5-dihydroxy-7-methoxy-6-methyl-1-oxo-3H-2-benzofuran-4-carboxylic acid	[M+H]+	277.03186	C11H10O7	M-phthalic acid and derivatives	MUGQOWYFWURKCM-UHFFFAOYSA-N	O=C(O)C=1C(O)=C(C(OC)=C2C(=O)OC(O)C12)C
556	1.145	79.02095	Dimethyl sulfoxide	[M+H]+	79.02121	C2H6OS	Sulfoxides	IAZDPXIOMUYVGZ-UHFFFAOYSA-N	O=S(C)C
557	1.145	118.05053	Homoserine	[M-H]-	118.05096	C4H9NO3	L-alpha-amino acids	UKAUYVFTDYCKQA-UHFFFAOYNA-N	O=C(O)C(N)CCO

558	1.145	275.12375	L-Saccharopine	[M-H]-	275.12485	C11H20N2O6	Glutamic acid and derivatives	ZDGJAHTZVHVLOT-YUMQZZPRSA-N	N[C=H](CCCCN[C=H](CCC(O)=O)C(O)=O)C(O)=O
559	1.147	317.11499	Heptelidic acid chlorohydrin_130129	[M+H]+	317.11499	C15H21ClO5	Terpene lactones	BJGUEPXNQKBVBW-RTWAVKEYSA-N	O=C(O)C1=CC2C(C(=O)OC1)C(O)(CCl)CCC2C(C)C
560	1.152	191.01877	ISOCITRIC ACID	[M-H]-	191.019	C6H8O7	Tricarboxylic acids and derivatives	ODBLHEXUDAPZAU-UHFFFAOYSA-N	O=C(O)CC(C(=O)O)C(O)C(=O)O
561	1.152	208.1178	Miglitol	[M+H]+	208.11795	C8H17NO5	Piperidines	IBAQFPQHRJAVAV-UHFFFAOYNA-N	OCCN1CC(O)C(O)C(O)C1CO
562	1.153	179.05449	Gluconolactone	[M+H]+	179.05501	C6H10O6	Gluconolactones	PHOQVHQSTUBQQK-UHFFFAOYNA-N	O=C1OC(CO)C(O)C(O)C1O
563	1.156	163.05965	1,6-Anhydro-beta-D-glucose	[M+H]+	163.0601	C6H10O5	Oxepanes	TWNIBLMWSKIRAT-VFUOTHLCSA-N	O[C=H]1[C=H]2CO[C=H](O2)[C=H](O)[C=H]1O
564	1.156	435.08917	Irisxanthone	[M-H]-	435.09329	C20H20O11	Xanthones	MTQVPZUZBBTLNO-HSLVGEKZSA-N	O=C1C2=CC=C(O)C(OC)=C2OC3=CC(O)=C(C(O)=C13)C4OC(CO)C(O)C(O)C4O
565	1.157	233.04103	2-pyridin-3-yl-thiazolidine-4-carboxylic acid	[M+H]+	233.03999	C9H10N2O2S	L-alpha-amino acids	FSNGLHIMQHWTNF-JGVFFNPUSA-N	O=C(O)C1NC(SC1)C=2C=NC=CC2
566	1.158	180.08737	Galactosamine	[M+H]+	180.08665	C6H13NO5	Hexoses	MSWZFWKMSRAUBD-UHFFFAOYNA-N	OCC1OC(O)C(N)C(O)C1O
567	1.158	97.02883	Propionic acid	[M+H]+	97.03	C3H6O2	Carboxylic acids	XBDQKXXYIPTUBI-UHFFFAOYSA-N	O=C(O)CC
568	1.159	184.05969	4-PYRIDOXATE	[M+H]+	184.06	C8H9NO4	Pyridinecarboxylic acids	HXACOUQIXZGNBF-UHFFFAOYSA-N	O=C(O)C1=C(O)C(=NC=C1CO)C
569	1.161	298.15143	(3aS,8aR)-1,3a,8-trimethyl-1,2,3,3a,8,8a-hexahydropyrrolo[2,3-b]indol-5-yl methylcarbamate	[M+H]+	298.14999	C15H21N3O2	Pyrroloindoles	PIJVFDBKTWXHHD-HIFRSBDPSA-N	O=C(OC1=CC=C2C(=C1)C3(C)CCN(C)C3N2C)NC
570	1.161	423.08878	5-hydroxy-6,8-dimethoxy-7-[(2S,3R,4S,5S,6R)-3,4,5-trihydroxy-6-(hydroxymethyl)oxan-2-yl]oxychromen-2-one	[M+H]+	423.08978	C17H20O11	Coumarin glycosides	KDWWYRPEBULRGU-KIZWGXMFSA-N	O=C1OC=2C(OC)=C(OC3OC(CO)C(O)C(O)C3O)C(OC)=C(O)C2C=C1
571	1.162	166.01382	"PYRIDINE-2,3-DICARBOXYLATE"	[M-H]-	166.01401	C7H5NO4	Pyridinecarboxylic acids	GJAWHXHKYYXBSV-UHFFFAOYSA-N	O=C(O)C1=NC=CC=C1C(=O)O
572	1.163	256.08228	Caramboxin	[M+H]+	256.082	C11H13NO6	Phenylalanine and derivatives	DYJIDGJHOLTGBO-ZETCQYMHSA-N	O=C(O)C1=C(O)C=C(OC)C=C1CC(N)C(=O)O
573	1.164	241.04602	7,8-dimethylalloxazine (lumichrome)	[M-H]-	241.04625	C12H10N4O2	Flavins	ZJTJUVIJVLLGSP-UHFFFAOYSA-N	OC=1N=C(O)C=2N=C3C=C(C(=CC3=NC2N1)C)C
574	1.169	170.10217	ADONITOL	[M+H]+	170.10229	C5H12O5	Sugar alcohols	HEBKCHPVOIAQTA-UHFFFAOYSA-N	OCC(O)C(O)C(O)CO
575	1.169	414.1597	narcotine	[M+H]+	414.16	C22H23NO7	Phthalide isoquinolines	AKNNEGZIBPJZJG-MSOLQXFVSA-N	O=C1OC(C2=CC=C(OC)C(OC)=C12)C3C4=C(OC)C=5OCOC5C=C4CCN3C
576	1.17	349.08817	(E)-3-[2-[(2S,3R,4S,5S,6R)-3,4,5-trihydroxy-6-(hydroxymethyl)oxan-2-yl]oxyphenyl]prop-2-enoic acid	[M+H]+	349.08939	C15H18O8	Phenolic glycosides	GVRIYIMNJGULCZ-ZMKUSUEASA-N	O=C(O)C=CC=1C=CC=CC1OC2OC(CO)C(O)C(O)C2O
577	1.171	350.0845	2-(2,6-dihydroxy-4-methoxycarbonylbenzoyl)-3-hydroxybenzoic acid	[M+H]+	350.08704	C16H12O8	Benzophenones	QPQRGIHXJVTGCW-UHFFFAOYSA-N	O=C(O)C=1C=CC=C(O)C1C(=O)C2=C(O)C=C(C=C2O)C(=O)OC
578	1.171	330.0582	3',5'-Cyclic AMP	[M+H]+	330.05978	C10H12N5O6P	3',5'-cyclic purine nucleotides	IVOMOUWHDPKRLL-KQYNXXCUSA-N	NC1=C2N=CN([C=H]3O[C=H]4COP(O)(=O)O[C=H]4[C=H]3O)C2=NC=N1
579	1.171	200.11229	Pyrimethanil	[M+H]+	200.11823	C12H13N3	Aniline and substituted anilines	ZLIBICFPKPWGIZ-UHFFFAOYSA-N	CC1=CC(C)=NC(NC2=CC=CC=C2)=N1
580	1.176	409.17883	Beclomethasone	[M+H]+	409.17801	C22H29ClO5	21-hydroxysteroids	NBMKJKDGKREAPL-WOOYFSPWSA-N	O=C1C=CC2(C(=C1)CCC3C4CC(C)C(O)(C(=O)CO)C4(C)CC(O)C32Cl)C
581	1.178	129.01866	4-Amino-2-chloropyridine	[M+H]+	129.02141	C5H5ClN2	Aminopyridines and derivatives	BLBDTBCGPHPIJK-UHFFFAOYSA-N	NC1=CC(Cl)=NC=C1
582	1.178	251.03645	Bisphenol S	[M+H]+	251.03726	C12H10O4S	Benzenesulfonyl compounds	VPWNQTHUCYMVMZ-UHFFFAOYSA-N	C=1C=C(C=CC1O)S(C2=CC=C(C=C2)O)(=O)=O
583	1.179	242.06512	1-(2-(1H-indol-3-yl)ethyl)thiourea	[M+H]+	242.07001	C11H13N3S	3-alkylindoles	HRBGONPRSGPFMT-UHFFFAOYSA-N	S=C(N)NCCC1=CNC=2C=CC=CC21
584	1.18	243.09708	beta-lapachone	[M+H]+	243.097	C15H14O3	Naphthopyranones	QZPQTZZNNJUOLS-UHFFFAOYSA-N	O=C1C(=O)C2=C(OC(C)(C)CC2)C=3C=CC=CC13
585	1.182	248.11403	1-Isothiocyanato-9-(methylsulfinyl)-nonane	[M+H]+	248.11374	C11H21NOS2	Sulfoxides	MQFLXLMNOHHPTC-UHFFFAOYNA-N	CS(=O)CCCCCCCCCN=C=S
586	1.183	300.12726	Lotaustralin	[M+H]+	300.12491	C11H19NO6	Cyanogenic glycosides	WEWBWVMTOYUPHH-GXUZYPEDSA-N	N#CC(OC1OC(CO)C(O)C(O)C1O)(C)CC
587	1.185	275.09473	(2R)-8-ethyl-7-hydroxy-5-methoxy-2-methyl-2,3-dihydrochromen-4-one	[M+H]+	275.09579	C13H16O4	Chromones	BPIMOIWHAHUBPJ-SSDOTTSWSA-N	O=C1C=2C(OC)=CC(O)=C(C2OC(C)C1)CC
588	1.185	73.02811	PROPENOIC ACID	[M+H]+	73.0284	C3H4O2	Acrylic acids	NIXOWILDQLNWCW-UHFFFAOYSA-N	OC(=O)C=C
589	1.187	213.05269	2,4-Dihydroxybenzophenone	[M-H]-	213.05573	C13H10O3	Benzophenones	ZXDDPOHVAMWLBH-UHFFFAOYSA-N	C1=CC=C(C=C1)C(C=2C=CC(=CC2O)O)=O
590	1.19	318.12933	ethyl 3-(2,4-dihydroxy-6-methylpyridin-3-yl)-3-(3-hydroxyphenyl)propanoate	[M+H]+	318.13	C17H19NO5	Pyridinones	YKJXWYJDQVADMO-UHFFFAOYSA-N	O=C(OCC)CC(C=1C=CC=C(O)C1)C2=C(O)N=C(C=C2O)C
591	1.191	244.12846	Agomelatine	[M+H]+	244.133	C15H17NO2	N-acetyl-2-arylethylamines	YJYPHIXNFHFHND-UHFFFAOYSA-N	O=C(NCCC1=CC=CC2=CC=C(OC)C=C21)C
592	1.191	320.14288	Norfloxacin (Norxacin)	[M+H]+	320.14099	C16H18FN3O3	Quinoline carboxylic acids	OGJPXUAPXNRGGI-UHFFFAOYSA-N	O=C(O)C1=CN(C2=CC(=C(F)C=C2C1=O)N3CCNCC3)CC
593	1.191	172.09665	terrein	[M+H]+	172.09682	C8H10O3	1,2-diols	MHOOPNKRBMHHEC-HZIBQTDNSA-N	O=C1C=C(C=CC)C(O)C1O
594	1.192	231.02568	3-[(1-Carboxyvinyl)oxy]benzoic acid	[M+H]+	231.02638	C10H8O5	Phenoxyacetic acid derivatives	HGVAHYJMDVROLE-UHFFFAOYSA-N	O=C(O)C(OC1=CC=CC(=C1)C(=O)O)=C
595	1.192	175.04327	4-Methylumbelliferone	[M-H]-	175.04201	C10H8O3	7-hydroxycoumarins	HSHNITRMYYLLCV-UHFFFAOYSA-N	O=C1OC=2C=C(O)C=CC2C(=C1)C
596	1.192	217.11894	5-isopropenyl-2-methyl-2-cyclohexen-1-yl acetate	[M+H]+	217.12	C12H18O2	Menthane monoterpenoids	YTHRBOFHFYZBRJ-VXGBXAGGSA-N	O=C(OC1C(=CCC(C(=C)C)C1)C)C
597	1.193	333.04517	Methoxyhaemoventosine	[M-H]-	333.04999	C16H14O8	Isochromanequinones	WPHIVUBMBWBSCK-SPHRHWQCSA-N	O=C1OC(C)C(OC)C2=C(O)C=3C(=O)C(OC)=CC(=O)C3C(O)=C12
598	1.194	283.09113	2-(5-formyl-3,4-dihydro-2,2-dimethyl-2H-pyran-6-yl)-Benzoic acid	[M+H]+	283.091	C15H14O3	Naphthopyranones	QZPQTZZNNJUOLS-UHFFFAOYSA-N	O=C1C(=O)C2=C(OC(C)(C)CC2)C=3C=CC=CC13
599	1.194	150.05768	Methionine	[M+H]+	150.058	C5H11NO2S	Methionine and derivatives	FFEARJCKVFRZRR-UHFFFAOYSA-N	O=C(O)C(N)CCSC
600	1.195	273.07199	5-Hydroxymethyluridine	[M-H]-	273.07281	C10H14N2O7	Pyrimidine nucleosides	VQAJJNQKTRZJIQ-UHFFFAOYNA-N	O=C1N=C(O)C(=CN1C2OC(CO)C(O)C2O)CO

601	1.195	312.11981	Dihydrolycorine	[M+H]+	312.12	C16H19NO4	Lycorine-type amaryllidaceae alkaloids	VJILFEGOWCJNIK-MGRBZGILSA-N	OC1CC2CCN3CC4=CC=5OCOC5C=C4C(C1O)C32
602	1.196	174.04013	N-ACETYL-L-ASPARTIC ACID	[M-H]-	174.03999	C6H9NO5	Aspartic acid and derivatives	OTCCIMWXFLJLIA-BYPYZUCNSA-N	O=C(O)CC(NC(=O)C)C(=O)O
603	1.196	172.05978	Patulin	[M+H]+	172.06042	C7H6O4	Pyrans	ZRWPUFFVAOMMNM-UHFFFAOYSA-N	O=C1OC2=CCOC(O)C2=C1
604	1.197	302.13315	Pesticide4_Metalaxyl_C15H21NO4_N-(2,6-Dimethylphenyl)-N-(methoxyacetyl)-DL-alanine methyl ester	[M+H]+	302.13599	C15H21NO4	Alpha amino acid esters	ZQEIXNIJLIKNTD-UHFFFAOYSA-N	O=C(OC)C(N(C(=O)COC)C=1C(=CC=CC1C)C)C
605	1.198	230.0654	TES	[M+H]+	230.06929	C6H15NO6S	Organosulfonic acids	JOCBASBOOFNAJA-UHFFFAOYSA-N	O=S(=O)(O)CCNC(CO)(CO)CO
606	1.199	226.07025	1h-indole-3-butanoic acid	[M+H]+	226.08	C12H13NO2	3-alkylindoles	JTEDVYBZBROSJT-UHFFFAOYSA-N	O=C(O)CCCC1=CNC=2C=CC=CC21
607	1.199	196.05981	N-Acetyl Mesalazine	[M+H]+	196.06044	C9H9NO4	Acylaminobenzoic acid and derivatives	GEFDRROBUCULOD-UHFFFAOYSA-N	CC(=NC=1C=CC(=C(C1)C(=O)O)O)O
608	1.199	153.04022	Xanthine	[M+H]+	153.0407	C5H4N4O2	Xanthines	LRFVTYWOQMYALW-UHFFFAOYSA-N	c1nc2c([nH]1)nc(nc2O)O
609	1.201	139.0499	UROCANATE	[M+H]+	139.05	C6H6N2O2	Imidazolyl carboxylic acids and derivatives	LOIYMIARKYCTBW-UHFFFAOYSA-N	O=C(O)C=CC1=CN=CN1
610	1.202	297.08325	2',4',6'-Trihydroxydihydrochalcone	[M+H]+	297.08313	C15H14O4	2'-Hydroxy-dihydrochalcones	QWQGMMRHTWIOGH-UHFFFAOYSA-N	O=C(C=1C(O)=CC(O)=CC1O)CCC=2C=CC=CC2
611	1.202	230.09502	Ergothioneine	[M+H]+	230.09578	C9H15N3O2S	Histidine and derivatives	SSISHJJTAXXQAX-UHFFFAOYNA-N	O=C(O)C(CC1=CN=C([S-])N1)[N+](C)(C)C
612	1.203	145.01317	2-Oxoglutarate	[M-H]-	145.01425	C5H6O5	Gamma-keto acids and derivatives	KPGXRSRHYNQIFN-UHFFFAOYSA-N	OC(=O)CCC(=O)C(O)=O
613	1.204	219.13463	Serylleucine	[M+H]+	219.1346	C9H18N2O4	Dipeptides	NFDYGNFETJVMSE-UHFFFAOYNA-N	O=C(O)C(NC(=O)C(N)CO)CC(C)C
614	1.206	294.11911	N-Acetylmuramic Acid	[M+H]+	294.11835	C11H19NO8	Acylaminosugars	MNLRQHMNZILYPY-MDMHTWEWSA-N	O=C(O)C(OC1C(O)C(OC(O)C1N=C(O)C)CO)C
615	1.206	113.03416	Uracil	[M+H]+	113.03455	C4H4N2O2	Pyrimidones	ISAKRJDGNUQOIC-UHFFFAOYSA-N	OC1=NC=CC(O)=N1
616	1.208	123.04363	4-Hydroxybenzaldehyde	[M+H]+	123.04405	C7H6O2	Hydroxybenzaldehydes	RGHHSNMVTDWUBI-UHFFFAOYSA-N	O=CC1=CC=C(O)C=C1
617	1.209	231.09647	3,4-dihydro-2,2-dimethyl-pyrano[3,2-c][2]benzopyran-6(2H)-one	[M+H]+	231.097	C14H14O3	Isocoumarins and derivatives	NHUYGTCSYVBPOA-UHFFFAOYSA-N	O=C1OC2=C(OC(C)(C)CC2)C=3C=CC=CC13
618	1.209	324.13004	BETA-Febrifugine	[M+H]+	324.13	C16H19N3O3	Quinazolines	FWVHWDSCPKXMDB-GJZGRUSLSA-N	O=C1C=2C=CC=CC2N=CN1CC(=O)CC3NCCCC3O
619	1.209	326.13281	Hyoscine	[M+H]+	326.13	C17H21NO4	Beta hydroxy acids and derivatives	STECJAGHUSJQJN-SDODBCEQSA-N	O=C(OC1CC2N(C)C(C1)C3OC32)C(C=4C=CC=CC4)CO
620	1.209	351.17471	Posaconazole	[M+H]+	351.173	C37H42F2N8O4	Phenylpiperazines	RAGOYPUPXAKGKH-XAKZXMRKSA-N	O=C1N(N=CN1C2=CC=C(C=C2)N3CCN(C4=CC=C(OCC5COC(C6=CC=C(F)C=C6F)(CN7N=CN=C7)C5)C=C4)CC3)C(CC)C(O)C
621	1.209	243.06142	Uridine	[M-H]-	243.06226	C9H12N2O6	Pyrimidine nucleosides	DRTQHJPVMGBUCF-XVFCMESISA-N	OC[C=H]1O[C=H]([C=H](O)[C=H]1O)N1C=CC(O)=NC1=O
622	1.21	623.20172	[(2R,3R,4S,5R,6R)-6-[2-(3,4-dihydroxyphenyl)ethoxy]-3,5-dihydroxy-4-[(3R,4R,5R,6S)-3,4,5-trihydroxy-6-methyloxan-2-yl]oxyoxan-2-yl]methyl (E)-	[M-H]-	623.19812	C29H36O15	Coumaric acids and derivatives	FNMHEHXNBNCPCI-KAUJWNRDSA-N	O=C(OCC1OC(OCCC2=CC=C(O)C(O)=C2)C(O)C(OC3OC(C)C(O)C(O)C3O)C1O)C=CC4=CC=C(O)C(O)=C4
623	1.21	273.10886	8-hydroxy-11-(hydroxymethyl)-1,5,11-trimethyltricyclo[6.2.1.0?,?]undec-2-en-9-one	[M+H]+	273.10822	C15H22O3	Tertiary alcohols	LUADJIBUFOIMNV-UHFFFAOYSA-N	O=C1CC2(C3=CCC(C)C3CC1(O)C2(C)CO)C
624	1.21	472.1543	Folinic acid	[M-H]-	472.15863	C20H23N7O7	Tetrahydrofolic acids	VVIAGPKUTFNRDU-UHFFFAOYNA-N	O=CN1C=2C(O)=NC(=N)NC2NCC1CNC3=CC=C(C=C3)C(=O)NC(C(=O)O)CCC(=O)O
625	1.21	392.16776	nortrachelogenin	[M+H]+	392.17038	C20H22O7	Dibenzylbutyrolactone lignans	ZITBJWXLODLDRH-UHFFFAOYSA-N	O=C1OCC(CC2=CC=C(O)C(OC)=C2)C1(O)CC3=CC=C(O)C(OC)=C3
626	1.211	346.05222	Guanosine cyclic monophosphate	[M+H]+	346.05469	C10H12N5O7P	3',5'-cyclic purine nucleotides	ZOOGRGPOEVQQDX-UUOKFMHZSA-N	O=P1(O)OCC2OC(N3C=NC=4C(O)=NC(=N)NC43)C(O)C2O1
627	1.212	383.08286	Hispidulin acetate	[M-H]-	383.07724	C22H18O9	6-O-methylated flavonoids	HIOZFEXHRPPNDN-UHFFFAOYSA-N	COC1=C(OC(C)=O)C=C2OC(=CC(=O)C2=C1OC(C)=O)C1=CC=C(OC(C)=O)C=C1
628	1.213	496.20001	(2R,3S,4S,5R,6S)-2-[(3,4,5-trihydroxyoxan-2-yl)oxymethyl]-6-(3,4,5-trimethoxyphenoxy)oxane-3,4,5-triol	[M+H]+	496.20245	C20H30O13	Phenolic glycosides	GGIDHIBVLYVTAU-QGPLIYBISA-N	OC1COC(OCC2OC(OC=3C=C(OC)C(OC)=C(OC)C3)C(O)C(O)C2O)C(O)C1O
629	1.213	225.12236	Atenolol-desisopropyl	[M+H]+	225.12337	C11H16N2O3	Phenylacetamides	UWMXVJVTKRSOPW-UHFFFAOYNA-N	NCC(O)COC1=CC=C(CC(O)=N)C=C1
630	1.213	171.11238	Levetiracetam	[M+H]+	171.11281	C8H14N2O2	Alpha amino acids and derivatives	HPHUVLMMVZITSG-UHFFFAOYNA-N	O=C1N(CCC1)C(C(=N)O)CC
631	1.214	270.09552	Linamarin	[M+H]+	270.09253	C10H17NO6	Cyanogenic glycosides	QLTCHMYAEJEXBT-ZEBDFXRSSA-N	N#CC(OC1OC(CO)C(O)C(O)C1O)(C)C
632	1.215	406.16959	geniposide	[M+H]+	406.17078	C17H24O10	Iridoid O-glycosides	IBFYXTRXDNAPMM-BVTMAQQCSA-N	O=C(OC)C1=COC(OC2OC(CO)C(O)C(O)C2O)C3C(=CCC13)CO
633	1.215	443.1647	methyl 10-acetyloxy-4,5,9-trihydroxy-1,4a-dimethyl-7-propan-2-yl-2,3,4,9,10,10a-hexahydrophenanthrene-1-carboxylate	[M+H]+	443.16708	C23H32O7	Diterpenoids	WBEFPLHFVLPKHQ-UHFFFAOYSA-N	O=C(OC1C(O)C2=CC(=CC(O)=C2C3(C)C(O)CCC(C(=O)OC)(C)C13)C(C)C)C
634	1.216	289.10376	(E)-5-(4-methoxy-5-methyl-6-oxopyran-2-yl)-3-methylhex-4-enoic acid	[M+H]+	289.10464	C14H18O5	Medium-chain fatty acids	MPAYQRIVOSEDQH-WEVVVXLNSA-N	O=C1OC(=CC(OC)=C1C)C(=CC(C)CC(=O)O)C
635	1.217	253.04019	(E)-2-(hydroxymethyl)-3-[3-(hydroxymethyl)-6-propan-2-ylcyclohex-2-en-1-yl]prop-2-enoic acid	[M-H]-	253.0407	C14H22O4	Menthane monoterpenoids	SLVSUVFUFJKMCV-WUXMJOGZSA-N	O=C(O)C(=CC1C=C(CO)CCC1C(C)C)CO
636	1.217	257.11221	5'-O-Methylthymidine	[M+H]+	257.11319	C11H16N2O5	Pyrimidine 2'-deoxyribonucleosides	KTCKNHCDUKONFQ-UHFFFAOYNA-N	O=C1N=C(O)C(=CN1C2OC(COC)C(O)C2)C
637	1.217	138.01888	6-Hydroxynicotinic acid	[M-H]-	138.01967	C6H5NO3	Pyridinecarboxylic acids	BLHCMGRVFXRYRN-UHFFFAOYSA-N	OC(=O)C1=CN=C(O)C=C1
638	1.217	150.05527	Acetominophen	[M-H]-	150.05605	C8H9NO2	1-hydroxy-2-unsubstituted benzenoids	RZVAJINKPMORJF-UHFFFAOYSA-N	OC1=CC=C(N=C(O)C)C=C1
639	1.218	182.99229	Carbamimidothioic acid	[M-H]-	182.99001	C3H8N2O3S2	Organosulfonic acids	LTHWZZOUNJCHES-UHFFFAOYSA-N	O=S(=O)(O)CCSC(=N)N
640	1.218	121.06429	Tyramine	[M+H]+	121.0645	C8H11NO	Amino acids	DZGWFCGJZKJUFP-UHFFFAOYSA-N	OC1=CC=C(C=C1)CCN
641	1.22	275.17053	[(1S,2S,5R)-2-hydroxy-2-methyl-5-prop-1-en-2-ylcyclohexyl] benzoate	[M+H]+	275.16415	C17H22O3	Aromatic monoterpenoids	KGTUUJMNKKRNKR-VYDXJSESSA-N	O=C(OC1CC(C(=C)C)CCC1(O)C)C=2C=CC=CC2
642	1.22	288.202	2-heptylquinoline-3,4-diol:Pseudomonas quinolone signal:PQS:Series 3 HAQ C7	[M+H]+	288.19699	C16H21NO2	Hydroxyquinolones	CEIUIHOQDSVZJQ-UHFFFAOYSA-N	O=C1C(O)=C(NC=2C=CC=CC12)CCCCCCC
643	1.22	462.12256	Scopolamine butylbromide	[M+H]+	462.12	C21H30BrNO4	Beta hydroxy acids and derivatives	HOZOZZFCZRXYEK-TWJYCNPASA-M	[Br-].O=C(OC1CC2C3OC3C(C1)[N+]2(C)CCCC)C(C=4C=CC=CC4)CO

644	1.22	269.15915	sophocarpine	[M+H]+	269.16	C15H22N2O	Matrine alkaloids	AAGFPTSOPGCENQ-KYEXWDHISA-N	O=C1C=CCC2N1CC3CCCN4CCCC2C43
645	1.221	213.12245	Prolylproline	[M+H]+	213.12489	C10H16N2O3	Dipeptides	RWCOTTLHDJWHRS-UHFFFAOYNA-N	O=C(O)C2N(C(=O)C1NCCC1)CCC2
646	1.223	344.03741	"GUANOSINE 3',5'-CYCLIC MONOPHOSPHATE"	[M-H]-	344.04001	C10H12N5O7P	3',5'-cyclic purine nucleotides	ZOOGRGPOEVQQDX-UUOKFMHZSA-N	O=C1N=C(N)NC2=C1N=CN2C3OC4COP(=O)(O)OC4C3O
647	1.223	247.05748	Haematommic Acid, Ethyl Ester	[M+H]+	247.05769	C11H12O5	p-Hydroxybenzoic acid alkyl esters	HUXJGSHUVDWZAM-UHFFFAOYSA-N	O=CC1=C(O)C=C(C(C(=O)OCC)=C1O)C
648	1.225	163.06049	2-DEOXY-D-GLUCOSE	[M-H]-	163.061	C6H12O5	Fatty alcohols	VRYALKFFQXWPIH-UHFFFAOYSA-N	O=CCC(O)C(O)C(O)CO
649	1.225	391.17041	Pseudolaric Acid C	[M+H]+	391.17001	C21H26O7	Diterpene lactones	RBXVTEUAOTYIME-GPGKBOPFSA-N	O=C(O)C(=CC=CC1(OC(=O)C23CC=C(C(=O)OC)CCC3(O)C1CC2)C)C
650	1.226	375.17523	3-[(E)-3,4-dihydroxy-3,5-dimethylhept-1-enyl]-7,8-dihydroxy-7-methyl-8,8a-dihydro-1H-isochromen-6-one	[M+H]+	375.1778	C19H28O6	Azaphilones	ZMOXNUGOJSHBRR-VOTSOKGWSA-N	O=C1C=C2C=C(OCC2C(O)C1(O)C)C=CC(O)(C)C(O)C(C)CC
651	1.226	241.1554	Alpha Cyperone	[M+Na]+	241.14999	C15H22O	Eudesmane, isoeudesmane or cycloeudesmane	KUFXJZXMWHNCEH-DOMZBBRYSA-N	O=C1C(=C2CC(C(=C)C)CCC2(C)CC1)C
652	1.228	308.09705	N-ACETYLNEURAMINATE	[M-H]-	308.09799	C11H19NO9	N-acylneuraminic acids	SQVRNKJHWKZAKO-UHFFFAOYSA-N	O=C(O)C1(O)OC(C(O)C(O)CO)C(NC(=O)C)C(O)C1
653	1.229	160.06078	ALPHA-AMINOADIPATE	[M-H]-	160.061	C6H11NO4	L-alpha-amino acids	OYIFNHCXNCRBQI-BYPYZUCNSA-N	O=C(O)CCCC(N)C(=O)O
654	1.229	178.04991	HIPPURATE	[M-H]-	178.05	C9H9NO3	Hippuric acids	QIAFMBKCNZACKA-UHFFFAOYSA-N	O=C(O)CNC(=O)C=1C=CC=CC1
655	1.23	280.10553	1-Methyladenosine	[M-H]-	280.10513	C11H15N5O4	Purine nucleosides	GFYLSDSUCHVORB-UHFFFAOYNA-N	N=C1C=2N=CN(C2N=CN1C)C3OC(CO)C(O)C3O
656	1.23	306.08517	PFSM-ammonio_N_CMAmP_FT_FOSA	[M-H]-	306.08667	[C9H18F3N2O4S]+	PFSA	UBADCRSMADBWHM-UHFFFAOYSA-O	O=C(O)CC[N+](C)(C)CCCNS(=O)(=O)C(F)(F)F
657	1.231	116.03471	N-ACETYLGLYCINE	[M-H]-	116.035	C4H7NO3	N-acyl-alpha amino acids	OKJIRPAQVSHGFK-UHFFFAOYSA-N	O=C(O)CNC(=O)C
658	1.232	298.11313	7-Methylguanosine	[M+H]+	298.11404	C11H16N5O5	Purine nucleosides	OGHAROSJZRTIOK-UHFFFAOYNA-O	N=C1N=C(O)C2=C(N1)N(C=[N+]2C)C3OC(CO)C(O)C3O
659	1.232	68.05067	Pyrrol	[M+H]+	68.04948	C4H5N	Heteroaromatic compounds	KAESVJOAVNADME-UHFFFAOYSA-N	C=1C=CNC1
660	1.235	321.04709	THYMIDINE 5'-MONOPHOSPHATE	[M-H]-	321.04901	C10H15N2O8P	Pyrimidine 2'-deoxyribonucleoside	GYOZYWVXFNDGLU-UHFFFAOYSA-N	O=C1NC(=O)N(C=C1C)C2OC(COP(=O)(O)O)C(O)C2
661	1.237	273.06055	Pesticide3_Neburon_C12H16Cl2N2O_Urea, N-butyl-N'-(3,4-dichlorophenyl)-N-methyl-	[M-H]-	273.05701	C12H16Cl2N2O	N-phenylureas	CCGPUGMWYLICGL-UHFFFAOYSA-N	ClC1=CC=C(N=C(O)N(C)CCCC)C=C1Cl
662	1.238	363.06802	Esculin	[M+H]+	363.07001	C15H16O9	Coumarin glycosides	XHCADAYNFIFUHF-TVKJYDDYSA-N	O=C1OC=2C=C(O)C(OC3OC(CO)C(O)C(O)C3O)=CC2C=C1
663	1.239	212.09132	isoferulic acid	[M+H]+	212.09174	C10H10O4	Hydroxycinnamic acids	QURCVMIEKCOAJU-HWKANZROSA-N	O=C(O)C=CC1=CC=C(OC)C(O)=C1
664	1.24	542.14038	(S,Z)-2-(2-((2-(3,4-dimethoxybenzylidene)-3-oxo-2,3-dihydrobenzofuran-6-yl)oxy)acetamido)-3-(4-hydroxyphenyl)propanoic acid	[M+H]+	542.14001	C28H25NO9	Tyrosine and derivatives	ZOLBWQPEZBEHMV-CLVGQAISSA-N	O=C(O)C(NC(=O)COC1=CC=C2C(=O)C(OC2=C1)=CC3=CC=C(OC)C(OC)=C3)CC4=CC=C(O)C=C4
665	1.24	300.1174	Octopamine, N-p-coumaroyl-	[M+H]+	300.12	C17H17NO4	Styrenes	VATOSFCFMOPAHX-XCVCLJGOSA-N	O=C(C=CC1=CC=C(O)C=C1)NCC(O)C2=CC=C(O)C=C2
666	1.242	233.14844	Threonylleucine (isomer of 925, 926)	[M+H]+	233.1485	C10H20N2O4	Dipeptides	BQBCIBCLXBKYHW-UHFFFAOYNA-N	O=C(O)C(NC(=O)C(N)C(O)C)CC(C)C
667	1.243	108.04404	2-Aminophenol	[M+H]+	108.04549	C6H7NO	Aniline and substituted anilines	CDAWCLOXVUBKRW-UHFFFAOYSA-N	OC=1C=CC=CC1N
668	1.243	263.0542	maclurin	[M+H]+	263.06	C13H10O6	Benzophenones	XNWPXDGRBWJIES-UHFFFAOYSA-N	O=C(C1=CC=C(O)C(O)=C1)C=2C(O)=CC(O)=CC2O
669	1.244	297.10928	4-hydroxy-8-methoxy-5-methyl-3-(3-methylbut-2-enyl)chromen-2-one	[M+H]+	297.10971	C16H18O4	4-hydroxycoumarins	YPKUMLKVFXFYOT-UHFFFAOYSA-N	O=C1OC2=C(OC)C=CC(=C2C(O)=C1CC=C(C)C)C
670	1.244	201.08812	Methyleugenol	[M+H]+	201.09	C11H14O2	Dimethoxybenzenes	ZYEMGPIYFIJGTP-UHFFFAOYSA-N	O(C1=CC=C(C=C1OC)CC=C)C
671	1.245	133.04996	DEOXYRIBOSE	[M-H]-	133.05	C5H10O4	Beta-hydroxy aldehydes	ASJSAQIRZKANQN-UHFFFAOYSA-N	O=CCC(O)C(O)CO
672	1.25	171.02905	3-DEHYDROSHIKIMATE	[M-H]-	171.02901	C7H8O5	Cyclohexenones	SLWWJZMPHJJOPH-PHDIDXHHSA-N	O=C(O)C1=CC(=O)C(O)C(O)C1
673	1.25	155.0811	Cyclo-prolylglycine	[M+H]+	155.0815	C7H10N2O2	Amino acids	OWOHLURDBZHNGG-UHFFFAOYNA-N	O=C1CNC(=O)C2CCCN12
674	1.25	215.10204	LEVODOPA	[M+H]+	215.10263	C9H11NO4	Tyrosine and derivatives	WTDRDQBEARUVNC-LURJTMIESA-N	O=C(O)C(N)CC1=CC=C(O)C(O)=C1
675	1.25	154.04971	N-(2,5-dioxocyclopentyl)acetamide	[M-H]-	154.049	C7H9NO3	Beta-diketones	QVZXVDWBZHYCGL-UHFFFAOYSA-N	O=C1CCC(=O)C1N=C(O)C
676	1.252	283.13013	(2S,8R)-8-hydroxy-2-[(1S)-1-hydroxyheptyl]-2,3,4,6,7,8-hexahydrochromen-5-one	[M+H]+	283.13016	C16H26O4	Benzopyrans	INHVGPIPHZJQOP-ZNMIVQPWSA-N	O=C1C2=C(OC(CC2)C(O)CCCCCC)C(O)CC1
677	1.257	271.04425	alternariol monomethyl ether	[M-H]-	271.04236	C15H12O5	Coumarins and derivatives	LCSDQFNUYFTXMT-UHFFFAOYSA-N	O=C1OC2=CC(O)=CC(=C2C3=CC(OC)=CC(O)=C13)C
678	1.257	411.09671	Artemitin	[M+H]+	411.10001	C20H20O8	7-O-methylated flavonoids	RIGYMJVFEJNCKD-UHFFFAOYSA-N	O=C1C(OC)=C(OC=2C=C(OC)C(OC)=C(O)C12)C=3C=CC(OC)=C(OC)C3
679	1.257	388.12189	fraxin	[M+H]+	388.12381	C16H18O10	Coumarin glycosides	CRSFLLTWRCYNNX-QBNNUVSCSA-N	O=C1OC=2C(OC3OC(CO)C(O)C(O)C3O)=C(O)C(OC)=CC2C=C1
680	1.258	229.03406	Asulam	[M-H]-	229.02885	C8H10N2O4S	Aminobenzenesulfonamides	VGPYEHKOIGNJKV-UHFFFAOYSA-N	COC(O)=NS(=O)(=O)C1=CC=C(N)C=C1
681	1.264	423.19849	N-Fructosyl isoleucylglutamate	[M+H]+	423.1994	C17H30N2O10	N-Fructosyl peptides	OJDUYMYADCXNAN-UHFFFAOYNA-N	O=C(O)CCC(NC(=O)C(NCC1(O)(OCC(O)C(O)C1(O)))C(C)CC)C(=O)O
682	1.265	274.05573	Flufenacet ESA	[M-H]-	274.05548	C11H14FNO4S	Anilides	SZCMHDLOUVZYST-UHFFFAOYSA-N	CC(C)N(C(=O)CS(O)(=O)=O)C1=CC=C(F)C=C1
683	1.266	229.08144	Dihydroresveratrol	[M-H]-	229.08702	C14H14O3	Stilbene glycosides	HITJFUSPLYBJPE-UHFFFAOYSA-N	OC1=CC=C(C=C1)CCC2=CC(O)=CC(O)=C2
684	1.268	403.1062	2-[3-ethenyl-5-methoxycarbonyl-2-[(2S,3R,4S,5S,6R)-3,4,5-trihydroxy-6-(hydroxymethyl)oxan-2-yl]oxy-3,4-dihydro-2H-pyran-4-yl]acetic acid	[M-H]-	403.10376	C17H24O11	Terpene glycosides	MQLSOVRLZHTATK-WNCYHATQSA-N	O=C(O)CC1C(=COC(OC2OC(CO)C(O)C(O)C2O)C1C=C)C(=O)OC
685	1.271	453.13568	bungeiside C	[M+H]+	453.13672	C19H26O11	Phenolic glycosides	YQOKGDRMWQLQNR-BMVMOQKNSA-N	O=C(C1=CC=C(OC2OC(COC3OCC(O)C(O)C3O)C(O)C(O)C2O)C=C1)C
686	1.271	303.0462	Flavanone base + 5O	[M-H]-	303.0498	C15H12O7	Flavanone O-glycosides	CXQWRCVTCMQVQX-UHFFFAOYNA-N	O=C2C3=C(O)C=C(O)C=C3(OC(C=1C=CC(O)=C(O)C=1)C2(O))

687	1.272	347.14658	[(1R,5S,6S)-5-acetyloxy-3-(hydroxymethyl)-2-oxo-6-propan-2-ylcyclohex-3-en-1-yl] (E)-2-methylbut-2-enoate	[M+H]+	347.14648	C17H24O6	Menthane monoterpenoids	NUENJLVLKHHYJS-KOPIJTNESA-N	O=C(OC1C(=O)C(=CC(OC(=O)C)C1C(C)C)CO)C(=CC)C
688	1.273	445.14679	(1R,3R,4S,6S,8R,9R,10S,11S)-9-(acetyloxy)-8-hydroxy-3,8-dimethyl-12-methylidene-7,13-dioxo-5,14-dioxatricyclo[9.3.0.0?,?]tetradecan-10-yl 2-	[M+H]+	445.14688	C21H26O9	Germacranolides and derivatives	BRBRVKOYZAMSBL-PXFMFDQNSA-N	O=C(OC1C(OC(=O)C)C(O)(C(=O)C2OC2C(C)CC3OC(=O)C(=C)C31)C)C(=C)C
689	1.275	85.02887	1,4-Butynediol	[M-H]-	85.0295	C4H6O2	Primary alcohols	DLDJFQGPPSQZKI-UHFFFAOYSA-N	OCC#CCO
690	1.275	446.14536	Rutilantinone	[M+H]+	446.14456	C22H20O9	Tetracenequinones	RWCVSDKDFSVZNF-KRYGIPSASA-N	O=C1C=2C=C3C(=C(O)C2C(=O)C=4C(O)=CC=C(O)C14)C(O)CC(O)(CC)C3C(=O)OC
691	1.276	102.05531	4-AMINOBUTANOATE	[M-H]-	102.056	C4H9NO2	Gamma amino acids and derivatives	BTCSSZJGUNDROE-UHFFFAOYSA-N	O=C(O)CCCN
692	1.278	239.14006	Atractylon	[M+H]+	239.14	C15H20O	Eremophilane, 8,9-secoeremophilane and	TYPSVDGIQAOBAD-DZGCQCFKSA-N	O1C=C(C2=C1CC3(C)CCCC(=C)C3C2)C
693	1.279	310.14813	(3R)-4,4-dimethyl-3-[(2S,3R,4S,5S,6R)-3,4,5-trihydroxy-6-(hydroxymethyl)oxan-2-yl]oxyoxolan-2-one	[M+H]+	310.14963	C12H20O8	Fatty acyl glycosides of mono- and disaccharides	BFCZPWYLRHFBNO-DTJAAUDXSA-N	O=C1OCC(C)(C)C1OC2OC(CO)C(O)C(O)C2O
694	1.283	129.01839	Mesaconic acid	[M-H]-	129.01933	C5H6O4	Methyl-branched fatty acids	HNEGQIOMVPPMNR-NSCUHMNNSA-N	C\C(=C/C(O)=O)C(O)=O
695	1.283	445.20892	Mitoxantrone Hydrochloride	[M+H]+	445.20801	C22H30Cl2N4O6	Anthraquinones	ZAHQPTJLOCWVPG-UHFFFAOYSA-N	Cl.Cl.O=C1C=2C(O)=CC=C(O)C2C(=O)C=3C(=CC=C(NCCNCCO)C13)NCCNCCO
696	1.286	461.04672	Kaempferol 3-glucuronide	[M-H]-	461.04681	C21H18O12	Flavonoid-3-O-glucuronides	FNTJVYCFNVUBOL-ZUGPOPFOSA-N	O=C(O)C1OC(OC=2C(=O)C=3C(O)=CC(O)=CC3OC2C=4C=CC(O)=CC4)C(O)C(O)C1O
697	1.291	297.08127	7,4'-Dimethoxy-3-hydroxyflavone	[M-H]-	297.07684	C17H14O5	Flavonols	IFHXSMSQHPVVSF-UHFFFAOYSA-N	COC1=CC=C(C=C1)C1=C(O)C(=O)C2=CC=C(OC)C=C2O1
698	1.291	301.1058	tricetin	[M-H]-	301.1048	C15H10O7	Flavones	ARSRJFRKVXALTF-UHFFFAOYSA-N	O=C1C=C(OC=2C=C(O)C=C(O)C12)C=3C=C(O)C(O)=C(O)C3
699	1.293	279.15839	Dibutyl phthalate	[M+H]+	279.15909	C16H22O4	Benzoic acid esters	DOIRQSBPFJWKBE-UHFFFAOYSA-N	CCCCOC(C1=CC=CC=C1C(=O)OCCCC)=O
700	1.31	377.19247	(E)-3-(4-acetyloxy-2,3-dihydroxy-2,5,5,8a-tetramethyl-3,4,4a,6,7,8-hexahydro-1H-naphthalen-1-yl)prop-2-enoic acid	[M+H]+	377.19345	C19H30O6	Dicarboxylic acids and derivatives	OZESVNMBZAEFCX-BQYQJAHWSA-N	O=C(O)C=CC1C(O)(C)C(O)C(OC(=O)C)C2C(C)(C)CCCC12C
701	1.31	318.11993	Tsitsikammamine B	[M+H]+	318.12	[C19H16N3O2]+	Pyrrolo[4,3,2-de]quinolines	AHSWTDRJRRNLLR-UHFFFAOYSA-O	O=C1C=2NC=C(C=3C=CC(O)=CC3)C2C4=[NH+]CCC5=CN(C1=C54)C
702	1.31	217.15541	Valylvaline	[M+H]+	217.1554	C10H20N2O3	Dipeptides	KRNYOVHEKOBTEF-UHFFFAOYNA-N	O=C(O)C(NC(=O)C(N)C(C)C)C(C)C
703	1.315	384.18521	Dihydrozeatin-9-beta-D-glucoside	[M+H]+	384.18777	C16H25N5O6	Glycosylamines	DRPMMLWYLAPTPK-UFZVAZPKSA-N	CC(CO)CCNC1=C2N=CN([C=H]3O[C=H](CO)[C=H](O)[C=H](O)[C=H]3O)C2=NC=N1
704	1.317	147.02901	Citramalic acid	[M-H]-	147.02989	C5H8O5	Hydroxy fatty acids	XFTRTWQBIOMVPK-RXMQYKEDSA-N	C[C=](O)(CC(O)=O)C(O)=O
705	1.318	374.15747	8-methoxy-7-[(E)-3-methyl-4-(4-methyl-5-oxo-2H-furan-2-yl)but-2-enoxy]chromen-2-one	[M+H]+	374.15979	C20H20O6	Coumarins and derivatives	QUKDLMFYXITHBM-XYOKQWHBSA-N	O=C1OC=2C(OC)=C(OCC=C(C)CC3OC(=O)C(=C3)C)C=CC2C=C1
706	1.321	391.28287	Bis(2-ethylhexyl) phthalate	[M+H]+	391.284	C24H38O4	Benzoic acid esters	BJQHLKABXJIVAM-UHFFFAOYSA-N	O=C(OCC(CC)CCCC)C=1C=CC=CC1C(=O)OCC(CC)CCCC
707	1.325	117.01865	SUCCINIC ACID	[M-H]-	117.01933	C4H6O4	Dicarboxylic acids and derivatives	KDYFGRWQOYBRFD-UHFFFAOYSA-N	OC(=O)CCC(O)=O
708	1.325	421.07367	Trehalose-6-Phosphate	[M-H]-	421.07526	C12H23O14P	Disaccharide phosphates	LABSPYBHMPDTEL-UHFFFAOYNA-N	O=P(O)(O)OCC1OC(OC2OC(CO)C(O)C(O)C2O)C(O)C(O)C1O
709	1.326	356.18954	N-isopentyl-2-(3,4,9-trimethyl-7-oxo-7H-furo[2,3-f]chromen-8-yl)acetamide	[M+H]+	356.19	C21H25NO4	Angular furanocoumarins	NXIXJPLNNMLKRR-UHFFFAOYSA-N	O=C1OC2=CC(=C3C(OC=C3C)=C2C(=C1CC(=O)NCCC(C)C)C)C
710	1.327	157.05011	2-(hydroxymethyl)-5-methoxy-4H-pyran-4-one	[M+H]+	157.05	C7H8O4	Pyranones and derivatives	RLWWKLWEBQOOAB-UHFFFAOYSA-N	O=C1C=C(OC=C1OC)CO
711	1.327	275.16135	3,8-dihydroxy-3,8-dimethyl-5-propan-2-ylidene-1,2,3a,4,7,8a-hexahydroazulen-6-one	[M+H]+	275.16174	C15H24O3	Guaianes	TXIKNNOOLCGADE-UHFFFAOYSA-N	O=C1C(=C(C)C)CC2C(CCC2(O)C)C(O)(C)C1
712	1.327	347.19406	Catechin Tetramethylether	[M+H]+	347.19571	C19H22O6	Catechins	GKPNPQODFXMCGO-IFXJQAMLSA-N	OC1CC=2C(OC)=CC(OC)=CC2OC1C3=CC=C(OC)C(OC)=C3
713	1.328	433.10739	Flavanone base + 3O, O-Hex	[M-H]-	433.10739	C21H22O10	Flavanone O-glycosides	DLIKSSGEMUFQOK-UHFFFAOYNA-N	O=C3C4=C(O)C=C(OC1OC(CO)C(O)C(O)C1(O))C=C4(OC(C2=CC=C(O)C=C2)C3)
714	1.328	175.06015	Isopropylmalic acid	[M-H]-	175.05991	C7H12O5	Organic acids	BITYXLXUCSKTJS-UHFFFAOYNA-N	O=C(O)CC(O)(C(=O)O)C(C)C
715	1.333	367.17056	Dehydrocorydalin	[M+H]+	367.17001	C22H24NO4+	Protoberberine alkaloids and derivatives	RFKQJTRWODZPHF-UHFFFAOYSA-N	O(C=1C=CC=2C(=C[N+]3=C(C4=CC(OC)=C(OC)C=C4CC3)C2C)C1OC)C
716	1.335	384.2215	18-Aminoabieta-8,11,13-Triene Sulfate	[M+H]+	384.22031	C20H33NO4S	Diterpenoids	FDYFHDKFDSGDKM-UHFFFAOYSA-N	O=S(=O)(O)O.NCC1(C)CCCC2(C3=CC=C(C=C3CCC21)C(C)C)C
717	1.342	550.17108	Bosentan	[M-H]-	550.17657	C27H29N5O6S	Bipyrimidines and oligopyrimidines	GJPICJJJRGTNOD-UHFFFAOYSA-N	CC(C)(C)C1=CC=C(C=C1)S(NC2=C(C(=NC(C3=NC=CC=N3)=N2)OCCO)OC4=CC=CC=C4OC)(=O)=O
718	1.343	353.17148	(4,7,7-trimethyl-3-bicyclo[2.2.1]heptanyl) (E)-3-(4-hydroxy-3-methoxyphenyl)prop-2-enoate	[M+H]+	353.17233	C20H26O4	Coumaric acids and derivatives	PKAIECBWQZFYRP-SOFGYWHQSA-N	O=C(OC1CC2CCC1(C)C2(C)C)C=CC3=CC=C(O)C(OC)=C3
719	1.343	149.06042	3,4-Dihydrocoumarin	[M+H]+	149.05971	C9H8O2	3,4-dihydrocoumarins	VMUXSMXIQBNMGZ-UHFFFAOYSA-N	O=C1CCC2=CC=CC=C2O1
720	1.343	306.09854	maesopsin	[M+H]+	306.0972	C15H12O6	Auronols	LOFYFDPXORJJEE-UHFFFAOYSA-N	O=C1C=2C(O)=CC(O)=CC2OC1(O)CC3=CC=C(O)C=C3
721	1.343	243.13449	PyroglutamylIsoleucine (isomer of 1005)	[M+H]+	243.1344	C11H18N2O4	Dipeptides	MKCVLTRITQNGQI-UHFFFAOYNA-N	O=C(O)C(NC(=O)C1NC(=O)CC1)C(C)CC
722	1.344	297.04752	KUHLMANNIN	[M-H]-	297.04343	C17H14O5	Neoflavones	ABUBCBFUQXIEAU-UHFFFAOYSA-N	O=C1OC=2C(OC)=C(OC)C(O)=CC2C(=C1)C=3C=CC=CC3
723	1.346	86.09614	2-Methylpyrrolidine	[M+H]+	86.09643	C5H11N	Pyrrolidines	RGHPCLZJAFCTIK-UHFFFAOYSA-N	CC1CCCN1
724	1.347	154.01508	Citrazinc acid	[M-H]-	154.0146	C6H5NO4	Pyridinecarboxylic acids	CSGQJHQYWJLPKY-UHFFFAOYSA-N	O=C(O)C=1C=C(O)N=C(O)C1
725	1.348	410.25287	N-(2-(((1S,9aR)-octahydro-1H-quinolizin-1-yl)methyl)-3-oxo-1,2,3,4-tetrahydrobenzo[4,5]imidazo[1,2-a]pyrazin-8-yl)propionamide	[M+H]+	410.254	C23H31N5O2	Quinolizines	NVXMWPFVIMTPPT-QFBILLFUSA-N	O=C(NC=1C=CC2=C(N=C3N2CC(=O)N(C3)CC4CCCN5CCCCC54)C1)CC
726	1.354	352.12247	3-methyl-8,10,20,22-tetraoxa-3-azapentacyclo[15.7.0.0?,??.0?,??.0??,??]tetracosa-1(17),5,7(11),12,18,23-	[M-H]-	352.11905	C20H19NO5	Benzodioxoles	HXCUIEYBVCYJPX-UHFFFAOYSA-N	O=C1C2=CC=3OCOC3C=C2CN(C)CC4=CC=5OCOC5C=C4CC1
727	1.356	403.10989	3-(3,4-dihydro-2H-benzo[b][1,4]dioxepin-7-yl)-2,8-dimethyl-4-oxo-4H-chromen-7-yl acetate	[M+H]+	403.10999	C22H20O6	Isoflavones	CQJGDYZEMIGJCJ-UHFFFAOYSA-N	O=C(OC1=CC=C2C(=O)C(C=3C=CC=4OCCCOC4C3)=C(OC2=C1C)C)C
728	1.356	132.10141	D-Alloisoleucine	[M+H]+	132.1019	C6H13NO2	Isoleucine and derivatives	AGPKZVBTJJNPAG-CRCLSJGQSA-N	CC[C=H](C)[C=H](N)C(O)=O
729	1.361	212.10281	2,3-Dideoxycytidine	[M+H]+	212.10297	C9H13N3O3	Pyrimidine 2',3'-dideoxyribonucleosides	WREGKURFCTUGRC-UHFFFAOYNA-N	N=C1N=C(O)N(C=C1)C2OC(CO)CC2

730	1.361	425.1936	2-[5-[2-[2-[5-(2-hydroxypropyl)oxolan-2-yl]propanoyloxy]propyl]oxolan-2-yl]propanoic acid	[M+H]+	425.1936	C20H34O7	Dicarboxylic acids and derivatives	VIHRKOVEWCGJQP-UHFFFAOYSA-N	O=C(O)C(C)C1OC(CC1)CC(OC(=O)C(C)C2OC(CC2)CC(O)C)C
731	1.361	415.17322	6-formyl-10-(hydroxymethyl)-5-methoxy-3-methylidene-2-oxo-2H,3H,3aH,4H,5H,8H,9H,11aH-cyclodeca[b]furan-4-yl 2-methylbutanoate	[M+H]+	415.1727	C21H28O7	Germacranolides and derivatives	LOFBWESEBFUNJU-FRYCFAQMSA-N	O=CC1=CCCC(=CC2OC(=O)C(=C)C2C(OC(=O)C(C)CC)C1OC)CO
732	1.361	204.12425	Isopentenyladenine	[M+H]+	204.12437	C10H13N5	6-alkylaminopurines	HYVABZIGRDEKCD-UHFFFAOYSA-N	N=1C=NC=2C1NC=NC2NCC=C(C)C
733	1.363	331.06491	Gallic acid hexoside	[M-H]-	331.06491	C13H16O10	Phenolic glycosides	GDVRUDXLQBVIKP-UHFFFAOYNA-N	O=C(OC1OC(CO)C(O)C(O)C1(O))C2=CC(O)=C(O)C(O)=C2
734	1.363	261.1459	Glutamylleucine	[M+H]+	261.1459	C11H20N2O5	Dipeptides	MYFMARDICOWMQP-UHFFFAOYNA-N	O=C(O)C(N)CCC(=O)NC(C(=O)O)CC(C)C
735	1.364	282.14529	Pendimethalin	[M+H]+	282.14484	C13H19N3O4	Dinitroanilines	CHIFOSRWCNZCFN-UHFFFAOYSA-N	CCC(CC)NC1=C(C=C(C)C(C)=C1N(=O)=O)N(=O)=O
736	1.369	247.0385	Fludioxonil	[M-H]-	247.03246	C12H6F2N2O2	Benzodioxoles	MUJOIMFVNIBMKC-UHFFFAOYSA-N	FC1(F)OC2=CC=CC(=C2O1)C1=CNC=C1C#N
737	1.371	296.1579	Nuciferine	[M+H]+	296.16	C19H21NO2	Aporphines	ORJVQPIHKOARKV-OAHLLOKOSA-N	O(C=1C=C2C3=C(C1OC)C4=CC=CC=C4CC3N(C)CC2)C
738	1.375	423.19608	5-[(Z)-12-(3,5-dihydroxyphenyl)dodec-8-enyl]benzene-1,3-diol	[M+H]+	423.19321	C24H32O4	Resorcinols	JDIDEXHTJXTVES-HYXAFXHYSA-N	OC=1C=C(O)C=C(C1)CCCC=CCCCCCCCC=2C=C(O)C=C(O)C2
739	1.375	366.07083	GALANTHAMINE HYDROBROMIDE	[M-H]-	366.07101	C17H22BrNO3	Galanthamine-type amaryllidaceae alkaloids	QORVDGQLPPAFRS-UHFFFAOYSA-N	Br.OC1C=CC23C4=C(OC2C1)C(OC)=CC=C4CN(C)CC3
740	1.378	439.08249	(Z)-methyl 4-((9-(furan-2-yl)-3,7-dioxo-3,7,8,9-tetrahydro-2H-furo[2,3-f]chromen-2-ylidene)methyl)benzoate	[M+H]+	439.07999	C24H16O7	Aurone flavonoids	KUNIPLHOIJFHHR-ODLFYWEKSA-N	O=C(OC)C1=CC=C(C=C1)C=C2OC=3C(=CC=C4OC(=O)CC(C=5OC=CC5)C43)C2=O
741	1.378	235.14507	Coumaroyl putrescin	[M+H]+	235.1447	C13H18N2O2	Coumaric acid and derivatives	CJHDBEPXEKGBDW-UHFFFAOYSA-N	O=C(C=CC1=CC=C(O)C=C1)NCCCCN
742	1.378	343.19937	Pesticide3_Iprovalicarb Isomer 1*_C18H28N2O3_Isopropyl [(2S)-3-methyl-1-{[1-(4-methylphenyl)ethyl]amino}-1-oxo-2-butanyl]carbamate	[M+H]+	343.19901	C18H28N2O3	Toluenes	NWUWYYSKZYIQAE-WMCAAGNKSA-N	OC(=NC(C(O)=NC(C1=CC=C(C=C1)C)C)C(C)C)OC(C)C
743	1.382	102.02722	Biuret	[M-H]-	102.0309	C2H5N3O2	Isoureas	OHJMTUPIZMNBFR-UHFFFAOYSA-N	N=C(O)NC(=N)O
744	1.388	131.07187	2-Hydroxy-4-methylpentanoic acid	[M-H]-	131.07137	C6H12O3	Hydroxy fatty acids	LVRFTAZAXQPQHI-UHFFFAOYNA-N	O=C(O)C(O)CC(C)C
745	1.388	330.11115	Nizatidine	[M-H]-	330.10638	C12H21N5O2S2	2,4-disubstituted thiazoles	SGXXNSQHWDMGGP-UHFFFAOYSA-N	O=N(=O)C=C(NC)NCCSCC=1N=C(SC1)CN(C)C
746	1.393	154.0139	4-Nitrocatechol	[M-H]-	154.01459	C6H5NO4	Nitrophenols	XJNPNXSISMKQEX-UHFFFAOYSA-N	O=N(=O)C1=CC=C(O)C(O)=C1
747	1.393	182.04498	4-Pyridoxic acid	[M-H]-	182.04588	C8H9NO4	Pyridinecarboxylic acids	HXACOUQIXZGNBF-UHFFFAOYSA-N	O=C(O)C1=C(O)C(=NC=C1CO)C
748	1.396	387.14172	[(3aR,4S,6R,11aR)-6,9-dihydroxy-6,10-dimethyl-3-methylidene-2,7-dioxo-3a,4,5,8,9,11a-hexahydrocyclodeca[b]furan-4-yl] 2-methylprop-2-enoate	[M+H]+	387.14142	C19H24O7	Germacranolides and derivatives	JGWDBQRLOBLTHU-XHOJKXOPSA-N	O=C(OC1CC(O)(C(=O)CC(O)C(=CC2OC(=O)C(=C)C21)C)C)C(=C)C
749	1.396	261.0957	3-hydroxyflavone	[M+H]+	261.09534	C15H10O3	Flavonols	HVQAJTFOCKOKIN-UHFFFAOYSA-N	O=C1C(O)=C(OC=2C=CC=CC21)C=3C=CC=CC3
750	1.396	438.24002	Dicoumaroyl Spermidine	[M+H]+	438.23981	C25H31N3O4	Coumaric acid and derivatives	QYBCBMVQSCJMSA-UHFFFAOYSA-N	O=C(C=CC1=CC=C(O)C=C1)NCCCNCCCCNC(=O)C=CC2=CC=C(O)C=C2
751	1.396	322.15076	Famciclovir	[M+H]+	322.15097	C14H19N5O4	Purines and purine derivatives	GGXKWVWZWMLJEH-UHFFFAOYSA-N	O=C(OCC(COC(=O)C)CCN1C=NC2=CNC(=N)N=C21)C
752	1.396	308.17105	Zolpidem	[M+H]+	308.17575	C19H21N3O	Phenylimidazoles	ZAFYATHCZYHLPB-UHFFFAOYSA-N	O=C(N(C)C)CC1=C(N=C2C=CC(=CN21)C)C3=CC=C(C=C3)C
753	1.4	218.13818	Propionylcarnitine	[M+H]+	218.13869	C10H19NO4	Acyl carnitines	UFAHZIUFPNSHSL-UHFFFAOYNA-N	O=C([O-])CC(OC(=O)CC)C[N+](C)(C)C
754	1.413	195.11346	Hydroxypyridin + C5H10NO or 6-Hydroxypseudooxynicotine (not validated)	[M+H]+	195.11369	C10H14N2O2	Nicotine and derivatives	UMLOUOBDBGOHHR-UHFFFAOYSA-N	O=C(C=1C=NC(O)=CC=1)CCCNC
755	1.422	392.11758	S-(2-Carboxypropyl)glutathione	[M-H]-	392.11249	C14H23N3O8S	Peptides	JQWABENXVMJJMW-UHFFFAOYNA-N	O=C(O)CNC(=O)C(NC(=O)CCC(N)C(=O)O)CSCC(C(=O)O)C
756	1.428	254.11336	Triamterene	[M+H]+	254.11501	C12H11N7	Pteridines and derivatives	FNYLWPVRPXGIIP-UHFFFAOYSA-N	N1=C(N=C(N)C2=NC(=C(N=C12)N)C3=CC=CC=C3)N
757	1.432	146.11812	(3R,4S)-3-Amino-4-methylhexanoic acid	[M+H]+	146.11755	C7H15NO2	Beta amino acids and derivatives	JHEDYGILOIBOTL-NTSWFWBYSA-N	CC[C=H](C)[C=H](N)CC(O)=O
758	1.432	362.11255	Bezafibrate	[M+H]+	362.11536	C19H20ClNO4	Phenoxyacetic acid derivatives	IIBYAHWJQTYFKB-UHFFFAOYSA-N	CC(C)(C(=O)O)OC1=CC=C(C=C1)CCNC(C2=CC=C(C=C2)Cl)=O
759	1.436	371.08096	Rebamipide	[M+H]+	371.07901	C19H15ClN2O4	Hippuric acids	ALLWOAVDORUJLA-UHFFFAOYSA-N	O=C1C=C(C=2C=CC=CC2N1)CC(NC(=O)C3=CC=C(Cl)C=C3)C(=O)O
760	1.439	203.13892	Ala-Ile	[M+H]+	203.13902	C9H18N2O3	Dipeptides	ZSOICJZJSRWNHX-UHFFFAOYNA-N	O=C(O)C(N=C(O)C(N)C)C(C)CC
761	1.442	341.10834	Trehalose	[M-H]-	341.10895	C12H22O11	O-glycosyl compounds	HDTRYLNUVZCQOY-UHFFFAOYNA-N	OCC1OC(OC2OC(CO)C(O)C(O)C2O)C(O)C(O)C1O
762	1.444	542.1701	PUROMYCIN HYDROCHLORIDE	[M-H]-	542.16907	C22H31Cl2N7O5	Purine 3'-deoxyribonucleosides	MKSVFGKWZLUTTO-FZFAUISWSA-N	Cl.Cl.OC(=NC1C(O)C(OC1CO)N2C=NC=3C2=NC=NC3N(C)C)C(N)CC4=CC=C(OC)C=C4
763	1.449	105.07031	"L-2,3-DIAMINOPROPIONIC ACID"	[M+H]+	105.07	C3H8N2O2	Alpha amino acids	PECYZEOJVXMISF-UHFFFAOYSA-N	O=C(O)C(N)CN
764	1.462	258.06073	2-Thiocytidine	[M-H]-	258.05539	C9H13N3O4S	Pyrimidine nucleosides	RHFUOMFWUGWKKO-UHFFFAOYNA-N	N=C1N=C(S)N(C=C1)C2OC(CO)C(O)C2O
765	1.464	263.052	Sorbitol-6-phosphate	[M+H]+	263.05264	C6H15O9P	Monosaccharide phosphates	GACTWZZMVMUKNG-UHFFFAOYNA-N	O=P(O)(O)OCC(O)C(O)C(O)C(O)CO
766	1.468	479.1684	2-[3,4-dihydroxy-2,2-dimethyl-8-(3-methylbut-2-enoxy)-3,4-dihydrochromen-6-yl]-5,7-dihydroxy-2,3-dihydrochromen-4-one	[M+H]+	479.16763	C25H28O8	3'-prenylated flavanones	LNFRDRVVARCALE-UHFFFAOYSA-N	O=C1C=2C(O)=CC(O)=CC2OC(C3=CC(OCC=C(C)C)=C4OC(C)(C)C(O)C(O)C4=C3)C1
767	1.47	282.11804	2'-O-Methyladenosine	[M+H]+	282.11969	C11H15N5O4	Purine nucleosides	FPUGCISOLXNPPC-UHFFFAOYNA-N	OCC1OC(N2C=NC=3C(=NC=NC32)N)C(OC)C1O
768	1.475	144.06543	4-Hydroxy-6-Methylpyran-2-One	[M+H]+	144.06552	C6H6O3	Pyranones and derivatives	NSYSSMYQPLSPOD-UHFFFAOYSA-N	O=C1OC(=CC(O)=C1)C
769	1.476	432.10999	HDMBOA + O-Hex	[M-H]-	432.11371	C16H21NO10	Benzoxazinoids	UOASSFRPBORTCT-UHFFFAOYNA-N	O=C2N(OC)C=3C=CC(OC)=CC=3(OC2(OC1OC(CO)C(O)C(O)C1(O)))
770	1.481	593.15564	kaempferol 7-neohesperidoside	[M-H]-	593.1579	C27H30O15	Flavonoid-7-O-glycosides	ZEJXENDZTYVXDP-CSJHBIPPSA-N	O=C1C(O)=C(OC2=CC(OC3OC(CO)C(O)C(O)C3OC4OC(C)C(O)C(O)C4O)=CC(O)=C12)C=5C=CC(O)=CC5
771	1.483	361.15149	Perivine	[M+H]+	361.14999	C20H22N2O3	Vobasan alkaloids	NKTORRNHKYVXSU-XXMLWKDOSA-N	O=C(OC)C1C2NCC(=CC)C1CC(=O)C=3NC=4C=CC=CC4C3C2
772	1.484	269.02911	3-deoxysappanchalcone	[M-H]-	269.02448	C16H14O4	Cinnamylphenols	PACBGANPVNHGNP-RUDMXATFSA-N	O=C(C=CC1=CC=C(O)C=C1)C2=CC=C(O)C=C2OC

773	1.485	711.21649	Tetrasaccharides (Hex-Hex-Hex-Hex)	[M-H]-	711.21698	C24H42O21	Sugars	UQZIYBXSHAGNOE-UHFFFAOYNA-N	OCC4OC(OCC3OC(OCC2OC(OC1(OC(CO)C(O)C1(O))(CO))C(O)C(O)C2(O))C(O)C(O)C3(O))C(O)C(O)C4(O)
774	1.486	493.21225	plumieride	[M+H]+	493.21024	C21H26O12	Iridoid O-glycosides	AOPMSFXOYJXDNJ-IRFSQMTFSA-N	O=C(OC)C1=COC(OC2OC(CO)C(O)C(O)C2O)C3C1C=CC43OC(=O)C(=C4)C(O)C
775	1.494	131.04543	Asparagine	[M-H]-	131.04622	C4H8N2O3	Asparagine and derivatives	DCXYFEDJOCDNAF-UHFFFAOYNA-N	O=C(O)C(N)CC(=N)O
776	1.499	419.10541	methyl 2-((3-(benzo[d][1,3]dioxol-5-yl)-4-oxo-6-propyl-4H-chromen-7-yl)oxy)acetate	[M+H]+	419.10999	C22H20O7	Isoflavones	NNOQXTZBOCAPIN-UHFFFAOYSA-N	O=C(OC)COC1=CC=2OC=C(C(=O)C2C=C1CCC)C=3C=CC=4OCOC4C3
777	1.505	205.03474	Lipoic acid	[M-H]-	205.03624	C8H14O2S2	Lipoic acids and derivatives	AGBQKNBQESQNJD-UHFFFAOYNA-N	O=C(O)CCCCC1SSCC1
778	1.511	325.11151	Prazepam	[M+H]+	325.11023	C19H17ClN2O	1,4-benzodiazepines	MWQCHHACWWAQLJ-UHFFFAOYSA-N	O=C1N(C=2C=CC(Cl)=CC2C(=NC1)C=3C=CC=CC3)CC4CC4
779	1.514	503.16153	Melezitose	[M-H]-	503.16174	C18H32O16	Oligosaccharides	QWIZNVHXZXRPDR-WSCXOGSTSA-N	OCC1OC(OC2C(O)C(OC2(OC3OC(CO)C(O)C(O)C3O)CO)CO)C(O)C(O)C1O
780	1.52	371.10016	Camptothecin	[M+H]+	371.10019	C20H16N2O4	Camptothecins	VSJKWCGYPAHWDS-FQEVSTJZSA-N	O=C1OCC=2C(=O)N3C(=CC2C1(O)CC)C=4N=C5C=CC=CC5=CC4C3
781	1.521	144.06584	4-ACETAMIDOBUTANOATE	[M-H]-	144.06599	C6H11NO3	Gamma amino acids and derivatives	UZTFMUBKZQVKLK-UHFFFAOYSA-N	O=C(O)CCCNC(=O)C
782	1.531	328.04486	Aristolochic acid C	[M+H]+	328.04517	C16H9NO7	Others	NBFGYDJKTHENDP-UHFFFAOYSA-N	OC(=O)C1=CC2=C(OCO2)C2=C1C(=CC1=CC=C(O)C=C21)[N+]([O-])=O
783	1.534	103.04038	DL-beta-Hydroxybutyric acid	[M-H]-	103.04007	C4H8O3	Beta hydroxy acids and derivatives	WHBMMWSBFZVSSR-UHFFFAOYNA-N	CC(O)CC(O)=O
784	1.54	330.13712	Metolachlor ESA	[M+H]+	330.13696	C15H23NO5S	Anilides	CIGKZVUEZXGYSV-UHFFFAOYNA-N	CCC1=CC=CC(C)=C1N(C(C)COC)C(CS(O)(=O)=O)=O
785	1.542	121.03838	NIACINAMIDE	[M-H]-	121.04073	C6H6N2O	Nicotinamides	DFPAKSUCGFBDDF-UHFFFAOYSA-N	N=C(O)C=1C=NC=CC1
786	1.552	405.10547	Shanzhiside methyl ester	[M-H]-	405.10574	C17H26O11	Iridoid O-glycosides	KKSYAZCUYVRKML-IRDZEPHTSA-N	O=C(OC)C1=COC(OC2OC(CO)C(O)C(O)C2O)C3C1C(O)CC3(O)C
787	1.563	179.12727	Triglyme	[M+H]+	179.12779	C8H18O4	Dialkyl ethers	YFNKIDBQEZZDLK-UHFFFAOYSA-N	COCCOCCOCCOC
788	1.574	111.04449	SORBATE	[M-H]-	111.045	C6H8O2	Medium-chain fatty acids	WSWCOQWTEOXDQX-MQQKCMAXSA-N	O=C(O)C=CC=CC
789	1.575	199.02409	ASCORBIC ACID	[M+H]+	199.0213	C6H8O6	Butenolides	CIWBSHSKHKDKBQ-JLAZNSOCSA-N	O=C1OC(C(O)=C1O)C(O)CO
790	1.577	319.11197	Rosin	[M+H]+	319.10999	C15H20O6	Fatty acyl glycosides of mono- and disaccharides	KHPCPRHQVVSZAH-GUNCLKARSA-N	OCC1OC(OCC=CC=2C=CC=CC2)C(O)C(O)C1O
791	1.59	446.11945	(Z)-methyl 2-(2-(3-hydroxy-4-methoxybenzylidene)-3-oxo-3,11-dihydro-2H-furo[2',3':5,6]chromeno[2,3-b]pyridin-11-yl)acetate	[M+H]+	446.12	C25H19NO7	Aurone flavonoids	INIACRGCTLLWRM-JAIQZWGSSA-N	O=C(OC)CC1C2=CC=CN=C2OC3=CC=C4C(=O)C(OC4=C31)=CC5=CC=C(OC)C(O)=C5
792	1.594	339.11703	Loureirin B	[M+H]+	339.12	C18H20O5	Retro-dihydrochalcones	ZPFRAPVRYLGYEC-UHFFFAOYSA-N	O=C(C1=CC=C(O)C=C1)CCC=2C(OC)=CC(OC)=CC2OC
793	1.608	144.06729	4-Acetamidobutyric acid	[M-H]-	144.06662	C6H11NO3	Gamma amino acids and derivatives	UZTFMUBKZQVKLK-UHFFFAOYSA-N	O=C(O)CCCN=C(O)C
794	1.614	388.17078	(2S,3aR,5aR,6aR,7S,8R,9R,10S,10bR)-3a-methyl-12-methylenedodecahydro-1H-5,7,10b-(epimethanetriyl)-6a,9-ethanodibenzo[cd,f]indole-2,8,10-triol	[M+H]+	388.17001	C20H28ClNO3	Atisane diterpenoids	FYJNFPYTJQUYMD-SNDJKJIZSA-N	Cl.OC1CC2(C)CN3C4CC56CC(=C)C7C(O)C6C3C(C1)(C5C7O)C42
795	1.617	387.36145	5Alpha-Cholestanol	[M-H]-	387.36322	C27H48O	Cholesterols and derivatives	QYIXCDOBOSTCEI-QCYZZNICSA-N	OC1CCC2(C)C(CCC3C4CCC(C(C)CCCC(C)C)C4(C)CCC32)C1
796	1.62	455.1012	Deoxyuridine	[M-H]-	455.10092	C9H12N2O5	Pyrimidine 2'-deoxyribonucleosides	MXHRCPNRJAMMIM-SHYZEUOFSA-N	O=C1N=C(O)C=CN1C2OC(CO)C(O)C2
797	1.629	211.07317	2,3-Dideoxyuridine	[M-H]-	211.07243	C9H12N2O4	Pyrimidine 2',3'-dideoxyribonucleosides	BTOTXLJHDSNXMW-UHFFFAOYNA-N	O=C1N=C(O)C=CN1C2OC(CO)CC2
798	1.629	232.15387	Butyryl carnitine (isomer of 919)	[M+H]+	232.1539	C11H21NO4	Acyl carnitines	QWYFHHGCZUCMBN-UHFFFAOYNA-N	O=C([O-])CC(OC(=O)CCC)C[N+](C)(C)C
799	1.634	362.14963	Indole-3-acetyl-L-tryptophan	[M+H]+	362.14993	C21H19N3O3	Amino acids	FOSPCYZZRVNHJS-UHFFFAOYNA-N	OC(CC1=CNC2=CC=CC=C12)=NC(CC1=CNC2=CC=CC=C12)C(O)=O
800	1.637	433.11819	Naringenin-7-O-glucoside	[M-H]-	433.11401	C21H22O10	Flavanone O-glycosides	DLIKSSGEMUFQOK-UHFFFAOYNA-N	O=C3C4=C(O)C=C(OC1OC(CO)C(O)C(O)C1(O))C=C4(OC(C2=CC=C(O)C=C2)C3)
801	1.641	159.08394	(S)-1-carbamoylpyrrolidine-2-carboxylic acid	[M+H]+	159.08	C6H10N2O3	Proline and derivatives	RTNFBJXLYMERBB-BYPYZUCNSA-N	O=C(N)N1CCCC1C(=O)O
802	1.642	407.10977	[(2R,3S,4R)-5-(2,4-dioxopyrimidin-1-yl)-4-hydroxy-2-(hydroxymethyl)oxolan-3-yl] 4-hydroxy-2-methoxy-6-methylbenzoate	[M-H]-	407.10959	C18H20N2O9	Pyrimidine nucleosides	LOUBNBRAJPSMEC-GYBIIRLBSA-N	O=C(OC1C(O)C(OC1CO)N2C=CC(O)=NC2=O)C=3C(OC)=CC(O)=CC3C
803	1.657	197.00208	Aconitic Acid	[M+H]+	197.00566	C6H6O6	Tricarboxylic acids and derivatives	GTZCVFVGUGFEME-IWQZZHSRSA-N	O=C(O)C=C(C(=O)O)CC(=O)O
804	1.658	365.18997	Oridonin	[M+H]+	365.19	C20H28O6	Kaurane diterpenoids	SDHTXBWLVGWJFT-RDQYUEOQSA-N	O=C1C(=C)C2CCC3C1(C2O)C4(O)OCC53C(O)CCC(C)(C)C5C4O
805	1.661	325.10602	7-[(E)-3-methyl-4-(4-methyl-5-oxo-2H-furan-2-yl)but-2-enoxy]chromen-2-one	[M-H]-	325.10815	C19H18O5	Coumarins and derivatives	CFNMUZCFSDMZPQ-KPKJPENVSA-N	O=C1OC=2C=C(OCC=C(C)CC3OC(=O)C(=C3)C)C=CC2C=C1
806	1.662	285.15472	(2R,3R,4S,5S,6R)-2-[(Z)-hex-3-enoxy]-6-(hydroxymethyl)oxane-3,4,5-triol	[M+H]+	285.155	C12H22O6	Fatty acyl glycosides of mono- and disaccharides	OZIPFYKAIOOVEJ-ODWUMMNUSA-N	OCC1OC(OCCC=CCC)C(O)C(O)C1O
807	1.668	226.11771	Kynurenine	[M+H]+	226.11861	C10H12N2O3	Alkyl-phenylketones	YGPSJZOEDVAXAB-UHFFFAOYSA-N	O=C(O)C(N)CC(=O)C=1C=CC=CC1N
808	1.673	173.04468	(-)-Shikimic acid	[M-H]-	173.04555	C7H10O5	Shikimic acids and derivatves	JXOHGGNKMLTUBP-HSUXUTPPSA-N	O[C=H]1CC(=C[C=H](O)[C=H]1O)C(O)=O
809	1.679	517.17938	[(2S,3S,4R,5R)-4-hydroxy-2,5-bis(hydroxymethyl)-2-[(2R,3R,4S,5S,6R)-3,4,5-trihydroxy-6-(hydroxymethyl)oxan-2-yl]oxyoxolan-3-yl] (E)-3-(4-hydroxy-3-	[M-H]-	517.17932	C22H30O14	Coumaric acids and derivatives	ZPEADZHVGOCGKH-YQTDNFGYSA-N	O=C(OC1C(O)C(OC1(OC2OC(CO)C(O)C(O)C2O)CO)CO)C=CC3=CC=C(O)C(OC)=C3
810	1.682	191.09154	Scopoletin	[M-H]-	191.09163	C10H8O4	7-hydroxycoumarins	RODXRVNMMDRFIK-UHFFFAOYSA-N	O=C1OC=2C=C(O)C(OC)=CC2C=C1
811	1.687	175.06206	2-Isopropylmalic acid	[M-H]-	175.0612	C7H12O5	Hydroxy fatty acids	BITYXLXUCSKTJS-UHFFFAOYNA-N	O=C(O)CC(O)(C(=O)O)C(C)C
812	1.691	227.15004	NPE_227.1543_9.6	[M+H]+	227.15428	C15H18N2	Aralkylamines	GDCVFNAQLOMGMS-UHFFFAOYNA-N	CNCCC(C1=CC=CC=C1)C2=CC=CC=N2
813	1.716	491.2319	Glimepiride	[M+H]+	491.23199	C24H34N4O5S	Benzenesulfonamides	WIGIZIANZCJQQY-RUCARUNLSA-N	O=C(NC1CCC(C)CC1)NS(=O)(=O)C2=CC=C(C=C2)CCNC(=O)N3C(=O)C(=C(C)C3)CC
814	1.733	345.17596	Rhodotulic acid	[M+H]+	345.17999	C14H24N4O6	Alpha amino acids and derivatives	PUWVNTVQJFSBDH-UHFFFAOYSA-N	O=C1NC(C(=O)NC1CCCN(O)C(=O)C)CCCN(O)C(=O)C
815	1.739	377.05698	2-(3,5-dihydroxy-1,4-dioxo-1,4-dihydronaphthalen-2-yl)-3-methoxy-5-methylbenzoic acid	[M+H]+	377.06317	C19H14O7	Phenylnaphthalenes	PBKZJIMGHNPKBJ-UHFFFAOYSA-N	O=C(O)C=1C=C(C=C(OC)C1C=2C(=O)C=3C=CC=C(O)C3C(=O)C2O)C

816	1.74	350.0643	[3,4,5-trihydroxy-6-(hydroxymethyl)oxan-2-yl] 9-(hydroxymethyl)-2,2,6a,6b,9,12a-hexamethyl-10-[3,4,5-trihydroxy-6-[(3,4,5-trihydroxy-6-	[M+H]+	350.06366	C14H17NO7	Phenolic glycosides	HVSMXONXCJBJIF-RKQHYHRCSA-N	N#CCC1=CC=C(O)C=C1OC2OC(CO)C(O)C(O)C2O
817	1.742	359.1438	Lychnopholide	[M+H]+	359.14401	C20H22O6	Terpene lactones	QATUWZPYBIHFFR-UVXIKMMUSA-N	O=C(OC1CC2(OC(=CC2=O)C(=CC3OC(=O)C(=C)C31)C)C)C(=CC)C
818	1.746	304.07684	Fenoldopam	[M-H]-	304.07458	C16H16ClNO3	Benzazepines	TVURRHSHRRELCG-UHFFFAOYNA-N	ClC=1C(O)=C(O)C=C2C1CCNCC2C3=CC=C(O)C=C3
819	1.754	285.15445	N-acetyltryptophan	[M+H]+	285.15442	C13H14N2O3	N-acyl-alpha amino acids	DZTHIGRZJZPRDV-UHFFFAOYSA-N	O=C(O)C(N=C(O)C)CC1=CNC=2C=CC=CC21
820	1.757	524.1969	Perseitol Heptaacetate	[M+H]+	524.19739	C21H30O14	Monosaccharides	UZIIHSGPIUZAET-UHFFFAOYSA-N	O=C(OCC(OC(=O)C)C(OC(=O)C)C(OC(=O)C)C(OC(=O)C)C(OC(=O)C)COC(=O)C)C
821	1.757	386.05215	Pesticide5_Flufenacet_C14H13F4N3O2S_4'-Fluoro-N-isopropyl-2-(5-trifluoromethyl-1,3,4-thiadiazol-2-yloxy)acetanilide	[M+H]+	386.05499	C14H13F4N3O2S	Anilides	IANUJLZYFUDJIH-UHFFFAOYSA-N	O=C(N(C1=CC=C(F)C=C1)C(C)C)COC2=NN=C(S2)C(F)(F)F
822	1.765	109.06451	BENZYL ALCOHOL	[M+H]+	109.07	C7H8O	Benzyl alcohols	WVDDGKGOMKODPV-UHFFFAOYSA-N	OCC=1C=CC=CC1
823	1.769	390.17474	syringin	[M+H]+	390.17584	C17H24O9	Phenolic glycosides	QJVXKWHHAMZTBY-GCPOEHJPSA-N	OCC=CC1=CC(OC)=C(OC2OC(CO)C(O)C(O)C2O)C(OC)=C1
824	1.772	473.14886	(2R,3S,4S,5R,6R)-2-(hydroxymethyl)-6-[[(2R,3S,4S,5R,6S)-3,4,5-trihydroxy-6-(2-hydroxy-4-prop-2-enylphenoxy)oxan-2-yl]methoxy]oxane-3,4,5-triol	[M-H]-	473.14813	C21H30O12	Phenolic glycosides	JSQKCXPOJUGOCX-LWZURRPWSA-N	OC1=CC(=CC=C1OC2OC(COC3OC(CO)C(O)C(O)C3O)C(O)C(O)C2O)CC=C
825	1.78	261.03772	Glucose-1-phosphate	[M+H]+	261.03699	C6H13O9P	Monosaccharide phosphates	HXXFSFRBOHSIMQ-UHFFFAOYNA-N	O=P(O)(O)OC1OC(CO)C(O)C(O)C1O
826	1.802	313.13931	Prulaurasin	[M+H]+	313.1394	C14H17NO6	Cyanogenic glycosides	ZKSZEJFBGODIJW-MXNNCRBYSA-N	N#CC(OC1OC(CO)C(O)C(O)C1O)C=2C=CC=CC2
827	1.804	241.08217	THYMIDINE	[M-H]-	241.082	C10H14N2O5	Pyrimidine 2'-deoxyribonucleosides	IQFYYKKMVGJFEH-UHFFFAOYSA-N	O=C1NC(=O)N(C=C1C)C2OC(CO)C(O)C2
828	1.816	378.1304	3,4-dihydro-papaveraldine	[M+H]+	378.13	C20H21NO5	Benzylisoquinolines	BSYFCWAPBDZVAX-UHFFFAOYSA-N	O=C(C1=NCCC=2C=C(OC)C(OC)=CC12)C3=CC=C(OC)C(OC)=C3
829	1.816	283.01263	Clafen (Cyclophosphamide)	[M+H]+	283.01401	C7H15Cl2N2O2P	Nitrogen mustard compounds	CMSMOCZEIVJLDB-UHFFFAOYSA-N	O=P1(OCCCN1)N(CCCl)CCCl
830	1.839	195.05008	GLUCONIC ACID	[M-H]-	195.05099	C6H12O7	Medium-chain hydroxy acids and derivatives	RGHNJXZEOKUKBD-UHFFFAOYSA-N	O=C(O)C(O)C(O)C(O)C(O)CO
831	1.846	405.0477	[(6E,10Z)-10-(acetyloxymethyl)-6-formyl-3-methylidene-2-oxo-3a,4,5,8,9,11a-hexahydrocyclodeca[b]furan-4-yl] 2-methylbutanoate	[M+H]+	405.04797	C22H28O7	Germacranolides and derivatives	ALJXWCROGAMOBL-DQHHJRCBSA-N	O=CC1=CCCC(=CC2OC(=O)C(=C)C2C(OC(=O)C(C)CC)C1)COC(=O)C
832	1.851	510.20691	(3R)-N-((2S,3R)-1-((benzo[d][1,3]dioxol-5-ylmethyl)amino)-3-methyl-1-oxopentan-2-yl)-2,2-dimethyl-5-oxo-2,3,5,9b-tetrahydrothiazolo[2,3-	[M+H]+	510.20999	C27H31N3O5S	Isoindolones	YYAHDCGHROJYNX-JEQJESNBSA-N	O=C1C=2C=CC=CC2C3SC(C)(C)C(C(=O)NC(C(=O)NCC4=CC=C5OCOC5=C4)C(C)CC)N13
833	1.852	160.0249	Chloridazone-methyl-desphenyl	[M+H]+	160.02722	C5H6ClN3O	Pyridazinones	XNSGCNYTNLWRKM-UHFFFAOYSA-N	CN1N=CC(N)=C(Cl)C1=O
834	1.878	578.11377	4-(4-methoxyphenyl)-2-oxo-2H-chromen-7-yl 2-(4-methylphenylsulfonamido)-2-phenylacetate	[M+H]+	578.12	C31H25NO7S	Neoflavones	POCJVFDFEBOVMF-SSEXGKCCSA-N	O=C1OC=2C=C(OC(=O)C(NS(=O)(=O)C3=CC=C(C=C3)C)C=4C=CC=CC4)C=CC2C(=C1)C=5C=CC(OC)=CC5
835	1.883	194.03369	Phenacylamine Hydrochloride	[M+H]+	194.0343	C8H10ClNO	Alkyl-phenylketones	CVXGFPPAIUELDV-UHFFFAOYSA-N	Cl.O=C(C=1C=CC=CC1)CN
836	1.884	579.11682	Apigenin 7-??-rutinoside	[M+H]+	579.12097	C27H30O14	Flavonoid-7-O-glycosides	FKIYLTVJPDLUDL-SLNHTJRHSA-N	O=C1C=C(OC2=CC(OC3OC(COC4OC(C)C(O)C(O)C4O)C(O)C(O)C3O)=CC(O)=C12)C=5C=CC(O)=CC5
837	1.894	148.05978	Glutamic acid	[M+H]+	148.06	C5H9NO4	Glutamic acid and derivatives	WHUUTDBJXJRKMK-VKHMYHEASA-N	O=C(O)CCC(N)C(=O)O
838	1.903	503.16125	Maltotriose	[M-H]-	503.16177	C18H32O16	Oligosaccharides	FYGDTMLNYKFZSV-DZOUCCHMSA-N	OC[C=H]1O[C=H](O[C=H]2[C=H](O)[C=H](O)[C=H](O[C=H]3[C=H](O)[C=H](O)C(O)O[C=H]3CO)O[C=H]2CO)[C=H](O)[C=H](O)[
839	1.903	497.11819	Roccellin	[M-H]-	497.12399	C21H24O11	Phenolic glycosides	AFCAWRFTSGVRQA-CMWLGVBASA-N	O=C(OCC1OC(OC2=CC=3OC(=CC(=O)C3C(O)=C2C)C)C(OC(=O)C)C(O)C1O)C
840	1.908	357.10168	Corymbosin	[M-H]-	357.09799	C19H18O7	7-O-methylated flavonoids	FLCVGMVLNHYJAW-UHFFFAOYSA-N	COC1=CC(O)=C2C(=O)C=C(OC2=C1)C1=CC(OC)=C(OC)C(OC)=C1
841	1.91	671.25378	Tetrahydrogambogic Acid	[M+H]+	671.25043	C38H48O8	Pyranoxanthones	CXFIQFGADOTDPF-XKZIYDEJSA-N	O=C(O)C(=CCC12OC(C)(C)C3CC(CC4C(=O)C5=C(O)C=6C=CC(OC6C(=C5OC431)CC=C(C)C)(C)CCC=C(C)C)C2O)C
842	1.916	180.03391	Carbocysteine	[M+H]+	180.0325	C5H9NO4S	L-cysteine-S-conjugates	GBFLZEXEOZUWRN-UHFFFAOYNA-N	O=C(O)CSCC(N)C(=O)O
843	1.918	480.2067	(2S,3R,4R,5R,6S)-2-[(2R,3R,4S,5R,6R)-2-[2-(3,4-dihydroxyphenyl)ethoxy]-3,5-dihydroxy-6-(hydroxymethyl)oxan-4-yl]oxy-6-methyloxane-3,4,5-triol	[M+H]+	480.20755	C20H30O12	O-glycosyl compounds	DORPKYRPJIIARM-GYAWPQPFSA-N	OC1=CC=C(C=C1O)CCOC2OC(CO)C(O)C(OC3OC(C)C(O)C(O)C3O)C2O
844	1.919	370.08267	Lansoprazole	[M+H]+	370.08301	C16H14F3N3O2S	Sulfinylbenzimidazoles	MJIHNNLFOKEZEW-UHFFFAOYSA-N	O=S(C1=NC=2C=CC=CC2N1)CC3=NC=CC(OCC(F)(F)F)=C3C
845	1.921	327.09174	Zapotinin	[M-H]-	327.0874	C18H16O6	6-O-methylated flavonoids	BFXYIQRRGIMMSR-UHFFFAOYSA-N	COC1=CC=C2OC(=CC(=O)C2=C1O)C1=C(OC)C=CC=C1OC
846	1.925	259.11325	2-methoxy-5-methyl-3-(2-methylbut-3-en-2-yl)chromen-4-one	[M+H]+	259.11777	C16H18O3	2-methoxychromones	WAPMVTHIMHEOAB-UHFFFAOYSA-N	O=C1C=2C(OC(OC)=C1C(C=C)(C)C)=CC=CC2C
847	1.934	344.13275	N-Fructosyl tyrosine	[M+H]+	344.13321	C15H21NO8	N-Fructosyl amino acids	QLJKHQIODQKMNW-UHFFFAOYNA-N	O=C(O)C(NCC1(O)(OC(CO)C(O)C1(O)))CC2=CC=C(O)C=C2
848	1.937	417.12265	Kaempferol 3-alpha-L-arabinopyranoside	[M-H]-	417.12012	C20H18O10	Flavonoid-3-O-glycosides	RNVUDWOQYYWXBJ-IEGSVRCHSA-N	O=C1C(OC2OCC(O)C(O)C2O)=C(OC=3C=C(O)C=C(O)C13)C=4C=CC(O)=CC4
849	1.939	442.03839	3-(4-chlorophenyl)-5-hydroxy-10-(pyridin-3-yl)-9,10-dihydropyrano[2,3-f]chromene-4,8-dione	[M+H]+	442.04001	C23H14ClNO5	Pyranoisoflavonoids	VVWFFNXZRDYJPX-UHFFFAOYSA-N	O=C1OC2=CC(O)=C3C(=O)C(=COC3=C2C(C=4C=NC=CC4)C1)C=5C=CC(Cl)=CC5
850	1.944	133.01329	Malic acid	[M-H]-	133.01425	C4H6O5	Beta hydroxy acids and derivatives	BJEPYKJPYRNKOW-UHFFFAOYNA-N	O=C(O)CC(O)C(=O)O
851	1.947	508.2023	(1S,4aS,7aR)-7-methyl-1-[(2S,3R,4S,5S,6R)-3,4,5-trihydroxy-6-(hydroxymethyl)oxan-2-yl]oxy-4a-[(2S,3R,4R,5R,6S)-3,4,5-trihydroxy-6-	[M+H]+	508.20245	C21H30O13	Iridoid O-glycosides	WDMQLCVSWBENIY-QZTRPTDZSA-N	O=C1C=C(C)C2C(OC=CC12OC3OC(C)C(O)C(O)C3O)OC4OC(CO)C(O)C(O)C4O
852	1.947	290.08679	N-Fructosyl pyroglutamate	[M-H]-	290.08719	C11H17NO8	N-Fructosyl amino acids	VMOJEEZNLOLYBG-UHFFFAOYNA-N	O=C(O)C1N(C(=O)CC1)CC2(O)(OC(CO)C(O)C2(O))
853	1.961	485.01715	5-(1,2,3,4,5,6-hexahydroxyhexoxy)-6-hydroxy-3-methoxy-9-methyl-4,5-dihydro-3H-pyrano[3,4-h][1]benzoxepine-2,7-dione	[M-H]-	485.01373	C21H26O13	Benzoxepines	NSIUWPVISVFSGF-UHFFFAOYSA-N	O=C1OC(=CC=2C=C3OC(=O)C(OC)CC(OC(O)C(O)C(O)C(O)C(O)CO)C3=C(O)C12)C
854	1.967	561.11017	4-(5-(3-(3,4-dimethoxyphenyl)-4,8-dioxo-4,8,9,10-tetrahydropyrano[2,3-f]chromen-10-yl)furan-2-yl)benzoic acid	[M+H]+	561.10999	C31H22O9	Pyranoisoflavonoids	OQANKTWSGPYURN-UHFFFAOYSA-N	O=C(O)C=1C=CC(=CC1)C=2OC(=CC2)C3C=4C(OC(=O)C3)=CC=C5C(=O)C(=COC54)C=6C=CC(OC)=C(OC)C6
855	1.973	267.04596	HOMOCYSTINE	[M-H]-	267.047	C8H16N2O4S2	Alpha amino acids	ZTVZLYBCZNMWCF-UHFFFAOYSA-N	O=C(O)C(N)CCSSCCC(N)C(=O)O
856	1.992	457.17859	medermycin	[M+H]+	457.17401	C24H27NO8	Benzoisochromanequinones	NYJGMJFBEVSQNN-UHFFFAOYSA-N	O=C1C2=CC=C(C(O)=C2C(=O)C3=C1C4OC(=O)CC4OC3C)C5OC(C)C(O)C(N(C)C)C5
857	2.001	149.05925	Cinnamic acid	[M+H]+	149.06	C9H8O2	Cinnamic acids	WBYWAXJHAXSJNI-VOTSOKGWSA-N	O=C(O)C=CC=1C=CC=CC1
858	2.007	349.15976	Eurycomalactone	[M+H]+	349.16	C19H24O6	Steroid lactones	OGHYZHNTIINXEO-JGXXKITOSA-N	O=C1OC2C(O)C3C(C(=O)CC4C(=CC(=O)C(O)C43C)C)(C)C1C2C

859	2.007	441.18497	Gomisin H	[M+H]+	441.189	C23H30O7	Hydrolyzable tannins	NLJJSPKWNBUDNS-DAOPMYJZSA-N	OC1=C(OC)C(OC)=CC2=C1C3=C(OC)C(OC)=C(OC)C=C3CC(O)(C)C(C)C2
860	2.008	164.07065	Phenylalanine	[M-H]-	164.0717	C9H11NO2	Phenylalanine and derivatives	COLNVLDHVKWLRT-UHFFFAOYNA-N	O=C(O)C(N)CC=1C=CC=CC1
861	2.011	311.13065	Desloratadine	[M+H]+	311.13095	C19H19ClN2	Benzocycloheptapyridines	JAUOIFJMECXRGI-UHFFFAOYSA-N	C1=CC=2CCC3=CC(=CC=C3C(=C4CCNCC4)C2N=C1)Cl
862	2.015	361.97177	1-Pentanesulfonamide, 1,1,2,2,3,3,4,4,5,5,5-undecafluoro-N-methyl-	[M-H]-	361.97144	C6H4F11NO2S	PFSA	BKKNDZBSSSAGIB-UHFFFAOYSA-N	O=S(=O)(NC)C(F)(F)C(F)(F)C(F)(F)C(F)(F)C(F)(F)F
863	2.022	279.01834	purpurin	[M+H]+	279.01999	C23H22O6	8-prenylated flavanones	QLKSLGRVBGVPPG-XODARUQUSA-N	O=C(OC1C2C=3C(OC2OC1(C)C)=CC=C4C(=O)CC(OC43)C=5C=CC=CC5)C
864	2.027	326.12341	Acetaminophen glucuronide	[M-H]-	326.12726	C14H17NO8	Phenolic glycosides	IPROLSVTVHAQLE-BYNIDDHOSA-N	O=C(O)C1OC(OC2=CC=C(N=C(O)C)C=C2)C(O)C(O)C1O
865	2.034	392.17993	4-methyl-N-(((1S,9aR)-octahydro-1H-quinolizin-1-yl)methyl)-2-phenylthiazole-5-carboxamide	[M+H]+	392.17999	C21H27N3OS	Quinolizines	CDDYZYIBFIQEGM-ZWKOTPCHSA-N	O=C(NCC1CCCN2CCCCC21)C=3SC(=NC3C)C=4C=CC=CC4
866	2.059	115.00298	FUMARATE	[M-H]-	115.003	C4H4O4	Dicarboxylic acids and derivatives	VZCYOOQTPOCHFL-UHFFFAOYSA-N	O=C(O)C=CC(=O)O
867	2.066	319.98358	PFSM-carboxylic_acid	[M-H]-	319.98331	C6H6F7NO4S	PFSA	QXQNEIFHTSUFRH-UHFFFAOYSA-N	O=C(O)CN(C)S(=O)(=O)C(F)(F)C(F)(F)C(F)(F)F
868	2.072	100.07543	2-Piperidone	[M+H]+	100.0757	C5H9NO	Piperidinones	XUWHAWMETYGRKB-UHFFFAOYSA-N	C1CCN=C(C1)O
869	2.079	311.12274	gamma-Glutamyltyrosine	[M+H]+	311.12375	C14H18N2O6	Tyrosine and derivatives	VVLXCWVSSLFQDS-UHFFFAOYNA-N	O=C(O)C(N=C(O)CCC(N)C(=O)O)CC1=CC=C(O)C=C1
870	2.081	278.15903	1-(1-hydroxybutyl)-1,3,4,5,6,7-hexahydro-2-benzofuran-4,5,6,7-tetrol	[M+H]+	278.15979	C12H20O6	Isobenzofurans	TWDKEWYUAIMHMA-UHFFFAOYSA-N	OC1C2=C(C(O)C(O)C1O)C(OC2)C(O)CCC
871	2.084	254.16238	Anabasamine	[M+H]+	254.16518	C16H19N3	Alkaloids	TZRDBHMKTWECOV-UHFFFAOYNA-N	CN1CCCCC1C1=CN=C(C=C1)C1=CN=CC=C1
872	2.116	443.19943	pomiferin	[M+H]+	443.2012	C25H24O6	6-prenylated isoflavanones	GHCZYXUOYFOXIP-UHFFFAOYSA-N	O=C1C(=COC=2C=3C=CC(OC3C(=C(O)C12)CC=C(C)C)(C)C)C=4C=CC(O)=C(O)C4
873	2.118	204.12241	O-Acetyl-L-carnitine	[M+H]+	204.12248	C9H18NO4	Acyl carnitines	RDHQFKQIGNGIED-MRVPVSSYSA-O	CC(=O)O[C=H](CC(O)=O)C[N+](C)(C)C
874	2.124	128.14313	DL-Coniine	[M+H]+	128.14337	C8H17N	Alkaloids and derivatives	NDNUANOUGZGEPO-UHFFFAOYNA-N	CCCC1CCCCN1
875	2.133	225.09425	3-(4-hydroxyphenyl)-1-phenyl-2-propen-1-one	[M+H]+	225.09	C15H12O2	Retrochalcones	PWWCDTYUYPOAIU-DHZHZOJOSA-N	O=C(C=CC1=CC=C(O)C=C1)C=2C=CC=CC2
876	2.136	281.14859	(5R)-trans-1,7-diphenyl-5-hydroxy-6-hepten-3-one	[M+H]+	281.14999	C19H20O2	Linear diarylheptanoids	NEQGOKAKPXXESR-YLZCUGDYSA-N	O=C(CCC=1C=CC=CC1)CC(O)C=CC=2C=CC=CC2
877	2.146	174.02158	Orthanilic acid	[M+H]+	174.02194	C6H7NO3S	Benzenesulfonic acids and derivatives	ZMCHBSMFKQYNKA-UHFFFAOYSA-N	O=S(=O)(O)C=1C=CC=CC1N
878	2.163	307.09982	mollugin	[M+H]+	307.10001	C17H16O4	Naphthopyrans	VLGATXOTCNBWIT-UHFFFAOYSA-N	O=C(OC)C=1C(O)=C2C=CC=CC2=C3OC(C=CC31)(C)C
879	2.169	342.13794	Feruloyl O-methyldehydrodopamine	[M+H]+	342.13489	C19H19NO5	Ferulic acid and derivatives	IQGMDSXKRPLDBE-CPSCNVPRSA-N	COC1=CC(\C=C\NC(=O)\C=C\C2=CC=C(O)C(OC)=C2)=CC=C1O
880	2.171	196.09621	Methyltyrosinate	[M+H]+	196.09682	C10H13NO3	Tyrosine and derivatives	MWZPENIJLUWBSY-UHFFFAOYNA-N	O=C(OC)C(N)CC1=CC=C(O)C=C1
881	2.185	392.18021	Corydaline	[M+H]+	392.17999	C22H27NO4	Protoberberine alkaloids and derivatives	VRSRXLJTYQVOHC-QUXALOBESA-N	O(C1=CC=C2C(=C1OC)CN3CCC4=CC(OC)=C(OC)C=C4C3C2C)C
882	2.192	323.12186	Neobavaisoflavone	[M+H]+	323.12	C20H18O4	Isoflavones	OBGPEBYHGIUFBN-UHFFFAOYSA-N	O=C1C(=COC2=CC(O)=CC=C12)C=3C=CC(O)=C(C3)CC=C(C)C
883	2.206	261.0932	FARNESOL	[M+H]+	261.09418	C15H26O	Sesquiterpenoids	CRDAMVZIKSXKFV-YFVJMOTDSA-N	OCC=C(C)CCC=C(C)CCC=C(C)C
884	2.223	422.21112	(S)-2-(3-(2,5-dimethyl-1H-pyrrol-1-yl)propanoyl)-6,7-dimethoxy-N-methyl-1,2,3,4-tetrahydroisoquinoline-3-carboxamide	[M+H]+	422.20999	C22H29N3O4	Alpha amino acid amides	OOAFHWZQYGAPFW-SFHVURJKSA-N	O=C(NC)C1N(C(=O)CCN2C(=CC=C2C)C)CC3=CC(OC)=C(OC)C=C3C1
885	2.227	539.28168	MUPIROCIN	[M+H]+	539.28137	C26H44O9	Medium-chain fatty acids	MINDHVHHQZYEEK-HBBNESRFSA-N	O=C(O)CCCCCCCCOC(=O)C=C(C)CC1OCC(CC2OC2C(C)C(O)C)C(O)C1O
886	2.23	453.19492	(1R,5S)-3-(1-oxo-2-(pyridin-4-ylmethyl)-1,2-dihydroisoquinoline-4-carbonyl)-3,4,5,6-tetrahydro-1H-1,5-methanopyrido[1,2-a][1,5]diazocin-	[M+H]+	453.19	C27H24N4O3	Cytisine and derivatives	HRIXOCFMOJEHEU-UXHICEINSA-N	O=C1C=CC=C2N1CC3CN(C(=O)C4=CN(C(=O)C=5C=CC=CC54)CC=6C=CN=CC6)CC2C3
887	2.235	395.20108	brucine	[M+H]+	395.20001	C23H26N2O4	Strychnos alkaloids	RRKTZKIUPZVBMF-IBTVXLQLSA-N	O=C1N2C3=CC(OC)=C(OC)C=C3C45CCN6CC7=CCOC(C1)C(C7CC64)C25
888	2.242	307.16434	Epigallocatechin	[M+H]+	307.16379	C15H14O7	Epigallocatechins	XMOCLSLCDHWDHP-IUODEOHRSA-N	OC=1C=C(O)C2=C(OC(C3=CC(O)=C(O)C(O)=C3)C(O)C2)C1
889	2.243	240.17947	Benzphetamine	[M+H]+	240.17468	C17H21N	Amphetamines and derivatives	YXKTVDFXDRQTKV-UHFFFAOYNA-N	C=1C=CC(=CC1)CN(C)C(C)CC=2C=CC=CC2
890	2.243	195.12186	Octyl acetate	[M+H]+	195.13	C10H20O2	Fatty alcohol esters	YLYBTZIQSIBWLI-UHFFFAOYSA-N	O=C(OCCCCCCCC)C
891	2.247	272.15967	Napropamid	[M+H]+	272.16449	C17H21NO2	Naphthalenes	WXZVAROIGSFCFJ-UHFFFAOYNA-N	CCN(CC)C(=O)C(C)OC1=CC=CC2=CC=CC=C12
892	2.248	368.09482	6,10a-dihydroxy-4-(hydroxymethyl)-4,7,11b-trimethyl-1,2,3,4a,5,6,6a,7,11,11a-decahydronaphtho[2,1-f][1]benzofuran-9-one	[M+H]+	368.09167	C20H30O5	Naphthofurans	LLKIVVHUJOPYIV-UHFFFAOYSA-N	O=C1OC2(O)C(=C1)C(C)C3C(O)CC4C(C)(CO)CCCC4(C)C3C2
893	2.249	420.17572	4-amino-5-(5-hydroxy-2',6-dimethyl-5'-prop-1-en-2-ylspiro[7-oxabicyclo[4.1.0]heptane-3,1'-cyclopentane]-2-yl)oxy-5-oxopentanoic acid	[M+H]+	420.17828	C20H31NO6	Glutamic acid and derivatives	RMFZYEBIQCUWPE-UHFFFAOYSA-N	O=C(O)CCC(N)C(=O)OC1C2OC2(C)C(O)CC13C(C(=C)C)CCC3C
894	2.258	331.19727	2',4-Dihydroxy-3,4',6'-Trimethoxychalcone	[M+H]+	331.19855	C18H18O6	2'-Hydroxychalcones	BXXKRESPUSMTEI-FNORWQNLSA-N	O=C(C=CC1=CC=C(O)C(OC)=C1)C=2C(O)=CC(OC)=CC2OC
895	2.258	158.08086	Acetylproline	[M+H]+	158.0816	C7H11NO3	Amino acids	GNMSLDIYJOSUSW-UHFFFAOYNA-N	O=C(O)C1N(C(=O)C)CCC1
896	2.264	378.16501	Droperidol	[M-H]-	378.16232	C22H22FN3O2	Alkyl-phenylketones	RMEDXOLNCUSCGS-UHFFFAOYSA-N	O=C(C1=CC=C(F)C=C1)CCCN2CC=C(N3C(O)=NC=4C=CC=CC43)CC2
897	2.269	127.0396	4-Hydroxy-6-methyl-2-pyrone	[M+H]+	127.03897	C6H6O3	Pyranones and derivatives	NSYSSMYQPLSPOD-UHFFFAOYSA-N	O=C1OC(=CC(O)=C1)C
898	2.271	131.03413	Glutaric acid	[M-H]-	131.03499	C5H8O4	Dicarboxylic acids and derivatives	JFCQEDHGNNZCLN-UHFFFAOYSA-N	OC(=O)CCCC(O)=O
899	2.273	218.10214	D-(+)-Pantothenic acid	[M-H]-	218.10339	C9H17NO5	Secondary alcohols	GHOKWGTUZJEAQD-ZETCQYMHSA-N	CC(C)(CO)[C=H](O)C(O)=NCCC(O)=O
900	2.274	260.11197	(Z)-2-methyl-4-[(2R,3R,4S,5S,6R)-3,4,5-trihydroxy-6-(hydroxymethyl)oxan-2-yl]oxybut-2-enenitrile	[M+H]+	260.11285	C11H17NO6	Fatty acyl glycosides of mono- and disaccharides	ZMELGIPFIBWPHX-GMLQCYRESA-N	N#CC(=CCOC1OC(CO)C(O)C(O)C1O)C
901	2.279	267.133	honokiol	[M+H]+	267.14001	C18H18O2	Biphenyls and derivatives	FVYXIJYOAGAUQK-UHFFFAOYSA-N	OC1=CC=C(C=C1C=2C=CC(O)=C(C2)CC=C)CC=C

902	2.279	290.17026	Hyoscyamine	[M+H]+	290.17001	C17H23NO3	Tropane alkaloids	RKUNBYITZUJHSG-LGGPCSOHSA-N	O=C(OC1CC2N(C)C(CC2)C1)C(C=3C=CC=CC3)CO
903	2.284	281.09781	1,5,9-trihydroxy-5,7,7-trimethyl-4,5a,6,8,8a,9-hexahydro-1H-azuleno[5,6-c]furan-3-one	[M-H]-	281.09955	C15H22O5	Terpene lactones	MWDNWQAVYQDZQI-UHFFFAOYSA-N	O=C1OC(O)C2=C1CC(O)(C)C3CC(C)(C)CC3C2O
904	2.29	233.14841	Threonylleucine (isomer of 809, 926)	[M+H]+	233.151	C10H20N2O4	Dipeptides	BQBCIBCLXBKYHW-UHFFFAOYNA-N	O=C(O)C(NC(=O)C(N)C(O)C)CC(C)C
905	2.292	378.9436	7-Chloro-perfluoroheptanoic acid	[M-H]-	378.94006	C7HClF12O2	PFSA	WMFYUMIVMKSLSX-UHFFFAOYSA-N	O=C(O)C(F)(F)C(F)(F)C(F)(F)C(F)(F)C(F)(F)C(F)(F)Cl
906	2.292	342.96674	FT-sulfonic_acid	[M-H]-	342.96921	C6H5F9O4S	PFSA	FVJAERWLICOZSD-UHFFFAOYSA-N	O=S(=O)(O)C(O)CC(F)(F)C(F)(F)C(F)(F)C(F)(F)F
907	2.299	140.07011	3-hydroxy-1,2-dimethylpyridin-4(1H)-one	[M+H]+	140.07001	C7H9NO2	Methylpyridines	TZXKOCQBRNJULO-UHFFFAOYSA-N	O=C1C=CN(C(=C1O)C)C
908	2.303	203.138	Ala-Leu	[M+H]+	203.13902	C9H18N2O3	Peptides	RDIKFPRVLJLMER-UHFFFAOYNA-N	O=C(O)C(N=C(O)C(N)C)CC(C)C
909	2.303	356.1192	Duguetine	[M+H]+	356.117	C20H21NO5	Hydroxy-7-aporphines	ZAVWGRCRKSHIMO-OALUTQOASA-N	OC1C2=CC(OC)=C(OC)C=C2C=3C=4OCOC4C=C5C3C1N(C)CC5
910	2.319	189.12254	Gly-Leu	[M+H]+	189.12337	C8H16N2O3	Peptides	DKEXFJVMVGETOO-UHFFFAOYNA-N	O=C(O)C(N=C(O)CN)CC(C)C
911	2.33	393.11371	(1R,2E,8S,10R,11S)-10,11-dihydroxy-6-(methoxymethyl)-1,10-dimethyl-5-oxo-4,14-dioxatricyclo[9.2.1.0?,?]tetradeca-2,6-dien-8-yl 2-methylprop-2-	[M-H]-	393.1105	C20H26O8	Butenolides	QYCVLCCQAORDAF-JVYLMLIESA-N	O=C1OC2=CC3(OC(O)(CC3)C(O)(C)CC(OC(=O)C(=C)C)C2=C1COC)C
912	2.339	290.15887	brevicarine	[M+H]+	290.16	C17H21N3	Harmala alkaloids	OMGIBPZQATWNBX-UHFFFAOYSA-N	N=1C=C(C=2C=3C=CC=CC3NC2C1C)CCCCNC
913	2.343	114.05534	L-PROLINE	[M-H]-	114.056	C5H9NO2	Proline and derivatives	ONIBWKKTOPOVIA-UHFFFAOYSA-N	O=C(O)C1NCCC1
914	2.344	501.26456	2-[(6,7-dimethoxy-3,4-dihydroisoquinolin-1-yl)methyl]-3-ethyl-9,10-dimethoxy-2,3,4,6,7,11b-hexahydro-1H-benzo[a]quinolizine	[M+H]+	501.26294	C29H38N2O4	Emetine alkaloids	FBRKYRSUSJWLHH-UHFFFAOYSA-N	N1=C(C=2C=C(OC)C(OC)=CC2CC1)CC3CC4C5=CC(OC)=C(OC)C=C5CN4CC3CC
915	2.345	122.02417	NIACIN	[M-H]-	122.02475	C6H5NO2	Pyridinecarboxylic acids	PVNIIMVLHYAWGP-UHFFFAOYSA-N	O=C(O)C=1C=NC=CC1
916	2.355	188.07187	Atrazine-desethyl	[M+H]+	188.06975	C6H10ClN5	1,3,5-triazine-2,4-diamines	DFWFIQKMSFGDCQ-UHFFFAOYSA-N	CC(C)N=C1NC(Cl)=NC(=N)N1
917	2.361	343.15005	8,9-epoxy-3,10-diisobutyryloxythymol	[M+H]+	343.14999	C18H24O5	Phenol esters	OLARKEMZPWGFJU-UHFFFAOYSA-N	O=C(OC1=CC(=CC=C1C2(OC2)COC(=O)C(C)C)C)C(C)C
918	2.364	232.15367	Butyryl carnitine (isomer of 920)	[M+H]+	232.1553	C11H21NO4	Acyl carnitines	LRCNOZRCYBNMEP-UHFFFAOYNA-N	O=C([O-])CC(OC(=O)C(C)C)C[N+](C)(C)C
919	2.372	347.19083	Rosmanol	[M+H]+	347.19	C20H26O5	Diterpene lactones	LCAZOMIGFDQMNC-FORWCCJISA-N	O=C1OC2C(O)C3=CC(=C(O)C(O)=C3C14CCCC(C)(C)C24)C(C)C
920	2.377	374.20126	3-(1H-indol-1-yl)-1-(8-methoxy-3,4-dihydro-1H-pyrido[4,3-b]indol-2(5H)-yl)propan-1-one	[M+H]+	374.20001	C23H23N3O2	N-alkylindoles	KQJOTRBNSBFLSW-UHFFFAOYSA-N	O=C(N1CC=2C=3C=C(OC)C=CC3NC2CC1)CCN4C=CC=5C=CC=CC54
921	2.379	105.06947	Styrene	[M+H]+	105.06988	C8H8	Styrenes	PPBRXRYQALVLMV-UHFFFAOYSA-N	C=CC=1C=CC=CC1
922	2.382	231.16911	Leucylvaline	[M+H]+	231.1713	C11H22N2O3	Dipeptides	MDSUKZSLOATHMH-UHFFFAOYNA-N	O=C(O)C(NC(=O)C(N)CC(C)C)C(C)C
923	2.402	488.2301	4-[[4-hydroxy-2-(2-methylpropyl)-1-oxo-3,3a-dihydro-2H-imidazo[1,2-a]indol-4-yl]methyl]-1-methyl-2,4-dihydro-1H-pyrazino[2,1-b]quinazoline-	[M+H]+	488.22922	C27H29N5O4	Quinazolines	XQIGVMVJGPFNDE-UHFFFAOYSA-N	O=C1C=2C=CC=CC2N=C3N1C(C(O)=NC3C)CC4(O)C=5C=CC=CC5N6C(=O)C(NC64)CC(C)C
924	2.42	157.08574	4,5-dihydroxy-3-propylcyclopent-2-en-1-one	[M+H]+	157.08592	C8H12O3	1,2-diols	LFRFNLMDZHTVHB-UHFFFAOYSA-N	O=C1C=C(CCC)C(O)C1O
925	2.42	100.11185	Cyclohexanamine	[M+H]+	100.11208	C6H13N	Cyclohexylamines	PAFZNILMFXTMIY-UHFFFAOYSA-N	NC1CCCCC1
926	2.42	146.117	N-Methylisoleucine	[M+H]+	146.11749	C7H15NO2	Isoleucine and derivatives	KSPIYJQBLVDRRI-UHFFFAOYSA-N	O=C(O)C(NC)C(C)CC
927	2.427	449.19739	Cycloveratrylene	[M-H]-	449.19696	C27H30O6	Anisoles	YECHEDVCXXVLIY-UHFFFAOYSA-N	O(C=1C=C2C(=CC1OC)CC3=CC(OC)=C(OC)C=C3CC4=CC(OC)=C(OC)C=C4C2)C
928	2.433	317.18011	Sodium Tetradecyl Sulfate	[M+H]+	317.17569	C14H29NaO4S	Sulfuric acid monoesters	UPUIQOIQVMNQAP-UHFFFAOYSA-M	[Na+].O=S(=O)([O-])OCCCCCCCCCCCCCC
929	2.445	255.13327	Helenine	[M+H]+	255.1355	C15H20O2	Eudesmanolides, secoeudesmanolides, and	PXOYOCNNSUAQNS-AGNJHWRGSA-N	O=C1OC2CC3(C(=CC2C1=C)C(C)CCC3)C
930	2.464	349.13773	(2-hydroxy-3,6-diphenylcyclohexyl) acetate	[M+H]+	349.13718	C20H22O3	Cyclohexanols	RRGRXDIGPVAQIW-UHFFFAOYSA-N	O=C(OC1C(O)C(C=2C=CC=CC2)CCC1C=3C=CC=CC3)C
931	2.467	391.21008	Rhodojaponin III	[M+H]+	391.20999	C20H32O6	Grayanoids	VUMZHZYKXUYIHM-GLHQSWFFSA-N	OC1CC23CC(O)(C)C(CCC2C(O)(C)C4C5OC5C(C)(C)C14O)C3O
932	2.474	321.16434	Artemether (SM-224)	[M+H]+	321.16699	C16H26O5	Artemisinins	SXYIRMFQILZOAM-RDAPPBCGSA-N	O1OC23C4OC(OC)C(C)C3CCC(C)C2CCC1(O4)C
933	2.478	542.24249	13-(2-(4-(2-methoxyphenyl)piperazin-1-yl)-2-oxoethyl)-7,8-dihydroindolo[2',3':3,4]pyrido[2,1-b]quinazolin-5(13H)-one	[M+H]+	542.23999	C31H29N5O3	Beta carbolines	OJFGYTHMOAYBRB-UHFFFAOYSA-N	O=C1C=2C=CC=CC2N=C3C4=C(C=5C=CC=CC5N4CC(=O)N6CCN(C=7C=CC=CC7OC)CC6)CCN13
934	2.488	298.12949	(3,4,5-trihydroxy-6-methyloxan-2-yl) 2-(methylamino)benzoate	[M+H]+	298.12851	C14H19NO6	Hexoses	XLGJBANZFAQPOK-UHFFFAOYSA-N	O=C(OC1OC(C)C(O)C(O)C1O)C=2C=CC=CC2NC
935	2.488	254.10384	5,6,7-Trimethoxycoumarin	[M+H]+	254.10229	C12H12O5	Coumarins and derivatives	FOBNRKTURPWTQX-UHFFFAOYSA-N	O=C1OC2=CC(OC)=C(OC)C(OC)=C2C=C1
936	2.488	220.11949	Cis-Zeatin	[M+H]+	220.11929	C10H13N5O	6-alkylaminopurines	UZKQTCBAMSWPJD-UQCOIBPSSA-N	C\C(CO)=C\CNC1=C2N=CN=C2NC=N1
937	2.488	390.13168	N-(1,3-dihydroxy-2-methylpropan-2-yl)-3-methyl-4-oxo-2-phenyl-4H-chromene-8-carboxamide	[M+H]+	390.13	C21H21NO5	Flavones	UYXBVRMASFBLKM-UHFFFAOYSA-N	O=C(NC(C)(CO)CO)C1=CC=CC=2C(=O)C(=C(OC12)C=3C=CC=CC3)C
938	2.489	352.13742	Tazarotene (Avage)	[M+H]+	352.13699	C21H21NO2S	Retinoids	OGQICQVSFDPSEI-UHFFFAOYSA-N	O=C(OCC)C1=CN=C(C#CC2=CC=C3SCCC(C3=C2)(C)C)C=C1
939	2.491	285.17957	Alpinetin Methyl Ether	[M+H]+	285.17853	C17H16O4	7-O-methylated flavonoids	IAFBOKYTDSDNHV-UHFFFAOYSA-N	O=C1C=2C(OC)=CC(OC)=CC2OC(C=3C=CC=CC3)C1
940	2.493	288.15585	Atropine	[M-H]-	288.16049	C17H23NO3	Tropane alkaloids	RKUNBYITZUJHSG-PJPHBNEVSA-N	O=C(OC1CC2N(C)C(CC2)C1)C(C=3C=CC=CC3)CO
941	2.495	461.2121	Pseudoaspidin	[M+H]+	461.20999	C25H32O8	Diphenylmethanes	ASBDWVACJRRBIZ-UHFFFAOYSA-N	O=C(C=1C(O)=C(C(O)=C(C1OC)C)CC2=C(O)C(C(=O)CCC)=C(OC)C(=C2O)C)CCC
942	2.498	222.1118	Hydrocotarnine	[M+H]+	222.11247	C12H15NO3	Tetrahydroisoquinolines	XXANNZJIZQTCBP-UHFFFAOYSA-N	COC1=C2CN(C)CCC2=CC2=C1OCO2
943	2.506	296.13388	2-methylidene-4-[(2R,3R,4S,5S,6R)-3,4,5-trihydroxy-6-(hydroxymethyl)oxan-2-yl]oxybutanoic acid	[M+H]+	296.13397	C11H18O8	Saccharolipids	QJALTHDWQWOLLT-ZBGLXGBJSA-N	O=C(O)C(=C)CCOC1OC(CO)C(O)C(O)C1O
944	2.509	259.1669	3,7-Epoxycaryophyllan-6-One	[M+H]+	259.16684	C15H24O2	Oxepanes	PPGUUDJMTMGALC-UHFFFAOYSA-N	O=C1CCC2(OC1(C)CCC3C2CC3(C)C)C

945	2.511	375.17459	Cyproterone	[M+H]+	375.17215	C22H27ClO3	Gluco/mineralocorticoids, progestogins and	DUSHUSLJJMDGTE-UHFFFAOYNA-N	CC(C1(CCC2C3C=C(C4=CC(C5CC5C4(C)C3CCC21C)=O)Cl)O)=O
946	2.52	305.16125	Propentofylline	[M-H]-	305.1619	C15H22N4O3	Xanthines	RBQOQRRFDPXAGN-UHFFFAOYSA-N	O=C1C2=C(N=CN2CCC)N(C(=O)N1CCCCC(=O)C)C
947	2.526	357.21194	galbulin	[M+H]+	357.20999	C22H28O4	Aryltetralin lignans	WYBOVISLCPAJFV-QLEMLULZSA-N	O(C1=CC=C(C=C1OC)C2C3=CC(OC)=C(OC)C=C3CC(C)C2C)C
948	2.534	174.02164	ANILINE-2-SULFONIC ACID	[M+H]+	174.02	C6H7N	Aniline and substituted anilines	PAYRUJLWNCNPSJ-UHFFFAOYSA-N	NC=1C=CC=CC1
949	2.537	125.05965	Isovaleric acid	[M+H]+	125.06	C5H10O2	Methyl-branched fatty acids	GWYFCOCPABKNJV-UHFFFAOYSA-N	O=C(O)CC(C)C
950	2.538	291.15387	1-(8-methyl-3a,4,5,6-tetrahydro-1H-pyrazino[3,2,1-jk]carbazol-3(2H)-yl)ethanone	[M+H]+	291.14999	C17H20N2O	Carbazoles	TVYGDDMZPBKRPM-UHFFFAOYSA-N	O=C(N1CCN2C=3C=CC(=CC3C4=C2C1CCC4)C)C
951	2.538	340.14975	Tetrahydroberberine	[M+H]+	340.14999	C20H21NO4	Protoberberine alkaloids and derivatives	VZTUIEROBZXUFA-MRXNPFEDSA-N	O(C1=CC=C2C(=C1OC)CN3CCC4=CC=5OCOC5C=C4C3C2)C
952	2.544	285.17981	Oseltamivir carboxylate	[M+H]+	285.18088	C14H24N2O4	Gamma amino acids and derivatives	NENPYTRHICXVCS-YNEHKIRRSA-N	CCC(CC)O[C=H]1C=C(C[C=H](N)[C=H]1N=C(C)O)C(O)=O
953	2.545	385.15891	(2R,3S,4S,5R,6S)-2-(hydroxymethyl)-6-(3,4,5-trimethoxyphenoxy)oxane-3,4,5-triol	[M+H]+	385.159	C15H22O9	Phenolic glycosides	NBLLRWANAFOKON-ZHZXCYKASA-N	OCC1OC(OC=2C=C(OC)C(OC)=C(OC)C2)C(O)C(O)C1O
954	2.545	317.16904	(S)-[6]gingerol	[M+H]+	317.17001	C17H26O4	Gingerols	NLDDIKRKFXEWBK-CQSZACIVSA-N	O=C(CCC1=CC=C(O)C(OC)=C1)CC(O)CCCCC
955	2.554	122.09717	2,6-Xylidine	[M+H]+	122.09643	C8H11N	m-Xylenes	UFFBMTHBGFGIHF-UHFFFAOYSA-N	CC=1C=CC=C(C)C1N
956	2.557	298.09607	5'-S-Methylthioadenosine	[M+H]+	298.09683	C11H15N5O3S	5'-deoxy-5'-thionucleosides	WUUGFSXJNOTRMR-IOSLPCCCSA-N	CSC[C=H]1O[C=H]([C=H](O)[C=H]1O)N1C=NC2=C(N)N=CN=C12
957	2.557	331.19568	8-[3-oxo-2-[(E)-pent-2-enyl]cyclopenten-1-yl]octanoic acid	[M+H]+	331.19421	C18H28O3	Medium-chain fatty acids	RHVWQVSLVMXMBR-XVNBXDOJSA-N	O=C(O)CCCCCCCC1=C(C(=O)CC1)CC=CCC
958	2.561	447.112	Cynaroside	[M-H]-	447.112	C21H20O11	Flavonoid-7-O-glycosides	PEFNSGRTCBGNAN-QNDFHXLGSA-N	O=C1C=C(OC2=CC(OC3OC(CO)C(O)C(O)C3O)=CC(O)=C12)C=4C=CC(O)=C(O)C4
959	2.566	224.12794	Bufexamac	[M+H]+	224.12801	C12H17NO3	Phenylacetamides	MXJWRABVEGLYDG-UHFFFAOYSA-N	O=C(NO)CC1=CC=C(OCCCC)C=C1
960	2.567	259.00827	Bromacil	[M-H]-	259.00876	C9H13BrN2O2	Halopyrimidines	CTSLUCNDVMMDHG-UHFFFAOYNA-N	CCC(C)N1C(O)=NC(C)=C(Br)C1=O
961	2.568	146.1171	(S)-3-Amino-5-methylhexanoic acid	[M+H]+	146.11755	C7H15NO2	Beta amino acids and derivatives	MLYMSIKVLAPCAK-LURJTMIESA-N	CC(C)C[C=H](N)CC(O)=O
962	2.58	247.1279	desmotroposantonin	[M+H]+	247.13	C15H18O3	Naphthofurans	ZLKGXNSQDUJSAU-PSEJXSGSSA-N	O=C1OC2C=3C(=C(O)C=C(C3CCC2C1C)C)C
963	2.582	208.0598	7,8-dimethoxy-benzo[d][1,2]oxazin-1-one	[M+H]+	208.06	C10H9NO4	Benzoxazines	KPEBLJUVHUTNCC-UHFFFAOYSA-N	O=C1ON=CC=2C=CC(OC)=C(OC)C12
964	2.592	292.04517	1,8-dinitropyrene	[M-H]-	292.04895	C16H8N2O4	Pyrenes	BLYXNIHKOMELAP-UHFFFAOYSA-N	C1=CC=2C=CC(=C3C=CC4=C(C=CC1=C4C23)N(=O)=O)N(=O)=O
965	2.598	319.15021	[(2R)-1-[(2S)-6-oxo-2,3-dihydropyran-2-yl]propan-2-yl] 3-(3,4-dihydroxyphenyl)propanoate	[M-H]-	319.14777	C17H20O6	Catechols	DEJPAJWPCTUPPL-YPMHNXCESA-N	O=C1OC(CC=C1)CC(OC(=O)CCC2=CC=C(O)C(O)=C2)C
966	2.598	239.10242	Felbamate	[M+H]+	239.10263	C11H14N2O4	Benzene and substituted derivatives	WKGXYQFOCVYPAC-UHFFFAOYSA-N	N=C(O)OCC(C=1C=CC=CC1)COC(=N)O
967	2.598	305.14777	Miltirone	[M+H]+	305.14999	C19H22O2	Tanshinones, isotanshinones, and	FEFAIBOZOKSLJR-UHFFFAOYSA-N	O=C1C(=O)C(=CC=2C=CC3=C(C21)CCCC3(C)C)C(C)C
968	2.598	272.14792	Pesticide5_Methoprotryne_C11H21N5OS_	[M+H]+	272.15399	C11H21N5OS	Methylthio-s-triazines	DDUIUBPJPOKOMV-UHFFFAOYSA-N	N=1C(=NC(=NC1NCCCOC)NC(C)C)SC
969	2.602	135.06491	2-Deoxyribose	[M+H]+	135.06519	C5H10O4	Beta-hydroxy aldehydes	ASJSAQIRZKANQN-UHFFFAOYNA-N	O=CCC(O)C(O)CO
970	2.602	283.16464	ANISOMYCIN	[M+H]+	283.16522	C14H19NO4	Anisoles	YKJYKKNCCRKFSL-BFHYXJOUSA-N	O=C(OC1C(O)CNC1CC2=CC=C(OC)C=C2)C
971	2.604	488.25916	deltamine	[M+H]+	488.26001	C25H39NO7	Aconitane-type diterpenoid alkaloids	GLMFFCVWRKKBJB-YVAUPFRZSA-N	OC1C2C3(C)CN(CC)C4C51OCOC65CC(OC)C7CC(O)(C6C7OC)C42C(OC)CC3
972	2.617	229.15366	Leucylproline	[M+H]+	229.15511	C11H20N2O3	Dipeptides	VTJUNIYRYIAIHF-UHFFFAOYNA-N	O=C(O)C1N(C(=O)C(N)CC(C)C)CCC1
973	2.623	373.17047	2-[2-[(Z)-pent-2-enyl]-3-[3,4,5-trihydroxy-6-(hydroxymethyl)oxan-2-yl]oxycyclopentyl]acetic acid	[M-H]-	373.17352	C18H30O8	Fatty acyl glycosides of mono- and disaccharides	GJZJZRWFRZFTEE-ARJAWSKDSA-N	O=C(O)CC1CCC(OC2OC(CO)C(O)C(O)C2O)C1CC=CCC
974	2.624	192.97765	PYROCATECHUIC ACID	[M+H]+	192.98041	C7H6O4	Salicylic acids	GLDQAMYCGOIJDV-UHFFFAOYSA-N	O=C(O)C=1C=CC=C(O)C1O
975	2.625	234.16899	(2R)-2-[(2R,5S)-5-[(2S)-2-hydroxybutyl]oxolan-2-yl]propanoic acid	[M+H]+	234.16997	C11H20O4	Oxolanes	HTCUURQJNZBKIA-XFWSIPNHSA-N	O=C(O)C(C)C1OC(CC1)CC(O)CC
976	2.625	221.09212	Ethyl ?-D-glucuronide	[M-H]-	221.09399	C8H14O7	O-glucuronides	IWJBVMJWSPZNJH-UQGZVRACSA-N	O=C(O)C1OC(OCC)C(O)C(O)C1O
977	2.629	551.24854	Pseudolaric Acid A-O-beta-D-glucopyranoside	[M+H]+	551.25	C28H38O11	Diterpene lactones	IVYWRYGMQNKDQB-VHJBJYHKSA-N	O=C(OC1OC(CO)C(O)C(O)C1O)C(=CC=CC2(OC(=O)C34CC=C(C)CCC4(OC(=O)C)C2CC3)C)C
978	2.631	277.11722	1-Pentanone, 3-hydroxy-1,5-diphenyl-	[M+H]+	277.1199	C17H18O2	Alkyl-phenylketones	QJUWGGININGVSC-UHFFFAOYSA-N	O=C(C=1C=CC=CC1)CC(O)CCC=2C=CC=CC2
979	2.64	307.08417	Clobenpropit	[M-H]-	307.07898	C14H17ClN4S	Chlorobenzenes	UCAIEVHKDLMIFL-UHFFFAOYSA-N	ClC1=CC=C(C=C1)CNC(=N)SCCCC2=CN=CN2
980	2.641	145.04965	Adipic acid	[M-H]-	145.05063	C6H10O4	Medium-chain fatty acids	WNLRTRBMVRJNCN-UHFFFAOYSA-N	O=C(O)CCCCC(=O)O
981	2.647	352.18637	Eseroline Fumarate	[M+H]+	352.18668	C17H22N2O5	Pyrroloindoles	PBZRRADJWNBPNY-WLHGVMLRSA-N	O=C(O)C=CC(=O)O.OC1=CC=C2C(=C1)C3(C)CCN(C)C3N2C
982	2.647	263.15131	phenylacetylglutamine	[M-H]-	263.15192	C13H16N2O4	N-acyl-alpha amino acids	JFLIEFSWGNOPJJ-JTQLQIEISA-N	O=C(O)C(N=C(O)CC=1C=CC=CC1)CCC(=N)O
983	2.653	159.09116	1-Benzylimidazole	[M+H]+	159.09167	C10H10N2	N-substituted imidazoles	KKKDZZRICRFGSD-UHFFFAOYSA-N	N=1C=CN(C1)CC=2C=CC=CC2
984	2.673	333.16385	(5R)-5-hydroxy-1-(4-hydroxy-3-methoxyphenyl)decan-3-one	[M+H]+	333.16513	C17H26O4	Gingerols	NLDDIKRKFXEWBK-CQSZACIVSA-N	O=C(CCC1=CC=C(O)C(OC)=C1)CC(O)CCCCC
985	2.673	263.14029	Prolylphenylalanine	[M+H]+	263.14069	C14H18N2O3	Dipeptides	IWIANZLCJVYEFX-UHFFFAOYNA-N	O=C(O)C(NC(=O)C1NCCC1)CC2=CC=CC=C2
986	2.673	102.09207	valeramide	[M+H]+	102.09134	C5H11NO	Fatty amides	IPWFJLQDVFKJDU-UHFFFAOYSA-N	N=C(O)CCCC
987	2.675	299.07574	Benzoic acid + 1O, O-Hex	[M-H]-	299.07611	C13H16O8	Phenolic glycosides	XSSDYIMYZONMBL-UHFFFAOYNA-N	O=C(O)C2=CC=C(OC1OC(CO)C(O)C(O)C1(O))C=C2

988	2.677	293.11203	Climbazole	[M+H]+	293.10513	C15H17ClN2O2	Phenol ethers	OWEGWHBOCFMBLP-UHFFFAOYNA-N	CC(C)(C)C(C(N1C=CN=C1)OC2=CC=C(C=C2)Cl)=O
989	2.68	196.06087	"3,4-DIHYDROXY-L-PHENYLALANINE"	[M-H]-	196.061	C9H11NO4	Tyrosine and derivatives	WTDRDQBEARUVNC-UHFFFAOYSA-N	O=C(O)C(N)CC1=CC=C(O)C(O)=C1
990	2.684	237.12273	Alanylphenylalanine	[M+H]+	237.1254	C12H16N2O3	Dipeptides	OMNVYXHOSHNURL-UHFFFAOYNA-N	O=C(O)C(NC(=O)C(N)C)CC1=CC=CC=C1
991	2.684	367.15933	Dehydroisoandrosterone sulfate	[M-H]-	367.15848	C19H28O5S	Sulfated steroids	CZWCKYRVOZZJNM-UHFFFAOYNA-N	O=C1CCC2C3CC=C4CC(OS(=O)(=O)O)CCC4(C)C3CCC12C
992	2.69	345.12744	santin	[M+H]+	345.12683	C18H16O7	6-O-methylated flavonoids	DWZAJFZEYZIHPO-UHFFFAOYSA-N	O=C1C(OC)=C(OC2=CC(O)=C(OC)C(O)=C12)C=3C=CC(OC)=CC3
993	2.692	348.13931	(R)-1-((4,8-dimethoxyfuro[2,3-b]quinolin-7-yl)oxy)-3-methylbutane-2,3-diol	[M+H]+	348.14001	C18H21NO6	Furanoquinolines	FGANMDNHTVJAHL-CYBMUJFWSA-N	OC(COC=1C=CC2=C(N=C3OC=CC3=C2OC)C1OC)C(O)(C)C
994	2.692	161.10818	Tryptamine (not validated)	[M+H]+	161.108	C10H12N2	Indole and derivatives	APJYDQYYACXCRM-UHFFFAOYSA-N	NCCC2=CNC1=CC=CC=C12
995	2.693	387.13974	DL-Praeruptorin A	[M+H]+	387.14001	C21H22O7	Angular pyranocoumarins	XGPBRZDOJDLKOT-IZZDOVSWSA-N	O=C1OC2=C(C=C1)C=CC=3OC(C)(C)C(OC(=O)C(=CC)C)C(OC(=O)C)C32
996	2.697	176.07047	3-Indoleacetic acid	[M+H]+	176.0706	C10H9NO2	Indole-3-acetic acid derivatives	SEOVTRFCIGRIMH-UHFFFAOYSA-N	c1ccc2c(c1)c(CC(=O)O)c[nH]2
997	2.697	227.07596	cis-resveratrol	[M-H]-	227.07137	C14H12O3	Stilbenes	LUKBXSAWLPMMSZ-UHFFFAOYSA-N	OC1=CC=C(C=CC2=CC(O)=CC(O)=C2)C=C1
998	2.698	204.14201	Crotamiton	[M+H]+	204.13829	C13H17NO	Anilides	DNTGGZPQPQTDQF-UHFFFAOYSA-N	CC=CC(N(CC)C1=CC=CC=C1C)=O
999	2.702	369.12857	gibberellic acid	[M+H]+	369.13	C19H22O6	C19-gibberellin 6-carboxylic acids	IXORZMNAPKEEDV-OBDJNFEBSA-N	O=C(O)C1C2C3(OC(=O)C2(C)C(O)C=C3)C4CCC5(O)C(=C)CC14C5
1000	2.705	571.29352	Khayanthone	[M+H]+	571.29016	C32H42O9	Limonoids	AKPJXLBXDLSOFY-YCUUMJCDSA-N	O=C(OC1CC(OC(=O)C)C2(C)C(CC(OC(=O)C)C3(C)C2CCC4(C)C(C(=O)C5OC543)C6=COC=C6)C1(C)C)C
1001	2.706	288.14313	11,22-dimethyl (6E,17E)-3,14-dioxo-8,19-bis({[(2S,3R,4S,5S,6R)-3,4,5-trihydroxy-6-(hydroxymethyl)oxan-2-yl]oxy})-4,9,15,20-	[M+H]+	288.14417	C13H18O6	O-glycosyl compounds	GKHCBYYBLTXYEV-UJPOAAIJSA-N	OCC1OC(OCC=2C=CC=CC2)C(O)C(O)C1O
1002	2.706	186.1485	Chrysanthemic Acid	[M+H]+	186.14885	C10H16O2	Monocyclic monoterpenoids	XLOPRKKSAJMMEW-UHFFFAOYSA-N	O=C(O)C1C(C=C(C)C)C1(C)C
1003	2.71	235.10803	Carbetamide	[M-H]-	235.10881	C12H16N2O3	Phenylcarbamic acid esters	AMRQXHFXNZFDCH-UHFFFAOYNA-N	CCN=C(O)C(C)OC(O)=NC1=CC=CC=C1
1004	2.736	561.31152	sodium (E)-2-((4S)-16-acetoxy-3,11-dihydroxy-4,8,10,14-tetramethyldodecahydro-1H-cyclopenta[a]phenanthren-17(2H,10H,14H)-	[M+H]+	561.31	C31H47NaO6	Steroid esters	HJHVQCXHVMGZNC-LHTWEMHRSA-M	[Na+].O=C([O-])C(=C1C(OC(=O)C)CC2(C)C1CC(O)C3C4(C)CCC(O)C(C)C4CCC32
1005	2.741	180.10284	(R)-3-Amino-4-phenylbutyric acid	[M+H]+	180.1019	C10H13NO2	Beta amino acids and derivatives	OFVBLKINTLPEGH-SECBINFHSA-N	N[C=H](CC(O)=O)CC1=CC=CC=C1
1006	2.745	295.12836	Aspartame	[M+H]+	295.12885	C14H18N2O5	Peptides	IAOZJIPTCAWIRG-QWRGUYRKSA-N	COC(=O)[C=H](Cc1ccccc1)N=C([C=H](CC(=O)O)N)O
1007	2.746	255.10432	Ketoprofen	[M+H]+	255.10158	C16H14O3	Benzophenones	DKYWVDODHFEZIM-UHFFFAOYNA-N	CC(C(O)=O)C1=CC(=CC=C1)C(=O)C1=CC=CC=C1
1008	2.749	479.16275	Flavin Mononucleotide	[M+H]+	479.16177	C17H21N4O9P	Flavin nucleotides	FVTCRASFADXXNN-SCRDCRAPSA-N	O=C1N=C(O)C2=NC3=CC(=C(C=C3N(C2=N1)CC(O)C(O)C(O)COP(=O)(O)O)C)C
1009	2.759	322.13937	Pesticide4_Pyriproxyfen_C20H19NO3_Pyridine, 2-[1-methyl-2-(4-phenoxyphenoxy)ethoxy]-	[M+H]+	322.14301	C20H19NO3	Diphenylethers	NHDHVHZZCFYRSB-UHFFFAOYSA-N	N=1C=CC=CC1OC(C)COC2=CC=C(OC=3C=CC=CC3)C=C2
1010	2.762	425.1907	Kushenol F	[M+H]+	425.19	C25H28O6	8-prenylated flavanones	XRYVAQQLDYTHCL-CMJOXMDJSA-N	O=C1C2=C(O)C=C(O)C(=C2OC(C3=CC=C(O)C=C3O)C1)CC(C(=C)C)CC=C(C)C
1011	2.774	316.07977	[2-[(E)-3,4-dihydroxypent-1-enyl]-6-oxooxan-3-yl] (E)-2-methylbut-2-enoate	[M+H]+	316.07916	C15H22O6	Delta valerolactones	WEDIEMBZPFJDNC-BZNITXMSSA-N	O=C(OC1CCC(=O)OC1C=CC(O)C(O)C)C(=CC)C
1012	2.778	229.11754	Proline-hydroxyproline	[M+H]+	229.11829	C10H16N2O4	Dipeptides	ONPXCLZMBSJLSP-UHFFFAOYNA-N	O=C(O)C1N(C(=O)C2NCCC2)CC(O)C1
1013	2.782	370.1123	kinetin riboside	[M+H]+	370.10999	C15H17N5O5	Purine nucleosides	CAGLGYNQQSIUGX-SDBHATRESA-N	OCC1OC(N2C=NC=3C(=NC=NC32)NCC=4OC=CC4)C(O)C1O
1014	2.784	480.1701	[(2R,3S,4S,5R,6R)-6-[(2S,3S,4S,5R)-3,4-dihydroxy-2,5-bis(hydroxymethyl)oxolan-2-yl]oxy-3,4,5-trihydroxyoxan-2-yl]methyl 4-	[M+H]+	480.17114	C19H26O13	O-glycosyl compounds	CDTIMEVVBBGLIF-OVUASUNJSA-N	O=C(OCC1OC(OC2(OC(CO)C(O)C2O)CO)C(O)C(O)C1O)C3=CC=C(O)C=C3
1015	2.785	407.24121	[4,5-dihydroxy-3,4-bis(hydroxymethyl)-4a,8,8-trimethyl-5,6,7,8a-tetrahydro-1H-naphthalen-1-yl] hexanoate	[M+H]+	407.24039	C21H36O6	Fatty acid esters	PBKUOHTZGOWWBK-UHFFFAOYSA-N	O=C(OC1C=C(CO)C(O)(CO)C2(C)C(O)CCC(C)(C)C12)CCCCC
1016	2.785	265.06323	2',2'-Difluoro-2'-deoxyuridine	[M+H]+	265.06305	C9H10F2N2O5	Pyrimidine 2'-deoxyribonucleosides	FIRDBEQIJQERSE-QPPQHZFASA-N	OC[C=H]1O[C=H](N2C=CC(O)=NC2=O)C(F)(F)[C=H]1O
1017	2.787	337.13873	aurapten	[M+H]+	337.13971	C19H22O3	Terpene lactones	RSDDHGSKLOSQFK-RVDMUPIBSA-N	O=C1OC=2C=C(OCC=C(C)CCC=C(C)C)C=CC2C=C1
1018	2.797	265.15353	Feruloyl putrescine (isomer of 1173)	[M+H]+	265.15469	C14H20N2O3	Ferulic acid and derivatives	SFUVCMKSYKHYLD-UHFFFAOYSA-N	O=C(C=CC=1C=CC(O)=C(OC)C=1)NCCCCN
1019	2.8	356.13199	(1R,3R,4S,5R)-1,3,4-trihydroxy-5-[(E)-3-(4-hydroxyphenyl)prop-2-enoyl]oxycyclohexane-1-carboxylic acid	[M+H]+	356.13397	C16H18O8	Quinic acids and derivatives	BMRSEYFENKXDIS-QHAYPTCMSA-N	O=C(OC1CC(O)(C(=O)O)CC(O)C1O)C=CC2=CC=C(O)C=C2
1020	2.8	361.19592	Prednisolone	[M+H]+	361.20096	C21H28O5	21-hydroxysteroids	OIGNJSKKLXVSLS-VWUMJDOOSA-N	C[C=]12C[C=H](O)[C=H]3[C=H](CCC4=CC(=O)C=C[C=]34C)[C=H]1CC[C=]2(O)C(=O)CO
1021	2.808	319.12817	Irigenol	[M+H]+	319.1286	C15H10O8	Isoflavones	DFURNQJNFBMYHM-UHFFFAOYSA-N	O=C1C(=COC2=CC(O)=C(O)C(O)=C12)C=3C=C(O)C(O)=C(O)C3
1022	2.808	128.07082	L-PIPECOLIC ACID	[M-H]-	128.071	C6H11NO2	Alpha amino acids	HXEACLLIILLPRG-UHFFFAOYSA-N	O=C(O)C1NCCCC1
1023	2.811	413.13992	Loganin	[M+H]+	413.14001	C17H26O10	Iridoid O-glycosides	AMBQHHVBBHTQBF-UOUCRYGSSA-N	O=C(OC)C1=COC(OC2OC(CO)C(O)C(O)C2O)C3C1CC(O)C3C
1024	2.812	347.13058	Aucubin	[M+H]+	347.13	C15H22O9	Iridoid O-glycosides	RJWJHRPNHPHBRN-FKVJWERZSA-N	OCC1=CC(O)C2C=COC(OC3OC(CO)C(O)C(O)C3O)C12
1025	2.819	401.23792	Bisucaberin	[M+H]+	401.23999	C18H32N4O6	Macrolactams	GTADQMQBQBOJIO-UHFFFAOYSA-N	O=C1N(O)CCCCCN=C(O)CCC(=O)N(O)CCCCCN=C(O)CC1
1026	2.832	285.12521	Articaine HCl	[M+H]+	285.12701	C13H21ClN2O3S	Alpha amino acid amides	GDWDBGSWGNEMGJ-UHFFFAOYSA-N	Cl.O=C(OC)C=1SC=C(C1NC(=O)C(NCCC)C)C
1027	2.832	110.02074	Hypotaurine	[M+H]+	110.02702	C2H7NO2S	Sulfinic acids	VVIUBCNYACGLLV-UHFFFAOYNA-N	NCCS(O)=O
1028	2.837	294.06021	N4-Acetylsulfamethoxazole	[M-H]-	294.05539	C12H13N3O4S	Benzenesulfonamides	GXPIUNZCALHVBA-UHFFFAOYSA-N	CC(O)=NC1=CC=C(C=C1)S(=O)(=O)NC1=NOC(C)=C1
1029	2.847	137.05943	2-Hydroxyacetophenone	[M+H]+	137.05971	C8H8O2	Alkyl-phenylketones	ZWVHTXAYIKBMEE-UHFFFAOYSA-N	O=C(C=1C=CC=CC1)CO
1030	2.854	287.16	2-(1-hydroxy-1-methoxy-3-methylbutyl)-6-methyl-5H-[1,3]dioxolo[4,5-c]pyridin-4-one	[M+H]+	287.16013	C13H19NO5	Pyridinones	VVLWYFUJJAWYBP-UHFFFAOYSA-N	OC=1N=C(C=C2OC(OC12)C(O)(OC)CC(C)C)C

1031	2.854	316.18579	Nordihydrocapsaicin	[M+H]+	316.17999	C17H27NO3	Methoxyphenols	VQEONGKQWIFHMN-UHFFFAOYSA-N	O=C(NCC1=CC=C(O)C(OC)=C1)CCCCCC(C)C
1032	2.855	217.07097	1-hydroxypyrene	[M-H]-	217.06589	C16H10O	Pyrenes	BIJNHUAPTJVVNQ-UHFFFAOYSA-N	C1=CC=2C=CC=3C=CC(=C4C=CC(=C1)C2C34)O
1033	2.855	253.12022	lindenenol	[M+H]+	253.12	C15H18O2	Naphthofurans	XRDJYSVGPBJZSG-HGUIZCDCSA-N	OC1C2=C(OC=C2C)CC3(C)C1C(=C)C4CC43
1034	2.875	421.2438	3-(3,4-dihydroxyphenyl)-5,7-dihydroxy-6,8-bis(3-methylbut-2-enyl)chromen-4-one	[M-H]-	421.24014	C25H26O6	6-prenylated isoflavanones	OAUIRSVJXOFAOO-UHFFFAOYSA-N	O=C1C(=COC2=C1C(O)=C(C(O)=C2CC=C(C)C)CC=C(C)C)C=3C=CC(O)=C(O)C3
1035	2.882	251.13829	Caffeoyl putrescin (isomer of 1059)	[M+H]+	251.1396	C13H18N2O3	Caffeic acid and derivatives	KTZNZCYTXQYEHT-UHFFFAOYSA-N	O=C(C=CC=1C=CC(O)=C(O)C=1)NCCCCN
1036	2.89	102.12746	1-Hexylamine	[M+H]+	102.12772	C6H15N	Monoalkylamines	BMVXCPBXGZKUPN-UHFFFAOYSA-N	CCCCCCN
1037	2.898	254.04308	Kinetin	[M+H]+	254.04387	C10H9N5O	6-alkylaminopurines	QANMHLXAZMSUEX-UHFFFAOYSA-N	N=1C=NC=2C1NC=NC2NCC=3OC=CC3
1038	2.899	359.19031	Prednisone	[M+H]+	359.1853	C21H26O5	21-hydroxysteroids	XOFYZVNMUHMLCC-UHFFFAOYNA-N	CC12CC(=O)C3C(CCC4=CC(=O)C=CC34C)C1CCC2(O)C(=O)CO
1039	2.912	285.13354	methyl 2-ethyl-4-[(3R,4R,5S)-5-hydroxy-4,5-dimethyl-2-oxooxolan-3-yl]-2-methyl-3-oxobutanoate	[M-H]-	285.13437	C14H22O6	Annonaceous acetogenins	VOYCJQSSBDMXNQ-YZGKFDMESA-N	O=C1OC(O)(C)C(C)C1CC(=O)C(C(=O)OC)(C)CC
1040	2.913	428.24899	Ranolazine (Ranexa)	[M+H]+	428.254	C24H33N3O4	Anisoles	XKLMZUWKNUAPSZ-UHFFFAOYSA-N	O=C(NC=1C(=CC=CC1C)C)CN2CCN(CC2)CC(O)COC=3C=CC=CC3OC
1041	2.919	332.09717	ADRENALINE BITARTRATE	[M-H]-	332.09869	C13H19NO9	Catechols	YLXIPWWIOISBDD-NDAAPVSOSA-N	O=C(O)C(O)C(O)C(=O)O.OC1=CC=C(C=C1O)C(O)CNC
1042	2.919	317.11469	eupomatenoid 5	[M+H]+	317.11481	C19H18O3	2-arylbenzofuran flavonoids	HMGCTPMQYVGXSC-SNAWJCMRSA-N	OC=1C=CC(=CC1OC)C=2OC3=CC=C(C=CC)C=C3C2C
1043	2.919	237.14984	Gliocladic acid_130132	[M+H]+	237.149	C14H22O4	Menthane monoterpenoids	SLVSUVFUFJKMCV-URFGDBDFSA-N	O=C(O)C(=CC1C=C(CO)CCC1C(C)C)CO
1044	2.932	418.23227	Cilazapril monohydrate (Inhibace)	[M+H]+	418.23401	C22H33N3O6	Dipeptides	JQRZBPFGBRIWSN-OXJRKUMDSA-N	O=C(O)C1N2C(=O)C(NC(C(=O)OCC)CCC=3C=CC=CC3)CCCN2CCC1.O
1045	2.936	255.04359	3-(5-phenylthiophen-2-yl)prop-2-ynyl Acetate	[M-H]-	255.04852	C15H12O2S	2,5-disubstituted thiophenes	QWLHSFULTXXQLE-UHFFFAOYSA-N	O=C(OCC#CC=1SC(=CC1)C=2C=CC=CC2)C
1046	2.939	263.138	8-acetyl-5,7-dimethoxy-2,2-dimethylchromene	[M+H]+	263.13	C15H18O4	Precocenes	JELWYGQTQBQPGH-UHFFFAOYSA-N	O=C(C1=C(OC)C=C(OC)C=2C=CC(OC21)(C)C)C
1047	2.939	372.09232	DIMBOA + O-Hex	[M-H]-	372.0929	C15H19NO10	Benzoxazinoids	WTGXAWKVZMQEDA-UHFFFAOYNA-N	O=C2N(O)C=3C=CC(OC)=CC=3(OC2(OC1OC(CO)C(O)C(O)C1(O)))
1048	2.944	144.08055	Tryptophanol (not validated)	[M+H-H2O]+	144.082	C10H11NO	Indole and derivatives	MBBOMCVGYCRMEA-UHFFFAOYSA-N	OCCC2=CNC1=CC=CC=C12
1049	2.956	265.1185	Magnolol	[M-H]-	265.12341	C18H18O2	Biphenyls and derivatives	VVOAZFWZEDHOOU-UHFFFAOYSA-N	OC1=C(C=C(CC=C)C=C1)C1=C(O)C=CC(CC=C)=C1
1050	2.957	305.07678	Glutamyl-S-(C3H5)-Cysteine sulfoxide	[M-H]-	305.07999	C11H18N2O6S	Peptides	LMNDKWXDMBGGAL-UHFFFAOYNA-N	O=C(O)C(N)CCC(=O)NC(C(=O)O)CS(=O)C=CC
1051	2.964	205.14316	3,5-dihydroxydecanoic acid	[M+H]+	205.14343	C10H20O4	Medium-chain hydroxy acids and derivatives	WQZRAWSNEARGPQ-UHFFFAOYSA-N	O=C(O)CC(O)CC(O)CCCCC
1052	2.983	180.10143	(S)-3-Amino-4-phenylbutyric acid	[M+H]+	180.1019	C10H13NO2	Beta amino acids and derivatives	OFVBLKINTLPEGH-VIFPVBQESA-N	N[C=H](CC(O)=O)CC1=CC=CC=C1
1053	2.986	190.05339	N-ACETYL-DL-METHIONINE	[M-H]-	190.054	C7H13NO3S	Methionine and derivatives	XUYPXLNMDZIRQH-UHFFFAOYSA-N	O=C(O)C(NC(=O)C)CCSC
1054	2.988	285.1077	isosakuranetin	[M-H]-	285.10324	C16H14O5	4'-O-methylated flavonoids	HMUJXQRRKBLVOO-AWEZNQCLSA-N	O=C1C=2C(O)=CC(O)=CC2OC(C3=CC=C(OC)C=C3)C1
1055	2.99	405.22357	(E)-Resveratroloside	[M-H]-	405.2236	C20H22O9	Stilbene glycosides	OLCVEOSSVCAFGR-CUYWLFDKSA-N	OC=1C=C(O)C=C(C=CC2=CC=C(OC3OC(CO)C(O)C(O)C3O)C(O)=C2)C1
1056	2.992	174.09094	7-methanesulfinylheptanenitrile	[M+H]+	174.0956	C8H15NOS	Glucosinolate breakdown metabolites	MVGLREOVTAENKK-UHFFFAOYNA-N	N#CCCCCCCS(=O)C
1057	2.998	192.10193	N,N-Diethyl-4-hydroxybenzamide	[M-H]-	192.103	C11H15NO2	Benzamides	WLRVSRJKZYZCJY-UHFFFAOYSA-N	CCN(CC)C(=O)C1=CC=C(O)C=C1
1058	2.998	190.08568	synephrine	[M+Na]+	190.086	C9H13NO2	1-hydroxy-2-unsubstituted benzenoids	YRCWQPVGYLYSOX-UHFFFAOYSA-N	OC1=CC=C(C=C1)C(O)CNC
1059	3.002	625.32233	Dauricine	[M+H]+	625.32001	C38H44N2O6	Benzylisoquinolines	AQASRZOCERRGBL-ROJLCIKYSA-N	OC1=CC=C(C=C1OC2=CC=C(C=C2)CC3C4=CC(OC)=C(OC)C=C4CCN3C)CC5C6=CC(OC)=C(OC)C=C6CCN5C
1060	3.003	352.16083	MITOMYCIN C	[M+H]+	352.16153	C15H18N4O5	Mitomycins	NWIBSHFKIJFRCO-WUDYKRTCSA-N	O=C1C2=C(C(=O)C(N)=C1C)C(COC(=N)O)C3(OC)N2CC4NC43
1061	3.005	329.17172	(2E)-N-(4-acetamidobutyl)-3-(4-hydroxy-3-methoxyphenyl)prop-2-enamide	[M+H]+	329.17133	C16H22N2O4	Hydroxycinnamic acids and derivatives	ZVMGKOAHBAIHLO-SOFGYWHQSA-N	OC(=NCCCCN=C(O)C)C=CC1=CC=C(O)C(OC)=C1
1062	3.008	300.19998	Dehydroabietamide	[M+H]+	300.19943	C20H29NO	Diterpenoids	AKKQGFKWEDCWJK-UHFFFAOYSA-N	N=C(O)C1(C)CCCC2(C3=CC=C(C=C3CCC12)C(C)C)C
1063	3.012	447.1604	2-[2-(4-methyl-2-oxo-2h-chromen-7-yloxy)-acetyl]-2,3,4,9-tetrahydro-1h-beta-carboline-3-carboxylic acid methyl ester	[M+H]+	447.16	C25H22N2O6	Beta carbolines	WNAYNBDAJBVOPW-OAQYLSRUSA-N	O=C1OC=2C=C(OCC(=O)N3CC=4NC=5C=CC=CC5C4CC3C(=O)OC)C=CC2C(=C1)C
1064	3.012	301.15369	Pesticide3_Bifenazate_C17H20N2O3_1-Methylethyl 2-(4-methoxybiphenyl-3-yl)hydrazinecarboxylate	[M+H]+	301.15399	C17H20N2O3	Biphenyls and derivatives	VHLKTXFWDRXILV-UHFFFAOYSA-N	OC(=NNC=1C=C(C=CC1OC)C=2C=CC=CC2)OC(C)C
1065	3.025	254.14897	Irgarol	[M+H]+	254.14339	C11H19N5S	Methylthio-s-triazines	HDHLIWCXDDZUFH-UHFFFAOYSA-N	CC(C)(C)NC1=NC(=NC2CC2)N=C(N1)SC
1066	3.033	400.18982	Thiethylperazine	[M+H]+	400.18756	C22H29N3S2	Phenothiazines	XCTYLCDETUVOIP-UHFFFAOYSA-N	CCSC1=CC2=C(SC3=CC=CC=C3N2CCCN2CCN(C)CC2)C=C1
1067	3.037	284.0397	CGS 15943	[M-H]-	284.03445	C13H8ClN5O	Quinazolinamines	MSJODEOZODDVGW-UHFFFAOYSA-N	C1C=1C=CC=2NC(=N)N3N=C(N=C3C2C1)C=4OC=CC4
1068	3.039	312.1459	7-methoxy-6-(1,2,3-trihydroxy-3-methylbutyl)chromen-2-one	[M+H]+	312.14417	C15H18O6	Coumarins and derivatives	PCSZTTAMZGNQNB-UHFFFAOYSA-N	O=C1OC=2C=C(OC)C(=CC2C=C1)C(O)C(O)C(O)(C)C
1069	3.04	245.18495	Leucylleucine	[M+H]+	245.187	C12H24N2O3	Dipeptides	LCPYQJIKPJDLLB-UHFFFAOYNA-N	O=C(O)C(NC(=O)C(N)CC(C)C)CC(C)C
1070	3.041	283.09219	4-methoxy-9-(2-methylbut-3-en-2-yl)furo[3,2-g]chromen-7-one	[M-H]-	283.09756	C17H16O4	5-methoxypsoralens	ZPLVEUIOSKJIDT-UHFFFAOYSA-N	O=C1OC2=C(C=C1)C(OC)=C3C=COC3=C2C(C=C)(C)C
1071	3.042	401.10739	Apodanthoside (Not validated)	[M-H]-	401.10849	C17H22O11	Iridoid glycosides	QXLZMFXGMGPPHW-UHFFFAOYNA-N	O=C(OC)C2=COC(OC1OC(CO)C(O)C(O)C1(O))C3C(C=CC23)C(=O)O
1072	3.045	262.11789	BIOTIN	[M+H]+	262.12198	C10H16N2O3S	Biotin and derivatives	YBJHBAHKTGYVGT-ZKWXMUAHSA-N	O=C(O)CCCCC1SCC2N=C(O)NC21
1073	3.046	285.10678	Epoxy (4,5Alpha)-4,5-Dihydrosantonin	[M+H]+	285.10971	C15H18O4	Eudesmanolides, secoeudesmanolides, and	GTDIWCITYRGAKM-OBRHJWQCSA-N	O=C1OC2C(CCC3(C=CC(=O)C4(OC243)C)C)C1C

1074	3.051	447.15884	Gefitinib (Iressa)	[M+H]+	447.159	C22H24ClFN4O3	Quinazolinamines	XGALLCVXEZPNRQ-UHFFFAOYSA-N	FC1=CC=C(C=C1Cl)NC=2N=CN=C3C=C(OC)C(OCCCN4CCOCC4)=CC32
1075	3.054	425.19089	6-Hydroxysumatrol	[M-H]-	425.19553	C23H22O8	Rotenones	RMAYNNFCPNGTQW-UHFFFAOYSA-N	O=C1C2=C(O)C=C3OC(C(=C)C)CC3=C2OC4C(O)OC5=CC(OC)=C(OC)C=C5C14
1076	3.054	420.14963	Chloroaustralasine C	[M+H]+	420.155	C22H26ClNO5	Pyranoquinolines	XVDZSHAEMLKCIW-DBFNBLSLSA-N	O=C(OCN1C(=O)C2=C(OC(C)(C)C3CCC(O)(C)C(Cl)C23)C=4C=CC=CC41)C
1077	3.056	350.12189	Voriconazole	[M+H]+	350.12201	C16H14F3N5O	Phenylpropanes	BCEHBSKCWLPMDN-MGPLVRAMSA-N	FC1=CC=C(C(F)=C1)C(O)(CN2N=CN=C2)C(C=3N=CN=CC3F)C
1078	3.06	311.0321	Cotarnine Chloride	[M+H]+	311.0321	[C12H15ClNO4]-	Tetrahydroisoquinolines	UONSFNPHCTWRGS-UHFFFAOYSA-M	[Cl-].OC1C2=C(OC)C=3OCOC3C=C2CCN1C
1079	3.062	283.10977	2-(4-oxoquinazolin-3(4H)-yl)ethyl isobutyrate	[M+H]+	283.10999	C14H16N2O3	Quinazolines	DXZNCVRUZDRWLR-UHFFFAOYSA-N	O=C(OCCN1C=NC=2C=CC=CC2C1=O)C(C)C
1080	3.062	237.07712	Pesticide5_Metribuzin_C8H14N4OS_4-Amino-6-(2-methyl-2-propanyl)-3-(methylsulfanyl)-1,2,4-triazin-5(4H)-one	[M+H]+	237.078	C8H14N4OS	Aryl thioethers	FOXFZRUHNHCZPX-UHFFFAOYSA-N	O=C1C(=NN=C(SC)N1N)C(C)(C)C
1081	3.064	404.14612	_130050	[M+H]+	404.147	C22H23NO5	Phenylquinolines	PJPKEOASXRGMDD-SIKLNZKXSA-N	O=C1NC=2C=CC=3C=CC(OC3C2C(O)(C4=CC=C(OC)C=C4)C1OC)(C)C
1082	3.074	261.04266	Methyl 7-Deshydroxypyrogallin-4-Carboxylate	[M-H]-	261.04044	C13H10O6	Tropolones	DQWQITFUWFBXON-UHFFFAOYSA-N	O=C(OC)C1=CC(=O)C(O)=C2C(O)=C(O)C=CC2=C1
1083	3.074	137.02448	P-HYDROXYBENZOIC ACID	[M-H]-	137.02441	C7H6O3	Hydroxybenzoic acid derivatives	FJKROLUGYXJWQN-UHFFFAOYSA-N	OC(=O)C1=CC=C(O)C=C1
1084	3.077	216.08636	N-(2-furylmethyl)-9H-purin-6-amine	[M+H]+	216.08701	C10H9N5O	6-alkylaminopurines	QANMHLXAZMSUEX-UHFFFAOYSA-N	N=1C=NC(NCC=2OC=CC2)=C3N=CNC13
1085	3.088	255.17235	alpha-Estradiol	[M+H]+	255.17435	C18H24O2	Estrogens and derivatives	VOXZDWNPVJITMN-AWDGRILASA-N	CC12CCC3C(CCC4=CC(O)=CC=C34)C1CC[C=H]2O
1086	3.089	317.11163	Tanshinone IIA	[M+H]+	317.10999	C19H18O3	Tanshinones, isotanshinones, and	HYXITZLLTYIPOF-UHFFFAOYSA-N	O=C1C(=O)C2=C(C=CC3=C2CCCC3(C)C)C=4OC=C(C14)C
1087	3.093	301.12839	5,7-dihydroxy-2-(4-hydroxy-3-methoxyphenyl)-2,3-dihydrochromen-4-one	[M-H]-	301.12933	C16H14O6	3'-O-methylated flavonoids	FTODBIPDTXRIGS-UHFFFAOYSA-N	O=C1C=2C(O)=CC(O)=CC2OC(C3=CC=C(O)C(OC)=C3)C1
1088	3.093	166.1223	Hordenine	[M+H]+	166.12	C10H15NO	Phenethylamines	KUBCEEMXQZUPDQ-UHFFFAOYSA-N	OC1=CC=C(C=C1)CCN(C)C
1089	3.097	285.12268	Prolylphenylalanine	[M+H]+	285.12094	C14H18N2O3	Dipeptides	IWIANZLCJVYEFX-UHFFFAOYSA-N	O=C(O)C(N=C(O)C1NCCC1)CC=2C=CC=CC2
1090	3.098	395.14761	Arctigenin	[M+H]+	395.147	C21H24O6	Dibenzylbutyrolactone lignans	NQWVSMVXKMHKTF-JKSUJKDBSA-N	O=C1OCC(CC2=CC=C(OC)C(OC)=C2)C1CC3=CC=C(O)C(OC)=C3
1091	3.114	376.10229	1-(3,4-dihydroxyphenyl)-6,7-dihydroxy-1,2-dihydronaphthalene-2,3-dicarboxylic acid	[M+H]+	376.10269	C18H14O8	Aryltetralin lignans	WJMFXQBNYLYADA-UHFFFAOYSA-N	O=C(O)C1=CC=2C=C(O)C(O)=CC2C(C3=CC=C(O)C(O)=C3)C1C(=O)O
1092	3.116	486.18063	(1S,3R,17S,19S)-3-hydroxy-9,9,16,16-tetramethyl-8-oxa-14,23,25-triazaheptacyclo[17.5.2.0?,??.0?,??.0?,??.0?,??.0??,??]hexacosa-	[M+H]+	486.17896	C26H29N3O4	2,2-dimethyl-1-benzopyrans	PYHKDROAWLAEDE-VKAHWXPLSA-N	O=C1N2CCCC32C(O)=NC14CC5(O)C6=CC=C7OC(C=CC7=C6N=C5C(C)(C)C4C3)(C)C
1093	3.119	383.22815	(3aR,3a1S,5S,5aS,10bS)-methyl 6-acetyl-3a-ethyl-2,3,3a,3a1,4,5,5a,6,11,12-decahydro-1H-indolizino[8,1-cd]carbazole-5-carboxylate	[M+H]+	383.23001	C23H30N2O3	Aspidospermatan-type alkaloids	DHHMVJWGDXKKSV-HFOUZCJZSA-N	O=C(OC)C1CC2(CC)CCCN3CCC4(C=5C=CC=CC5N(C(=O)C)C14)C32
1094	3.12	274.12805	phenylglucoside	[M+H]+	274.12851	C12H16O6	Phenolic glycosides	NEZJDVYDSZTRFS-RMPHRYRLSA-N	OCC1OC(OC=2C=CC=CC2)C(O)C(O)C1O
1095	3.127	343.18509	4,5-dihydroxy-4,5,6-trimethyl-2,8-dioxa-13-azatricyclo[8.5.1.0??,??]hexadec-10-ene-3,7-dione	[M+H]+	343.18634	C16H23NO6	Pyrrolizines	WOPYGCANEAIOEM-UHFFFAOYSA-N	O=C1OCC2=CCN3CCC(OC(=O)C(O)(C)C(O)(C)C1C)C23
1096	3.129	317.1116	6-(2-hydroxyethyl)-6,6a-dihydroisoindolo[2,1-a]quinazoline-5,11-dione	[M+H]+	317.10999	C17H14N2O3	Indoloquinazolines	CWGIHUKKVIGOGZ-UHFFFAOYSA-N	O=C1C=2C=CC=CC2C3N1C=4C=CC=CC4C(=O)N3CCO
1097	3.137	253.16391	Hexazinone	[M+H]+	253.16591	C12H20N4O2	Dialkylarylamines	CAWXEEYDBZRFPE-UHFFFAOYSA-N	CN(C)C1=NC(N(C2CCCCC2)C(N1C)=O)=O
1098	3.137	286.13019	Piperine	[M+H]+	286.14001	C17H19NO3	Alkaloids and derivatives	MXXWOMGUGJBKIW-YPCIICBESA-N	O=C(C=CC=CC1=CC=C2OCOC2=C1)N3CCCCC3
1099	3.14	295.12747	bakuchiol	[M+H]+	295.12744	C18H24O	Aromatic monoterpenoids	LFYJSSARVMHQJB-QIXNEVBVSA-N	OC1=CC=C(C=C1)C=CC(C=C)(C)CCC=C(C)C
1100	3.145	192.13791	4-Methylethcathinone	[M+H]+	192.138	C12H17NO	Alkyl-phenylketones	ZOXZWYWOECCBSH-UHFFFAOYSA-N	O=C(C1=CC=C(C=C1)C)C(NCC)C
1101	3.163	266.13104	8-acetamido-2-methyl-7-oxononanoic acid	[M+H]+	266.13626	C12H21NO4	Medium-chain fatty acids	UALLEVOGEQZKSX-UHFFFAOYSA-N	O=C(O)C(C)CCCCC(=O)C(N=C(O)C)C
1102	3.163	682.36823	S4:19(P3:17/F1:2)	[M+H]+	682.37207	C31H52O15	Saccharolipids	NNYRMMBHXZJRGM-UHFFFAOYNA-N	CCCCCC(=O)OC1C(OC2(CO)OC(CO)C(OC(C)=O)C2O)OC(CO)C(OC(=O)CCCC)C1OC(=O)CCCCC
1103	3.169	197.07062	benzo[b][1,8]naphthyridin-5-ol	[M+H]+	197.07001	C12H8N2O	Hydroquinolones	DAFMSUZBESPPSV-UHFFFAOYSA-N	OC1=C2C=CC=NC2=NC=3C=CC=CC31
1104	3.178	299.15927	6-Shogaol	[M+H]+	299.16	C17H24O3	Shogaols	OQWKEEOHDMUXEO-BQYQJAHWSA-N	O=C(C=CCCCCC)CCC1=CC=C(O)C(OC)=C1
1105	3.182	318.20459	Nateglinide (Starlix)	[M+H]+	318.20599	C19H27NO3	Phenylalanine and derivatives	OELFLUMRDSZNSF-BRWVUGGUSA-N	O=C(O)C(NC(=O)C1CCC(CC1)C(C)C)CC=2C=CC=CC2
1106	3.186	160.03951	2,8-Quinolinediol	[M-H]-	160.04041	C9H7NO2	Quinolones and derivatives	ZXZKYYHTWHJHFT-UHFFFAOYSA-N	OC=1N=C2C(O)=CC=CC2=CC1
1107	3.195	476.22162	1-(9-hydroxy-2-isopropyl-10-(2,3,4-trimethoxyphenyl)pyrrolo[3',4':6,7]azepino[4,3,2-cd]indol-8(2H,7H,10H)-	[M+H]+	476.22	C27H29N3O5	Benzazepines	QAPJGIXRNRUPRW-UHFFFAOYSA-N	O=C(N1C(O)=C2C(=NC3=CC=CC4=C3C(=CN4C(C)C)C2C5=CC=C(OC)C(OC)=C5OC)C1)C
1108	3.2	341.16959	Cinanserin	[M+H]+	341.16821	C20H24N2OS	Cinnamic acid amides	RSUVYMGADVXGOU-UHFFFAOYSA-N	OC(=NC=1C=CC=CC1SCCCN(C)C)C=CC=2C=CC=CC2
1109	3.205	217.09842	1,2,3,4-tetrahydro-6-methoxy-1-oxo-beta-carboline	[M+H]+	217.10001	C12H12N2O2	Beta carbolines	PWARHDUWOJUADN-UHFFFAOYSA-N	O=C1NCCC=2C=3C=C(OC)C=CC3NC12
1110	3.205	293.1235	Glutamylphenylalanine	[M-H]-	293.12369	C14H18N2O5	Amino acids	XHHOHZPNYFQJKL-UHFFFAOYNA-N	O=C(O)C(N)CCC(=O)NC(C(=O)O)CC1=CC=CC=C1
1111	3.213	337.17365	Hapalindole D	[M+H]+	337.17401	C21H24N2S	Bicyclic monoterpenoids	PPHWDUZMWNUINO-QAJUQPOASA-N	S=C=NC1C(C2=CNC=3C=CC=CC32)C(C(=C)C)CCC1(C=C)C
1112	3.22	438.23776	Lunarine	[M+H]+	438.23871	C25H31N3O4	Macrolactams	YIWJEBPTHXRHQF-LLMLIWGDSA-N	O=C1CCC23C=CC(O)=NCCCCNCCCN=C(O)C=CC=4C=CC(OC2C1)=C3C4
1113	3.222	178.05336	Cyclamate	[M-H]-	178.05434	C6H13NO3S	Cyclamates	HCAJEUSONLESMK-UHFFFAOYSA-N	C1CCC(CC1)NS(O)(=O)=O
1114	3.222	312.14291	Domoic acid	[M+H]+	312.14301	C15H21NO6	Kainoids	VZFRNCSOCOPNDB-ZDUSDAAASA-N	O=C(O)CC1C(NCC1C(=CC=CC(C(=O)O)C)C)C(=O)O
1115	3.244	269.04083	(3R)-8-hydroxy-3-(4-methoxyphenyl)-3,4-dihydroisochromen-1-one	[M-H]-	269.04114	C16H14O4	2-benzopyrans	DEFIJGJJJKEYGS-CQSZACIVSA-N	O=C1OC(C2=CC=C(OC)C=C2)CC=3C=CC=C(O)C13
1116	3.244	187.18019	Lupinine	[M+H]+	187.18048	C10H19NO	Lupinine-type alkaloids	HDVAWXXJVMJBAR-VHSXEESVSA-N	OCC1CCCN2CCCCC21

1117	3.244	283.16461	Panaxydiol	[M+H]+	283.16	C17H24O2	Long-chain fatty alcohols	DSVMWGREWREVQQ-ODQHEUEKSA-N	OC(C#CC#CC=CC(O)CCCCCCC)C=C
1118	3.253	138.05614	2-Pyridylacetic acid	[M+H]+	138.05496	C7H7NO2	Pyridines and derivatives	BPSNETAIJADFTO-UHFFFAOYSA-N	c1ccnc(c1)CC(=O)O
1119	3.253	359.23135	Ochropposinine	[M+H]+	359.233	C21H30N2O3	Beta carbolines	CMGYMWAXDJQKLJ-DEYYWGMASA-N	OCCC1CC2C=3NC=4C=C(OC)C(OC)=CC4C3CCN2CC1CC
1120	3.255	112.0389	2,3-Dihydroxypyridine	[M+H]+	112.03931	C5H5NO2	Pyridinones	GGOZGYRTNQBSSA-UHFFFAOYSA-N	c1cc(c(nc1)O)O
1121	3.261	162.05431	Indole-3-carboxylic acid	[M+H]+	162.05496	C9H7NO2	Indolecarboxylic acids and derivatives	KMAKOBLIOCQGJP-UHFFFAOYSA-N	O=C(O)C1=CNC=2C=CC=CC21
1122	3.264	316.18655	Nateglinide	[M-H]-	316.1918	C19H27NO3	Phenylalanine and derivatives	OELFLUMRDSZNSF-UHFFFAOYNA-N	O=C(O)C(N=C(O)C1CCC(CC1)C(C)C)CC=2C=CC=CC2
1123	3.268	154.04942	5-Aminosalicylic??acid	[M+H]+	154.04987	C7H7NO3	Aminobenzoic acids	KBOPZPXVLCULAV-UHFFFAOYSA-N	O=C(O)C1=CC(N)=CC=C1O
1124	3.274	167.10629	Pesticide4_Cyromazine_C6H10N6_Vetrazin	[M+H]+	167.104	C6H10N6	N-aliphatic s-triazines	LVQDKIWDGQRHTE-UHFFFAOYSA-N	N=C1NC(=N)NC(=NC2CC2)N1
1125	3.279	229.09686	Ozagrel	[M+H]+	229.097	C13H12N2O2	Cinnamic acids	SHZKQBHERIJWAO-AATRIKPKSA-N	O=C(O)C=CC1=CC=C(C=C1)CN2C=NC=C2
1126	3.281	145.04991	2-Methylglutaric acid	[M-H]-	145.05063	C6H10O4	Methyl-branched fatty acids	AQYCMVICBNBXNA-UHFFFAOYNA-N	CC(CCC(O)=O)C(O)=O
1127	3.284	190.04993	5-HYDROXYINDOLEACETATE	[M-H]-	190.05	C10H9NO3	Indole-3-acetic acid derivatives	DUUGKQCEGZLZNO-UHFFFAOYSA-N	O=C(O)CC1=CNC=2C=CC(O)=CC21
1128	3.29	490.2728	Delsoline	[M+H]+	490.26999	C25H41NO7	Aconitane-type diterpenoid alkaloids	JVBLTQQBEQQLEV-DIZROUMASA-N	OC1CCC2(COC)CN(CC)C3C4(O)C(OC)C2C13C5CC6C(OC)CC4(O)C5C6OC
1129	3.292	263.13879	Methohexital	[M+H]+	263.13901	C14H18N2O3	Barbituric acid derivatives	NZXKDOXHBHYTKP-UHFFFAOYNA-N	O=C1N=C(O)C(C(=O)N1C)(CC=C)C(C#CCC)C
1130	3.305	352.10245	Parfumine	[M-H]-	352.10706	C20H19NO5	Indanones	AHNUBWYOIHCGFN-UHFFFAOYSA-N	O=C1C2=C3OCOC3=CC=C2CC14C5=CC(O)=C(OC)C=C5CCN4C
1131	3.314	198.13458	2-Hydroxyatrazine	[M+H]+	198.13493	C8H15N5O	1,3,5-triazines	NFMIMWNQWAWNDW-UHFFFAOYSA-N	CCN=C1NC(=NC(C)C)NC(=N1)O
1132	3.315	201.0761	Diethyloxalpropionate	[M-H]-	201.07684	C9H14O5	Gamma-keto acids and derivatives	NGRAIMFUWGFAEM-UHFFFAOYSA-N	O=C(OCC)C(=O)CCC(=O)OCC
1133	3.317	554.26862	(12bS)-7-(4-ethylphenyl)-12b-methyl-2-(3,4,5-trimethoxybenzyl)-2,3,6,7-tetrahydropyrazino[1',2':1,2]pyrido[3,4-b]indole-1,4(12H,12bH)-dione	[M+H]+	554.27002	C33H35N3O5	Beta carbolines	WNGVOVVMNRIJOX-RULBIBMZSA-N	O=C1N2CC(C3=CC=C(C=C3)CC)C=4C=5C=CC=CC5NC4C2(C(=O)N(CC6=CC(OC)=C(OC)C(OC)=C6)C1)C
1134	3.319	167.03412	4-Hydroxymandelic acid	[M-H]-	167.03499	C8H8O4	1-hydroxy-2-unsubstituted benzenoids	YHXHKYRQLYQUIH-UHFFFAOYNA-N	O=C(O)C(O)C1=CC=C(O)C=C1
1135	3.326	354.11731	2-(4a,9,10a-trihydroxy-1-methyl-5,10-dioxo-3,4-dihydro-1H-benzo[g]isochromen-3-yl)acetic acid	[M+H]+	354.11835	C16H16O8	Naphthopyranones	JJKCJXIYVNHIOS-UHFFFAOYSA-N	O=C(O)CC1OC(C)C2(O)C(=O)C=3C(O)=CC=CC3C(=O)C2(O)C1
1136	3.326	435.18414	Artocaprin	[M-H]-	435.1813	C26H28O6	3-prenylated flavones	KRGDFVQWQJIMEK-RMKNXTFCSA-N	COC1=C(\C=C\C(C)C)C(O)=C2C(OC(=C(CC=C(C)C)C2=O)C2=CC=C(O)C=C2O)=C1
1137	3.327	243.1702	Isoleucylisoleucine	[M-H]-	243.1716	C12H24N2O3	Peptides	BCVIOZZGJNOEQS-UHFFFAOYNA-N	O=C(O)C(NC(=O)C(N)C(C)CC)C(C)CC
1138	3.338	217.09648	Carboline base + 4H, carboxylic acid	[M+H]+	217.10069	C12H12N2O2	Carbolines	FSNCEEGOMTYXKY-UHFFFAOYNA-N	O=C(O)C3NCC=2NC1=CC=CC=C1C=2C3
1139	3.34	251.01828	FT-thioether	[M-H]-	251.01706	C7H9F5O2S	PFSA	KVVLYHRZJIPMQE-UHFFFAOYSA-N	O=C(O)CCSCCC(F)(F)C(F)(F)F
1140	3.348	464.2598	(3S,3'R,3'aS,6'S,6aS,6bS,7'aR,9R,11bR)-3-hydroxy-3',6',10,11b-tetramethylspiro[1,2,3,4,6,6a,6b,7,8,11a-decahydrobenzo[a]fluorene-9,2'-	[M+H]+	464.25613	C27H39NO3	Jerveratrum-type alkaloids	CLEXYFLHGFJONT-GDMKTYFQSA-N	O=C1C2=C(C)C3(OC4CC(C)CNC4C3C)CCC2C5CC=C6CC(O)CCC6(C)C15
1141	3.352	595.16992	5,7-dihydroxy-2-(2-hydroxyphenyl)-3-(((2R,3S,4R,5R,6S)-3,4,5-trihydroxy-6-((((2R,3S,4S,5S,6S)-3,4,5-trihydroxy-6-methyltetrahydro-2H-pyran-2-	[M+H]+	595.16998	C27H30O15	Flavonoid-3-O-glycosides	BJJCTXDEJUWVIC-RGLPBSMOSA-N	O=C1C(OC2OC(COC3OC(C)C(O)C(O)C3O)C(O)C(O)C2O)=C(OC=4C=C(O)C=C(O)C14)C=5C=CC=CC5O
1142	3.352	489.13254	Agnuside	[M+H]+	489.13	C22H26O11	Iridoid O-glycosides	GLACGTLACKLUJX-QNAXTHAFSA-N	O=C(OCC1=CC(O)C2C=COC(OC3OC(CO)C(O)C(O)C3O)C12)C4=CC=C(O)C=C4
1143	3.356	457.10947	Naringenin-7-O-beta-D-glucoside	[M+H]+	457.10999	C21H22O10	Flavonoid-7-O-glycosides	DLIKSSGEMUFQOK-SFTVRKLSSA-N	O=C1C2=C(O)C=C(OC3OC(CO)C(O)C(O)C3O)C=C2OC(C4=CC=C(O)C=C4)C1
1144	3.357	455.09698	Riboflavin-5'-monophosphate	[M-H]-	455.09735	C17H21N4O9P	Flavin nucleotides	FVTCRASFADXXNN-SCRDCRAPSA-N	CC1=CC2=C(C=C1C)N(C[C=H](O)[C=H](O)[C=H](O)COP(O)(O)=O)C1=NC(=O)N=C(O)C1=N2
1145	3.36	180.0291	DIBOA	[M-H]-	180.02921	C8H7NO4	Benzoxazinoids	COVOPZQGJGUPEY-UHFFFAOYNA-N	O=C1N(O)C2=CC=CC=C2(OC1(O))
1146	3.362	136.03967	4-AMINOBENZOATE	[M-H]-	136.03999	C7H7NO2	Aminobenzoic acids	ALYNCZNDIQEVRV-UHFFFAOYSA-N	O=C(O)C1=CC=C(N)C=C1
1147	3.368	207.14842	Pesticide3_Isoproturon_C12H18N2O_Arelon	[M+H]+	207.149	C12H18N2O	N-phenylureas	PUIYMUZLKQOUOZ-UHFFFAOYSA-N	O=C(NC1=CC=C(C=C1)C(C)C)N(C)C
1148	3.371	293.10114	_120106	[M+H]+	293.10101	C15H16O6	2-benzopyrans	MMHTXEATDNFMMY-UHFFFAOYSA-N	O=C1OC2(C(=CC(O)C(O)C2)C=3C=C(OC)C=C(O)C13)C
1149	3.375	273.21927	5-beta-Androstan-17-beta-ol-3-one	[M+H]+	273.22128	C19H30O2	Androgens and derivatives	NVKAWKQGWWIWPM-NOLKSFPRSA-N	CC12CCC3C(CC[C=H]4CC(=O)CCC34C)C1CC[C=H]2O
1150	3.375	436.18503	PHYSOSTIGMINE SALICYLATE	[M+H]+	436.18427	C22H27N3O5	Pyrroloindoles	HZOTZTANVBDFOF-PBCQUBLHSA-N	O=C(O)C=1C=CC=CC1O.OC(=NC)OC1=CC=C2C(=C1)C3(C)CCN(C)C3N2C
1151	3.381	250.09113	(2E,4E)-N-(2-methylpropyl)-6-thiophen-2-ylhexa-2,4-dienamide	[M+H]+	250.09003	C14H19NOS	N-acyl amines	KNGBXFMEGLRFHV-PRKJJMSOSA-N	OC(=NCC(C)C)C=CC=CCC=1SC=CC1
1152	3.386	353.16858	7-[(2E,5E)-7-hydroxy-3,7-dimethylocta-2,5-dienoxy]chromen-2-one	[M+H]+	353.1683	C19H22O4	Coumarins and derivatives	ZHQPRJMIAALFHX-ATTWWORZSA-N	O=C1OC=2C=C(OCC=C(C)CC=CC(O)(C)C)C=CC2C=C1
1153	3.388	230.11665	Sebuthylazine	[M+H]+	230.1167	C9H16ClN5	1,3,5-triazine-2,4-diamines	BZRUVKZGXNSXMB-UHFFFAOYNA-N	CCN=C1NC(NC(Cl)=N1)=NC(C)CC
1154	3.397	212.12766	Mescaline	[M+H]+	212.12801	C11H17NO3	Phenethylamines	RHCSKNNOAZULRK-UHFFFAOYSA-N	O(C=1C=C(C=C(OC)C1OC)CCN)C
1155	3.397	187.13246	Royal jelly acid	[M+H]+	187.13	C10H18O3	Medium-chain hydroxy acids and derivatives	QHBZHVUGQROELI-SOFGYWHQSA-N	O=C(O)C=CCCCCCCCO
1156	3.398	425.1954	2-(3,4-dihydroxyphenyl)-5,7-dihydroxy-6,8-dimethoxychromen-4-one	[M+H]+	425.1951	C17H14O8	8-O-methylated flavonoids	GLMWHQIFJIURES-UHFFFAOYSA-N	O=C1C=C(OC=2C(OC)=C(O)C(OC)=C(O)C12)C=3C=CC(O)=C(O)C3
1157	3.398	324.11081	Dehydroevodiamine	[M+H]+	324.11099	C19H15N3O	Quinazolines	VXHNSVKJHXSKKM-UHFFFAOYSA-N	O=C1C=2C=CC=CC2[N+](=C3C=4[N-]C=5C=CC=CC5C4CCN13)C
1158	3.415	188.06987	Desethylatrazine	[M+H]+	188.06975	C6H10ClN5	1,3,5-triazine-2,4-diamines	DFWFIQKMSFGDCQ-UHFFFAOYSA-N	CC(C)N=C1NC(Cl)=NC(=N)N1
1159	3.416	151.039	L-(-)-Mandelic acid	[M-H]-	151.04007	C8H8O3	Benzene and substituted derivatives	IWYDHOAUDWTVEP-SSDOTTSWSA-N	O[C=H](C(O)=O)C1=CC=CC=C1

1160	3.421	226.10651	6-Benzyladenine	[M+H]+	226.10899	C12H11N5	6-alkylaminopurines	NWBJYWHLCVSVIJ-UHFFFAOYSA-N	N=1C=NC=2C1NC=NC2NCC=3C=CC=CC3
1161	3.426	334.17429	convolicine	[M+H]+	334.17001	C18H23NO5	P-methoxybenzoic acids and derivatives	HZEOENYXYFFKIU-QDMKHBRRSA-N	O=C(OC1CC2N(C(=O)C)C(CC2)C1)C3=CC=C(OC)C(OC)=C3
1162	3.427	359.12326	9-methoxy-4,4-dimethyl-13-phenyl-6,14-dioxatetracyclo[8.4.0.0?,?.0?,?]tetradeca-1,7,9-trien-11-one	[M+H]+	359.12537	C21H20O4	Pyranoflavonoids	NGRRXMZEVSBATO-UHFFFAOYSA-N	O=C1C2=C(OC)C=C3OC4C(C3=C2OC(C=5C=CC=CC5)C1)C4(C)C
1163	3.445	154.06151	L-HISTIDINE	[M-H]-	154.062	C6H9N3O2	Histidine and derivatives	HNDVDQJCIGZPNO-UHFFFAOYSA-N	O=C(O)C(N)CC1=CN=CN1
1164	3.45	411.17828	Gomisin J	[M+H]+	411.17801	C22H28O6	Hydrolyzable tannins	PICOUNAPKDEPCA-TXEJJXNPSA-N	OC1=CC2=C(C(OC)=C1OC)C3=C(OC)C(OC)=C(O)C=C3CC(C)C(C)C2
1165	3.45	377.1488	Vincamine	[M+H]+	377.14786	C21H26N2O3	Eburnan-type alkaloids	RXPRRQLKFXBCSJ-GIVPXCGWSA-N	O=C(OC)C1(O)N2C=3C=CC=CC3C4=C2C5N(CC4)CCCC5(CC)C1
1166	3.458	305.12299	(2S,3S)-2-(3,4,5-trihydroxyphenyl)-3,4-dihydro-2H-chromene-3,5,7-triol	[M-H]-	305.12299	C15H14O7	Epigallocatechins	XMOCLSLCDHWDHP-WFASDCNBSA-N	OC=1C=C(O)C2=C(OC(C3=CC(O)=C(O)C(O)=C3)C(O)C2)C1
1167	3.464	179.03427	Caffeic acid	[M-H]-	179.03499	C9H8O4	Hydroxycinnamic acids	QAIPRVGONGVQAS-RQOWECAXSA-N	OC(=O)\C=C/C1=CC(O)=C(O)C=C1
1168	3.472	251.14108	Caffeoyl putrescin (isomer of 1060)	[M+H]+	251.1407	C13H18N2O3	Caffeic acid and derivatives	KTZNZCYTXQYEHT-UHFFFAOYSA-N	O=C(C=CC=1C=CC(O)=C(O)C=1)NCCCCN
1169	3.476	304.16455	scopolamine	[M+H]+	304.16	C17H21NO4	Beta hydroxy acids and derivatives	STECJAGHUSJQJN-KXKVQDCLSA-N	O=C(OC1CC2N(C)C(C1)C3OC32)C(C=4C=CC=CC4)CO
1170	3.48	188.07005	4-hydroxy-4-(pyridin-2-yl)butan-2-one	[M+H]+	188.07001	C9H11NO2	Pyridines and derivatives	NYKHQRHPWDLZTL-UHFFFAOYSA-N	O=C(C)CC(O)C1=NC=CC=C1
1171	3.48	194.11719	Salsoline	[M+H]+	194.11749	C11H15NO2	Tetrahydroisoquinolines	YTPRLBGPGZHUPD-UHFFFAOYSA-N	OC=1C=C2C(=CC1OC)C(NCC2)C
1172	3.481	346.22156	XLR11 N-(4-HYDROXYPENTYL)	[M+H]+	346.21701	C21H28FNO2	N-alkylindoles	MDPXVRYUGDTVIS-UHFFFAOYSA-N	O=C(C1=CN(C=2C=CC=CC12)CCCC(O)CF)C3C(C)(C)C3(C)C
1173	3.483	226.08589	2'-Deoxycytidine	[M-H]-	226.08333	C9H13N3O4	Pyrimidine 2'-deoxyribonucleosides	CKTSBUTUHBMZGZ-UHFFFAOYNA-N	N=C1N=C(O)N(C=C1)C2OC(CO)C(O)C2
1174	3.492	73.06451	ETHYL METHYL KETONE	[M+H]+	73.06479	C4H8O	Ketones	ZWEHNKRNPOVVGH-UHFFFAOYSA-N	CCC(C)=O
1175	3.497	130.08614	N-Methyl-L-proline	[M+H]+	130.08626	C6H11NO2	Proline and derivatives	CWLQUGTUXBXTLF-YFKPBYRVSA-N	CN1CCC[C=H]1C(=O)O
1176	3.498	587.33295	Anthranoyllycoctonine	[M+H]+	587.3327	C32H46N2O8	Alkaloids	NNDHDYDFEDRMGH-UHFFFAOYNA-N	CCN1CC2(COC(=O)C3=C(N)C=CC=C3)CCC(OC)C34C5CC6C(OC)C5C(O)(CC6OC)C(O)(C(OC)C23)C14
1177	3.498	213.16159	Caryophyllene [T(-)]	[M+H]+	213.16136	C14H22	Branched unsaturated hydrocarbons	INOSMXBKABLUIL-CHWSQXEVSA-N	C=C1CC=C(C)CCC2C1CC2(C)C
1178	3.498	441.22687	Deacetylgedunin	[M+H]+	441.22714	C26H32O6	Limonoids	HCEYJYMNIQHPPK-DXTZDJJUSA-N	O=C1OC(C2=COC=C2)C3(C)CCC4C5(C=CC(=O)C(C)(C)C5CC(O)C4(C)C63OC16)C
1179	3.498	371.19586	Splendoline	[M+H]+	371.19699	C21H26N2O4	Strychnos alkaloids	PUJOYKKZYBTUSK-LQOCACJHSA-N	O=C(N1C=2C=CC=CC2C34CCN5CC6C(OCC(C6CC53O)C14)C)CO
1180	3.5	178.04909	ABOA	[M+H]+	178.05051	C9H7NO3	Benzoxazinoids	FZAQRVWPQCXSPC-UHFFFAOYSA-N	O=C1OC2=CC=CC(C(=O)C)=C2(N1)
1181	3.504	159.06555	Pimelic acid	[M-H]-	159.06628	C7H12O4	Medium-chain fatty acids	WLJVNTCWHIRURA-UHFFFAOYSA-N	O=C(O)CCCCCC(=O)O
1182	3.505	154.08597	Dopamine	[M+H]+	154.08626	C8H11NO2	Catecholamines and derivatives	VYFYYTLLBUKUHU-UHFFFAOYSA-N	c1cc(c(cc1CCN)O)O
1183	3.505	297.18936	Exemestane	[M+H]+	297.185	C20H24O2	Androgens and derivatives	BFYIZQONLCFLEV-DAELLWKTSA-N	O=C1C=CC2(C(=C1)C(=C)CC3C2CCC4(C(=O)CCC34)C)C
1184	3.518	374.19846	Hetisine	[M-H]-	374.1973	C20H27NO3	Alkaloids	PIWJSAMCEMZIDO-UHFFFAOYNA-N	CC12CN3C4CC56CC(=C)C7C(O)C5C3C(CC(O)C1)(C24)C6C7O
1185	3.518	356.09747	HMBOA + O-Hex	[M-H]-	356.09811	C15H19NO9	Benzoxazinoids	PMBZSEOAOIYRMW-UHFFFAOYNA-N	O=C2NC3=CC=C(OC)C=C3(OC2(OC1OC(CO)C(O)C(O)C1(O)))
1186	3.523	487.19064	2-[1-[4,5-dihydroxy-3-[(2S,3R,4S,5S,6R)-3,4,5-trihydroxy-6-(hydroxymethyl)oxan-2-yl]oxyoxan-2-yl]indol-3-yl]acetic acid	[M+H]+	487.19223	C21H27NO11	Indole-3-acetic acid derivatives	ISMUGWMKUVABAZ-XRNKWZDJSA-N	O=C(O)CC1=CN(C=2C=CC=CC21)C3OCC(O)C(O)C3OC4OC(CO)C(O)C(O)C4O
1187	3.523	344.25702	Denudatine	[M+H]+	344.26001	C22H33NO2	Napelline-type diterpenoid alkaloids	FIAOWYBSINDVNF-ARXIJESMSA-N	OC1C(=C)C2CC13C4CC5C6(C)CN(CC)C4C5(CCC6)C3CC2O
1188	3.525	468.24429	Penigequinolones A and B (diastereomers)_120177	[M+H]+	468.241	C27H33NO6	Phenylquinolines	CVWJKBJRSZXDIW-IUEPNFFLSA-N	O=C1NC2=CC=C(C=CC3(OCC(C)(C)CC3)C)C(O)=C2C(O)(C4=CC=C(OC)C=C4)C1OC
1189	3.526	298.13885	stepharine	[M+H]+	298.14001	C18H19NO3	Proaporphines	OGJKMZVUJJYWKO-CYBMUJFWSA-N	O=C1C=CC2(C=C1)C=3C(OC)=C(OC)C=C4C3C(NCC4)C2
1190	3.527	213.06482	Harmine	[M+H]+	213.06572	C13H12N2O	Harmala alkaloids	BXNJHAXVSOCGBA-UHFFFAOYSA-N	N=1C=CC=2C=3C=CC(OC)=CC3NC2C1C
1191	3.533	355.14783	Notopterol	[M+H]+	355.14999	C21H22O5	Terpene lactones	BKIACVAZUKISOR-MKMNVTDBSA-N	O=C1OC=2C=C3OC=CC3=C(OCC=C(C)CC(O)C=C(C)C)C2C=C1
1192	3.535	239.14024	CMPF	[M-H]-	239.14258	C12H16O5	Furanoid fatty acids	WMCQWXZMVIETAO-UHFFFAOYSA-N	O=C(O)C1=C(OC(=C1C)CCC)CCC(=O)O
1193	3.536	319.97372	PFSM-sulfonic_acid	[M-H]-	319.96912	C5H8F5NO5S2	PFSA	RNRXXRAIQNUKCT-UHFFFAOYSA-N	O=S(=O)(O)CCCNS(=O)(=O)C(F)(F)C(F)(F)F
1194	3.538	210.07664	Methoxytyrosine	[M-H]-	210.0766	C10H13NO4	Amino acids	PFDUUKDQEHURQC-UHFFFAOYNA-N	O=C(O)C(N)CC1=CC=C(O)C(OC)=C1
1195	3.542	285.1329	Fisetin	[M-H]-	285.13168	C15H10O6	Flavonols	XHEFDIBZLJXQHF-UHFFFAOYSA-N	O=C1C(O)=C(OC2=CC(O)=CC=C12)C=3C=CC(O)=C(O)C3
1196	3.543	289.15344	Indole-3-acetyl-L-isoleucine	[M+H]+	289.15466	C16H20N2O3	Amino acids	WPTUQMUCTTVOFW-UHFFFAOYNA-N	CCC(C)C(N=C(O)CC1=CNC2=CC=CC=C12)C(O)=O
1197	3.543	425.16571	Pentose-Hexose + C5H9	[M-H]-	425.16531	C16H28O10	Terpene glycosides	NYSQQJIJJJAWCE-UHFFFAOYNA-N	OC2COC(OCC1OC(OCC=C(C)C)C(O)C(O)C1(O))C(O)C2(O)
1198	3.545	331.12976	SPECTINOMYCIN	[M-H]-	331.12915	C14H24N2O7	1,4-dioxanes	UNFWWIHTNXNPBV-WXKVUWSESA-N	O=C1CC(OC2OC3C(O)C(NC)C(O)C(NC)C3OC12O)C
1199	3.547	302.10492	Dimethachlor ESA	[M+H]+	302.10568	C13H19NO5S	Anilides	RVSCDWJKJDBFRS-UHFFFAOYSA-N	COCCN(C(=O)CS(O)(=O)=O)C1=C(C)C=CC=C1C
1200	3.55	418.93854	Fipronil sulfide	[M-H]-	418.93652	C12H4Cl2F6N4S	Phenylpyrazoles	FQXWEKADCSXYOC-UHFFFAOYSA-N	C1=C(C=C(C(=C1Cl)N2C(=C(C(C#N)=N2)SC(F)(F)F)N)Cl)C(F)(F)F
1201	3.563	261.14432	Isoleucylglutamate	[M+H]+	261.14429	C11H20N2O5	Dipeptides	KTGFOCFYOZQVRJ-UHFFFAOYNA-N	O=C(O)CCC(NC(=O)C(N)C(C)CC)C(=O)O
1202	3.564	363.11801	(4R,6S,8S,9Z,11E)-16-chloro-17,19-dihydroxy-4-methyl-3,7-dioxatricyclo[13.4.0.0?,?]nonadeca-1(15),9,11,16,18-pentaene-2,13-dione	[M-H]-	363.1167	C18H17ClO6	Hydroxybenzoic acid derivatives	WYZWZEOGROVVHK-RPJJWQTDSA-N	O=C1OC(C)CC2OC2C=CC=CC(=O)CC=3C(Cl)=C(O)C=C(O)C13

1203	3.566	401.23819	Irigenin Trimethyl Ether	[M-H]-	401.23575	C21H22O8	7-O-methylisoflavones	NCEXCGUAXVAJMI-UHFFFAOYSA-N	O=C1C(=COC=2C=C(OC)C(OC)=C(OC)C12)C=3C=C(OC)C(OC)=C(OC)C3
1204	3.57	243.13617	PyroglutamylIsoleucine (isomer of 1006)	[M+H]+	243.13541	C11H18N2O4	Dipeptides	MKCVLTRITQNGQI-UHFFFAOYNA-N	O=C(O)C(NC(=O)C1NC(=O)CC1)C(C)CC
1205	3.58	213.06549	Propyl gallate	[M+H]+	213.07001	C10H12O5	Galloyl esters	ZTHYODDOHIVTJV-UHFFFAOYSA-N	O=C(OCCC)C1=CC(O)=C(O)C(O)=C1
1206	3.582	234.11162	Mecarbinate	[M+H]+	234.112	C13H15NO3	Indolecarboxylic acids and derivatives	YTBNTDMBGXAOCG-UHFFFAOYSA-N	O=C(OCC)C=1C=2C=C(O)C=CC2N(C1C)C
1207	3.583	249.12315	Caffeoyl putrescin (isomer of 391)	[M-H]-	249.1228	C13H18N2O3	Caffeic acid and derivatives	KTZNZCYTXQYEHT-UHFFFAOYSA-N	O=C(C=CC=1C=CC(O)=C(O)C=1)NCCCCN
1208	3.59	371.19052	Glyasperin D	[M+H]+	371.19	C22H26O5	7-O-methylated isoflavonoids	DDMAUIOCNQXFHL-AWEZNQCLSA-N	OC1=CC=C(C(O)=C1)C2COC3=CC(OC)=C(C(OC)=C3C2)CC=C(C)C
1209	3.591	331.17554	(2R,3R,4S,5S,6R)-2-[(2E)-4-hydroxy-3,7-dimethylocta-2,6-dienoxy]-6-(hydroxymethyl)oxane-3,4,5-triol	[M-H]-	331.17621	C16H28O7	Terpene glycosides	PBPYEEMQIFDGSQ-MIYBPCGNSA-N	OCC1OC(OCC=C(C)C(O)CC=C(C)C)C(O)C(O)C1O
1210	3.596	243.16881	2,6-ditert-butyl-4-methylphenol	[M+H]+	243.17	C15H24O	Phenylpropanes	NLZUEZXRPGMBCV-UHFFFAOYSA-N	OC=1C(=CC(=CC1C(C)(C)C)C)C(C)(C)C
1211	3.596	359.17322	methyl (1R,18R)-17-ethyl-3,13-diazapentacyclo[13.3.1.0?,??.0?,?.0??,??]nonadeca-2(10),4(9),5,7,16-	[M+H]+	359.17297	C21H24N2O2	Ibogan-type alkaloids	CMKFQVZJOWHHDV-QYWJTTNJSA-N	O=C(OC)C12C=3NC=4C=CC=CC4C3CCN5CC(C=C(CC)C51)C2
1212	3.609	349.11566	4-[2-[(1R,4aS,5R,6R,8aS)-6-hydroxy-5-(hydroxymethyl)-5,8a-dimethyl-2-methylidene-3,4,4a,6,7,8-hexahydro-1H-naphthalen-1-yl]-1-hydroxyethyl]-2H-	[M-H]-	349.11481	C20H30O5	Diterpene lactones	KHQNSSJNIXVKMK-QSCDNJGLSA-N	O=C1OCC=C1C(O)CC2C(=C)CCC3C(C)(CO)C(O)CCC23C
1213	3.614	469.16434	7-Hydroxymethotrexat	[M-H]-	469.15897	C20H22N8O6	Folic acids	HODZDDDNGRLGSI-UHFFFAOYNA-N	CN(CC=1C(N=C2C(=C(N)NC(=N)N2)N1)=O)C3=CC=C(C=C3)C(NC(CCC(=O)O)C(=O)O)=O
1214	3.618	320.11179	(2R)-5,8-dihydroxy-2-(2-hydroxyphenyl)-7-methoxy-2,3-dihydrochromen-4-one	[M+H]+	320.11285	C16H14O6	7-O-methylated flavonoids	NJUWLUPGDQOJCR-GFCCVEGCSA-N	O=C1C2=C(O)C=C(OC)C(O)=C2OC(C=3C=CC=CC3O)C1
1215	3.619	150.12756	Methamphetamine	[M+H]+	150.12773	C10H15N	Amphetamines and derivatives	MYWUZJCMWCOHBA-UHFFFAOYNA-N	C=1C=CC(=CC1)CC(NC)C
1216	3.62	259.07156	Peucenin	[M-H]-	259.07358	C15H16O4	Chromones	DTNGBIGBPPPNNB-UHFFFAOYSA-N	O=C1C=C(OC2=CC(O)=C(C(O)=C12)CC=C(C)C)C
1217	3.621	498.24292	_130048	[M+H]+	498.246	C28H35NO7	Phenylquinolines	KMVGZHAXSWPDLJ-CHMKTJRNSA-N	O=C1NC2=CC=C(C=CC3(OC(OC)C(C)(C)CC3)C)C(O)=C2C(O)(C4=CC=C(OC)C=C4)C1OC
1218	3.621	492.28992	4-((9S,13R)-10-((E)-((2,2-dimethoxyethyl)imino)methyl)-3,5,14-trihydroxy-13-methylhexadecahydro-1H-cyclopenta[a]phenanthren-17-yl)furan-2(5H)-one	[M+H]+	492.29001	C27H41NO7	Cardenolides and derivatives	XWFDREYZNGAQBP-MDCZLNDTSA-N	O=C1OCC(=C1)C2CCC3(O)C4CCC5(O)CC(O)CCC5(C=NCC(OC)OC)C4CCC23C
1219	3.621	408.18555	methyl (1S)-7-hydroxy-7-methyl-1-[(2S,3R,4S,5S,6R)-3,4,5-trihydroxy-6-(hydroxymethyl)oxan-2-yl]oxy-4a,5,6,7a-tetrahydro-1H-cyclopenta[c]pyran-4-	[M+H]+	408.1864	C17H26O10	Iridoid O-glycosides	XBGJTRDIWPEIMG-YVAUHRMASA-N	O=C(OC)C1=COC(OC2OC(CO)C(O)C(O)C2O)C3C1CCC3(O)C
1220	3.621	177.06541	Pemoline	[M+H]+	177.06586	C9H8N2O2	Benzene and substituted derivatives	NRNCYVBFPDDJNE-UHFFFAOYNA-N	N=C1N=C(O)C(O1)C=2C=CC=CC2
1221	3.623	441.2225	methyl (4R,8aS)-1-hydroxy-2-(hydroxymethyl)-5,5,8a-trimethyl-4-[(2E,4E,6E)-octa-2,4,6-trienoyl]oxy-4a,6,7,8-tetrahydro-4H-naphthalene-1-	[M+H]+	441.22473	C24H34O6	Fatty acid esters	QIDMCIFFMHMTBT-YKBPFZMUSA-N	O=C(OC1C=C(CO)C(O)(C(=O)OC)C2(C)CCCC(C)(C)C12)C=CC=CC=CC
1222	3.625	521.25934	Okaramine H_120150	[M+H]+	521.258	C32H32N4O3	Pyrroloindoles	CNTMYVFPLVDMFY-HIVUMZBNSA-N	O=C1C2=CC=3C=4C=CC=CC4NC3C(C=CN2C(=O)C5N1C6NC=7C(=CC=CC7C6(O)C5)CC=C(C)C)(C)C
1223	3.629	323.12402	Flavanone base + 2O, 1Prenyl	[M-H]-	323.12741	C20H20O4	Prenylated chalcones	KYFBXCHUXFKMGQ-UHFFFAOYNA-N	O=C2C=3C=CC(O)=C(C=3(OC(C1=CC=C(O)C=C1)C2))CC=C(C)C
1224	3.632	136.02103	Benzothiazole	[M+H]+	136.02155	C7H5NS	Benzothiazoles	IOJUPLGTWVMSFF-UHFFFAOYSA-N	C1=CC=C2C(=C1)N=CS2
1225	3.634	563.23291	5,7-dihydroxy-2-(4-hydroxyphenyl)-6-[3,4,5-trihydroxy-6-(hydroxymethyl)oxan-2-yl]-8-(3,4,5-trihydroxyoxan-2-yl)chromen-4-one	[M-H]-	563.22931	C26H28O14	Flavonoid 8-C-glycosides	MMDUKUSNQNWVET-UHFFFAOYSA-N	O=C1C=C(OC2=C1C(O)=C(C(O)=C2C3OCC(O)C(O)C3O)C4OC(CO)C(O)C(O)C4O)C=5C=CC(O)=CC5
1226	3.634	517.22675	cephalochromin	[M-H]-	517.22937	C28H22O10	Naphthopyranones	JGQBYBXYRUCBQY-UHFFFAOYSA-N	O=C1C=2C(O)=C3C(O)=CC(O)=C(C3=CC2OC(C)C1)C4=C(O)C=C(O)C=5C(O)=C6C(=O)CC(OC6=CC54)C
1227	3.635	635.16156	9-methoxy-7-[4-[3,4,5-trihydroxy-6-[[3,4,5-trihydroxy-6-(hydroxymethyl)oxan-2-yl]oxymethyl]oxan-2-yl]oxyphenyl]-[1,3]dioxolo[4,5-	[M-H]-	635.16174	C29H32O16	Isoflavonoid O-glycosides	FIMKKXUVGBDUNE-UHFFFAOYSA-N	O=C1C(=COC=2C=C3OCOC3=C(OC)C12)C=4C=CC(OC5OC(COC6OC(CO)C(O)C(O)C6O)C(O)C(O)C5O)=CC4
1228	3.635	553.20477	Selaginpulvilin N	[M-H]-	553.20203	C37H30O5	Fluorenes	SGAVFJAMHHIGIH-UHFFFAOYSA-N	OC1=CC=C(C#CC=2C(=CC=C3C=4C=CC(O)=CC4C(C5=CC=C(OC)C=C5)(C6=CC=C(OC)C=C6)C23)COC)C=C1
1229	3.637	325.13812	Quinoxalinedione + 2Methyl + C5H11O4	[M+H]+	325.1391	C15H20N2O6	Others	SLOQSNHEHSCLGS-UHFFFAOYNA-N	O=C2NC=1C=C(C(=CC=1N(C2(=O))CC(O)C(O)C(O)CO)C)C
1230	3.64	303.18042	2-Methylene-5-(2,5-Dioxotetrahydrofuran-3-Yl)-6-Oxo--10,10-Dimethylbicyclo[7: 2: 0]Undecane	[M-H]-	303.1864	C18H24O4	Dicarboxylic acids and derivatives	HIFLEGNWVFLGLY-UHFFFAOYSA-N	O=C1OC(=O)C(C1)C2C(=O)CCC3C(C(=C)CC2)CC3(C)C
1231	3.64	495.16333	3,4-di-O-galloylquinic acid	[M-H]-	495.16208	C21H20O14	Quinic acids and derivatives	SKUCQDOSGKINGP-YQMRLJPGSA-N	O=C(O)C1(O)CC(O)C(OC(=O)C2=CC(O)=C(O)C(O)=C2)C(OC(=O)C3=CC(O)=C(O)C(O)=C3)C1
1232	3.64	437.27539	4-hydroxy-7-methyl-2-[2-[3,4,5-trihydroxy-6-(hydroxymethyl)oxan-2-yl]oxypropan-2-yl]-2,3-dihydrofuro[3,2-g]chromen-5-one	[M-H]-	437.26904	C21H26O10	Furanochromones	KHWKLNXMSVVKCH-UHFFFAOYSA-N	O=C1C=C(OC=2C=C3OC(CC3=C(O)C12)C(OC4OC(CO)C(O)C(O)C4O)(C)C)C
1233	3.642	287.17017	(1S,2R,9R,10S,12S)-12-hydroxy-7,15-diazatetracyclo[7.7.1.0^{2,7}.0^{10,15}]heptadecan-6-one	[M+H]+	287.17001	C15H24N2O2	Sparteine, lupanine, and related alkaloids	JVYKIBAJVKEZSQ-VJTDZRGJSA-N	O=C1N2CC3CC(CN4CCC(O)CC43)C2CCC1
1234	3.643	271.16718	Sophoridine	[M+H]+	271.17001	C15H24N2O	Matrine alkaloids	ZSBXGIUJOOQZMP-BHPKHCPMSA-N	O=C1N2CC3CCCN4CCCC(C2CCC1)C43
1235	3.644	279.09811	15,16-Dihydrotanshinone I	[M+2H]2+	279.10001	C18H14O3	Tanshinones, isotanshinones, and	HARGZZNYNSYSGJ-JTQLQIEISA-N	O=C1C(=O)C2=C(OCC2C)C=3C=CC4=C(C=CC=C4C)C13
1236	3.646	373.14172	[(1S,3aR,5R,5aR,8aR,9S,9aR)-1,5,8a-trimethyl-2,8-dioxo-3a,4,5,5a,9,9a-hexahydro-1H-azuleno[6,5-b]furan-9-yl] 2-methylpropanoate	[M+H]+	373.14117	C19H26O5	Sesquiterpene lactones	WKUOPGZYLRFCHJ-APDQSUQKSA-N	O=C(OC1C2C(OC(=O)C2C)CC(C)C3C=CC(=O)C13C)C(C)C
1237	3.649	269.11221	ellipticine	[M+H]+	269.10999	C17H14N2	Carbazoles	CTSPAMFJBXKSOY-UHFFFAOYSA-N	N=1C=CC=2C(C1)=C(C=3C=4C=CC=CC4NC3C2C)C
1238	3.655	308.1514	Tebuconazole	[M+H]+	308.1524	C16H22ClN3O	Phenylbutylamines	PXMNMQRDXWABCY-UHFFFAOYNA-N	CC(C)(C)C(O)(CCC1=CC=C(C1)C=C1)CN1C=NC=N1
1239	3.667	289.15353	Indole-3-acetyl-L-leucine	[M+H]+	289.15466	C16H20N2O3	Leucine and derivatives	HCZNPUHZYPPINM-UHFFFAOYNA-N	CC(C)CC(N=C(O)CC1=CNC2=CC=CC=C12)C(O)=O
1240	3.669	213.1041	methyl 1-methyl-9H-beta-carbolin-7-yl ether	[M+H]+	213.105	C13H12N2O	Harmala alkaloids	BXNJHAXVSOCGBA-UHFFFAOYSA-N	N=1C=CC=2C=3C=CC(OC)=CC3NC2C1C
1241	3.669	296.14151	Pethoxamide	[M+H]+	296.14117	C16H22ClNO2	Benzene and substituted derivatives	CSWIKHNSBZVWNQ-UHFFFAOYSA-N	CCOCCN(C(=O)CCl)C(=C(C)C)C1=CC=CC=C1
1242	3.669	310.13135	Zacopride	[M+H]+	310.13168	C15H20ClN3O2	Aminophenyl ethers	FEROPKNOYKURCJ-UHFFFAOYNA-N	ClC1=CC(C(O)=NC2CN3CCC2CC3)=C(OC)C=C1N
1243	3.671	525.17126	(5Z)-4-[2-[2-(3,4-dihydroxyphenyl)ethoxy]-2-oxoethyl]-5-ethylidene-6-[(2S,3R,4S,5S,6R)-3,4,5-trihydroxy-6-(hydroxymethyl)oxan-2-yl]oxy-4H-	[M-H]-	525.17108	C24H30O13	Terpene glycosides	HKVGJQVJNQRJPO-GSDSTAPDSA-N	O=C(O)C1=COC(OC2OC(CO)C(O)C(O)C2O)C(=CC)C1CC(=O)OCCC3=CC=C(O)C(O)=C3
1244	3.671	339.11002	2-morpholino-7H-naphtho[1,2,3-de]quinolin-7-one	[M+H]+	339.10999	C20H16N2O2	Anthracenes	BUWARJBQHWMPTL-UHFFFAOYSA-N	O=C1C=2C=CC=CC2C3=CC(=NC4=CC=CC1=C43)N5CCOCC5
1245	3.694	375.13251	Geniposidic acid	[M+H]+	375.13	C16H22O10	Iridoid O-glycosides	ZJDOESGVOWAULF-OGJQONSISA-N	O=C(O)C1=COC(OC2OC(CO)C(O)C(O)C2O)C3C(=CCC13)CO

1246	3.697	584.33673	Shearinine F_120146	[M+H]+	584.33899	C37H45NO5	Naphthopyrans	QYRAHJVRFPJIQW-MHXJJHONSA-N	O=C1C=C2C3(OC1C(O3)(C)C)CCC4(C)C2(O)CCC5CC=6C=7C=C8C(=CC7NC6C54C)C9=C(C8)C(OC(C)(C)C9)(C)C
1247	3.697	359.16888	tabersonine	[M+H]+	359.17001	C21H24N2O2	Plumeran-type alkaloids	FNGGIPWAZSFKCN-HBMCJLEFSA-N	O=C(OC)C1=C2NC=3C=CC=CC3C24CCN5CC=CC(C1)(CC)C54
1248	3.708	265.08093	(S)-3-(4-hydroxyphenyl)chroman-7-ol	[M+H]+	265.07999	C15H14O3	Isoflavanols	ADFCQWZHKCXPAJ-GFCCVEGCSA-N	OC1=CC=C(C=C1)C2COC3=CC(O)=CC=C3C2
1249	3.71	439.29025	Ochrolifuanine A	[M+H]+	439.28601	C29H34N4	Harmala alkaloids	BYHWAEAVIGYEBJ-QJTMEEEXSA-N	C=1C=CC2=C(C1)NC3=C2CCNC3CC4CC5C=6NC=7C=CC=CC7C6CCN5CC4CC
1250	3.711	221.091	2-amino-3-(5-hydroxy-1H-indol-3-yl)propanoic acid	[M+H]+	221.09	C19H22N2O	Eburnan-type alkaloids	WYJAPUKIYAZSEM-MOPGFXCFSA-N	O=C1N2C=3C=CC=CC3C4=C2C5N(CC4)CCCC5(C1)CC
1251	3.719	383.16577	Isolaserpitin	[M+H]+	383.16812	C19H20O6	Angular pyranocoumarins	KJWFOHVSTFGWGZ-YHYXMXQVSA-N	O=C1OC2=C(C=C1)C=CC=3OC(C)(C)C(OC(=O)C(=CC)C)C(O)C32
1252	3.719	405.18011	reserpinine	[M+H]+	405.17999	C22H26N2O4	Yohimbine alkaloids	KXEMQEGRZWUKJS-RXDRHKEWSA-N	O=C(OC)C1=COC(C)C2CN3CCC=4C=5C=CC(OC)=CC5NC4C3CC12
1253	3.72	183.09167	Harman	[M+H]+	183.09167	C12H10N2	Harmala alkaloids	PSFDQSOCUJVVGF-UHFFFAOYSA-N	CC1=C2NC3=CC=CC=C3C2=CC=N1
1254	3.734	227.07654	aspernigrin A	[M-H]-	227.0789	C13H12N2O2	Nicotinamides	RUUZDCZRFRLJFC-UHFFFAOYSA-N	O=C1C=C(NC=C1C(=N)O)CC=2C=CC=CC2
1255	3.735	106.06487	3-Methylthiopropylamine	[M+H]+	106.0685	C4H11NS	Dialkylthioethers	KKYSBGWCYXYOHA-UHFFFAOYSA-N	S(C)CCCN
1256	3.736	593.15002	Apigenin-6-C-glucoside-7-O-glucoside	[M-H]-	593.15118	C27H30O15	Flavone C,O-glycosides	HGUVPEBGCAVWID-UHFFFAOYNA-N	O=C2C=C(OC=3C=C(OC1OC(CO)C(O)C(O)C1(O))C(=C(O)C2=3)C4OC(CO)C(O)C(O)C4(O))C5=CC=C(O)C=C5
1257	3.744	152.07027	Acetaminophen	[M+H]+	152.0706	C8H9NO2	1-hydroxy-2-unsubstituted benzenoids	RZVAJINKPMORJF-UHFFFAOYSA-N	CC(=Nc1ccc(cc1)O)O
1258	3.746	323.14667	1-[4,5-bis(hydroxymethyl)-3-methoxy-2-methylphenoxy]-3-methylbutane-2,3-diol	[M+H]+	323.14648	C15H24O6	Benzyl alcohols	MAQMWSUGBCIGLJ-UHFFFAOYSA-N	OCC1=CC(OCC(O)C(O)(C)C)=C(C(OC)=C1CO)C
1259	3.746	584.34082	Shearinine A_120147	[M+H]+	584.34003	C37H45NO5	Naphthopyrans	YAGQIZPAJNEIKG-KBCUUGKHSA-N	O=C1C=C2C3(OC1C(O3)(C)C)CCC4(C)C2(O)CCC5CC=6C7=CC8=C(C=C7NC6C54C)C9=CC(OC(C)(C)C9C8)(C)C
1260	3.748	421.24219	(1,9,9b-trihydroxy-6,6,9a-trimethyl-3,5,5a,7,8,9-hexahydro-1H-benzo[e][2]benzofuran-5-yl) hexanoate	[M+H]+	421.24319	C21H34O6	Naphthofurans	GFMGOVRWOOAPMN-UHFFFAOYSA-N	O=C(OC1C=C2COC(O)C2(O)C3(C)C(O)CCC(C)(C)C13)CCCCC
1261	3.748	317.15945	(2S,3S)-2-(3,4-dihydroxyphenyl)-3,5,7-trihydroxy-6-methyl-2,3-dihydrochromen-4-one	[M-H]-	317.16092	C16H14O7	Flavanonols	KPCWWZLBHGSXPW-CVEARBPZSA-N	O=C1C2=C(O)C(=C(O)C=C2OC(C3=CC=C(O)C(O)=C3)C1O)C
1262	3.754	310.12711	Ketotifen	[M+H]+	310.12601	C19H19NOS	Cycloheptathiophenes	ZCVMWBYGMWKGHF-UHFFFAOYSA-N	O=C1C=2SC=CC2C(C=3C=CC=CC3C1)=C4CCN(C)CC4
1263	3.756	565.15277	methyl (2S,3R,4S)-3-ethenyl-4-[2-(3,4,5-trihydroxybenzoyl)oxyethyl]-2-[3,4,5-trihydroxy-6-(hydroxymethyl)oxan-2-yl]oxy-3,4-dihydro-2H-pyran-5-	[M+H]+	565.15277	C24H30O14	Terpene glycosides	SMTKSCGLXONVGL-FMDXMRPMSA-N	O=C(OC)C1=COC(OC2OC(CO)C(O)C(O)C2O)C(C=C)C1CCOC(=O)C3=CC(O)=C(O)C(O)=C3
1264	3.758	373.24261	11A-Acetoxyprogesterone	[M+H]+	373.23734	C23H32O4	Gluco/mineralocorticoids, progestogins and	IWRPVTXREVYBHT-QHXXRISESA-N	O=C(OC1CC2(C)C(C(=O)C)CCC2C3CCC4=CC(=O)CCC4(C)C13)C
1265	3.758	192.06592	Actrarit	[M-H]-	192.06662	C10H11NO3	Benzene and substituted derivatives	MROJXXOCABQVEF-UHFFFAOYSA-N	O=C(O)CC1=CC=C(N=C(O)C)C=C1
1266	3.759	355.10211	Feruloyl Hexoside (isomer of 847)	[M-H]-	355.1022	C16H20O9	Ferulic acid and derivatives	JWRQVQWBNRGGPK-UHFFFAOYNA-N	O=C(OC1OC(CO)C(O)C(O)C1(O))C=CC=2C=CC(O)=C(OC)C=2
1267	3.76	358.24216	diacetylsongorine	[M+H]+	358.23999	C22H31NO3	Napelline-type diterpenoid alkaloids	CBOSLVQFGANWTL-DVPYZRQCSA-N	O=C1CC2C3(CC1C(=C)C3O)C4CC5C6(C)CN(CC)C4C25C(O)CC6
1268	3.763	147.04362	Coumaric acid (isomer of 189, 194)	[M+H-H2O]+	147.0443	C9H8O3	Coumaric acid and derivatives	NGSWKAQJJWESNS-UHFFFAOYSA-N	O=C(O)C=CC1=CC=C(O)C=C1
1269	3.763	335.15857	triptophenolide	[M+H]+	335.16	C20H24O3	Oxosteroids	KPXIBWGPZSPABK-PXNSSMCTSA-N	O=C1OCC2=C1CCC3(C4=CC=C(C(O)=C4CCC23)C(C)C)C
1270	3.773	313.11658	5,7-dimethoxy-3-(4-methoxyphenyl)-4H-chromen-4-one	[M+H]+	313.10999	C18H16O5	7-O-methylisoflavones	PVVORTURQPBPEQ-UHFFFAOYSA-N	O=C1C(=COC2=CC(OC)=CC(OC)=C21)C=3C=CC(OC)=CC3
1271	3.778	399.18561	4-hydroxy-9-(4-hydroxy-3,5-dimethoxyphenyl)-5a,6,8a,9-tetrahydro-5H-[2]benzofuro[5,6-f][1,3]benzodioxol-8-one	[M-H]-	399.1853	C21H20O8	Lignan lactones	JGGWNGRBXJWAOC-UHFFFAOYSA-N	O=C1OCC2CC3=C(O)C=4OCOC4C=C3C(C5=CC(OC)=C(O)C(OC)=C5)C12
1272	3.781	241.09642	Cyanazine	[M+H]+	241.0963	C9H13ClN6	Aminotriazines	MZZBPDKVEFVLFF-UHFFFAOYSA-N	CCN=C1NC(NC(C)(C)C#N)=NC(Cl)=N1
1273	3.794	593.15302	2-(3,4-dihydroxyphenyl)-5-hydroxy-7-[(2S,3R,4S,5S,6R)-3,4,5-trihydroxy-6-[[(2R,3R,4R,5R,6S)-3,4,5-trihydroxy-6-methyloxan-2-yl]oxymethyl]oxan-2-	[M-H]-	593.1524	C27H30O15	Flavonoid-7-O-glycosides	MGYBYJXAXUBTQF-FOBVWLSUSA-N	O=C1C=C(OC2=CC(OC3OC(COC4OC(C)C(O)C(O)C4O)C(O)C(O)C3O)=CC(O)=C12)C=5C=CC(O)=C(O)C5
1274	3.797	432.27969	veratramine	[M+H]+	432.28	C27H39NO2	Fluorenes	MALFODICFSIXPO-AZAJSESZSA-N	OC1CC2=CCC3C4=CC=C(C(=C4CC3C2(C)CC1)C)C(C)C5NCC(C)CC5O
1275	3.814	373.16257	4-[2-(2,6-dimethoxy-4-prop-2-enylphenoxy)-1-hydroxypropyl]-2-methoxyphenol	[M-H]-	373.16565	C21H26O6	Lignans, neolignans and related compounds	ULZFTGWWPHYLGI-UHFFFAOYSA-N	OC1=CC=C(C=C1OC)C(O)C(OC2=C(OC)C=C(C=C2OC)CC=C)C
1276	3.815	374.22787	SH373-1	[M+H]+	374.229	C17H31N3O6	Peptides	UKFUHWPKKCYCQR-UHFFFAOYSA-N	O=C(O)C(N=C(O)C(N=C(O)CCC(N)C(=O)O)C(C)CC)CC(C)C
1277	3.815	324.11893	Tetrahydrocoptisine	[M+H]+	324.12	C19H17NO4	Protoberberine alkaloids and derivatives	UXYJCYXWJGAKQY-OAHLLOKOSA-N	O1C2=CC=C3C(=C2OC1)CN4CCC5=CC=6OCOC6C=C5C4C3
1278	3.821	226.14308	Terbutaline	[M+H]+	226.14377	C12H19NO3	Resorcinols	XWTYSIMOBUGWOL-UHFFFAOYNA-N	CC(C)(C)NCC(O)C1=CC(O)=CC(O)=C1
1279	3.823	504.29684	andrastin A	[M+H]+	504.29556	C28H38O7	Steroid esters	GRBXNADBNJGZRK-GJEDHNSHSA-N	O=CC12CCC(OC(=O)C)C(C)(C)C2CCC3(C)C1C=C(C)C4(C(=O)C(C(=O)C43C(=O)OC)C)C
1280	3.823	362.21054	Octocrylene	[M+H]+	362.21146	C24H27NO2	Diphenylmethanes	FMJSMJQBSVNSBF-UHFFFAOYNA-N	CCCCC(CC)COC(C(C#N)=C(C1=CC=CC=C1)C2=CC=CC=C2)=O
1281	3.823	223.15576	Tetraglyme	[M+H]+	223.15401	C10H22O5	Dialkyl ethers	ZUHZGEOKBKGPSW-UHFFFAOYSA-N	COCCOCCOCCOCCOC
1282	3.824	506.23178	3,15-dibenzyl-1,4,12-trimethyl-1,4,7,10,13-pentazacyclopentadecane-2,5,8,11,14-pentone	[M-H]-	506.23819	C27H33N5O5	Macrolactams	MSIMWLAIWYFFLY-UHFFFAOYSA-N	O=C1N(C)C(C(=O)N(C)C(C(O)=NC(C(O)=NCC(O)=NC1)C)CC=2C=CC=CC2)CC=3C=CC=CC3
1283	3.826	384.17468	Quetiapine	[M+H]+	384.17401	C21H25N3O2S	Dibenzothiazepines	URKOMYMAXPYINW-UHFFFAOYSA-N	OCCOCCN1CCN(C2=NC=3C=CC=CC3SC=4C=CC=CC42)CC1
1284	3.827	300.14331	Metoclopramide	[M+H]+	300.14734	C14H22ClN3O2	Aminophenyl ethers	TTWJBBZEZQICBI-UHFFFAOYSA-N	CCN(CC)CCN=C(O)C1=C(OC)C=C(N)C(Cl)=C1
1285	3.827	246.11154	resveratrol	[M+H]+	246.11246	C14H12O3	Stilbenes	LUKBXSAWLPMMSZ-OWOJBTEDSA-N	OC1=CC=C(C=C1)C=CC=2C=C(O)C=C(O)C2
1286	3.828	121.02873	2-Hydroxybenzaldehyde	[M-H]-	121.0295	C7H6O2	Hydroxybenzaldehydes	SMQUZDBALVYZAC-UHFFFAOYSA-N	O=CC=1C=CC=CC1O
1287	3.834	299.12369	angoletin	[M-H]-	299.12888	C18H20O4	2'-Hydroxy-dihydrochalcones	HBRYKWADRULLHU-UHFFFAOYSA-N	O=C(C=1C(O)=C(C(O)=C(C1OC)C)C)CCC=2C=CC=CC2
1288	3.838	281.03995	Pseudobaptigenin	[M-H]-	281.04553	C16H10O5	Isoflavones	KNJNBKINYHZUGC-UHFFFAOYSA-N	OC1=CC=C2C(=O)C(=COC2=C1)C1=CC2=C(OCO2)C=C1

1289	3.839	335.11554	(E)-3-hydroxy-6-(4-hydroxy-6-methoxy-7-methyl-3-oxo-1H-2-benzofuran-5-yl)-4-methylhex-4-enoic acid	[M-H]-	335.11362	C17H20O7	Phthalides	OGJCZLOIKMTMKF-XBXARRHUSA-N	O=C1OCC2=C1C(O)=C(C(OC)=C2C)CC=C(C)C(O)CC(=O)O
1290	3.84	364.14859	(+)-Corypalmine	[M+H]+	364.14999	C20H23NO4	Protoberberine alkaloids and derivatives	BMCZTYDZHNTKPR-MRXNPFEDSA-N	OC=1C=C2C(=CC1OC)C3N(CC4=C(OC)C(OC)=CC=C4C3)CC2
1291	3.84	123.04374	3-Hydroxybenzaldehyde	[M+H]+	123.04405	C7H6O2	Hydroxybenzaldehydes	IAVREABSGIHHMO-UHFFFAOYSA-N	O=CC=1C=CC=C(O)C1
1292	3.845	372.04974	Gluconapin (3-butenylglucosinolate)	[M-H]-	372.05542	C26H44O9	Medium-chain fatty acids	MINDHVHHQZYEEK-HBBNESRFSA-N	O=C(O)CCCCCCCCOC(=O)C=C(C)CC1OCC(CC2OC2C(C)C(O)C)C(O)C1O
1293	3.849	303.19116	Hesperetin	[M+H]+	303.19211	C16H14O6	4'-O-methylated flavonoids	AIONOLUJZLIMTK-AWEZNQCLSA-N	O=C1C=2C(O)=CC(O)=CC2OC(C3=CC=C(OC)C(O)=C3)C1
1294	3.855	322.10635	methyl 1-oxo-2-(o-tolyl)-1,2,3,6,7,7a-hexahydro-3a,6-epoxyisoindole-7-carboxylate	[M+H]+	322.10501	C17H17NO4	Phenylpyrrolidines	NDOPUHIEVZKCTM-UHFFFAOYSA-N	O=C(OC)C1C2OC3(C=C2)CN(C(=O)C13)C=4C=CC=CC4C
1295	3.865	129.05515	Ketoleucine	[M-H]-	129.05573	C6H10O3	Short-chain keto acids and derivatives	BKAJNAXTPSGJCU-UHFFFAOYSA-N	O=C(O)C(=O)CC(C)C
1296	3.866	506.26962	(2E,6E,11E)-18-(2,6-dioxopiperidin-4-yl)-9,13-dihydroxy-8-methoxy-10,12,14-trimethyl-15-oxooctadeca-2,6,11-trienoic acid	[M-H]-	506.27161	C27H41NO8	Lineolic acids and derivatives	HJCZOTBHYAPUHT-XHOLQODBSA-N	O=C(O)C=CCCC=CC(OC)C(O)C(C=C(C)C(O)C(C(=O)CCCC1CC(=O)N=C(O)C1)C)C
1297	3.866	434.1893	(7,8-dimethoxy-2,2,3-trimethyl-2,3-dihydro-1H-benzo[d]azepin-4-yl)(3,4-dimethoxyphenyl)methanone	[M+H]+	434.19	C24H29NO5	Benzazepines	IINUIABWYMTUJM-UHFFFAOYSA-N	O=C(C1=CC=C(OC)C(OC)=C1)C2=CC=3C=C(OC)C(OC)=CC3CC(N2C)(C)C
1298	3.869	241.11798	Pyroglutamyl-Isoleucine	[M-H]-	241.11909	C11H18N2O4	Peptides	MKCVLTRITQNGQI-UHFFFAOYNA-N	O=C(O)C(NC(=O)C1NC(=O)CC1)C(C)CC
1299	3.881	283.12076	verruculotoxin	[M+H]+	283.12073	C15H20N2O	Alpha amino acid amides	CUANCTHYEDWUMU-KBPBESRZSA-N	OC1=NC(CC=2C=CC=CC2)CN3CCCCC13
1300	3.885	197.15053	(2E)-3,7-dimethyl-2,6-octadienyl acetate	[M+H]+	197.14999	C12H20O2	Fatty alcohol esters	HIGQPQRQIQDZMP-DHZHZOJOSA-N	O=C(OCC=C(C)CCC=C(C)C)C
1301	3.885	508.28674	deltaline	[M+H]+	508.29001	C27H41NO8	Aconitane-type diterpenoid alkaloids	DTTPWCNKTMQMTE-TYNNPWLESA-N	O=C(OC1C2C3(C)CN(CC)C4C51OCOC65CC(OC)C7CC(O)(C6C7OC)C42C(OC)CC3)C
1302	3.885	541.17786	gossypol	[M+H]+	541.17999	C30H30O8	Sesquiterpenoids	QBKSWRVVCFFDOT-UHFFFAOYSA-N	O=CC1=C(O)C(O)=C(C2=CC(=C(C(O)=C12)C3=C(O)C4=C(C=O)C(O)=C(O)C(=C4C=C3C)C(C)C)C)C(C)C
1303	3.885	300.1803	Rinderine	[M+H]+	300.18054	C15H25NO5	Alkaloids	SFVVQRJOGUKCEG-UHFFFAOYNA-N	CC(C)C(O)(C(C)O)C(=O)OCC1=CCN2CCC(O)C12
1304	3.887	363.17471	[5-acetyloxy-3-(hydroxymethyl)-2-oxo-6-propan-2-ylcyclohex-3-en-1-yl] 3-methylpentanoate	[M+H]+	363.1778	C18H28O6	Menthane monoterpenoids	MPXXSICHRNCHHK-UHFFFAOYSA-N	O=C(OC1C=C(C(=O)C(OC(=O)CC(C)CC)C1C(C)C)CO)C
1305	3.892	492.25519	Imatinib	[M-H]-	492.25174	C29H31N7O	Benzanilides	KTUFNOKKBVMGRW-UHFFFAOYSA-N	O=C(NC1=CC=C(C(=C1)NC=2N=CC=C(N2)C3=CN=CC=C3)C)C4=CC=C(C=C4)CN5CCN(C)CC5
1306	3.897	311.10065	(3S,3'R)-3'-(3-hydroxyphenyl)-4-methylspiro[1H-1,4-benzodiazepine-3,2'-oxirane]-2,5-dione	[M+H]+	311.10263	C17H14N2O4	1,4-benzodiazepines	BDDNYDPRCCDQQJ-PBHICJAKSA-N	O=C1C=2C=CC=CC2N=C(O)C3(OC3C=4C=CC=C(O)C4)N1C
1307	3.9	282.16809	judaicin (eudesmane naphthofuran)	[M+H]+	282.16998	C15H20O4	Eudesmanolides, secoeudesmanolides, and	NGPDZEACIWDCKX-WUDKWMPASA-N	O=C1OC2C(CCC3(C(=O)C=CC(O)(C)C23)C)C1C
1308	3.901	370.17041	N-methylglaucine	[M+H]+	370.17001	[C22H28NO4]+	Aporphines	WKHHFWJJIRCXHA-INIZCTEOSA-N	O(C=1C=C2C=3C(OC)=C(OC)C=C4C3C(CC2=CC1OC)[N+](C)(C)CC4)C
1309	3.901	215.05664	Pesticide3_Monolinuron_C9H11ClN2O2_Urea, N'-(4-chlorophenyl)-N-methoxy-N-methyl-	[M+H]+	215.058	C9H11ClN2O2	N-phenylureas	LKJPSUCKSLORMF-UHFFFAOYSA-N	O=C(NC1=CC=C(Cl)C=C1)N(OC)C
1310	3.901	265.09973	Peucedanol	[M+H]+	265.10001	C14H16O5	7-hydroxycoumarins	WRTWKAQFZYXAEJ-GFCCVEGCSA-N	O=C1OC=2C=C(O)C(=CC2C=C1)CC(O)C(O)(C)C
1311	3.909	256.11725	3,4,5-trimethoxycinnamic acid	[M+H]+	256.11795	C12H14O5	Coumaric acids and derivatives	YTFVRYKNXDADBI-SNAWJCMRSA-N	O=C(O)C=CC1=CC(OC)=C(OC)C(OC)=C1
1312	3.909	389.21622	icariside B5	[M+H]+	389.216	C19H32O8	Fatty acyl glycosides of mono- and disaccharides	CZYPGTRKJFYXLT-SUHXYMONSA-N	O=C1C=C(C)C(O)(CCC(OC2OC(CO)C(O)C(O)C2O)C)C(C)(C)C1
1313	3.913	290.14923	Antibiotic TAN 1169B	[M+H]+	290.14999	C15H19N3O3	Pyrroloindoles	OXGGYEZTRLRHHP-UHFFFAOYSA-N	O=C(OC1=CC=C2NC3N(C(=O)C)CCC3(C2=C1)C)NC
1314	3.917	287.07208	licochalcone B	[M+H]+	287.07257	C16H14O5	Retrochalcones	DRDRYGIIYOPBBZ-XBXARRHUSA-N	O=C(C=CC1=CC=C(O)C(O)=C1OC)C2=CC=C(O)C=C2
1315	3.918	374.14645	Gatifloxacin	[M-H]-	374.14301	C19H22FN3O4	Quinoline carboxylic acids	XUBOMFCQGDBHNK-UHFFFAOYSA-N	O=C(O)C1=CN(C2=C(OC)C(=C(F)C=C2C1=O)N3CCNC(C)C3)C4CC4
1316	3.924	359.22754	(Z)-5-[(1S,2R,4aR,8aR)-5-(hydroxymethyl)-1,2,4a-trimethyl-2,3,4,7,8,8a-hexahydronaphthalen-1-yl]-3-(hydroxymethyl)pent-2-enoic acid	[M+H]+	359.22626	C20H32O4	Colensane and clerodane diterpenoids	LRLROPFPFABQRF-CBYQQGIISA-N	O=C(O)C=C(CO)CCC1(C)C(C)CCC2(C(=CCCC21)CO)C
1317	3.939	314.2077	(2E,4E)-N-[2-(4-hydroxyphenyl)ethyl]dodeca-2,4-dienamide	[M-H]-	314.21255	C20H29NO2	1-hydroxy-2-unsubstituted benzenoids	VVRNYAJXAUQHEN-BNFZFUHLSA-N	OC(=NCCC1=CC=C(O)C=C1)C=CC=CCCCCCCC
1318	3.94	179.03389	trans-Caffeic acid	[M-H]-	179.03497	C9H8O4	Caffeic acid and derivatives	QAIPRVGONGVQAS-UHFFFAOYSA-N	O=C(O)C=CC1=CC=C(O)C(O)=C1
1319	3.943	244.15416	Pinacidil	[M-H]-	244.15677	C13H19N5	Pyridines and derivatives	IVVNZDGDKPTYHK-UHFFFAOYNA-N	N#CN=C(N=C1C=CNC=C1)NC(C)C(C)(C)C
1320	3.944	231.16985	Isoleucylvaline	[M+H]+	231.1707	C11H22N2O3	Dipeptides	BCXBIONYYJCSDF-UHFFFAOYNA-N	O=C(O)C(NC(=O)C(N)C(C)CC)C(C)C
1321	3.95	323.19946	16-Heptadecyne-1,2,4-triol	[M+H]+	323.1983	C17H32O3	Long-chain fatty alcohols	OHLQBKZXSJYBMK-UHFFFAOYSA-N	C#CCCCCCCCCCCCC(O)CC(O)CO
1322	3.95	292.20428	Terbinafine	[M+H]+	292.20596	C21H25N	Naphthalenes	DOMXUEMWDBAQBQ-UHFFFAOYSA-N	CC(C)(C)C#CC=CCN(C)CC1=CC=CC2=CC=CC=C21
1323	3.951	241.14296	Imiquimod	[M+H]+	241.145	C14H16N4	Imidazoquinolines	DOUYETYNHWVLEO-UHFFFAOYSA-N	N1=CN(C2=C1C(=NC=3C=CC=CC32)N)CC(C)C
1324	3.952	270.16925	(8aR,12S,12aR)-12-hydroxy-4-methyl-4,5,6,7,8,8a,12,12a-octahydro-1H-3-benzoxecine-2,9-dione	[M+H]+	270.16998	C14H20O4	Oxocins	WTQWDNIIFGEBSZ-ROAVRGLVSA-N	O=C1OC(C)CCCCC2C(=O)C=CC(O)C2C1
1325	3.965	151.07516	1-(4-methoxyphenyl)ethanone	[M+H]+	151.08	C9H10O2	Alkyl-phenylketones	NTPLXRHDUXRPNE-UHFFFAOYSA-N	O=C(C1=CC=C(OC)C=C1)C
1326	3.97	160.09651	N-Isovalerylglycine	[M+H]+	160.09682	C7H13NO3	N-acyl-alpha amino acids	ZRQXMKMBBMNNQC-UHFFFAOYSA-N	O=C(O)CN=C(O)CC(C)C
1327	3.971	187.09625	2-(1-hydroxyethyl)-4-(2-hydroxypropyl)-2H-furan-5-one	[M+H]+	187.09648	C9H14O4	Butenolides	JAAFNWYGLROOGI-UHFFFAOYSA-N	O=C1OC(C=C1CC(O)C)C(O)C
1328	3.976	321.15485	8:1+2O fatty acyl hexoside	[M-H]-	321.1546	C14H26O8	Fatty acyl hexosides	YUPCLHHTUNDMAN-UHFFFAOYNA-N	O=C(OCCCC)CC(OC1OC(CO)C(O)C(O)C1(O))C
1329	3.978	271.13528	alpinetin	[M+H]+	271.1337	C16H14O4	5-O-methylated flavonoids	QQQCWVDPMPFUGF-UHFFFAOYSA-N	O=C1C=2C(OC)=CC(O)=CC2OC(C=3C=CC=CC3)C1
1330	3.98	625.15057	3-O-beta-(6''-trans-caffeoyl)-galactopyranosyl quercetin	[M-H]-	625.14801	C30H26O15	Flavonoid 3-O-p-coumaroyl glycosides	IHBVMUCQCZEAPW-YBSDSQCQSA-N	O=C(OCC1OC(OC=2C(=O)C=3C(O)=CC(O)=CC3OC2C=4C=CC(O)=C(O)C4)C(O)C(O)C1O)C=CC5=CC=C(O)C(O)=C5
1331	3.981	427.18152	[8,8-dimethyl-10-(3-methylbut-2-enoyloxy)-2-oxo-9,10-dihydropyrano[2,3-f]chromen-9-yl] 3-methylbutanoate	[M-H]-	427.17621	C24H28O7	Angular pyranocoumarins	MYXNOIIPXWBFFR-UHFFFAOYSA-N	O=C1OC2=C(C=C1)C=CC=3OC(C)(C)C(OC(=O)CC(C)C)C(OC(=O)C=C(C)C)C32

1332	3.982	257.17389	((6aR,9R,10aR)-7-methyl-4,6,6a,7,8,9,10,10a-octahydroindolo[4,3-fg]quinolin-9-yl)methanol	[M+H]+	257.17001	C16H20N20	Clavines and derivatives	UFKTZIXVYHGAES-WDBKCZKBSA-N	OCC1CN(C)C2CC3=CNC4=CC=CC(=C43)C2C1
1333	3.986	245.1019	Indole-3-acetyl-L-alanine	[M-H]-	245.09317	C13H14N2O3	N-acyl-L-alpha-amino acids	FBDCJLXTUCMFLF-UHFFFAOYNA-N	CC(N=C(0)CC1=CNC2=CC=CC=C12)C(0)=0
1334	3.987	338.12286	barceloneic acid A	[M+H]+	338.12341	C16H16O7	Diphenylethers	ULPVIBVOKQAXJB-UHFFFAOYSA-N	0=C(0)C1=C(0)C=C(C=C10C2=C(0)C=C(0C)C=C2C0)C
1335	3.989	328.23236	UR-144 N(4-hydroxypentyl)	[M+H]+	328.22699	C21H29NO2	N-alkylindoles	YHFHYHZNLCEAAP-UHFFFAOYSA-N	0=C(C1=CN(C=2C=CC=CC12)CCCC(0)C)C3C(C)(C)C3(C)C
1336	3.99	190.05022	(2-oxo-2,3-dihydro-1H-indol-3-yl)acetic acid	[M-H]-	190.05096	C10H9NO3	Indolyl carboxylic acids and derivatives	ILGMGHZPXRDCCS-UHFFFAOYNA-N	0=C(0)CC1C(0)=NC=2C=CC=CC21
1337	3.994	247.08559	(3Z)-3-butylidene-6,7-dihydroxy-4,5,6,7-tetrahydro-2-benzofuran-1-one	[M+H]+	247.09	C12H16O4	Isobenzofurans	DQNGMIQSXNGHOA-WTKPLQERSA-N	0=C10C(=CCCC)C2=C1C(0)C(0)CC2
1338	4.001	408.19009	3-(2-((7H-purin-6-yl)amino)ethyl)-2-methyl-4-oxo-3,4,5,6-tetrahydro-2H-2,6-methanobenzo[g][1,3]oxazocine-5-carboxamide	[M+H]+	408.19	C20H21N7O3	6-alkylaminopurines	MMJORIFPGDWBAA-UHFFFAOYSA-N	0=C(N)C1C(=0)N(CCNC2=NC=NC=3N=CNC32)C4(0C=5C=CC=CC5C1C4)C
1339	4.001	390.14249	Taldalafil	[M+H]+	390.14484	C22H19N3O4	Beta carbolines	WOXKDUGGOYFFRN-UHFFFAOYNA-N	0=C1N2C(C3=CC=C40C0C4=C3)C=5NC=6C=CC=CC6C5CC2C(=0)N(C)C1
1340	4.002	251.17474	Rivastigmine tartrate (Exelon)	[M+H]+	251.175	C18H28N2O8	Phenoxy compounds	GWHQHAUAXRMMOT-MBANBULQSA-N	0=C(0)C(0)C(0)C(=0)0.0=C(0C1=CC=CC(=C1)C(N(C)C)C)N(C)CC
1341	4.004	313.11664	2-(3,4-dimethoxyphenyl)-7-methoxy-4H-chromen-4-one	[M+H]+	313.10999	C18H16O5	7-O-methylated flavonoids	VSFZYCDPDWSYSS-UHFFFAOYSA-N	0=C1C=C(0C2=CC(0C)=CC=C12)C=3C=CC(0C)=C(0C)C3
1342	4.009	434.22311	Valsartan	[M-H]-	434.21976	C24H29N5O3	Valine and derivatives	ACWBQPMHZXGDFX-UHFFFAOYNA-N	CCCCC(N(CC1=CC=C(C=C1)C2=CC=CC=C2C3=NNN=N3)C(C(C)C)C(=0)0)=0
1343	4.01	309.08609	Gastrodin	[M+H]+	309.09	C13H18O7	Phenolic glycosides	PUQSUZTXKPLAPR-UJPOAAIJSA-N	OCC1=CC=C(0C20C(C0)C(0)C(0)C20)C=C1
1344	4.01	386.2088	Uncarine c	[M+H]+	386.20743	C21H24N2O4	Indolizidines	JMIAZDVHNCCPDM-UHFFFAOYSA-N	0=C(0C)C1=COC(C)C2CN3CCC4(C(0)=NC=5C=CC=CC54)C3CC12
1345	4.031	332.9382	PFAP-diPAP	[M-H]-	332.93799	C4HF10O4P	PFSA	HJLWRXZHQHRZCH-UHFFFAOYSA-N	0=P(0)(0C(F)(F)F)0C(F)(F)C(F)(F)C(F)(F)F
1346	4.04	257.14899	Isocurcumenol	[M+H]+	257.14999	C15H22O2	Guaianes	DEBDFZGNZTYPMF-NZBPQXDJSA-N	OC120C3(CC1=C(C)C)C(C(=C)C2)CCC3C
1347	4.041	165.05421	P-Coumaric acid	[M+H]+	165.05499	C9H8O3	Hydroxycinnamic acids	NGSWKAQJJWESNS-ZZXKWVIFSA-N	0=C(0)C=CC1=CC=C(0)C=C1
1348	4.042	579.12897	quercetin-3-O-deoxyhexosyl(1-2)pentoside	[M-H]-	579.13397	C26H28O15	Flavonoid-3-O-glycosides	WRLBRIWXGBKVHQ-UHFFFAOYSA-N	0=C1C(0C20C(C)C(0)C(0)C20C30CC(0)C(0)C30)=C(0C=4C=C(0)C=C(0)C14)C=5C=CC(0)=C(0)C5
1349	4.043	474.27902	Chasmanine	[M+H]+	474.28	C25H41NO6	Aconitane-type diterpenoid alkaloids	DBODJJZRZFZBBD-XXYWQWTJSA-N	OC1C2CC3C1C(0)(CC20C)C4C(0C)C5C6(C0C)CN(CC)C4C35C(0C)CC6
1350	4.045	506.30612	2-((4R)-4-((3R,5S,7R,8R,9S,12S,14S,17R)-3,7,12-trihydroxy-10,13-dimethylhexadecahydro-1H-cyclopenta[a]phenanthren-17-yl)pentanamido)acetic	[M+H]+	506.31	C26H45NO7	Glycinated bile acids and derivatives	WDKPRHOCWKLQPK-SSBNMPHASA-N	0=C(0)CNC(=0)CCC(C)C1CCC2C3C(0)CC4CC(0)CCC4(C)C3CC(0)C12C.0
1351	4.049	152.01614	2-Hydroxybenzothiazole	[M+H]+	152.01646	C7H5NOS	Benzothiazoles	YEDUAINPPJYDJZ-UHFFFAOYSA-N	C1=CC=C2C(=C1)N=C(0)S2
1352	4.061	239.12686	7-Hydroxyflavone	[M+H]+	239.12721	C15H10O3	Flavones	MQGPSCMMNJKMHQ-UHFFFAOYSA-N	0=C1C=C(0C2=CC(0)=CC=C12)C=3C=CC=CC3
1353	4.073	613.17047	Safflomin A	[M+H]+	613.16998	C27H32O16	C-glycosyl compounds	IAVUBSCVWHLRGE-UXEKTNMQSA-N	0=C1C(=C(0)C=CC2=CC=C(0)C=C2)C(0)=C(C(=0)C1(0)C30C(C0)C(0)C(0)C30)C40C(C0)C(0)C(0)C40
1354	4.08	373.11234	(2S,3S,4S,5R,6R)-6-(3-benzoyloxy-2-hydroxypropoxy)-3,4,5-trihydroxyoxane-2-carboxylic acid	[M+H]+	373.11292	C16H20O10	O-glucuronides	DLZMKPZOEYKXHQ-MQCRQNCBSA-N	0=C(0)C10C(0CC(0)C0C(=0)C=2C=CC=CC2)C(0)C(0)C10
1355	4.08	395.09357	[(E)-3-acetyloxy-7-hydroxy-6-methoxy-7-(6-oxo-2,3-dihydropyran-2-yl)hept-4-en-2-yl] acetate	[M+H]+	395.09351	C17H24O8	Tricarboxylic acids and derivatives	STNNWPKHRBGYMO-CMDGGOBGSA-N	0=C10C(CC=C1)C(0)C(0C)C=CC(0C(=0)C)C(0C(=0)C)C
1356	4.085	209.04498	Hydroxyferulic acid	[M-H]-	209.04469	C10H10O5	Hydroxyferulic acid and derivatives	YFXWTVLDSKSYLW-UHFFFAOYSA-N	0=C(0)C=CC1=CC(0)=C(0)C(0C)=C1
1357	4.093	609.14478	kaempferol-3-O-hexoxyl(1-6)hexoside	[M-H]-	609.14502	C27H30O16	Flavonoid-3-O-glycosides	BITPRCODIALMOV-UHFFFAOYSA-N	0=C1C(0C20C(C0C30C(C0)C(0)C(0)C30)C(0)C(0)C20)=C(0C=4C=C(0)C=C(0)C14)C=5C=CC(0)=CC5
1358	4.101	263.08356	Indarubicin	[M+H]+	263.08151	C16H10N2O2	Indolines	CRDNMYFJWFXOCH-UHFFFAOYSA-N	0=C1C=2C=CC=CC2NC1=C3C(0)=NC=4C=CC=CC43
1359	4.11	323.19476	2-(3,5-difluorophenyl)-N-(((1S,9aR)-octahydro-1H-quinolizin-1-yl)methyl)acetamide	[M+H]+	323.19199	C18H24F2N2O	Quinolizines	LINCEHCWNRFISH-WMLDXEAASA-N	0=C(NCC1CCCN2CCCCC21)CC=3C=C(F)C=C(F)C3
1360	4.112	231.12202	(2S)-2-[(1S,2R)-1,2-dihydroxypentyl]-4-methoxy-2,3-dihydropyran-6-one	[M+H]+	231.1227	C11H18O5	Dihydropyranones	YLHOHANRUSKHKO-YWVKMMECSA-N	0=C10C(CC(0C)=C1)C(0)C(0)CCC
1361	4.127	609.14258	Flavonol base + 4O, O-dHex-Hex	[M-H]-	609.14423	C27H30O16	Flavonol O-glycosides	DFNXNCCYQRPZMD-UHFFFAOYNA-N	0=C3C(0C20C(C)C(0)C(0)C2(0C10C(C0)C(0)C(0)C1(0)))=C(0C4=CC(0)=CC(0)=C34)C=5C=CC(0)=C(0)C=5
1362	4.132	365.13239	[(E)-3-acetyloxy-6,7-dihydroxy-7-(6-oxo-2,3-dihydropyran-2-yl)hept-4-en-2-yl] acetate	[M+H]+	365.12869	C16H22O8	Tricarboxylic acids and derivatives	RAZHULSADOGMPF-BQYQJAHWSA-N	0=C10C(CC=C1)C(0)C(0)C=CC(0C(=0)C)C(0C(=0)C)C
1363	4.136	529.22656	(2R,3R,6R,8R,9S,12S,13R,14R,15R,16R)-6,8,14,15-tetrahydroxy-2,6,13,16-tetramethyl-3-{[(2R,3R,4S,5S,6R)-3,4,5-trihydroxy-6-(hydroxymethyl)oxan-2-	[M-H]-	529.22906	C25H38O12	Eudesmanolides, secoeudesmanolides, and	TYXFVKGQQWDJKA-YFXVJPQZSA-N	0=C10C2C(0)C3C(0)(C=CC(0C40C(C0)C(0)C(0)C40)C3(C)C5C(0)C(0)C(C)C1C25C)C
1364	4.137	290.12238	6,7-dimethoxy-1-phenyl-3,4-dihydro-isoquinoline	[M+H]+	290.12	C17H17NO2	Dihydroisoquinolines	WGTCMJBJRPKENJ-UHFFFAOYSA-N	N1=C(C=2C=CC=CC2)C=3C=C(0C)C(0C)=CC3CC1
1365	4.147	259.14377	1-isopropyl-1,2,3,4,-tetrahydro-beta-carboline-3-carboxylic acid	[M+H]+	259.14999	C15H18N2O2	Harmala alkaloids	PQJWDNCGZBQHRJ-OLZOCXBDSA-N	0=C(0)C1NC(C=2NC=3C=CC=CC3C2C1)C(C)C
1366	4.149	164.03465	MBOA	[M-H]-	164.03461	C8H7NO3	Benzoxazinoids	MKMCJLMBVKHUMS-UHFFFAOYSA-N	0=C10C2=CC(0C)=CC=C2(N1)
1367	4.157	272.18494	3-OH-C10-HSL	[M+H]+	272.185	C14H25NO4	N-acyl-alpha amino acids and derivatives	DOICJCCMIBBSOO-KIYNQFGBSA-N	0=C10CCC1N=C(0)CC(0)CCCCCCC
1368	4.159	289.08102	Malonyltryptophan	[M-H]-	289.0813	C14H14N2O5	Amino acids	OVEAWSPZRGBTSS-UHFFFAOYNA-N	0=C(0)CC(=0)NC(C(=0)0)CC2=CNC=1C=CC=CC=12
1369	4.161	119.04945	4-Vinylphenol	[M-H]-	119.05024	C8H8O	Styrenes	FUGYGGDSWSUORM-UHFFFAOYSA-N	OC1=CC=C(C=C)C=C1
1370	4.162	163.03909	Phenylpyruvic acid	[M-H]-	163.04007	C9H8O3	Phenylpyruvic acid derivatives	BTNMPGBKDVTSJY-UHFFFAOYSA-N	0=C(0)C(=0)CC=1C=CC=CC1
1371	4.17	380.21655	pipernonaline	[M+H]+	380.21512	C21H27NO3	Benzodioxoles	PKLGRWSJBLGIBF-JMQWPVDRSA-N	0=C(C=CCCCCC=CC1=CC=C20C0C2=C1)N3CCCCC3
1372	4.171	127.07519	Melamine	[M+H]+	127.07267	C3H6N6	1,3,5-triazines	JDSHMPZPIAZGSV-UHFFFAOYSA-N	C1(=N)NC(=N)NC(=N)N1
1373	4.18	215.13794	DETHIOBIOTIN	[M+H]+	215.14	C10H18N2O3	Imidazolyl carboxylic acids and derivatives	AUTOLBMXDDTRRT-UHFFFAOYSA-N	0=C1NC(C)C(N1)CCCCCC(=0)0
1374	4.181	172.09668	N-Acetylleucine	[M-H]-	172.09792	C8H15NO3	Leucine and derivatives	WXNXCEHXYPACJF-UHFFFAOYNA-N	0=C(0)C(N=C(0)C)CC(C)C

1375	4.184	625.17059	Isorhamnetin 3-O-neohesperoside	[M+H]+	625.16998	C28H32O16	Flavonoid-3-O-glycosides	QHLKSZBFIJJREC-SPSUIZEHSA-N	O=C1C(OC2OC(CO)C(O)C(O)C2OC3OC(C)C(O)C(O)C3O)=C(OC=4C=C(O)C=C(O)C14)C=5C=CC(O)=C(OC)C5
1376	4.191	180.10138	N-acetyltyramine	[M+H]+	180.1019	C10H13NO2	1-hydroxy-2-unsubstituted benzenoids	ATDWJOOPFDQZNK-UHFFFAOYSA-N	OC1=CC=C(C=C1)CCN=C(O)C
1377	4.196	171.13741	gamma-Decalactone	[M+H]+	171.13795	C10H18O2	Gamma butyrolactones	IFYYFLINQYPWGJ-UHFFFAOYNA-N	CCCCCCC1CCC(=O)O1
1378	4.201	333.10757	Isopongaflavone	[M-H]-	333.11322	C21H18O4	Pyranoflavonoids	DPAGRPSAFDXQDN-UHFFFAOYSA-N	O=C1C=C(OC2=C3C=CC(OC3=CC(OC)=C12)(C)C)C=4C=CC=CC4
1379	4.211	327.20206	hydroquinidine	[M+H]+	327.20001	C20H26N2O2	Cinchona alkaloids	LJOQGZACKSYWCH-NIHZBZNOSA-N	OC(C=1C=CN=C2C=CC(OC)=CC21)C3N4CCC(C3)C(C4)CC
1380	4.214	434.20047	(2R,3S,4S,5R,6R)-2-[[(2S,3R,4R)-3,4-dihydroxy-4-(hydroxymethyl)oxolan-2-yl]oxymethyl]-6-(2-phenylethoxy)oxane-3,4,5-triol	[M+NH4]+	434.20206	C19H28O10	O-glycosyl compounds	GZSQKOFXMZDKPV-DERWZFJFSA-N	OCC1(O)COC(OCC2OC(OCCC=3C=CC=CC3)C(O)C(O)C2O)C1O
1381	4.222	407.14322	Amlodipine	[M-H]-	407.13791	C20H25ClN2O5	Dihydropyridinecarboxylic acids and derivatives	HTIQEAQVCYTUBX-UHFFFAOYNA-N	O=C(OC)C1=C(NC(=C(C(=O)OCC)C1C=2C=CC=CC2Cl)COCCN)C
1382	4.229	327.08633	5-hydroxy-3,7-dimethoxy-2-(4-methoxyphenyl)-4H-chromen-4-one	[M-H]-	327.086	C18H16O6	7-O-methylated flavonoids	WSQWAMGRHJQANC-UHFFFAOYSA-N	O=C1C(OC)=C(OC=2C=C(OC)C=C(O)C12)C=3C=CC(OC)=CC3
1383	4.233	153.05418	Vanillin	[M+H]+	153.05463	C8H8O3	Methoxyphenols	MWOOGOJBHIARFG-UHFFFAOYSA-N	O=CC1=CC=C(O)C(OC)=C1
1384	4.234	381.1911	Histamine-trifluoromethyltoluide	[M-H]-	381.19077	C19H25F3N4O	Trifluoromethylbenzenes	PMKJGGBYYNEYPA-UHFFFAOYNA-N	O=C(NC1=CC=C(C=C1)C(F)(F)F)CCCCC(NCCC2=CN=CN2)C
1385	4.236	182.08073	Tyrosine	[M+H]+	182.08099	C9H11NO3	Tyrosine and derivatives	OUYCCCASQSFEME-UHFFFAOYSA-N	O=C(O)C(N)CC1=CC=C(O)C=C1
1386	4.253	433.1106	Vitexin	[M+H]+	433.11292	C21H20O10	Flavonoid 8-C-glycosides	SGEWCQFRYRRZDC-VPRICQMDSA-N	OC[C=H]1O[C=H]([C=H](O)[C=H](O)[C=H]1O)C1=C(O)C=C(O)C2=C1OC(=CC2=O)C1=CC=C(O)C=C1
1387	4.255	320.2059	Tributyrin	[M+H]+	320.207	C15H26O6	Triacylglycerols	UYXTWWCETRIEDR-UHFFFAOYSA-N	O=C(OCC(OC(=O)CCC)COC(=O)CCC)CCC
1388	4.258	211.14357	Cyclo(Leu-Pro)	[M+H]+	211.1441	C11H18N2O2	Alpha amino acids and derivatives	SZJNCZMRZAUNQT-UHFFFAOYNA-N	O=C1N2CCCC2C(O)=NC1CC(C)C
1389	4.259	189.07564	Hydroxysuberic acid	[M-H]-	189.0766	C8H14O5	Organic acids	ARJZZFJXSNJKGR-UHFFFAOYNA-N	O=C(O)CCCCC(O)CC(=O)O
1390	4.261	357.09692	5-Hydroxy-2',4',7,8-Tetramethoxyflavone	[M-H]-	357.09796	C19H18O7	8-O-methylated flavonoids	AHUAGKUYEMQHTH-UHFFFAOYSA-N	O=C1C=C(OC2=C(OC)C(OC)=CC(O)=C12)C=3C=CC(OC)=CC3OC
1391	4.264	380.94601	PFSA-H	[M-H]-	380.94601	C6H2F12O3S	PFSA	DOWJLTPWPGXVFD-UHFFFAOYSA-N	O=S(=O)(O)C(F)(F)C(F)(F)C(F)(F)C(F)(F)C(F)C(F)(F)F
1392	4.265	369.09677	Acacetin Diacetate	[M+H]+	369.09686	C20H16O7	4'-O-methylated flavonoids	VYHHSBHXZVSILO-UHFFFAOYSA-N	O=C(OC=1C=C(OC(=O)C)C=2C(=O)C=C(OC2C1)C=3C=CC(OC)=CC3)C
1393	4.266	347.11563	Torasemide	[M-H]-	347.11835	C16H20N4O3S	Pyridinesulfonamides	NGBFQHCMQULJNZ-UHFFFAOYSA-N	CC(C)N=C(NS(C1=CNC=CC1=NC2=CC=CC(C)=C2)(=O)=O)O
1394	4.276	179.15379	6,7-Dihydroxycoumarin;esculetin	[M+H]+	179.14999	C9H6O4	6,7-dihydroxycoumarins	ILEDWLMCKZNDJK-UHFFFAOYSA-N	O=C1OC=2C=C(O)C(O)=CC2C=C1
1395	4.282	510.25674	[3,4,5-trihydroxy-6-[[3,4,5-trihydroxy-6-(hydroxymethyl)oxan-2-yl]oxymethyl]oxan-2-yl] 2,6,6-trimethylcyclohexene-1-carboxylate	[M+H]+	510.25449	C22H36O12	O-glycosyl compounds	URTXXFGOVQACIC-UHFFFAOYSA-N	O=C(OC1OC(COC2OC(CO)C(O)C(O)C2O)C(O)C(O)C1O)C3=C(C)CCCC3(C)C
1396	4.288	253.10754	Phomalone	[M-H]-	253.10814	C13H18O5	Alkyl-phenylketones	PTBQWWHUOMDVFS-UHFFFAOYSA-N	O=C(C1=C(O)C(=C(O)C=C1OC)CCO)CCC
1397	4.288	496.24182	Silodosin (Rapaflo)	[M+H]+	496.242	C25H32F3N3O4	Indolecarboxamides and derivatives	PNCPYILNMDWPEY-QGZVFWFLSA-N	O=C(N)C1=CC(=CC2=C1N(CC2)CCCO)CC(NCCOC=3C=CC=CC3OCC(F)(F)F)C
1398	4.289	470.29544	(E)-5-hydroxy-N-[3-[5-[3-[[(E)-5-hydroxy-3-methylpent-2-enoyl]amino]propyl]-3,6-dioxopiperazin-2-yl]propyl]-3-methylpent-2-enamide	[M+H]+	470.2973	C22H36N4O6	Alpha amino acids and derivatives	CNQCBOHTKBVCFM-WXUKJITCSA-N	OC(=NCCCC1N=C(O)C(N=C1O)CCCN=C(O)C=C(C)CCO)C=C(C)CCO
1399	4.299	508.27811	Epothilon B	[M+H]+	508.27301	C27H41NO6S	Epothilones and analogues	QXRSDHAAWVKZLJ-FBAHCSNKSA-N	O=C1OC(C(=CC=2N=C(SC2)C)C)CC3OC3(C)CCCC(C)C(O)C(C(=O)C(C)(C)C(O)C1)C
1400	4.303	203.04448	THYMOQUINONE	[M+H]+	203.04689	C10H12O2	P-benzoquinones	KEQHJBNSCLWCAE-UHFFFAOYSA-N	O=C1C=C(C(=O)C=C1C)C(C)C
1401	4.304	343.12796	2,6-dihydroxy-7-methoxy-1,1,4a-trimethyl-3,4,10,10a-tetrahydro-2H-phenanthren-9-one	[M+H]+	343.13062	C18H24O4	Diterpenoids	JKTSDLANZJEZMK-UHFFFAOYSA-N	O=C1C2=CC(OC)=C(O)C=C2C3(C)CCC(O)C(C)(C)C3C1
1402	4.306	538.27393	taurohyocholic acid	[M+H]+	538.28003	C26H45NO7S	Trihydroxy bile acids, alcohols and derivatives	XSOLDPYUICCHJX-DSNLCWFJSA-N	O=S(=O)(O)CCN=C(O)CCC(C)C1CCC2C3C(O)C(O)C4CC(O)CCC4(C)C3CCC12C
1403	4.307	409.11041	(1s)-1,5-anhydro-1-[2,4,6-trihydroxy-3-(4-hydroxybenzoyl)phenyl]-d-glucitol	[M+H]+	409.11292	C19H20O10	Phenolic glycosides	BZYKNVLTMWYEFA-ZJKJAXBQSA-N	O=C(C1=CC=C(O)C=C1)C2=C(O)C=C(O)C(=C2O)C3OC(CO)C(O)C(O)C3O
1404	4.311	269.03339	kavain	[M+H]+	269.02936	C14H14O3	Kavalactones	XEAQIWGXBXCYFX-GUOLPTJISA-N	O=C1OC(C=CC=2C=CC=CC2)CC(OC)=C1
1405	4.311	329.21667	Peramivir Trihydrate	[M+H]+	329.21799	C15H34N4O7	Gamma amino acids and derivatives	RFUCJKFZFXNIGB-ZBBHRWOZSA-N	O=C(O)C1CC(NC(=N)N)C(C1O)C(NC(=O)C)C(CC)CC.O.O.O
1406	4.312	607.16565	Flavonol base + 3O, 1MeO, O-dHex-dHex	[M-H]-	607.16571	C28H32O15	Flavonol O-glycosides	DGFGSUOQDJHNFL-UHFFFAOYNA-N	O=C3C(OC2OC(C)C(OC1OC(C)C(O)C(O)C1(O))C(O)C2(O))=C(OC4=CC(OC)=CC(O)=C34)C=5C=CC(O)=C(O)C=5
1407	4.314	543.23834	Symplocosin	[M+H]+	543.23749	C26H32O11	Lignan glycosides	QLJNETOQFQXTLI-UHFFFAOYSA-N	OC1=CC=C(C=C1OC)C2OCC3C(OCC23)C4=CC=C(OC5OC(CO)C(O)C(O)C5O)C(OC)=C4
1408	4.322	116.07043	L-(-)-Proline	[M+H]+	116.0706	C5H9NO2	Proline and derivatives	ONIBWKKTOPOVIA-BYPYZUCNSA-N	OC(=O)[C=H]1CCCN1
1409	4.331	284.14798	Metolachlor	[M+H]+	284.14117	C15H22ClNO2	Anilides	WVQBLGZPHOPPFO-UHFFFAOYNA-N	CCC1=CC=CC(C)=C1N(C(C)COC)C(=O)CCl
1410	4.333	385.11234	Sinapoylhexoside (isomer of 955)	[M-H]-	385.1131	C17H22O10	Sinapinic acid and derivatives	XRKBRPFTFKKHEF-UHFFFAOYNA-N	O=C(OC1OC(CO)C(O)C(O)C1(O))C=CC=2C=C(OC)C(O)=C(OC)C=2
1411	4.336	341.13715	Flavanone base + 3O, 1Prenyl	[M+H]+	341.13721	C20H20O5	Prenylated flavanones	LPEPZZAVFJPLNZ-UHFFFAOYNA-N	O=C2C=3C(O)=CC(O)=C(C=3(OC(C1=CC=C(O)C=C1)C2))CC=C(C)C
1412	4.337	405.15057	Desoxyrhaponticin	[M+H]+	405.14999	C21H24O8	Stilbene glycosides	MFMQRDLLSRLUJY-DXKBKAGUSA-N	OC=1C=C(OC2OC(CO)C(O)C(O)C2O)C=C(C=CC3=CC=C(OC)C=C3)C1
1413	4.339	149.0959	ANETHOLE	[M+H]+	149.09608	C10H12O	Anisoles	RUVINXPYWBROJD-ARJAWSKDSA-N	O(C1=CC=C(C=C1)C=CC)C
1414	4.354	309.09512	D-(-)-salicin	[M+H]+	309.09448	C13H18O7	Phenolic glycosides	NGFMICBWJRZIBI-UHFFFAOYSA-N	OCC=1C=CC=CC1OC2OC(CO)C(O)C(O)C2O
1415	4.355	560.15576	(2S)-6-[(2S)-5,7-dihydroxy-2-(4-hydroxyphenyl)-4-oxo-2,3-dihydrochromen-8-yl]-5,7-dihydroxy-2-(4-hydroxyphenyl)-2,3-dihydrochromen-4-one	[M+H]+	560.15509	C30H22O10	Biflavonoids and polyflavonoids	YBDIZQWDBBOOFB-GOTSBHOMSA-N	O=C1C=2C(O)=CC(O)=C(C2OC(C3=CC=C(O)C=C3)C1)C=4C(O)=CC=5OC(C6=CC=C(O)C=C6)CC(=O)C5C4O
1416	4.356	227.01837	Trinitrotoluene	[M-H]-	227.01839	C7H5N3O6	Nitrobenzenes	SPSSULHKWOKEEL-UHFFFAOYSA-N	CC1=C(C=C(C=C1N(=O)=O)N(=O)=O)N(=O)=O
1417	4.364	237.03891	SINAPIC ACID METHYL ETHER	[M-H]-	237.04083	C12H14O5	Coumaric acids and derivatives	YTFVRYKNXDADBI-SNAWJCMRSA-N	O=C(O)C=CC1=CC(OC)=C(OC)C(OC)=C1

1418	4.37	154.15851	d-LIMONENE	[M+H]+	154.15901	C10H16	Menthane monoterpenoids	XMGQYMWWDOXHJM-JTQLQIEISA-N	C=C(C)C1CC=C(C)CC1
1419	4.375	326.13467	Boldine	[M-H]-	326.13977	C19H21NO4	Alkaloids	LZJRNLRASBVRRX-UHFFFAOYNA-N	COC1=CC2=C(CC3N(C)CCC4=CC(O)=C(OC)C2=C34)C=C1O
1420	4.375	387.19943	Sinapoylhexoside	[M+H]+	387.20081	C17H22O10	Sinapinic acid and derivatives	XRKBRPFTFKKHEF-ONEGZZNKNA-N	O=C(OC1OC(CO)C(O)C(O)C1(O))C=CC=2C=C(OC)C(O)=C(OC)C=2
1421	4.377	490.31979	Oxysporidinone_130003	[M+H]+	490.31699	C28H43NO6	Terpene glycosides	CYNJYGDSSURTLH-CPNJWEJPSA-N	O=C1C(=C(O)C(=CN1C)C2(O)CCC(=O)CC2O)C3OC(C(=CC(C)CC(C)CC)C)C(C)CC3
1422	4.388	647.16846	4-(3-oxobutyl)phenyl 6-O-[(2E)-3-(4-hydroxyphenyl)prop-2-enoyl]-2-O-(3,4,5-trihydroxybenzoyl)-beta-D-glucopyranoside	[M+H]+	647.16998	C32H32O13	Phenolic glycosides	QIEOAEKNEKYVTH-IQDFXBJLSA-N	O=C(OCC1OC(OC2=CC=C(C=C2)CCC(=O)C)C(OC(=O)C3=CC(O)=C(O)C(O)=C3)C(O)C1O)C=CC4=CC=C(O)C=C4
1423	4.39	314.11316	5-[(E)-3-hydroxybut-1-enyl]-4-[4-[(E)-3-oxobut-1-enyl]anilino]oxolan-2-one	[M-H]-	314.11084	C18H21NO4	Cinnamic acids and derivatives	HGCMCLAKARHJBW-OXWOXFIQSA-N	O=C1OC(C=CC(O)C)C(NC2=CC=C(C=CC(=O)C)C=C2)C1
1424	4.392	413.14447	3-oxo-3-[[(2R,3S,4S,5R,6S)-3,4,5-trihydroxy-6-(4-hydroxy-5-methyl-2-propan-2-ylphenoxy)oxan-2-yl]methoxy]propanoic acid	[M-H]-	413.14532	C19H26O10	Terpene glycosides	VJVGTGQETNCOEX-LQDZTQBFSA-N	O=C(O)CC(=O)OCC1OC(OC2=CC(=C(O)C=C2C(C)C)C)C(O)C(O)C1O
1425	4.395	304.1167	N-(6-methoxypyridin-3-yl)-1-methyl-1H-indole-6-carboxamide	[M+H]+	304.10999	C16H15N3O2	Indolecarboxamides and derivatives	IEYPTSQYHVVDAC-UHFFFAOYSA-N	O=C(NC1=CN=C(OC)C=C1)C=2C=CC=3C=CN(C3C2)C
1426	4.395	454.16919	Phlorhizin	[M+H]+	454.17078	C21H24O10	Flavonoid O-glycosides	IOUVKUPGCMBWBT-QNDFHXLGSA-N	O=C(C=1C(O)=CC(O)=CC1OC2OC(CO)C(O)C(O)C2O)CCC3=CC=C(O)C=C3
1427	4.398	178.01389	S-CARBOXYMETHYL-L-CYSTEINE	[M-H]-	178.017	C5H9NO4S	L-cysteine-S-conjugates	GBFLZEXEOZUWRN-VKHMYHEASA-N	O=C(O)CSCC(N)C(=O)O
1428	4.399	250.10684	Goniothalenol	[M+H]+	250.10738	C13H12O4	Furopyrans	ZKIRVBNLJKGIEM-UHFFFAOYSA-N	O=C1OC2C(OC(C=3C=CC=CC3)C2O)C=C1
1429	4.409	385.21753	(4aR)-5-hydroxy-6-methoxy-1,1-dimethyl-7-propan-2-yl-2,3,4,9,10,10a-hexahydrophenanthrene-4a-carboxylic acid	[M+H]+	385.21786	C21H30O4	Diterpenoids	QQNSARJGBPMQDI-MZVUKIKXSA-N	O=C(O)C12C=3C(O)=C(OC)C(=CC3CCC1C(C)(C)CCC2)C(C)C
1430	4.415	347.10925	Demethyleneberberine	[M+H]+	347.10999	C19H18NO4+	Protoberberine alkaloids and derivatives	HVTCKKMWZDDWOY-UHFFFAOYSA-O	OC=1C=C2C=3C=C4C=CC(OC)=C(OC)C4=C[N+]3CCC2=CC1O
1431	4.415	327.11804	Dendrophenol	[M+H]+	327.12	C17H20O5	Stilbenes	YTRAYUIKLRABOQ-UHFFFAOYSA-N	OC1=CC=C(C=C1OC)CCC2=CC(OC)=C(O)C(OC)=C2
1432	4.417	288.18002	(4-oxido-2,3,5,6,7,8-hexahydro-1H-pyrrolizin-4-ium-1-yl)methyl 2,3-dihydroxy-3-methylpentanoate	[M+H]+	288.18054	C14H25NO5	Pyrrolizidines	QWYNXLBAFAPSKQ-UHFFFAOYSA-N	O=C(OCC1CCN2(=O)CCCC12)C(O)C(O)(C)CC
1433	4.428	367.18542	Tobramycin 1-N- or 3-N-carbamoyl, ion source fragment	[M+H]+	367.18301	C14H30N4O7	Aminocyclitol glycosides	IUFQFQNFCIGWHJ-FZEZGWBXSA-N	OCC1OC(OC2C(O)C(OCCN)C(N)CC2N)C(O)C(N)C1O
1434	4.434	267.16922	Panaxynol	[M+H]+	267.17001	C17H24O	Long-chain fatty alcohols	UGJAEDFOKNAMQD-ZHACJKMWSA-N	OC(C#CC#CCC=CCCCCCCC)C=C
1435	4.44	285.15955	Tropisetron	[M+H]+	285.15976	C17H20N2O2	Indolecarboxylic acids and derivatives	ZNRGQMMCGHDTEI-UHFFFAOYNA-N	O=C(OC1CC2N(C)C(CC2)C1)C3=CNC=4C=CC=CC43
1436	4.445	273.14383	Sempervirine	[M+H]+	273.13861	C19H16N2	Beta carbolines	UQVUEULZDJRMJR-UHFFFAOYSA-N	C1CCC2=CC3=C4N=C5C=CC=CC5=C4C=CN3C=C2C1
1437	4.448	271.16354	19-Norandrosta-4,9-dien-3,17-dione	[M+H]+	271.16299	C18H22O2	Oxosteroids	BHTWZQKERRCPRZ-RYRKJORJSA-N	O=C1C=C2C(=C3CCC4(C(=O)CCC4C3CC2)C)CC1
1438	4.465	313.13956	6-(1,1-DIMETHYLALLYL)-2-(1-HYDROXY-1-METHYLETHYL)-2,3-DIHYDRO-7H-FURO[3,2-G]CHROMEN-7-ONE	[M-H]-	313.14453	C19H22O4	Psoralens	JCDLLLXYAICSQV-INIZCTEOSA-N	O=C1OC=2C=C3OC(CC3=CC2C=C1C(C=C)(C)C)C(O)(C)C
1439	4.467	299.13745	3-Hydroxy-3',4'-Dimethoxyflavone	[M+H]+	299.13629	C17H14O5	Flavonols	BXLAVJWSFYZDPF-UHFFFAOYSA-N	O=C1C(O)=C(OC=2C=CC=CC21)C=3C=CC(OC)=C(OC)C3
1440	4.471	327.1333	8,8-dimethyl-2-phenylpyrano[2,3-f]chromen-4-one	[M+H]+	327.13269	C20H16O3	Pyranoflavonoids	RBKWJAHRWPDNPM-UHFFFAOYSA-N	O=C1C=C(OC=2C1=CC=C3OC(C=CC32)(C)C)C=4C=CC=CC4
1441	4.484	305.15897	[(3aS,6Z,10Z,11aR)-6-(hydroxymethyl)-3-methylidene-2-oxo-3a,4,5,8,9,11a-hexahydrocyclodeca[b]furan-10-yl]methyl acetate	[M-H]-	305.15668	C17H22O5	Germacranolides and derivatives	XGODCDTVQAHYAH-DZPPMGJRSA-N	O=C1OC2C=C(COC(=O)C)CCC=C(CO)CCC2C1=C
1442	4.486	463.12106	(1S,3R,6R,7S,8S,9R,10S,13S,16S,17R)-8-tert-butyl-6,9,12,17-tetrahydroxy-16-methyl-2,4,14,19-	[M+H]+	463.12106	C20H24O11	Ginkgolides and bilobalides	AMOGMTLMADGEOQ-PDZSGAKNSA-N	O=C1OC2OC34C(=O)OC5C(O)C(C(C)(C)C)C2(C1O)C54C(O)C6OC(=O)C(C)C63O
1443	4.486	325.10226	Licarin A	[M-H]-	325.10001	C20H22O4	2-arylbenzofuran flavonoids	ITDOFWOJEDZPCF-AATRIKPKSA-N	OC1=CC=C(C=C1OC)C2OC=3C(OC)=CC(C=CC)=CC3C2C
1444	4.488	351.1058	1-(4-hydroxyphenyl)-3-[(2R,3R,4S,5S,6R)-3,4,5-trihydroxy-6-(hydroxymethyl)oxan-2-yl]oxypropan-1-one	[M+H]+	351.10504	C15H20O8	Fatty acyl glycosides of mono- and disaccharides	NPAQHLFPEOMKAL-UXXRCYHCSA-N	O=C(C1=CC=C(O)C=C1)CCOC2OC(CO)C(O)C(O)C2O
1445	4.49	411.18939	Picraline	[M+H]+	411.19101	C23H26N2O5	Corynanthean-type alkaloids	DXTJMQCRVFWNBD-FTHDSKJGSA-N	O=C(OCC1(C(=O)OC)C2C(=CC)CN3C4OC5(NC=6C=CC=CC6C51C4)C3C2)C
1446	4.494	165.05476	3-Phenyllactic acid	[M-H]-	165.05573	C9H10O3	Phenylpropanoic acids	VOXXWSYKYCBWHO-UHFFFAOYNA-N	O=C(O)C(O)CC=1C=CC=CC1
1447	4.528	281.06595	Feruloyl Lactate	[M-H]-	281.06479	C13H14O7	Ferulic acid and derivatives	ZWAYKTOAUPIGML-HWKANZRONA-N	COC1=CC=C(\C=C\C(=O)OCC(O)C(O)=O)C=C1O
1448	4.531	109.02869	CATECHOL	[M-H]-	109.029	C6H6O2	Catechols	YCIMNLLNPGFGHC-UHFFFAOYSA-N	OC=1C=CC=CC1O
1449	4.535	316.2113	[5-hydroxy-3-(hydroxymethyl)-2-oxo-6-propan-2-ylcyclohex-3-en-1-yl] 3-methylpentanoate	[M+H]+	316.21182	C16H26O5	Menthane monoterpenoids	BUOADWXGPJXKRM-UHFFFAOYSA-N	O=C(OC1C(=O)C(=CC(O)C1C(C)C)CO)CC(C)CC
1450	4.541	188.03438	4-HYDROXY-2-QUINOLINECARBOXYLIC ACID	[M-H]-	188.035	C10H7NO3	Quinoline carboxylic acids	HCZHHEIFKROPDY-UHFFFAOYSA-N	O=C(O)C1=CC(=O)C=2C=CC=CC2N1
1451	4.542	167.03738	3,4-Dihydroxyphenylacetic acid	[M-H]-	167.03499	C8H8O4	Catechols	CFFZDZCDUFSOFZ-UHFFFAOYSA-N	O=C(O)CC1=CC=C(O)C(O)=C1
1452	4.543	120.08041	Indoline	[M+H]+	120.08077	C8H9N	Indolines	LPAGFVYQRIESJQ-UHFFFAOYSA-N	C1=CC=C2C(=C1)CCN2
1453	4.543	208.09602	N-Acetylphenylalanine	[M+H]+	208.09682	C11H13NO3	Phenylalanine and derivatives	CBQJSKKFNMDLON-UHFFFAOYNA-N	O=C(O)C(N=C(O)C)CC=1C=CC=CC1
1454	4.544	275.01813	6-Phospho-D-gluconic acid	[M-H]-	275.01736	C6H13O10P	Monosaccharide phosphates	BIRSGZKFKXLSJQ-SQOUGZDYSA-N	O[C=H](COP(O)(O)=O)[C=H](O)[C=H](O)[C=H](O)C(O)=O
1455	4.545	429.20035	elegantissine	[M+H]+	429.20001	C23H28N2O6	Indolizidines	TTZWEOINXHJHCY-DWROLHPLSA-N	O=C(OC)C1=COC(C)C2CN3CCC4(C(=O)NC5=C(OC)C(OC)=CC=C54)C3CC12
1456	4.549	367.20892	3-[2-(6,7-dihydroxy-1,2,4a-trimethylspiro[3,4,6,7,8,8a-hexahydro-2H-naphthalene-5,2'-oxirane]-1-yl)ethyl]-2-hydroxy-2H-furan-5-one	[M+H]+	367.21152	C20H30O6	Butenolides	QMWJARHMJRTJMQ-UHFFFAOYSA-N	O=C1OC(O)C(=C1)CCC2(C)C(C)CCC3(C)C2CC(O)C(O)C43OC4
1457	4.549	534.34595	9-hydroxy-5b,8,11a-trimethyl-1-prop-1-en-2-yl-1,2,3,4,5,6,7,7a,9,10,11,11b,12,13,13a,13b-	[M+H]+	534.34253	C30H44O7	Triterpenoids	FTGKHGAVFGGOMW-UHFFFAOYSA-N	O=C(O)C12CCC(C(=C)C)C2C3CCC4C5(C)CCC(O)C(C(=O)O)(C)C5CCC4(C)C3(C(=O)O)CC1
1458	4.552	233.00833	FT-sulfone	[M-H]-	233.01009	C6H9F3O4S	PFSA	FTHRJQNFESLACH-UHFFFAOYSA-N	O=C(O)CCS(=O)(=O)CCC(F)(F)F
1459	4.562	562.25378	(S)-2-(((S)-7-acetamido-1,2,3-trimethoxy-9-oxo-5,6,7,9-tetrahydrobenzo[a]heptalen-10-yl)amino)-N-(4-methoxyphenyl)propanamide	[M+H]+	562.26001	C31H35N3O7	Alpha amino acid amides	RVLHHZKCFQNHII-XDHUDOTRSA-N	O=C1C=C2C(=CC=C1NC(C(=O)NC3=CC=C(OC)C=C3)C)C4=C(OC)C(OC)=C(OC)C=C4CCC2NC(=O)C
1460	4.563	322.09183	Metazachlor ESA	[M-H]-	322.0867	C14H17N3O4S	Anilides	IPVCSECPEVHQOV-UHFFFAOYSA-N	CC1=CC=CC(C)=C1N(CN1C=CC=N1)C(=O)CS(O)(=O)=O

1461	4.564	255.12265	DL-Liquiritigenin	[M-H]-	255.12166	C15H12O4	Flavanones	FURUXTVZLHCCNA-UHFFFAOYSA-N	O=C1C2=CC=C(O)C=C2OC(C3=CC=C(O)C=C3)C1
1462	4.568	217.09651	4-Formyl-antipyrine	[M+H]+	217.09715	C12H12N2O2	Phenylpyrazoles	QFYZFYDOEJZMDX-UHFFFAOYSA-N	CN1N(C(=O)C(C=O)=C1C)C1=CC=CC=C1
1463	4.573	357.17981	Arnicolide C	[M+H]+	357.17001	C19H26O5	Sesquiterpene lactones	WKUOPGZYLRFCHJ-APDQSUQKSA-N	O=C(OC1C2C(OC(=O)C2C)CC(C)C3C=CC(=O)C13C)C(C)C
1464	4.582	308.11105	Tolnaftate	[M+H]+	308.10999	C19H17NOS	Naphthalenes	FUSNMLFNXJSCDI-UHFFFAOYSA-N	S=C(OC=1C=CC=2C=CC=CC2C1)N(C3=CC=CC(=C3)C)C
1465	4.588	301.11749	Pesticide9_Desmedipham_C16H16N2O4_3-[(Ethoxycarbonyl)amino]phenyl phenylcarbamate	[M+H]+	301.11801	C16H16N2O4	Phenylcarbamic acid esters	WZJZMXBKUWKXTQ-UHFFFAOYSA-N	OC(=NC=1C=CC=CC1)OC=2C=CC=C(N=C(O)OCC)C2
1466	4.591	171.06548	1,4-Cyclohexanedicarboxylic acid	[M-H]-	171.06628	C8H12O4	Dicarboxylic acids and derivatives	PXGZQGDTEZPERC-UHFFFAOYSA-N	O=C(O)C1CCC(C(=O)O)CC1
1467	4.594	254.06645	methyl 2-[(2,3-dihydroxybenzoyl)amino]-3-hydroxypropanoate	[M-H]-	254.067	C11H13NO6	Hippuric acids and derivatives	NQIZIOGKLJAGSU-UHFFFAOYSA-N	O=C(OC)C(N=C(O)C=1C=CC=C(O)C1O)CO
1468	4.596	481.12912	Lusitanicoside	[M+H]+	481.13086	C21H30O10	Phenolic glycosides	DAELTTGCCPRYTP-ZLQZEYEISA-N	OC1C(O)C(OCC2OC(OC3=CC=C(C=C3)CC=C)C(O)C(O)C2O)OC(C)C1O
1469	4.603	299.10245	Scutellarein 4'-methyl ether	[M-H]-	299.10306	C16H12O6	4'-O-methylated flavonoids	XVMMEYCPXZYLAI-UHFFFAOYSA-N	O=C1C=C(OC2=CC(O)=C(O)C(O)=C12)C=3C=CC(OC)=CC3
1470	4.604	339.11752	Licodione base + 3O, 2Prenyl	[M-H]-	339.12079	C20H20O5	Prenylated licodiones	IXMVCKVQOAGJGJ-UHFFFAOYSA-N	O=C(C=C(O)C1=CC=C(O)C=C1)C=2C=C(C(O)=CC=2(O))CC=C(C)C
1471	4.615	153.01921	2,3-Dihydroxybenzoic acid	[M-H]-	153.01933	C7H6O4	Salicylic acids	GLDQAMYCGOIJDV-UHFFFAOYSA-N	O=C(O)C=1C=CC=C(O)C1O
1472	4.617	204.02948	XANTHURENIC ACID	[M-H]-	204.03	C10H7NO4	Quinoline carboxylic acids	FBZONXHGGPHHIY-UHFFFAOYSA-N	O=C(O)C1=CC(=O)C=2C=CC=C(O)C2N1
1473	4.618	207.0112	2-Naphthalenesulfonic acid	[M-H]-	207.01213	C10H8O3S	2-naphthalene sulfonates	KVBGVZZKJNLNJU-UHFFFAOYSA-N	OS(=O)(=O)C1=CC2=CC=CC=C2C=C1
1474	4.62	317.11447	Rhodocladonic Acid	[M-H]-	317.11322	C15H10O8	Naphthofurans	XKWWCTISHMFHJB-UHFFFAOYSA-N	O=C1C(=O)C=2C(O)=C3C(O)=C(OC3=CC2C(O)=C1OC)C(=O)C
1475	4.623	217.10738	Hydroxysebacic acid	[M-H]-	217.10789	C10H18O5	Organic acids	OQYZCCKCJQWHIE-UHFFFAOYNA-N	O=C(O)CCCCCCC(O)CC(=O)O
1476	4.629	269.11374	Galangin	[M-H]-	269.11237	C15H10O5	Flavonols	VCCRNZQBSJXYJD-UHFFFAOYSA-N	O=C1C(O)=C(OC=2C=C(O)C=C(O)C12)C=3C=CC=CC3
1477	4.635	301.09186	2-(3-hydroxy-5-methoxyphenoxy)-6-(hydroxymethyl)oxane-3,4,5-triol	[M-H]-	301.0929	C13H18O8	Phenolic glycosides	SJBWDSQCMPAGHA-UHFFFAOYSA-N	OC=1C=C(OC)C=C(OC2OC(CO)C(O)C(O)C2O)C1
1478	4.648	363.23743	2-[(2E)-3,7-dimethylocta-2,6-dienyl]-3-methoxy-5-(2-phenylethyl)phenol	[M-H]-	363.23294	C25H32O2	Stilbenes	ZDSBUUDXDQSCSK-DEDYPNTBSA-N	OC=1C=C(C=C(OC)C1CC=C(C)CCC=C(C)C)CCC=2C=CC=CC2
1479	4.651	587.29132	[(2R,3S,4S,5R,6S)-6-[3,5-dihydroxy-4-[3-(4-hydroxyphenyl)propanoyl]phenoxy]-3,4,5-trihydroxyoxan-2-yl]methyl 3,4,5-	[M-H]-	587.29706	C28H28O14	Flavonoid O-glycosides	YPFUHFNAEWURDN-ZKPBLSLZSA-N	O=C(OCC1OC(OC2=CC(O)=C(C(O)=C2)C(=O)CCC3=CC=C(O)C=C3)C(O)C(O)C1O)C4=CC(O)=C(O)C(O)=C4
1480	4.653	370.07071	Pesticide6_Diniconazole_C15H17Cl2N3O_(1E)-1-(2,4-Dichlorphenyl)-4,4-dimethyl-2-(1H-1,2,4-triazol-1-yl)pent-1-en-3-ol	[M-H]-	370.073	C15H17Cl2N3O	Cinnamyl alcohols	FBOUIAKEJMZPQG-AWNIVKPZSA-N	ClC1=CC=C(C=C(N2N=CN=C2)C(O)C(C)(C)C)C(Cl)=C1
1481	4.658	224.12704	4-Hydroxybenzoylcholine	[M+H]+	224.12756	C12H18NO3	Benzoic acid esters	BAPAICNRGIBFJT-UHFFFAOYSA-O	C[N+](C)(C)CCOC(O)=C1C=CC(=O)C=C1
1482	4.658	315.11966	Pinoquercetin	[M-H]-	315.11609	C16H12O7	Flavonols	DTFXGVGIKNSCQQ-UHFFFAOYSA-N	O=C1C(O)=C(OC2=CC(O)=C(C(O)=C12)C)C=3C=CC(O)=C(O)C3
1483	4.66	503.24872	4-[(E)-3-[(2R,3R,4S,5S,6R)-3-[(2S,3R,4R)-3,4-dihydroxy-4-(hydroxymethyl)oxolan-2-yl]oxy-4,5-dihydroxy-6-(hydroxymethyl)oxan-2-	[M+H]+	503.24869	C24H38O11	Fatty acyl glycosides of mono- and disaccharides	IDPRQTHSAHGSDW-BTOUMFIXSA-N	O=C1C=C(C)C(C=CC(OC2OC(CO)C(O)C(O)C2OC3OCC(O)(CO)C3O)C)C(C)(C)C1
1484	4.667	279.14386	3-[(3aS,8R,8aR)-8-hydroxy-6,8-dimethyl-3-methylidene-2-oxo-3a,4,5,8a-tetrahydrocyclohepta[b]furan-7-yl]propanoic acid	[M-H]-	279.14893	C15H20O5	Terpene lactones	PPUVIGSDWLRHCY-PSOPSSQASA-N	O=C1OC2C(C1=C)CCC(=C(CCC(=O)O)C2(O)C)C
1485	4.668	457.19702	2,7,7,11,15,17-hexamethyl-18-methylidene-5,13,16-trioxo-6,14-dioxatetracyclo[9.8.0.0?,?.0??,??]nonadec-3-en-10-yl acetate	[M-H]-	457.19882	C26H34O7	Naphthopyrans	JFYYCQXZVIOBKU-UHFFFAOYSA-N	O=C1OC(C)(C)C2CC(OC(=O)C)C3(C)C4C(=O)OC(C(=O)C4(C(=C)CC3C2(C=C1)C)C)C
1486	4.669	220.0966	Aniracetam	[M+H]+	220.097	C12H13NO3	Benzoic acids and derivatives	ZXNRTKGTQJPIJK-UHFFFAOYSA-N	O=C(C1=CC=C(OC)C=C1)N2C(=O)CCC2
1487	4.67	230.14677	N-cyano-N'-(1,1-dimethylpropyl)-N'-3-pyridinyl-guanidine	[M-H]-	230.14111	C12H17N5	Pyridines and derivatives	HKZNADVVGXKQDL-UHFFFAOYSA-N	N#CN=C(NC=1C=NC=CC1)NC(C)(C)CC
1488	4.672	481.19406	[3-(13-methyltetradecyl)-5-sulfooxyphenyl] hydrogen sulfate	[M+H]+	481.19241	C21H36O8S2	Phenylsulfates	NGWLEVXPPLCHNC-UHFFFAOYSA-N	O=S(=O)(O)OC=1C=C(OS(=O)(=O)O)C=C(C1)CCCCCCCCCCCCC(C)C
1489	4.678	271.18942	(E)-2-decylpent-2-enedioic acid	[M+H]+	271.19037	C15H26O4	Long-chain fatty acids	BWSQDEWKZUMVIG-ACCUITESSA-N	O=C(O)C(=CCC(=O)O)CCCCCCCCCC
1490	4.681	572.34351	Veratrosine	[M+H]+	572.34998	C33H49NO7	Fluorenes	WXQHVBNTINGJJR-UHFFFAOYSA-N	OCC1OC(OC2CC3=CCC4C5=CC=C(C(=C5CC4C3(C)CC2)C)C(C)C6NCC(C)CC6O)C(O)C(O)C1O
1491	4.685	467.28165	10-hydroxyusambarine	[M+2H]2+	467.28	C30H34N4O	Harmala alkaloids	QYONROBEKPAESK-YBTNASEBSA-N	OC=1C=CC=2NC3=C(C2C1)CCN4CC(C=C)C(CC5C=6NC=7C=CC=CC7C6CCN5C)CC34
1492	4.687	589.30579	12-O-[deca-2Z,4E,6E-trienoyl]-13-isobutyroyloxy-4-deoxyphorbol	[M+H]+	589.31	C34H46O7	Tigliane and ingenane diterpenoids	WHPZSYIEWQPEHY-OMZYKPILSA-N	O=C(OC1C(C)C2(O)C3C=C(C(=O)C3CC(=CC2C4C1(OC(=O)C(C)C)C4(C)C)CO)C)C=CC=CC=CCCC
1493	4.695	578.37183	4-Ketoalloxanthin	[M+H]+	578.37543	C40H50O3	Diterpenoids	MMOXINAUTUSUTD-RREWOKSTSA-N	C\C(\C=C\C=C(/C)C#CC1=C(C)C[C=H](O)CC1(C)C)=C/C=C/C=C(\C)/C=C/C=C(\C)C#CC1=C(C)C(=O)[C=H](O)CC1(C)C
1494	4.695	249.09604	Carnosine	[M+H]+	249.09581	C9H14N4O3	Hybrid peptides	CQOVPNPJLQNMDC-ZETCQYMHSA-N	O=C(O)C(N=C(O)CCN)CC1=CN=CN1
1495	4.696	182.11682	(S)-4-methyl-2-(1H-pyrrol-1-yl)pentanoic acid	[M+H]+	182.11	C10H15NO2	Alpha amino acids and derivatives	CCIUIWFXXDCCOJ-VIFPVBQESA-N	O=C(O)C(N1C=CC=C1)CC(C)C
1496	4.706	211.06017	2,4,5-trimethoxybenzoic acid	[M-H]-	211.06	C10H12O5	P-methoxybenzoic acids and derivatives	KVZUCOGWKYOPID-UHFFFAOYSA-N	O=C(O)C1=CC(OC)=C(OC)C=C1OC
1497	4.706	264.08649	Albendazole	[M-H]-	264.08121	C12H15N3O2S	2-benzimidazolylcarbamic acid esters	HXHWSAZORRCQMX-UHFFFAOYSA-N	CCCSC=1C=CC2=C(C1)NC(=N2)N=C(O)OC
1498	4.721	204.06543	Cinnamoylglycine	[M-H]-	204.0666	C11H11NO3	N-acyl-alpha amino acids	YAADMLWHGMUGQL-VOTSOKGWSA-N	O=C(O)CN=C(O)C=CC=1C=CC=CC1
1499	4.721	302.1593	Phenylethyl 2-Glucoside	[M+H]+	302.15979	C14H20O6	O-glycosyl compounds	MLRIJUWUQTVDQE-RKQHYHRCSA-N	OCC1OC(OCCC=2C=CC=CC2)C(O)C(O)C1O
1500	4.728	252.12213	Alibendol	[M+H]+	252.123	C13H17NO4	Salicylamides	UMJHTFHIQDEGKB-UHFFFAOYSA-N	O=C(NCCO)C1=CC(=CC(OC)=C1O)CC=C
1501	4.728	233.09171	Nalidixic acid (NegGram)	[M+H]+	233.092	C12H12N2O3	Naphthyridine carboxylic acids and derivatives	MHWLWQUZZRMNGJ-UHFFFAOYSA-N	O=C(O)C1=CN(C2=NC(=CC=C2C1=O)C)CC
1502	4.731	273.16623	(E)-5-hydroxy-3-isobutyl-6-(3-methylbenzylidene)-1,6-dihydropyrazin-2(3H)-one	[M+H]+	273.16	C16H20N2O2	Alpha amino acids and derivatives	VVXVZIGIGDNTDE-NTEUORMPSA-N	O=C1NC(=CC2=CC=CC(=C2)C)C(O)=NC1CC(C)C
1503	4.734	437.23785	5-O-methyllicoricidin	[M-H]-	437.23334	C27H34O5	7-O-methylated isoflavonoids	GDAAEAXMNLVRCZ-SFHVURJKSA-N	OC1=CC=C(C(O)=C1CC=C(C)C)C2COC3=CC(OC)=C(C(OC)=C3C2)CC=C(C)C

1504	4.736	250.10771	Dimethachlor OXA	[M-H]-	250.10847	C13H17NO4	Alpha amino acids and derivatives	MHGMSAFPNAKIRZ-UHFFFAOYSA-N	COCCN(C(=O)C(O)=O)C1=C(C)C=CC=C1C
1505	4.741	308.11472	CP_M308a	[M+H]+	308.11603	C15H18ClN3O2	Chlorobenzenes	IVFRBVSPAOYOOK-UHFFFAOYNA-N	C1CC1C(CO)C(CN2C=NC=N2)(C3=CC=C(C=C3)Cl)O
1506	4.741	326.12619	Monocrotaline	[M+H]+	326.12665	C16H23NO6	Pyrrolizines	QVCMHGGNRFRMAD-XFGHUUIASA-N	O=C1OC2CCN3CC=C(COC(=O)C(O)(C)C(O)(C)C1C)C32
1507	4.741	182.08051	Tyr	[M+H]+	182.08118	C9H11NO3	Tyrosine and derivatives	OUYCCCASQSFEME-QMMMGPOBSA-N	N[C=H](CC1=CC=C(O)C=C1)C(O)=O
1508	4.744	199.086	Harmol Hydrochloride	[M+H]+	199.08658	C12H10N2O	Harmala alkaloids	LBBJNGFCXDOYMQ-UHFFFAOYSA-N	O=C1C=CC=2C(=C1)NC=3C2C=CNC3C
1509	4.775	176.03455	FORMYL-L-METHIONYL PEPTIDE	[M-H]-	176.03799	C6H11NO3S	Methionine and derivatives	PYUSHNKNPOHWEZ-UHFFFAOYSA-N	O=CNC(C(=O)O)CCSC
1510	4.784	369.09335	paulownin	[M-H]-	369.09796	C20H18O7	Furanoid lignans	CAQZFLPWHBKTTR-WNISUXOKSA-N	OC12COC(C3=CC=C4OCOC4=C3)C2COC1C5=CC=C6OCOC6=C5
1511	4.787	291.12567	curvularin	[M-H]-	291.12378	C16H20O5	Macrolides and analogues	VDUIGYAPSXCJFC-UHFFFAOYSA-N	O=C1OC(C)CCCCCC(=O)C=2C(O)=CC(O)=CC2C1
1512	4.792	308.11157	altenusin	[M+H]+	308.11285	C15H14O6	Biphenyls and derivatives	ADPBTBPPIIKLEH-UHFFFAOYSA-N	O=C(O)C1=C(O)C=C(OC)C=C1C=2C=C(O)C(O)=CC2C
1513	4.794	437.16907	Picropodophyllin	[M+H]+	437.16586	C22H22O8	Podophyllotoxins	YJGVMLPVUAXIQN-HAEOHBJNSA-N	O=C1OCC2C(O)C3=CC=4OCOC4C=C3C(C5=CC(OC)=C(OC)C(OC)=C5)C12
1514	4.804	326.09903	Tumonoic acid E	[M+H]+	326.10001	C18H31NO4	Proline and derivatives	UUFOMZNZQYHUJH-XTVPCCRVSA-N	O=C(O)C1N(C(=O)C(C)C(O)C(=CC(C)CCCC)C)CCC1
1515	4.835	305.09125	Oxypeucedanin hydrate	[M+H]+	305.10001	C16H16O6	Psoralens	PEWFWDOPJISUOK-CYBMUJFWSA-N	O=C1OC=2C=C3OC=CC3=C(OCC(O)C(O)(C)C)C2C=C1
1516	4.844	208.13234	Benzoylcholine	[M+H]+	208.13266	C12H18NO2	Benzoic acid esters	HOPVGFKDVOOCHD-UHFFFAOYSA-N	C[N+](C)(C)CCOC(=O)C1=CC=CC=C1
1517	4.847	281.09552	culpin	[M+2H]2+	281.09384	C16H18O2	Prenylated hydroquinones	QMYWKFZKRYCUMA-UHFFFAOYSA-N	OC1=CC(=C(O)C=C1C#CC(=C)C)CC=C(C)C
1518	4.848	283.12842	biochanin A	[M-H]-	283.13037	C16H12O5	4'-O-methylisoflavones	WUADCCWRTIWANL-UHFFFAOYSA-N	O=C1C(=COC=2C=C(O)C=C(O)C12)C=3C=CC(OC)=CC3
1519	4.852	621.18243	5-[4,5-dihydroxy-6-(hydroxymethyl)-3-(3,4,5-trihydroxyoxan-2-yl)oxyoxan-2-yl]oxy-7,8-dimethoxy-3-(4-methoxyphenyl)chromen-4-one	[M-H]-	621.1825	C29H34O15	Isoflavonoid O-glycosides	NGUSMSQYMDPBGF-UHFFFAOYSA-N	O=C1C(=COC=2C(OC)=C(OC)C=C(OC3OC(CO)C(O)C(O)C3OC4OCC(O)C(O)C4O)C12)C=5C=CC(OC)=CC5
1520	4.853	539.22491	methyl (1S,2R,4R,8R,9S,10R,13R,15R)-2-(acetyloxy)-13-(furan-3-yl)-9-(2-methoxy-2-oxoethyl)-4,8,10,12-tetramethyl-7-oxo-16-	[M-H]-	539.22864	C30H36O9	Limonoids	NHOIBRJOQAYBJT-WILUFPLZSA-N	O=C(OC1C2OC3C(=C(C)C(C4=COC=C4)C3)C2(C)C(CC(=O)OC)C5(C(=O)C=CC(C(=O)OC)(C)C15)C)C
1521	4.854	543.21814	methyl 3-(1-(2-(1H-indol-3-yl)ethyl)-2-hydroxy-6-methyl-4-oxo-1,4-dihydropyridin-3-yl)-3-(3,4,5-trimethoxyphenyl)propanoate	[M+H]+	543.21002	C29H32N2O7	3-alkylindoles	OUBFEEIFMSTDPQ-UHFFFAOYSA-N	O=C1C=C(N(C(O)=C1C(C2=CC(OC)=C(OC)C(OC)=C2)CC(=O)OC)CCC3=CNC=4C=CC=CC43)C
1522	4.854	664.09021	NICOTINAMIDE HYPOXANTHINE DINUCLEOTIDE	[M-H]-	664.09302	C21H26N6O15P2	(5'->5')-dinucleotides	DGVSIBCCYUVRNA-UHFFFAOYSA-N	O=C1N=CNC2=C1N=CN2C3OC(COP(=O)(O)OP(=O)([O-])OCC4OC([N+]=5C=CC=C(C5)C(=O)N)C(O)C4O)C(O)C3O
1523	4.858	186.11198	3-(2-amino-2-oxoethyl)-5-methyl-hexanoic acid	[M-H]-	186.11099	C9H17NO3	Medium-chain fatty acids	NPDKTSLVWGFPQG-UHFFFAOYSA-N	O=C(O)CC(CC(=N)O)CC(C)C
1524	4.864	280.09427	9-Methoxy-2,2-dimethyl-2,6-dihydro-pyrano[3,2-c]quinolin-5-one	[M+2H]2+	280.09439	C15H15NO3	Pyranoquinolines	IXRKDGGGFFCRIR-UHFFFAOYSA-N	OC1=NC=2C=CC(OC)=CC2C=3OC(C=CC13)(C)C
1525	4.867	178.04965	Alliin	[M+H]+	178.05	C6H11NO3S	L-alpha-amino acids	XUHLIQGRKRUKPH-JDLXGLENSA-N	O=C(O)C(N)CS(=O)CC=C
1526	4.869	300.19412	(5S)-5-hydroxy-1,7-diphenylheptan-3-one	[M+H]+	300.1958	C19H22O2	Linear diarylheptanoids	CCNKTMMNRPJQHV-SFHVURJKSA-N	O=C(CCC=1C=CC=CC1)CC(O)CCC=2C=CC=CC2
1527	4.871	362.18076	2-[1-hydroxy-1-(4-methoxyphenyl)propan-2-yl]oxy-6-(hydroxymethyl)oxane-3,4,5-triol	[M+H]+	362.18094	C16H24O8	O-glycosyl compounds	JYWSARFDLXXOHT-UHFFFAOYSA-N	OCC1OC(OC(C)C(O)C2=CC=C(OC)C=C2)C(O)C(O)C1O
1528	4.873	285.13025	3-Hexen-1-ol O-b-D-glucopyranoside	[M+H]+	285.13	C12H22O6	Fatty acyl glycosides of mono- and disaccharides	OZIPFYKAIOOVEJ-ODWUMMNUSA-N	OCC1OC(OCCC=CCC)C(O)C(O)C1O
1529	4.875	479.18799	4-(2-Nitroethyl)phenyl 6-O-?-D-xylopyranosyl-?-D-glucopyranoside	[M+H]+	479.18713	C19H27NO12	Phenolic glycosides	ZHVLPKFAODFQST-BMVMOQKNSA-N	O=N(=O)CCC1=CC=C(OC2OC(COC3OCC(O)C(O)C3O)C(O)C(O)C2O)C=C1
1530	4.894	423.12613	2-[[(2R,3S)-2-(3,4-dihydroxyphenyl)-3,5-dihydroxy-3,4-dihydro-2H-chromen-7-yl]oxy]oxane-3,4,5-triol	[M+H]+	423.1264	C20H22O10	Flavonoid-7-O-glycosides	UQKKDJWFQBNZBJ-PTVYBAHFSA-N	OC1=CC=C(C=C1O)C2OC3=CC(OC4OCC(O)C(O)C4O)=CC(O)=C3CC2O
1531	4.905	430.30078	PF-1052_130138	[M+H]+	430.29599	C26H39NO4	N-alkylpyrrolidines	NPWKEUKXVOMELT-LNVKXUELSA-N	O=C1C(C(=O)C(N1C)C(C)CC)=C(O)C2C(C=CC3CC(C)CC(C)C32)C4(OC4C)C
1532	4.912	383.13275	Ochrephilone	[M+H]+	383.13297	C23H26O5	Cyclohexenones	GAIVGINVGXHEIA-ZAAJWPAISA-N	O=C1OC2(C(=O)C=C3C=C(OC=C3C2C1C(=O)C)C=CC(=CC(C)CC)C)C
1533	4.917	428.21677	Pesticide2_Benfuracarb_C20H30N2O5S_Oncol	[M+H]+	428.22198	C20H30N2O5S	Coumarans	FYZBOYWSHKHDMT-UHFFFAOYSA-N	O=C(OC1=CC=CC2=C1OC(C)(C)C2)N(SN(CCC(=O)OCC)C(C)C)C
1534	4.926	197.11649	Loliolide	[M+H]+	197.117	C11H16O3	Benzofurans	XEVQXKKKAVVSMW-UHFFFAOYSA-N	O=C1OC2(C(=C1)C(C)(C)CC(O)C2)C
1535	4.928	193.04929	Monoethyl phthalate	[M-H]-	193.05063	C10H10O4	Benzoic acid esters	YWWHKOHZGJFMIE-UHFFFAOYSA-N	CCOC(C1=CC=CC=C1C(=O)O)=O
1536	4.932	631.17334	4-(3-Oxobutyl)phenyl 6-O-[(2E)-3-phenyl-2-propenoyl]-2-O-(3,4,5-trihydroxybenzoyl)-beta-D-glucopyranoside	[M+H]+	631.17999	C32H32O12	Phenolic glycosides	AHLBWJHZDLMJOJ-SIHJTGPPSA-N	O=C(OCC1OC(OC2=CC=C(C=C2)CCC(=O)C)C(OC(=O)C3=CC(O)=C(O)C(O)=C3)C(O)C1O)C=CC=4C=CC=CC4
1537	4.94	331.1752	(3R,4S,5S,6R)-2-(6-hydroxy-2,6-dimethylocta-2,7-dienoxy)-6-(hydroxymethyl)oxane-3,4,5-triol	[M-H]-	331.17621	C16H28O7	Fatty acyl glycosides of mono- and disaccharides	WEHZDNHJZBEGME-SPGNZJCFSA-N	OCC1OC(OCC(=CCCC(O)(C=C)C)C)C(O)C(O)C1O
1538	4.943	451.22998	[4,8-dihydroxy-3-(3-methoxy-3-oxoprop-1-en-2-yl)-8a-methyl-5-oxo-1,2,3,4,4a,6,7,8-octahydronaphthalen-2-yl] 3,4-dihydroxy-2-	[M+H]+	451.2262	C20H28O9	Beta hydroxy acids and derivatives	NOGWCMMYTAXOCY-UHFFFAOYSA-N	O=C(OC)C(=C)C1C(O)C2C(=O)CCC(O)C2(C)CC1OC(=O)C(=C)C(O)CO
1539	4.944	666.42291	(4aS,6aR,6bR,10S,12aR)-1,2,6b,9,9,12a-hexamethyl-10-[(2R,3R,4S,5S,6R)-3,4,5-trihydroxy-6-(hydroxymethyl)oxan-2-yl]oxy-	[M+H]+	666.42114	C36H56O10	Triterpene saponins	AXNXSFBKZQIMPF-RIBCLQTCSA-N	O=C(O)C12C(=CCC3C4(C)CCC(OC5OC(CO)C(O)C(O)C5O)C(C)(C)C4CCC31C)C6C(C)C(C)CCC6(C(=O)O)CC2
1540	4.945	471.24402	3Alpha-Hydroxy-3-Deoxyangolensic Acid Methyl Ester	[M-H]-	471.24536	C27H36O7	Delta valerolactones	QFRUZVNPYYYLAN-UOICJKMOSA-N	O=C(OC)CC1C(C)(C)C(O)CC2OC34C(=C)C(CCC4(C)C(OC(=O)C3)C5=COC=C5)C21C
1541	4.945	478.18442	Pralatrexate(Folotyn)	[M+H]+	478.18301	C23H23N7O5	Glutamic acid and derivatives	OGSBUKJUDHAQEA-WMCAAGNKSA-N	O=C(O)CCC(NC(=O)C1=CC=C(C=C1)C(CC#C)CC=2N=C3C(=NC2)N=C(N=C3N)N)C(=O)O
1542	4.946	347.12262	Nifedipine	[M+H]+	347.12375	C17H18N2O6	Dihydropyridinecarboxylic acids and derivatives	HYIMSNHJOBLJNT-UHFFFAOYSA-N	O=C(OC)C1=C(NC(=C(C(=O)OC)C1C=2C=CC=CC2N(=O)=O)C)C
1543	4.952	148.03983	4-Hydroxymandelonitrile	[M-H]-	148.04041	C8H7NO2	1-hydroxy-2-unsubstituted benzenoids	HOOOPXDSCKBLFG-UHFFFAOYNA-N	N#CC(O)C1=CC=C(O)C=C1
1544	4.97	231.1232	Melatonin	[M-H]-	231.12207	C13H16N2O2	3-alkylindoles	DRLFMBDRBRZALE-UHFFFAOYSA-N	OC(=NCCC1=CNC=2C=CC(OC)=CC21)C
1545	4.974	601.3418	Desferrioxamine E	[M+H]+	601.34198	C27H48N6O9	Macrolactams	NHKCCADZVLTPPO-UHFFFAOYSA-N	O=C1N(O)CCCCCN=C(O)CCC(=O)N(O)CCCCCN=C(O)CCC(=O)N(O)CCCCCN=C(O)CC1
1546	4.98	327.04868	Acid Orange 7 (free acid)	[M-H]-	327.04449	C16H12N2O4S	Naphthols and derivatives	RDUJRVXKAIVTDH-UHFFFAOYSA-N	C1=CC=C2C(=C1)C=CC(=C2N=NC3=CC=C(C=C3)S(O)(=O)=O)O

1547	4.98	361.1012	Demethoxycurcumin	[M+H]+	361.10001	C20H18O5	Curcuminoids	HJTVQHVGMGKONQ-LUZURFALSA-N	O=C(C=CC1=CC=C(O)C=C1)CC(=O)C=CC2=CC=C(O)C(OC)=C2
1548	4.985	262.09906	(E)-3-(3-chlorobut-2-en-1-yl)-2,8-dimethylquinolin-4-ol	[M+H]+	262.10001	C15H16ClNO	Hydroquinolones	YRXDKQKYWMHRSY-JXMROGBWSA-N	ClC(=CCC1=C(O)C=2C=CC=C(C2N=C1C)C)C
1549	4.993	268.16434	Azacyclonol	[M+H]+	268.17001	C18H21NO	Diphenylmethanes	ZMISODWVFHHWNR-UHFFFAOYSA-N	OC(C=1C=CC=CC1)(C=2C=CC=CC2)C3CCNCC3
1550	4.994	440.20276	Protostemonine	[M+H]+	440.20001	C23H31NO6	Stemoamide-type alkaloids	JDGNFRYDHRYXNL-ROHJGGRPSA-N	O=C1OC(C(OC)=C1C)=C2OC3CCCN4C(CCC4C3C2C)C5OC(=O)C(C)C5
1551	4.999	150.09082	2`-Methylacetanilide	[M+H]+	150.09134	C9H11NO	Acetanilides	BPEXTIMJLDWDTL-UHFFFAOYSA-N	CC1=CC=CC=C1N=C(C)O
1552	5.003	423.29526	licoricidin	[M-H]-	423.2905	C26H32O5	5-O-methylated isoflavonoids	GBRZTUJCDFSIHM-KRWDZBQOSA-N	OC1=CC=C(C(O)=C1CC=C(C)C)C2COC3=CC(O)=C(C(OC)=C3C2)CC=C(C)C
1553	5.015	357.16394	Hycanthone	[M+H]+	357.16312	C20H24N2O2S	Thiochromenes	MFZWMTSUNYWVBU-UHFFFAOYSA-N	CCN(CC)CCNC1=C2C(=O)C3=CC=CC=C3SC2=C(CO)C=C1
1554	5.017	146.05962	Indole-3-carboxyaldehyde	[M+H]+	146.06004	C9H7NO	Indoles	OLNJUISKUQQNIM-UHFFFAOYSA-N	O=CC1=CNC2=CC=CC=C12
1555	5.017	524.26886	sodium (2R)-2-((2R,5S,6R)-6-((3E,5E)-6-((3aR,4S,7aS)-4-(1H-pyrrole-2-carbonyl)-2,3,3a,4,5,7a-hexahydro-1H-inden-5-yl)hexa-3,5-dien-3-yl)-5-	[M+H]+	524.27002	C30H40NNaO4	Aryl alkyl ketones	BPPSGNVGAIGXQI-AREVZEEPSA-M	[Na+].O=C([O-])C(CC)C1OC(C(=CC=CC2C=CC3CCCC3C2C(=O)C4=CC=CN4)CC)C(C
1556	5.018	361.20032	Cortisone	[M+H]+	361.20093	C21H28O5	21-hydroxysteroids	MFYSYFVPBJMHGN-ZPOLXVRWSA-N	O=C1C=C2CCC3C(C(=O)CC4(C)C3CCC4(O)C(=O)CO)C2(C)CC1
1557	5.021	407.20593	[6,10a-dihydroxy-4-(hydroxymethyl)-4,7,11b-trimethyl-9-oxo-1,2,3,4a,5,6,6a,7,11,11a-decahydronaphtho[2,1-f][1]benzofuran-5-yl]	[M-H]-	407.20752	C22H32O7	Naphthofurans	GWEGFCVAOPNYPT-UHFFFAOYSA-N	O=C1OC2(O)C(=C1)C(C)C3C(O)C(OC(=O)C)C4C(C)(CO)CCCC4(C)C3C2
1558	5.021	379.14713	Glyasperin C	[M+H]+	379.14999	C21H24O5	5-O-methylated isoflavonoids	RCZMWVKBVFOCEE-UHFFFAOYSA-N	OC1=CC=C(C(O)=C1)C2COC3=CC(O)=C(C(OC)=C3C2)CC=C(C)C
1559	5.023	609.33606	Reserpine	[M+H]+	609.33337	C33H40N2O9	Yohimbine alkaloids	QEVHRUUCFGRFIF-MDEJGZGSSA-N	O=C(OC1CC2CN3CCC=4C=5C=CC(OC)=CC5NC4C3CC2C(C(=O)OC)C1OC)C6=CC(OC)=C(OC)C(OC)=C6
1560	5.028	361.13901	Nitrendipine	[M+H]+	361.13901	C18H20N2O6	Dihydropyridinecarboxylic acids and derivatives	PVHUJELLJLJGLN-UHFFFAOYSA-N	O=C(OC)C1=C(NC(=C(C(=O)OCC)C1C=2C=CC=C(C2)[N+](=O)[O-])C)C
1561	5.03	144.04489	4-Hydroxyquinoline	[M-H]-	144.04549	C9H7NO	Hydroquinolones	PMZDQRJGMBOQBF-UHFFFAOYSA-N	O=C1C=CNC2=CC=CC=C12
1562	5.031	206.11729	Dehydrosalsolidine	[M+H]+	206.11755	C12H15NO2	Others	VASUQTGZAPZKFK-UHFFFAOYSA-N	COC1=CC2=C(C=C1OC)C(C)=NCC2
1563	5.036	269.12769	(3aS,5aS,9bR)-5a,9-dimethyl-3-methylidene-3a,4,6,7,8,9b-hexahydrobenzo[g][1]benzofuran-2,5-dione	[M+H]+	269.12912	C15H18O3	Eudesmanolides, secoeudesmanolides, and	CPHPAWJXZHHPEN-PSOPSSQASA-N	O=C1OC2C3=C(C)CCCC3(C(=O)CC2C1=C)C
1564	5.036	221.08017	Dihydropiplartine	[M+H]+	221.078	C17H21NO5	Cinnamic acids and derivatives	HPXUNEGPCPQBQY-BQYQJAHWSA-N	O=C(C=CC1=CC(OC)=C(OC)C(OC)=C1)N2C(=O)CCCC2
1565	5.04	427.16019	(2R,3S,4S,5R,6R)-2-[[(2R,3S,4S,5R)-3,4-dihydroxy-5-(hydroxymethyl)oxolan-2-yl]oxymethyl]-6-[(E)-3-phenylprop-2-enoxy]oxane-3,4,5-triol	[M-H]-	427.16095	C20H28O10	Fatty acyl glycosides of mono- and disaccharides	IEBFEMIXXHIISM-RJSCAXEBSA-N	OCC1OC(OCC2OC(OCC=CC=3C=CC=CC3)C(O)C(O)C2O)C(O)C1O
1566	5.055	195.06493	trans-4-Hydroxy-3-methoxycinnamate	[M+H]+	195.06519	C10H10O4	Hydroxycinnamic acids	KSEBMYQBYZTDHS-HWKANZROSA-N	COC1=C(O)C=CC(\C=C\C(O)=O)=C1
1567	5.062	305.18475	Piperanine	[M+H]+	305.18594	C17H21NO3	Benzodioxoles	QHWOFMXDKFORMO-XVNBXDOJSA-N	O=C(C=CCCC1=CC=C2OCOC2=C1)N3CCCCC3
1568	5.068	276.08633	ACTIPHENOL	[M+H]+	276.08603	C15H17NO4	Alkyl-phenylketones	YTLMIHBTPWTPEV-UHFFFAOYSA-N	O=C1N=C(O)CC(C1)CC(=O)C=2C=C(C=C(C2O)C)C
1569	5.075	218.21074	Triacetin	[M+H]+	218.21001	C9H14O6	Triacylglycerols	URAYPUMNDPQOKB-UHFFFAOYSA-N	O=C(OCC(OC(=O)C)COC(=O)C)C
1570	5.084	415.19446	Hexose + C13H19O (isomer of 1062)	[M-H]-	415.19489	C19H30O7	Terpene glycosides	SZOPSAFLRCYJCX-UHFFFAOYNA-N	O=C2C=C(C)C(C=CC(OC1OC(CO)C(O)C(O)C1(O))C)C(C)(C)C2
1571	5.092	470.2735	Hypaconine	[M+H]+	470.27399	C24H39NO8	Aconitane-type diterpenoid alkaloids	BQTYHFZQSAKNQU-UHFFFAOYSA-N	OC1C(OC)C2(O)CC3C(C2O)C1(O)C4C(OC)C5C6(COC)CN(C)C4C35C(OC)CC6
1572	5.094	197.16608	(1R,3r,8S,9r)-1,8-dimethyl-3,6-diazatricyclo[4.3.1.1(3,8)]undecan-9-ol	[M+H]+	197.164	C11H20N2O	1,4-diazepanes	JNARJHDJLSPXGM-UHFFFAOYSA-N	OC1C2(C)CN3CCN(C2)CC1(C)C3
1573	5.121	179.06984	Coniferylaldehyde	[M+H]+	179.07027	C10H10O3	Methoxyphenols	DKZBBWMURDFHNE-UHFFFAOYSA-N	O=CC=CC1=CC=C(O)C(OC)=C1
1574	5.136	345.14279	Tyr-Tyr	[M+H]+	345.1445	C18H20N2O5	Dipeptides	JAQGKXUEKGKTKX-UHFFFAOYNA-N	O=C(O)C(N=C(O)C(N)CC1=CC=C(O)C=C1)CC2=CC=C(O)C=C2
1575	5.143	164.10643	N-acetyl-2-phenylethylamine	[M+H]+	164.10698	C10H13NO	N-acetyl-2-arylethylamines	MODKMHXGCGKTLE-UHFFFAOYSA-N	OC(=NCCC=1C=CC=CC1)C
1576	5.143	443.20349	Perlatolic acid	[M-H]-	443.20001	C25H32O7	Depsides and depsidones	UTAQXQVSEKIWBB-UHFFFAOYSA-N	O=C(O)C1=C(O)C=C(OC(=O)C2=C(O)C=C(OC)C=C2CCCCC)C=C1CCCCC
1577	5.15	381.20328	1-Myristoyl-2-hydroxy-sn-glycero-3-phosphate	[M-H]-	381.20477	C17H35O7P	1-acylglycerol-3-phosphates	FAZBDRGXCKPVJU-MRXNPFEDSA-N	CCCCCCCCCCCCCC(=O)OC[C=H](O)COP(O)(O)=O
1578	5.151	509.30313	18b-glycyrrhetinic acid	[M+2H]2+	509.30276	C30H46O4	Triterpenoids	MPDGHEJMBKOTSU-AWOGJAJBSA-N	O=C(O)C1(C)CCC2(C)CCC3(C(=CC(=O)C4C5(C)CCC(O)C(C)(C)C5CCC43C)C2C1)C
1579	5.152	369.22314	5-(3-methoxyphenyl)-N-((octahydro-1H-quinolizin-1-yl)methyl)-1H-pyrazole-3-carboxamide	[M+H]+	369.23001	C21H28N4O2	Phenylpyrazoles	ZZFSKJIAKIBPSR-UHFFFAOYSA-N	O=C(NCC1CCCN2CCCCC21)C3=NNC(=C3)C=4C=CC=C(OC)C4
1580	5.152	382.14096	Capecitabine (Xeloda)	[M+H]+	382.138	C15H22FN3O6	5'-deoxyribonucleosides	GAGWJHPBXLXJQN-GMTLJSCSSA-N	O=C(OCCCCC)NC1=NC(=O)N(C=C1F)C2OC(C)C(O)C2O
1581	5.152	353.16904	meso-dihydroguaiaretic acid	[M+H]+	353.17001	C20H26O4	Dibenzylbutane lignans	ADFOLUXMYYCTRR-ZIAGYGMSSA-N	OC1=CC=C(C=C1OC)CC(C)C(C)CC2=CC=C(O)C(OC)=C2
1582	5.153	193.1331	Isoproturon-monodemethyl	[M+H]+	193.13354	C11H16N2O	N-phenylureas	DOULWWSSZVEPIN-UHFFFAOYSA-N	CN=C(O)NC1=CC=C(C=C1)C(C)C
1583	5.157	282.13272	4,7,8-trimethoxy-3,5-dimethylchromen-2-one	[M+H]+	282.13361	C14H16O5	Coumarins and derivatives	PKKTXAMCHLIVDS-UHFFFAOYSA-N	O=C1OC2=C(OC)C(OC)=CC(=C2C(OC)=C1C)C
1584	5.159	383.19971	Voacristine	[M-H]-	383.19763	C22H28N2O4	Alkaloids	OYMQKBZMKFJPMH-UHFFFAOYNA-N	COC(=O)C12CC3CC(C(C)O)C1N(C3)CCC1=C2NC2=CC=C(OC)C=C12
1585	5.174	174.18509	MENTHOL(-)	[M+H]+	174.18523	C10H20O	Menthane monoterpenoids	NOOLISFMXDJSKH-UHFFFAOYSA-N	OC1CC(C)CCC1C(C)C
1586	5.175	401.1597	aschantin	[M+H]+	401.15948	C22H24O7	Furanoid lignans	ONDWGDNAFRAXCN-VUEDXXQZSA-N	O(C=1C=C(C=C(OC)C1OC)C2OCC3C(OCC23)C4=CC=C5OCOC5=C4)C
1587	5.176	327.08426	Isosaxalin	[M+H]+	327.08401	C16H16O6	Psoralens	FOINLJRVEBYARJ-UHFFFAOYSA-N	O=C1OC=2C(C=C1)=CC=3C=COC3C2OCC(O)C(O)(C)C
1588	5.179	209.07835	Sinapyl alcohol	[M-H]-	209.08194	C11H14O4	Methoxyphenols	LZFOPEXOUVTGJS-UHFFFAOYSA-N	OC=1C(OC)=CC(C=CCO)=CC1OC
1589	5.184	309.11179	Warfarin	[M+H]+	309.11212	C19H16O4	4-hydroxycoumarins	PJVWKTKQMONHTI-UHFFFAOYNA-N	CC(CC(C1=CC=CC=C1)C2=C(C3=CC=CC=C3OC2=O)O)=O

1590	5.186	427.15753	9,11,12a-trihydroxy-2,3-dimethoxy-8-(3-methylbut-2-enyl)-6,6a-dihydrochromeno[3,4-b]chromen-12-one	[M-H]-	427.16016	C23H24O8	Rotenones	LAUJMTGOOIBBOB-UHFFFAOYSA-N	O=C1C2=C(O)C=C(O)C(=C2OC3COC4=CC(OC)=C(OC)C=C4C13O)CC=C(C)C
1591	5.186	187.09703	Azelaic acid (Not validated)	[M-H]-	187.0974	C9H16O4	Organic acids	BDJRBEYXGGNYIS-UHFFFAOYSA-N	O=C(O)CCCCCCCC(=O)O
1592	5.189	276.99207	FT-PFCA	[M-H]-	276.99167	C6H3F9O2	PFSA	PMNVMTIBUIRJHZ-UHFFFAOYSA-N	O=C(O)CC(F)(F)C(F)(F)C(F)(F)C(F)(F)F
1593	5.191	317.065	methyl 2-(2,6-dihydroxy-4-methylbenzoyl)-3,5-dihydroxybenzoate	[M-H]-	317.06668	C16H14O7	Benzophenones	GIJDACMRFIMBET-UHFFFAOYSA-N	O=C(OC)C=1C=C(O)C=C(O)C1C(=O)C2=C(O)C=C(C=C2O)C
1594	5.191	466.23239	Norgenamide D_130072	[M+H]+	466.233	C26H31N3O5	Pyrroloindoles	DMIIGSHOFYCWKW-RRGUOAIQSA-N	O=C1N2CCCC2(O)C(=O)N3C1CC4(O)C5=CC=C6OC(C=CC6=C5NC34C(C=C)(C)C)(C)C
1595	5.195	651.35663	Lopinavir (ABT-378)	[M+H]+	651.35199	C37H48N4O5	Valine and derivatives	KJHKTHWMRKYKJE-SUGCFTRWSA-N	O=C1NCCCN1C(C(=O)NC(CC=2C=CC=CC2)CC(O)C(NC(=O)COC=3C(=CC=CC3C)C)CC=4C=CC=CC4)C(C)C
1596	5.197	493.13254	Anthocyanidin base + 4O, 2MeO, O-Hex	[M+H]+	493.13281	C23H25O12	Anthocyanidin O-glycosides	PXUQTDZNOHRWLI-UHFFFAOYNA-O	OC=2C=C(O)C=3C=C(OC1OC(CO)C(O)C(O)C1(O))C(=[O+]C=3(C=2))C=4C=C(OC)C(O)=C(OC)C=4
1597	5.197	293.1012	Benzyl glucopyranoside	[M+H]+	293.10001	C13H18O6	O-glycosyl compounds	GKHCBYYBLTXYEV-UJPOAAIJSA-N	OCC1OC(OCC=2C=CC=CC2)C(O)C(O)C1O
1598	5.197	310.20081	Methadone	[M+H]+	310.20099	C21H27NO	Diphenylmethanes	USSIQXCVUWKGNF-UHFFFAOYSA-N	O=C(CC)C(C=1C=CC=CC1)(C=2C=CC=CC2)CC(N(C)C)C
1599	5.199	288.25189	Emodin	[M+H]+	288.25159	C15H10O5	Hydroxyanthraquinones	RHMXXJGYXNZAPX-UHFFFAOYSA-N	O=C1C=2C=C(O)C=C(O)C2C(=O)C3=C(O)C=C(C=C13)C
1600	5.201	189.11185	Azelaic acid	[M+H]+	189.11211	C9H16O4	Medium-chain fatty acids	BDJRBEYXGGNYIS-UHFFFAOYSA-N	O=C(O)CCCCCCCC(=O)O
1601	5.205	295.06955	alpha-Naphthoflavone	[M+H]+	295.07001	C19H12O2	Flavones	VFMMPHCGEFXGIP-UHFFFAOYSA-N	O=C1C=C(OC2=C1C=CC=3C=CC=CC32)C=4C=CC=CC4
1602	5.242	367.16376	4-(4-(2,3-dihydrobenzo[b][1,4]dioxin-6-yl)-3-methyl-1H-pyrazol-5-yl)-6-propylbenzene-1,3-diol	[M+H]+	367.164	C21H22N2O4	Phenylpyrazoles	FOUMOIACEBZYPT-UHFFFAOYSA-N	OC=1C=C(O)C(=CC1C=2NN=C(C2C3=CC=C4OCCOC4=C3)C)CCC
1603	5.251	365.14929	1-hydroxy-4-[(2S,3R,4S,5S,6R)-3,4,5-trihydroxy-6-(hydroxymethyl)oxan-2-yl]oxynaphthalene-2-carboxylic acid	[M-H]-	365.15024	C17H18O9	Phenolic glycosides	SGTNXGSCHXKQJX-OQAPJDJNSA-N	O=C(O)C=1C=C(OC2OC(CO)C(O)C(O)C2O)C=3C=CC=CC3C1O
1604	5.251	235.16858	Curcumenol	[M+H]+	235.16925	C15H22O2	Guaianes	ISFMXVMWEWLJGJ-UHFFFAOYSA-N	OC12OC3(CC1=C(C)C)C(C(=C2)C)CCC3C
1605	5.251	316.03421	Efavirenz	[M+H]+	316.03467	C14H9ClF3NO2	Benzoxazines	XPOQHMRABVBWPR-UHFFFAOYNA-N	C1CC1C#CC2(C=3C=C(C=CC3N=C(O)O2)Cl)C(F)(F)F
1606	5.253	351.11884	Isoflavone base + 2O + 1MeO + 1Prenyl	[M-H]-	351.1218	C21H20O5	Prenylated isoflavones	JQNSUDIGIIGIOL-UHFFFAOYSA-N	O=C1C(=COC=2C=C(O)C(=C(O)C1=2)CC=C(C)C)C3=CC=C(OC)C=C3
1607	5.255	377.12982	Griseofulvic Acid	[M+H]+	377.12964	C16H15ClO6	Benzofurans	KUTVEHNZHKAAAO-UHFFFAOYSA-N	O=C1C=C(O)C2(OC=3C(Cl)=C(OC)C=C(OC)C3C2=O)C(C)C1
1608	5.265	515.18005	Cilnidipine	[M+H]+	515.17902	C27H28N2O7	Dihydropyridinecarboxylic acids and derivatives	KJEBULYHNRNJTE-DHZHZOJOSA-N	O=C(OCC=CC=1C=CC=CC1)C2=C(NC(=C(C(=O)OCCOC)C2C=3C=CC=C(C3)[N+](=O)[O-])C)C
1609	5.268	435.16284	4-[(2R,3R,4S,5S,6R)-3,4,5-trihydroxy-6-(hydroxymethyl)oxan-2-yl]oxypentan-2-yl (E)-3-(4-hydroxyphenyl)prop-2-enoate	[M+H]+	435.16254	C20H28O9	Fatty acyl glycosides of mono- and disaccharides	RMFDCHYDPYVNFA-AKVWERCLSA-N	O=C(OC(C)CC(OC1OC(CO)C(O)C(O)C1O)C)C=CC2=CC=C(O)C=C2
1610	5.282	355.14804	Isoxanthohumol	[M+H]+	355.14999	C21H22O5	8-prenylated flavanones	YKGCBLWILMDSAV-SFHVURJKSA-N	O=C1C=2C(OC)=CC(O)=C(C2OC(C3=CC=C(O)C=C3)C1)CC=C(C)C
1611	5.287	643.38629	Cimigenol-3-O-alpha-L-arabinoside	[M+2H]2+	643.38202	C35H56O9	Cucurbitacin glycosides	BTPYUWOBZFGKAI-BKJHYQRZSA-N	OC1COC(OC2CCC34CC54CCC6(C)C7C(C)CC8OC7(OC8C(O)(C)C)C(O)C6(C)C5CCC3C2(C)C)C(O)C1O
1612	5.302	206.08046	Methoxyindoleacetic acid	[M+H]+	206.08118	C11H11NO3	Indole-3-acetic acid derivatives	COCNDHOPIHDTHK-UHFFFAOYSA-N	COC1=CC2=C(NC=C2CC(O)=O)C=C1
1613	5.303	570.35529	Secopenitrem D_120253	[M+2H]2+	570.35699	C37H47NO4	Naphthopyrans	YZQRMJFPWMHAJV-DTOWOETDSA-N	OC1C=C2C(OC1C(=C)C)CCC3(C)C2(O)CCC4CC=5C=6C(=CC=C7C6C8C(C(=C)C7)CC8C(O)(C)C)NC5C43C
1614	5.328	225.14819	Methyl jasmonate	[M+H]+	225.14851	C13H20O3	Jasmonic acids	GEWDNTWNSAZUDX-UHFFFAOYNA-N	O=C(OC)CC1CCC(=O)C1CC=CCC
1615	5.337	562.37933	Rhodoxanthin	[M+H]+	562.38055	C40H50O2	Xanthophylls	VWXMLZQUDPCJPL-ZDHAIZATSA-N	C\C(\C=C\C=C\C(\C)=C\C=C\C(\C)=C\C=C1\C(C)=CC(=O)CC1(C)C)=C/C=C/C(/C)=C/C=C1/C(C)=CC(=O)CC1(C)C
1616	5.339	351.1651	candidone	[M-H]-	351.16019	C22H24O4	8-prenylated flavanones	JYESOAFLKFHYHP-SFHVURJKSA-N	O=C1C2=C(OC)C=C(OC)C(=C2OC(C=3C=CC=CC3)C1)CC=C(C)C
1617	5.339	397.95212	Perfluorohexanesulfonamide	[M-H]-	397.95258	C6H2F13NO2S	PFSA	WDZLGCSJJWEQJO-UHFFFAOYSA-N	O=S(=O)(N)C(F)(F)C(F)(F)C(F)(F)C(F)(F)C(F)(F)C(F)(F)F
1618	5.349	232.09625	Pesticide3_Propoxur_C11H15NO3_Baygon	[M+H]+	232.09399	C11H15NO3	Phenol ethers	ISRUGXGCCGIOQO-UHFFFAOYSA-N	OC(=NC)OC=1C=CC=CC1OC(C)C
1619	5.35	167.07001	3,4-Dimethoxybenzaldehyde	[M+H]+	167.07027	C9H10O3	Dimethoxybenzenes	WJUFSDZVCOTFON-UHFFFAOYSA-N	O=CC1=CC=C(OC)C(OC)=C1
1620	5.355	235.06218	austadiol	[M-H]-	235.06119	C12H12O5	Azaphilones	QVMUHZHZYCDMAI-RYUDHWBXSA-N	O=CC=1C(=O)C(O)(C)C(O)C2=COC(=CC12)C
1621	5.356	148.07509	Cinnamamide	[M+H]+	148.07568	C9H9NO	Cinnamic acid amides	APEJMQOBVMLION-UHFFFAOYSA-N	C1=CC=C(C=C1)C=CC(=N)O
1622	5.36	180.1378	MEX_180.1383_11.9	[M+H]+	180.13829	C11H17NO	Phenol ethers	VLPIATFUUWWMKC-UHFFFAOYNA-N	CC=1C=CC=C(C)C1OCC(C)N
1623	5.372	309.14584	1,7,8-trihydroxy-3-methyl-2,3,4,7-tetrahydro-1H-benzo[a]anthracen-12-one	[M-H]-	309.14124	C19H18O4	Phenanthrols	FDVOVSCQDWKKHH-UHFFFAOYSA-N	O=C1C=2C=CC=C(O)C2C(O)C3=CC=C4C(=C13)C(O)CC(C)C4
1624	5.385	336.16632	Olopatadine	[M-H]-	336.16052	C21H23NO3	Dibenzoxepines	JBIMVDZLSHOPLA-UHFFFAOYSA-N	O=C(O)CC1=CC=C2OCC=3C=CC=CC3C(=CCCN(C)C)C2=C1
1625	5.404	229.06003	Pesticide2_Aldicarb sulfoxide_C7H14N2O3S_Propanal, 2-methyl-2-(methylsulfinyl)-, O-[(methylamino)carbonyl]oxime, (1E)-	[M+H]+	229.062	C7H14N2O3S	Oxime carbamates	BXPMAGSOWXBZHS-WEVVVXLNSA-N	O=S(C)C(C=NOC(O)=NC)(C)C
1626	5.405	162.09091	Tryptophol	[M+H]+	162.091	C10H11NO	3-alkylindoles	MBBOMCVGYCRMEA-UHFFFAOYSA-N	OCCC1=CNC=2C=CC=CC21
1627	5.407	144.08031	1-Naphthylamine	[M+H]+	144.08078	C10H9N	Naphthalenes	RUFPHBVGCFYCNW-UHFFFAOYSA-N	C1=CC=C2C(=C1)C=CC=C2N
1628	5.418	326.06641	Fluazifop	[M-H]-	326.06458	C15H12F3NO4	Aryloxyphenoxypropionic acids	YUVKUEAFAVKILW-UHFFFAOYNA-N	CC(OC1=CC=C(OC2=NC=C(C=C2)C(F)(F)F)C=C1)C(O)=O
1629	5.434	189.98151	2,6-Dichlorobenzamide	[M+H]+	189.9821	C7H5Cl2NO	2-halobenzoic acids and derivatives	JHSPCUHPSIUQRB-UHFFFAOYSA-N	C1=CC(=C(C(=C1)Cl)C(=N)O)Cl
1630	5.435	261.11215	violaceol i	[M-H]-	261.11188	C14H14O5	Diphenylethers	YRZXKRQRZJMBFT-UHFFFAOYSA-N	OC=1C=C(C=C(OC2=CC(=CC(O)=C2O)C)C1O)C
1631	5.437	303.23126	Methyltestosterone	[M+H]+	303.23184	C20H30O2	Androgens and derivatives	GCKMFJBGXUYNAG-UHFFFAOYNA-N	O=C1C=C2CCC3C(CCC4(C)C3CCC4(O)C)C2(C)CC1
1632	5.461	259.15439	3-hydroxy-2-octylpentanedioic acid	[M-H]-	259.15509	C13H24O5	Medium-chain hydroxy acids and derivatives	ROYIOVVKJBERGN-UHFFFAOYSA-N	O=C(O)CC(O)C(C(=O)O)CCCCCCCC

1633	5.469	497.19168	Strictosamide	[M-H]-	497.19171	C26H30N2O8	Carbolines	LBRPLJCNRZUXLS-UHFFFAOYNA-N	O=C5C2=COC(OC1OC(CO)C(O)C(O)C1(O))C(C=C)C2CC6C=4NC=3C=CC=CC=3C=4CCN56
1634	5.47	265.11737	conocarpan	[M-H]-	265.12051	C18H18O2	2-arylbenzofuran flavonoids	GXJSAHXNLJFDPO-CGBXWHSCSA-N	OC1=CC=C(C=C1)C2OC3=CC=C(C=CC)C=C3C2C
1635	5.471	403.16257	(Z)-3-(4-hydroxyphenyl)-N-[4-[[(E)-3-(4-hydroxyphenyl)prop-2-enoyl]amino]butyl]prop-2-enamide	[M+H]+	403.16281	C22H24N2O4	Coumaric acids and derivatives	PYVBFDCHJDMSMM-MFUUIURDSA-N	OC(=NCCCCN=C(O)C=CC1=CC=C(O)C=C1)C=CC2=CC=C(O)C=C2
1636	5.474	417.13855	7-hydroxy-2-[4-[(2S,3R,4S,5S,6R)-3,4,5-trihydroxy-6-(hydroxymethyl)oxan-2-yl]oxyphenyl]-2,3-dihydrochromen-4-one	[M-H]-	417.13687	C21H22O9	Flavonoid O-glycosides	DEMKZLAVQYISIA-UZQFATADSA-N	O=C1C2=CC=C(O)C=C2OC(C3=CC=C(OC4OC(CO)C(O)C(O)C4O)C=C3)C1
1637	5.475	449.14609	Isosakuranin	[M+H]+	449.14499	C22H24O10	Flavonoid-7-O-glycosides	KEEWIHDTSNESJZ-ZJHVPRRPSA-N	O=C1C2=C(O)C=C(OC3OC(CO)C(O)C(O)C3O)C=C2OC(C4=CC=C(OC)C=C4)C1
1638	5.495	430.27469	Mifepristone	[M+H]+	430.27405	C29H35NO2	Oxosteroids	VKHAHZOOUSRJNA-UHFFFAOYNA-N	O=C1C=C2C(=C3C(C4=CC=C(C=C4)N(C)C)CC5(C)C(CCC5(O)C#CC)C3CC2)CC1
1639	5.498	183.07932	Triethylphosphate	[M+H]+	183.07808	C6H15O4P	Trialkyl phosphates	DQWPFSLDHJDLRL-UHFFFAOYSA-N	CCOP(=O)(OCC)OCC
1640	5.504	619.36914	Desferrioxamine G1	[M+2H]2+	619.37	C27H50N6O10	N-acyl amines	MIVGUYBAQIHKPJ-UHFFFAOYSA-N	O=C(O)CCC(=O)N(O)CCCCCNC(=O)CCC(=O)N(O)CCCCCNC(=O)CCC(=O)N(O)CCCCCN
1641	5.511	335.1221	5-hydroxy-2,2-dimethyl-10-(2-methylbut-3-en-2-yl)pyrano[3,2-g]chromen-8-one	[M+H]+	335.12537	C19H20O4	Linear pyranocoumarins	WYMMNGQSPNWCIK-UHFFFAOYSA-N	O=C1OC2=C(C=C1)C(O)=C3C=CC(OC3=C2C(C=C)(C)C)(C)C
1642	5.512	617.36523	Cimicidanol-3-O-alpha-L-arabinoside	[M+H]+	617.37	C35H52O9	Cycloartanols and derivatives	PYBFXJMIKJNNAJ-UHFFFAOYSA-N	O=C(CC(C)C1C(=O)CC2(C3=CCC4C(C)(C)C(OC5OCC(O)C(O)C5O)CCC64CC36C(O)CC12C)C)C7OC7(C)C
1643	5.515	325.14825	4-amino-2-hydroxy-5-[[1-hydroxy-1-(5-oxo-6-bicyclo[4.1.0]hept-3-enyl)propan-2-yl]amino]-5-oxopentanoic acid	[M-H]-	325.14774	C15H22N2O6	Glutamic acid and derivatives	SYGQXRLXOOXDRP-UHFFFAOYSA-N	O=C(O)C(O)CC(N)C(O)=NC(C)C(O)C12C(=O)C=CCC2C1
1644	5.52	284.12744	Coumaroyl tyramine	[M+H]+	284.12851	C17H17NO3	Coumaric acid and derivatives	RXGUTQNKCXHALN-UHFFFAOYSA-N	O=C(C=CC1=CC=C(O)C=C1)NCCC2=CC=C(O)C=C2
1645	5.523	389.25284	Ilicicolin A	[M-H]-	389.2518	C23H31ClO3	Sesquiterpenoids	MHWOMRMBQGSTFS-JTCWOHKRSA-N	O=CC1=C(O)C(=C(O)C(Cl)=C1C)CC=C(C)CCC=C(C)CCC=C(C)C
1646	5.537	213.07297	N,N-Dimethyl-N'-p-tolylsulphamide	[M-H]-	213.07033	C9H14N2O2S	Sulfanilides	UDCDOJQOXWCCSD-UHFFFAOYSA-N	CN(C)S(=O)(=O)NC1=CC=C(C)C=C1
1647	5.551	257.12698	6-ethyl-2,3,5-trimethyl-7H-furo[3,2-g]chromen-7-one	[M+H]+	257.12	C16H16O3	Psoralens	USSZROTZVDHBKM-UHFFFAOYSA-N	O=C1OC=2C=C3OC(=C(C3=CC2C(=C1CC)C)C)C
1648	5.568	650.4295	(1S,2R,4aS,6aR,6bR,10S,12aR,14bS)-1,2,6b,9,9,12a-hexamethyl-10-[(2R,3R,4S,5S,6R)-3,4,5-trihydroxy-6-methyloxan-2-yl]oxy-	[M+H]+	650.42621	C36H56O9	Triterpene saponins	PUOQHFWXBKTHST-MPLMROMBSA-N	O=C(O)C12C(=CCC3C4(C)CCC(OC5OC(C)C(O)C(O)C5O)C(C)(C)C4CCC31C)C6C(C)C(C)CCC6(C(=O)O)CC2
1649	5.573	321.24225	5-[(8aS)-2,5,5,8a-tetramethyl-3-oxo-4a,6,7,8-tetrahydro-4H-naphthalen-1-yl]-3-methylpentanoic acid	[M+H]+	321.2424	C20H32O3	Diterpenoids	BARZJLALPXZLMN-RJGDCVCESA-N	O=C(O)CC(C)CCC1=C(C(=O)CC2C1(C)CCCC2(C)C)C
1650	5.582	322.12711	HERALENOL	[M+H]+	322.12851	C16H16O6	Psoralens	FOINLJRVEBYARJ-UHFFFAOYSA-N	O=C1OC=2C(C=C1)=CC=3C=COC3C2OCC(O)C(O)(C)C
1651	5.588	179.03394	4-Hydroxyphenylpyruvic acid	[M-H]-	179.03499	C9H8O4	Phenylpyruvic acid derivatives	KKADPXVIOXHVKN-UHFFFAOYSA-N	O=C(O)C(=O)CC1=CC=C(O)C=C1
1652	5.588	259.09692	6-methoxy-7-(3-methylbut-2-enoxy)chromen-2-one	[M-H]-	259.09756	C15H16O4	Coumarins and derivatives	ZLPLFUBVEZVYDX-UHFFFAOYSA-N	O=C1OC=2C=C(OCC=C(C)C)C(OC)=CC2C=C1
1653	5.596	433.17191	osmanthuside H	[M+H]+	433.17044	C19H28O11	O-glycosyl compounds	IVRQZYXJBVMHCW-OTCFHACESA-N	OC1=CC=C(C=C1)CCOC2OC(COC3OCC(O)(CO)C3O)C(O)C(O)C2O
1654	5.598	263.12848	Abscisic acid	[M-H]-	263.12888	C15H20O4	Abscisic acids and derivatives	JLIDBLDQVAYHNE-UHFFFAOYNA-N	O=C(O)C=C(C=CC1(O)C(=CC(=O)CC1(C)C)C)C
1655	5.601	137.02368	4-Hydroxybenzoic acid	[M-H]-	137.02441	C7H6O3	Hydroxybenzoic acid derivatives	FJKROLUGYXJWQN-UHFFFAOYSA-N	OC(=O)C1=CC=C(O)C=C1
1656	5.602	843.35504	(8R,10R,11S,11aS,13S)-9-ethyl-8,13-dihydroxy-7-(2-(((10S,13R)-14-hydroxy-10,13-dimethyl-17-(5-oxo-2,5-dihydrofuran-3-yl)hexadecahydro-1H-	[M+H]+	843.34998	C45H61BrN2O7	Cardenolides and derivatives	JPBBOIVVPLONQC-YWVZAZLPSA-M	[Br-].O=C1OCC(=C1)C2CCC3(O)C4CCC5CC(OC(=O)C[N+]67C(O)C(CC)
1657	5.604	507.15305	Scutellarioside II	[M-H]-	507.15079	C24H28O12	Coumaric acid esters	MVTLXFDHKDVAIC-LQQBYVAQSA-N	O=C(OCC12OC2C(O)C3C=COC(OC4OC(CO)C(O)C(O)C4O)C31)C=CC5=CC=C(O)C=C5
1658	5.604	352.16827	Usaramine	[M+H]+	352.17001	C18H25NO6	Macrolides and analogues	BCJMNZRQJAVDLD-VMOBWIEOSA-N	O=C1OC2CCN3CC=C(COC(=O)C(O)(CO)C(C)CC1=CC)C32
1659	5.608	265.1431	(9E)-11a-hydroxy-3,6,10-trimethyl-6,7,8,11-tetrahydro-4H-cyclodeca[b]furan-2,5-dione	[M+H]+	265.14343	C15H20O4	Germacranolides and derivatives	SCOXWKWLFRIELY-WEVVVXLNSA-N	O=C1OC2(O)C(=C1C)CC(=O)C(C)CCC=C(C)C2
1660	5.608	273.13394	afzelechin	[M-H]-	273.13797	C15H14O5	Flavan-3-ols	RSYUFYQTACJFML-DZGCQCFKSA-N	OC1=CC=C(C=C1)C2OC=3C=C(O)C=C(O)C3CC2O
1661	5.612	255.11237	Nepafenac	[M+H]+	255.11301	C15H14N2O2	Benzophenones	QEFAQIPZVLVERP-UHFFFAOYSA-N	O=C(N)CC=1C=CC=C(C(=O)C=2C=CC=CC2)C1N
1662	5.614	448.27548	Jervine	[M+H]+	448.28	C27H39NO3	Jerveratrum-type alkaloids	CLEXYFLHGFJONT-UHFFFAOYSA-N	O=C1C2=C(C)C3(OC4CC(C)CNC4C3C)CCC2C5CC=C6CC(O)CCC6(C)C15
1663	5.619	274.08472	Pomalidomide	[M+H]+	274.082	C13H11N3O4	Phthalimides	UVSMNLNDYGZFPF-UHFFFAOYSA-N	O=C1C=2C=CC=C(N)C2C(=O)N1C3C(=O)NC(=O)CC3
1664	5.63	479.15591	Isolindleyin	[M+H]+	479.155	C23H26O11	Phenolic glycosides	KHUVRRVIZOSFTI-UHFFFAOYSA-N	O=C(OC1C(OC2=CC=C(C=C2)CCC(=O)C)OC(CO)C(O)C1O)C3=CC(O)=C(O)C(O)=C3
1665	5.664	457.2757	Mundulone	[M+H]+	457.2749	C26H26O6	6-prenylated isoflavanones	KJTDZAHLWHEULN-UHFFFAOYSA-N	O=C1C(=COC=2C=C3OC(C)(C)C(O)CC3=CC21)C=4C=CC=5OC(C=CC5C4OC)(C)C
1666	5.668	218.1898	Prolintane	[M+H]+	218.19032	C15H23N	Amphetamines and derivatives	OJCPSBCUMRIPFL-UHFFFAOYNA-N	CCCC(CC1=CC=CC=C1)N1CCCC1
1667	5.67	331.12057	sulochrin	[M-H]-	331.11725	C17H16O7	Benzophenones	YJRLSCDUYLRBIZ-UHFFFAOYSA-N	O=C(OC)C=1C=C(O)C=C(OC)C1C(=O)C2=C(O)C=C(C=C2O)C
1668	5.672	255.11191	Obtusaquinone	[M+H]+	255.11137	C16H14O3	P-quinomethanes	LUZUAYAKZLCOCQ-BHHNFLQBSA-N	O=C1C=C(OC)C(=CC=CC=2C=CC=CC2)C=C1O
1669	5.674	465.18945	2-[[4-(2,4-dihydroxy-3,5,6-trimethylbenzoyl)oxy-2-hydroxy-6-methylbenzoyl]amino]-3-hydroxybutanoic acid	[M+H]+	465.18674	C22H25NO9	Depsides and depsidones	NJVQCZVYEIAGLE-UHFFFAOYSA-N	O=C(O)C(N=C(O)C1=C(O)C=C(OC(=O)C=2C(O)=C(C(O)=C(C2C)C)C)C=C1C)C(O)C
1670	5.676	319.20114	Chaulmoogric Acid	[M+H]+	319.20337	C18H32O2	Long-chain fatty acids	XMVQWNRDPAAMJB-UHFFFAOYSA-N	O=C(O)CCCCCCCCCCCCC1C=CCC1
1671	5.677	238.14311	N,N-Diethyl-3,4-dimethoxybenzamide	[M+H]+	238.14377	C13H19NO3	Dimethoxybenzenes	ZQTJMIZZICSOBJ-UHFFFAOYSA-N	CCN(CC)C(=O)C1=CC(OC)=C(OC)C=C1
1672	5.678	463.28424	Methyl-mappain	[M+H]+	463.28201	C30H38O4	Stilbenes	MRXGJOGASBBDTM-FVFOHYRDSA-N	OC=1C=C(C=CC=2C=C(OC)C(O)=C(C2)CC=C(C)CCC=C(C)C)C=C(O)C1CC=C(C)C
1673	5.68	208.13298	Pesticide3_Fenobucarb_C12H17NO2_Bassa	[M+H]+	208.133	C12H17NO2	Phenyl methylcarbamates	DIRFUJHNVNOBMY-UHFFFAOYSA-N	OC(=NC)OC=1C=CC=CC1C(C)CC
1674	5.681	479.15488	calceolarioside A	[M+H]+	479.15479	C23H26O11	Coumaric acids and derivatives	UHIGZYLCYRQESL-VJWFJHQPSA-N	O=C(OC1C(O)C(O)C(OCCC2=CC=C(O)C(O)=C2)OC1CO)C=CC3=CC=C(O)C(O)=C3
1675	5.684	281.19653	Aspidofractinine	[M+H]+	281.20099	C19H24N2	Aspidofractine alkaloids	BIBZWCCWSCCFBB-MKCYZYCBSA-N	C=1C=CC2=C(C1)NC34CCC5(CCCN6CCC23C65)CC4

1676	5.684	267.11902	Ponatinib (AP24534)	[M+H]+	267.11801	C29H27F3N6O	Benzanilides	PHXJVRSECIGDHY-UHFFFAOYSA-N	O=C(NC1=CC=C(C(=C1)C(F)(F)F)CN2CCN(C)CC2)C3=CC=C(C(C#C C4=CN=C5C=CC=NN45)=C3)C
1677	5.686	229.10693	2-(6-hydroxyhexyl)-3-methylidenebutanedioic acid	[M-H]-	229.10814	C11H18O5	Medium-chain hydroxy acids and derivatives	DHVXMTMJTVCPBB-UHFFFAOYSA-N	O=C(O)C(=C)C(C(=O)O)CCCCCCO
1678	5.687	504.23227	N-(1-(2-amino-2-oxoethyl)piperidin-4-yl)-3-(4-hydroxy-6-methyl-2-oxo-2H-pyran-3-yl)-3-(2,3,4-trimethoxyphenyl)propanamide	[M+H]+	504.23001	C25H33N3O8	Alpha amino acid amides	YEIWCFPXBQSECZ-UHFFFAOYSA-N	O=C1OC(=CC(O)=C1C(C2=CC=C(OC)C(OC)=C2OC)CC(=O)NC3CCN(C C(=O)N)CC3)C
1679	5.693	349.13013	[(10Z)-6,10-bis(hydroxymethyl)-3-methylidene-2-oxo-3a,4,5,6,7,8,9,11a-octahydrocyclodeca[b]furan-4-yl] 2-methylprop-2-enoate	[M-H]-	349.13354	C19H26O6	Germacranolides and derivatives	RJHSXEZOOUJAQE-ZSOIEALJSA-N	O=C(OC1CC(CO)CCCC(=CC2OC(=O)C(=C)C21)CO)C(=C)C
1680	5.702	279.1813	cyclopeptine	[M-H]-	279.17978	C17H16N2O2	1,4-benzodiazepines	KSQNKZMAMGACTL-UHFFFAOYSA-N	O=C1C=2C=CC=CC2N=C(O)C(N1C)CC=3C=CC=CC3
1681	5.72	488.97534	Pesticide1_Temephos_C16H20O6P2S3_O,O,O',O'-Tetramethyl O,O'-(sulfanediyldi-4,1-phenylene) bis(phosphorothioate)	[M+H]+	488.979	C16H20O6P2S3	Phenyl thiophosphates	WWJZWCUNLNYYAU-UHFFFAOYSA-N	S=P(OC1=CC=C(SC2=CC=C(OP(=S)(OC)OC)C=C2)C=C1)(OC)OC
1682	5.723	483.30066	TRIDESACETOXYKHIVORIN	[M+H]+	483.30237	C26H36O7	Limonoids	MGQUMSFBIYXTTE-FIYJXLSPSA-N	O=C1OC(C2=COC=C2)C3(C)CCC4C5(C)C(O)CC(O)C(C)(C)C5CC(O) C4(C)C63OC16
1683	5.725	477.15842	Benzyl alcohol + Hex-Hex	[M-H]-	477.15961	C19H28O11	Alkyl benzyl hexosides	WKXRKKUZTXIFJY-UHFFFAOYNA-N	OCC3OC(OCC1=CC=CC=C1)C(OC2OC(CO)C(O)C(O)C2(O))C(O)C3(O)
1684	5.727	370.21509	(1S,8R,16S,17R)-11,12-dimethoxy-5,15-diazahexacyclo[13.4.2.0?,??.0?,??.0?,??.0?,??]henicosa-2,9,11,13-tetraen-	[M+H]+	370.21249	C21H24N2O3	Aminoquinolines and derivatives	BOPPUHZKIKFBHH-GEOUXAIOSA-N	O=C1N2C3=CC(OC)=C(OC)C=C3C4CCN5CC=CC6(C1)CCC4C256
1685	5.727	537.16302	[(1aS,1bS,2S,5aR,6S,6aS)-1a-(hydroxymethyl)-2-[(2S,3R,4S,5S,6R)-3,4,5-trihydroxy-6-(hydroxymethyl)oxan-2-yl]oxy-2,5a,6,6a-tetrahydro-1bH-	[M-H]-	537.16138	C25H30O13	Coumaric acids and derivatives	LRHHPZILMPIMIY-GGKKSNITSA-N	O=C(OC1C2OC2(CO)C3C(OC=CC13)OC4OC(CO)C(O)C(O)C4O)C=CC5 =CC=C(OC)C(O)=C5
1686	5.727	161.0594	4-Methylcoumarin	[M+H]+	161.05971	C10H8O2	Coumarins and derivatives	PSGQCCSGKGJLRL-UHFFFAOYSA-N	CC1=CC(=O)OC2=CC=CC=C12
1687	5.727	295.04913	Butin	[M+H]+	295.04999	C15H12O5	Flavanones	MJBPUQUGJNAPAZ-AWEZNQCLSA-N	O=C1C2=CC=C(O)C=C2OC(C3=CC=C(O)C(O)=C3)C1
1688	5.727	439.18497	Diferuloyl putrescine	[M-H]-	439.18689	C24H28N2O6	Ferulic acid and derivatives	CHEMZHJQHCVLFI-UHFFFAOYSA-N	OC(=NCCCCN=C(O)C=CC=1C=CC(O)=C(OC)C=1)C=CC=2C=CC(O)=C(OC)C=2
1689	5.727	336.07516	Gossypetin	[M+H]+	336.07138	C15H10O8	Flavonols	YRRAGUMVDQQZIY-UHFFFAOYSA-N	O=C1C(O)=C(OC=2C(O)=C(O)C=C(O)C12)C=3C=CC(O)=C(O)C3
1690	5.727	341.05438	lecanoric acid	[M+H]+	341.06	C16H14O7	Depsides and depsidones	HEMSJKZDHNSSEW-UHFFFAOYSA-N	O=C(O)C1=C(O)C=C(OC(=O)C=2C(O)=CC(O)=CC2C)C=C1C
1691	5.727	339.08389	methyl 2-(9-hydroxy-1-methyl-5,10-dioxo-3,4-dihydro-1H-benzo[g]isochromen-3-yl)acetate	[M+H]+	339.08389	C17H16O6	Benzoisochromanequinones	FMEUWIKCSICJBO-UHFFFAOYSA-N	O=C(OC)CC1OC(C=2C(=O)C=3C(O)=CC=CC3C(=O)C2C1)C
1692	5.729	881.39075	3-[(3S,10S,13R)-14-hydroxy-3-[4-methoxy-6-methyl-5-[3,4,5-trihydroxy-6-[[3,4,5-trihydroxy-6-(hydroxymethyl)oxan-2-yl]oxymethyl]oxan-2-yl]oxyoxan-	[M+H]+	881.39313	C42H66O17	Cardenolide glycosides and derivatives	ITKDSJDYFJAVTJ-BMRHGRNRSA-N	O=C1OCC(=C1)C2CCC3(O)C4CCC5CC(OC6OC(C)C(OC7OC(COC8OC(C O)C(O)C(O)C8O)C(O)C(O)C7O)C(OC)C6)CCC5(C)C4CCC23C
1693	5.73	289.08957	(2S,3S)-2-(3,4-dihydroxyphenyl)-3,7-dihydroxy-2,3-dihydrochromen-4-one	[M+2H]2+	289.08755	C15H12O6	Flavanonols	FNUPUYFWZXZMIE-CABCVRRESA-N	O=C1C2=CC=C(O)C=C2OC(C3=CC=C(O)C(O)=C3)C1O
1694	5.732	290.08984	(2R,3R,4S,5R)-2-(6-amino-9H-purin-9-yl)-5-(hydroxymethyl)tetrahydrofuran-3,4-diol	[M+2H]2+	290.09	C10H13N5O4	Purine nucleosides	OIRDTQYFTABQOQ-KQYNXXCUSA-N	OCC1OC(N2C=NC=3C(=NC=NC32)N)C(O)C1O
1695	5.732	879.37732	[(2R,3R,4R,5R,6S)-6-[(6E)-3,7-dimethyl-8-oxo-8-[(2S,3R,4S,5S,6R)-3,4,5-trihydroxy-6-(hydroxymethyl)oxan-2-yl]oxyocta-1,6-dien-3-yl]oxy-5-hydroxy-	[M+H]+	879.3775	C42H64O17	Saccharolipids	OIMUSOLEDHKDCV-WJHJMGTLSA-N	O=C(OC1OC(CO)C(O)C(O)C1O)C(=CCCC(OC2OC(CO)C(OC(=O)C(=C CCC(O)(C=C)C)C(OC(=O)C(=CCCC(O)(C=C)C)C)C2O)(C=C)C)C
1696	5.732	325.9588	Bifenox free acid	[M-H]-	325.96286	C13H7Cl2NO5	Diphenylethers	IUSYSZLVZMUVDO-UHFFFAOYSA-N	C1=CC(=C(C=C1Cl)Cl)OC=2C=CC(=C(C2)C(=O)O)N(=O)=O
1697	5.732	239.01831	Cystine	[M-H]-	239.01657	C6H12N2O4S2	L-cysteine-S-conjugates	LEVWYRKDKASIDU-UHFFFAOYNA-N	O=C(O)C(N)CSSCC(N)C(=O)O
1698	5.732	750.35034	N-((S)-1,2,3-trimethoxy-10-(((S)-4-methyl-1-oxo-1-(4-(piperidin-1-ylsulfonyl)-1,4-diazepan-1-yl)pentan-2-yl)amino)-9-oxo-5,6,7,9-	[M+H]+	750.34998	C37H53N5O8S	Leucine and derivatives	OJTOQAJKIWTRSX-SMCANUKXSA-N	O=C1C=C2C(=CC=C1NC(C(=O)N3CCN(CCC3)S(=O)(=O)N4CCCCC4)C C(C)C)C5=C(OC)C(OC)=C(OC)C=C5CCC2NC(=O)C
1699	5.738	282.01874	Pesticide3_Forchlorfenuron_C12H10ClN3O_Urea, N-(2-chloro-4-pyridinyl)-N'-phenyl-	[M-H]-	282.01999	C12H10ClN3O	2-halopyridines	GPXLRLUVLMHHIK-UHFFFAOYSA-N	C1C1=NC=CC(=C1)NC(O)=NC=2C=CC=CC2
1700	5.744	371.18033	Ingenol	[M+H]+	371.17999	C20H28O5	Tigliane and ingenane diterpenoids	VEBVPUXQAPLADL-WOVWOYSOSA-N	O=C1C2C=C(CO)C(O)C3(O)C(O)C(=CC13C(C)CC4C2C4(C)C)C
1701	5.748	738.47974	Phosphatidylethanolamine (16:0/18:2) Abbr: PLPE	[M+H]+	738.48199	C39H74NO8P	Phosphatidylethanolamines	HBZNVZIRJWODIB-NHCUFCNUSA-N	O=C(OCC(OC(=O)CCCCCCCC=CCC=CCCCCC)COP(=O)(O)OCCN)CCCCC CCCCCCCCCC
1702	5.761	399.19025	11,12-Methylenedioxykopsinaline	[M+H]+	399.19144	C22H26N2O5	Alkaloids	JVIKUDVTJCANPX-UHFFFAOYNA-N	COC(=O)C1(O)CC23CCCN4CCC5(C24)C2=CC=C4OCOC4=C2N C15CC3
1703	5.77	372.27298	5-[5-hydroxy-3-(hydroxymethyl)pentyl]-8a-(hydroxymethyl)-5,6-dimethyl-3,4,4a,6,7,8-hexahydronaphthalene-1-carboxylic acid	[M+H]+	372.27444	C20H34O5	Colensane and clerodane diterpenoids	VOCJFEUCJDTXSA-UHFFFAOYSA-N	O=C(O)C1=CCCC2C1(CO)CCC(C)C2(C)CCC(CO)CCO
1704	5.774	343.21155	FA 18:2+4O	[M-H]-	343.21219	C18H32O6	Oxidized fatty acids	BHWUKOCNOLCGLB-UHFFFAOYNA-N	O=C(O)CCCCCCCC(O)C=CC1OC(CC1(O))C(O)CC
1705	5.779	271.04926	(2S,3S)-3-hydroxy-4-methoxy-2-(2-phenylethyl)-2,3-dihydropyran-6-one	[M+H]+	271.05197	C14H16O4	Kavalactones	VJCNEDVMYQCMBK-FZMZJTMJSA-N	O=C1OC(CCC=2C=CC=CC2)C(O)C(OC)=C1
1706	5.78	391.14954	(2S)-8-[(E)-3-hydroxy-3-methylbut-1-enyl]-5,7-dimethoxy-2-phenyl-2,3-dihydrochromen-4-one	[M+H]+	391.15158	C22H24O5	7-O-methylated flavonoids	DKYFESWWIPJYTA-DVQDXYAYSA-N	O=C1C=2C(OC)=CC(OC)=C(C=CC(O)(C)C)C2OC(C=3C=CC=CC3)C1
1707	5.787	278.17386	PF1140_120159	[M+H]+	278.177	C16H23NO3	Chromenopyridines	YXWXVUSGNDTEJY-LRXDAGSUSA-N	O=C1C2=C(OC(C)C3(C)CC(C)CC(C)C23)C=CN1O
1708	5.791	257.10278	1,1'-biisoquinoline	[M+H]+	257.10001	C18H12N2	Bipyridines and oligopyridines	MVVRNAUFPUDQIB-UHFFFAOYSA-N	N=1C=CC=2C=CC=CC2C1C3=NC=CC4=CC=CC=C43
1709	5.796	380.12756	3-(3,5-dimethyl-7-oxo-7H-furo[3,2-g]chromen-6-yl)-N-(2-fluorophenyl)propanamide	[M+H]+	380.13	C22H18FNO4	Psoralens	WGJRLHFGQWIIHP-UHFFFAOYSA-N	O=C1OC=2C=C3OC=C(C3=CC2C(=C1CCC(=O)NC=4C=CC=CC4F)C)C
1710	5.799	242.13823	(E,2S,3R)-5-[3-hydroxy-2-(hydroxymethyl)phenyl]pent-4-ene-2,3-diol	[M+H]+	242.13867	C12H16O4	Cinnamyl alcohols	MCAIMPGCWVIODY-PHTUBKJSSA-N	OC1=CC=CC(C=CC(O)C(O)C)=C1CO
1711	5.799	521.3092	4-({5-[5-hydroxy-3-({[(2Z)-2-methylbut-2-enoyl]oxy}methyl)pentyl]-8a-(hydroxymethyl)-5,6-dimethyl-3,4,4a,5,6,7,8,8a-octahydronaphthalen-1-	[M-H]-	521.31195	C29H46O8	Colensane and clerodane diterpenoids	OVBBFGXDDIFFMB-SDPNRITHSA-N	O=C(OCC(CCO)CCC1(C)C(C)CCC2(C(=CCCC21)COC(=O)CCC(=O)O) CO)C(=CC)C
1712	5.805	367.20786	Hirsuteine	[M+H]+	367.20001	C22H26N2O3	Corynanthean-type alkaloids	TZUGIFAYWNNSAO-CPHCKLLXSA-N	O=C(OC)C(=COC)C1CC2C=3NC=4C=CC=CC4C3CCN2CC1C=C
1713	5.817	518.32831	Retapamulin	[M+H]+	518.33002	C30H47NO4S	Pleuromutilin and derivatives	STZYTFJPGGDRJD-FAJFTSBPSA-N	O=C(OC1CC(C=C)(C)C(O)C(C)C23CCC(=O)C2C1(C)C(C)CC3)CSC4 CC5N(C)C(CC5)C4
1714	5.819	344.10837	Omeprazole	[M-H]-	344.10742	C17H19N3O3S	Sulfinylbenzimidazoles	SUBDBMMJDZJVOS-UHFFFAOYNA-N	CC=1C=NC(CS(C2=NC=3C=CC(=CC3N2)OC)=O)=C(C)C1OC
1715	5.86	527.32709	Polygalacic acid	[M+H]+	527.33002	C30H48O6	Triterpenoids	KGGGRGBDMBZXKF-PWFIOIGMSA-N	O=C(O)C12CCC(C)(C)CC2C3=CCC4C5(C)CC(O)C(O)C(C)(CO)C5CC C4(C)C3(C)CC1O
1716	5.861	533.33875	Phytolaccagenin	[M+H]+	533.34003	C31H48O7	Triterpenoids	CYJWWQALTIKOAG-FLORRLIPSA-N	O=C(O)C12CCC(C(=O)OC)(C)CC2C3=CCC4C5(C)CC(O)C(O)C(C)(C O)C5CCC4(C)C3(C)CC1
1717	5.874	253.11693	Furanodienon	[M+H]+	253.12	C15H18O2	Germacrane sesquiterpenoids	XVOHELPNOXGRBQ-NXAIOARDSA-N	O=C1C=C(C)CCC=C(C)CC=2OC=C(C12)C
1718	5.891	340.21075	(3aS,3a1S,10bR,13S)-3a-ethyl-5-(methoxycarbonyl)-1,2,3,3a,3a1,4,6,11,12,13-decahydroindolizino[8,1-cd]carbazol-13-ium	[M+H]+	340.20999	C21H27N2O2+	Aspidospermatan-type alkaloids	GIGFIWJRTMBSRP-ACRUOGEOSA-O	O=C(OC)C1=C2NC=3C=CC=CC3C24CC[NH+]5CCCC(C1)(CC)C54

1719	5.897	238.10706	Bupropion	[M-H]-	238.10042	C13H18ClNO	Alkyl-phenylketones	SNPPWIUOZRMYNY-UHFFFAOYNA-N	O=C(C=1C=CC=C(C1)C1)C(NC(C)(C)C)C
1720	5.899	542.33514	(3E,7E,11E,15E)-5,9,13,17,18-pentahydroxy-4,6,8,10-tetramethyl-2-[(E)-2-methylbut-2-enyl]nonadeca-3,7,11,15-tetraenedioic acid	[M+H]+	542.33234	C28H44O9	Very long-chain fatty acids	YKAQENKKQWMYFZ-SLELSECNSA-N	O=C(O)C(O)C(O)C=CCC(O)C=CC(C)C(O)C(=CC(C)C(O)C(=CC(C(=O)O)CC(=CC)C)C)C
1721	5.939	296.10309	1-Methylguanosine	[M-H]-	296.10004	C11H15N5O5	Purine nucleosides	UTAIYTHAJQNQDW-UHFFFAOYNA-N	O=C1C=2N=CN(C2NC(=N)N1C)C3OC(CO)C(O)C3O
1722	5.941	213.0648	3-(2-hydroxyethyl)quinazolin-4(3H)-one	[M+H]+	213.06	C10H10N2O2	Quinazolines	OCFIUNGSKQZESJ-UHFFFAOYSA-N	O=C1C=2C=CC=CC2N=CN1CCO
1723	5.941	284.05457	Cladribine	[M-H]-	284.0556	C10H12ClN5O3	Purine 2'-deoxyribonucleosides	PTOAARAWEBMLNO-UHFFFAOYNA-N	C1C=1N=C(N)C=2N=CN(C2N1)C3OC(CO)C(O)C3
1724	5.943	285.03915	3',4',5,7-tetrahydroxyflavone	[M-H]-	285.04047	C15H10O6	Flavones	IQPNAANSBPBGFQ-UHFFFAOYSA-N	O=C1C=C(OC=2C=C(O)C=C(O)C12)C=3C=CC(O)=C(O)C3
1725	5.947	562.35303	(6aR,8aS)-10-(4-(N-acetylacetamido)-3-methylbutyl)-6a,8a,9-trimethyl-3,4,5,6,6a,6b,7,8,8a,8b,11a,12,12a,12b-tetradecahydro-1H-	[M+H]+	562.34998	C33H49NO5	Furostanes and derivatives	WURLGASLEZVNHV-PLECQVPFSA-N	O=C(OC1CC2=CCC3C(CCC4(C)C5C(=C(OC5CC34)CCC(C)CN(C(=O)C)C(=O)C)C)C2(C)CC1)C
1726	5.954	425.20551	Benazepril hydrochloride	[M+H]+	425.207	C24H29ClN2O5	Dipeptides	VPSRQEHTHIMDQM-FKLPMGAJSA-N	Cl.O=C(O)CN1C(=O)C(NC(C(=O)OCC)CCC=2C=CC=CC2)CCC=3C=CC=CC31
1727	5.954	268.11746	Citrinin	[M+H]+	268.11795	C13H14O5	Benzopyrans	CQIUKKVOEOPUDV-IYSWYEEDSA-N	O=C(O)C=1C(=O)C(=C2C(=COC(C)C2C)C1O)C
1728	5.979	393.16168	Corynanthin	[M+H]+	393.1575	C21H26N2O3	Yohimbine alkaloids	BLGXFZZNTVWLAY-UHFFFAOYSA-N	O=C(OC)C1C(O)CCC2CN3CCC=4C=5C=CC=CC5NC4C3CC12
1729	5.979	415.31894	diosgenin	[M+H]+	415.32001	C27H42O3	Triterpenoids	WQLVFSAGQJTQCK-VKROHFNGSA-N	OC1CC2=CCC3C(CCC4(C)C3CC5OC6(OCC(C)CC6)C(C)C54)C2(C)CC1
1730	5.982	577.36346	Polyphyllin A	[M+H]+	577.37	C33H52O8	Steroidal saponins	WXMARHKAXWRNDM-GAMIEDRGSA-N	OCC1OC(OC2CC3=CCC4C(CCC5(C)C4CC6OC7(OCC(C)CC7)C(C)C65)C3(C)CC2)C(O)C(O)C1O
1731	5.988	317.06497	3-O-methylquercetin	[M+H]+	317.065	C16H12O7	3-O-methylated flavonoids	WEPBGSIAWZTEJR-UHFFFAOYSA-N	O=C1C(OC)=C(OC=2C=C(O)C=C(O)C12)C=3C=CC(O)=C(O)C3
1732	6.001	464.09738	Delphinidin-3-glucoside	[M-H]-	464.09549	C21H21O12	Anthocyanidin-3-O-glycosides	XENHPQQLDPAYIJ-UHFFFAOYNA-O	OC=1C=C(O)C=2C=C(OC3OC(CO)C(O)C(O)C3O)C(=[O+]C2C1)C=4C=C(O)C(O)=C(O)C4
1733	6.004	251.16333	2-[(1S,2S,4aR,8aS)-1-hydroxy-4a-methyl-8-methylidene-1,2,3,4,5,6,7,8a-octahydronaphthalen-2-yl]prop-2-enoic acid	[M+H]+	251.16417	C15H22O3	Eudesmane, isoeudesmane or cycloeudesmane	QTSQCCVLTMKKDP-SFDCQRBFSA-N	O=C(O)C(=C)C1CCC2(C)CCCC(=C)C2C1O
1734	6.005	270.11206	(2Z)-2-benzylidene-6-methoxy-1-benzofuran-3(2H)-one	[M+H]+	270.11246	C16H12O3	Aurone flavonoids	LKWWJGGLULNRBP-DHDCSXOGSA-N	O=C1C(OC2=CC(OC)=CC=C21)=CC=3C=CC=CC3
1735	6.005	297.06497	(5E)-4-methoxy-5-[methoxy-[(2R,3S)-3-phenyloxiran-2-yl]methylidene]furan-2-one	[M+H]+	297.06296	C15H14O5	Butenolides	RTNGMIUMJUOABT-ONKYJICLSA-N	O=C1OC(C(OC)=C1)=C(OC)C2OC2C=3C=CC=CC3
1736	6.007	301.06006	Carbazochrome sulfonate	[M+H]+	301.06012	C10H12N4O5S	Indole and derivatives	OZCACMPSTYQSMM-UHFFFAOYNA-N	CN1C(CC2=CC(=NNC(O)=N)C(=O)C=C12)S(O)(=O)=O
1737	6.008	537.15936	(1S,4aS,7R,7aS)-7-hydroxy-7-[[(E)-3-(4-hydroxyphenyl)prop-2-enoyl]oxymethyl]-1-[(2S,3R,4S,5S,6R)-3,4,5-trihydroxy-6-	[M+H]+	537.16028	C25H28O13	Iridoid O-glycosides	WJPRKOJNQIZCGY-OZOLURDPSA-N	O=C(O)C1=COC(OC2OC(CO)C(O)C(O)C2O)C3C1C=CC3(O)COC(=O)C=CC4=CC=C(O)C=C4
1738	6.008	283.26233	Oleic_acid	[M+H]+	283.26401	C18H34O2	Long-chain fatty acids	ZQPPMHVWECSIRJ-MDZDMXLPSA-N	O=C(O)CCCCCCCC=CCCCCCCCC
1739	6.018	201.1127	Sebacic acid	[M-H]-	201.11324	C10H18O4	Medium-chain fatty acids	CXMXRPHRNRROMY-UHFFFAOYSA-N	OC(=O)CCCCCCCCC(O)=O
1740	6.019	301.03595	Ellagic Acid	[M-H]-	301.03583	C14H6O8	Hydrolyzable tannins	AFSDNFLWKVMVRB-UHFFFAOYSA-N	O=C1OC2=C(O)C(O)=CC=3C(=O)OC=4C(O)=C(O)C=C1C4C23
1741	6.021	581.14795	Flavanone base + 4O, O-Hex, C-Pen	[M-H]-	581.15021	C26H30O15	Flavanone C,O-glycosides	QETQPQDBDULWLC-UHFFFAOYNA-N	OCC1OC(OC2=CC(O)=C(C3OCC(O)C(O)C3O)C3=C2C(=O)CC(O3)C2=CC(O)=C(O)C=C2)C(O)C(O)C1O
1742	6.024	579.35144	(1S,2S,5R,7R,10S,11S,14R,16R,19S,20S,23R,25R)-5,14-diethyl-2,11,20,23-tetramethyl-4,13,22,28,29,30-	[M-H]-	579.35382	C32H52O9	Macrolides and analogues	MXZXIBZNZUOHER-VHWZNAHMSA-N	O=C1OC(C)CC2OC(CC2)C(C(=O)OC(CC)CC3OC(CC3)C(C(=O)OC(CC)CC4OC(CC4)C1C)C)C
1743	6.024	527.26678	3Beta-Acetoxydeoxodihydrogedunin	[M-H]-	527.26721	C30H40O8	Limonoids	LCTHKUJMKQOYDY-ZYIGBFDLSA-N	O=C(OC1CCC2(C)C3CCC4(C)C(OC(=O)C5OC54C3(C)C(OC(=O)C)CC2C1(C)C)C6=COC=C6)C
1744	6.03	375.10605	2-(3,4-dimethoxyphenyl)-5,7-dihydroxy-3,6-dimethoxy-4H-chromen-4-one	[M+H]+	375.10699	C19H18O8	6-O-methylated flavonoids	SDTFURCSGWUESP-UHFFFAOYSA-N	O=C1C(OC)=C(OC2=CC(O)=C(OC)C(O)=C12)C=3C=CC(OC)=C(OC)C3
1745	6.03	487.25217	Alpha-Dihydrogedunol	[M+H]+	487.25009	C28H38O7	Limonoids	IVHWWYNBMIROCV-AJIQNVKKSA-N	O=C(OC1CC2C(C)(C)C(O)CCC2(C)C3CCC4(C)C(OC(=O)C5OC54C13C)C6=COC=C6)C
1746	6.033	491.29947	caudatin	[M+H]+	491.29999	C28H42O7	Gluco/mineralocorticoids, progestogins and	VWLXIXALPNYWFH-UVJZLUDHSA-N	O=C(OC1CC2C(O)(CC=C3CC(O)CCC32C)C4(O)CCC(O)(C(=O)C)C14C)C=C(C)C(C)C
1747	6.034	272.12668	3-(alpha,alpha-dimethylallyl)psoralen	[M+H]+	272.12811	C16H14O3	Psoralens	FYCCCUNGXGKNJV-UHFFFAOYSA-N	O=C1OC=2C=C3OC=CC3=CC2C=C1C(C=C)(C)C
1748	6.034	524.3335	Phosphatidylcholine lyso 16	[M-H]-	524.33575	C24H48NO7P	2-acyl-sn-glycero-3-phosphocholines	GMEZONVWGDDWOJ-NXVVXOECNA-N	CCCCCCCCCCCC\C=C/CCC(=O)OC(CO)COP([O-])(=O)OCC[N+](C)(C)C
1749	6.054	453.19968	5-[(2R,3S)-6-hydroxy-2-(4-hydroxyphenyl)-4-[(E)-2-(4-hydroxyphenyl)ethenyl]-2,3-dihydro-1-benzofuran-3-yl]benzene-1,3-diol	[M-H]-	453.20023	C28H22O6	2-arylbenzofuran flavonoids	FQWLMRXWKZGLFI-PAKBJIOWSA-N	OC1=CC=C(C=C1)C=CC=2C=C(O)C=C3OC(C4=CC=C(O)C=C4)C(C=5C=C(O)C=C(O)C5)C32
1750	6.057	285.08676	(2R,3R)-3,7-dihydroxy-6-methoxy-2-phenyl-2,3-dihydrochromen-4-one	[M-H]-	285.0853	C16H14O5	6-O-methylated flavonoids	GUMFPOIJOXLCLA-JKSUJKDBSA-N	O=C1C2=CC(OC)=C(O)C=C2OC(C=3C=CC=CC3)C1O
1751	6.071	174.08405	Citrulline	[M-H]-	174.08841	C6H13N3O3	L-alpha-amino acids	RHGKLRLOHDJJDR-UHFFFAOYNA-N	O=C(O)C(N)CCCNC(=N)O
1752	6.085	765.43829	2-[2-[19-acetamido-6-(3,4-dicarboxybutanoyloxy)-16,18-dihydroxy-5,9-dimethylicosan-7-yl]oxy-2-oxoethyl]butanedioic aci	[M+H]+	765.43793	C36H61NO15	Fumonisins	GQCJWFPDXATUKS-UHFFFAOYSA-N	O=C(O)CC(C(=O)O)CC(=O)OC(CC(C)CCCCCCC(O)CC(O)C(N=C(O)C)C)C(OC(=O)CC(C(=O)O)CC(=O)O)C(C)CCCC
1753	6.09	487.21536	Vardenafil	[M-H]-	487.21329	C23H32N6O4S	Benzenesulfonamides	SECKRCOLJRRGGV-UHFFFAOYSA-N	CCCC1=NC(C)=C2C(N=C(C=3C=C(C=CC3OCC)S(N4CCN(CC)CC4)(=O)=O)NN12)=O
1754	6.096	622.28156	6-(furan-3-yl)-6,8,12,16,21-pentahydroxy-7,15-dimethyl-9-oxo-3,17,19-trioxaheptacyclo[9.9.3.0?,??.0?,?.0?,?.0??,??.0??,??]tricosan-14-yl 2-	[M+H]+	622.28577	C31H40O12	Limonoids	GSAWBDXYMSQMRT-UHFFFAOYSA-N	O=C(OC1CC(O)C23C4OCC5(C(O)CC2C1(C)C(O)O4)C3C(=O)C(O)C6(C)C(O)(C7=COC=C7)CC8OC856)C(C)CC
1755	6.098	294.13675	Pizotifen	[M-H]-	294.1322	C19H21NS	Cycloheptathiophenes	FIADGNVRKBPQEU-UHFFFAOYSA-N	S1C=CC2=C1CCC=3C=CC=CC3C2=C4CCN(C)CC4
1756	6.109	602.38922	dehydroxynocardamine	[M+H]+	602.38715	C27H48N6O8	Macrolactams	ABHHIGWFFMCQOC-UHFFFAOYSA-N	O=C1N(O)CCCCCN=C(O)CCC(=O)N(O)CCCCCN=C(O)CCC(O)=NCCCCCN=C(O)CC1
1757	6.123	867.37531	(8Z,8'Z)-8,8'-bis(((3,4-dimethoxyphenethyl)amino)methylene)-1,1',6,6'-tetrahydroxy-5,5'-diisopropyl-3,3'-dimethyl-[2,2'-binaphthalene]-	[M+H]+	867.38	C50H56N2O10	Lignans, neolignans and related compounds	KXCCPUJITGZBSJ-DQWRGTGXSA-N	O=C1C(O)=C(C=2C=C(C(=C(O)C2C1=CNCCC3=CC=C(OC)C(OC)=C3)C=4C(O)=C5C(=CNCCC6=CC=C(OC)C(OC)=C6)C(=O)C(O)=C(C5=CC
1758	6.134	361.20157	CORTISOL	[M-H]-	361.202	C21H30O5	21-hydroxysteroids	JYGXADMDTFJGBT-VWUMJDOOSA-N	O=C1C=C2CCC3C4CCC(O)(C(=O)CO)C4(C)CC(O)C3C2(C)CC1
1759	6.154	403.10098	5,7-dihydroxy-3,6-dimethoxy-2-(3,4,5-trimethoxyphenyl)-4H-chromen-4-one	[M-H]-	403.10101	C20H20O9	6-O-methylated flavonoids	GGNGEESEARSAMF-UHFFFAOYSA-N	O=C1C(OC)=C(OC2=CC(O)=C(OC)C(O)=C12)C=3C=C(OC)C(OC)=C(OC)C3
1760	6.158	243.15872	(Z)-2-octylpent-2-enedioic acid	[M+H]+	243.15907	C13H22O4	Medium-chain fatty acids	NIXDINZDFZJZHG-LUAWRHEFSA-N	O=C(O)C(=CCC(=O)O)CCCCCCCC
1761	6.158	287.06055	Dihydrokaempferol	[M-H]-	287.05612	C15H12O6	Flavanone O-glycosides	PADQINQHPQKXNL-UHFFFAOYNA-N	OC1C(OC2=CC(O)=CC(O)=C2C1=O)C1=CC=C(O)C=C1

1762	6.168	357.09528	Byakangelicin	[M+H]+	357.095	C17H18O7	5-methoxypsoralens	PKRPFNXROFUNDE-LLVKDONJSA-N	O=C1OC=2C(OCC(O)C(O)(C)C)=C3OC=CC3=C(OC)C2C=C1
1763	6.169	309.14288	Biflorin	[M+H]+	309.14301	C20H20O3	Naphthoquinones	DSVUOBIQVPVZST-UHFFFAOYSA-N	O=C1C(=O)C(=C2OC=C(C=3C=CC(=C1C23)C)CCC=C(C)C)C
1764	6.171	343.21268	ERGONOVINE MALEATE	[M+H]+	343.21283	C19H23N3O2	Lysergamides	WVVSZNPYNCNODU-XTQGRXLLSA-N	OC(=NC(C)CO)C1C=C2C=3C=CC=C4NC=C(C43)CC2N(C)C1
1765	6.172	495.28561	2-[(6,7-dimethoxy-1,2,3,4-tetrahydroisoquinolin-1-yl)methyl]-3-ethyl-9,10-dimethoxy-5-oxido-2,3,4,6,7,11b-hexahydro-1H-benzo[a]quinolizin-5-ium	[M-H]-	495.28644	C29H40N2O5	Emetine alkaloids	SSQAXSNCMRBPGJ-UHFFFAOYSA-N	O=N12CCC3=CC(OC)=C(OC)C=C3C2CC(CC4NCCC5=CC(OC)=C(OC)C=C54)C(CC)C1
1766	6.172	547.2948	Cymarine	[M-H]-	547.29126	C30H44O9	Cardenolide glycosides and derivatives	XQCGNURMLWFQJR-ZNDDOCHDSA-N	O=CC12CCC(OC3OC(C)C(O)C(OC)C3)CC2(O)CCC4C1CCC5(C)C(C6=CC(=O)OC6)CCC45O
1767	6.187	168.02977	N-(2-Furoyl)glycine	[M-H]-	168.03023	C7H7NO4	N-acyl-alpha amino acids	KSPQDMRTZZYQLM-UHFFFAOYSA-N	O=C(O)CNC(=O)C=1OC=CC1
1768	6.189	349.21048	Roxatidine acetate HCl	[M+H]+	349.21201	C19H29ClN2O4	N-benzylpiperidines	FEWCTJHCXOHWNL-UHFFFAOYSA-N	Cl.O=C(OCC(=O)NCCCOC1=CC=CC(=C1)CN2CCCCC2)C
1769	6.19	305.10461	15,17-dihydroxy-9-methyl-4,10-dioxatricyclo[11.4.0.0?,?]heptadeca-1(17),13,15-triene-2,11-dione	[M-H]-	305.10306	C16H18O6	Aryl alkyl ketones	BVDHPBILFRQGEC-UHFFFAOYSA-N	O=C1OC(C)CCCC2OC2C(=O)C=3C(O)=CC(O)=CC3C1
1770	6.217	897.38678	3-[(3S,5R,10S,13R,14S,17S)-14-hydroxy-3-[(2R,3S,4S,5S,6S)-3-hydroxy-4-methoxy-6-methyl-5-[(2S,3R,4S,5S,6R)-3,4,5-trihydroxy-6-	[M+H]+	897.38806	C42H66O18	Cardenolide glycosides and derivatives	GZVMBXDQUQRICT-OZPZHZNKSA-N	O=C1OCC(=C1)C2CCC3(O)C4CCC5CC(OC6OC(C)C(OC7OC(COC8OC(CO)C(O)C(O)C8O)C(O)C(O)C7O)C(OC)C6O)CCC5(C)C4CCC23C
1771	6.225	256.19073	3-hydroxy-5,5,8a-trimethyl-3,4,4a,6,7,8-hexahydronaphthalene-2-carboxylic acid	[M+H]+	256.1907	C14H22O3	Beta hydroxy acids and derivatives	LQZXAHKUYJVAAR-UHFFFAOYSA-N	O=C(O)C1=CC2(C)CCCC(C)(C)C2CC1O
1772	6.23	257.17358	alpha-dihydrolysergol	[M+H]+	257.17001	C16H20N2O	Clavines and derivatives	UFKTZIXVYHGAES-XEGUGMAKSA-N	OCC1CN(C)C2CC3=CNC4=CC=CC(=C43)C2C1
1773	6.235	584.2735	Tricoumaroyl spermidine (isomer of 2952)	[M+H]+	584.27441	C34H37N3O6	Coumaric acid and derivatives	PFDVWJCSCYDRMZ-UHFFFAOYSA-N	O=C(C=CC1=CC=C(O)C=C1)NCCCN(C(=O)C=CC2=CC=C(O)C=C2)CCCCNC(=O)C=CC3=CC=C(O)C=C3
1774	6.248	483.29504	(2R,3S,4S,5S,6R)-2-[[7-hydroxy-4-[(Z)-5-hydroxy-3-methylpent-3-enyl]-4a,8,8-trimethyl-3-methylidene-2,4,5,6,7,8a-hexahydro-1H-naphthalen-2-	[M-H]-	483.29633	C26H44O8	Diterpene glycosides	JECOCDKQCZSANH-RFKAOMIISA-N	OCC=C(C)CCC1C(=C)C(OC2OC(CO)C(O)C(O)C2O)CC3C(C)(C)C(O)CCC13C
1775	6.254	403.14877	3-[5-hydroxy-7-methoxy-2,3-dimethyl-6-(3-methylbut-2-enyl)-4-oxo-2,3-dihydrochromen-8-yl]hexanoic acid	[M-H]-	403.14771	C23H32O6	Chromones	UDVTWMLZUGXNQM-UHFFFAOYSA-N	O=C(O)CC(C1=C(OC)C(=C(O)C2=C1OC(C)C(C2=O)C)CC=C(C)C)CCC
1776	6.274	339.17966	coronardine	[M+H]+	339.17966	C21H26N2O2	Ibogan-type alkaloids	NVVDQMVGALBDGE-UHFFFAOYSA-N	O=C(OC)C12C=3NC=4C=CC=CC4C3CCN5CC(CC(CC)C51)C2
1777	6.275	345.18036	Formoterol	[M+H]+	345.18088	C19H24N2O4	Amphetamines and derivatives	BPZSYCZIITTYBL-UHFFFAOYNA-N	OC=NC1=CC(=CC=C1O)C(O)CNC(C)CC2=CC=C(OC)C=C2
1778	6.296	188.03595	Acetylisatin	[M-H]-	188.03532	C10H7NO3	Indoles and derivatives	LPGDEHBASRKTDG-UHFFFAOYSA-N	O=C1C(=O)N(C(=O)C)C=2C=CC=CC12
1779	6.296	271.10748	naringenin	[M-H]-	271.10629	C15H12O5	Flavanones	FTVWIRXFELQLPI-UHFFFAOYSA-N	O=C1C=2C(O)=CC(O)=CC2OC(C3=CC=C(O)C=C3)C1
1780	6.312	512.20221	(R,E)-6-acetyl-7,9-dihydroxy-2-(1-((3-(3-(2-methoxyethyl)-1,2,4-oxadiazol-5-yl)propyl)amino)ethylidene)-8,9b-dimethyldibenzo[b,d]furan-1,3(2H,9bH)-	[M+H]+	512.20001	C26H29N3O8	Acetophenones	GCKXCHKHBLNGAF-WGKDYUSESA-N	O=C1C=C2OC3=C(C(=O)C)C(O)=C(C(O)=C3C2(C(=O)C1=C(NCCCC4=NC(=NO4)CCOC)C)C)C
1781	6.312	539.16595	8-[(2R,3S)-5,7-dihydroxy-2-(4-hydroxyphenyl)-4-oxo-2,3-dihydrochromen-3-yl]-5,7-dihydroxy-2-(4-hydroxyphenyl)chromen-4-one	[M-H]-	539.16309	C30H20O10	Biflavonoids and polyflavonoids	YOGANETYFUQWIM-PXJZQJOASA-N	O=C1C=C(OC=2C1=C(O)C=C(O)C2C3C(=O)C=4C(O)=CC(O)=CC4OC3C5=CC=C(O)C=C5)C=6C=CC(O)=CC6
1782	6.316	250.0712	Phenylacetylaspartic acid	[M-H]-	250.0713	C12H13NO5	Amino acids	SVFKZPQPMMZHLZ-UHFFFAOYNA-N	O=C(O)CC(NC(=O)CC1=CC=CC=C1)C(=O)O
1783	6.325	877.35803	4-[(7R,11S,14S,16R)-14-[(4-methoxy-6-methyl-5-{[(2S,3R,4S,5S,6R)-3,4,5-trihydroxy-6-({[(2R,3R,4S,5S,6R)-3,4,5-trihydroxy-6-(hydroxymethyl)oxan-2-	[M+H]+	877.36182	C42H62O17	O-glycosyl compounds	DVFXEUYOOYUTOA-DYCWBZJYSA-N	O=C1OCC(=C1)C2=CCC34OC54CCC6CC(OC7OC(C)C(OC8OC(COC9OC(CO)C(O)C(O)C9O)C(O)C(O)C8O)C(OC)C7)CCC6(C)C5CCC23C
1784	6.326	879.3772	(7S,9E,11S,12R,13S,14S,15S,16R,17S,18S,19E,21Z)-13-(acetyloxy)-26-(dimethylamino)-15,17-dihydroxy-11-methoxy-3,7,12,14,16,18,22-heptamethyl-	[M+H]+	879.37	C47H56N2O13	Macrolactams	CHQPDRGLGJNQTJ-ZXWRESOLSA-N	O=C(OC=1C=2C(=O)C=3NC(=O)C(=CC=CC(C)C(O)C(C)C(O)C(C)C(OC(=O)C)C(C)C(OC)C=COC4(OC(=C(C4=O)C2C(=O)C3N(C)C)C1C)
1785	6.326	202.0515	Indole-3-pyruvic acid	[M-H]-	202.05096	C11H9NO3	Indolyl carboxylic acids and derivatives	RSTKLPZEZYGQPY-UHFFFAOYSA-N	O=C(O)C(=O)CC1=CNC=2C=CC=CC21
1786	6.328	649.27307	(S)-2-(((S)-7-acetamido-1,2,3-trimethoxy-9-oxo-5,6,7,9-tetrahydrobenzo[a]heptalen-10-yl)amino)-4-(methylthio)-N-(2-(2-	[M+H]+	649.27002	C32H42N4O7S	Methionine and derivatives	PMKXOQVXLVOSJT-ZCYQVOJMSA-N	O=C1C=C2C(=CC=C1NC(C(=O)NCCN3C(=O)CCC3)CCSC)C4=C(OC)C(OC)=C(OC)C=C4CCC2NC(=O)C
1787	6.337	211.13293	(+-)-Jasmonic acid	[M+H]+	211.13287	C12H18O3	Jasmonic acids	ZNJFBWYDHIGLCU-HWKXXFMVSA-N	CC\C=C/C[C=H]1[C=H](CC(O)=O)CCC1=O
1788	6.346	389.00107	PFAP-FT_diPAP	[M-H]-	389.00061	C8H9F10O4P	PFSA	ZPRPFGDNLHUJEV-UHFFFAOYSA-N	O=P(O)(OCCC(F)(F)F)OCCC(F)(F)C(F)(F)C(F)(F)F
1789	6.357	515.30316	Ganoderic acid LM2	[M+H]+	515.29999	C30H42O7	Triterpenoids	YVIVANDTNNUXRH-UGRVWEMWSA-N	O=C(O)C(=CC(O)CC(C)C1CC(=O)C2(C3=C(C(=O)CC12C)C4(C)CCC(=O)C(C)(C)C4CC3O)C)C
1790	6.358	307.12851	Caffeoyl lysine	[M-H]-	307.12729	C15H20N2O5	Caffeic acid and derivatives	WVFMZTORJFNZPK-UHFFFAOYNA-N	NC(CCCCNC(=O)C=CC1=CC(O)=C(O)C=C1)C(O)=O
1791	6.36	249.10913	2-hydroxyalantolactone	[M+H]+	249.10852	C15H20O3	Eudesmanolides, secoeudesmanolides, and	FZSKLHDEGWSLTB-ATYMOLOSSA-N	O=C1OC2CC3(C(=CC2C1=C)C(C)CC(O)C3)C
1792	6.36	229.03857	3-methyl-3,4-dihydro-1H-1,4-benzodiazepine-2,5-dione	[M+H]+	229.03738	C10H10N2O2	1,4-benzodiazepines	LJONQULKOKMKBR-UHFFFAOYSA-N	OC1=NC(C(O)=NC=2C=CC=CC12)C
1793	6.361	326.09048	Midazolam	[M+H]+	326.086	C18H13ClFN3	Imidazo[1,5-a][1,4]benzodiazepines	DDLIGBOFAVUZHB-UHFFFAOYSA-N	FC=1C=CC=CC1C2=NCC3=CN=C(N3C=4C=CC(Cl)=CC24)C
1794	6.366	369.18057	8-hydroxy-6-methoxy-7-[(2S,3R,4S,5S,6R)-3,4,5-trihydroxy-6-(hydroxymethyl)oxan-2-yl]oxychromen-2-one	[M-H]-	369.17978	C16H18O10	Coumarin glycosides	RVWWDDKOKZXKCJ-ZOLYHUHESA-N	O=C1OC=2C(O)=C(OC3OC(CO)C(O)C(O)C3O)C(OC)=CC2C=C1
1795	6.383	302.19473	heliocurassavicine n-oxyde	[M+H]+	302.194	C15H27NO5	Pyrrolizidines	DLNWZIVYKQXLTN-YOPIWALVSA-N	O=C(OCC1CC[N+]2([O-])CCCC12)C(O)(C(O)C)C(C)C
1796	6.384	431.314	Hecogenin	[M+H]+	431.31558	C27H42O4	Triterpenoids	QOLRLLFJMZLYQJ-LOBDNJQFSA-N	O=C1CC2C(CCC3CC(O)CCC32C)C4CC5OC6(OCC(C)CC6)C(C)C5C14C
1797	6.395	601.37	Timosaponin A1	[M+H]+	601.37	C33H54O8	Steroidal saponins	ZNEIIZNXGCIAAL-UHFFFAOYSA-N	OCC1OC(OC2CCC3(C)C(CCC4C3CCC5(C)C4CC6OC7(OCC(C)CC7)C(C)C65)C2)C(O)C(O)C1O
1798	6.411	429.11752	Formononetin-7-O-glucoside	[M-H]-	429.11911	C22H22O9	Isoflavone O-glycosides	MGJLSBDCWOSMHL-UHFFFAOYNA-N	COC1=CC=C(C=C1)C1=COC2=CC(OC3OC(CO)C(O)C(O)C3O)=CC=C2C1=O
1799	6.415	300.18002	Tegaserod	[M-H]-	300.18298	C16H23N5O	Indoles and derivatives	JWXDRFUZDKKGDV-UHFFFAOYSA-N	N=C(NNC=C1C=NC=2C=CC(OC)=CC21)NCCCCC
1800	6.436	375.27365	(E)-5-[(1S,4aR,8aR)-2-formyl-5,5,8a-trimethyl-1,4,4a,6,7,8-hexahydronaphthalen-1-yl]-3-(acetyloxymethyl)pent-2-enoic acid	[M-H]-	375.27643	C22H32O5	Diterpenoids	KLGPVOFVVYHEGO-VBIQWIQDSA-N	O=CC1=CCC2C(C)(C)CCCC2(C)C1CCC(=CC(=O)O)COC(=O)C
1801	6.436	545.27655	7-Deacetylkhivorin	[M+H]+	545.27448	C30H40O9	Limonoids	MRMHZWKIOFZZID-WNXWNNQMSA-N	O=C(OC1CC(OC(=O)C)C2(C)C(CC(O)C3(C)C2CCC4(C)C(OC(=O)C5OC543)C6=COC=C6)C1(C)C)C
1802	6.445	777.2204	methyl (1S,4aS,7aS)-5'-oxo-1-[(2S,3R,4S,5S,6R)-3,4,5-trihydroxy-6-(hydroxymethyl)oxan-2-yl]oxy-4'-[(1S)-1-[(E)-3-[4-[(2S,3R,4S,5S,6R)-3,4,5-	[M-H]-	777.22473	C36H42O19	Iridoid O-glycosides	AFYIWKNGSIYXCQ-WLJLAZGQSA-N	O=C(OC(C1=CC2(OC1=O)C=CC3C(=COC(OC4OC(CO)C(O)C(O)C4O)C32)C(=O)OC)C)C=CC5=CC=C(OC6OC(CO)C(O)C(O)C6O)C=C5
1803	6.447	481.34943	brassinolide	[M+H]+	481.35001	C28H48O6	Brassinolides and derivatives	IXVMHGVQKLDRKH-KNBKMWSGSA-N	O=C1OCC2C3CCC(C(C)C(O)C(O)C(C)C(C)C)C3(C)CCC2C4(C)CC(O)C(O)CC14
1804	6.452	303.25253	2-Myristoylglycerol	[M+H]+	303.25299	C17H34O4	2-monoacylglycerols	TVIMZSOUQXNWHO-UHFFFAOYSA-N	O=C(OC(CO)CO)CCCCCCCCCCCCC

1805	6.457	404.19101	Nifekalant	[M-H]-	404.19394	C19H27N5O5	Phenylpropylamines	OEBPANQZQGQPHF-UHFFFAOYSA-N	O=C1C=C(NCCN(CCO)CCCC2=CC=C(C=C2)N(=O)=O)N(C(=O)N1C)C
1806	6.462	135.06587	Pentostatin	[M+H]+	135.06599	C11H16N4O4	Imidazodiazepines	FPVKHBSQESCIEP-JQCXWYLXSA-N	OCC1OC(N2C=NC3=C2NC=NCC3O)CC1O
1807	6.47	220.09612	Pesticide3_3-Hydroxycarbofuran_C12H15NO4_3,7-Benzofurandiol, 2,3-dihydro-2,2-dimethyl-, 7-(methylcarbamate)	[M+H]+	220.09599	C12H15NO4	Coumarans	RHSUJRQZTQNSLL-UHFFFAOYSA-N	OC(=NC)OC1=CC=CC2=C1OC(C)(C)C2O
1808	6.471	395.21036	FA 18:0+2O+SO4	[M-H]-	395.2095	C18H36O7S	Oxidized fatty acids	SODLPGVKTPLRNA-UHFFFAOYNA-N	CCCCCCCCC(O)C(O)CCCCCCCC(=O)OS(O)(=O)=O
1809	6.473	511.12134	6''-O-Acetylglycitin	[M+H]+	511.12	C24H24O11	Isoflavonoid O-glycosides	DUBPGEJGGVZKDD-LKAHAZCJSA-N	O=C(OCC1OC(OC2=CC=3OC=C(C(=O)C3C=C2OC)C=4C=CC(O)=CC4)C(O)C(O)C1O)C
1810	6.489	289.17883	(16alpha,17beta)-estra-1,3,5(10)-triene-3,16,17-triol	[M+H]+	289.17999	C18H24O3	Estrogens and derivatives	PROQIPRRNZUXQM-ZXXIGWHRSA-N	OC1=CC=C2C(=C1)CCC3C2CCC4(C)C(O)C(O)CC34
1811	6.489	186.1132	Ecgonine	[M+H]+	186.11247	C9H15NO3	Tropane alkaloids	PHMBVCPLDPDESM-UHFFFAOYNA-N	O=C(O)C1C(O)CC2N(C)C1CC2
1812	6.497	487.26788	Tonantzitlolone A	[M+H]+	487.26801	C26H40O7	Fatty acid esters	YMEAIOHYSIGDJY-VCOIMRFZSA-N	O=C(OC1C(C)CC2OC1(O)C(O)C3(OC(CC3)C(C=CC(C2=O)C)(C)C)C)C=C(C)CC
1813	6.5	347.91959	Tetrachlorosalicylanilide	[M-H]-	347.9158	C13H7Cl4NO2	P-chlorophenols	BDOBMVIEWHZYDL-UHFFFAOYSA-N	C1=CC=C(C=C1)N=C(C2=C(C(=C(C(=C2O)C1)C1)C1)C1)O
1814	6.508	383.11044	2-[2-hydroxy-1-(4-methoxyphenyl)propoxy]-6-(hydroxymethyl)oxane-3,4,5-triol	[M+H]+	383.11026	C16H24O8	O-glycosyl compounds	IBLFDTIZHJLTIF-UHFFFAOYSA-N	OCC1OC(OC(C2=CC=C(OC)C=C2)C(O)C)C(O)C(O)C1O
1815	6.516	676.35193	Euphorbia factor L14 - Lathyrane diterpenoid	[M+H]+	676.34601	C38H42O10	Diterpenoids	JBQJQRQPOXLFNB-IEWKGUOQSA-N	O=C(OC1C(C)CC2(OC(=O)C)C(=O)C(=CC3C(CC(OC(=O)C=4C=CC=CC4O)C(=C)C(OC(=O)C)C12)C3(C)C)C)C=5C=CC=CC5
1816	6.519	216.19469	Rhodinyl Acetate	[M+H]+	216.1958	C12H22O2	Fatty alcohol esters	JOZKFWLRHCDGJA-UHFFFAOYSA-N	O=C(OCCC(C)CCC=C(C)C)C
1817	6.521	199.16882	delta-Dodecalactone	[M+H]+	199.16925	C12H22O2	Delta valerolactones	QRPLZGZHJABGRS-UHFFFAOYNA-N	CCCCCCCC1CCCC(=O)O1
1818	6.532	427.19339	3-[(2R,3S)-5-hydroxy-2,3,8,8-tetramethyl-4-oxo-2,3-dihydropyrano[2,3-h]chromen-6-yl]hexanoic acid	[M+H]+	427.19211	C22H28O6	Pyranochromenes	MXFUJXIOAUPXPT-LAGVYOHYSA-N	O=C(O)CC(C1=C(O)C=2C(=O)C(C)C(OC2C=3C=CC(OC31)(C)C)C)CCC
1819	6.532	278.21088	Venlafaxine	[M+H]+	278.211	C17H28ClNO2	Anisoles	QYRYFNHXARDNFZ-UHFFFAOYSA-N	Cl.OC1(CCCCC1)C(C2=CC=C(OC)C=C2)CN(C)C
1820	6.536	288.25256	Lauryl diethanolamide	[M+H]+	288.25333	C16H33NO3	N-acyl amines	AOMUHOFOVNGZAN-UHFFFAOYSA-N	CCCCCCCCCCCC(N(CCO)CCO)=O
1821	6.54	319.26248	bilobol	[M+H]+	319.26315	C21H34O2	Resorcinols	TUGAUFMQYWZJAB-FPLPWBNLSA-N	OC=1C=C(O)C=C(C1)CCCCCCCC=CCCCCCC
1822	6.543	381.11511	3-[4-methoxy-2-[(2S,3R,4S,5S,6R)-3,4,5-trihydroxy-6-(hydroxymethyl)oxan-2-yl]oxyphenyl]propanoic acid	[M+H]+	381.1156	C16H22O9	Phenolic glycosides	LIVNOUBJFOXZOR-YMILTQATSA-N	O=C(O)CCC1=CC=C(OC)C=C1OC2OC(CO)C(O)C(O)C2O
1823	6.543	474.30865	Tomatidine HCl	[M+H]+	474.31	C27H46ClNO2	Spirosolanes and derivatives	SXXHVPYRDFJKPG-VXJLCZPPSA-N	Cl.OC1CCC2(C)C(CCC3C2CCC4(C)C3CC5OC6(NCC(C)CC6)C(C)C54)C1
1824	6.557	407.29913	(3R,5R)-7-[(1S,2S,8S,8aR)-2-methyl-8-(2-methylbutanoyloxy)-1,2,6,7,8,8a-hexahydronaphthalen-1-yl]-3,5-dihydroxyheptanoic acid	[M-H]-	407.29395	C23H36O6	Medium-chain hydroxy acids and derivatives	BOZILQFLQYBIIY-PJXHJHJJSA-N	O=C(O)CC(O)CC(O)CCC1C(C=CC2=CCCC(OC(=O)C(C)CC)C21)C
1825	6.578	257.22168	5-beta-Androstane-3-alpha,17-beta-diol	[M+H]+	257.22638	C19H32O2	Androgens and derivatives	CBMYJHIOYJEBSB-GCFMDFLISA-N	CC12CCC3C(CC[C=H]4C[C=H](O)CCC34C)C1CC[C=H]2O
1826	6.592	809.43079	6-[[(2R,3R,6aR,6bS,8aS,12aS,14bR)-2-hydroxy-4,4,6a,6b,11,11,14b-heptamethyl-8a-[3,4,5-trihydroxy-6-(hydroxymethyl)oxan-2-yl]oxycarbonyl-	[M-H]-	809.42987	C42H66O15	Triterpene saponins	GZNCPAASLBFTKY-BIOGTAEPSA-N	O=C(O)C1OC(OC2C(O)CC3(C)C4CC=C5C6CC(C)(C)CCC6(C(=O)OC7OC(CO)C(O)C(O)C7O)CCC5(C)C4(C)CCC3C2(C)C)C(O)C(O)C1O
1827	6.601	483.23465	3-Deoxo-3Beta-Hydroxymexicanolide 16-Enol Ether	[M-H]-	483.23724	C28H36O7	Naphthopyrans	WJHQHAFOOMOTBJ-MSHADUKESA-N	O=C(OC)CC1C2(C(=O)C(CC3=C4C=C(OC)OC(C5=COC=C5)C4(C)CCC32)C(O)C1(C)C)C
1828	6.612	202.21573	DDAO	[M+H]+	202.21654	C12H27NO	Long-chain alkyl amine oxides	ZRKZFNZPJKEWPC-UHFFFAOYSA-N	CCCCCCCCCCN(C)(C)=O
1829	6.623	507.14731	[(2R,3S,4S,5R,6R)-3,4,5-trihydroxy-6-[2-(3-hydroxy-5-oxooxolan-3-yl)propoxy]oxan-2-yl]methyl (E)-3-(3,4-dihydroxyphenyl)prop-2-enoate	[M+H]+	507.14728	C22H28O12	Coumaric acids and derivatives	YNPOIPFNQJKGQJ-SALKWGHLSA-N	O=C(OCC1OC(OCC(C)C2(O)COC(=O)C2)C(O)C(O)C1O)C=CC3=CC=C(O)C(O)=C3
1830	6.627	277.25204	2-heptadecanone	[M+H]+	277.25	C17H34O	Ketones	TVTCXPXLRKTHAU-UHFFFAOYSA-N	O=C(C)CCCCCCCCCCCCCCC
1831	6.641	182.11716	3,4-Dimethoxyphenethylamine	[M+H]+	182.117	C10H15NO2	Dimethoxybenzenes	ZHSFEDDRTVLPHH-UHFFFAOYSA-N	O(C=1C=C(OC)C=C(C1)CCN)C
1832	6.652	380.16968	N-(1-oxo-1-(3,5,6,7-tetrahydrospiro[imidazo[4,5-c]pyridine-4,4'-piperidin]-1'-yl)propan-2-yl)furan-2-carboxamide	[M+H]+	380.17001	C18H23N5O3	N-acyl-alpha amino acids and derivatives	JQBOIGWHYPDAGZ-UHFFFAOYSA-N	O=C(NC(C(=O)N1CCC2(NCCC=3N=CNC32)CC1)C)C=4OC=CC4
1833	6.656	389.21774	(2R,3R,4S,5S,6R)-2-[6-hydroxy-3-[(E)-3-hydroxybut-1-enyl]-2,4,4-trimethylcyclohexyl]oxy-6-(hydroxymethyl)oxane-3,4,5-triol	[M-H]-	389.21808	C19H34O8	Terpene glycosides	FPWYMVMNRFRHQC-BVWWHRDXSA-N	OCC1OC(OC2C(O)CC(C)(C)C(C=CC(O)C)C2C)C(O)C(O)C1O
1834	6.66	527.35828	12-(acetyloxy)-6-(furan-3-yl)-14-hydroxy-1,7,11,15,15-pentamethyl-5-oxo-3-oxapentacyclo[8.8.0.0?,?.0?,?.0??,??]octadecan-18-yl acetate	[M-H]-	527.35876	C30H40O8	Limonoids	LZMUZSFHEIXIJR-UHFFFAOYSA-N	O=C(OC1CC(O)C(C)(C)C2CC(OC(=O)C)C3(C)C(CCC4(C)C(C(=O)C5OC543)C6=COC=C6)C12C)C
1835	6.672	303.21649	Aleuretic Acid	[M-H]-	303.21768	C16H32O5	Long-chain fatty acids	MEHUJCGAYMDLEL-LSDHHAIUSA-N	O=C(O)CCCCCCCC(O)C(O)CCCCCCO
1836	6.676	238.14369	Albuterol	[M-H]-	238.14487	C13H21NO3	Benzyl alcohols	NDAUXUAQIAJITI-UHFFFAOYNA-N	OC1=CC=C(C=C1CO)C(O)CNC(C)(C)C
1837	6.681	943.48547	3,4,5-trihydroxy-6-(hydroxymethyl)oxan-2-yl (2E,6R)-6-[(1R,3R,6S,8R,12S,15R,16R)-13,17-dihydroxy-7,7,12,16-tetramethyl-6-{[(2R)-	[M-H]-	943.49078	C47H76O19	Cucurbitacin glycosides	NZBYUSXAWFZDAY-SDCBSSCYSA-N	O=C(OC1OC(CO)C(O)C(O)C1O)C(=CCCC(C)C2CC(O)C3(C)C4CCC5C(C)(C)C(OC6OC(COC7OCC(O)C(O)C7O)C(O)C(O)C6O)CCC85CC48C
1838	6.688	583.20563	Tanegoside (Not validated)	[M-H]-	583.20441	C26H34O12	Lignols	WMABCPOXSNGIJO-UHFFFAOYNA-N	OC=1C=CC(=CC=1(OC))C4OCC(C(OC2OC(CO)C(O)C(O)C2(O))C3=CC=C(O)C(OC)=C3)C4(CO)
1839	6.691	645.2395	Irinotecan Hydrochloride	[M+H]+	645.23999	C33H39ClN4O6	Camptothecins	GURKHSYORGJETM-WAQYZQTGSA-N	Cl.O=C(OC=1C=CC2=NC=3C4=CC5=C(C(=O)N4CC3C(=C2C1)CC)COC(=O)C5(O)CC)N6CCC(N7CCCCC7)CC6
1840	6.704	511.19415	Alpha-Alectoronic acid	[M-H]-	511.19101	C28H32O9	Depsides and depsidones	ZDIOCINAHISORH-UHFFFAOYSA-N	O=C(O)C1=C(O)C=C2OC(=O)C=3C(OC2=C1CC(=O)CCCCC)=CC(O)=C3CC(=O)CCCCC
1841	6.709	574.35779	Phosphatidylcholine lyso 20	[M-H]-	574.35144	C28H50NO7P	2-acyl-sn-glycero-3-phosphocholines	XHKVQFBZSMLFNL-AILJCPQKNA-N	CCCCCC\C=C/C\C=C/C\C=C/C\C=C/CCC(=O)OC(CO)COP([O-])(=O)OCC[N+](C)(C)C
1842	6.731	513.2431	methyl (1R,4S,4aS,7R,8S,9R)-4,8,9-triacetyloxy-7-ethenyl-1,4a,7-trimethyl-3,4,5,6,8,9,10,10a-octahydro-2H-phenanthrene-1-carboxylate	[M+H]+	513.24585	C27H38O8	16-oxosteroids	HPLALXILNGTIHC-WJKVYVNYSA-N	O=C(OC1C2=C(CCC(C=C)(C)C2OC(=O)C)C3(C)C(OC(=O)C)CCC(C(=O)OC)(C)C3C1)C
1843	6.734	467.20972	ginnalin A	[M-H]-	467.21286	C20H20O13	Galloyl esters	IGKWMHUOBKCUQU-LTCOOKNTSA-N	O=C(OCC1OCC(OC(=O)C2=CC(O)=C(O)C(O)=C2)C(O)C1O)C3=CC(O)=C(O)C(O)=C3
1844	6.736	538.35596	Phosphatidylcholine lyso 17	[M-H]-	538.35144	C25H50NO7P	2-acyl-sn-glycero-3-phosphocholines	WRNNGLADZYOSJO-MSUUIHNZNA-N	CCCCCCCCCCCC\C=C/CCC(=O)OC(CO)COP([O-])(=O)OCC[N+](C)(C)C
1845	6.748	350.23053	9-hydroxy-7-(2-hydroxypropan-2-yl)-1,4a-dimethyl-2,3,4,9,10,10a-hexahydrophenanthrene-1-carboxylic acid	[M+H]+	350.23257	C20H28O4	Diterpenoids	ALGYTGOYQATWBA-UHFFFAOYSA-N	O=C(O)C1(C)CCCC2(C3=CC=C(C=C3C(O)CC12)C(O)(C)C)C
1846	6.748	268.15479	Rizatriptan	[M-H]-	268.15677	C15H19N5	Tryptamines and derivatives	ULFRLSNUDGIQQP-UHFFFAOYSA-N	N=1C=NN(C1)CC=2C=CC=3NC=C(C3C2)CCN(C)C
1847	6.762	477.23862	[3,4,5-trihydroxy-6-[[(E)-3-(4-hydroxyphenyl)prop-2-enoyl]oxymethyl]oxan-2-yl] 3,4,5-trihydroxybenzoate	[M-H]-	477.23224	C22H22O12	Tannins	LWOXGQKLDYQLMZ-ZZXKWVIFSA-N	O=C(OCC1OC(OC(=O)C2=CC(O)=C(O)C(O)=C2)C(O)C(O)C1O)C=CC3=CC=C(O)C=C3

1848	6.763	527.15222	(1S,4aS,7aS)-7-[[(E)-3-phenylprop-2-enoyl]oxymethyl]-1-[(2S,3R,4S,5S,6R)-3,4,5-trihydroxy-6-(hydroxymethyl)oxan-2-yl]oxy-1,4a,5,7a-	[M+H]+	527.15234	C25H28011	Iridoid O-glycosides	VTQUQEWGIJRVHB-VHPPWYILSA-N	O=C(O)C1=COC(OC2OC(CO)C(O)C(O)C2O)C3C(=CCC13)COC(=O)C=CC=4C=CC=CC4
1849	6.772	302.13748	Nicotianamine	[M-H]-	302.13577	C12H21N306	L-alpha-amino acids	KRGPXXHMOXVMMM-CIUDSAMLSA-N	N[C=H](CCN[C=H](CCN1CC[C=H]1C(O)=O)C(O)=O)C(O)=O
1850	6.785	651.18842	(R)-3-(2,3-dihydrobenzo[b][1,4]dioxin-6-yl)-4-oxo-2-(trifluoromethyl)-4H-chromen-7-yl 2-((tert-butoxycarbonyl)amino)-3-(1H-indol-3-yl)propanoate	[M+H]+	651.19	C34H29F3N208	Isoflavones	ORGFYMVNMFQORV-XMMPIXPASA-N	O=C(OC(C)(C)C)NC(C(=O)OC1=CC=C2C(=O)C(C=3C=CC=4OCCOC4C3)=C(OC2=C1)C(F)(F)F)CC5=CNC=6C=CC=CC65
1851	6.817	197.15352	Chrysanthemic Acid, Ethyl Ester	[M+H]+	197.15359	C12H2002	Monocyclic monoterpenoids	VIMXTGUGWLAOFZ-UHFFFAOYSA-N	O=C(OCC)C1C(C=C(C)C)C1(C)C
1852	6.82	819.23462	Smiglaside C (Not validated)	[M-H]-	819.23499	C38H44020	Ferulic acid and derivatives	VIYLZUDAAGEJSD-UHFFFAOYNA-N	O=C(OCC3OC(OC1OC(COC(=O)C)C(OC(=O)C)C(O)C1(OC(=O)C))(CO)C(O)C(O)C3O)CCC5(C)C4(C)CCC3C2(C)CO)C(O)C(O)C1O
1853	6.831	361.09164	Rosmarinic acid	[M+H]+	361.0918	C18H1608	Coumaric acids and derivatives	DOUMFZQKYFQNTF-WUTVXBCWSA-N	OC(=O)[C=H](CC1=CC(O)=C(O)C=C1)OC(=O)\C=C\C1=CC(O)=C(O)C=C1
1854	6.834	263.08026	indirubin	[M+H]+	263.07999	C16H10N202	Indolines	CRDNMYFJWFXOCH-BUHFOSPRSA-N	O=C1NC=2C=CC=CC2C1=C3NC=4C=CC=CC4C3=O
1855	6.838	849.36938	(2S,3S,4S,5R,6R)-6-[[(3S,4R,6aR,6bS,8aS,14bR)-4-(hydroxymethyl)-4,6a,6b,11,11,14b-hexamethyl-8a-[(2S,3R,4S,5S,6R)-3,4,5-trihydroxy-6-	[M+H]+	849.37683	C42H66015	Triterpene saponins	RZQHWSDMLZHIRN-MCTPBCADSA-N	O=C(O)C1OC(OC2CCC3(C)C4CC=C5C6CC(C)(C)CCC6(C(=O)OC7OC(CO)C(O)C(O)C7O)CCC5(C)C4(C)CCC3C2(C)CO)C(O)C(O)C1O
1856	6.843	425.18719	Clindamycin	[M+2H]2+	425.18713	C18H33C1N205S	Proline and derivatives	KDLRVYVGXIQJDK-NOWPCOIGSA-N	CCC[C=H]1C[C=H](N(C)C1)C(O)=NC(C(C)Cl)[C=H]1O[C=H](SC)[C=H](O)[C=H](O)[C=H]1O
1857	6.856	385.15005	2-(1-(benzo[d][1,3]dioxol-5-ylmethyl)-2-hydroxy-5-oxo-4,5-dihydro-1H-imidazol-4-yl)-N-((1-methyl-1H-pyrrol-2-yl)methyl)acetamide	[M+H]+	385.14999	C19H20N405	Hydantoins	MCCCQAKRHCMRDG-UHFFFAOYSA-N	O=C(NCC1=CC=CN1C)CC2N=C(O)N(C2=O)CC3=CC=C4OCOC4=C3
1858	6.859	401.21713	CAPERATIC ACID	[M-H]-	401.2226	C21H3807	Tricarboxylic acids and derivatives	HMJJWFFZRCLIDN-UHFFFAOYSA-N	O=C(OC)CC(O)(C(=O)O)C(C(=O)O)CCCCCCCCCCCCCC
1859	6.86	313.05972	Catechin	[M+H]+	313.06	C15H1406	Catechins	PFTAWBLQPZVEMU-DZGCQCFKSA-N	OC=1C=C(O)C2=C(OC(C3=CC=C(O)C(O)=C3)C(O)C2)C1
1860	6.863	315.08347	methyl (1S,4S,8R,11S,14S)-11-ethyl-12-oxo-7,9,13-trioxatetracyclo[6.5.1.0?,??.0?,??]tetradeca-2,5-diene-5-carboxylate	[M+H]+	315.08389	C15H1606	Terpene lactones	LEYGRBGYJYVHJL-ZKQFBGJWSA-N	O=C(OC)C1=COC2OC3C(C(=O)OC43C=CC1C24)CC
1861	6.867	363.19339	4-benzoyl-9,13,13-trimethyl-8-oxatetracyclo[7.4.1.0?,?.0??,??]tetradeca-2,4,6-triene-3,5-diol	[M-H]-	363.1936	C23H2404	Benzophenones	BQIVXBUAUNSLAJ-UHFFFAOYSA-N	O=C(C=1C=CC=CC1)C2=C(O)C=C3OC4(C)CCC5C4C(C3=C2O)C5(C)C
1862	6.868	519.25415	Deacetoxy(7)-7-Oxokhivorinic Acid	[M-H]-	519.25684	C27H36010	Naphthopyrans	WROKSYFOSDWFHL-UQVLWQGYSA-N	O=C(OC1CC(OC(=O)C)C2(C)C(CC(=O)C3(C)C2CCC4(C)C(OC(=O)C5OC534)C(=O)O)C1(C)C)C
1863	6.868	203.09045	Metamitron	[M+H]+	203.09274	C10H10N40	1,2,4-triazines	VHCNQEUWZYOAEV-UHFFFAOYSA-N	CC1=NN=C(C2=CC=CC=C2)C(N1N)=O
1864	6.87	383.14978	methyl (1S,12S,13E)-13-ethylidene-18-(hydroxymethyl)-4-methoxy-8,15-diazapentacyclo[10.5.1.0?,?.0?,?.0??,??]octadeca-2,4,6,8-tetraene-18-	[M+H]+	383.14841	C22H26N204	Corynanthean-type alkaloids	JBFBTBFJYNNYPW-ZXWRBSQSSA-N	O=C(OC)C1(CO)C2C(=CC)CN3CCC41C=5C=C(OC)C=CC5N=C4C3C2
1865	6.87	277.12698	Triethylcitrate	[M+H]+	277.12817	C12H2007	Tricarboxylic acids and derivatives	DOOTYTYQINUNNV-UHFFFAOYSA-N	CCOC(CC(CC(=O)OCC)(C(=O)OCC)O)=O
1866	6.872	395.20297	2,4-dihydroxy-5-methoxy-1'',6'',8''-trimethyldispiro[bis(oxolane)-3,2':5',7''-[3]oxatricyclo[6.3.1.0?,??]dodecane]-2''-one	[M-H]-	395.20752	C21H3207	O-glycosyl compounds	JFEFKYNVTFPFRI-UHFFFAOYSA-N	O=C1OC2CC(C)C3(OC4(CC3)C(O)OC(OC)C4O)C5(C)CCCC1(C)C25
1867	6.874	327.21576	FA 18:2+3O	[M-H]-	327.21631	C18H3205	Oxidized fatty acids	MKYUCBXUUSZMQB-UHFFFAOYNA-N	O=C(O)CCCCCCCC(O)C=CC(O)C(O)CC=CCC
1868	6.874	357.13553	Matairesinol	[M-H]-	357.13437	C20H2206	Lignols	MATGKVZWFZHCLI-UHFFFAOYNA-N	COC1=CC(CC2COC(=O)C2CC2=CC=C(O)C(OC)=C2)=CC=C1O
1869	6.877	765.43903	Lirioprolioside B	[M+H]+	765.44	C41H64013	Steroidal saponins	KSIVGTKSVYIZEB-GDUJLLAZSA-N	O=C(OC1C(O)C(OC(C)C1O)OC2C(OC(C)C(O)C2O)OC3CC(O)CC4=CC5C(CCC6(C)C5CC7OC8(OCC(C)CC8)C(C)C76)C43C)C
1870	6.878	805.43445	[(2S,3R,4S,5S,6R)-3,4,5-trihydroxy-6-(hydroxymethyl)oxan-2-yl] (4aS,6aS,6bR,9R,10R,11S,12aR)-11-hydroxy-9-(hydroxymethyl)-	[M+H]+	805.43445	C41H66014	Triterpene saponins	RTFKMVMUZLBFEW-HEUPFQDZSA-N	O=C(OC1OC(CO)C(O)C(O)C1O)C23CCC(C)(C)CC3C4=CCC5C6(C)CC(O)C(OC7OCC(O)C(O)C7O)C(C)(CO)C6CCC5(C)C4(C)CC2
1871	6.88	471.34668	Enoxolone	[M+H]+	471.34686	C30H4604	Triterpenoids	MPDGHEJMBKOTSU-YKLVYJNSSA-N	O=C(O)C1(C)CCC2(C)CCC3(C(=CC(=O)C4C5(C)CCC(O)C(C)(C)C5CCC43C)C2C1)C
1872	6.887	349.20126	8-(3-((1-ethoxy-2-methyl-1-oxopropan-2-yl)oxy)phenyl)octanoic acid	[M-H]-	349.20099	C20H3005	Phenoxyacetic acid derivatives	BLLVLMYXVKOFGW-UHFFFAOYSA-N	O=C(O)CCCCCCCC=1C=CC=C(OC(C(=O)OCC)(C)C)C1
1873	6.893	879.37744	(7S,9E,11S,12R,13S,14S,15S,16R,17S,18S,19E,21Z)-2,15,17-trihydroxy-11-methoxy-3,7,12,14,16,18,22-heptamethyl-6,23,27,29-tetraoxo-26-[4-(pyridin-	[M+H]+	879.38	C46H56N4012	Macrolactams	OTBYDWMNPJJQRG-KLDNQBIDSA-N	O=C(OC1C(C)C(OC)C=COC2(OC3=C(C2=O)C=4C(=O)C(=C(NC(=O)C(=CC=CC(C)C(O)C(C)C(O)C1C)C)C(=O)C4C(O)=C3C)N5CCN(C6=N
1874	6.908	465.22546	1-[(2E,4E)-6,7-Dihydroxy-2,4-octadienoyl]prolyl-N-methylvalyl-N2-methylalaninamide	[M-H]-	465.22525	C23H38N406	Dipeptides	YHEIGYJZSXWHJB-MKICQXMISA-N	O=C(C=CC=CC(O)C(O)C)N1CCCC1C(=O)N(C)C(C(=O)N(C)C(C(=N)O)C)C(C)C
1875	6.913	349.25797	Oxyphenonium	[M+H]+	349.26059	C21H34N03	Benzene and substituted derivatives	GFRUPHOKLBPHTQ-UHFFFAOYNA-N	O=C(OCC[N+](C)(CC)CC)C(O)(C=1C=CC=CC1)C2CCCCC2
1876	6.921	395.19562	Deguelin(-)	[M+H]+	395.19589	C23H2206	Rotenones	ORDAZKGHSNRHTD-UXHICEINSA-N	O=C1C2=CC=C3OC(C=CC3=C2OC4COC5=CC(OC)=C(OC)C=C5C14)(C)C
1877	6.923	519.29565	(3S,4S,5R)-4-[(2R,3R)-2,3-dihydroxy-3-[(2S,3R,5R,10R,13R,14S,17S)-2,3,14-trihydroxy-10,13-dimethyl-6-oxo-2,3,4,5,9,11,12,15,16,17-decahydro-1H-	[M-H]-	519.29633	C29H4408	Triterpenoids	NEFYSBQJYCICOG-RJRZVDSCSA-N	O=C1OC(C)C(CC(O)C(O)(C)C2CCC3(O)C4=CC(=O)C5CC(O)C(O)CC5(C)C4CCC23C)C1C
1878	6.955	600.33636	destruxin A	[M+H]+	600.33673	C29H47N507	Cyclic depsipeptides	XIYSEKITPHTMJT-UHFFFAOYSA-N	O=C1OC(C(=O)N2CCCC2C(O)=NC(C(=O)N(C)C(C(=O)N(C)C(C(O)=NCC1)C)C(C)C)C(C)CC)CC=C
1879	6.958	221.08014	3,5-dimethyl-7-oxo-6-oxabicyclo[3.2.1]octane-2-carboxylic acid	[M+H]+	221.08	C10H1404	Gamma butyrolactones	BTIKJPRUQUGBSU-UHFFFAOYSA-N	O=C(O)C1C2C(=O)OC(C)(C2)CC1C
1880	6.963	461.23773	[(2S,3R,4S,5S,6R)-3,4,5-trihydroxy-6-[[(E)-3-phenylprop-2-enoyl]oxymethyl]oxan-2-yl] 3,4,5-trihydroxybenzoate	[M-H]-	461.23715	C22H22011	Tannins	REBRERQNUHGTMS-PCGJYRBUSA-N	O=C(OCC1OC(OC(=O)C2=CC(O)=C(O)C(O)=C2)C(O)C(O)C1O)C=CC=3C=CC=CC3
1881	6.966	415.13657	[(5R,6S,7S,8aR)-8a-ethenyl-5-hydroxy-6-(3-methoxy-3-oxoprop-1-en-2-yl)-4-methylidene-3-oxo-1,4a,5,6,7,8-hexahydroisochromen-7-yl] 2-	[M+H]+	415.13632	C20H2408	Terpene lactones	FZALQGCQQJLGKE-ZBUWNZDNSA-N	O=C(OC)C(=C)C1C(O)C2C(=C)C(=O)OCC2(C=C)CC1OC(=O)C(=C)CO
1882	6.971	527.11969	[(2S,3R,4S,5S,6R)-4,5-dihydroxy-6-(hydroxymethyl)-2-[2,4,6-trihydroxy-3-(4-hydroxybenzoyl)phenyl]oxan-3-yl] 4-hydroxybenzoate	[M-H]-	527.11951	C26H24012	Phenolic glycosides	CIQDSZUZMUTHGY-FUFTYFEXSA-N	O=C(OC1C(O)C(O)C(OC1C=2C(O)=CC(O)=C(C(=O)C3=CC=C(O)C=C3)C2O)CO)C4=CC=C(O)C=C4
1883	6.973	470.92853	Fumarprotocetraric Acid	[M-H]-	470.92581	C22H16012	Depsides and depsidones	VEGGRTFDFMUBPD-ONEGZZNKSA-N	O=CC1=C(O)C=C(C=2C(=O)OC3=C(OC12)C(=C(C(=O)O)C(O)=C3COC(=O)C=CC(=O)O)C)C
1884	6.973	437.11972	Picropodophyllotoxin	[M+H]+	437.12	C22H2208	Podophyllotoxins	YJGVMLPVUAXIQN-UHFFFAOYSA-N	O=C1OCC2C(O)C3=CC=4OCOC4C=C3C(C5=CC(OC)=C(OC)C(OC)=C5)C12
1885	6.979	851.31927	Microsclerodermin D	[M+H]+	851.31299	C40H49C1N8012	Oligopeptides	VXSZBEKMOUAWID-IQEALIBRSA-N	O=C1N(C)CC(O)=NC(C(O)=NCC(O)=NCC(O)CC(O)=NC(C(O)C(O)=NC2CC(O)=NC2(O)C1)C(O)C(O)CC=CC=3C=CC=CC3)CC4=CNC=5C=C(
1886	7.025	316.17493	PSEUDO-ANISATIN	[M+H]+	316.17545	C15H2206	Lactones	NOHXYGXFTACADH-JHLBFKOCSA-N	O=C1OCC2(C)C(O)(C(=O)CC3(C1)C(C)CC(O)C23O)C
1887	7.043	273.16931	Heteropeucenin, Methyl Ether	[M-H]-	273.16537	C16H1804	Chromones	KVNRDSLYCOJCRD-UHFFFAOYSA-N	O=C1C=C(OC=2C=1C(O)C=C(OC)C2CC=C(C)C)C
1888	7.044	629.27155	2-[3-[(E)-6-acetyloxy-2-hydroxy-6-methyl-3-oxohept-4-en-2-yl]-6-(carboxymethyl)-2-hydroxy-3a,5a,9b-trimethyl-5-oxo-2,3,4,6,9,9a-hexahydro-	[M+2H]2+	629.27222	C32H46010	Triterpenoids	AEGZYCGXUREGSM-OUKQBFOZSA-N	O=C(O)CC1C(=CCC2C1(C(=O)CC3(C)C(C(O)CC23C)C(O)(C(=O)C=CC(OC(=O)C)(C)C)C)C(C(=O)O)(C)C
1889	7.047	431.08652	kaempferol-7-O-deoxyhexoside	[M-H]-	431.09299	C21H20010	Flavonoid-7-O-glycosides	HQNOUCSPWAGQND-UHFFFAOYSA-N	O=C1C(O)=C(OC2=CC(OC3OC(C)C(O)C(O)C3O)=CC(O)=C12)C=4C=CC(O)=CC4
1890	7.048	337.10251	3-p-Coumaroylquinic acid	[M-H]-	337.10001	C16H1808	Quinic acids and derivatives	BMRSEYFENKXDIS-FASCMIHUSA-N	O=C(OC1CC(O)(C(=O)O)CC(O)C1O)C=CC2=CC=C(O)C=C2

1891	7.053	280.14859	Isocycloheximide	[M-H]-	280.14496	C15H23NO4	Piperidinediones	YPHMISFOHDHNIV-UHFFFAOYSA-N	O=C1N=C(O)CC(C1)CC(O)C2C(=O)C(C)CC(C)C2
1892	7.057	180.17378	Rimantadine (Flumadine)	[M+H]+	180.175	C12H21N	Monoalkylamines	UBCHPRBFMUDMNC-UHFFFAOYSA-N	NC(C)C12CC3CC(CC(C3)C1)C2
1893	7.063	436.25153	paxilline	[M+H]+	436.25	C27H33NO4	Naphthopyrans	ACNHBCIZLNNLRS-FAWQAIAASA-N	O=C1C=C2C(OC1C(O)(C)C)CCC3(C)C2(O)CCC4CC=5C=6C=CC=CC6N C5C43C
1894	7.07	706.39783	(2S)-2-[2-[(5R,6R,7S,9S,16R,18S,19S)-19-amino-6-[(3S)-3,4-dicarboxybutanoyl]oxy-16,18-dihydroxy-5,9-dimethylicosan-7-yl]oxy-2-	[M+H]+	706.40082	C34H59NO14	Fumonisins	UXDPXZQHTDAXOZ-CYCMICDFSA-N	O=C(O)CC(C(=O)O)CC(=O)OC(CC(C)CCCCCCC(O)CC(O)C(N)C)C(OC(=O)CC(C(=O)O)CC(=O)O)C(C)CCCC
1895	7.074	273.18375	Galaxolidone	[M+H]+	273.18491	C18H24O2	2-benzopyrans	PGMHPYRIXBRRQD-UHFFFAOYNA-N	CC1C(C)(C)C2=C(C=C3C(=O)OCC(C)C3=C2)C1(C)C
1896	7.075	477.13519	Flavone base + 2O, 2MeO, C-Hex	[M+H]+	477.13708	C23H24O11	Flavone C-glycosides	JCIFZANQIXZLGH-UHFFFAOYNA-N	O=C1C=C(OC=2C=C(OC)C(=C(O)C1=2)C3OC(CO)C(O)C(O)C3(O))C=4C=CC(O)=C(OC)C=4
1897	7.077	419.22565	(E)-3-(4-methoxyphenyl)-1-[2,4,6-trimethoxy-3-(3-methylbut-2-enyl)phenyl]prop-2-en-1-one	[M+H]+	419.22476	C24H28O5	3-prenylated chalcones	GGNVSANPVOEGII-GXDHUFHOSA-N	O=C(C=CC1=CC=C(OC)C=C1)C2=C(OC)C=C(OC)C(=C2OC)CC=C(C)C
1898	7.08	271.01752	Canarione	[M-H]-	271.01801	C14H8O6	Naphthopyranones	NPUXQAACUNUHIX-UHFFFAOYSA-N	O=C1C=C(O)C=2C(O)=C3C(=O)C=C(OC3=CC2C1=O)C
1899	7.104	275.13879	Indole-3-acetyl-L-valine	[M+H]+	275.13901	C15H18N2O3	Amino acids	AZEGJHGXTSUPPG-UHFFFAOYNA-N	CC(C)C(NC(=O)CC1=CNC2=CC=CC=C12)C(O)=O
1900	7.106	316.28351	Dehydrophytosphingosine (not validated, isomer of 1679)	[M+H]+	316.28461	C18H37NO3	Lipids	CQKNELOTFUSOTP-UHFFFAOYNA-N	OCC(N)C(O)C(O)CCCC=CCCCCCCCCC
1901	7.108	193.06279	9-Phenanthrenol	[M-H]-	193.06589	C14H10O	Phenanthrols	DZKIUEHLEXLYKM-UHFFFAOYSA-N	OC1=CC=2C=CC=CC2C=3C=CC=CC13
1902	7.113	336.0748	Famotidine	[M-H]-	336.07318	C8H15N7O2S3	2,4-disubstituted thiazoles	XUFQPHANEAPEMJ-UHFFFAOYSA-N	O=S(=O)(N)NC(=N)CCSCC=1N=C(SC1)NC(=N)N
1903	7.115	577.22717	5,7-dihydroxy-2-(4-hydroxyphenyl)-6-[(2S,3R,4R,5S,6R)-3,4,5-trihydroxy-6-(hydroxymethyl)oxan-2-yl]-8-[(2S,3R,4R,5R,6S)-3,4,5-trihydroxy-6-	[M-H]-	577.22791	C27H30O14	Flavonoid 8-C-glycosides	MVOUGOXRXQDXDC-RSPRXDBDSA-N	O=C1C=C(OC2=C1C(O)=C(C(O)=C2C3OC(C)C(O)C(O)C3O)C4OC(CO)C(O)C(O)C4O)C=5C=CC(O)=CC5
1904	7.118	305.12875	(3S)-8-hydroxy-3-methyl-3,4-dihydro-2H-benzo[a]anthracene-1,7,12-trione	[M-H]-	305.12527	C19H14O4	Angucyclines	ZAWXOCUFQSQDJS-VIFPVBQESA-N	O=C1C2=CC=C3C(C(=O)CC(C)C3)=C2C(=O)C=4C=CC=C(O)C14
1905	7.125	581.27362	2-[[7-hydroxy-1-(4-hydroxy-3,5-dimethoxyphenyl)-3-(hydroxymethyl)-6,8-dimethoxy-1,2,3,4-tetrahydronaphthalen-2-yl]methoxy]-6-	[M-H]-	581.26746	C28H38O13	Lignan glycosides	PQQRNPDHSJDAGV-UHFFFAOYSA-N	OC1=C(OC)C=C(C=C1OC)C2C3=C(OC)C(O)=C(OC)C=C3CC(CO)C2COC4OC(CO)C(O)C(O)C4O
1906	7.127	301.19992	1-(2,4-dihydroxyphenyl)-2-(3,5-dimethoxyphenyl)propan-1-one	[M-H]-	301.19559	C17H18O5	Alpha-methyldeoxybenzoin flavonoids	TURHNAJJDDIVGC-UHFFFAOYSA-N	O=C(C1=CC=C(O)C=C1O)C(C=2C=C(OC)C=C(OC)C2)C
1907	7.133	957.505	[(2S,3R,4S,5S,6R)-6-[[(2R,3R,4R,5S,6R)-3,4-dihydroxy-6-(hydroxymethyl)-5-[(2S,3R,4R,5R,6S)-3,4,5-trihydroxy-6-methyloxan-2-yl]oxyoxan-2-	[M-H]-	957.50641	C48H78O19	Triterpene saponins	WYQVAPGDARQUBT-ZKFJACKJSA-N	O=C(OC1OC(COC2OC(CO)C(OC3OC(C)C(O)C(O)C3O)C(O)C2O)C(O)C(O)C1O)C45CCC(C)C(C)C5C6=CCC7C8(C)CC(O)C(O)C(C)(CO)C8
1908	7.141	497.10834	9-methoxy-7-[4-[(2S,3R,4S,5S,6R)-3,4,5-trihydroxy-6-(hydroxymethyl)oxan-2-yl]oxyphenyl]-[1,3]dioxolo[4,5-g]chromen-8-one	[M+H]+	497.10544	C23H22O11	Isoflavonoid O-glycosides	MLRDPISQDDYRHC-BSTKLLGTSA-N	O=C1C(=COC=2C=C3OCOC3=C(OC)C12)C=4C=CC(OC5OC(CO)C(O)C(O)C5O)=CC4
1909	7.205	171.13734	delta-Decalactone	[M+H]+	171.13795	C10H18O2	Delta valerolactones	GHBSPIPJMLAMEP-UHFFFAOYNA-N	CCCCCC1CCCC(=O)O1
1910	7.209	491.11758	Malvidin-3-O-glucoside	[M-H]-	491.11896	C23H25O12	Anthocyanidin O-glycosides	PXUQTDZNOHRWLI-UHFFFAOYNA-O	COC1=CC(=CC(OC)=C1O)C1=[O+]C2=CC(O)=CC(O)=C2C=C1OC1OC(CO)C(O)C(O)C1O
1911	7.214	405.24692	(E)-10,10-dimethyl-10a-(3-(pentyloxy)styryl)-3,4,10,10a-tetrahydropyrimido[1,2-a]indol-2-ol	[M+H]+	405.25	C26H32N2O2	Indoles and derivatives	IEFRVMKLIFGNMU-JQIJEIRASA-N	OC1=NC2(C=CC=3C=CC=C(OCCCCC)C3)N(C=4C=CC=CC4C2(C)C)CC1
1912	7.222	351.21356	7-beta-Hydroxylathyrol	[M+H]+	351.20999	C20H30O5	Diterpenoids	NVDZRSWQWPPKAI-TWGQIWQCSA-N	O=C1C(=CC2C(CC(O)C(=C)C(O)C3C(O)C(C)CC13O)C2(C)C)C
1913	7.224	429.11575	7-[3,4-dihydroxy-5-(hydroxymethyl)oxolan-2-yl]oxy-2-(4-hydroxyphenyl)-3,4-dihydro-2H-chromene-3,5-diol	[M+H]+	429.1156	C20H22O9	Flavonoid-7-O-glycosides	XWFQRXGYJJOFCO-UHFFFAOYSA-N	OC1=CC=C(C=C1)C2OC3=CC(OC4OC(CO)C(O)C4O)=CC(O)=C3CC2O
1914	7.226	533.30847	3-[(3S,5R,10S,13R,14S,17S)-3-[(2R,3S,4R,5S,6S)-3,5-dihydroxy-4-methoxy-6-methyloxan-2-yl]oxy-14-hydroxy-10,13-dimethyl-	[M-H]-	533.31195	C30H46O8	Cardenolide glycosides and derivatives	VPUNMTHWNSJUOG-JGFVFIPGSA-N	O=C1OCC(=C1)C2CCC3(O)C4CCC5CC(OC6OC(C)C(O)C(OC)C6O)CCC5(C)C4CCC23C
1915	7.248	523.2594	[(3S,4R,5S)-5-[(2S,3R,4S,5S,6R)-4,5-dihydroxy-6-(hydroxymethyl)-2-(4-hydroxyphenoxy)oxan-3-yl]oxy-3,4-dihydroxyoxolan-3-yl]methyl 4-	[M-H]-	523.26324	C24H28O13	Phenolic glycosides	GDCHGVOUTCXBSI-JALRXJJFSA-N	O=C(OCC1(O)COC(OC2C(O)C(O)C(OC2OC3=CC=C(O)C=C3)CO)C1O)C4=CC=C(O)C=C4
1916	7.253	639.3717	Furostane base -2H + O-Hex	[M-H]-	639.37299	C33H54O9	Steroidal saponins	DFVRTHJUFCVHTR-UHFFFAOYNA-N	OCC6OC(OCC(C)CCC4(O)(OC3CC2C5CC=C1CC(O)CCC1(C)C5(CCC2(C)C3C4(C))))C(O)C(O)C6(O)
1917	7.263	298.16418	2-hydroxy-4-(hydroxymethyl)-6-(1-hydroxy-3-methylbut-2-enyl)-3-[(E)-prop-1-enyl]-7-oxabicyclo[4.1.0]hept-3-en-5-one	[M+H]+	298.16489	C15H20O5	Cyclohexenones	FSYVGCMCWALMCX-SNAWJCMRSA-N	O=C1C(=C(C=CC)C(O)C2OC12C(O)C=C(C)C)CO
1918	7.271	282.11319	methyl 2-benzamido-3-phenylpropanoate	[M-H]-	282.11356	C17H17NO3	Phenylalanine and derivatives	BRQQPHKRRCUYLA-UHFFFAOYSA-N	O=C(OC)C(N=C(O)C=1C=CC=CC1)CC=2C=CC=CC2
1919	7.282	649.39435	[(2S,3R,4S,5S,6R)-3,4,5-trihydroxy-6-(hydroxymethyl)oxan-2-yl] (1R,2R,4aS,6aS,6bR,10R,11R,12aR)-1,10,11-trihydroxy-1,2,6a,6b,9,9,12a-	[M-H]-	649.39569	C36H58O10	Triterpene saponins	MLKQAGPAYHTNQQ-UJKXLRNUSA-N	O=C(OC1OC(CO)C(O)C(O)C1O)C23CCC(C)C(O)(C)C3C4=CCC5C6(C)CC(O)C(O)C(C)(C)C6CCC5(C)C4(C)CC2
1920	7.285	284.09009	skimmianine	[M+H]+	284.09	C14H15NO4	Dihydrofuranoquinolines	CKDKGHDDRAMHGK-UHFFFAOYSA-N	N=1C=2OCCC2C(OC)=C3C=CC(OC)=C(OC)C13
1921	7.291	583.20343	Naringin dihydrochalcone	[M+H]+	583.20001	C27H34O14	Flavonoid O-glycosides	CWBZAESOUBENAP-QVNVHUMTSA-N	O=C(C1=C(O)C=C(OC2OC(CO)C(O)C(O)C2OC3OC(C)C(O)C(O)C3O)C=C1O)CCC4=CC=C(O)C=C4
1922	7.294	389.16071	9R,10R)-10-acetoxy-8,8-dimethyl-2-oxo-2,8,9,10-tetrahydropyrano[2,3-f]chromen-9-yl 3-methylbutanoate	[M+H]+	389.16	C21H24O7	Angular pyranocoumarins	ALKTVPFKDYZFGA-WOJBJXKFSA-N	O=C1OC2=C(C=C1)C=CC=3OC(C)(C)C(OC(=O)CC(C)C)C(OC(=O)C)C32
1923	7.295	305.08936	1,4-dihydroxy-6,6,9a-trimethyl-4,5,5a,7,8,9-hexahydro-1H-benzo[e][2]benzofuran-3-one	[M+H]+	305.08911	C15H22O4	Naphthofurans	FFDNVMGPKVVVOG-UHFFFAOYSA-N	O=C1OC(O)C2=C1C(O)CC3C2(C)CCCC3(C)C
1924	7.297	605.18787	methyl (4S,5Z,6S)-5-(2-acetyloxyethylidene)-4-[2-[2-(4-hydroxyphenyl)ethoxy]-2-oxoethyl]-6-[(2S,3R,4S,5S,6R)-3,4,5-trihydroxy-6-	[M+H]+	605.18408	C27H34O14	Terpene glycosides	DKRXODJAISNRGA-KIOYJQFDSA-N	O=C(OC)C1=COC(OC2OC(CO)C(O)C(O)C2O)C(=CCOC(=O)C)C1CC(=O)OCCC3=CC=C(O)C=C3
1925	7.298	348.11127	Adenosine Monophosphate	[M+H]+	348.11115	C10H14N5O7P	Purine ribonucleoside monophosphates	UDMBCSSLTHHNCD-KQYNXXCUSA-N	O=P(O)(O)OCC1OC(N2C=NC=3C(=NC=NC32)N)C(O)C1O
1926	7.299	515.33972	arthrobactin	[M+2H]2+	515.33728	C20H36N4O9	Hydroxy fatty acids	BPEXJHGGARTCIR-UHFFFAOYSA-N	O=C(O)C(O)(CC(O)=NCCCCCN(O)C(=O)C)CC(O)=NCCCCCN(O)C(=O)C
1927	7.3	497.30975	(5R)-5-[(1S,2S,3R,4R)-3-[(4E)-hex-4-enoyl]-6-[(2E,4E)-hexa-2,4-dienoyl]-5,7-dihydroxy-4,7-dimethyl-8-oxobicyclo[2.2.2]oct-5-en-2-yl]-4-hydroxy-	[M-H]-	497.30447	C28H34O8	Cyclohexenones	CXIHBIKIPKUWBP-XPIMNQETSA-N	O=C1OC(C(O)=C1C)(C)C2C(C(=O)CCC=CC)C3(C(=O)C(O)(C)C2C(C(=O)C=CC=CC)=C3O)C
1928	7.3	407.17282	[5-chloro-3-[(1E,3E)-3,5-dimethylhepta-1,3-dienyl]-7-methyl-6,8-dioxo-2H-isoquinolin-7-yl] acetate	[M+H]+	407.17319	C21H24ClNO4	Azaphilones	DOUKEKLEUAGITQ-NJHPPEEMSA-N	O=C(OC1(C(=O)C(Cl)=C2C=C(C=CC(=CC(C)CC)C)NC=C2C1=O)C)C
1929	7.301	307.0856	(-)-Epigallocatechin (EGC)	[M+H]+	307.082	C15H14O7	Epigallocatechins	XMOCLSLCDHWDHP-IUODEOHRSA-N	OC=1C=C(O)C2=C(OC(C3=CC(O)=C(O)C(O)=C3)C(O)C2)C1
1930	7.301	177.1266	geraniol	[M+H]+	177.13	C10H18O	Acyclic monoterpenoids	GLZPCOQZEFWAFX-JXMROGBWSA-N	OCC=C(C)CCC=C(C)C
1931	7.302	350.21988	(3R,5S,17R)-3-hydroxy-10,13-dimethylhexadecahydro-1H-cyclopenta[a]phenanthren-17-aminium chloride	[M+2H]2+	350.22	C19H34ClNO	Androgens and derivatives	QUJWGPKTCDNZHS-YHGFUEDJSA-N	[Cl-].OC1CCC2(C)C(CCC3C4CCC([NH3+])C4(C)CCC32)C1
1932	7.303	236.09065	1-{4-hydroxy-2-oxo-6-[(1E)-prop-1-en-1-yl]-2H-pyran-3-yl}butane-1,2-dione	[M+H]+	236.09027	C12H12O5	Aryl ketones	CEVGSSZDDUFFQW-HWKANZROSA-N	O=C1OC(C=CC)=CC(O)=C1C(=O)C(=O)CC
1933	7.303	353.22754	Anileridine	[M+H]+	353.22235	C22H28N2O2	Phenylpiperidines	LKYQLAWMNBFNJT-UHFFFAOYSA-N	CCOC(=O)C1(CCN(CCC2=CC=C(N)C=C2)CC1)C1=CC=CC=C1

1934	7.306	329.23157	FA 18:1+3O	[M-H]-	329.2312	C18H34O5	Oxidized fatty acids	MDIUMSLCYIJBQC-UHFFFAOYNA-N	O=C(O)CCCCCCCC(O)C=CC(O)C(O)CCCCC
1935	7.31	385.1658	Schisandrin C	[M+H]+	385.16501	C22H24O6	Hydrolyzable tannins	HTBWBWWADZJXID-TXEJJXNPSA-N	O(C1=C2OCOC2=CC3=C1C4=C(OC)C=5OCOC5C=C4CC(C)C(C)C3)C
1936	7.337	459.18942	(2R,3S,4S,5R,6R)-2-[[(2R,3R,4R)-3,4-dihydroxy-5-(hydroxymethyl)oxolan-2-yl]oxymethyl]-6-[4-(4-hydroxyphenyl)butan-2-yloxy]oxane-3,4,5-triol	[M-H]-	459.18716	C21H32O11	Fatty acyl glycosides of mono- and disaccharides	DJDISCKFJXDADJ-COUYJTLKSA-N	OC1=CC=C(C=C1)CCC(OC2OC(COC3OC(CO)C(O)C3O)C(O)C(O)C2O)C
1937	7.346	315.19052	14-(hydroxymethyl)-5,9-dimethyltetracyclo[11.2.1.0?,??.0?,?]hexadecan-5-ol	[M+H]+	315.19052	C19H32O2	Kaurane diterpenoids	LZAZGDZVFQSCFO-UHFFFAOYSA-N	OCC1CC23CCC4C(O)(C)CCCC4(C)C3CCC1C2
1938	7.346	476.27585	LPE 18:2	[M-H]-	476.27609	C23H44NO7P	Lipids	DBHKHNGBVGWQJE-UHFFFAOYNA-N	O=C(OCC(O)COP(=O)(O)OCCN)CCCCCCCC=CCC=CCCCCC
1939	7.349	809.24084	Echinacoside	[M+H]+	809.23999	C35H46O20	Oligosaccharides	FSBUXLDOLNLABB-ISAKITKMSA-N	O=C(OC1C(OC(OCCC2=CC=C(O)C(O)=C2)C(O)C1OC3OC(C)C(O)C(O)C3O)COC4OC(CO)C(O)C(O)C4O)C=CC5=CC=C(O)C(O)=C5
1940	7.357	383.29346	Geranylgeranyl 1,4-hydroquinone	[M+H]+	383.29199	C26H38O2	Diterpenoids	FIPAQYYZFDCRDD-FPFQZNTGSA-N	OC1=CC=C(O)C(=C1)CC=C(C)CCC=C(C)CCC=C(C)CCC=C(C)C
1941	7.362	611.20062	Dihydrohesperetin-7-O-neohesperidoside	[M-H]-	611.19812	C28H36O15	Flavonoid O-glycosides	ITVGXXMINPYUHD-UHFFFAOYNA-N	COC1=C(O)C=C(CCC(=O)C2=C(O)C=C(OC3OC(CO)C(O)C(O)C3OC3OC(C)C(O)C(O)C3O)C=C2O)C=C1
1942	7.362	635.19659	Neohesperidin Dihydrochalcone	[M+H]+	635.19	C28H36O15	Flavonoid O-glycosides	ITVGXXMINPYUHD-CUVHLRMHSA-N	O=C(C1=C(O)C=C(OC2OC(CO)C(O)C(O)C2OC3OC(C)C(O)C(O)C3O)C=C1O)CCC4=CC=C(OC)C(O)=C4
1943	7.37	285.06439	Indigo Carmine	[M+H]+	285.06345	C16H10N2O2	Indolines	COHYTHOBJLSHDF-BUHFOSPRSA-N	O=C1C=2C=CC=CC2NC1=C3NC=4C=CC=CC4C3=O
1944	7.37	593.24634	Pantethine	[M+H]+	593.24792	C22H42N4O8S2	Beta amino acids and derivatives	DJWYOLJPSHDSAL-UHFFFAOYSA-N	OC(=NCCSSCCN=C(O)CCN=C(O)C(O)C(C)(C)CO)CCN=C(O)C(O)C(C)(C)CO
1945	7.371	527.20685	13-(acetyloxy)-1,6-dihydroxy-8-(hydroxymethyl)-4,12,12,15-tetramethyl-5-oxotetracyclo[8.5.0.0?,?.0??,??]pentadeca-3,8-dien-14-yl (2E)-2-methylbut-	[M+2H]2+	527.20416	C27H36O8	Phorbol esters	PCASZNXKLGTEGB-MDWZMJQESA-N	O=C(OC1C(C)C2(O)C(C=C(CO)CC3(O)C(=O)C(=CC32)C)C4C1(OC(=O)C)C4(C)C)C(=CC)C
1946	7.375	401.30441	5-[5-(acetyloxymethyl)-1,2,4a-trimethyl-7-oxo-3,4,8,8a-tetrahydro-2H-naphthalen-1-yl]-3-methylpentanoic acid	[M+H]+	401.30591	C22H34O5	Colensane and clerodane diterpenoids	OLDFOHABNQZMKB-UHFFFAOYSA-N	O=C(OCC1=CC(=O)CC2C1(C)CCC(C)C2(C)CCC(C)CC(=O)O)C
1947	7.383	314.26846	Dehydrophytosphingosine (not validated) - 2H	[M+H]+	314.2691	C18H35NO3	Lipids	WWXZQFWOWANITM-AQASXUMVNA-N	CCCCCCCCC\C=C\C=C\CC(O)C(O)C(N)CO
1948	7.384	357.22641	2-(2-hydroxy-6-methylhept-5-en-2-yl)-7-(3-methylbut-2-enyl)-2,3-dihydro-1-benzofuran-5-carboxylic acid	[M-H]-	357.22589	C22H30O4	Coumarans	GZLIPAFSJXROEC-UHFFFAOYSA-N	O=C(O)C=1C=C(C=2OC(CC2C1)C(O)(C)CCC=C(C)C)CC=C(C)C
1949	7.386	563.18872	[4a,7-dihydroxy-7-methyl-1-[3,4,5-trihydroxy-6-(hydroxymethyl)oxan-2-yl]oxy-1,5,6,7a-tetrahydrocyclopenta[c]pyran-5-yl] (E)-3-(4-	[M+H]+	563.18829	C25H32O12	Iridoid O-glycosides	LGFYNKGLEKBLDA-VMPITWQZSA-N	O=C(OC1CC(O)(C)C2C(OC=CC12)OC3OC(CO)C(O)C(O)C3O)C=CC4=CC=C(OC)C=C4
1950	7.387	626.2533	(S)-4-((7-acetamido-1,2,3-trimethoxy-9-oxo-5,6,7,9-tetrahydrobenzo[a]heptalen-10-yl)amino)-N-(benzo[d][1,3]dioxol-5-	[M+H]+	626.25	C33H37N3O8	Gamma amino acids and derivatives	PQOCSSYRLUIAGG-DEOSSOPVSA-N	O=C1C=C2C(=CC=C1NCCCC(=O)NCC3=CC=C4OCOC4=C3)C5=C(OC)C(OC)=C(OC)C=C5CCC2NC(=O)C
1951	7.392	328.1402	1-O-trans-cinnamoyl-beta-D-glucopyranose	[M+2H]2+	328.13907	C15H18O7	O-cinnamoyl glycosides	CJGRGYBLAHPYOM-HOLMNUNMSA-N	O=C(OC1OC(CO)C(O)C(O)C1O)C=CC=2C=CC=CC2
1952	7.399	351.25076	1,2-dihydroxyheptadec-16-en-4-yl acetate	[M+H]+	351.25055	C19H36O4	Long-chain fatty alcohols	MFLWBVVCOWPUBA-UHFFFAOYSA-N	O=C(OC(CCCCCCCCCCCC=C)CC(O)CO)C
1953	7.406	823.44666	(3?,8?,9?,24E)-3-[(6-O-Acetyl-4-O-pentopyranosylhexopyranosyl)oxy]-12,16-dihydroxy-9,19-cyclolanost-24-en-26-oic acid	[M-H]-	823.44849	C43H68O15	Cucurbitacin glycosides	IRXIEUDBDWXTTL-NKVVMBDVSA-N	O=C(O)C(=CCCC(C)C1C(O)CC2(C)C3CCC4C(C)(C)C(OC5OC(COC(=O)C)C(OC6OCC(O)C(O)C6O)C(O)C5O)CCC74CC37CC(O)C12C)C
1954	7.415	499.31714	methyl (1S,5S,9S,10R,15R)-15-(acetyloxy)-8-hydroxy-4,5,7,10,14,14-hexamethyl-6,18-dioxo-19-oxapentacyclo[10.5.2.0?,??.0?,??.0?,?]nonadeca-	[M-H]-	499.32227	C28H36O8	Steroid esters	JYWRCSAWBUAIFO-KRRNMHNDSA-N	O=C(OC1CCC23C(=O)OC(CC4(C)C2C=C(C)C5(C(=O)C(=C(O)C54C(=O)OC)C)C)C3C1(C)C)C
1955	7.425	356.24191	Pesticide4_Piperonyl butoxide_C19H30O5_Butacide	[M+H]+	356.24301	C19H30O5	Benzodioxoles	FIPWRIJSWJWJAI-UHFFFAOYSA-N	O1C=2C=C(C(=CC2OC1)CCC)COCCOCCOCCCC
1956	7.43	388.19675	Alfuzosin	[M-H]-	388.19904	C19H27N5O4	Quinazolinamines	WNMJYKCGWZFFKR-UHFFFAOYNA-N	N=C1NC(=NC=2C=C(OC)C(OC)=CC12)N(C)CCCN=C(O)C3OCCC3
1957	7.439	459.09171	oroxindin	[M-H]-	459.09329	C22H20O11	Flavonoid-7-O-glucuronides	LNOHXHDWGCMVCO-NTKSAMNMSA-N	O=C(O)C1OC(OC2=CC(O)=C3C(=O)C=C(OC3=C2OC)C=4C=CC=CC4)C(O)C(O)C1O
1958	7.441	500.37161	(2aS,6aS,8aS)-10-(4-acetamido-3-methylbutyl)-6a,8a,9-trimethyl-2,2a,3,4,5,6,6a,6b,7,8,8a,8b,11a,12,12a,12b-hexadecahydro-1H-	[M+H]+	500.37	C31H49NO4	Furostanes and derivatives	NNRLQJLRICHBII-FWVIVEMUSA-N	O=C(OC1CCC2(C)C(CCC3C2CCC4(C)C5C(=C(OC5CC34)CCC(C)CNC(=O)C)C)C1)C
1959	7.445	256.09549	Toradol	[M+H]+	256.09683	C15H13NO3	Aryl-phenylketones	OZWKMVRBQXNZKK-UHFFFAOYNA-N	O=C(O)C1C2=CC=C(C(=O)C=3C=CC=CC3)N2CC1
1960	7.446	287.20041	Androstenedione	[M+H]+	287.20056	C19H26O2	Androgens and derivatives	AEMFNILZOJDQLW-UHFFFAOYNA-N	O=C1C=C2CCC3C4CCC(=O)C4(C)CCC3C2(C)CC1
1961	7.448	303.19485	2-Methoxyestradiol	[M+H]+	303.19501	C19H26O3	Estrogens and derivatives	CQOQDQWUFQDJMK-ZGPDXJMCSA-N	OC=1C=C2C(=CC1OC)C3CCC4(C)C(O)CCC4C3CC2
1962	7.449	299.09183	(2Z)-4,6-dihydroxy-2-[(3-hydroxy-4-methoxyphenyl)methylidene]-1-benzofuran-3-one	[M-H]-	299.09195	C16H12O6	Aurone flavonoids	WKEOZXMSVNVPCM-RZNTYIFUSA-N	O=C1C(OC=2C=C(O)C=C(O)C12)=CC3=CC=C(OC)C(O)=C3
1963	7.451	137.13232	myrcene	[M+H]+	137.13	C10H16	Acyclic monoterpenoids	UAHWPYUMFXYFJY-UHFFFAOYSA-N	C=CC(=C)CCC=C(C)C
1964	7.452	483.23553	(1R,3E,5R,7S,11R,12R,13S,14S)-1,13-bis(acetyloxy)-3,6,6,14-tetramethyl-10-methylidene-2-oxotricyclo[10.3.0.0?,?]pentadec-3-en-11-yl acetate	[M+H]+	483.23532	C26H36O7	Diterpenoids	WFMPZQDIRPRCNA-PNWNLVJFSA-N	O=C(OC1C(=C)CCC2C(C=C(C(=O)C3(OC(=O)C)CC(C)C(OC(=O)C)C13)C)C2(C)C)C
1965	7.452	270.17044	4-oxo-4-(3-oxodecan-2-ylamino)butanoic acid	[M-H]-	270.17108	C14H25NO4	N-acyl amines	DGBNCMKQNOJCFB-UHFFFAOYSA-N	O=C(O)CCC(O)=NC(C(=O)CCCCCCC)C
1966	7.459	719.30804	Ritonavir	[M-H]-	719.30548	C37H48N6O5S2	N-carbamoyl-alpha amino acids and derivatives	NCDNCNXCDXHOMX-UHFFFAOYNA-N	OC(=NC(CC=1C=CC=CC1)C(O)CC(N=C(O)C(N=C(O)N(C)CC=2N=C(SC2)C(C)C)C(C)C)CC=3C=CC=CC3)OCC=4SC=NC4
1967	7.466	619.38342	(2E)-6-{13,17-dihydroxy-7,7,12,16-tetramethyl-6-[(3,4,5-trihydroxyoxan-2-yl)oxy]pentacyclo[9.7.0.0?,?.0?,?.0??,??]octadecan-15-yl}-2-methylhept-2-	[M-H]-	619.38513	C35H56O9	Cucurbitacin glycosides	LIFSGPPJRHEFEY-DJKKODMXSA-N	O=C(O)C(=CCCC(C)C1CC(O)C2(C)C3CCC4C(C)(C)C(OC5OCC(O)C(O)C5O)CCC64CC36CC(O)C12C)C
1968	7.467	413.21765	purpactin A	[M-H]-	413.21661	C23H26O7	Diarylethers	NUYFKDBCHFKOBT-UHFFFAOYSA-N	O=C1OCC2=CC(=CC(O)=C2OC3=CC=C(C(OC)=C13)C(OC(=O)C)CC(C)C)C
1969	7.48	432.22995	11,12-dihydroxy-13-methoxy-4,5,8-trimethyl-3-(2-methylpropyl)-1H,2H,3H,4H,6aH,9H,10H,11H,12H,13H,14H,15H,15bH-cycloundeca[e]isoindole-	[M-H]-	432.22992	C25H39NO5	Aspochalasins	VMCFTVWDHVWSOB-UHFFFAOYSA-N	O=C1CC(OC)C(O)C(O)CCC(=CC2C=C(C)C(C)C3C(N=C(O)C123)CC(C)C)C
1970	7.485	587.1543	[(2R,3S,4S,5R,6S)-6-[4-[(E)-3-(2,4-dihydroxyphenyl)-3-oxoprop-1-enyl]phenoxy]-3,4,5-trihydroxyoxan-2-yl]methyl (E)-3-(4-	[M+H]+	587.15234	C30H28O11	Flavonoid O-glycosides	FCYCVETXTGWXBR-CMUGPJGNSA-N	O=C(OCC1OC(OC2=CC=C(C=CC(=O)C3=CC=C(O)C=C3O)C=C2)C(O)C(O)C1O)C=CC4=CC=C(O)C=C4
1971	7.486	795.45044	Soyasapogenol B base + O-HexA-Hex	[M-H]-	795.45752	C42H68O14	Triterpene saponins	OKIHRVKXRCAJFQ-UHFFFAOYNA-N	O=C(O)C7OC(OC2CCC3(C)(C4CC=C1C5CC(C)(C)CC(O)C5(C)(CCC1(C)C4(C)(CCC3(C2(C)(CO))))))C(OC6OC(CO)C(O)C(O)C6(O))C
1972	7.494	659.28058	Fucoxanthin	[M+2H]2+	659.27808	C42H58O6	Xanthophylls	SJWWTRQNNRNTPU-ABBNZJFMSA-N	O=C(OC1CC(O)(C(=C=CC(=CC=CC(=CC=CC=C(C=CC=C(C(=O)CC23OC3(C)CC(O)CC2(C)C)C)C)C)C)C(C)(C)C1)C)C
1973	7.541	349.98532	PFAP-N_PFOSE	[M-H]-	349.9892	C6H11F5NO6PS	PFSA	BGGDXJKUDZPPBR-UHFFFAOYSA-N	O=P(O)(O)OCCN(CC)S(=O)(=O)C(F)(F)C(F)(F)F
1974	7.549	811.44879	3-Glu-28-Glu-Bayogenin (NMR)	[M-H]-	811.44855	C42H68O15	Triterpene saponins	ZUILGDNVKPMVIA-YRTOFAGUSA-N	CC1(C)CC[C=]2(CC[C=]3(C)C(=CCC4[C=]5(C)C[C=H](O)[C=H](O[C=H]6O[C=H](CO)[C=H](O)[C=H](O)[C=H]6O)[C=](C)(CO)C5
1975	7.551	169.01572	Gallic acid	[M-H]-	169.01425	C7H6O5	Phenolic glycosides	LNTHITQWFMADLM-UHFFFAOYSA-N	OC(=O)C1=CC(O)=C(O)C(O)=C1
1976	7.561	222.11174	Pesticide3_Carbofuran_C12H15NO3_Furadan	[M+H]+	222.112	C12H15NO3	Coumarans	DUEPRVBVGDRKAG-UHFFFAOYSA-N	OC(=NC)OC1=CC=CC2=C1OC(C)(C)C2

1977	7.623	304.26459	Fenpropimorph	[M+H]+	304.26349	C20H33NO	Phenylpropanes	RYAUSSKQMZRMAI-UHFFFAOYNA-N	CC(CC1=CC=C(C=C1)C(C)(C)C)CN2CC(C)OC(C)C2
1978	7.624	390.28265	Roquefortine C	[M+H]+	390.27811	C22H23N5O2	Pyrroloindoles	SPWSUFUPTSJWNG-LIMLNZRCSA-N	O=C1C(N=C(O)C2N1C3NC=4C=CC=CC4C3(C2)C(C=C)(C)C)=CC5=CN=CN5
1979	7.65	284.29617	1-desoxymethylsphinganine	[M+H]+	284.29401	C17H37NO	1,2-aminoalcohols	UGVBFHUWZNNKIK-QGZVFWFLSA-N	OC(CN)CCCCCCCCCCCCCCC
1980	7.662	172.16907	Chrysanthemyl Alcohol	[M+H]+	172.16959	C10H18O	Monocyclic monoterpenoids	HIPIENNKVJCMAP-DTWKUNHWSA-N	OCC1C(C=C(C)C)C1(C)C
1981	7.664	599.17377	4-[3,4,5-trihydroxy-6-(hydroxymethyl)oxan-2-yl]oxy-9-(3,4,5-trimethoxyphenyl)-5a,6,8a,9-tetrahydro-5H-[2]benzofuro[6,5-	[M+H]+	599.17352	C28H32O13	Lignan glycosides	PAIASCMUTMHGHU-UHFFFAOYSA-N	O=C1OCC2CC3=C(OC4OC(CO)C(O)C(O)C4O)C=5OCOC5C=C3C(C6=CC(OC)=C(OC)C(OC)=C6)C12
1982	7.668	199.12221	Tacrine	[M+H]+	199.12297	C13H14N2	Acridines	YLJREFDVOIBQDA-UHFFFAOYSA-N	N=C1C=2C=CC=CC2NC3=C1CCCC3
1983	7.669	254.17445	MPL-dm	[M+H]+	254.17506	C14H23NO3	Tyrosols and derivatives	CUKXSBOAIJILRY-UHFFFAOYNA-N	CC(C)NCC(COC1=CC=C(C=C1)CCO)O
1984	7.679	292.18994	(4aS)-6,7-dihydroxy-1,1,4a-trimethyl-3,4,10,10a-tetrahydro-2H-phenanthren-9-one	[M+H]+	292.1907	C17H22O3	Diterpenoids	JMBKXUYCBVKSSY-OMOCHNIRSA-N	O=C1C2=CC(O)=C(O)C=C2C3(C)CCCC(C)(C)C3C1
1985	7.684	449.22769	Irigenin, 7-Benzyl Ether	[M-H]-	449.22253	C25H22O8	3'-hydroxy,4'-methoxyisoflavonoids	WSKWDXWDFFVUKX-UHFFFAOYSA-N	O=C1C(=COC=2C=C(OCC=3C=CC=CC3)C(OC)=C(O)C12)C=4C=C(O)C(OC)=C(OC)C4
1986	7.684	957.50177	Soyasaponin Ba	[M-H]-	957.50647	C48H78O19	Triterpene saponins	WFRQIKSNAYYUJZ-UHFFFAOYNA-N	CC1(C)CC(O)C2(C)CCC3(C)C(=CCC4C5(C)CCC(OC6OC(C(O)C(O)C6OC6OC(CO)C(O)C(O)C6OC6OC(CO)C(O)C(O)C6O)C(O)=O)C(C)(C
1987	7.69	433.09933	Reynoutrin	[M-H]-	433.10001	C20H18O11	Flavonoid-3-O-glycosides	PZZRDJXEMZMZFD-ODPGBAFUSA-N	O=C1C(OC2OCC(O)C(O)C2O)=C(OC=3C=C(O)C=C(O)C13)C=4C=CC(O)=C(O)C4
1988	7.695	389.20721	3-[3-[(2E)-3,7-dimethylocta-2,6-dienyl]-4-hydroxyphenyl]-7-hydroxychromen-4-one	[M-H]-	389.20807	C25H26O4	Isoflavones	ZBHUUXLHDOUMKM-REZTVBANSA-N	O=C1C(=COC2=CC(O)=CC=C12)C=3C=CC(O)=C(C3)CC=C(C)CCC=C(C)C
1989	7.706	281.14209	4-(2,6-dihydroxy-6-methylheptan-2-yl)-3-hydroxybenzoic acid	[M-H]-	281.13943	C15H22O5	Sesquiterpenoids	YCUWMGPYKGLQQF-UHFFFAOYSA-N	O=C(O)C1=CC=C(C(O)=C1)C(O)(C)CCCC(O)(C)C
1990	7.707	326.14691	6-(2-aminopyrimidin-4-yl)-4-butyl-7-hydroxy-8-methyl-2H-chromen-2-one	[M+H]+	326.14999	C18H19N3O3	7-hydroxycoumarins	KMPDNLYICWJCPY-UHFFFAOYSA-N	O=C1OC2=C(C=C(C=3N=C(N=CC3)N)C(O)=C2C)C(=C1)CCCC
1991	7.707	274.27383	ISOPALMITIC ACID	[M+H]+	274.27405	C16H32O2	Long-chain fatty acids	ZONJATNKKGGVSU-UHFFFAOYSA-N	O=C(O)CCCCCCCCCCCCC(C)C
1992	7.71	455.13159	(2R,3S,4S,5R,6R)-5-[(2S,3R,4R)-3,4-dihydroxy-4-(hydroxymethyl)oxolan-2-yl]oxy-2-(hydroxymethyl)-6-(2-phenylethoxy)oxane-3,4-diol	[M+H]+	455.13141	C19H28O10	O-glycosyl compounds	UWKRNCNWJVCHGZ-DERWZFJFSA-N	OCC1OC(OCCC=2C=CC=CC2)C(OC3OCC(O)(CO)C3O)C(O)C1O
1993	7.719	192.13783	DEET	[M+H]+	192.13829	C12H17NO	N,N-dialkyl-m-toluamides	MMOXZBCLCQITDF-UHFFFAOYSA-N	CCN(CC)C(C1=CC=CC(C)=C1)=O
1994	7.733	357.26324	5-[6-(3-hydroxy-4-methoxyphenyl)-1,3,3a,4,6,6a-hexahydrofuro[3,4-c]furan-3-yl]-2-methoxyphenol	[M-H]-	357.2594	C20H22O6	Furanoid lignans	VRHZMFGDDGFRLP-UHFFFAOYSA-N	OC1=CC(=CC=C1OC)C2OCC3C(OCC23)C4=CC=C(OC)C(O)=C4
1995	7.733	325.20099	FA 18:3+3O	[M-H]-	325.20071	C18H30O5	Oxidized fatty acids	PLHUEKVESBJIRC-UHFFFAOYNA-N	O=C(O)CCCCCCCC(=O)C=CC(O)C(O)CC=CCC
1996	7.752	617.36725	(2S,3R,4S,5R)-2-{[2-hydroxy-22-(2-hydroxypropan-2-yl)-3,8,8,17,19-pentamethyl-23,24-	[M-H]-	617.36951	C35H54O9	Cycloartanols and derivatives	LTVCFOSNIVVOBK-RSGHPYSYSA-N	OC1COC(OC2CCC34CC54C(=CCC3C2(C)C)C6(C)C(O)C78OC(CC(C)C7C6(C)CC5)C(O8)C(O)(C)C)C(O)C1O
1997	7.771	663.37299	Medicagenic acid base + O-Hex	[M-H]-	663.37347	C36H56O11	Triterpene saponins	XCHARIIIZLLEBL-UHFFFAOYNA-N	O=C(O)C6(C)(C(OC1OC(CO)C(O)C(O)C1(O))C(O)CC5(C)(C6(CCC3(C)(C5(CC=C2C4CC(C)(C)CCC4(C(=O)O)(CCC23(C)))))))
1998	7.773	391.23688	Periplogenin	[M+H]+	391.23999	C23H34O5	Cardenolides and derivatives	QJPCKAJTLHDNCS-FBAXFMHRSA-N	O=C1OCC(=C1)C2CCC3(O)C4CCC5(O)CC(O)CCC5(C)C4CCC23C
1999	7.774	221.0811	Monoisobutyl phthalate	[M-H]-	221.08194	C12H14O4	Benzoic acid esters	RZJSUWQGFCHNFS-UHFFFAOYSA-N	CC(C)COC(C1=CC=CC=C1C(=O)O)=O
2000	7.775	317.07245	(3aR,4R,6E,10Z,11aR)-4-hydroxy-10-(hydroxymethyl)-3-methylidene-2-oxo-3a,4,5,8,9,11a-hexahydrocyclodeca[b]furan-6-carbaldehyde	[M+H]+	317.07056	C15H18O5	Germacranolides and derivatives	CVISOHZWXIVDFN-GSWZGBFJSA-N	O=CC1=CCCC(=CC2OC(=O)C(=C)C2C(O)C1)CO
2001	7.787	431.09491	Apigetrin	[M-H]-	431.09399	C21H20O10	Flavonoid-7-O-glycosides	KMOUJOKENFFTPU-QNDFHXLGSA-N	O=C1C=C(OC2=CC(OC3OC(CO)C(O)C(O)C3O)=CC(O)=C12)C=4C=CC(O)=CC4
2002	7.801	252.12329	N-[1-(4-methoxy-6-oxopyran-2-yl)-2-methylbutyl]acetamide	[M-H]-	252.12413	C13H19NO4	Pyranones and derivatives	LFXMHSJWYXKODM-UHFFFAOYSA-N	O=C1OC(=CC(OC)=C1)C(N=C(O)C)C(C)CC
2003	7.807	433.11081	Aloenin	[M+H]+	433.11099	C19H22O10	Phenolic glycosides	KFJNVVJUICKJEQ-LQDZTQBFSA-N	O=C1OC(=CC(OC)=C1)C=2C(OC3OC(CO)C(O)C(O)C3O)=CC(O)=CC2C
2004	7.82	173.11754	FA 9:0+1O	[M-H]-	173.11819	C9H18O3	Oxidized fatty acids	XBUXARJOYUQNTC-UHFFFAOYNA-N	O=C(O)CC(O)CCCCCC
2005	7.823	484.13998	Uridine 5_-triphosphate	[M-H]-	484.14001	C9H15N2O15P3	Pyrimidine ribonucleoside	PGAVKCOVUIYSFO-XVFCMESISA-N	O=C1N=C(O)C=CN1C2OC(COP(=O)(O)OP(=O)(O)OP(=O)(O)O)C(O)C2O
2006	7.829	419.13232	liquiritin	[M+H]+	419.13	C21H22O9	Flavonoid O-glycosides	DEMKZLAVQYISIA-KKPFLKIHSA-N	O=C1C2=CC=C(O)C=C2OC(C3=CC=C(OC4OC(CO)C(O)C(O)C4O)C=C3)C1
2007	7.867	171.10194	FA 9:1+1O	[M-H]-	171.10181	C9H16O3	Oxidized fatty acids	WLGDDELKYAWBBL-UHFFFAOYSA-N	O=CCCCCCCCC(=O)O
2008	7.879	360.13171	Rabeprazole	[M+H]+	360.13763	C18H21N3O3S	Sulfinylbenzimidazoles	YREYEVIYCVEVJK-UHFFFAOYNA-N	O=S(C1=NC=2C=CC=CC2N1)CC3=NC=CC(OCCCOC)=C3C
2009	7.879	861.25421	Smiglaside C (Not validated) + 1Acetyl	[M-H]-	861.24689	C40H46O21	Ferulic acid and derivatives	OGAPXYQOTIOKDV-UHFFFAOYNA-N	O=C(OCC3OC(OC1OC(COC(=O)C)C(OC(=O)C)C(O)C1(OC(=O)C))(COC(=O)C)C(OC(=O)C=CC=2C=CC(O)=C(OC)C=2)C3(O))C=CC=4C=C
2010	7.882	571.18127	(E)-2-(2,4,5-trimethoxybenzylidene)-9-(2,3,4-trimethoxyphenyl)-8,9-dihydro-2H-furo[2,3-f]chromene-3,7-dione	[M+H]+	571.17999	C30H28O10	Linear diarylheptanoids	GYZBHTVPNWMIBE-WYMPLXKRSA-N	O=C1OC2=CC=C3C(=O)C(OC3=C2C(C4=CC=C(OC)C(OC)=C4OC)C1)=CC5=CC(OC)=C(OC)C=C5OC
2011	7.885	755.23157	[(2R,3S,4S,5R,6S)-6-[(2S,4R,5R,6S)-2-[2-(3,4-dihydroxyphenyl)-5,7-dihydroxy-4-oxochromen-3-yl]oxy-4,5-dihydroxy-6-methyloxan-3-yl]oxy-3,4,5-	[M-H]-	755.23706	C36H36O18	Flavonoid-3-O-glycosides	LSMKTLJKBSXMMR-YQBWYNSVSA-N	O=C(OCC1OC(OC2C(O)C(O)C(OC2OC=3C(=O)C=4C(O)=CC(O)=CC4OC3C=5C=CC(O)=C(O)C5)C)C(O)C(O)C1O)C=CC6=CC=C(O)C=C6
2012	7.885	491.13141	[5-ethenyl-3-hydroxy-4-(3-hydroxyprop-1-en-2-yl)-2-(3-methoxy-3-oxoprop-1-en-2-yl)-5-methylcyclohexyl] (Z)-4-acetyloxy-2-(hydroxymethyl)but-2-enoate	[M+H]+	491.12585	C23H32O9	Elemane sesquiterpenoids	IWDKJDOUWOTZPS-PXNMLYILSA-N	O=C(OC)C(=C)C1C(O)C(C(=C)CO)C(C=C)(C)CC1OC(=O)C(=CCOC(=O)C)CO
2013	7.888	345.09595	Eupatorin	[M+H]+	345.10001	C18H16O7	7-O-methylated flavonoids	KLAOKWJLUQKWIF-UHFFFAOYSA-N	O=C1C=C(OC=2C=C(OC)C(OC)=C(O)C12)C=3C=CC(OC)=C(O)C3
2014	7.908	438.25751	Bimatoprost	[M+H]+	438.26099	C25H37NO4	Prostaglandins and related compounds	AQOKCDNYWBIDND-GCYMYIANSA-N	O=C(NCC)CCCC=CCC1C(O)CC(O)C1C=CC(O)CCC=2C=CC=CC2
2015	7.921	370.13846	Fleroxacin (Quinodis)	[M+H]+	370.13699	C17H18F3N3O3	Quinoline carboxylic acids	XBJBPGROQZJDOJ-UHFFFAOYSA-N	O=C(O)C1=CN(C2=C(F)C(=C(F)C=C2C1=O)N3CCN(C)CC3)CCF
2016	7.942	323.08047	Salidroside	[M+H]+	323.07999	C14H20O7	O-glycosyl compounds	ILRCGYURZSFMEG-RKQHYHRCSA-N	OC1=CC=C(C=C1)CCOC2OC(CO)C(O)C(O)C2O
2017	7.949	479.09573	5,7-dihydroxy-3-[(2S,3R,4S,5R,6R)-3,4,5-trihydroxy-6-(hydroxymethyl)oxan-2-yl]oxy-2-(3,4,5-trihydroxyphenyl)chromen-4-one	[M-H]-	479.09613	C21H20O13	Flavonoid-3-O-glycosides	FOHXFLPXBUAOJM-MGMURXEASA-N	O=C1C(OC2OC(CO)C(O)C(O)C2O)=C(OC=3C=C(O)C=C(O)C13)C=4C=C(O)C(O)=C(O)C4
2018	7.952	428.33258	Dendroamide C	[M+H]+	428.32901	C21H24N6O5S3	Macrolactams	VBKOUAVWXRGGDI-UHFFFAOYSA-N	O=C1NC(C2=NC(C(=O)NC(C3=NC(=CS3)C(=O)NC(C4=NC1=CS4)CCS(=O)C)C)=C(O2)C)C
2019	7.952	481.11157	Rhodiolin	[M+H]+	481.11301	C25H20O10	Flavonolignans	POVCYOFRCMBMKD-LMNIDFBRSA-N	O=C1C(O)=C(OC2=C3OC(CO)C(OC3=CC(O)=C12)C4=CC=C(O)C(OC)=C4)C=5C=CC(O)=CC5

2020	7.974	250.17645	N,O-Didesmethylvenlafaxine	[M+H]+	250.18016	C15H23NO2	Cyclohexanols	MMSWXJSQCAEDLK-UHFFFAOYNA-N	CNCC(C1=CC=C(O)C=C1)C1(O)CCCCC1
2021	7.979	449.0849	astilbin	[M-H]-	449.07956	C21H22O11	Flavonoid-3-O-glycosides	ZROGCCBNZBKLEL-MPRHSVQHSA-N	O=C1C=2C(O)=CC(O)=CC2OC(C3=CC=C(O)C(O)=C3)C1OC4OC(C)C(O)C(O)C4O
2022	7.984	595.36383	(2R,6R)-6-[(3R,10S,12S,13R,17R)-3-(2-carboxyacetyl)oxy-12-hydroxy-4,4,10,13,14-pentamethyl-2,3,5,6,7,11,12,15,16,17-decahydro-1H-	[M+H]+	595.36047	C34H52O7	Triterpenoids	HMOMGSPWDYKCAH-CBLCVTABSA-N	O=C(O)CC(=O)OC1CCC2(C3=C(CCC2C1(C)C)C4(C)CCC(C(C)CCC(=C)C(C(=O)O)C)C4(C)C(O)C3)C
2023	7.997	270.07587	Dimethenamid OXA	[M-H]-	270.08057	C12H17NO4S	Alpha amino acids and derivatives	HOYCASTVMCEOTP-UHFFFAOYNA-N	COCC(C)N(C(=O)C(O)=O)C1=C(C)SC=C1C
2024	8.008	407.17255	Secoisolariciresinol	[M-H]-	407.17114	C20H26O6	Lignols	PUETUDUXMCLALY-UHFFFAOYNA-N	COC1=CC(CC(CO)C(CO)CC2=CC=C(O)C(OC)=C2)=CC=C1O
2025	8.009	272.09085	(E)-8-(1-(hydroxyimino)ethyl)-4,9-dimethyl-2H-furo[2,3-h]chromen-2-one	[M+H]+	272.09	C15H13NO4	Angular furanocoumarins	VEUCARGUUJVDOF-CXUHLZMHSA-N	O=C1OC2=C(C=CC=3OC(C(=NO)C)=C(C32)C)C(=C1)C
2026	8.009	601.26843	_130131	[M+H]+	601.263	C30H42O11	Terpene lactones	LBRJFDBQNUVAGM-VGCGLKNISA-N	O=C(O)C1=CC2C(C(=O)OC1)C(O)(COC(=O)C3=CC4C(C(=O)OC3)C(O)(CO)CCC4C(C)C)CCC2C(C)C
2027	8.022	252.23137	(2E,4E)-N-(2-methylpropyl)dodeca-2,4-dienamide	[M+H]+	252.23218	C16H29NO	N-acyl amines	BBRMJCAPNGJKEM-AQASXUMVSA-N	OC(=NCC(C)C)C=CC=CCCCCCCC
2028	8.023	533.27032	9-hydroxy-9-[[(E)-2-(hydroxymethyl)-3-[3-(hydroxymethyl)-6-propan-2-ylcyclohex-2-en-1-yl]prop-2-enoyl]oxymethyl]-1-oxo-6-propan-2-yl-	[M-H]-	533.27557	C29H42O9	Terpene lactones	RCOCBDNPQGVEDL-KEBDBYFISA-N	O=C(O)C1=CC2C(C(=O)OC1)C(O)(COC(=O)C(=CC3C=C(CO)CCC3C(C)C)CO)CCC2C(C)C
2029	8.036	495.12662	[(4E)-7-acetyloxy-6-hydroxy-2-methyl-10-oxo-2,3,6,7,8,9-hexahydrooxecin-3-yl] (E)-but-2-enoate	[M+H]+	495.12616	C24H24O10	Hydroxycinnamic acid glycosides	IAVJHQVGTRSIOM-UVMZMRPTSA-N	O=C(OCC1OC(OC(=O)C=CC2=CC=C(O)C=C2)C(O)C(O)C1O)C=CC3=CC=C(O)C=C3
2030	8.052	213.14804	Dihydrojasmonic Acid	[M+H]+	213.14851	C12H20O3	Jasmonic acids	PQEYTAGBXNEUQL-UHFFFAOYSA-N	O=C(O)CC1CCC(=O)C1CCCCC
2031	8.055	598.30341	5-[[4-[5-[[4-[5-[acetyl(hydroxy)amino]pentylamino]-4-oxobutanoyl]-hydroxyamino]pentylamino]-4-oxobutanoyl]-hydroxyamino]pentanoic acid	[M+H]+	598.30585	C25H45N5O10	N-acyl amines	ZMCWALAQRPULMT-UHFFFAOYSA-N	O=C(O)CCCCN(O)C(=O)CCC(O)=NCCCCCN(O)C(=O)CCC(O)=NCCCCCN(O)C(=O)C
2032	8.055	167.14282	Dihydrojasmone	[M+H]+	167.14304	C11H18O	Cyclic ketones	YCIXWYOBMVNGTB-UHFFFAOYSA-N	CCCCCC1=C(C)CCC1=O
2033	8.057	103.03149	Ethylenethiourea	[M+H]+	103.03245	C3H6N2S	Imidazolidines	PDQAZBWRQCGBEV-UHFFFAOYSA-N	C1CNC(=N1)S
2034	8.058	279.09235	Triphenylphosphine oxide	[M+H]+	279.09332	C18H15OP	Phenylphosphines and derivatives	FIQMHBFVRAXMOP-UHFFFAOYSA-N	C1=CC=C(C=C1)P(C2=CC=CC=C2)(C3=CC=CC=C3)=O
2035	8.063	360.21573	9-phenyl-1-(2,4,6-trihydroxyphenyl)nonan-1-one	[M+H]+	360.21692	C21H26O4	Alkyl-phenylketones	OOGZWXIAHJKVAH-UHFFFAOYSA-N	O=C(C=1C(O)=CC(O)=CC1O)CCCCCCCCC=2C=CC=CC2
2036	8.066	260.20001	Vesamicol	[M+H]+	260.2009	C17H25NO	Phenylpiperidines	YSSBJODGIYRAMI-UHFFFAOYNA-N	OC1CCCCC1N2CCC(C=3C=CC=CC3)CC2
2037	8.075	316.1167	Piplartine	[M-H]-	316.11905	C17H19NO5	Cinnamic acids and derivatives	VABYUUZNAVQNPG-BQYQJAHWSA-N	O=C(C=CC1=CC(OC)=C(OC)C(OC)=C1)N2C(=O)C=CCC2
2038	8.078	236.1111	1-(4-methoxyphenyl)isoquinoline	[M+2H]2+	236.11	C16H13NO	Phenylpyridines	VRGXPRJRDIGSKC-UHFFFAOYSA-N	N=1C=CC=2C=CC=CC2C1C=3C=CC(OC)=CC3
2039	8.083	367.20914	Pesticide4_Boscalid_C18H12Cl2N2O_3-Pyridinecarboxamide, 2-chloro-N-(4'-chloro[1,1'-biphenyl]-2-yl)-	[M-H]-	367.203	C18H12Cl2N2O	Aromatic anilides	WYEMLYFITZORAB-UHFFFAOYSA-N	ClC=1C=CC(=CC1)C=2C=CC=CC2N=C(O)C3=CC=CN=C3Cl
2040	8.087	286.1058	arborinine	[M+H]+	286.10599	C16H15NO4	Acridones	ATBZZQPALSPNMF-UHFFFAOYSA-N	O=C1C=2C=CC=CC2N(C3=CC(OC)=C(OC)C(O)=C13)C
2041	8.091	223.16907	KOBUSONE	[M+H]+	223.16925	C14H22O2	Ketones	UETZJEZFLKASPR-UZWIWUQPSA-N	O=C1CCC2OC2(C)CCC3C1CC3(C)C
2042	8.093	234.11253	Euparin	[M+H]+	234.11246	C13H12O3	Benzofurans	OPUFDNZTKHPZHM-UHFFFAOYSA-N	O=C(C1=CC=2C=C(OC2C=C1O)C(=C)C)C
2043	8.097	397.21783	Mitragynine	[M-H]-	397.21329	C23H30N2O4	Alkaloids	LELBFTMXCIIKKX-UHFFFAOYNA-N	CCC1CN2CCC3=C(NC4=C3C(OC)=CC=C4)C2CC1C(=COC)C(=O)OC
2044	8.098	743.19354	jaceidin	[M+H]+	743.18933	C18H16O8	6-O-methylated flavonoids	XUWTZJRCCPNNJR-UHFFFAOYSA-N	O=C1C(OC)=C(OC2=CC(O)=C(OC)C(O)=C12)C=3C=CC(O)=C(OC)C3
2045	8.1	497.28586	Epoxygedunin	[M-H]-	497.28519	C28H34O8	Limonoids	AZGNIKZHFDJEPU-FJONONFLSA-N	O=C(OC1CC2C(C(=O)C3OC3C2(C)C4CCC5(C)C(OC(=O)C6OC65C14C)C7=COC=C7)(C)C)C
2046	8.104	193.08611	Butylparaben	[M-H]-	193.08702	C11H14O3	p-Hydroxybenzoic acid alkyl esters	QFOHBWFCKVYLES-UHFFFAOYSA-N	CCCCOC(C1=CC=C(C=C1)O)=O
2047	8.107	617.40112	Phorbol-12-Myristate-13-Acetate	[M+H]+	617.40002	C36H56O8	Phorbol esters	PHEDXBVPIONUQT-RGYGYFBISA-N	O=C(OC12C(OC(=O)CCCCCCCCCCCCC)C(C)C3(O)C(C=C(CO)CC4(O)C(=O)C(=CC43)C)C1C2(C)C)C
2048	8.123	419.32953	(3S,10R,13R,14R,17R)-17-((2R,E)-5,6-dimethylhept-3-en-2-yl)-10,13-dimethyl-2,3,4,9,10,11,12,13,14,15,16,17-dodecahydro-1H-	[M+H]+	419.32999	C28H44O	Ergosterols and derivatives	DNVPQKQSNYMLRS-QRFHLYGJSA-N	OC1CC2=CC=C3C(CCC4(C)C3CCC4C(C=CC(C)C(C)C)C)C2(C)CC1
2049	8.129	308.22067	Dihydrocapsaicin	[M+H]+	308.22101	C18H29NO3	Methoxyphenols	XJQPQKLURWNAAH-UHFFFAOYSA-N	OC1=CC=C(C=C1OC)CN=C(O)CCCCCCC(C)C
2050	8.142	340.22595	Propoxyphene	[M+H]+	340.22711	C22H29NO2	Stilbenes	XLMALTXPSGQGBX-UHFFFAOYNA-N	O=C(OC(C=1C=CC=CC1)(CC=2C=CC=CC2)C(C)CN(C)C)CC
2051	8.144	403.13757	Hexamethylquercetagetin	[M+H]+	403.138	C21H22O8	7-O-methylated flavonoids	CHXSDKWBSFDZEU-UHFFFAOYSA-N	O=C1C(OC)=C(OC=2C=C(OC)C(OC)=C(OC)C12)C=3C=CC(OC)=C(OC)C3
2052	8.16	387.14737	Cetirizine	[M-H]-	387.1481	C21H25ClN2O3	Diphenylmethanes	ZKLPARSLTMPFCP-UHFFFAOYNA-N	OC(=O)COCCN1CCN(CC1)C(C1=CC=CC=C1)C1=CC=C(Cl)C=C1
2053	8.163	322.20074	Jasmonyl isoleucine	[M-H]-	322.203	C22H32O8	Trichothecenes	PNKLMTPXERFKEN-UHFFFAOYSA-N	O=C(OCC12CC(OC(=O)CC(C)C)C(=CC2OC3C(O)C(O)C1(C)C43OC4)C)C
2054	8.164	375.10617	Casticin	[M+H]+	375.10745	C19H18O8	7-O-methylated flavonoids	PJQLSMYMOKWUJG-UHFFFAOYSA-N	COC1=C(O)C=C(C=C1)C1=C(OC)C(=O)C2=C(O)C(OC)=C(OC)C=C2O1
2055	8.167	417.23776	N-(3-(dimethylamino)propyl)-2-((3,4,8,8-tetramethyl-2-oxo-2,8,9,10-tetrahydropyrano[2,3-f]chromen-5-yl)oxy)acetamide	[M+H]+	417.23801	C23H32N2O5	Angular pyranocoumarins	SYJCVRHCBPLGHM-UHFFFAOYSA-N	O=C1OC2=C(C(OCC(=O)NCCCN(C)C)=CC=3OC(C)(C)CCC32)C(=C1C)C
2056	8.18	385.20999	Corynoxine	[M+H]+	385.20999	C22H28N2O4	Indolizidines	DAXYUDFNWXHGBE-RCFOJTTRSA-N	O=C(OC)C(=COC)C1CC2N(CCC32C(=O)NC=4C=CC=CC43)CC1CC
2057	8.181	286.20013	Viridiflorine	[M+H]+	286.20129	C15H27NO4	Pyrrolizidines	BWQSLRZZOVFVHJ-ABHRYQDASA-N	CC(C)[C=](O)([C=H](C)O)C(=O)OC[C=H]1CCN2CCC[C=H]12
2058	8.182	400.23981	Karacoline	[M+H]+	400.23999	C22H35NO4	Aconitane-type diterpenoid alkaloids	HKQZUYOVMYOFIT-JEJCSOMWSA-N	OC1CCC2(C)CN(CC)C3C4CC2C13C5CC6C(O)C5C4(O)CC6OC
2059	8.192	376.03693	Pesticide4_Prochloraz_C15H16Cl3N3O2_	[M+H]+	376.03799	C15H16Cl3N3O2	Phenol ethers	TVLSRXXIMLFWEO-UHFFFAOYSA-N	O=C(N1C=NC=C1)N(CCOC=2C(Cl)=CC(Cl)=CC2Cl)CCC
2060	8.204	589.16992	genipin 1-gentiobioside	[M+H]+	589.16925	C23H34O15	Iridoid O-glycosides	FYZYXYLPBWLLGI-AUOPOVQUSA-N	O=C(OC)C1=COC(OC2OC(COC3OC(CO)C(O)C(O)C3O)C(O)C(O)C2O)C4C(=CCC14)CO
2061	8.213	827.25452	2-[[4-[4,6-dihydroxy-3-(3-methylbut-2-enyl)-2-[3,4,5-trihydroxy-6-(hydroxymethyl)oxan-2-yl]oxyphenyl]-3-hydroxy-2-(4-hydroxyphenyl)-5-	[M+H]+	827.25208	C39H48O18	Flavonoid-7-O-glycosides	GQOCHCOFYNMUOJ-UHFFFAOYSA-N	OC1=CC=C(C=C1)C2OC=3C=C(OC4OC(CO)C(O)C(O)C4O)C=C(OC)C3C(C=5C(O)=CC(O)=C(C5OC6OC(CO)C(O)C(O)C6O)CC=C(C)C)C2O
2062	8.227	505.27747	Tonantzitlolone B	[M+H]+	505.28101	C28H42O9	Fatty acid esters	BKJAMMLKHPCMJI-SMKITLBYSA-N	O=C(OC1C(C)CC2OC1(O)C(O)C3(OC(CC3)C(C=CC(C2=O)C)(C)C)C)C=C(C)C(OC(=O)C)C

2063	8.245	281.09052	4-hydroxy-1'-methylspiro[benzo[e][1,3]oxazine-2,3'-indolin]-2'-one	[M+H]+	281.09	C16H12N2O3	Benzoxazines	OZPNYAHEDUKMPF-UHFFFAOYSA-N	O=C1N(C=2C=CC=CC2C31N=C(O)C=4C=CC=CC4O3)C
2064	8.256	384.15884	Meropenem	[M+H]+	384.159	C17H25N3O5S	Thienamycins	DMJNNHOOLUXYBV-UHFFFAOYSA-N	O=C(O)C1=C(SC2CNC(C(=O)N(C)C)C2)C(C)C3N1C(=O)C3C(O)C
2065	8.258	578.31488	(2S,3R)-2-(1-((S)-2-(tert-butoxycarbonyl)-1,2,3,4-tetrahydroisoquinoline-3-carbonyl)-4-phenylpiperidine-4-carboxamido)-3-methylpentanoic acid	[M+H]+	578.32001	C33H43N3O6	Hybrid peptides	NBWLCAPFQGXOGJ-ILRLXPBVSA-N	O=C(OC(C)(C)C)N1CC=2C=CC=CC2CC1C(=O)N3CCC(C(=O)NC(C(=O)O)C(C)CC)(C=4C=CC=CC4)CC3
2066	8.258	306.27872	[(1R,5R,9S,13S)-5,9,13-trimethyltetracyclo[11.2.1.0?,??.0?,?]hexadec-14-en-5-yl]methanol	[M+H]+	306.27911	C20H32O	Diterpenoids	CIGQQQTZOIDQQR-SGWMQEFOSA-N	OCC1(C)CCCC2(C)C3CCC4(C=CC3(CCC12)C4)C
2067	8.258	583.2702	4-((11aS)-1,3-dioxo-5-(p-tolyl)-11,11a-dihydro-1H-imidazo[1',5':1,6]pyrido[3,4-b]indol-2(3H,5H,6H)-yl)-N-(4-phenylbutan-2-	[M+H]+	583.27002	C37H34N4O3	Beta carbolines	VHMCLONZXOFDIQ-QPQITGAISA-N	O=C(NC(C)CCC=1C=CC=CC1)C2=CC=C(C=C2)N3C(=O)N4C(C5=CC=C(C=C5)C)C=6NC=7C=CC=CC7C6CC4C3=O
2068	8.258	306.16904	Anisodamine	[M+H]+	306.17001	C17H23NO4	Tropane alkaloids	WTQYWNWRJNXDEG-UHFFFAOYSA-N	O=C(OC1CC2N(C)C(C1)C(O)C2)C(C=3C=CC=CC3)CO
2069	8.26	323.18753	calopocarpin	[M-H]-	323.19284	C20H20O4	Pterocarpans	CYXCYFYWIZXENQ-JXFKEZNVSA-N	OC1=CC=C2C(OC3C4=CC(=C(O)C=C4OCC23)CC=C(C)C)=C1
2070	8.267	461.24326	7-(3,4-dihydroxyphenyl)-1-(4-hydroxyphenyl)-5-[(2S,3R,4S,5R)-3,4,5-trihydroxyoxan-2-yl]oxyheptan-3-one	[M-H]-	461.2435	C24H30O9	Linear diarylheptanoids	KQQLSXWIDDTWRR-WUOZLOAJSA-N	O=C(CCC1=CC=C(O)C=C1)CC(OC2OCC(O)C(O)C2O)CCC3=CC=C(O)C(O)=C3
2071	8.276	267.14206	3,6-diphenylcyclohexane-1,2-diol	[M-H]-	267.13904	C18H20O2	Cyclohexanols	KIDYUEHQXIEBGK-UHFFFAOYSA-N	OC1C(O)C(C=2C=CC=CC2)CCC1C=3C=CC=CC3
2072	8.284	527.45996	N-lignoceroyl-5-hydroxytryptamide	[M+H]+	527.45801	C34H58N2O2	Long-chain N-acylserotonins	QLZQXTNKCAWWES-UHFFFAOYSA-N	O=C(NCCC1=CNC=2C=CC(O)=CC21)CCCCCCCCCCCCCCCCCCCCCCC
2073	8.284	635.4093	O-glucopyranosylepiederagenin, 28-	[M+H]+	635.40997	C36H58O9	Triterpene saponins	WJMMBVSOQPALFO-DLQTVUGOSA-N	O=C(OC1OC(CO)C(O)C(O)C1O)C23CCC(C)(C)CC3C4=CCC5C6(C)CCC(O)C(C)(CO)C6CCC5(C)C4(C)CC2
2074	8.284	165.09103	Phenethylacetate	[M+H]+	165.091	C10H12O2	Benzene and substituted derivatives	MDHYEMXUFSJLGV-UHFFFAOYSA-N	O=C(OCCC=1C=CC=CC1)C
2075	8.303	485.2753	MGMG 16:3	[M-H]-	485.27411	C25H42O9	Lipids	LJVATEUJGAWSPM-UHFFFAOYNA-N	O=C(OC(CO)COC1OC(CO)C(O)C(O)C1(O))CCCCCC=CCC=CCC=CCC
2076	8.326	511.15887	2R,3S,4S,5R,6S)-2-[[(2R,3R,4R)-3,4-dihydroxy-4-(hydroxymethyl)oxolan-2-yl]oxymethyl]-6-[4-hydroxy-3-(3-methylbut-2-enyl)phenoxy]oxane-3,4,5-triol	[M+H]+	511.15762	C22H32O11	Phenolic glycosides	UEAATLOMPYZOFF-PYEGHPNJSA-N	OC1=CC=C(OC2OC(COC3OCC(O)(CO)C3O)C(O)C(O)C2O)C=C1CC=C(C)C
2077	8.331	243.15901	(5E)-3,4,9-trihydroxy-2-propyl-2,3,4,7,8,9-hexahydrooxecin-10-one	[M-H]-	243.15417	C12H20O5	Oxocins	KQJGPGHQDDZVHJ-ZZXKWVIFSA-N	O=C1OC(CCC)C(O)C(O)C=CCCC1O
2078	8.332	529.31622	(2S,3R,5S,9R)-2,3-dihydroxy-14-(hydroxymethyl)-5,9-dimethyl-14-{[(2S,3R,4S,5S,6R)-3,4,5-trihydroxy-6-(hydroxymethyl)oxan-2-	[M-H]-	529.31628	C26H42O11	Diterpene glycosides	QUCGBWQRLWOVBR-BBJJSLPGSA-N	O=C(O)C1(C)CCCC2(C)C3CCC4CC3(CC4(OC5OC(CO)C(O)C(O)C5O)CO)C(O)C(O)C12
2079	8.333	313.1467	Clomipramine	[M-H]-	313.14771	C19H23ClN2	Dibenzazepines	GDLIGKIOYRNHDA-UHFFFAOYSA-N	ClC1=CC=C2C(=C1)N(C=3C=CC=CC3CC2)CCCN(C)C
2080	8.35	582.25183	Tricoumaroyl spermidine	[M-H]-	582.25781	C34H37N3O6	Coumaric acid and derivatives	PFDVWJCSCYDRMZ-UHFFFAOYSA-N	O=C(C=CC1=CC=C(O)C=C1)NCCCN(C(=O)C=CC2=CC=C(O)C=C2)CCCCNC(=O)C=CC3=CC=C(O)C=C3
2081	8.352	343.08032	Coptisine	[M+H]+	343.07999	C19H14NO4+	Protoberberine alkaloids and derivatives	XYHOBCMEDLZUMP-UHFFFAOYSA-N	O1C=2C=CC3=CC=4C5=CC=6OCOC6C=C5CC[N+]4C=C3C2OC1
2082	8.354	304.15405	Cetraxate	[M-H]-	304.15543	C17H23NO4	Phenylpropanoic acids	FHRSHSOEWXUORL-UHFFFAOYSA-N	O=C(O)CCC1=CC=C(OC(=O)C2CCC(CN)CC2)C=C1
2083	8.358	310.27383	Bentyl	[M+H]+	310.27399	C19H35NO2	Carboxylic acid esters	CURUTKGFNZGFSE-UHFFFAOYSA-N	O=C(OCCN(CC)CC)C1(CCCCC1)C2CCCCC2
2084	8.366	356.27881	Aphidicolin	[M+H]+	356.27951	C20H34O4	Aphidicolane and stemodane diterpenoids	NOFOAYPPHIUXJR-APNQCZIXSA-N	OCC1(O)CCC23CC1CC3CCC4C(C)(CO)C(O)CCC42C
2085	8.393	230.24718	N,N-Dimethyldodecylamine N-oxide	[M+H]+	230.24785	C14H31NO	Long-chain alkyl amine oxides	SYELZBGXAIXKHU-UHFFFAOYSA-N	CCCCCCCCCCCCN(C)(C)=O
2086	8.399	276.12268	Convolidine	[M-H]-	276.12411	C15H19NO4	Alkaloids	GWWGRYGNRKFSSX-UHFFFAOYNA-N	COC1=C(O)C=CC(=C1)C(=O)OC1CC2CCC(C1)N2
2087	8.413	417.33566	Sarsasapogenin	[M+H]+	417.33633	C27H44O3	Triterpenoids	GMBQZIIUCVWOCD-WWASVFFGSA-N	C[C=H]1[C=H]2[C=H](C[C=H]3[C=H]4CC[C=H]5C[C=H](O)CC[C=]5(C)[C=H]4CC[C=]23C)O[C=]11CC[C=H](C)CO1
2088	8.421	240.2316	(4,8,8-trimethyldecahydro-1,4-methanoazulen-9-yl)methanol	[M+H]+	240.23218	C15H26O	Sesquiterpenoids	VZJHQHUOVIDRCF-UHFFFAOYSA-N	OCC1C2CCC3C2C(C)(C)CCCC13C
2089	8.43	509.27969	Andrastin (Putative_Observed from A. flavus)	[M+H]+	509.28	C28H38O7	Steroid esters	GRBXNADBNJGZRK-UHFFFAOYSA-N	O=CC12CCC(OC(=O)C)C(C)(C)C2CCC3(C)C1C=C(C)C4(C(=O)C(C(=O)C43C(=O)OC)C)C
2090	8.45	297.11026	2-(2,3-dihydrobenzo[b][1,4]dioxin-6-yl)-6-methylchroman-4-one	[M+H]+	297.10999	C18H16O4	Flavonolignans	FVNYMYXXKXAQHS-UHFFFAOYSA-N	O=C1C2=CC(=CC=C2OC(C3=CC=C4OCCOC4=C3)C1)C
2091	8.46	459.27405	(2R,3S,4S,5R,6R)-2-[[(2S,3R,4R)-3,4-dihydroxy-4-(hydroxymethyl)oxolan-2-yl]oxymethyl]-6-[4-(4-hydroxyphenyl)butan-2-yloxy]oxane-3,4,5-triol	[M-H]-	459.27216	C21H32O11	Fatty acyl glycosides of mono- and disaccharides	QJVVGCSWIOLQDG-PROUKMOUSA-N	OC1=CC=C(C=C1)CCC(OC2OC(COC3OCC(O)(CO)C3O)C(O)C(O)C2O)C
2092	8.469	488.37164	15-formyl-16-hydroxy-1,2,14,17,17-pentamethyl-8-(prop-1-en-2-yl)pentacyclo[11.7.0.0?,??.0?,?.0??,??]icosane-5-carboxylic acid	[M+H]+	488.37341	C30H46O4	Triterpenoids	SLWJVQQNDGLXTK-UHFFFAOYSA-N	O=CC1C(O)C(C)(C)C2CCC3(C)C(CCC4C5C(C(=C)C)CCC5(C(=O)O)CCC43C)C12C
2093	8.482	536.43976	alpha-Carotene	[M+H]+	536.43768	C40H56	Carotenes	ANVAOWXLWRTKGA-JLTXGRSLNA-N	C\C(\C=C\C=C(/C)\C=C\C1C(C)=CCCC1(C)C)=C/C=C/C=C(\C)/C=C/C=C(\C)/C=C/C1=C(C)CCCC1(C)C
2094	8.508	386.2525	Buspirone	[M+H]+	386.25507	C21H31N5O2	N-arylpiperazines	QWCRAEMEVRGPNT-UHFFFAOYSA-N	O=C1CC2(CCCC2)CC(=O)N1CCCCN1CCN(CC1)C1=NC=CC=N1
2095	8.508	358.29504	Putative (3-hydroxyoctadecanoyl)glycine	[M+H]+	358.29501	C20H39NO4	N-acyl-alpha amino acids	PDEJWCOMIACOMR-UHFFFAOYSA-N	O=C(O)CN=C(O)CC(O)CCCCCCCCCCCCCCC
2096	8.512	763.42737	Digitoxin	[M-H]-	763.42737	C41H64O13	Cardenolide glycosides and derivatives	WDJUZGPOPHTGOT-XUDUSOBPSA-N	O=C1OCC(=C1)C2CCC3(O)C4CCC5CC(OC6OC(C)C(OC7OC(C)C(OC8OC(C)C(O)C(O)C8)C(O)C7)C(O)C6)CCC5(C)C4CCC23C
2097	8.537	280.13458	Remerine	[M+H]+	280.13321	C18H17NO2	Alkaloids	JCTYWRARKVGOBK-UHFFFAOYNA-N	CN1CCC2=CC3=C(OCO3)C3=C2C1CC1=CC=CC=C31
2098	8.538	309.10275	Picrotin	[M-H]-	309.10239	C15H18O7	Furopyrans	RYEFFICCPKWYML-QCGISDTRSA-N	O=C1OC2C3OC(=O)C45OC5CC(O)(C1C2C(O)(C)C)C34C
2099	8.575	541.17023	(2R,3R)-3,5-dihydroxy-2-(4-hydroxyphenyl)-8-(3-methylbut-2-en-1-yl)-7-(((2S,3R,4S,5S,6R)-3,4,5-trihydroxy-6-(hydroxymethyl)tetrahydro-2H-pyran-	[M+H]+	541.16998	C26H30O11	Flavonoid-7-O-glycosides	GRDZTDZJQRPNCN-YIANMRPHSA-N	O=C1C2=C(O)C=C(OC3OC(CO)C(O)C(O)C3O)C(=C2OC(C4=CC=C(O)C=C4)C1O)CC=C(C)C
2100	8.58	540.47223	zeta-Carotene	[M+H]+	540.46893	C40H60	Carotenes	BIWLELKAFXRPDE-WTXAYMOSSA-N	CC(C)=CCC\C(C)=C\CC\C(C)=C\C=C\C(\C)=C\C=C\C=C(/C)\C=C\C=C(/C)CC\C=C(/C)CCC=C(C)C
2101	8.589	384.14633	Yaequinolone B_120182	[M+H]+	384.14499	C21H21NO6	Phenylquinolines	XYRSQOXRWJBEIG-PXODQFSGSA-N	O=C(C=CC1=CC=C2NC(=O)C(OC)C(O)(C3=CC=C(OC)C=C3)C2=C1O)C
2102	8.601	367.11938	Lasepitin	[M+H]+	367.1152	C19H20O6	Angular pyranocoumarins	QPLSCFLMIOADPA-YHYXMXQVSA-N	O=C1OC2=C(C=C1)C=CC=3OC(C)(C)C(O)C(OC(=O)C(=CC)C)C32
2103	8.62	305.17374	FA 18:5+2O	[M-H]-	305.17459	C18H26O4	Oxidized fatty acids	PQPRTPXWQQQKJC-UHFFFAOYSA-N	O=C(O)CCCCCCC(=O)C=CC=CC=CC(=O)CC
2104	8.622	332.98032	6-chloro-4,5,7-trihydroxy-9,10-dioxoanthracene-2-carboxylic acid	[M-H]-	332.98074	C15H7ClO7	Anthracenecarboxylic acids	JGHTWRZCNLXYPF-UHFFFAOYSA-N	O=C(O)C1=CC(O)=C2C(=O)C3=C(O)C(Cl)=C(O)C=C3C(=O)C2=C1
2105	8.669	367.11569	(2R,3S,4S,5R,6S)-2-(hydroxymethyl)-6-(4-hydroxy-2-methyl-5-propan-2-ylphenoxy)oxane-3,4,5-triol	[M+H]+	367.11536	C16H24O7	Phenolic glycosides	XFPSRAYOBUHKRV-IBEHDNSVSA-N	OC=1C=C(C(OC2OC(CO)C(O)C(O)C2O)=CC1C(C)C)C

2106	8.688	425.37234	17(21)-Hopen-6-one	[M+H]+	425.37201	C30H48O	Hopanoids	OZFAGHUNGAZEMO-VKNUIDEPSA-N	O=C1CC2(C)C(CCC3C4(C(=C(CC4)C(C)C)CCC32C)C)C5(C)CCCC(C)(C)C15
2107	8.709	298.09604	5'-Methylthioadenosine	[M+H]+	298.09683	C11H15N5O3S	5'-deoxy-5'-thionucleosides	WUUGFSXJNOTRMR-UHFFFAOYNA-N	OC1C(O)C(OC1N2C=NC=3C(=NC=NC32)N)CSC
2108	8.72	252.13965	(E)-3-phenyl-N-(2-phenylethyl)prop-2-enamide	[M+H]+	252.13829	C17H17NO	Cinnamic acid amides	BOSUEWCVNFFBGV-VAWYXSNFSA-N	OC(=NCCC=1C=CC=CC1)C=CC=2C=CC=CC2
2109	8.733	343.08133	5,7-dihydroxy-3,6-dimethoxy-2-(4-methoxyphenyl)-4H-chromen-4-one	[M-H]-	343.08099	C18H16O7	6-O-methylated flavonoids	DWZAJFZEYZIHPO-UHFFFAOYSA-N	O=C1C(OC)=C(OC2=CC(O)=C(OC)C(O)=C12)C=3C=CC(OC)=CC3
2110	8.733	285.12445	Brazilin	[M-H]-	285.11926	C16H14O5	1-benzopyrans	UWHUTZOCTZJUKC-JKSUJKDBSA-N	OC1=CC=C2C(OCC3(O)CC4=CC(O)=C(O)C=C4C23)=C1
2111	8.768	187.13326	10-Hydroxydecanoic acid	[M-H]-	187.13397	C10H20O3	Medium-chain hydroxy acids and derivatives	YJCJVMMDTBEITC-UHFFFAOYSA-N	O=C(O)CCCCCCCCCO
2112	8.771	282.15103	N-nornuciferine	[M+H]+	282.14999	C18H19NO2	Aporphines	QQKAHDMMPBQDAC-UHFFFAOYSA-N	O(C=1C=C2C3=C(C1OC)C4=CC=CC=C4CC3NCC2)C
2113	8.783	633.40021	Echinocystic acid-3-O-glucoside	[M-H]-	633.40082	C36H58O9	Triterpene saponins	WYDPEADEZMZKNM-UHFFFAOYNA-N	CC1(C)CCC2(C(O)CC3(C)C(=CCC4C5(C)CCC(OC6OC(CO)C(O)C(O)C6O)C(C)(C)C5CCC34C)C2C1)C(O)=O
2114	8.784	559.30896	MGMG 18:3	[M-H]-	559.31073	C27H46O9	Lipids	HUSISCNTLUEZCN-UHFFFAOYNA-N	O=C(OCC(O)COC1OC(CO)C(O)C(O)C1(O))CCCCCCCC=CCC=CCC=CCC
2115	8.786	346.3306	icos-19-ene-1,2,4-triol	[M+H]+	346.33154	C20H40O3	Long-chain fatty alcohols	QVLYXPULBWQTJW-UHFFFAOYSA-N	OCC(O)CC(O)CCCCCCCCCCCCCC=C
2116	8.791	561.32367	(2S,3R,4S,5S,6R)-2-[4-[[(3S,4R,5S)-3-hydroxy-5-(4-hydroxy-3-methoxyphenyl)-4-(hydroxymethyl)oxolan-3-yl]methyl]-2-methoxyphenoxy]-6-	[M+H]+	561.32379	C26H34O12	Lignan glycosides	SXGSYHDLSPXCMU-CTQTXEDXSA-N	OC1=CC=C(C=C1OC)C2OCC(O)(CC3=CC=C(OC4OC(CO)C(O)C(O)C4O)C(OC)=C3)C2CO
2117	8.795	287.18497	2-decyl-3-hydroxypentanedioic acid	[M-H]-	287.1864	C15H28O5	Long-chain fatty acids	KOLGVIJQEZBWIQ-UHFFFAOYSA-N	O=C(O)CC(O)C(C(=O)O)CCCCCCCCCC
2118	8.825	765.44208	Cauloside C (3-Glu(1-2)Ara Hederagenin) (NMR)	[M-H]-	765.44305	C41H66O13	Triterpenoids	RROGHRHLBLVQSG-UUWFFIQNSA-N	CC1(C)CC[C=]2(CC[C=]3(C)C(=CC[C=H]4[C=]5(C)CC[C=H](O[C=H]6OC[C=H](O)[C=H](O)[C=H]6O[C=H]6O[C=H](CO)[C=H](O)[
2119	8.825	438.34378	tomatidine	[M+H]+	438.34	C27H45NO2	Spirosolanes and derivatives	XYNPYHXGMWJBLV-JYAGXCGRSA-N	OC1CCC2(C)C(CCC3C2CCC4(C)C3CC5OC6(NCC(C)CC6)C(C)C54)C1
2120	8.835	302.17462	Dobutamine	[M+H]+	302.17508	C18H23NO3	Catecholamines and derivatives	JRWZLRBJNMZMFE-UHFFFAOYNA-N	CC(CCC1=CC=C(O)C=C1)NCCC1=CC(O)=C(O)C=C1
2121	8.859	179.10637	(4-methylcyclohex-3-ene-1,1-diyl)dimethanol	[M+H]+	179.10001	C9H16O2	Primary alcohols	JTRLVVUONAUOHI-UHFFFAOYSA-N	OCC1(CO)CC=C(C)CC1
2122	8.859	359.11212	5-hydroxy-3,6,7-trimethoxy-2-(4-methoxyphenyl)-4H-chromen-4-one	[M+H]+	359.112	C19H18O7	7-O-methylated flavonoids	ADNCDMHZHONBRR-UHFFFAOYSA-N	O=C1C(OC)=C(OC=2C=C(OC)C(OC)=C(O)C12)C=3C=CC(OC)=CC3
2123	8.866	453.34576	Boldenone_Undecylenate	[M+H]+	453.33633	C30H44O3	Steroid esters	AHMMSNQYOPMLSX-KEZAGTACSA-N	CC12CC[C=H]3C(CCC4=CC(=O)C=CC34C)[C=H]1CC[C=H]2OC(=O)CCCCCCCCC=C
2124	8.904	297.1116	[(2E)-2-hexa-2,4-diynylidene-1,6-dioxaspiro[4.4]nona-3,7-dien-9-yl] 3-methylbut-2-enoate	[M+H]+	297.11212	C18H16O4	Fatty acid esters	VJOIIYDQDMELSU-OVCLIPMQSA-N	O=C(OC1C=COC21OC(=CC#CC#CC)C=C2)C=C(C)C
2125	8.957	519.2373	neoandrographolide	[M+H]+	519.23547	C26H40O8	Diterpene glycosides	YGCYRQKJYWQXHG-RDNQFMDVSA-N	O=C1OCC=C1CCC2C(=C)CCC3C(C)(COC4OC(CO)C(O)C(O)C4O)CCCC23C
2126	8.958	547.34485	N-[5-[[4-[4-[acetyl(hydroxy)amino]butylamino]-4-oxobutanoyl]-hydroxyamino]pentyl]-N'-(5-aminopentyl)-N'-hydroxybutanediamide	[M+H]+	547.34497	C24H46N6O8	N-acyl amines	GUZJCKBQAGGXGH-UHFFFAOYSA-N	O=C(N(O)CCCCN=C(O)CCC(=O)N(O)CCCCCN=C(O)CCC(=O)N(O)CCCCCN)C
2127	8.968	373.12799	Isosinensetin	[M+H]+	373.12799	C20H20O7	8-O-methylated flavonoids	UYCWETIUOAGWIL-UHFFFAOYSA-N	O=C1C=C(OC=2C(OC)=C(OC)C=C(OC)C12)C=3C=CC(OC)=C(OC)C3
2128	8.974	343.08334	Eupatilin	[M-H]-	343.08234	C18H16O7	6-O-methylated flavonoids	DRRWBCNQOKKKOL-UHFFFAOYSA-N	COC1=CC=C(C=C1OC)C1=CC(=O)C2=C(O)C(OC)=C(O)C=C2O1
2129	8.974	353.23441	Prostaglandin E1	[M-H]-	353.23334	C20H34O5	Prostaglandins and related compounds	GMVPRGQOIOIIMI-UHFFFAOYNA-N	O=C(O)CCCCCCC1C(=O)CC(O)C1C=CC(O)CCCCC
2130	8.984	301.21802	dehydroabietic acid	[M+H]+	301.21701	C20H28O2	Diterpenoids	NFWKVWVWBFBAOV-MISYRCLQSA-N	O=C(O)C1(C)CCCC2(C3=CC=C(C=C3CCC12)C(C)C)C
2131	8.984	319.22861	steviol	[M+H]+	319.23001	C20H30O3	Kaurane diterpenoids	QFVOYBUQQBFCRH-VQSWZGCSSA-N	O=C(O)C1(C)CCCC2(C)C1CCC34CC(=C)C(O)(CCC32)C4
2132	8.992	589.31848	"12b-O-[deca-2Z,4E,7Z-trienoyl]-13_-isobutyroyloxy-4b-deoxyphorbol "	[M+H]+	589.31702	C34H46O7	Tigliane and ingenane diterpenoids	FHTSKUYELRWGGJ-PUDBDWLBSA-N	O=C(OC1C(C)C2(O)C3C=C(C(=O)C3CC(=CC2C4C1(OC(=O)C(C)C)C4(C)C)CO)C)C=CC=CCC=CCC
2133	8.992	611.30243	Daurisoline	[M+H]+	611.31	C37H42N2O6	Benzylisoquinolines	BURJAQFYNVMZDV-FIRIVFDPSA-N	OC1=CC=C(C=C1OC2=CC=C(C=C2)CC3C4=CC(OC)=C(OC)C=C4CCN3C)CC5C6=CC(O)=C(OC)C=C6CCN5C
2134	8.997	453.3671	(2R)-2,5,7,8-tetramethyl-2-[(4R,8R)-4,8,12-trimethyltridecyl]-3,4-dihydro-2H-chromen-6-ol	[M+Na]+	453.37	C29H50O2	Tocopherols	GVJHHUAWPYXKBD-IEOSBIPESA-N	OC=1C(=C(C=2OC(C)(CCC2C1C)CCCC(C)CCCC(C)CCCC(C)C)C)C
2135	8.998	225.1501	Methyl Dihydrojasmonate	[M-H]-	225.14961	C13H22O3	Jasmonic acids	KVWWIYGFBYDJQC-UHFFFAOYNA-N	CCCCCC1C(CC(=O)OC)CCC1=O
2136	9.031	229.15651	Carylophyllene Oxide	[M+H]+	229.15628	C14H22O	Epoxides	AMTSLHRSERHBCF-IBQNZTRDSA-N	O1C2CC(=C)C3CC(C)(C)C3CCC12C
2137	9.032	427.1376	N-Fructosyl gamma-glutamyl-S-methylcysteine	[M+H]+	427.13791	C15H26N2O10S	N-Fructosyl peptides	OXQONDBZMLPJFL-UHFFFAOYNA-N	CSCC(NC(=O)CCC(NCC1(O)OCC(O)C(O)C1O)C(O)=O)C(O)=O
2138	9.041	256.05988	Propachlor ESA	[M-H]-	256.06491	C11H15NO4S	Anilides	BFSZJLBDHMMCAH-UHFFFAOYSA-N	CC(C)N(C(=O)CS(O)(=O)=O)C1=CC=CC=C1
2139	9.054	318.29846	Phytosphingosine	[M+H]+	318.30026	C18H39NO3	1,3-aminoalcohols	AERBNCYCJBRYDG-UHFFFAOYNA-N	OCC(N)C(O)C(O)CCCCCCCCCCCCCC
2140	9.061	302.11743	roemerine	[M+H]+	302.12	C18H17NO2	Aporphines	JCTYWRARKVGOBK-CQSZACIVSA-N	O1C=2C=C3C4=C(C2OC1)C5=CC=CC=C5CC4N(C)CC3
2141	9.079	242.21098	tetradec-5-ynoic acid	[M+H]+	242.21144	C14H24O2	Long-chain fatty acids	RHOQRBVFJSAQTR-UHFFFAOYSA-N	O=C(O)CCCC#CCCCCCCCC
2142	9.085	309.2056	FA 18:3+2O	[M-H]-	309.20541	C18H30O4	Oxidized fatty acids	RCMABBHQYMBYKV-UHFFFAOYNA-N	O=C(O)CCCCCCC(=O)C=CC1OC1(CCCCC)
2143	9.085	208.14352	Pesticide6_Ethirimol_C11H19N3O_882476	[M-H]-	208.146	C11H19N3O	Hydroxypyrimidines	BBXXLROWFHWFQY-UHFFFAOYSA-N	OC1=NC(=NCC)NC(=C1CCCC)C
2144	9.085	289.21542	Testosterone	[M+H]+	289.21622	C19H28O2	Androgens and derivatives	MUMGGOZAMZWBJJ-UHFFFAOYNA-N	O=C1C=C2CCC3C(CCC4(C)C(O)CCC34)C2(C)CC1
2145	9.086	302.30704	Dihydrosphingosine	[M+H]+	302.30536	C18H39NO2	1,2-aminoalcohols	OTKJDMGTUTTYMP-UHFFFAOYNA-N	OCC(N)C(O)CCCCCCCCCCCCCCC
2146	9.102	418.27939	bonactin	[M+H]+	418.27991	C21H36O7	Dicarboxylic acids and derivatives	JZCRGJSEBZCNAR-UHFFFAOYSA-N	O=C(O)C(C)C1OC(CC1)CC(OC(=O)C(C)C2OC(CC2)CC(O)CC)C
2147	9.104	327.0076	Tris(1-chloro-2-propyl)phosphate	[M+H]+	327.00812	C9H18Cl3O4P	Trialkyl phosphates	KVMPUXDNESXNOH-UHFFFAOYNA-N	CC(CCl)OP(=O)(OC(C)CCl)OC(C)CCl
2148	9.109	340.28418	(1R,4aS,5R,8aS)-5-(5-hydroxy-3-methylpentyl)-1,4a-dimethyl-6-methylidene-3,4,5,7,8,8a-hexahydro-2H-naphthalene-1-carboxylic acid	[M+H]+	340.28461	C20H34O3	Diterpenoids	NSRKLZRKJJQJLD-KXBJDGJWSA-N	O=C(O)C1(C)CCCC2(C)C(C(=C)CCC12)CCC(C)CCO

2149	9.182	364.13214	NALOXONE HYDROCHLORIDE	[M+H]+	364.13101	C19H22ClNO4	Phenanthrenes and derivatives	RGPDIGOSVORSAK-STHHAXOLSA-N	Cl.O=C1CCC2(O)C3N(CC=C)CCC42C5=C(OC14)C(O)=CC=C5C3
2150	9.203	300.28912	Sphingosine	[M+H]+	300.29001	C18H37NO2	1,2-aminoalcohols	WWUZIQQURGPMPG-KRWOKUGFSA-N	OCC(N)C(O)C=CCCCCCCCCCCCCC
2151	9.209	324.16183	Dimefline	[M+H]+	324.15942	C20H21NO3	7-O-methylated flavonoids	ZXFQRFXLFWWKLX-UHFFFAOYSA-N	COC1=C(CN(C)C)C2=C(C=C1)C(=O)C(C)=C(O2)C1=CC=CC=C1
2152	9.211	311.22409	FA 18:2+2O	[M-H]-	311.22159	C18H32O4	Oxidized fatty acids	JGUNZIWGNMQSBM-UHFFFAOYNA-N	O=C(O)CCCCCCCC(OO)C=CC=CCCCCC
2153	9.239	360.24966	12-epinapelline	[M+H]+	360.25	C22H33NO3	Napelline-type diterpenoid alkaloids	AZAZKLKDEOMJBJ-WMLJXHKFSA-N	OC1C(=C)C2CC13C4CC5C6(C)CN(CC)C4C5(C(O)CC6)C3CC2O
2154	9.264	561.34277	(1R,2S,4R,5S,6S,11R,12R,14S,16R)-5,6,11-trihydroxy-4-(hydroxymethyl)-8,12,15,15-tetramethyl-7-oxo-3-	[M-H]-	561.34326	C32H50O8	Bicyclic monoterpenoids	JGUYJMIAKPTIAH-CPYUNXKESA-N	O=C(OC12CC(C)C3(O)C4C=C(C(=O)C4(O)C(O)C5(OC5C3C1C2(C)C)CO)C)CCCCCCCCCCC
2155	9.264	535.27087	Dihydrocelastryl Diacetate	[M-H]-	535.27069	C33H44O6	Phenanthrenes and derivatives	SAOOBRUHTPONGX-UANCAJPASA-N	O=C(OC=1C=C2C(=C(C1OC(=O)C)C)CC=C3C2(C)CCC4(C)C5CC(C(=O)O)(C)CCC5(C)CCC34C)C
2156	9.264	527.33997	madecassic acid	[M+H]+	527.34003	C30H48O6	Triterpenoids	PRAUVHZJPXOEIF-PHCWQIJGSA-N	O=C(O)C12CCC(C)C(C)C2C3=CCC4C5(C)CC(O)C(O)C(C)(CO)C5C(O)CC4(C)C3(C)CC1
2157	9.267	320.14639	(2S,3S,4S)-3,4-dihydroxy-6-methoxy-3-methyl-7-methylidene-2-pentyl-2,4-dihydropyrano[2,3-c]pyrrol-5-one	[M+2H]2+	320.14682	C15H23NO5	Pyrrolines	PQXBZFLVJGBOAD-VZJVUDMVSA-N	O=C1C2=C(OC(CCCCC)C(O)(C)C2O)C(=C)N1OC
2158	9.292	396.31415	N-Benzylstearamide	[M+H]+	396.32001	C25H43NO	N-acyl amines	IXODJGLAVBPVSW-UHFFFAOYSA-N	O=C(NCC=1C=CC=CC1)CCCCCCCCCCCCCCCCC
2159	9.316	301.12207	Sternbin	[M-H]-	301.12216	C16H14O6	7-O-methylated flavonoids	DSAJORLEPQBKDA-UHFFFAOYSA-N	O=C1C2=C(O)C=C(OC)C=C2OC(C3=CC=C(O)C(O)=C3)C1
2160	9.34	462.34244	D-Glucosyl-beta1-1'-D-erythoro-Sphingosine	[M+H]+	462.34253	C24H47NO7	Glycosphingolipids	HHJTWTPUPVQKNA-JIAPQYILSA-N	CCCCCCCCCCCCC\C=C\[C=H](O)[C=H](N)CO[C=H]1O[C=H](CO)[C=H](O)[C=H](O)[C=H]1O
2161	9.347	563.18951	Glomeratose A	[M+H]+	563.19	C24H34O15	Coumaric acids and derivatives	PQHNJDATPYXLIX-GFBDJRIRSA-N	O=C(OC1C(O)C(OC1(OC2OC(CO)C(O)C(O)C2O)CO)CO)C=CC3=CC(OC)=C(OC)C(OC)=C3
2162	9.348	625.31787	Neferine	[M+H]+	625.32001	C38H44N2O6	Benzylisoquinolines	MIBATSHDJRIUJK-XGDNGBMYSA-N	OC1=CC=C(C=C1OC=2C=C3C(=CC2OC)CCN(C)C3CC4=CC=C(OC)C=C4)CC5C6=CC(OC)=C(OC)C=C6CCN5C
2163	9.4	323.1871	4-(8-prop-1-en-2-yl-3,4,8,9-tetrahydro-2H-furo[2,3-h]chromen-3-yl)benzene-1,3-diol	[M-H]-	323.17963	C20H20O4	Isoflavanols	LBZMXGLPRHGPRE-UHFFFAOYSA-N	OC1=CC=C(C(O)=C1)C2COC=3C(=CC=C4OC(C(=C)C)CC43)C2
2164	9.412	603.33459	4,15,26-trihydroxy-1-oxa-4,9,15,20,26,31-hexazacyclotritriacontane-5,8,16,19,27,30-hexone	[M+H]+	603.33478	C26H46N6O10	Macrolactams	OYFVPOABQXXDNO-UHFFFAOYSA-N	O=C1N(O)CCOCCN=C(O)CCC(=O)N(O)CCCCCN=C(O)CCC(=O)N(O)CCCCCN=C(O)CC1
2165	9.416	325.19995	N-((octahydro-1H-quinolizin-1-yl)methyl)-2,4,5,6-tetrahydrocyclopenta[c]pyrazole-3-carboxamide	[M+H]+	325.20001	C17H26N4O	Quinolizines	IYOACTFZIFFILK-UHFFFAOYSA-N	O=C(NCC1CCCN2CCCCC21)C=3NN=C4C3CCC4
2166	9.418	659.40759	Ginsenoside Rh8	[M+H]+	659.40997	C36H60O9	Triterpenoids	VGJOYFZLAIERID-UHFFFAOYSA-N	O=C1CC2C3(C)CCC(O)C(C)(C)C3C(O)CC2(C)C4(C)CCC(C14)C(OC5OC(CO)C(O)C(O)C5O)(C)CCC=C(C)C
2167	9.423	654.45587	5-[[17-(5,6-dihydroxy-6-methylheptan-2-yl)-3,12-dihydroxy-4,4,10,13,14-pentamethyl-2,3,5,6,7,11,12,15,16,17-decahydro-1H-	[M+H]+	654.45752	C36H60O9	Triterpenoids	SWRXIGFQDQTNKP-UHFFFAOYSA-N	O=C(O)CC(O)(C)CC(=O)OC1CC2(C3=C(CCC2C(C)(C)C1O)C4(C)CCC(C(C)CCC(O)C(O)(C)C)C4(C)C(O)C3)C
2168	9.426	457.36655	Betulinic Acid	[M+H]+	457.36661	C30H48O3	Triterpenoids	QGJZLNKBHJESQX-FZFNOLFKSA-N	O=C(O)C12CCC(C(=C)C)C2C3CCC4C5(C)CCC(O)C(C)(C)C5CCC4(C)C3(C)CC1
2169	9.436	294.21323	tBuO-val-hexanoate (tert-butyl hexanoylvalinate)	[M+H]+	294.21399	C15H29NO3	N-acyl-alpha amino acids and derivatives	NVXHLYRVFXNJGK-UHFFFAOYSA-N	O=C(OC(C)(C)C)C(N=C(O)CCCCC)C(C)C
2170	9.473	435.24942	5-[(Z)-14-(3,5-dihydroxyphenyl)tetradec-10-enyl]benzene-1,3-diol	[M+H]+	435.25055	C26H36O4	Resorcinols	PFIQXUYXVYYERO-ALCCZGGFSA-N	OC=1C=C(O)C=C(C1)CCCC=CCCCCCCCCCC=2C=C(O)C=C(O)C2
2171	9.473	284.29434	C18(Plasm)-18:1 PC	[M+H]+	284.29401	C44H86NO7P	1-(1Z-alkenyl),2-acyl-glycerophosphocholines	ZOTYCHIFTCFAHC-DJRFHFPXSA-N	O=C(OC(COC=CCCCCCCCC=CCCCCCCCC)COP(=O)([O-])OCC[N+](C)(C)C)CCCCCCCCCCCCCCCCC
2172	9.482	418.2933	((6R,6aR,13R,13aS)-2,3,4,6,6a,7,8,9,10,12,13,13a-dodecahydro-1H-6,13-methanodipyrido[1,2-a:3',2'-e]azocin-1-yl)(1-isopropyl-1H-indol-6-	[M+H]+	418.29001	C27H35N3O	Aloperine and related alkaloids	IFQVDHKHKVDYQC-OUJSJNLKSA-N	O=C(C=1C=CC=2C=CN(C2C1)C(C)C)N3CCCC4=CC5CC(CN6CCCCC65)C43
2173	9.499	256.22614	SPI_256.2273_14.6	[M+H]+	256.22711	C15H29NO2	Ketals	AUYSPPNVFQBFHX-UHFFFAOYNA-N	CCNCC1COC2(CCC(CC2)C(C)(C)C)O1
2174	9.537	486.04254	4:2 Fluorotelomer thioether amido sulfonic acid	[M-H]-	486.04608	C13H18F9NO4S2	PFSA	VIHJAWYQCFERKN-UHFFFAOYSA-N	O=C(NC(C)(C)CS(=O)(=O)O)CCSCCC(F)(F)C(F)(F)C(F)(F)C(F)(F)F
2175	9.603	282.27844	Dodemorph	[M+H]+	282.27914	C18H35NO	Morpholines	JMXKCYUTURMERF-UHFFFAOYNA-N	CC1CN(CC(C)O1)C2CCCCCCCCCCC2
2176	9.611	406.14978	3-O-methylfunicone	[M+H]+	406.14963	C20H20O8	Aryl-phenylketones	WGLRJONCGNNMKL-VOTSOKGWSA-N	O=C(OC)C=1C=C(OC)C=C(OC)C1C(=O)C2=COC(C=CC)=C(OC)C2=O
2177	9.626	307.19278	FA 18:4+2O	[M-H]-	307.19101	C18H28O4	Oxidized fatty acids	KLFMLBSZQZVKDC-UHFFFAOYNA-N	O=C(O)CCCCCCCC(=O)C=CC=CC=CC(O)CC
2178	9.63	194.08086	N-Methyltyrosine	[M-H]-	194.08226	C10H13NO3	Tyrosine and derivatives	AXDLCFOOGCNDST-UHFFFAOYNA-N	O=C(O)C(NC)CC1=CC=C(O)C=C1
2179	9.631	293.17636	CINCHONIDINE	[M-H]-	293.16592	C19H22N2O	Cinchona alkaloids	KMPWYEUPVWOPIM-KODHJQJWSA-N	OC(C=1C=CN=C2C=CC=CC21)C3N4CCC(C3)C(C=C)C4
2180	9.64	268.10818	13-methoxy-1,6-diazatetracyclo[7.6.1.0?,??.0??,??]hexadeca-3,5,7,9(16),10,12,14-heptaen-2-one	[M+2H]2+	268.10803	C15H10N2O2	Indolonaphthyridine alkaloids	OWCRARVHWCCRAG-UHFFFAOYSA-N	O=C1C=CC2=NC=CC=3C4=CC=C(OC)C=C4N1C23
2181	9.64	135.08035	CINNAMYL ALCOHOL	[M+H]+	135.08044	C9H10O	Cinnamyl alcohols	OOCCDEMITAIZTP-QPJJXVBHSA-N	OC\C=C\C1=CC=CC=C1
2182	9.643	499.1619	(2R,2'S,4'aS,5'R,5''S,6'R,8'aS)-2'-(acetyloxy)-5''-(furan-3-yl)-6'-methyl-2'',8'-dioxo-octahydrodispiro[oxirane-2,1'-naphthalene-5',3''-oxolane]-	[M+H]+	499.16165	C24H28O9	Diterpene lactones	UWRDCNVTKORVMI-QSQWZJICSA-N	O=C(OCC12C(=O)CC(C)C3(C(=O)OC(C4=COC=C4)C3)C2CCC(OC(=O)C)C51OC5)C
2183	9.643	413.19354	(2R,3S,4S,5R,6R)-2-(hydroxymethyl)-6-[4-(4-hydroxy-2,6,6-trimethylcyclohexen-1-yl)butan-2-yloxy]oxane-3,4,5-triol	[M+H]+	413.1936	C19H34O7	Fatty acyl glycosides of mono- and disaccharides	QOLJILMTPKQQQA-KWEBYEANSA-N	OCC1OC(OC(C)CCC2=C(C)CC(O)CC2(C)C)C(O)C(O)C1O
2184	9.644	453.16556	5-O-methylvisammioside	[M+H]+	453.17001	C22H28O10	Furanochromones	QVGFPTYGKPLXPK-OOBAEQHESA-N	O=C1C=C(OC=2C=C3OC(CC3=C(OC)C12)C(OC4OC(CO)C(O)C(O)C4O)(C)C)C
2185	9.645	119.08529	beta-N-Methylaminoalanine	[M+H]+	119.0815	C4H10N2O2	Alpha amino acids	UJVHVMNGOZXSOZ-UHFFFAOYNA-N	O=C(O)C(N)CNC
2186	9.646	460.26678	Bullatine B	[M+H]+	460.267	C24H39NO6	Aconitane-type diterpenoid alkaloids	XRARAKHBJHWUHW-MBVMIALBSA-N	OC1CCC2(COC)CN(CC)C3C4C(OC)C2C13C5CC6C(O)C5C4(O)CC6OC
2187	9.646	589.24646	methyl (4S,5Z,6S)-4-[2-[[(1S,2R,3R,5R)-5-(1,3-dihydroxypropan-2-yl)-3-hydroxy-2-methylcyclopentyl]methoxy]-2-oxoethyl]-5-ethylidene-6-	[M-H]-	589.25018	C27H42O14	Terpene glycosides	ZVTBPVGTFNEYQP-ZQOOCPEKSA-N	O=C(OC)C1=COC(OC2OC(CO)C(O)C(O)C2O)C(=CC)C1CC(=O)OCC3C(C)C(O)CC3C(CO)CO
2188	9.647	409.18906	eudesmin	[M+H]+	409.18918	C22H26O6	Furanoid lignans	PEUUVVGQIVMSAW-RZTYQLBFSA-N	O(C1=CC=C(C=C1OC)C2OCC3C(OCC23)C4=CC=C(OC)C(OC)=C4)C
2189	9.647	517.13593	Isoflavone base + 1O, 1MeO, O-MalonylHex	[M+H]+	517.13489	C25H24O12	Isoflavone O-glycosides	RDTAGQKYPGLCBK-UHFFFAOYNA-N	O=C(O)CC(=O)OCC4OC(OC1=CC=C2C(=O)C(=COC2(=C1))C3=CC=C(OC)C=C3)C(O)C(O)C4(O)
2190	9.649	278.09595	Crystamidine	[M+H]+	278.10001	C18H15NO4	Erythrinanes	NZMLLYZLUYBOGO-UHFFFAOYSA-N	O=C1C=C2C=CC(OC)CC23C=4C=C5OCOC5=CC4C=CN13
2191	9.658	609.26526	Khivorin	[M+H]+	609.26703	C32H42O10	Limonoids	ODLCLZLDYDHRGT-NEGPAABLSA-N	O=C(OC1CC(OC(=O)C)C2(C)C(CC(OC(=O)C)C3(C)C2CCC4(C)C(OC(=O)C)C5OC543)C6=COC=C6)C1(C)C)C

2192	9.663	351.21027	Pesticide4_Tebufenozide_C22H28N2O2_Benzoic acid, 3,5-dimethyl-, 1-(1,1-dimethylethyl)-2-(4-ethylbenzoyl)hydrazide	[M-H]-	351.20801	C22H28N2O2	Benzoic acids and derivatives	QYPNKSZPJQQLRK-UHFFFAOYSA-N	O=C(NN(C(=O)C=1C=C(C=C(C1)C)C)C(C)(C)C)C2=CC=C(C=C2)CC
2193	9.663	213.18513	Tridecanoic acid	[M-H]-	213.186	C13H26O2	Long-chain fatty acids	SZHOJFHSIKHZHA-UHFFFAOYSA-N	CCCCCCCCCCCCC(O)=O
2194	9.666	301.1189	MORIN	[M-H]-	301.11871	C15H10O7	Flavonols	YXOLAZRVSSWPPT-UHFFFAOYSA-N	O=C1C(O)=C(OC=2C=C(O)C=C(O)C12)C=3C=CC(O)=CC3O
2195	9.699	340.1073	Topiramate	[M+H]+	340.10608	C12H21NO8S	Dioxolopyrans	KJADKKWYZYXHBB-UHFFFAOYNA-N	O=S(=O)(OCC12OCC3OC(OC3C2OC(O1)(C)C)(C)C)N
2196	9.702	312.11264	Naulafine	[M+H]+	312.11301	C20H13N3O	Beta carbolines	OLOYVFQOIBGLFJ-UHFFFAOYSA-N	O=C1C2=CN=CC=3C=CC(C32)=C4C=5NC=6C=CC=CC6C5CCN14
2197	9.714	579.28992	Cucurbitacin E	[M+H]+	579.28998	C32H44O8	Cucurbitacins	NDYMQXYDSVBNLL-MUYMLXPFSA-N	O=C(OC(C=CC(=O)C(O)(C)C1C(O)CC2(C)C3CC=C4C(C=C(O)C(=O)C4(C)C)C3(C(=O)CC12C)(C)C)C
2198	9.727	342.29944	Lactose	[M+H]+	342.29999	C12H22O11	O-glycosyl compounds	GUBGYTABKSRVRQ-DCSYEGIMSA-N	OCC1OC(OC2C(O)C(O)C(O)OC2CO)C(O)C(O)C1O
2199	9.757	330.13992	Sodium Houttuyfonate	[M+H]+	330.14001	C14H27NaO5S	Beta-hydroxy ketones	WEFFNAOSGJCISM-UHFFFAOYSA-M	[Na+].O=C(CCCCCCCCCCC)CC(O)S(=O)(=O)[O-]
2200	9.782	485.28745	TOBRAMYCIN	[M+H]+	485.29294	C18H37N5O9	4,6-disubstituted 2-deoxystreptamines	NLVFBUXFDBBNBW-PBSUHMDJSA-N	OCC1OC(OC2C(O)C(OC3OC(CN)C(O)CC3N)C(N)CC2N)C(O)C(N)C1O
2201	9.808	195.10149	BUTYL PARABEN	[M+H]+	195.10156	C11H14O3	p-Hydroxybenzoic acid alkyl esters	QFOHBWFCKVYLES-UHFFFAOYSA-N	O=C(OCCCC)C1=CC=C(O)C=C1
2202	9.825	409.34201	(9S,14S)-10,13-dimethyl-17-(6-methylheptan-2-yl)-2,3,4,7,8,9,10,11,12,13,14,15,16,17-tetradecahydro-1H-	[M+H]+	409.34	C27H46O	Cholesterols and derivatives	HVYWMOMLDIMFJA-GONJIEHTSA-N	OC1CC2=CCC3C(CCC4(C)C(CCC34)C(C)CCCC(C)C)C2(C)CC1
2203	9.837	273.17044	Epiafzelechin (2R,3R)(-)	[M-H]-	273.17474	C15H14O5	Flavan-3-ols	RSYUFYQTACJFML-UKRRQHHQSA-N	OC1=CC=C(C=C1)C2OC=3C=C(O)C=C(O)C3CC2O
2204	9.856	218.12941	(R)-2-amino-3-(1H-indol-3-yl)-N-methylpropanamide oxalate	[M+H]+	218.13	C12H15N3O	Tryptamines and derivatives	YCTBLMQFTMPXBD-SNVBAGLBSA-N	O=C(NC)C(N)CC1=CNC=2C=CC=CC21
2205	9.858	779.45599	Saikosaponin b2	[M-H]-	779.45874	C42H68O13	Triterpene saponins	WRYJYFCCMSVEPQ-UHFFFAOYNA-N	CC1OC(OC2CCC3(C)C(CCC4(C)C3C=CC3=C5CC(C)(C)CCC5(CO)C(O)CC43C)C2(C)CO)C(O)C(OC2OC(CO)C(O)C(O)C2O)C1O
2206	9.865	456.14658	Amygdalin	[M-H]-	456.15112	C20H27NO11	Others	XUCIJNAGGSZNQT-UHFFFAOYNA-N	OCC1OC(OCC2OC(OC(C#N)C3=CC=CC=C3)C(O)C(O)C2O)C(O)C(O)C1O
2207	9.87	349.21576	2,4-bis(3-methylbut-2-enyl)-5-(2-phenylethyl)benzene-1,3-diol	[M-H]-	349.21729	C24H30O2	Stilbenes	PHPXAJCVSGIDFH-UHFFFAOYSA-N	OC=1C=C(C(=C(O)C1CC=C(C)C)CC=C(C)C)CCC=2C=CC=CC2
2208	9.871	437.37347	gamma-sitosterol	[M+H]+	437.37537	C29H50O	Stigmastanes and derivatives	KZJWDPNRJALLNS-VJSFXXLFSA-N	OC1CC2=CCC3C(CCC4(C)C(CCC34)C(C)CCC(CC)C(C)C)C2(C)CC1
2209	9.876	371.17221	SPARTEINE SULFATE	[M+H]+	371.1702	C15H28N2O4S	Sparteine, lupanine, and related alkaloids	FCEHFCFHANDXMB-UMEYXWOPSA-N	O=S(=O)(O)O.N12CCCCC1C3CN4CCCCC4C(C2)C3
2210	9.877	375.19208	Enalapril	[M-H]-	375.19254	C20H28N2O5	Dipeptides	GBXSMTUPTTWBMN-UHFFFAOYNA-N	CCOC(C(CCC1=CC=CC=C1)NC(C)C(N2CCCC2C(=O)O)=O)=O
2211	9.897	317.20975	Cafestol	[M+H]+	317.211	C20H28O3	Naphthofurans	DNJVYWXIDISQRD-JTSSGKSMSA-N	OCC1(O)CC23CCC4C=5C=COC5CCC4(C)C3CCC1C2
2212	9.9	438.32489	(1S,4S,5R,9S,10R,13R,14R)-14-hydroxy-5,9-dimethyl-14-{[(3-methylbutanoyl)oxy]methyl}tetracyclo[11.2.1.0?,??.0?,?]hexadecane-5-	[M+H]+	438.32138	C25H40O5	Kaurane diterpenoids	FIOZUJXSEZUYCS-MTSAZYMTSA-N	O=C(OCC1(O)CC23CCC4C(C(=O)O)(C)CCCC4(C)C3CCC1C2)CC(C)C
2213	9.91	373.29297	Quercetin Pentamethyl Ether	[M+H]+	373.29263	C20H20O7	7-O-methylated flavonoids	ALGDHWVALRSLBT-UHFFFAOYSA-N	O=C1C(OC)=C(OC2=CC(OC)=CC(OC)=C21)C=3C=CC(OC)=C(OC)C3
2214	9.924	721.36835	DGMG 18:3	[M-H]-	721.36603	C33H56O14	Lipids	MPSGDHOYFIUPSO-UHFFFAOYNA-N	O=C(OCC(O)COC2OC(COC1OC(CO)C(O)C(O)C1(O))C(O)C(O)C2(O))CCCCCCCC=CCC=CCC=CCC
2215	9.928	515.2674	Lucidenic acid D	[M+H]+	515.26001	C29H38O8	Triterpenoids	LTJSBYAKDOGXLX-JTJCPSTFSA-N	O=C(OC1C(=O)C2=C(C(=O)CC3C(C(=O)CCC23C)(C)C)C4(C(=O)CC(C(C)CCC(=O)O)C14C)C)C
2216	9.929	413.28748	Leupeptin analogue LVR (or VLR)	[M+H]+	413.28799	C19H36N6O4	Dipeptides	SEQYPUIPGIGBNQ-UHFFFAOYSA-N	O=CC(NC(=O)C(NC(=O)C(NC(=O)C)CC(C)C)C(C)C)CCCN=C(N)N
2217	9.929	264.00366	Parathion-methyl	[M+H]+	264.009	C8H10NO5PS	Phenyl thiophosphates	RLBIQVVOMOPOHC-UHFFFAOYSA-N	COP(OC)(OC1=CC=C(C=C1)N(=O)=O)=S
2218	9.944	283.15561	[5-hydroxy-3-(hydroxymethyl)-2-oxo-6-propan-2-ylcyclohex-3-en-1-yl] 3-methylbutanoate	[M-H]-	283.15509	C15H24O5	Menthane monoterpenoids	LBZXZXIGWNHGTB-UHFFFAOYSA-N	O=C(OC1C(=O)C(=CC(O)C1C(C)C)CO)CC(C)C
2219	9.998	326.23166	Bisoprolol	[M+H]+	326.23257	C18H31NO4	Benzylethers	VHYCDWMUTMEGQY-UHFFFAOYNA-N	CC(C)NCC(COC1=CC=C(C=C1)COCCOC(C)C)O
2220	10.017	377.20795	2-(2-hydroxybut-3-en-2-yl)-3a,6,6,9a-tetramethyl-2,4,5,5a,7,8,9,9b-octahydro-1H-benzo[e][1]benzofuran-4,5-diol	[M+H]+	377.20886	C20H34O4	Naphthofurans	UNORIIWPFZFSEC-UHFFFAOYSA-N	OC1C(O)C2(OC(CC2C3(C)CCCC(C)(C)C13)C(O)(C=C)C)C
2221	10.05	363.21771	HYDROCORTISONE	[M+H]+	363.21701	C21H30O5	21-hydroxysteroids	JYGXADMDTFJGBT-VWUMJDOOSA-N	O=C1C=C2CCC3C4CCC(O)(C(=O)CO)C4(C)CC(O)C3C2(C)CC1
2222	10.053	341.23569	YOHIMBIC ACID HYDRATE	[M+H]+	341.23315	C20H26N2O4	Corynanthean-type alkaloids	UXXCUDYYOMDFPX-GPRFVYSASA-N	O=C(O)C1C(O)CCC2CN3CCC=4C=5C=CC=CC5NC4C3CC12.O
2223	10.061	452.27612	LPE 16:0	[M-H]-	452.27661	C21H44NO7P	Lipids	YVYMBNSKXOXSKW-UHFFFAOYNA-N	O=C(OCC(O)COP(=O)(O)OCCN)CCCCCCCCCCCCCCC
2224	10.064	623.33997	deferrioxamine E	[M+H]+	623.33746	C27H48N6O9	Macrolactams	NHKCCADZVLTPPO-UHFFFAOYSA-N	O=C1N(O)CCCCCN=C(O)CCC(=O)N(O)CCCCCN=C(O)CCC(=O)N(O)CCCCCN=C(O)CC1
2225	10.094	334.3093	(2E,4E,12Z)-N-(2-methylpropyl)octadeca-2,4,12-trienamide	[M+H]+	334.31042	C22H39NO	N-acyl amines	PCWWIOUZYOPZHT-VBUSEHTESA-N	OC(=NCC(C)C)C=CC=CCCCCCCC=CCCCCC
2226	10.099	377.21164	"(8S,9R,10S,11S,13S,14S,16R,17S)-9-fluoro-11-hydroxy-17-(2-hydroxyacetyl)-10,13,16-trimethyl-7,8,11,12,14,15,16,17-octahydro-6H-	[M+H]+	377.21201	C22H29FO4	21-hydroxysteroids	VWVSBHGCDBMOOT-IIEHVVJPSA-N	O=C1C=CC2(C(=C1)CCC3C4CC(C)C(C(=O)CO)C4(C)CC(O)C32F)C
2227	10.132	359.11188	Gardenin B	[M+H]+	359.11301	C19H18O7	8-O-methylated flavonoids	LXEVSYZNYDZSOB-UHFFFAOYSA-N	O=C1C=C(OC2=C(OC)C(OC)=C(OC)C(O)=C12)C=3C=CC(OC)=CC3
2228	10.167	527.33551	Arjungenin	[M+H]+	527.33502	C30H48O6	Triterpenoids	IFIQVSCCFRXSJV-NWCCWSSZSA-N	O=C(O)C12CCC3(C(=CCC4C5(C)CC(O)C(O)C(C)(CO)C5CCC43C)C2C(O)C(C)(C)CC1)C
2229	10.167	401.38135	Campesterol	[M+H]+	401.38	C28H48O	Ergosterols and derivatives	SGNBVLSWZMBQTH-ZRUUVFCLSA-N	OC1CC2=CCC3C(CCC4(C)C(CCC34)C(C)CCC(C)C(C)C)C2(C)CC1
2230	10.169	370.25784	[(1R,2S,3S,4R,4aS)-2,3-dihydroxy-3,4a,8,8-tetramethyl-4-[(E)-3-oxobut-1-enyl]-2,4,5,6,7,8a-hexahydro-1H-naphthalen-1-yl] acetate	[M+H]+	370.25879	C20H32O5	Sesquiterpenoids	BNOWPELKBYSHKU-GFZHNTIHSA-N	O=C(OC1C(O)C(O)(C)C(C=CC(=O)C)C2(C)CCCC(C)(C)C12)C
2231	10.171	424.24683	1-Myristoyl-2-Hydroxy-sn-Glycero-3-Phosphoethanolamine	[M-H]-	424.24695	C19H40NO7P	1-acyl-sn-glycero-3-phosphoethanolamines	RPXHXZNGZBHSMJ-GOSISDBHSA-N	CCCCCCCCCCCCCC(=O)OC[C=H](O)COP(O)(=O)OCCN
2232	10.185	699.35126	Neocoprogen I	[M+H]+	699.34998	C31H50N6O12	N-acyl-alpha amino acids and derivatives	OEZAYKMYHOLRMX-FRCMOREXSA-N	O=C(C=C(C)CCO)N(O)CCCC(NC(=O)C)C(=O)OCCC(=CC(=O)N(O)CCCC1NC(=O)C(NC1=O)CCCN(O)C(=O)C)C
2233	10.185	417.23853	Ramipril	[M+H]+	417.2384	C23H32N2O5	Dipeptides	HDACQVRGBOVJII-UHFFFAOYNA-N	O=C(O)C1N(C(=O)C(NC(C(=O)OCC)CCC=2C=CC=CC2)C)C3CCCC3C1
2234	10.187	694.39655	Monensin sodium salt	[M+H]+	694.39697	C36H61NaO11	Ketals	XOIQMTLWECTKJL-PDHYURILSA-M	[Na+].O=C([O-])C(C)C(OC)C(C)C1OC2(OC(C)(CC2)C3OC(CC)(CC3)C4OC(CC4C)

2235	10.198	270.24213	SPI_270.2429_14.9	[M+H]+	270.24277	C16H31NO2	Ketals	YYICKHZBDGQJBW-UHFFFAOYNA-N	CCCNCC1COC2(CCC(CC2)C(C)(C)C)O1
2236	10.208	402.35745	Sorbitane Monopalmitate - Polysorbate 40 in-source fragment	[M+H]+	402.35999	C22H42O6	Fatty acid esters	IYFATESGLOUGBX-UHFFFAOYSA-N	O=C(OCC(O)C1OCC(O)C1O)CCCCCCCCCCCCCCC
2237	10.21	468.30682	(3S,6S,6aR,7R,7aR,8S,9R,10S,11aR,12R,13S,14R)-1-ethyl-3-(hydroxymethyl)-6,8,10,13-tetramethoxytetradecahydro-1H-3,6a,12-(epiethane[1,1,2]triyl)-	[M+H]+	468.29999	C25H41NO7	Aconitane-type diterpenoid alkaloids	YOTUXHIWBVZAJQ-TYKVCMGYSA-N	OCC12CN(CC)C3C4(O)C(OC)C1C3(C(OC)CC2)C5CC6C(OC)CC4(O)C5C6OC
2238	10.252	311.22458	methyl (2E,4E,8E)-7,13-dihydroxy-4,8,12-trimethyltetradeca-2,4,8-trienoate	[M+H]+	311.22168	C18H30O4	Sesquiterpenoids	NLZZWSRYPUGPEO-QUYVSBDASA-N	O=C(OC)C=CC(=CCC(O)C(=CCCC(C)C(O)C)C)C
2239	10.259	333.20145	dihydroalbocycline	[M+Na]+	333.20361	C18H30O4	Macrolides and analogues	XIHUXEHAQPSMTF-KBVZDLPWSA-N	O=C1OC(C)C(C)CCC=C(C)C(OC)C=CC(O)(C)CC1
2240	10.267	476.27673	LPE 18:3	[M+H]+	476.2756	C23H42NO7P	Lipids	QYXNIRQICOBIAE-CSLWLMPESA-N	O=C(OC(CO)COP(=O)(O)OCCN)CCCCC=CCC=CCC=CCCCCC
2241	10.267	408.17227	Trifluoperazine 2HCl	[M+H]+	408.172	C21H26Cl2F3N3S	Phenothiazines	BXDAOUXDMHXPDI-UHFFFAOYSA-N	Cl.Cl.FC(F)(F)C1=CC=C2SC=3C=CC=CC3N(C2=C1)CCCN4CCN(C)CC4
2242	10.281	203.142	Alanylleucine (isomer of 675)	[M+H]+	203.14011	C9H18N2O3	Dipeptides	RDIKFPRVLJLMER-UHFFFAOYNA-N	O=C(O)C(NC(=O)C(N)C)CC(C)C
2243	10.298	617.23755	(R)-N-((R)-1-(2-aminoacetamido)-4-methyl-1-oxopentan-2-yl)-1-(2-((3-(4-fluorophenyl)-2-methyl-4-oxo-4H-chromen-7-yl)oxy)acetyl)pyrrolidine-2-	[M+H]+	617.23999	C31H35FN4O7	Dipeptides	YYDNTRUSBQCYPN-DNQXCXABSA-N	O=C1C2=CC=C(OCC(=O)N3CCCC3C(=O)NC(C(=O)NC(=O)CN)CC(C)C)C=C2OC(=C1C=4C=CC(F)=CC4)C
2244	10.302	571.28833	Phosphatidylinositol lyso 16	[M-H]-	571.28888	C25H49O12P	2-acyl-sn-glycerol-3-phosphoinositols	NUDRHGDMWNFXMD-PYXWJOJKSA-N	CCCCCCCCCCCCCCCC(=O)OC(CO)COP(O)(=O)O[C=H]1[C=H](O)[C=H](O)[C=H](O)[C=H](O)[C=H]1O
2245	10.303	286.12042	methyl 1-oxo-4-(1H-pyrrol-2-yl)-2H-isoquinoline-3-carboxylate	[M+2H]2+	286.11862	C15H12N2O3	Isoquinolones and derivatives	IADWMRZHOGHTMQ-UHFFFAOYSA-N	O=C(OC)C1=NC(O)=C2C=CC=CC2=C1C3=CC=CN3
2246	10.325	219.13988	1,7,7-trimethylbicyclo[2.2.1]hept-2-yl acetate	[M+H]+	219.14	C12H20O2	Bicyclic monoterpenoids	KGEKLUUHTZCSIP-HOSYDEDBSA-N	O=C(OC1CC2CCC1(C)C2(C)C)C
2247	10.331	562.3136	LPC 18:3	[M+FA-H]-	562.31299	C26H48NO7P	Lipids	WKQNRCYKYCKESD-UHFFFAOYNA-N	O=C(OCC(O)COP(=O)([O-])OCC[N+](C)(C)C)CCCCCCCC=CCC=CCC=CCC
2248	10.445	406.18301	Nebivolol (Bystolic)	[M+H]+	406.18201	C22H26ClF2NO4	1-benzopyrans	JWEXHQAEWHKGCW-VCVZPGOSSA-N	Cl.FC1=CC=C2OC(CCC2=C1)C(O)CNCC(O)C3OC4=CC=C(F)C=C4CC3
2249	10.531	397.26749	Ginkgolic Acid C17-1	[M+H]+	397.26999	C24H38O3	Salicylic acids	MBYNDKVOZOAOIS-FPLPWBNLSA-N	O=C(O)C=1C(O)=CC=CC1CCCCCCCCCC=CCCCCCC
2250	10.54	408.17038	Trifluoperazine	[M+H]+	408.17157	C21H24F3N3S	Phenothiazines	ZEWQUBUPAILYHI-UHFFFAOYSA-N	FC(F)(F)C1=CC=C2SC=3C=CC=CC3N(C2=C1)CCCN4CCN(C)CC4
2251	10.568	574.35614	(3S,10R,13R)-10,13-dimethyl-17-octyl-2,3,4,7,8,9,10,11,12,13,14,15,16,17-tetradecahydro-1H-cyclopenta[a]phenanthren-3-yl (4-nitrophenyl) carbonate	[M+H]+	574.34998	C34H49NO5	Androstane steroids	AFNDTSIACJAGSO-ZNBDOVCMSA-N	O=C(OC1=CC=C(C=C1)[N+](=O)[O-])OC2CC3=CCC4C(CCC5(C)C(CCCCCCCC)CCC45)C3(C)CC2
2252	10.575	279.16187	(1R,2E,7R,10E,12S,13S,15R)-12,15-dihydroxy-7-methyl-8-oxabicyclo[11.3.0]hexadeca-2,10-dien-9-one	[M-H]-	279.16019	C16H24O4	Macrolides and analogues	KQNZDYYTLMIZCT-SWGFJCPHSA-N	O=C1OC(C)CCCC=CC2CC(O)CC2C(O)C=C1
2253	10.579	495.32556	24-epimakisterone A	[M+H]+	495.32999	C28H46O7	Hydroxy bile acids, alcohols and derivatives	IJRBORPEVKCEQD-JMQWOFAPSA-N	O=C1C=C2C(CCC3(C)C(CCC23O)C(O)(C)C(O)CC(C)C(O)(C)C)C4(C)CC(O)C(O)CC14
2254	10.587	528.28351	2-[(4S,5S,5aS,9aS)-4-methoxy-6,6,9a-trimethyl-5-[(2E,4E,6E)-octa-2,4,6-trienoyl]oxy-1-oxo-4,5,5a,7,8,9-hexahydro-3H-benzo[e]isoindol-2-	[M-H]-	528.28381	C29H39NO8	Glutamic acid and derivatives	FXPWZCUQBSEHTH-QZRFOHQJSA-N	O=C(OC1C(OC)C2=C(C(=O)N(C2)C(C(=O)O)CCC(=O)O)C3(C)CCCC(C)(C)C13)C=CC=CC=CC
2255	10.601	353.22574	5,7-dihydroxy-2-(4-methoxyphenyl)-6-(3-methylbut-2-enyl)-2,3-dihydrochromen-4-one	[M-H]-	353.2283	C21H22O5	6-prenylated flavanones	ZTDRTMVBTLHQII-UHFFFAOYSA-N	O=C1C=2C(OC(C3=CC=C(OC)C=C3)C1)=CC(O)=C(C2O)CC=C(C)C
2256	10.606	411.20984	Ingenol-5,20-acetonide	[M+H]+	411.20999	C23H32O5	Ketals	ONMDPPVVEFWDOD-FMBNQKDMSA-N	O=C1C2C=C3COC(OC3C4(O)C(O)C(=CC14C(C)CC5C2C5(C)C)C)(C)C
2257	10.609	248.63942	Tobramycin 2 -N- or 6 -N-formyl	[M+H]+	248.63499	C19H37N5O10	4,6-disubstituted 2-deoxystreptamines	ZAMVUPFBJSDSMB-XWNHDZSNSA-N	O=CNCC1OC(OC2C(O)C(OC3OC(CO)C(O)C(N)C3O)C(N)CC2N)C(N)CC1O
2258	10.615	359.11514	_130094	[M+H]+	359.116	C20H14N4O3	Quinazolines	PRYGKAJEGXTQNF-UHFFFAOYSA-N	O=C1NC(=O)C(N2C(=O)C=3C=CC=CC3N=C12)CC4=CNC=5C=CC=CC54
2259	10.621	610.34027	pseudojervine	[M+H]+	610.34003	C33H49NO8	Steroidal saponins	HYDDDNUKNMMWBD-VWTOGNIVSA-N	O=C1C2=C(C)C3(OC4CC(C)CNC4C3C)CCC2C5CC=C6CC(OC7OC(CO)C(O)C(O)C7O)CCC6(C)C15
2260	10.634	256.13962	2,5-Dimethoxy-4-isopropylthiophenethylamine	[M+2H]2+	256.13699	C13H21NO2S	Dimethoxybenzenes	HDYZSVKZKDPLDT-UHFFFAOYSA-N	O(C1=CC(=C(OC)C=C1SC(C)C)CCN)C
2261	10.642	407.25543	3-(2,4-dihydroxyphenyl)-7-hydroxy-6,8-bis(3-methylbut-2-enyl)-2,3-dihydrochromen-4-one	[M-H]-	407.25299	C25H28O5	8-prenylated isoflavanones	QUBRRXBPXPHDJO-UHFFFAOYSA-N	O=C1C=2C=C(C(O)=C(C2OCC1C3=CC=C(O)C=C3O)CC=C(C)C)CC=C(C)C
2262	10.649	328.31964	Stearoyl Ethanolamide	[M+H]+	328.32001	C20H41NO2	N-acylethanolamines	OTGQIQQTPXJQRG-UHFFFAOYSA-N	O=C(NCCO)CCCCCCCCCCCCCCCCC
2263	10.658	435.2536	19,20-dihydrousambarensine	[M+H]+	435.254	C29H30N4	Harmala alkaloids	QODPASWLKWJHBB-PTORSQSXSA-N	N=1C=CC=2C=3C=CC=CC3NC2C1CC4CC5C=6NC=7C=CC=CC7C6CCN5CC4CC
2264	10.666	396.14536	2-methoxy-3,5-dimethyl-6-[(4Z)-4-[(E)-2-methyl-3-(4-nitrophenyl)prop-2-enylidene]oxolan-2-yl]pyran-4-one	[M-H]-	396.14526	C22H23NO6	Nitrobenzenes	GQKXCBCSVYJUMI-RCGCBBHSSA-N	O=C1C(=C(OC(=C1C)C2OCC(=CC(=CC3=CC=C(C=C3)N(=O)=O)C)C2)OC)C
2265	10.678	211.13217	Jasmonic Acid	[M+H]+	211.133	C12H18O3	Jasmonic acids	ZNJFBWYDHIGLCU-ONEGZZNKSA-N	O=C(O)CC1CCC(=O)C1CC=CCC
2266	10.703	640.34503	Hexosyl LPE 18:2	[M+H]+	640.34412	C29H54NO12P	Lipids	MNNGEUSDGJJARF-JLXBFWJWNA-N	CCCCCCCCCCCCC\C=C\C=C\C(=O)OCC(O)COP(O)(=O)OCCNC1OC(CO)C(O)C(O)C1O
2267	10.726	241.2263	oxacyclohexadecan-2-one	[M+H]+	241.22	C15H28O2	Macrolides and analogues	FKUPPRZPSYCDRS-UHFFFAOYSA-N	O=C1OCCCCCCCCCCCCCC1
2268	10.742	276.15854	Rolipram	[M+H]+	276.159	C16H21NO3	Phenylpyrrolidines	HJORMJIFDVBMOB-UHFFFAOYSA-N	O=C1NCC(C2=CC=C(OC)C(OC3CCCC3)=C2)C1
2269	10.745	351.16949	Perakine	[M+H]+	351.17001	C21H22N2O3	Ajmaline-sarpagine alkaloids	GDXJMOGWONJRHL-FXRWJBKJSA-N	O=CC1C(N2C3C4=NC=5C=CC=CC5C46CC2C(C6OC(=O)C)C1C3)C
2270	10.76	431.24567	(1S,4aS,5S,7aS)-7-(acetyloxymethyl)-5-hydroxy-1-[(2S,3R,4S,5S,6R)-3,4,5-trihydroxy-6-(hydroxymethyl)oxan-2-yl]oxy-1,4a,5,7a-	[M-H]-	431.24692	C18H24O12	Iridoid O-glycosides	DGDWCRWJRNMRKX-DILZHRMZSA-N	O=C(O)C1=COC(OC2OC(CO)C(O)C(O)C2O)C3C(=CC(O)C13)COC(=O)C
2271	10.777	373.35107	3,4',5,6,7-Pentamethoxyflavone	[M+H]+	373.34515	C20H20O7	7-O-methylated flavonoids	OWNRDLYPIYHOQK-UHFFFAOYSA-N	O=C1C(OC)=C(OC=2C=C(OC)C(OC)=C(OC)C21)C=3C=CC(OC)=CC3
2272	10.803	291.19485	FA 18:4+1O	[M-H]-	291.19449	C18H28O3	Oxidized fatty acids	IYEHRWJNUWAXAD-UHFFFAOYNA-N	O=C(O)CCCCCCCC1C(=O)C=CC1(CC=CCC)
2273	10.804	205.15819	3,4-Dimethoxybenzoic acid;Veratric acid	[M+H]+	205.16	C9H10O4	P-methoxybenzoic acids and derivatives	DAUAQNGYDSHRET-UHFFFAOYSA-N	O=C(O)C1=CC=C(OC)C(OC)=C1
2274	10.807	602.25208	(S)-methyl 2-((S)-2-(((S)-7-acetamido-1,2,3-trimethoxy-9-oxo-5,6,7,9-tetrahydrobenzo[a]heptalen-10-yl)amino)-4-	[M+H]+	602.25	C30H39N3O8S	Peptides	QKYMKDOHBLWSEL-YROCYRMSSA-N	O=C1C=C2C(=CC=C1NC(C(=O)NC(C(=O)OC)C)CCSC)C3=C(OC)C(OC)=C(OC)C=C3CCC2NC(=O)C
2275	10.819	277.14505	(3E)-3-benzylidene-4-methyl-1H-1,4-benzodiazepine-2,5-dione	[M-H]-	277.14868	C17H14N2O2	1,4-benzodiazepines	FYVKHLSOIIPVEH-RVDMUPIBSA-N	O=C1C=2C=CC=CC2N=C(O)C(=CC=3C=CC=CC3)N1C
2276	10.821	514.33588	Maraviroc	[M+H]+	514.33502	C29H41F2N5O	Tropane alkaloids	GSNHKUDZZFZSJB-ILVMPNSOSA-N	O=C(NC(C=1C=CC=CC1)CCN2C3CCC2CC(N4C(=NN=C4C(C)C)C)C3)C5CCC(F)(F)CC5
2277	10.859	320.25864	(1R,7R)-7-ethenyl-1,4a,7-trimethyl-3,4,4b,5,6,9,10,10a-octahydro-2H-phenanthrene-1-carboxylic acid	[M+H]+	320.25839	C20H30O2	Diterpenoids	MHVJRKBZMUDEEV-WANGQUDJSA-N	O=C(O)C1(C)CCCC2(C)C3C(=CC(C=C)(C)CC3)CCC12

2278	10.879	608.32068	6-(1H-indol-3-ylmethyl)-9-methyl-3,15-di(propan-2-yl)-1,4,7,10,13,16-hexazabicyclo[16.3.0]henicosane-2,5,8,11,14,17-hexone	[M-H]-	608.32019	C31H43N7O6	Oligopeptides	RXOMDRQVYULRRU-UHFFFAOYSA-N	O=C1N2CCCC2C(O)=NC(C(O)=NCC(O)=NC(C(O)=NC(C(O)=NC1C(C)C)CC3=CNC=4C=CC=CC43)C)C(C)C
2279	10.89	438.26413	1-Lauroyl-2-hydroxy-sn-glycero-3-phosphocholine	[M-H]-	438.2626	C20H42NO7P	1-acyl-sn-glycero-3-phosphocholines	BWKILASWCLJPBO-LJQANCHMSA-N	CCCCCCCCCCCC(=O)OC[C=H](O)COP([O-])(=O)OCC[N+](C)(C)C
2280	10.904	149.02435	2-Carboxybenzaldehyde	[M-H]-	149.02441	C8H6O3	Benzoic acids	DYNFCHNNOHNJFG-UHFFFAOYSA-N	O=CC=1C=CC=CC1C(=O)O
2281	10.918	151.06017	ARABITOL(D)	[M-H]-	151.0612	C5H12O5	Sugar alcohols	HEBKCHPVOIAQTA-QWWZWVQMSA-N	OCC(O)C(O)C(O)CO
2282	10.923	327.0773	Triphenylphosphate	[M+H]+	327.07806	C18H15O4P	Aryl phosphotriesters	XZZNDPSIHUTMOC-UHFFFAOYSA-N	C1=CC=C(C=C1)OP(=O)(OC2=CC=CC=C2)OC3=CC=CC=C3
2283	10.932	239.06728	Dinoseb	[M-H]-	239.06735	C10H12N2O5	Dinitrophenols	OWZPCEFYPSAJFR-UHFFFAOYNA-N	CCC(C)C1=C(O)C(=CC(=C1)N(=O)=O)N(=O)=O
2284	10.933	715.35278	Subsessiline	[M-H]-	715.3501	C43H48N4O6	Alkaloids	RZBFPDQKWUWUCK-UHFFFAOYNA-N	COC(=O)C1=C2NC3=C(C=CC=C3)C22CCN3C2C2(CCOC2C24CC5CC67COC6CCN6CCC8(C76)C6=C(N(C2)C58OC34)C(OC)=CC=C6)C1
2285	10.936	629.22266	(15S,16Z,17S)-16-ethylidene-4-hydroxy-15-{[(2S,3R,4S,5S,6R)-3,4,5-trihydroxy-6-(hydroxymethyl)oxan-2-yl]oxy}-2,10,14,20-	[M+H]+	629.22284	C32H36O13	Macrolides and analogues	YRQNFHSTTZXQBU-HTVHDVGDSA-N	O=C1OCCC2=CC=C(O)C(OC3=CC=C(C=C3)CCOC(=O)CC4C1=COC(OC5OC(CO)C(O)C(O)C5O)C4=CC)=C2
2286	10.939	369.15524	(1R,2E,8S,10R,11S)-10,11-dihydroxy-6-(methoxymethyl)-1,10-dimethyl-5-oxo-4,14-dioxatricyclo[9.2.1.0?,?]tetradeca-2,6-dien-8-yl acetate	[M+2H]2+	369.15439	C18H24O8	Butenolides	OPAWQGXUUDQIPB-SFNIKPSXSA-N	O=C1OC2=CC3(OC(O)(CC3)C(O)(C)CC(OC(=O)C)C2=C1COC)C
2287	10.939	368.15662	methyl 3-(3-hydroxy-5-(4-methoxybenzyl)-1H-pyrazol-4-yl)-3-(pyridin-3-yl)propanoate	[M+2H]2+	368.16	C20H21N3O4	Anisoles	GPHUERASPIVMNY-UHFFFAOYSA-N	O=C(OC)CC(C=1C=NC=CC1)C=2C(O)=NNC2CC3=CC=C(OC)C=C3
2288	10.945	614.3299	Hexosyl LPE 16:0	[M-H]-	614.32831	C27H54NO12P	Lipids	UISOORRILGPHLE-OEYVFKEPSA-N	[H][C=](O)(COC(=O)CCCCCCCCCCCCCCC)COP(O)(=O)OCCNC1OC(CO)C(O)C(O)C1O
2289	10.946	472.3251	Terfenadine	[M+H]+	472.32101	C32H41NO2	Diphenylmethanes	GUGOEEXESWIERI-UHFFFAOYNA-N	CC(C)(C)C1=CC=C(C=C1)C(O)CCCN1CCC(CC1)C(O)(C1=CC=CC=C1)C1=CC=CC=C1
2290	10.949	267.17145	Tri-N-butyl phosphate	[M+H]+	267.17197	C12H27O4P	Trialkyl phosphates	STCOOQWBFONSKY-UHFFFAOYSA-N	CCCCOP(=O)(OCCCC)OCCCC
2291	10.962	701.3678	Cimiracemoside D	[M+H]+	701.38	C37H58O11	Cucurbitacin glycosides	HZIBYJCDCHVSPK-RKUDFBBFSA-N	O=C(OC1CC23CC43CCC(OC5OCC(O)C(O)C5O)C(C)(C)C4CCC2C6(C)C(O)C78OC(CC(C)C7C16C)C(O8)C(O)(C)C)C
2292	10.982	604.3667	desmethylenylnocardamine	[M+H]+	604.36646	C26H46N6O9	Macrolactams	SXTGVXKFOVZYIK-UHFFFAOYSA-N	O=C1N(O)CCCCN=C(O)CCC(=O)N(O)CCCCCN=C(O)CCC(=O)N(O)CCCCCN=C(O)CC1
2293	11.061	197.15291	Nerylacetate	[M+H]+	197.15361	C12H20O2	Fatty alcohol esters	HIGQPQRQIQDZMP-UHFFFAOYSA-N	O=C(OCC=C(C)CCC=C(C)C)C
2294	11.08	378.21191	Donepezil	[M-H]-	378.20746	C24H29NO3	N-benzylpiperidines	ADEBPBSSDYVVLD-UHFFFAOYNA-N	O=C1C2=CC(OC)=C(OC)C=C2CC1CC3CCN(CC=4C=CC=CC4)CC3
2295	11.081	712.25403	PAROMOMYCIN SULFATE	[M-H]-	712.25641	C23H47N5O18S	4,5-disubstituted 2-deoxystreptamines	LJRDOKAZOAKLDU-UDXJMMFXSA-N	O=S(=O)(O)O.OCC1OC(OC2C(OC3OC(CO)C(OC4OC(CN)C(O)C(O)C4N)C3O)C(O)C(N)CC2N)C(N)C(O)C1O
2296	11.091	411.23691	Grayanotoxin I	[M-H]-	411.2388	C22H36O7	Leucothol and grayanotoxane	NXCYBYJXCJWMRY-VGBBEZPXSA-N	O=C(OC1C2CCC3C(O)(C)C4CC(O)C(C)(C)C4(O)C(O)CC13CC2(O)C)C
2297	11.114	104.10658	Choline [M]+	[M+H]+	104.10645	C5H14NO	Cholines	OEYIOHPDSNJKLS-UHFFFAOYSA-N	OCC[N+](C)(C)C
2298	11.151	443.34738	21-hydroxy-10-methoxy-1,4,14,19,19-pentamethyl-2,7,18-trioxapentacyclo[11.9.0.0?.??.0?.?.0??.??]docosa-3(11),4,9-triene-8,17-	[M-H]-	443.35153	C25H32O7	Xanthenes	GBKCPBPYMRGRKL-UHFFFAOYSA-N	O=C1OCC2=C1C(OC)=C3C(OC4(C)CC(O)C5C(OC(=O)CCC5(C)C4C3)(C)C)=C2C
2299	11.152	537.3009	senegenin	[M+H]+	537.29999	C30H45ClO6	12-alpha-hydroxysteroids	CWHJIJJSDGEHNS-RRWWVBFDSA-N	O=C(O)C12CCC3=C(C(CC1)CC4C3(C)CCC5C(C(=O)O)(C)C(O)C(O)CC54C)C2CC(C)(C)CC1
2300	11.16	275.16684	shogaol	[M-H]-	275.16525	C17H24O3	Shogaols	OQWKEEOHDMUXEO-BQYQJAHWSA-N	O=C(C=CCCCCC)CCC1=CC=C(O)C(OC)=C1
2301	11.176	391.24814	"[(1S,7S,8S,8aR)-8-[2-[(2R,4R)-4-hydroxy-6-oxooxan-2-yl]ethyl]-7-methyl-1,2,3,7,8,8a-hexahydronaphthalen-1-yl] (2S)-2-methylbutanoate"	[M+H]+	391.24799	C23H34O5	Delta valerolactones	AJLFOPYRIVGYMJ-INTXDZFKSA-N	O=C1OC(CCC2C(C=CC3=CCCC(OC(=O)C(C)CC)C32)C)CC(O)C1
2302	11.177	249.18427	Matrine	[M+H]+	249.19	C15H24N2O	Matrine alkaloids	ZSBXGIUJOOQZMP-JLNYLFASSA-N	O=C1N2CC3CCCN4CCCC(C2CCC1)C43
2303	11.183	247.16965	(3aR,5aR,9bR)-3a-hydroxy-5a,9-dimethyl-3-methylidene-4,5,6,7,8,9b-hexahydrobenzo[g][1]benzofuran-2-one	[M-H]-	247.16972	C15H20O3	Eudesmanolides, secoeudesmanolides, and	XMPDAVDYIOMTLX-BPLDGKMQSA-N	O=C1OC2C3=C(C)CCCC3(C)CCC2(O)C1=C
2304	11.193	379.22244	Neotame	[M+H]+	379.22275	C20H30N2O5	Peptides	HLIAVLHNDJUHFG-HOTGVXAUSA-N	COC(=O)[C=H](CC1=CC=CC=C1)N=C(O)[C=H](CC(O)=O)NCCC(C)(C)C
2305	11.212	496.37802	1-O-hexadecyl-2-C-methyl-3-phosphatidylcholine	[M+H]+	496.37616	C25H54NO6P	Glycerophosphocholines	BZLKOORCGCYQJZ-UHFFFAOYNA-N	CCCCCCCCCCCCCCCCOCC(C)(O)COP([O-])(=O)OCC[N+](C)(C)C
2306	11.226	610.36517	Apicidin C_120251	[M+H]+	610.362	C33H47N5O6	Oligopeptides	GWLHFNKLJRZBAI-KGKLHAENSA-N	O=C1NC(C(=O)NC(C(=O)N2CCCCC2C(=O)NC1CCCCCC(=O)CC)C(C)C)CC3=CN(OC)C=4C=CC=CC43
2307	11.233	647.33624	4-(3-(((2R,4S,5S,6R)-4,5-dihydroxy-6-methyltetrahydro-2H-pyran-2-yl)oxy)-5,14-dihydroxy-13-methyl-10-((E)-((pyridin-3-	[M+H]+	647.33002	C35H48N2O8	Cardenolide glycosides and derivatives	HGJKGQUGPACAGO-HASSKWOGSA-N	O=C1OCC(=C1)C2CCC3(O)C4CCC5(O)CC(OC6OC(C)C(O)C(O)C6)CCC5(C=NCC=7C=NC=CC7)C4CCC23C
2308	11.233	172.13275	Gabapentin (Neurontin)	[M+H]+	172.133	C9H17NO2	Gamma amino acids and derivatives	UGJMXCAKCUNAIE-UHFFFAOYSA-N	O=C(O)CC1(CN)CCCCC1
2309	11.24	649.39526	(2S,3S,4S,5R,6R)-6-[[(3S,6aR,6bS,8aS,14bR)-8a-carboxy-4-(hydroxymethyl)-4,6a,6b,11,11,14b-hexamethyl-1,2,3,4a,5,6,7,8,9,10,12,12a,14,14a-	[M+H]+	649.39459	C36H56O10	Triterpene saponins	DYFUVQSGLMQLRX-CGBUBQHTSA-N	O=C(O)C1OC(OC2CCC3(C)C4CC=C5C6CC(C)(C)CCC6(C(=O)O)CCC5(C)C4(C)CCC3C2(C)CO)C(O)C(O)C1O
2310	11.246	391.17853	Rosmic acid	[M+H]+	391.17999	C21H26O7	Terpene lactones	CAXSJVGHYYBJKT-GVXCCEMESA-N	O=C(O)C1=C(C=C(C(=O)OC)C(C)C)C(=O)C2OC(=O)C13CCCC(C)(C)C23
2311	11.262	394.1918	Pirenperone	[M+H]+	394.19254	C23H24FN3O2	Alkyl-phenylketones	HXCNRYXBZNHDNE-UHFFFAOYSA-N	O=C1C(=C(N=C2C=CC=CN12)C)CCN3CCC(C(=O)C4=CC=C(F)C=C4)CC3
2312	11.277	318.24219	Drofenine	[M+H]+	318.24277	C20H31NO2	Benzene and substituted derivatives	AGJBLWCLQCKRJP-UHFFFAOYNA-N	CCN(CC)CCOC(=O)C(C1CCCCC1)C1=CC=CC=C1
2313	11.306	422.28882	Talatisamine	[M+H]+	422.29001	C24H39NO5	Aconitane-type diterpenoid alkaloids	BDCURAWBZJMFIK-CVJSJHTBSA-N	OC1C2CC3C1C(O)(CC2OC)C4CC5C6(COC)CN(CC)C4C35C(OC)CC6
2314	11.328	305.20981	8-Shogaol	[M+H]+	305.20999	C19H28O3	Shogaols	LGZSMXJRMTYABD-MDZDMXLPSA-N	O=C(C=CCCCCCCC)CCC1=CC=C(O)C(OC)=C1
2315	11.344	327.2301	14-(hydroxymethyl)-5,9-dimethyltetracyclo[11.2.1.0?,??.0?,?]hexadecane-5-carbaldehyde	[M+H]+	327.22943	C20H32O2	Kaurane diterpenoids	ACXLVKYMISNUFD-UHFFFAOYSA-N	O=CC1(C)CCCC2(C)C1CCC34CC(CO)C(CCC32)C4
2316	11.353	578.25952	methyl (2R)-2-[(1S,2R,4S,9R,10R,14S,15S,17S)-17-(acetyloxy)-9-(furan-3-yl)-1-hydroxy-10,14,16,16-tetramethyl-7,18-dioxo-3,8-	[M+H]+	578.25958	C29H36O11	Limonoids	QBOFNNDGXMMAOI-SQTWQLQFSA-N	O=C(OC1C2(O)C(=O)C(C)(C3CCC4(C)C(OC(=O)CC4C53OC25)C6=COC=C6)C(C(O)C(=O)OC)C1(C)C)C
2317	11.368	806.49792	Phosphatidylserine 16	[M-H]-	806.49774	C44H74NO10P	Phosphatidylserines	PWBBJQOVCTWPIM-LGFBOSHVNA-N	CCCCCCCCCCCCCCCC(=O)OCC(COP(O)(=O)OCC(N)C(O)=O)OC(=O)CC\C=C/C\C=C/C\C=C/C\C=C/C\C=C/C\C=C/CC
2318	11.385	288.15906	galanthamine	[M+2H]2+	288.16	C17H21NO3	Galanthamine-type amaryllidaceae alkaloids	ASUTZQLVASHGKV-SUYBPPKGSA-N	OC1C=CC23C4=C(OC2C1)C(OC)=CC=C4CN(C)CC3
2319	11.41	310.23691	Dihydoroferuloyl Octylamine	[M+H]+	310.2363	C18H31NO3	Ferulic acid and derivatives	HNHHAOUUFVYDEW-UHFFFAOYNA-N	CCCCCCCCNC(O)CCC1=CC(OC)=C(O)C=C1
2320	11.411	311.22107	9-HPODE	[M-H]-	311.22278	C18H32O4	Lineolic acids and derivatives	JGUNZIWGNMQSBM-SIGMCMEVNA-N	CCCCCC=C\C=C\C(CCCCCCCC(O)=O)OO

2321	11.425	349.19492	(E)-4-[(1R,2S,3S,4R,8aS)-2,3,4-trihydroxy-2,5,5,8a-tetramethyl-3,4,4a,6,7,8-hexahydro-1H-naphthalen-1-yl]but-3-en-2-one	[M-H]-	349.19348	C18H3004	Sesquiterpenoids	GUVJPXABQYFWPD-GSZDNMEJSA-N	0=C(C=CC1C(0)(C)C(0)C(0)C2C(C)(C)CCCC12C)C
2322	11.472	336.25244	Eicosanoids_18-HEPE_C20H3003	[M+H]+	336.25299	C20H3003	Hydroxyeicosapentaenoic acids	LRWYBGFSVUBWMO-UAAZXLHOSA-N	0=C(0)CCCC=CCC=CCC=CCC=CC=CC(0)CC
2323	11.487	440.30017	7-[2,6-dimethyl-8-(2-methylbutanoyloxy)-1,2,6,7,8,8a-hexahydronaphthalen-1-yl]-3,5-dihydroxyheptanoic acid	[M+H]+	440.30066	C24H3806	Medium-chain hydroxy acids and derivatives	QLJODMDSTUBWDW-UHFFFAOYSA-N	0=C(0)CC(0)CC(0)CCC1C(C=CC2=CC(C)CC(0C(=0)C(C)CC)C21)C
2324	11.494	622.37769	Pesticide4_Moxidectin_C37H53NO8_(1'R,2R,4Z,4'S,5S,6S,8'R,10'E,13'R,14'E,16'E,20'R,21'R,24'S)-21',24'-Dihydroxy-4-(methoxyimino)-5,11',13',22'-	[M+H]+	622.37299	C37H53N08	Milbemycins	YZBLFMPOMVTDJY-CBYMMZEQSA-N	0=C10C2CC(0C3(0C(C(=CC(C)C)C)C(C(=N0C)C3)C)C2)CC=C(C)CC(C=CC=C4C0C5C(0)C(=CC1C450)C)C
2325	11.503	640.41327	Halocynthiaxanthin acetate	[M+H]+	640.41223	C42H5605	Triterpenoids	ZXBKJGIDFYDFMF-KCMHMNNUSA-N	CC(=0)0[C=H]1CC(C)=C(C#C\C(C)=C\C=C\C(\C)=C\C=C\C=C(/C)\C=C\C=C(/C)C(=0)C[C=]230[C=]2(C)C[C=H](0)CC3(C)C)C(C
2326	11.508	511.33981	asiatic acid	[M+H]+	511.34	C30H4805	Triterpenoids	JXSVIVRDWWRQRT-LHZTXBFTSA-N	0=C(0)C12CCC(C)C(C)C2C3=CCC4C5(C)CC(0)C(0)C(C)(C0)C5CCC4(C)C3(C)CC1
2327	11.512	317.20798	Eicosanoids_9-HOTrE_C18H3003	[M+H]+	317.20901	C18H3003	Lineolic acids and derivatives	RIGGEAZDTKMXSI-MEBVTJQTSA-N	0=C(0)CCCCCCCC(0)C=CC=CCC=CCC
2328	11.526	295.22626	13-OxoODE	[M+H]+	295.22678	C18H3003	Lineolic acids and derivatives	JHXAZBBVQSRKJR-QNQPVOBRSA-N	0=C(0)CCCCCCCC=CC=CC(=0)CCCCC
2329	11.528	567.31122	3',4',5',6'-tetradehydrolongicaudatine Y	[M+H]+	567.31201	[C38H39N40]+	Strychnos alkaloids	INVOSXVUPRCXKV-JGGOXZBPSA-O	0C1C(=CN2C=3C=CC=CC3C45CCN6CC(=CC)C(CC64)C1C25)C7C(=CC)CN8C=CC9=C%10C=CC=CC%10=[NH+]C9=C8C7
2330	11.586	597.30432	Phosphatidylinositol lyso 18	[M-H]-	597.30457	C27H51012P	2-acyl-sn-glycerol-3-phosphoinositols	OVHGUPNHWUHPOM-DBFVMBBUSA-N	CCCCCCCCCCCCC\C=C/CCC(=0)0C(C0)C0P(0)(=0)0[C=H]1[C=H](0)[C=H](0)[C=H](0)[C=H](0)[C=H]10
2331	11.587	313.23599	(9Z,12E)-15,16-dihydroxyoctadeca-9,12-dienoic acid	[M+H]+	313.23734	C18H3204	Lineolic acids and derivatives	LKLLJYJTYPVCID-JVVXYUKTSA-N	0=C(0)CCCCCCCC=CCC=CCC(0)C(0)CC
2332	11.597	348.28854	Palmitoyl Ethanolamide	[M+H]+	348.29001	C18H37N02	Carboximidic acids	HXYVTAGFYLMHSO-UHFFFAOYSA-N	0=C(NCC0)CCCCCCCCCCCCCCC
2333	11.607	414.39227	SOLASODINE	[M+H]+	414.39029	C27H43N02	Spirosolanes and derivatives	KWVISVAMQJWJSZ-VKROHFNGSA-N	0C1CC2=CCC3C(CCC4(C)C3CC50C6(NCC(C)CC6)C(C)C54)C2(C)CC1
2334	11.616	262.1431	suberosin	[M+H]+	262.14377	C15H1603	Coumarins and derivatives	RSZDAYHEZSRVHS-UHFFFAOYSA-N	0=C10C=2C=C(0C)C(=CC2C=C1)CC=C(C)C
2335	11.644	307.15659	8-(3-methoxy-2-(methoxycarbonyl)phenyl)octanoic acid	[M-H]-	307.15399	C17H2405	O-methoxybenzoic acids and derivatives	RTUJIYBESVJPOD-UHFFFAOYSA-N	0=C(0C)C=1C(0C)=CC=CC1CCCCCCCC(=0)0
2336	11.65	478.32898	N-(4-((6aR,8aS)-4-hydroxy-6a,8a,9-trimethyl-3,4,5,6,6a,6b,7,8,8a,8b,11a,12,12a,12b-tetradecahydro-1H-	[M+H]+	478.32999	C29H45N03	Furostanes and derivatives	CNHACNVJEKRLHF-UDCIVARJSA-N	0=C(NCC(C)CCC=10C2CC3C4CC=C5CC(0)CCC5(C)C4CCC3(C)C2C1C)C
2337	11.652	243.19516	beta-Hydroxymyristic acid	[M-H]-	243.19656	C14H2803	Long-chain fatty acids	ATRNZOYKSNPPBF-UHFFFAOYNA-N	0=C(0)CC(0)CCCCCCCCCCC
2338	11.672	267.15979	3-[4-methyl-1-(2-methylpropanoyl)-3-oxocyclohexyl]butanoic acid	[M-H]-	267.16019	C15H2404	Menthane monoterpenoids	ZIOCYJNRYIRTQD-UHFFFAOYSA-N	0=C(0)CC(C)C1(C(=0)C(C)C)CC(=0)C(C)CC1
2339	11.685	540.32983	LPC 16:0	[M+FA-H]-	540.33069	C24H50N07P	Lipids	ASWBNKHCZGQVJV-UHFFFAOYNA-N	0=C(0CC(0)C0P(=0)([0-])0CC[N+](C)(C)C)CCCCCCCCCCCCCCC
2340	11.688	296.14441	Sumatriptan succinate	[M+2H]2+	296.14301	C18H27N306S	Tryptamines and derivatives	PORMUFZNYQJOEI-UHFFFAOYSA-N	0=C(0)CCC(=0)0.0=S(=0)(NC)CC=1C=CC=2NC=C(C2C1)CCN(C)C
2341	11.689	518.31952	(6aR,8aS)-11-(3-acetamido-2-methylpropyl)-6a,8a,9-trimethyl-10-oxo-1,3,4,5,6,6a,6b,7,8,8a,8b,9,10,12,12a,12b-hexadecahydropentaleno[2,1-	[M+H]+	518.32001	C31H45N04	Steroid esters	KESBUZBXJMXUNA-SFXPRKOZSA-N	0=C(0C1CC2=CCC3C4CC5=C(C(=0)C(C)C5C4(C)CCC3C2(C)CC1)CC(C)CNC(=0)C)C
2342	11.691	534.29504	Alpha-Ergocryptine	[M+H]+	534.29932	C32H41N505	Lysergamides	YDOTUXAWKBPQJW-NSLWYYNWSA-N	0=C1N2CCCC2C3(0)0C(N=C(0)C4C=C5C=6C=CC=C7NC=C(C76)CC5N(C)C4)(C(=0)N3C1CC(C)C)C(C)C
2343	11.719	481.31097	emetine	[M+H]+	481.31	C29H40N204	Emetine alkaloids	AUVVAXYIELKVAI-CKBKHPSWSA-N	0(C=1C=C2C(=CC10C)C(NCC2)CC3CC4C5=CC(0C)=C(0C)C=C5CCN4CC3CC)C
2344	11.743	355.1763	DUARTIN (-)	[M+H]+	355.17551	C18H2006	3'-hydroxy,4'-methoxyisoflavonoids	QVVPJFBYFYYVDM-LLVKDONJSA-N	0C1=CC=C2C(0CC(C3=CC=C(0C)C(0)=C30C)C2)=C10C
2345	11.751	561.32635	MGMG 18:2	[M-H]-	561.32898	C27H4809	Lipids	LBHUIJRTHBBWHP-UHFFFAOYNA-N	0=C(0CC(0)C0C10C(C0)C(0)C(0)C1(0))CCCCCCCC=CCC=CCCCCC
2346	11.756	509.2872	_120156	[M+H]+	509.29099	C32H36N402	Alpha amino acids and derivatives	UHTOUBUBRNIMLK-UIOOFZCWSA-N	0=C1NC(C(=0)NC1CC=2C=3C=CC=CC3NC2C(C=C)(C)C)CC=4C=5C=CC=CC5NC4C(C=C)(C)C
2347	11.787	466.29333	Phosphatidylethanolamine lyso 17	[M-H]-	466.29391	C22H46N07P	2-acyl-sn-glycero-3-phosphoethanolamines	SZAICHDWEOHRLW-UHFFFAOYNA-N	CCCCCCCCCCCCCCCCC(=0)0C(C0)C0P(0)(=0)0CCN
2348	11.818	413.37292	Fucosterol	[M+H]+	413.37	C29H480	Stigmastanes and derivatives	OSELKOCHBMDKEJ-QPSGOUHRSA-N	0C1CC2=CCC3C(CCC4(C)C(CCC34)C(C)CCC(=CC)C(C)C)C2(C)CC1
2349	11.837	407.28937	2-hydroxy-4-methoxy-3,5-bis(3-methylbut-2-enyl)-6-(2-phenylethyl)benzoic acid	[M-H]-	407.28934	C26H3204	Stilbenes	KTBAIBUBSGVINZ-UHFFFAOYSA-N	0=C(0)C=1C(0)=C(C(0C)=C(C1CCC=2C=CC=CC2)CC=C(C)C)CC=C(C)C
2350	11.872	653.36469	tert-butyl (1-(4-((7-acetamido-1,2,3-trimethoxy-9-oxo-5,6,7,9-tetrahydrobenzo[a]heptalen-10-yl)amino)butanoyl)piperidin-4-yl)carbamate	[M+H]+	653.35999	C35H48N408	Gamma amino acids and derivatives	IREXLBYIZXPNNP-UHFFFAOYSA-N	0=C(0C(C)(C)C)NC1CCN(C(=0)CCCNC2=CC=C3C4=C(0C)C(0C)=C(0C)C=C4CCC(NC(=0)C)C3=CC2=0)CC1
2351	11.877	407.18576	Flavanole base + 20, 2Prenyl	[M-H]-	407.18579	C25H2805	Prenylated flavanones	LAQLCZKPJGMFRM-UHFFFAOYNA-N	0=C2C=3C=CC(0)=C(C=3(0C(C1=CC=C(0)C(=C1)CC=C(C)C)C2(0)))CC=C(C)C
2352	11.883	532.30115	5-amino-2-(3,4'-dihydroxy-4,4,7,8a-tetramethyl-6'-oxospiro[2,3,4a,5,6,7-hexahydro-1H-naphthalene-8,2'-3,8-dihydrofuro[2,3-e]isoindole]-7'-yl)-5-	[M+H]+	532.3017	C28H38N207	Glutamine and derivatives	VGRPJNIVIGBNNK-UHFFFAOYSA-N	0=C(0)C(N1C(=0)C2=CC(0)=C3C(0C4(C3)C(C)CCC5C(C)(C)C(0)CCC54C)=C2C1)CCC(=N)0
2353	11.897	441.14493	Epicatechin Monogallate	[M-H]-	441.14694	C22H18010	Catechin gallates	LSHVYAFMTMFKBA-TZIWHRDSSA-N	0=C(0C1CC=2C(0)=CC(0)=CC20C1C3=CC=C(0)C(0)=C3)C4=CC(0)=C(0)C(0)=C4
2354	11.897	295.22589	FA 18:3+10	[M+H]+	295.228	C18H3003	Oxidized fatty acids	LUZSWWYKKLTDHU-UHFFFAOYSA-N	0=C(0)CCCCCCCC(=0)C=CC=CCCCCC
2355	11.91	392.17514	Linopirdine	[M+H]+	392.17575	C26H21N30	Indoles and derivatives	YEJCDKJIEMIWRQ-UHFFFAOYSA-N	0=C1N(C=2C=CC=CC2)C=3C=CC=CC3C1(CC=4C=CN=CC4)CC=5C=CN=CC5
2356	11.918	443.31516	Paxisterol_120160	[M+H]+	443.31601	C28H4204	Ergostane steroids	WUKYEOQJBJKFPH-VHCNFDLSSA-N	0C1CCC2(C)C(C1)CC30C34C2CCC56C70C(CC5C(07)(C)CCC(=C)C(C)C)C46
2357	11.932	298.27585	Spiroxamine	[M+H]+	298.27405	C18H35N02	Ketals	PUYXTUJWRLOUCW-UHFFFAOYNA-N	CCCN(CC)CC1C0C2(CCC(CC2)C(C)(C)C)01
2358	11.95	359.21805	ethyl 2-(3-(8-hydroxyoctyl)phenoxy)-2-methylpropanoate	[M+H]+	359.21899	C20H3204	Phenoxyacetic acid derivatives	OMXAYACYQBTOQS-UHFFFAOYSA-N	0=C(0CC)C(0C1=CC=CC(=C1)CCCCCCCC0)(C)C
2359	11.958	289.13022	Diazoxon	[M+H]+	289.13116	C12H21N204P	Dialkyl phosphates	VBLJFQYCTRKKKF-UHFFFAOYSA-N	CC0P(=0)(0CC)0C1=NC(=NC(C)=C1)C(C)C
2360	11.964	361.0675	rabelomycin	[M+H]+	361.06824	C19H1406	Angucyclines	JJOLHRYZQSDLSA-UHFFFAOYSA-N	0=C1C=2C=CC=C(0)C2C(=0)C3=C(0)C=C4C(C(=0)CC(0)(C)C4)=C13
2361	11.969	319.22571	(E)-5-(1,2,4a,5-tetramethyl-7-oxo-3,4,8,8a-tetrahydro-2H-naphthalen-1-yl)-3-methylpent-2-enoic acid	[M+H]+	319.22675	C20H3003	Colensane and clerodane diterpenoids	SLMFLTPPPXRYHP-JLHYYAGUSA-N	0=C(0)C=C(C)CCC1(C)C(C)CCC2(C(=CC(=0)CC21)C)C
2362	11.969	317.07803	8-(2,3-dihydroxy-3-methylbutyl)-7-methoxychromen-2-one	[M+H]+	317.07858	C15H1805	Coumarins and derivatives	KGGUASRIGLRPAX-UHFFFAOYSA-N	0=C10C2=C(C=C1)C=CC(0C)=C2CC(0)C(0)(C)C
2363	11.988	223.0961	Diethyl phthalate	[M+H]+	223.09648	C12H1404	Benzoic acid esters	FLKPEMZONWLCSK-UHFFFAOYSA-N	CC0C(C1=CC=CC=C1C(=0)0CC)=0

2364	12.016	289.14178	(4aR,5S)-9,9a-dihydroxy-3,4a,5-trimethyl-5,6,7,8,8a,9-hexahydro-4H-benzo[f][1]benzofuran-2-one	[M+H]+	289.14102	C15H22O4	Terpene lactones	WVFNARDJAJTRAI-ZRGNYTFBSA-N	O=C1OC2(O)C(=C1C)CC3(C)C(C)CCCC3C2O
2365	12.02	522.35254	LPC 18:1	[M+H]+	522.35632	C26H52NO7P	Lipids	YAMUFBLWGFFICM-UHFFFAOYNA-N	O=C(OCC(O)COP(=O)([O-])OCC[N+](C)(C)C)CCCCCCCC=CCCCCCCCC
2366	12.024	506.32321	Phosphatidylethanolamine lyso 20	[M-H]-	506.32523	C25H50NO7P	2-acyl-sn-glycero-3-phosphoethanolamines	URXPORXPKXWHEF-MSUUIHNZNA-N	CCCCCCCCCCCCCC\C=C/CCC(=O)OC(CO)COP(O)(=O)OCCN
2367	12.031	832.51373	Phosphatidylserine 18	[M-H]-	832.51343	C46H76NO10P	Phosphatidylserines	ZBHJZQBBODCTLM-QRPORDCINA-N	CCCCCCCCCCCCC\C=C/CCC(=O)OCC(COP(O)(=O)OCC(N)C(O)=O)OC(=O)CC\C=C/C\C=C/C\C=C/C\C=C/C\C=C/C\C=C/CC
2368	12.032	621.27112	(E)-6-((7-acetamido-1,2,3-trimethoxy-9-oxo-5,6,7,9-tetrahydrobenzo[a]heptalen-10-yl)amino)-N-(3,4,5,6-tetrahydro-2H-	[M+H]+	621.27002	C33H40N4O6S	Tropones	NIYIYLBOTBYWCY-UHFFFAOYSA-N	O=C(N=C1SC2=C(N1)CCC2)CCCCCNC3=CC=C4C5=C(OC)C(OC)=C(OC)C=C5CCC(NC(=O)C)C4=CC3=O
2369	12.039	396.08395	(2S,3S)-6'-methyl-3-phenylspiro[oxirane-2,7'-quinazolino[3,2-a][1,4]benzodiazepine]-5',13'-dione	[M+H]+	396.07788	C24H17N3O3	Pyrimidodiazepines	TWDKBDSVUUKABK-GBXCKJPGSA-N	O=C1C=2C=CC=CC2N=C3N1C=4C=CC=CC4C(=O)N(C)C53OC5C=6C=CC=CC6
2370	12.039	504.34012	Glycocholic Acid	[M+H]+	504.34152	C26H43NO6	Glycinated bile acids and derivatives	RFDAIACWWDREDC-FRVQLJSFSA-N	O=C(O)CN=C(O)CCC(C)C1CCC2C3C(O)CC4CC(O)CCC4(C)C3CC(O)C12C
2371	12.044	298.27328	Pesticide4_Spiroxamine Isomer 1*_C18H35NO2_N-Ethyl-N-{[8-(2-methyl-2-propanyl)-1,4-dioxaspiro[4.5]dec-2-yl]methyl}-1-propanamine	[M+H]+	298.27399	C18H35NO2	Ketals	PUYXTUJWRLOUCW-UHFFFAOYSA-N	O1CC(OC12CCC(CC2)C(C)(C)C)CN(CC)CCC
2372	12.045	624.30249	LTC4	[M-H]-	624.29602	C30H47N3O9S	Oligopeptides	GWNVDXQDILPJIG-NXOLIXFESA-N	CCCCC\C=C/C\C=C/C=C/C=C/[C=H](SC[C=H](N=C(O)CC[C=H](N)C(O)=O)C(O)=NCC(O)=O)[C=H](O)CCCC(O)=O
2373	12.055	190.04948	Kynurenic acid	[M+H]+	190.04987	C10H7NO3	Quinoline carboxylic acids	HCZHHEIFKROPDY-UHFFFAOYSA-N	c1ccc2c(c1)c(=O)cc(C(=O)O)[nH]2
2374	12.065	205.08562	(3Z,5E)-4-hydroxy-6-(4-hydroxyphenyl)hexa-3,5-dien-2-one	[M+H]+	205.08592	C12H12O3	Styrenes	IWINXMWZYVGFKC-JHLWKMQHSA-N	O=C(C=C(O)C=CC1=CC=C(O)C=C1)C
2375	12.082	210.06622	2-(2-chlorophenyl)-4,4-dimethyl-4,5-dihydrooxazole	[M+H]+	210.06799	C11H12ClNO	Chlorobenzenes	VOMZEVDJNHHMLC-UHFFFAOYSA-N	ClC=1C=CC=CC1C2=NC(C)(C)CO2
2376	12.096	652.41357	(3S,6R,9S,12R,15S,18R)-3-butan-2-yl-4,10,16-trimethyl-6,9,12,15,18-penta(propan-2-yl)-1,7,13-trioxa-4,10,16-triazacyclooctadecane-	[M-H]-	652.41785	C34H59N3O9	Cyclic depsipeptides	UQCSETXJXJTMKO-HLRZJIOYSA-N	O=C1OC(C(=O)N(C)C(C(=O)OC(C(=O)N(C)C(C(=O)OC(C(=O)N(C)C1C(C)C)C(C)C)C(C)CC)C(C)C)C(C)C)C(C)C
2377	12.117	487.38541	hyperin	[M+H]+	487.39001	C21H20O12	Flavonoid-3-O-glycosides	OVSQVDMCBVZWGM-DTGCRPNFSA-N	O=C1C(OC2OC(CO)C(O)C(O)C2O)=C(OC=3C=C(O)C=C(O)C13)C=4C=CC(O)=C(O)C4
2378	12.143	555.28967	Ganoderic Acid G	[M+H]+	555.28998	C30H44O8	Triterpenoids	BPJPBLZKOVIJQD-UHFFFAOYSA-N	O=C(O)C(C)CC(=O)CC(C)C1CC(=O)C2(C3=C(C(=O)C(O)C12C)C4(C)CCC(O)C(C)(C)C4CC3O)C
2379	12.158	377.19916	5-[(4S,8aS)-4-acetyloxy-2,5,5,8a-tetramethyl-3-oxo-4a,6,7,8-tetrahydro-4H-naphthalen-1-yl]-3-methylpentanoic acid	[M-H]-	377.20065	C22H34O5	Diterpenoids	XGQQRJZRTBKBRM-APTQHQDLSA-N	O=C(OC1C(=O)C(=C(CCC(C)CC(=O)O)C2(C)CCCC(C)(C)C12)C)C
2380	12.17	311.08905	5,10-dimethoxy-2,2-dimethylpyrano[3,2-g]chromen-8-one	[M+H]+	311.08899	C16H16O5	Linear pyranocoumarins	QZUNAFWZEXJWGD-UHFFFAOYSA-N	O=C1OC2=C(OC)C=3OC(C=CC3C(OC)=C2C=C1)(C)C
2381	12.208	428.30798	Peimisine	[M+H]+	428.31	C27H41NO3	Jerveratrum-type alkaloids	KYELXPJVGNZIGC-GKFGJCLESA-N	O=C1CC2C3C(=C(C)C4(OC5CC(C)CNC5C4C)CC3)CC2C6(C)CCC(O)CC16
2382	12.21	505.31271	Strepin P1	[M+H]+	505.31	C25H40N6O5	Peptides	ARFSAPKKATWHHO-JERZPMBSSA-N	O=CC(NC(=O)C(NC(=O)C(NC(=O)CC(C)C)CC1=CC=C(O)C=C1)C(C)C)CCCN=C(N)N
2383	12.211	409.29294	CHOLIC ACID	[M+H]+	409.29483	C24H40O5	Trihydroxy bile acids, alcohols and derivatives	BHQCQFFYRZLCQQ-OELDTZBJSA-N	O=C(O)CCC(C)C1CCC2C3C(O)CC4CC(O)CCC4(C)C3CC(O)C12C
2384	12.216	744.48236	Phosphatidylethanolamine (20:1/16:1) Abbr: GPoPE	[M+H]+	744.48798	C41H78NO8P	Phosphatidylethanolamines	VOHAPZCYHVKGHA-ZSIBMOKUSA-N	O=C(OCC(OC(=O)CCCCCCCC=CCCCCCC)COP(=O)(O)OCCN)CCCCCCCC=CCCCCCCCCCC
2385	12.221	414.31937	(2Z)-2-[(E)-6-(hydroxymethyl)-2,4,8,10-tetramethyldodec-2-enylidene]-4-methylpentanedioic acid	[M+H]+	414.32138	C23H40O5	Acyclic diterpenoids	NNPPLRJHBQPBAQ-UKRBZAIKSA-N	O=C(O)C(=CC(=CC(C)CC(CO)CC(C)CC(C)CC)C)CC(C(=O)O)C
2386	12.229	262.6478	Tobramycin, N-propionyl derivative likely	[M+H]+	262.64999	C21H41N5O10	4,6-disubstituted 2-deoxystreptamines	XMVDIYAEPSBCDQ-HXNOWKDOSA-N	O=C(NCC1OC(OC2C(O)C(OC3OC(CO)C(O)C(N)C3O)C(N)CC2N)C(N)CC1O)CC
2387	12.232	445.31314	Menaquinone-4	[M+H]+	445.31009	C31H40O2	Menaquinones	DKHGMERMDICWDU-GHDNBGIDSA-N	O=C1C=2C=CC=CC2C(=O)C(=C1C)CC=C(C)CCC=C(C)CCC=C(C)CCC=C(C)C
2388	12.234	427.30487	Leupeptin	[M+H]+	427.30273	C20H38N6O4	Dipeptides	GDBQQVLCIARPGH-IKGGRYGDSA-N	CC(C)C[C=H](N=C(C)O)C(O)=N[C=H](CC(C)C)C(O)=N[C=H](CCCNC(N)=N)C=O
2389	12.259	449.26788	Celastrol	[M-H]-	449.26971	C29H38O4	Triterpenoids	KQJSQWZMSAGSHN-JJWQIEBTSA-N	O=C1C=C2C(=CC=C3C2(C)CCC4(C)C5CC(C(=O)O)(C)CCC5(C)CCC34C)C(=C1O)C
2390	12.263	291.1384	(E)-5-(3-hydroxy-2,3-dimethyl-2-bicyclo[2.2.1]heptanyl)-2-methylpent-2-enoic acid	[M+2H]2+	291.13568	C15H24O3	Sesquiterpenoids	QPVGQZZKAUMQSM-BJMVGYQFSA-N	O=C(O)C(=CCCC1(C)C2CCC(C2)C1(O)C)C
2391	12.268	324.28888	Linoleoyl Ethanolamide	[M+H]+	324.289	C20H37NO2	N-acylethanolamines	KQXDGUVSAAQARU-HZJYTTRNSA-N	O=C(NCCO)CCCCCCCC=CCC=CCCCCC
2392	12.276	139.07495	4-Hydroxyphenylethanol	[M+H]+	139.07536	C8H10O2	1-hydroxy-2-unsubstituted benzenoids	PMRFBLQVGJNGLU-UHFFFAOYNA-N	OC1=CC=C(C=C1)C(O)C
2393	12.28	203.03508	4-hydroxy-7H-furo[3,2-g]chromen-7-one	[M+H]+	203.035	C11H6O4	5-hydroxypsoralens	GIJHDGJRTUSBJR-UHFFFAOYSA-N	O=C1OC=2C=C3OC=CC3=C(O)C2C=C1
2394	12.301	377.31863	Polanrazine B_120170	[M+H]+	377.32001	C18H23N3O2S2	Alpha amino acids and derivatives	JXDWMFMAEDSXNI-QZTJIDSGSA-N	O=C1NC(SC)(C(=O)NC1(SC)CC2=CNC=3C=CC=CC32)C(C)C
2395	12.316	451.31876	(1S,2R,5R,6R,10R,13S,15S)-5-[(2R,3E,5R)-5,6-dimethylhept-3-en-2-yl]-6,10-dimethyl-16,17-dioxapentacyclo[13.2.2.0?,?.0?,?.0??,??]nonadec-18-en-13-ol	[M+H]+	451.31824	C28H44O3	Ergostane steroids	VXOZCESVZIRHCJ-LNSUSAKYSA-N	OC1CCC2(C)C3CCC4(C)C(CCC4C53OOC2(C=C5)C1)C(C=CC(C)C(C)C)C
2396	12.33	217.15816	(5E)-6,10-dimethyl-5,9-undecadien-2-one	[M+H]+	217.16	C13H22O	Acyclic monoterpenoids	HNZUNIKWNYHEJJ-FMIVXFBMSA-N	O=C(C)CCC=C(C)CCC=C(C)C
2397	12.336	365.31436	DOCOSANOL	[M+H]+	365.31799	C22H46O	Fatty alcohols	NOPFSRXAKWQILS-UHFFFAOYSA-N	OCCCCCCCCCCCCCCCCCCCCCC
2398	12.4	393.23969	monolinolein	[M+H]+	393.24014	C21H38O4	Lineolic acids and derivatives	WECGLUPZRHILCT-HZJYTTRNSA-N	O=C(OCC(O)CO)CCCCCCCC=CCC=CCCCCC
2399	12.402	441.28305	methyl 8-hydroxy-4,5,7,10,14,14-hexamethyl-6,17-dioxo-16-oxapentacyclo[13.2.2.0?,??.0?,??.0?,?]nonadeca-3,7-diene-9-carboxylate	[M-H]-	441.28088	C26H34O6	Naphthopyrans	LISLGICORSJAKO-UHFFFAOYSA-N	O=C(OC)C12C(O)=C(C(=O)C2(C(=CC3C45C(=O)OC(CC4)C(C)(C)C5CCC31C)C)C)C
2400	12.409	389.26645	5-Demethylnobiletin	[M+H]+	389.26886	C20H20O8	8-O-methylated flavonoids	DOFJNFPSMUCECH-UHFFFAOYSA-N	O=C1C=C(OC2=C(OC)C(OC)=C(OC)C(O)=C12)C=3C=CC(OC)=C(OC)C3
2401	12.45	537.45093	beta,beta-carotene	[M+H]+	537.45001	C40H56	Carotenes	OENHQHLEOONYIE-JLTXGRSLSA-N	C(=CC=C(C=CC=C(C=CC1=C(C)CCCC1(C)C)C)C)C=C(C=CC=C(C=CC2=C(C)CCCC2(C)C)C)C
2402	12.454	335.18787	(2R)-5-methoxy-8,8-dimethyl-2-phenyl-2,3-dihydropyrano[2,3-h]chromen-4-one	[M-H]-	335.18427	C21H20O4	Pyranoflavonoids	ITOTUSMHIQFNHJ-MRXNPFEDSA-N	O=C1C2=C(OC)C=C3OC(C=CC3=C2OC(C=4C=CC=CC4)C1)(C)C
2403	12.48	494.9671	(1R,3R,6R,8S,10R,13S,16S,17R)-8-tert-butyl-6,17-dihydroxy-16-methyl-2,4,14,19-tetraoxahexacyclo[8.7.2.0?,??.0?,?.0?,??.0??,??]nonadecane-	[M-H]-	494.96539	C22H24O13	Macrolides and analogues	CALDIMUKIDAVEP-UHFFFAOYSA-N	O=C1OC(C)C(O)C2=C(O)C(O)=C(OC)C(O)=C2C(=O)OC(C)C(O)C3=CC(O)=C(OC)C(O)=C13
2404	12.48	369.22769	TxB2	[M-H]-	369.22827	C20H34O6	Thromboxanes	XNRNNGPBEPRNAR-JQBLCGNGSA-N	CCCCC[C=H](O)\C=C\[C=H]1OC(O)C[C=H](O)[C=H]1C\C=C/CCCC(O)=O
2405	12.563	605.43982	Isoginsenoside Rh3	[M+H]+	605.44	C36H60O7	Triterpenoids	PHLXREOMFNVWOH-UJQJYVIBSA-N	OCC1OC(OC2CCC3(C)C(CCC4(C)C3CC(O)C5C(C(=CCC=C(C)C)C)CCC54C)C2(C)C)C(O)C(O)C1O
2406	12.582	349.20309	4-acetyloxy-8-(3-oxo-2-pent-2-enylcyclopenten-1-yl)octanoic acid	[M-H]-	349.20203	C20H30O5	Medium-chain fatty acids	QWDCJWDUPSTHHK-SNAWJCMRSA-N	O=C(OC(CCC(=O)O)CCCCC1=C(C(=O)CC1)CC=CCC)C

2407	12.593	427.38852	3-Epilupeol	[M+H]+	427.39343	C30H50O	Triterpenoids	MQYXUWHLBZFQQO-ISZJTHHZSA-N	OC1CCC2(C)C(CCC3(C)C2CCC4C5C(C(=C)C)CCC5(C)CCC43C)C1(C)C
2408	12.613	409.23532	beta-TOXICAROL	[M-H]-	409.23636	C23H22O7	Rotenones	MTTUVGLGBORPBI-UHFFFAOYSA-N	O=C1C2=C(OC3COC4=CC(OC)=C(OC)C=C4C13)C=C5OC(C=CC5=C2O)(C)C
2409	12.622	249.14821	2-[(2S,4aR,8aS)-2-hydroxy-4a-methyl-8-methylidene-3,4,5,6,7,8a-hexahydro-1H-naphthalen-2-yl]prop-2-enoic acid	[M-H]-	249.14961	C15H22O3	Eudesmane, isoeudesmane or cycloeudesmane	WQMBCGHXXVLQTE-CFVMTHIKSA-N	O=C(O)C(=C)C1(O)CCC2(C)CCCC(=C)C2C1
2410	12.637	277.14157	3,7-Epoxycaryophyllan-6-Ol	[M+2H]2+	277.14157	C15H26O2	Oxepanes	KSMZUKWIDPCGBS-UHFFFAOYSA-N	OC1CCC2(OC1(C)CCC3C2CC3(C)C)C
2411	12.648	499.25668	Lucidenic acid C	[M+H]+	499.26001	C27H40O7	Triterpenoids	XIMQDJNNBMWDIH-VHPIRQCPSA-N	O=C(O)CCC(C)C1CC(=O)C2(C3=C(C(=O)C(O)C12C)C4(C)CCC(O)C(C)(C)C4CC3O)C
2412	12.653	247.64191	Imatinib Mesylate	[M+2H]2+	247.63699	C30H35N7O4S	Benzanilides	YLMAHDNUQAMNNX-UHFFFAOYSA-N	O=C(NC1=CC=C(C(=C1)NC=2N=CC=C(N2)C3=CN=CC=C3)C)C4=CC=C(C=C4)CN5CCN(C)CC5.O=S(=O)(O)C
2413	12.655	462.09622	7-Methylthioheptyl glucosinolate	[M-H]-	462.09317	C15H29NO9S3	Alkylglucosinolates	SJHVRBSHKTUXLG-MFIRQCQASA-N	CSCCCCCCC\C(S[C=H]1O[C=H](CO)[C=H](O)[C=H](O)[C=H]1O)=N/OS(O)(=O)=O
2414	12.662	517.22748	Angeloylgomisin H	[M+H]+	517.22394	C28H36O8	Hydrolyzable tannins	ZSAUXCVJDYCLRS-XSIRQHFTSA-N	O=C(OC1=C(OC)C(OC)=CC2=C1C3=C(OC)C(OC)=C(OC)C=C3CC(O)(C)C(C)C2)C(=CC)C
2415	12.68	134.04462	L-Aspartic acid	[M+H]+	134.04478	C4H7NO4	Aspartic acid and derivatives	CKLJMWTZIZZHCS-REOHCLBHSA-N	C([C=H](C(=O)O)N)C(=O)O
2416	12.69	277.1413	3-hydroxy-1a,5-bis(hydroxymethyl)-5,6b-dimethyl-1,3,3a,4,6,6a-hexahydrocyclopropa[e]inden-2-one	[M+2H]2+	277.14102	C14H22O4	Cyclic alcohols and derivatives	CUAPOUHDJKWLOB-UHFFFAOYSA-N	O=C1C(O)C2CC(C)(CO)CC2C3(C)CC13CO
2417	12.696	347.22128	2-[4a-methyl-8-methylidene-4-(3-methylpentanoyloxy)-1,2,3,4,5,6,7,8a-octahydronaphthalen-2-yl]prop-2-enoic acid	[M-H]-	347.22278	C21H32O4	Eudesmane, isoeudesmane or cycloeudesmane	QVSFEOQJKPOOHP-UHFFFAOYSA-N	O=C(O)C(=C)C1CC(OC(=O)CC(C)CC)C2(C)CCCC(=C)C2C1
2418	12.698	519.32819	(2E,4E)-1-[(1R,3S,8R,10S,14S)-12-[(2E,4E)-hexa-2,4-dienoyl]-1,6,8,13-tetrahydroxy-3,7,10,14-tetramethyl-2,9-	[M+H]+	519.32812	C28H32O8	Oxepanes	VATYWOIGKMMCMM-QHQUPYHQSA-N	O=C(C=CC=CC)C1=C(O)C2(C)C3C(C(=O)C=CC=CC)=C(O)C4(C)C1C5(OC4(O)C3(OC52O)C)C
2419	12.699	499.25378	(3E,5S,7R,8R,11E,13S,15R,16R)-3,5,7,11,13,15-hexamethyl-8,16-bis(1,3-oxazol-5-ylmethyl)-1,9-dioxacyclohexadeca-3,11-diene-2,10-dione	[M+H]+	499.25061	C28H38N2O6	Macrolides and analogues	LAJRJVDLKYGLOO-FWSDNECISA-N	O=C1OC(CC=2OC=NC2)C(C)CC(C=C(C(=O)OC(CC=3OC=NC3)C(C)CC(C=C1C)C)C)C
2420	12.7	443.26395	Strophanthidin	[M+2H]2+	443.25845	C23H32O6	Cardenolides and derivatives	ODJLBQGVINUMMR-HZXDTFASSA-N	O=CC12CCC(O)CC2(O)CCC3C1CCC4(C)C(C5=CC(=O)OC5)CCC34O
2421	12.701	451.26227	Repaglinide	[M-H]-	451.26022	C27H36N2O4	Phenylpiperidines	FAEKWTJYAYMJKF-UHFFFAOYNA-N	O=C(O)C1=CC=C(C=C1OCC)CC(O)=NC(C=2C=CC=CC2N3CCCCC3)CC(C)C
2422	12.708	458.97763	Pesticide3_Hexaflumuron_C16H8Cl2F6N2O3_	[M-H]-	458.97501	C16H8Cl2F6N2O3	N-benzoyl-N'-phenylureas	RGNPBRKPHBKNKX-UHFFFAOYSA-N	O=C(NC(=O)C=1C(F)=CC=CC1F)NC2=CC(Cl)=C(OC(F)(F)C(F)F)C(Cl)=C2
2423	12.719	499.33569	Poricoic acid A	[M+H]+	499.34	C31H46O5	Triterpenoids	KVAQLXUMUVEKGR-SMFZDKLCSA-N	O=C(O)CCC1(C2=CCC3(C)C(C(O)CC3(C2=CCC1C(=C)C)C)C)C(C(=O)O)CCC(=C)C(C)C
2424	12.721	471.22241	(2R,3S,4S,5R,6R)-2-[[(2R,3R,4R,5S)-3,4-dihydroxy-5-(hydroxymethyl)oxolan-2-yl]oxymethyl]-6-[(2E)-3,7-dimethylocta-2,6-dienoxy]oxane-3,4,5-triol	[M+H]+	471.22006	C21H36O10	Terpene glycosides	AWDKYYYAAQQLEF-XRSPUIAPSA-N	OCC1OC(OCC2OC(OCC=C(C)CCC=C(C)C)C(O)C(O)C2O)C(O)C1O
2425	12.721	258.12772	Telmisartan (Micardis)	[M+H]+	258.12601	C33H30N4O2	Biphenyls and derivatives	RMMXLENWKUUMAY-UHFFFAOYSA-N	O=C(O)C=1C=CC=CC1C=2C=CC(=CC2)CN3C4=CC(=CC(=C4N=C3CCC)C)C5=NC=6C=CC=CC6N5C
2426	12.748	621.43817	Ginsenoside compound K	[M-H]-	621.43719	C36H62O8	Triterpenoids	FVIZARNDLVOMSU-UHFFFAOYNA-N	CC(C)=CCCC(C)(OC1OC(CO)C(O)C(O)C1O)C1CCC2(C)C1C(O)CC1C3(C)CCC(O)C(C)(C)C3CCC21C
2427	12.752	333.27866	(1R,2R,4aS,8aS)-1-((S)-3-hydroxy-3-methylpentyl)-2,5,5,8a-tetramethyldecahydronaphthalen-2-ol	[M+H]+	333.28	C20H38O2	Diterpenoids	VWPNQCLIQCRCCG-HHUCQEJWSA-N	OC(C)(CC)CCC1C(O)(C)CCC2C(C)(C)CCCC12C
2428	12.771	515.31677	2-hydroxy-3-{[3,4,5-trihydroxy-6-(hydroxymethyl)oxan-2-yl]oxy}propyl (9Z,12Z,15Z)-octadeca-9,12,15-trienoate	[M+H]+	515.32001	C27H46O9	Glycosylmonoacylglycerols	HUSISCNTLUEZCN-PDBXOOCHSA-N	O=C(OCC(O)COC1OC(CO)C(O)C(O)C1O)CCCCCCCC=CCC=CCC=CCC
2429	12.776	573.29968	Arenobufagin 3-hemisuberate	[M+H]+	573.29999	C32H44O9	Bufanolides and derivatives	LYCCSNYEMCXNRM-NUFOOTQESA-N	O=C1OC=C(C=C1)C2CCC3(O)C4CCC5CC(OC(=O)CCCCCCC(=O)O)CCC5(C)C4C(O)C(=O)C23C
2430	12.821	495.34952	Saikogenin D	[M+H]+	495.35001	C30H48O4	Triterpenoids	QGNVMEXLLPGQEV-UHFFFAOYSA-N	OCC12C(=C3C=CC4C5(C)CCC(O)C(C)(CO)C5CCC4(C)C3(C)CC1O)CC(C)(C)CC2
2431	12.836	592.38336	Edpetiline	[M+H]+	592.38501	C33H53NO8	Steroidal saponins	DHQFYEJMFMYGCV-UHFFFAOYSA-N	O=C1CC2C3CCC4C(CN5CC(C)CCC5C4(O)C)C3CC2C6(C)CCC(OC7OC(CO)C(O)C(O)C7O)CC16
2432	12.847	317.21088	18-HEPE	[M-H]-	317.21222	C20H30O3	Hydroxyeicosapentaenoic acids	LRWYBGFSVUBWMO-UXNZXXPINA-N	CCC(O)\C=C\C=C/C\C=C/C\C=C/C\C=C/CCCC(O)=O
2433	12.847	529.38843	pachymic acid	[M+H]+	529.39001	C33H52O5	Triterpenoids	VDYCLYGKCGVBHN-FBVYQCEESA-N	O=C(OC1CCC2(C3=C(CCC2C1(C)C)C4(C)CC(O)C(C(C(=O)O)CCC(=C)C(C)C)C4(C)CC3)C)C
2434	12.852	429.29916	Neoruscogenin	[M+H]+	429.29999	C27H40O4	Triterpenoids	ALTRINCJVPIQNK-UHFFFAOYSA-N	OC1CC2=CCC3C(CCC4(C)C3CC5OC6(OCC(=C)CC6)C(C)C54)C2(C)C(O)C1
2435	12.906	333.2211	5,9-dimethyltetracyclo[11.2.1.0?,??.0?,?]hexadecane-5,14-dicarboxylic acid	[M-H]-	333.22446	C20H30O4	Kaurane diterpenoids	HITLMPHPGUZLGI-UHFFFAOYSA-N	O=C(O)C1CC23CCC4C(C(=O)O)(C)CCCC4(C)C3CCC1C2
2436	12.91	621.43201	(2R,3R,4S,5R,6R)-2-[[7-[(2R,3R,4R,5S)-3,4-dihydroxy-5-(hydroxymethyl)oxolan-2-yl]oxy-2-ethenyl-2,4b,8,8-tetramethyl-	[M+H]+	621.4303	C31H50O10	Diterpene glycosides	LABWQTNVUKYYIP-ANNCGNFZSA-N	OCC1OC(OC2CCC3(C)C4C(=CC(C=C)(C)C(OC5OC(C)C(O)C(O)C5O)C4)CCC3C2(C)C)C(O)C1O
2437	12.957	579.28485	(2S)-5,7-dihydroxy-2-(4-hydroxyphenyl)-6-[(2S,3R,4R,5S,6R)-3,4,5-trihydroxy-6-[[(2R,3R,4R,5R,6S)-3,4,5-trihydroxy-6-methyloxan-2-	[M-H]-	579.28961	C27H32O14	Flavonoid C-glycosides	HFSJWNXFQYKQNX-GTTIYPEBSA-N	O=C1C2=C(O)C(=C(O)C=C2OC(C3=CC=C(O)C=C3)C1)C4OC(COC5OC(C)C(O)C(O)C5O)C(O)C(O)C4O
2438	12.96	392.26859	10'-Apozeaxanthinal	[M+H]+	392.271	C27H36O2	Sesterterpenoids	HQPQTQQUHPFUOA-ZCPYHVQDSA-N	C\C(\C=C\C=O)=C/C=C/C=C(\C)/C=C/C=C(\C)/C=C/C1=C(C)C[C=H](O)CC1(C)C
2439	12.963	632.38892	Cevadine	[M+H]+	632.38635	C32H49NO9	Cerveratrum-type alkaloids	DBUCFOVFALNEOO-HWBIYQLFSA-N	O=C(OC1CCC2(C)C3CCC4C5(O)CC(O)C6(O)C(CN7CC(C)CCC7C6(O)C)C5(O)CC42OC13O)C(=CC)C
2440	12.985	557.44965	(4R)-5',6a,8a,9-tetramethyldocosahydrospiro[naphtho[2',1':4,5]indeno[2,1-b]furan-10,2'-pyran]-4-yl nonanoate	[M+H]+	557.45001	C36H60O4	Triterpenoids	BVMVRPVYRJQROP-XYUNULTHSA-N	O=C(OC1CCC2(C)C(CCC3C2CCC4(C)C3CC5OC6(OCC(C)CC6)C(C)C54)C1)CCCCCCCC
2441	13.004	489.35828	Bayogenin	[M+H]+	489.358	C30H48O5	Triterpenoids	RWNHLTKFBKYDOJ-JEERONPWSA-N	O=C(O)C12CCC(C)(C)CC2C3=CCC4C5(C)CC(O)C(O)C(C)(CO)C5CCC4(C)C3(C)CC1
2442	13.016	565.33295	Teraspiridole C_130091	[M+H]+	565.33002	C33H44N2O6	Naphthofurans	ZLBRGMMCUDDXLB-UHFFFAOYSA-N	O=C(OC1CC2C(OC(=O)CCC2(C)C3CCC4(OC5(C=6C=CC=CC6N7C(=O)C(NC75)C)CC4C13C)C)(C)C)C
2443	13.054	455.27646	Lobaric Acid	[M-H]-	455.28067	C25H28O8	Depsides and depsidones	JHEWMLHQNRHTQX-UHFFFAOYSA-N	O=C(O)C1=C(O)C=C2OC(=O)C=3C(OC2=C1CCCCC)=CC(OC)=CC3C(=O)CCCC
2444	13.073	279.13977	Huperzine B	[M+2H]2+	279.14001	C16H20N2O	Phenanthrolines	YYWGABLTRMRUIT-HWWQOWPSSA-N	O=C1C=CC2=C(N1)CC3C=C(C)CC42NCCCC34
2445	13.082	403.23343	[2-[2-(2-methylpropanoyloxy)propan-2-yl]-7-oxo-2,3-dihydrofuro[3,2-g]chromen-3-yl] 2-methylpropanoate	[M+H]+	403.23438	C22H26O7	Psoralens	TZCDAGMXXYRIKO-UHFFFAOYSA-N	O=C1OC=2C=C3OC(C(OC(=O)C(C)C)C3=CC2C=C1)C(OC(=O)C(C)C)(C)C
2446	13.089	425.21628	[(1S,6S,7R)-6-acetyloxy-1-(3-methylbutanoyloxy)spiro[4a,5,6,7a-tetrahydro-1H-cyclopenta[c]pyran-7,2'-oxirane]-4-yl]methyl 3-methylbutanoate	[M+H]+	425.21698	C22H32O8	Iridoids and derivatives	PHHROXLDZHUIGO-VKLNETFXSA-N	O=C(OC1CC2C(=COC(OC(=O)CC(C)C)C2C31OC3)COC(=O)CC(C)C)C
2447	13.104	304.26239	Pesticide7_Fenpropimorph_C20H33NO_Morpholine, 4-[3-[4-(1,1-dimethylethyl)phenyl]-2-methylpropyl]-2,6-dimethyl-, (2R,6S)-	[M+H]+	304.263	C20H33NO	Phenylpropanes	RYAUSSKQMZRMAI-ALOPSCKCSA-N	O1C(C)CN(CC1C)CC(C)CC2=CC=C(C=C2)C(C)(C)C
2448	13.121	563.35352	Hemslecin A	[M+H]+	563.34998	C32H50O8	Cucurbitacins	LKYNAQSYQLFTCM-GYXNDICUSA-N	O=C(OC(C)(C)CCC(=O)C(O)(C)C1C(O)CC2(C)C3CC=C4C(CC(O)C(O)C4(C)C)C3(C(=O)CC12C)C)C
2449	13.17	342.26303	LAUROYLCARNITINE	[M-H]-	342.26401	C19H37NO4	Acyl carnitines	FUJLYHJROOYKRA-QGZVFWFLSA-N	O=C([O-])CC(OC(=O)CCCCCCCCCCC)C[N+](C)(C)C

2450	13.183	293.24792	Androstane-3,17-diol	[M+H]+	293.2475	C19H32O2	Androgens and derivatives	CBMYJHIOYJEBSB-UHFFFAOYNA-N	OC1CCC2(C)C(CCC3C4CCC(O)C4(C)CCC32)C1
2451	13.197	517.30054	3,5-dihydroxy-2-(4-hydroxyphenyl)-6-(3-methylbut-2-enyl)-7-[3,4,5-trihydroxy-6-(hydroxymethyl)oxan-2-yl]oxy-2,3-dihydrochromen-4-one	[M-H]-	517.3028	C26H30O11	Flavonoid-7-O-glycosides	DZBBOCDYINLWCH-UHFFFAOYSA-N	O=C1C=2C(O)=C(C(OC3OC(CO)C(O)C(O)C3O)=CC2OC(C4=CC=C(O)C=C4)C1O)CC=C(C)C
2452	13.204	468.31549	Buprenorphine	[M+H]+	468.31082	C29H41NO4	Phenanthrenes and derivatives	RMRJXGBAOAMLHD-UHFFFAOYNA-N	COC12CCC3(CC1C(C)(O)C(C)(C)C)C1CC4=C5C(OC2C35CCN1CC1CC1)=C(O)C=C4
2453	13.211	478.31293	Serine-Cholic Acid	[M+H]+	478.31601	C27H45NO7	Glycinated bile acids and derivatives	LLEBFOGPKXFKIL-UHFFFAOYSA-N	O=C(O)C(N=C(O)CCC(C)C1CCC2C3C(O)CC4CC(O)CCC4(C)C3CC(O)C12C)CO
2454	13.245	596.25555	NETILMICIN SULFATE	[M+H]+	596.2572	C21H43N5O11S	Aminocyclitol glycosides	PQHZSWZPVGHDEZ-UIQTUGNFSA-N	O=S(=O)(O)O.OC1C(OCC(O)(C)C1NC)OC2C(O)C(OC3OC(=CCC3N)CN)C(N)CC2NCC
2455	13.246	624.44574	Myxovirescin A	[M+2H]2+	624.44702	C35H61NO8	Macrolide lactams	VQWNGCSUNKJFLW-CLEPEUEQSA-N	O=C1OC(C(O)=NCC(O)CC(O)C(O)CCC(=CC=CC(CC)CCCC(=O)CCCCC(C)CC1C)COC)CCC
2456	13.273	269.2114	(Z)-3-Hydroxyhexadec-7-enoic acid (NMR)	[M-H]-	269.21222	C16H30O3	Long-chain fatty acids	CBHANIXDPMXBGZ-KTKRTIGZNA-N	CCCCCCCC\C=C/CCCC(O)CC(O)=O
2457	13.282	399.39243	24-Ethylepicoprostanol	[M+H]+	399.39853	C29H52O	Stigmastanes and derivatives	LGJMUZUPVCAVPU-RFCGSYLUSA-N	CC[C=H](CC[C=H](C)C1CC[C=H]2[C=H]3CC[C=H]4C[C=H](O)CC[C=]4(C)[C=H]3CC[C=]12C)C(C)C
2458	13.292	602.43121	5,6-Dihydroxylutein	[M+H]+	602.43298	C40H58O4	Xanthophylls	FJRIGPREPNIRNO-VQFXDPJJSA-N	C\C(\C=C\C=C(/C)\C=C\[C=H]1C(C)=C[C=H](O)CC1(C)C)=C/C=C/C=C(\C)/C=C/C=C(\C)/C=C/[C=]1(O)C(C)(C)C[C=H](O)C[C=
2459	13.323	295.1391	[(2S,3S)-2-[(E,3R,4S)-3,4-dihydroxypent-1-enyl]-6-oxo-2,3-dihydropyran-3-yl] (E)-2-methylbut-2-enoate	[M-H]-	295.14008	C15H20O6	Dihydropyranones	JTHHOHSDOJJNFN-ZXSHYABSSA-N	O=C1OC(C=CC(O)C(O)C)C(OC(=O)C(=CC)C)C=C1
2460	13.333	301.17908	Adrenosterone	[M+H]+	301.17981	C19H24O3	Androgens and derivatives	RZRPTBIGEANTGU-UHFFFAOYNA-N	O=C1C=C2CCC3C(C(=O)CC4(C(=O)CCC34)C)C2(C)CC1
2461	13.352	495.34396	Corosolic acid	[M+H]+	495.345	C30H48O4	Triterpenoids	HFGSQOYIOKBQOW-ZSDYHTTISA-N	O=C(O)C12CCC(C)C(C)C2C3=CCC4C5(C)CC(O)C(O)C(C)(C)C5CCC4(C)C3(C)CC1
2462	13.352	489.31641	Polygalic Acid	[M+H]+	489.32001	C29H44O6	12-alpha-hydroxysteroids	VZRKWGPIZJDNHC-LUNVCWBOSA-N	O=C(O)C12CCC3=C(CCC4C3(C)CCC5C(C(=O)O)(C)C(O)C(O)CC45C)C2CC(C)(C)CC1
2463	13.382	451.36157	vitamin K1	[M+H]+	451.35999	C31H46O2	Vitamin K compounds	MBWXNTAXLNYFJB-UDCSOKOMSA-N	O=C1C=2C=CC=CC2C(=O)C(=C1C)CC=C(C)CCCC(C)CCCC(C)CCCC(C)C
2464	13.399	413.26324	2-[5-[2-[2-[5-(2-hydroxybutyl)oxolan-2-yl]propanoyloxy]butyl]oxolan-2-yl]propanoic acid	[M-H]-	413.26575	C22H38O7	Dicarboxylic acids and derivatives	WGVQPPKZKAJDGR-UHFFFAOYSA-N	O=C(O)C(C)C1OC(CC1)CC(OC(=O)C(C)C2OC(CC2)CC(O)CC)CC
2465	13.399	483.28903	Gedunol	[M-H]-	483.28754	C28H36O7	Limonoids	IYGKIJUHLXBVCF-AJIQNVKKSA-N	O=C(OC1CC2C(C=CC(O)C2(C)C)(C)C3CCC4(C)C(OC(=O)C5OC54C13C)C6=COC=C6)C
2466	13.407	361.23288	3-hydroxy-6-[[(E)-3-hydroxy-2,4-dimethylhept-4-enoyl]amino]-2,4-dimethyl-5-oxohexanoic acid	[M+H]+	361.23331	C17H29NO6	Medium-chain hydroxy acids and derivatives	JNKVBUQSDAHKDQ-VQHVLOKHSA-N	O=C(O)C(C)C(O)C(C(=O)CN=C(O)C(C)C(O)C(=CCC)C)C
2467	13.43	347.29465	3-[(Z)-heptadec-10-enyl]benzene-1,2-diol	[M+H]+	347.29443	C23H38O2	Catechols	FNLMCNKPGGHKQL-FPLPWBNLSA-N	OC1=CC=CC(=C1O)CCCCCCCCCC=CCCCCCC
2468	13.433	653.38068	3-(dimethyl(3-((4R)-4-((3R,7R,12S,17R)-3,7,12-trihydroxy-10,13-dimethylhexadecahydro-1H-cyclopenta[a]phenanthren-17-	[M+H]+	653.38	C32H58N2O8S	Trihydroxy bile acids, alcohols and derivatives	GUQQBLRVXOUDTN-AKTHIIOISA-N	O=C(NCCC[N+](C)(C)CC(O)CS(=O)(=O)[O-])CCC(C)C1CCC2C3C(O)CC4CC(O)CCC4(C)C3CC(O)C12C
2469	13.49	420.3096	terpestacin	[M+H]+	420.31082	C25H38O4	Secondary alcohols	UTGBBPSEQPITLF-LJZQDFFTSA-N	O=C1C(O)=C(C(C)CO)C2CC=C(C)C(O)CCC(=CCCC(=CCC12C)C)C
2470	13.51	513.35236	culantraramine	[M+H]+	513.34998	C32H46N2O2	Aromatic monoterpenoids	ZULSSCAFEXMMQF-LYVYPOQBSA-N	O(C1=CC=C(C=C1C2C=C(C)CC(C3=CC(=CC=C3OC)CCN(C)C)C2C(=C)C)CCN(C)C)C
2471	13.522	751.34552	2-((7-acetamido-1,2,3-trimethoxy-9-oxo-5,6,7,9-tetrahydrobenzo[a]heptalen-10-yl)amino)-N-(3-(3,4-dimethoxyphenethyl)-1H-1,2,4-triazol-5-yl)-3-	[M+H]+	751.34003	C39H48N6O8	Isoleucine and derivatives	KYPPZUHEYJBGDV-UHFFFAOYSA-N	O=C1C=C2C(=CC=C1NC(C(=O)NC3=NC(=NN3)CCC4=CC=C(OC)C(OC)=C4)C(C)CC)C5=C(OC)C(OC)=C(OC)C=C5CCC2NC(=O)C
2472	13.524	635.35181	Astaxanthin	[M+H]+	635.34967	C40H52O4	Xanthophylls	MQZIGYBFDRPAKN-QISQUURKSA-N	O=C1C(=C(C=CC(=CC=CC(=CC=CC=C(C=CC=C(C=CC2=C(C(=O)C(O)CC2(C)C)C)C)C)C)C)C(C)(C)CC1O)C
2473	13.537	393.103	Sesamolin	[M+H]+	393.10001	C20H18O7	Benzodioxoles	ZZMNWJVJUKMZJY-AFHBHXEDSA-N	O1C2=CC=C(OC3OCC4C(OCC34)C5=CC=C6OCOC6=C5)C=C2OC1
2474	13.547	395.36667	Stigmasterol	[M+H]+	395.36722	C29H48O	Stigmastanes and derivatives	HCXVJBMSMIARIN-WWUFFIEYSA-N	CCC(\C=C\C(C)C1CCC2C3CC=C4C[C=H](O)CCC4(C)C3CCC12C)C(C)C
2475	13.563	411.39359	STIGMASTA-4,22-DIEN-3-ONE	[M+H]+	411.39267	C29H46O	Stigmastanes and derivatives	MKGZDUKUQPPHFM-LPJPOILFSA-N	O=C1C=C2CCC3C(CCC4(C)C(CCC34)C(C=CC(CC)C(C)C)C)C2(C)CC1
2476	13.57	399.25046	Tri(butoxyethyl) phosphate	[M+H]+	399.25061	C18H39O7P	Trialkyl phosphates	WTLBZVNBAKMVDP-UHFFFAOYSA-N	CCCCOCCOP(=O)(OCCOCCCC)OCCOCCCC
2477	13.588	398.28864	5-(4-acetyloxy-3-hydroxy-2,5,5,8a-tetramethyl-3,4,4a,6,7,8-hexahydronaphthalen-1-yl)-3-methylpentanoic acid	[M+H]+	398.29007	C22H36O5	Diterpenoids	XFCLFWWTDWKJAH-UHFFFAOYSA-N	O=C(OC1C(O)C(=C(CCC(C)CC(=O)O)C2(C)CCCC(C)(C)C12)C)C
2478	13.609	516.15112	Piperacillin	[M-H]-	516.15582	C23H27N5O7S	Peptides	IVBHGBMCVLDMKU-GXNBUGAJSA-N	CCN1CCN(C(O)=N[C=H](C(O)=N[C=H]2[C=H]3SC(C)(C)[C=H](N3C2=O)C(O)=O)C2=CC=CC=C2)C(=O)C1=O
2479	13.615	265.14206	4-epivulgarin	[M+2H]2+	265.14001	C15H20O4	Eudesmanolides, secoeudesmanolides, and	NGPDZEACIWDCKX-WCLYBPTNSA-N	O=C1OC2C(CCC3(C(=O)C=CC(O)(C)C23)C)C1C
2480	13.617	266.13867	N-Carbobenzyloxyleucine	[M+2H]2+	266.13867	C14H19NO4	Leucine and derivatives	USPFMEKVPDBMCG-UHFFFAOYNA-N	O=C(O)C(N=C(O)OCC=1C=CC=CC1)CC(C)C
2481	13.618	483.34064	Polyporenic acid C	[M+H]+	483.34	C31H46O4	Monohydroxy bile acids, alcohols and derivatives	KPKYWYZPIVAHKU-WMNQUVFJSA-N	O=C(O)C(CCC(=C)C(C)C)C1C(O)CC2(C3=CCC4C(C(=O)CCC4(C3=CCC12C)C)(C)C)C
2482	13.62	251.04628	Diphenylphosphate	[M+H]+	251.04677	C12H11O4P	Aryl phosphodiesters	ASMQGLCHMVWBQR-UHFFFAOYSA-N	C1=CC=C(C=C1)OP(O)(=O)OC2=CC=CC=C2
2483	13.627	429.29871	8-[2-(3,4-dihydroxy-2,5-dimethoxyoxolan-3-yl)ethyl]-4a,7,8-trimethylspiro[2,3,5,6,7,8a-hexahydro-1H-naphthalene-4,2'-oxirane]-2,3-	[M-H]-	429.29941	C22H38O8	O-glycosyl compounds	RLYPFWBODVDUHT-UHFFFAOYSA-N	OC1CC2C(C)(CCC3(O)C(OC)OC(OC)C3O)C(C)CCC2(C)C4(OC4)C1O
2484	13.675	548.24103	Darunavir Ethanolate (Prezista)	[M+H]+	548.242	C29H43N3O8S	Aminobenzenesulfonamides	QWSHKNICRJHQCY-VBTXLZOXSA-N	O=C(OC1COC2OCCC21)NC(CC=3C=CC=CC3)C(O)CN(CC(C)C)S(=O)(=O)C4=CC=C(N)C=C4.OCC
2485	13.704	599.31989	Oleandrin	[M+H]+	599.32001	C32H48O9	Cardenolide glycosides and derivatives	JLPDBLFIVFSOCC-XYXFTTADSA-N	O=C1OCC(=C1)C2C(OC(=O)C)CC3(O)C4CCC5CC(OC6OC(C)C(O)C(OC)C6)CCC5(C)C4CCC23C
2486	13.718	537.24811	(1R,19R,20S)-19-ethenyl-18-{[(2S,3R,4S,5S,6R)-3,4,5-trihydroxy-6-(hydroxymethyl)oxan-2-yl]oxy}-17-oxa-3,13-	[M+H]+	537.24878	C26H30N2O8	Beta carbolines	LBRPLJCNRZUXLS-YTMAOMSOSA-N	O=C1C2=COC(OC3OC(CO)C(O)C(O)C3O)C(C=C)C2CC4C=5NC=6C=CC=CC6C5CCN14
2487	13.748	495.27069	"4_,12-dideoxyphorbol-13-(2,3-dimethyl)butyrate-20-acetate"	[M+H]+	495.272	C28H40O6	Tigliane and ingenane diterpenoids	QCQACYPNQZJHQN-ZBKRNWOUSA-N	O=C(OCC1=CC2C3C(OC(=O)C(C)C(C)C)(CC(C)C2(O)C4C=C(C(=O)C4C1)C)C3(C)C)C
2488	13.752	609.26923	3-[(Z)-5-[6-hydroxy-5,5,8a-trimethyl-2-methylidene-3-[3,4,5-trihydroxy-6-(hydroxymethyl)oxan-2-yl]oxy-3,4,4a,6,7,8-hexahydro-1H-naphthalen-1-yl]-3-	[M+H]+	609.26715	C29H46O11	Diterpene glycosides	APZDDWSEXQOLKA-DHDCSXOGSA-N	O=C(O)CC(=O)OCC=C(C)CCC1C(=C)C(OC2OC(CO)C(O)C(O)C2O)CC3C(C)(C)C(O)CCC13C
2489	13.756	479.2782	Schweinfurthin F	[M+H]+	479.27899	C30H38O5	Xanthenes	BUFNPAYKUGAAAO-CMDGGOBGSA-N	OC=1C=C(C=CC=2C=C(OC)C=3OC4(C)CCC(O)C(C)(C)C4CC3C2)C=C(O)C1CC=C(C)C
2490	13.765	499.37555	Tsugaric acid A	[M+H]+	499.379	C32H50O4	Triterpenoids	FIWGZIBLJWZUEA-UHFFFAOYSA-N	O=C(OC1CCC2(C3=C(CCC2C1(C)C)C4(C)CCC(C(C(=O)O)CCC=C(C)C)C4(C)CC3)C)C
2491	13.784	445.1174	[5-[4-(3,4-dihydroxyphenyl)-2,3-dimethylbutyl]-2-hydroxyphenyl] 4-hydroxybenzoate	[M+H]+	445.11423	C25H26O6	Depsides and depsidones	ORMBYUPZHHWUEM-UHFFFAOYSA-N	O=C(OC1=CC(=CC=C1O)CC(C)C(C)CC2=CC=C(O)C(O)=C2)C3=CC=C(O)C=C3
2492	13.81	440.12564	1-(3-(3,4-dihydro-2H-benzo[b][1,4]dioxepin-7-yl)-7-hydroxy-2-methyl-4-oxo-4H-chromen-8-yl)-N,N-dimethylmethanaminium chloride	[M+H]+	440.12	C22H24ClNO5	Isoflavones	CFWGNOPKGSPMNS-UHFFFAOYSA-N	[Cl-].O=C1C2=CC=C(O)C(=C2OC(=C1C=3C=CC=4OCCCOC4C3)C)C[NH+]

2493	13.815	438.05661	(2R)-2-Hydroxy-2-phenethylglucosinolate	[M-H]-	438.05341	C15H21NO10S2	Glucosinolates	GAPDDBFHNYHZIS-UHFFFAOYNA-N	OCC1OC(SC(CC(O)C2=CC=CC=C2)=NOS(O)(=O)=O)C(O)C(O)C1O
2494	13.823	335.14978	Pyrenophorol	[M+H]+	335.14999	C16H24O6	Macrolides and analogues	RBQNDQOKFICJGL-UTBFYLPBSA-N	O=C1OC(C)CCC(O)C=CC(=O)OC(C)CCC(O)C=C1
2495	13.825	471.2706	Ingenol-5,20-acetonide-3-O-angelate	[M+H]+	471.26999	C28H38O6	Fatty acid esters	STFFIQHUWFISBB-RHKHLFRASA-N	O=C(OC1C(=CC23C(=O)C(C=C4COC(OC4C12O)(C)C)C5C(CC3C)C5(C)C)C)C(=CC)C
2496	13.831	403.26736	ascochlorin	[M-H]-	403.26532	C23H29ClO4	Hydroxybenzaldehydes	SETVRSKZJJWOPA-FLDGXQSCSA-N	O=CC1=C(O)C(=C(O)C(C1)=C1C)CC=C(C=CC2(C)C(C(=O)CCC2C)C)C
2497	13.839	277.21613	a-Linolenic acid (NMR)	[M-H]-	277.21732	C18H30O2	Lineolic acids and derivatives	DTOSIQBPPRVQHS-PDBXOOCHSA-N	CC\C=C/C\C=C/C\C=C/CCCCCCCC(O)=O
2498	13.853	751.46173	alpha-Hederin	[M+H]+	751.46271	C41H66O12	Triterpenoids	KEOITPILCOILGM-UHFFFAOYNA-N	CC1OC(OC2C(O)C(O)COC2OC2CCC3(C)C(CCC4(C)C3CC=C3C5CC(C)(C)CCC5(CCC43C)C(O)=O)C2(C)CO)C(O)C(O)C1O
2499	13.871	381.29797	2,3-dihydroxypropyl stearate	[M+H]+	381.29999	C21H42O4	1-monoacylglycerols	VBICKXHEKHSIBG-FQEVSTJZSA-N	O=C(OCC(O)CO)CCCCCCCCCCCCCCCCC
2500	13.884	280.16101	Schinifoline	[M+H]+	280.16	C17H23NO	Hydroquinolones	HCUSLQJBGQJQCB-UHFFFAOYSA-N	O=C1C=C(N(C=2C=CC=CC12)C)CCCCCCC
2501	13.896	507.27579	(2E,4E)-1-[(2R,6S,14S,22S,25R)-25-(3,3-dimethyloxiran-2-yl)-15-methyl-1,3,13,15-	[M-H]-	507.27655	C32H36N4O2	Alpha carbolines	XZFSMUXVAYCHFO-QREHLELFSA-N	O=C(C=CC=CC)N1CCC23C=4C=CC=CC4NC5N(C6=CC=CC7=C6C53CCN(C7C8OC8(C)C)C12)C
2502	13.905	417.29654	6-[[5-(hydroxymethyl)-2,5,8a-trimethyl-1,4,4a,6,7,8-hexahydronaphthalen-1-yl]methyl]-3-methylidene-7-oxabicyclo[4.1.0]heptane-2,4,5-triol	[M+H]+	417.29257	C22H34O5	Oxepanes	GVERAYRFYVBXKO-UHFFFAOYSA-N	OCC1(C)CCCC2(C)C(C(=CCC12)C)CC34OC4C(O)C(=C)C(O)C3O
2503	13.92	581.30774	Isocucurbitacin B	[M+H]+	581.30902	C32H46O8	Cucurbitacins	WTBZNVRBNJWSPF-OUKQBFOZSA-N	O=C(OC(C=CC(=O)C(O)(C)C1C(O)CC2(C)C3CC=C4C(CC(=O)C(O)C4(C)C)C3(C(=O)CC12C)C)(C)C)C
2504	13.93	542.16547	Doxorubicin	[M-H]-	542.16681	C27H29NO11	Anthracyclines	AOJJSUZBOXZQNB-UHFFFAOYNA-N	O=C1C=2C=CC=C(OC)C2C(=O)C=3C(O)=C4C(=C(O)C13)CC(O)(C(=O)CO)CC4OC5OC(C)C(O)C(N)C5
2505	13.937	691.43793	MONENSIN SODIUM (monensin A is shown)	[M+H]+	691.43915	C37H63NaO10	Ketals	GYPNBQUSNIBSGD-DUDHYVAZSA-M	[Na+].O=C([O-])C(C)C(OC)C(C)C1OC2(OC(C)(CC2)C3OC(CC)(CC3)C4OC(CC4C)
2506	13.94	278.14941	cyclo(tyrosyl-prolyl)	[M+2H]2+	278.1499	C14H16N2O3	Alpha amino acids and derivatives	LSGOTAXPWMCUCK-UHFFFAOYSA-N	O=C1N2CCCC2C(O)=NC1CC3=CC=C(O)C=C3
2507	13.943	250.02029	Clothianidin	[M+H]+	250.01601	C6H8ClN5O2S	2,5-disubstituted thiazoles	PGOOBECODWQEAB-UHFFFAOYSA-N	CN=C(NCC1=CN=C(Cl)S1)NN(=O)=O
2508	13.949	281.24762	Trans-Vaccenic acid	[M-H]-	281.2486	C18H34O2	Long-chain fatty acids	UWHZIFQPPBDJPM-BQYQJAHWSA-N	CCCCCC\C=C\CCCCCCCCCC(O)=O
2509	13.954	511.21042	Flemiphilippinin A	[M+H]+	511.20999	C30H32O6	6-prenylated isoflavanones	VBTMMYSERQFPPK-UHFFFAOYSA-N	O=C1C(=COC=2C1=C(O)C=3C=C(C(OC3C2CC=C(C)C)(C)C)C(C=C)(C)C)C=4C=CC(O)=C(O)C4
2510	13.957	577.44592	Daucosterol;Sitogluside	[M+H]+	577.44702	C35H60O6	Stigmastanes and derivatives	NPJICTMALKLTFW-OFUAXYCQSA-N	OCC1OC(OC2CC3=CCC4C(CCC5(C)C(CCC45)C(C)CCC(CC)C(C)C)C3(C)CC2)C(O)C(O)C1O
2511	13.959	529.34937	Gymnemagenin	[M+H]+	529.34998	C30H50O6	Triterpenoids	VKJLHZZPVLQJKG-ABHKXHSUSA-N	OCC1(C)C(O)CCC2(C)C3CC=C4C5CC(C)(C)C(O)C(O)C5(CO)C(O)CC4(C)C3(C)CCC12
2512	13.96	269.14359	4-benzyl-7-hydroxy-1-hydroxymethyl-3,5,6,7-tetrahydro-8h-pyrrolizinium	[M+H]+	269.14001	C15H20NO2+	Alkaloids and derivatives	MPDVSGSUYGUBLA-ARFHVFGLSA-N	OCC1=CC[N+]2(CC=3C=CC=CC3)CCC(O)C12
2513	13.97	439.30093	(2E,4E,6E,8E,10E,12E,14E,16E)-2,6,11,15-tetramethyl-17-(2,6,6-trimethyl-1-cyclohexen-1-yl)-2,4,6,8,10,12,14,16-heptadecaoctaenal	[M+H]+	439.29999	C30H40O	Triterpenoids	DFMMVLFMMAQXHZ-DOKBYWHISA-N	O=CC(=CC=CC(=CC=CC=C(C=CC=C(C=CC1=C(C)CCCC1(C)C)C)C)C)C
2514	13.971	338.27582	5-(1,2,4a,5-tetramethyl-7-oxo-3,4,8,8a-tetrahydro-2H-naphthalen-1-yl)-3-methylpentanoic acid	[M+H]+	338.26895	C20H32O3	Colensane and clerodane diterpenoids	RNWHJFUXZQBBLK-UHFFFAOYSA-N	O=C(O)CC(C)CCC1(C)C(C)CCC2(C(=CC(=O)CC21)C)C
2515	13.972	231.02675	D-(-)-quinic acid	[M+H]+	231.02655	C7H12O6	Quinic acids and derivatives	AAWZDTNXLSGCEK-LNVDRNJUSA-N	O=C(O)C1(O)CC(O)C(O)C(O)C1
2516	14.043	497.35797	Soyasapogenol A	[M+H]+	497.35999	C30H50O4	Triterpenoids	CDDWAYFUFNQLRZ-UHFFFAOYSA-N	OCC1(C)C(O)CCC2(C)C1CCC3(C)C2CC=C4C5CC(C)(C)C(O)C(O)C5(C)CCC43C
2517	14.067	595.25153	6-(furan-3-yl)-12,16,21-trihydroxy-7,15-dimethyl-9-oxo-3,17,19-trioxaheptacyclo[9.9.3.0?,??.0?,?.0?,?.0??,??.0??,??]tricosan-14-yl 2-	[M+H]+	595.25134	C31H40O10	Limonoids	QATFYHWTNMSERU-UHFFFAOYSA-N	O=C(OC1CC(O)C23C4OCC5(C(O)CC2C1(C)C(O)O4)C3C(=O)CC6(C)C(C7=COC=C7)CC8OC865)C(C)CC
2518	14.079	326.30429	N-Oleoylethanolamine	[M+H]+	326.306	C20H39NO2	N-acylethanolamines	BOWVQLFMWHZBEF-KTKRTIGZSA-N	O=C(NCCO)CCCCCCCC=CCCCCCCCC
2519	14.088	683.30658	N-((S)-10-(((2S,3R)-1-(4-(furan-2-carbonyl)piperazin-1-yl)-3-methyl-1-oxopentan-2-yl)amino)-1,2,3-trimethoxy-9-oxo-5,6,7,9-	[M+H]+	683.31	C36H44N4O8	Isoleucine and derivatives	AYNMYQSRRVGZDT-FKEGGDLNSA-N	O=C1C=C2C(=CC=C1NC(C(=O)N3CCN(C(=O)C=4OC=CC4)CC3)C(C)CC)C5=C(OC)C(OC)=C(OC)C=C5CCC2NC(=O)C
2520	14.128	551.26422	Ganoderenic acid E	[M+H]+	551.26001	C30H40O8	Triterpenoids	UFIFFDILGAASQL-HRUOLRGGSA-N	O=C(O)C(C)CC(=O)C=C(C)C1CC(=O)C2(C3=C(C(=O)C(O)C12C)C4(C)CCC(=O)C(C)(C)C4CC3O)C
2521	14.152	449.28802	[(2E,6Z,10Z)-7,11-bis(acetyloxymethyl)-3,15-dimethylhexadeca-2,6,10,14-tetraenyl] acetate	[M+H]+	449.28976	C26H40O6	Acyclic diterpenoids	KJBDTBUYUDRWMT-VDUSSSJLSA-N	O=C(OCC=C(C)CCC=C(COC(=O)C)CCC=C(COC(=O)C)CCC=C(C)C)C
2522	14.162	574.37097	2-(21-amino-3,20-dihydroxydocosan-2-yl)oxy-6-(hydroxymethyl)oxane-3,4,5-triol	[M+H]+	574.37158	C28H57NO8	Fatty acyl glycosides of mono- and disaccharides	FZSYYVOKNJZJTF-UHFFFAOYSA-N	OCC1OC(OC(C)C(O)CCCCCCCCCCCCCCCCC(O)C(N)C)C(O)C(O)C1O
2523	14.173	549.24921	[1a-(hydroxymethyl)-2-[3,4,5-trihydroxy-6-(hydroxymethyl)oxan-2-yl]oxy-2,5a,6,6a-tetrahydro-1bH-oxireno[5,6]cyclopenta[1,3-c]pyran-6-yl] 3,4-	[M+H]+	549.25061	C24H30O13	Hydrolyzable tannins	JPLOCWOFCUFHBG-UHFFFAOYSA-N	O=C(OC1C2OC2(CO)C3C(OC=CC13)OC4OC(CO)C(O)C(O)C4O)C5=CC=C(OC)C(OC)=C5
2524	14.214	341.26727	2-hydroxy-6-[(8Z,11Z)-pentadeca-8,11,14-trienyl]benzoic acid	[M-H]-	341.26553	C22H30O3	Salicylic acids	QUVGEKPNSCFQIR-UTOQUPLUSA-N	O=C(O)C=1C(O)=CC=CC1CCCCCCCC=CCC=CCC=C
2525	14.234	231.12012	Hydroxypestalotin	[M+2H]2+	231.12	C11H18O5	Dihydropyranones	YLHOHANRUSKHKO-YWVKMMECSA-N	O=C1OC(CC(OC)=C1)C(O)C(O)CCC
2526	14.256	734.45154	Phosphatidylethanolamine (16:1/18:3) Abbr: PoLnPE	[M+H]+	734.44702	C39H70NO8P	Phosphatidylethanolamines	BMBUWKNHXZNMFT-BVEGXQDJSA-N	O=C(OCC(OC(=O)CCCCCCCC=CCC=CCC=CCC)COP(=O)(O)OCCN)CCCCCCCC=CCCCCCC
2527	14.256	439.32001	tigogenin	[M+H]+	439.32001	C27H44O3	Triterpenoids	GMBQZIIUCVWOCD-UQHLGXRBSA-N	OC1CCC2(C)C(CCC3C2CCC4(C)C3CC5OC6(OCC(C)CC6)C(C)C54)C1
2528	14.275	305.24677	"(5S,8R,9S,10S,13S,14S,17S)-17-hydroxy-10,13,17-trimethyl-2,4,5,6,7,8,9,11,12,14,15,16-dodecahydro-1H-cyclopenta[a]phenanthren-3-	[M+H]+	305.24701	C20H32O2	Androgens and derivatives	WYZDXEKUWRCKOB-YDSAWKJFSA-N	O=C1CCC2(C)C(C1)CCC3C2CCC4(C)C3CCC4(O)C
2529	14.296	567.25751	3-Deacetylkhivorin	[M+H]+	567.25641	C30H40O9	Limonoids	PDKGFQJSCXMICA-GBOCIRNBSA-N	O=C(OC1CC(O)C(C)(C)C2CC(OC(=O)C)C3(C)C(CCC4(C)C(OC(=O)C)C5OC543)C6=COC=C6)C12C)C
2530	14.314	581.23737	(2R)-2-(2-((11aS)-5-(3-methoxyphenyl)-1,3-dioxo-11,11a-dihydro-1H-imidazo[1',5':1,6]pyrido[3,4-b]indol-2(3H,5H,6H)-yl)benzamido)-4-	[M+H]+	581.23999	C33H32N4O6	Leucine and derivatives	AAQMEKVMBSHQSI-KRYHRGCLSA-N	O=C(O)C(NC(=O)C=1C=CC=CC1N2C(=O)N3C(C=4C=CC=C(OC)C4)C=5NC=6C=CC=CC6C5CC3C2=O)CC(C)C
2531	14.321	315.25253	Octadecanedioic acid	[M+H]+	315.25299	C18H34O4	Long-chain fatty acids	BNJOQKFENDDGSC-UHFFFAOYSA-N	O=C(O)CCCCCCCCCCCCCCCCC(=O)O
2532	14.366	706.45544	Solanidine base + O-Hex-dHex	[M+H]+	706.45343	C39H63NO10	Steroidal saponins	ZLSYCIYRYZUJCZ-UHFFFAOYNA-N	OCC8OC(OC6CC5=CCC4C(CCC3(C)(C4(CC2N1CC(C)CCC1C(C)C23)))C5(C)CC6)C(OC7OC(C)C(O)C(O)C7(O))C(O)C8(O)
2533	14.383	251.1993	norambreinolide	[M+H]+	251.2	C16H26O2	Naphthofurans	IMKJGXCIJJXALX-SHUKQUCYSA-N	O=C1OC2(C)CCC3C(C)(C)CCCC3(C)C2C1
2534	14.404	319.22519	9-hydroxy-10,12-octadecadienoic acid	[M+H]+	319.22437	C18H32O3	Lineolic acids and derivatives	NPDSHTNEKLQQIJ-SIGMCMEVSA-N	O=C(O)CCCCCCCC(O)C=CC=CCCCCC
2535	14.412	623.24774	[(2R,3S,4S,5R,6R)-6-[1,7-bis(4-hydroxyphenyl)-5-oxoheptan-3-yl]oxy-3,4,5-trihydroxyoxan-2-yl]methyl (E)-3-(4-hydroxyphenyl)prop-2-enoate	[M+H]+	623.24866	C34H38O11	Curcuminoids	MHWAHKIDOYBXCN-FHVVLDLYSA-N	O=C(OCC1OC(OC(CC(=O)CCC2=CC=C(O)C=C2)CCC3=CC=C(O)C=C3)C(O)C(O)C1O)C=CC4=CC=C(O)C=C4

2536	14.413	421.27527	3-[(7S,8S)-5-hydroxy-2,2,7,8-tetramethyl-6-oxo-7,8-dihydropyrano[3,2-g]chromen-10-yl]-3-phenylpropanoic acid	[M-H]-	421.27563	C25H2606	Pyranochromenes	CEJSYHIZUAMEQV-CBVZESEGSA-N	0=C(0)CC(C=1C=CC=CC1)C2=C30C(C)C(C(=0)C3=C(0)C=4C=CC(0C42)(C)C)C
2537	14.437	674.461	4-((9S,10R,17S)-10-((E)-(decylimino)methyl)-3-(((2R,4S,5S,6R)-4,5-dihydroxy-6-methyltetrahydro-2H-pyran-2-yl)oxy)-5,14-dihydroxy-13-	[M+H]+	674.46002	C39H63N08	Cardenolide glycosides and derivatives	VVPXASQNOLYBCR-DYPVWMHDSA-N	0=C10CC(=C1)C2CCC3(0)C4CCC5(0)CC(0C60C(C)C(0)C(0)C6)CCC5(C=NCCCCCCCCCC)C4CCC23C
2538	14.439	553.25421	Emetine Dihydrochloride	[M+H]+	553.26001	C29H42C12N204	Emetine alkaloids	JROGBPMEKVAPEH-GXGBFOEMSA-N	C1.C1.0(C=1C=C2C(=CC10C)C(NCC2)CC3CC4C5=CC(0C)=C(0C)C=C5CCN4CC3CC)C
2539	14.487	345.2384	Darutigenol	[M+Na]+	345.23999	C20H3403	Diterpenoids	NCAZLDCEJHFJDT-KHKZNYETSA-N	0CC(0)C1(C=C2CCC3C(C)(C)C(0)CCC3(C)C2CC1)C
2540	14.515	625.26349	(2S)-4-amino-2-((2R)-2-((2S)-1-(2-(2-aminoacetamido)-4-methylpentanoyl)pyrrolidine-2-carboxamido)-3-mercaptopropanamido)-4-	[M+H]+	625.26001	C25H42N609S	Oligopeptides	UTGUKTQOTCXQJK-OZCCTXIFSA-N	0=C(0C(=0)C(NC(=0)C(NC(=0)C1N(C(=0)C(NC(=0)CN)CC(C)C)CCC1)CS)CC(=0)N)0C(C)(C)C
2541	14.529	480.30908	LPE 18:1	[M+H]+	480.30969	C23H46N07P	Lipids	PYVRVRFVLRNJLY-UHFFFAOYNA-N	0=C(0CC(0)C0P(=0)(0)0CCN)CCCCCCCC=CCCCCCCCC
2542	14.531	385.27493	Scalarin	[M+H]+	385.276	C27H4005	Scalarane sesterterpenoids	VLFJWLVMFJQJEU-HWOCEHATSA-N	0=C10C(0)C2C1=CCC3C4(C)CCC5C(C)(C)CCCC5(C)C4CC(0C(=0)C)C23C
2543	14.535	309.27832	sclareol	[M+H]+	309.27878	C20H3602	Diterpenoids	XVULBTBTFGYVRC-UHFFFAOYSA-N	0C(C=C)(C)CCC1C(0)(C)CCC2C(C)(C)CCCC12C
2544	14.547	356.25769	4-[(E)-3-hydroxy-8,10-dimethyl-2-(methylamino)dodec-6-enyl]phenol	[M+H]+	356.25598	C21H35N02	Amphetamines and derivatives	RNCWKUCWPBNWOH-SOFGYWHQSA-N	0C1=CC=C(C=C1)CC(NC)C(0)CCC=CC(C)CC(C)CC
2545	14.614	314.30475	Heptadecanoyl Ethanolamide	[M+H]+	314.31	C19H39N02	N-acylethanolamines	GCCFMSAXQJECNH-UHFFFAOYSA-N	0=C(NCC0)CCCCCCCCCCCCCCCC
2546	14.639	514.33569	2-[(3R,8aS)-3,4'-dihydroxy-4,4,7,8a-tetramethyl-6'-oxospiro[2,3,4a,5,6,7-hexahydro-1H-naphthalene-8,2'-3,8-dihydrofuro[2,3-e]isoindole]-7'-	[M-H]-	514.33154	C28H37N08	Glutamic acid and derivatives	QZGOQORSUIQVIR-UIXQMLBBSA-N	0=C(0)CCC(C(=0)0)N1C(=0)C2=CC(0)=C3C(0C4(C3)C(C)CCC5C(C)(C)C(0)CCC54C)=C2C1
2547	14.655	261.06039	4-Thiouridine	[M-H]-	261.05396	C9H12N205S	Pyrimidine nucleosides	ZLOIGESWDJYCTF-UHFFFAOYNA-N	0=C1N=C(S)C=CN1C20C(C0)C(0)C20
2548	14.668	282.28036	Oleamide	[M+H]+	282.27914	C18H35N0	Fatty amides	FATBGEAMYMYZAF-MDZDMXLPSA-N	0=C(N)CCCCCCCC=CCCCCCCCC
2549	14.687	429.33484	Ergosterol Peroxide_120246	[M+H]+	429.336	C28H4403	Ergostane steroids	VXOZCESVZIRHCJ-KGHQQZOUSA-N	0C1CCC2(C)C3CCC4(C)C(CCC4C5300C2(C=C5)C1)C(C=CC(C)C(C)C)C
2550	14.72	465.29523	Pristimerin	[M+H]+	465.29501	C30H4004	Triterpenoids	JFACETXYABVHFD-RKHSXEAASA-N	0=C1C=C2C(=CC=C3C2(C)CCC4(C)C5CC(C(=0)0C)(C)CCC5(C)CCC34C)C(=C10)C
2551	14.745	504.36578	(1R,2R,5S,8R,14R,15R,16S)-16-hydroxy-1,2,14,17,17-pentamethyl-8-(prop-1-en-2-yl)pentacyclo[11.7.0.0?,??.0?,?.0??,??]icosane-5,15-dicarboxylic acid	[M+H]+	504.36832	C30H4605	Triterpenoids	WLCHQSHZHFLMJH-GAVPBTRYSA-N	0=C(0)C1C(0)C(C)(C)C2CCC3(C)C(CCC4C5C(C(=C)C)CCC5(C(=0)0)CCC43C)C12C
2552	14.746	396.33215	Solasodiene (not validated)	[M+H]+	396.32709	C27H41N0	Steroidal saponins	OYNIUJOJEWHJPN-UHFFFAOYNA-N	04C3CC2C6CC=C1C=CCCC1(C)C6(CCC2(C)C3C(C)C45(NCC(C)CC5))
2553	14.775	399.39212	24-Ethylcoprostanol	[M+H]+	399.39853	C29H520	Stigmastanes and derivatives	LGJMUZUPVCAVPU-RMEUMSAISA-N	CC[C=H](CC[C=H](C)[C=H]1CC[C=H]2[C=H]3CC[C=H]4C[C=H](0)CC[C=]4(C)[C=H]3CC[C=]12C)C(C)C
2554	14.801	721.44489	(2S,3R)-2-(((S)-7-acetamido-1,2,3-trimethoxy-9-oxo-5,6,7,9-tetrahydrobenzo[a]heptalen-10-yl)amino)-N-(3-methoxypropyl)-3-methyl-N-	[M+H]+	721.45001	C41H60N407	Isoleucine and derivatives	ANKZXIMZBGQTDU-LUZOJMLRSA-N	0=C1C=C2C(=CC=C1NC(C(=0)N(CCC0C)CC3CCCN4CCCCC43)C(C)CC)C5=C(0C)C(0C)=C(0C)C=C5CCC2NC(=0)C
2555	14.804	430.28949	(E,2S,3R,4R,5S)-4-acetyloxy-2-amino-3,5,14-trihydroxyicos-6-enoic acid	[M-H]-	430.28845	C22H41N07	Long-chain fatty acids	OOEOVXMORBPOKC-LNBSYBDWSA-N	0=C(0)C(N)C(0)C(0C(=0)C)C(0)C=CCCCCCCC(0)CCCCCC
2556	14.81	271.20459	Dehydroepiandrosterone	[M+H]+	271.20563	C19H2802	Androgens and derivatives	FMGSKLZLMKYGDP-IHFGAHDLSA-N	CC12CCC3C(CC=C4C[C=H](0)CCC34C)C1CCC2=0
2557	14.819	639.24323	(S)-3-(4-fluorophenyl)-4-oxo-4H-chromen-7-yl 2-(6-((tert-butoxycarbonyl)amino)hexanamido)-3-phenylpropanoate	[M+H]+	639.23999	C35H37FN207	Isoflavones	SGQLWFYYEYBZNX-LJAQVGFWSA-N	0=C(0C(C)(C)C)NCCCCCC(=0)NC(C(=0)0C1=CC=C2C(=0)C(=C0C2=C1)C=3C=CC(F)=CC3)CC=4C=CC=CC4
2558	14.822	381.35129	Brassicasterol	[M+H]+	381.35159	C28H460	Ergosterols and derivatives	OILXMJHPFNGGTO-FQRXBOAXSA-N	CC(C)C(C)\C=C\C(C)C1CCC2C3CC=C4C[C=H](0)CCC4(C)C3CCC12C
2559	14.837	631.25201	6,8-bis(morpholino-4-iummethyl)-4-oxo-3-(4-(((2S,3R,4S,5S,6R)-3,4,5-trihydroxy-6-(hydroxymethyl)tetrahydro-2H-pyran-2-yl)oxy)phenyl)-4H-	[M+H]+	631.25	C31H38N2012	Isoflavonoid O-glycosides	VFUPDGRCPOEQAB-QVBFNDMOSA-N	0=C1C(=C0C2=C1C([0-])=C(C([0-])=C2C[NH+]3CC0CC3)C[NH+]4CC0CC4)C=5C=CC(0C60C(C0)C(0)
2560	14.838	373.26123	2-hydroxy-4-methoxy-3,5-bis(3-methylbut-2-enyl)-6-pentylbenzoic acid	[M-H]-	373.26044	C23H3404	P-methoxybenzoic acids and derivatives	ONMLNUSSUHTGJO-UHFFFAOYSA-N	0=C(0)C=1C(0)=C(C(0C)=C(C1CCCCC)CC=C(C)C)CC=C(C)C
2561	14.839	487.25729	1-[[(2S,3R,11bR)-3-ethyl-9,10-dimethoxy-2,3,4,6,7,11b-hexahydro-1H-benzo[a]quinolizin-2-yl]methyl]-7-methoxy-3,4-dihydro-2H-isoquinolin-6-one	[M+H]+	487.25671	C28H36N204	Isoquinolones and derivatives	CLNILJPYOXIKRO-WPSZSDGUSA-N	0=C1C=C2C(C=C10C)=C(NCC2)CC3CC4C5=CC(0C)=C(0C)C=C5CCN4CC3CC
2562	14.842	295.22678	FA 18:2+10	[M-H]-	295.2272	C18H3203	Oxidized fatty acids	HNICUWMFWZBIFP-UHFFFAOYNA-N	0=C(0)CCCCCCCC=CC=CC(0)CCCCC
2563	14.842	437.3399	Ganoderal A	[M+H]+	437.34	C30H4402	Triterpenoids	RHNFCIPJKSUUES-FLXXZUNSSA-N	0=CC(=CCCC(C)C1CCC2(C3=CCC4C(C(=0)CCC4(C3=CCC12C)C)(C)C)C)C
2564	14.845	424.15359	Domperidone	[M-H]-	424.15457	C22H24ClN502	Benzimidazoles	FGXWKSZFVQUSTL-UHFFFAOYSA-N	C1=CC=C2C(=C1)N=C(N2CCCN3CCC(CC3)N4C=5C=CC(=CC5N=C40)C1)0
2565	14.846	584.42078	Antheraxanthin	[M+H]+	584.42242	C40H5603	Xanthophylls	OFNSUWBAQRCHAV-OYQUVCAXSA-N	C\C(\C=C\C=C(/C)\C=C\C1=C(C)C[C=H](0)CC1(C)C)=C/C=C/C=C(\C)/C=C/C=C(\C)/C=C/[C=]120[C=]1(C)C[C=H](0)CC2(C)C
2566	14.847	814.59851	Phosphatidylcholine 16	[M-H]-	814.59674	C44H82N08P	Phosphatidylcholines	XWDUIGPTBYOBBP-BQYMPCJENA-N	CCCCCCCCCCCCCCCC(=0)0CC(C0P([0-])(=0)0CC[N+](C)(C)C)0C(=0)CC\C=C/C\C=C/C\C=C/CCCCCCCC
2567	14.851	793.59222	(2E,6E)-3,7,11,15,19,23,27,31,35-nonamethylhexatriaconta-2,6,34-triene-1,10,11,15,19,23,27,31-octol	[M+H]+	793.59058	C45H8608	Polyprenols	MFQDKOXWQXOOAO-YAHSWYSTSA-N	0CC=C(C)CCC=C(C)CCC(0)C(0)(C)CCCC(0)(C)CCCC(0)(C)CCCC(0)(C)CCCC(0)(C)CCCC(0)(C)CCC=C(C)C
2568	14.852	295.13544	PLATYPHYLLENONE	[M-H]-	295.13397	C19H2003	Linear diarylheptanoids	GIKJADRKBZHVCY-DUXPYHPUSA-N	0=C(C=CCCC1=CC=C(0)C=C1)CCC2=CC=C(0)C=C2
2569	14.854	184.07259	Phosphocholine	[M+H]+	184.07277	C5H15N04P	Phosphocholines	YHHSONZFOIEMCP-UHFFFAOYSA-O	0=P(0)(0)0CC[N+](C)(C)C
2570	14.855	392.29092	(9Z,12Z)-N-Benzyloctadeca-9,12-dienamide	[M+H]+	392.29001	C25H39N0	N-acyl amines	YJWLCIANOBCQGW-HZJYTTRNSA-N	0=C(NCC=1C=CC=CC1)CCCCCCCC=CCC=CCCCCC
2571	14.855	318.29959	Phytosphingosine (not validated, isomer of 1696)	[M+H]+	318.29941	C18H39N03	Lipids	AERBNCYCJBRYDG-UHFFFAOYNA-N	0CC(N)C(0)C(0)CCCCCCCCCCCCCC
2572	14.86	491.37003	(5aS,7S,11aS,12aR)-3-((2S,5R)-5-(2-hydroxypropan-2-yl)-2-methyltetrahydrofuran-2-yl)-2a,5a,8,8-	[M+H]+	491.37	C30H5005	Triterpene saponins	WENNXORDXYGDTP-NIDIBBFBSA-N	0C1CC2C3(C)CC(0)C(C4(0C(CC4)C(0)(C)C)C)C3(C)CCC52CC65CCC(0)C(C)(C)C16
2573	14.86	528.12073	DAUNORUBICIN	[M+H]+	528.12701	C27H29N010	Anthracyclines	STQGQHZAVUOBTE-VGBVRHCVSA-N	0=C1C=2C=CC=C(0C)C2C(=0)C=3C(0)=C4C(=C(0)C13)CC(0)(C(=0)C)CC40C50C(C)C(0)C(N)C5
2574	14.866	589.42218	(1S,2R,4S,9R,10R,14S,15S,17S)-9-(furan-3-yl)-1-hydroxy-15-[(1R)-1-hydroxy-2-methoxy-2-oxoethyl]-10,14,16,16-tetramethyl-7,18-dioxo-3,8-	[M+H]+	589.4231	C31H40011	Limonoids	ADDLXGZBTXNVDS-PSKLQRDRSA-N	0=C10C(C2=C0C=C2)C3(C)CCC4C5(0C5C6(0)C(=0)C4(C)C(C(0)C(=0)0C)C(C)(C)C60C(=0)C(C)C)C3C1
2575	14.866	182.00829	2-(Methylthio)benzothiazole	[M+H]+	182.00926	C8H7NS2	Benzothiazoles	UTBVIMLZIRIFFR-UHFFFAOYSA-N	CSC1=NC2=CC=CC=C2S1
2576	14.867	437.26663	4-[3-[(2E)-3,7-dimethylocta-2,6-dienyl]-2,6-dihydroxybenzoyl]-3-formyl-5-hydroxybenzoic acid	[M-H]-	437.26691	C25H2607	Benzophenones	MLSVKXQHUTYXHD-VIZOYTHASA-N	0=CC1=CC(=CC(0)=C1C(=0)C=2C(0)=CC=C(C20)CC=C(C)CCC=C(C)C)C(=0)0
2577	14.87	325.18271	Dodecylbenzenesulfonic acid	[M-H]-	325.1843	C18H3003S	Benzenesulfonic acids and derivatives	KWXICGTUELOLSQ-UHFFFAOYSA-N	CCCCCCCCCCCCC1=CC=C(C=C1)S(0)(=0)=0
2578	14.87	151.11151	Thymol	[M+H]+	151.11174	C10H140	Aromatic monoterpenoids	MGSRCZKZVOBKFT-UHFFFAOYSA-N	0C1=CC(=CC=C1C(C)C)C

2579	14.874	265.14716	Dodecyl sulfate	[M-H]-	265.1474	C12H26O4S	Sulfate containing metabolites	MOTZDAYCYVMXPC-UHFFFAOYSA-N	O=S(=O)(O)OCCCCCCCCCCCC
2580	14.876	457.34753	(4aS,6aS,6bR,10S,12aR)-10-hydroxy-2,2,6a,6b,9,9,12a-heptamethyl-1,3,4,5,6,6a,7,8,8a,10,11,12,13,14b-tetradecahydropicene-4a-carboxylic	[M+H]+	457.34576	C30H48O3	Triterpenoids	MIJYXULNPSFWEK-FQRDBSNUSA-N	O=C(O)C12CCC(C)(C)CC2C3=CCC4C5(C)CCC(O)C(C)(C)C5CCC4(C)C3(C)CC1
2581	14.878	315.23056	Progesterone	[M+H]+	315.23184	C21H30O2	Gluco/mineralocorticoids, progestogins and	RJKFOVLPORLFTN-UHFFFAOYNA-N	CC(C1CCC2C3CCC4=CC(CCC4(C)C3CCC12C)=O)=O
2582	14.879	332.25717	5-(8,11,14-pentadecatrienyl)resorcinol	[M+H]+	332.25839	C21H30O2	Resorcinols	OOXBEOHCOCMKAC-UTOQUPLUSA-N	OC=1C=C(O)C=C(C1)CCCCCCCC=CCC=CCC=C
2583	14.879	445.29095	Fusaproliferin_130137	[M+H]+	445.29501	C27H40O5	Carboxylic acid esters	VRGWBRLULZUWAJ-SXSUBTACSA-N	O=C(OCC(C1=C(O)C(=O)C2(C)CC=C(C)CCC=C(C)CCC(O)C(=CCC12)C)C)C
2584	14.894	431.26257	fucosyltransferase V	[M-H]-	431.25714	C21H20O10	Hydroxyanthraquinones	HSWIRQIYASIOBE-JNHRPPPUSA-N	O=C1C=2C=C(O)C=C(OC3OC(CO)C(O)C(O)C3O)C2C(=O)C4=C(O)C=C(C=C14)C
2585	14.901	335.22101	Difucol Hexamethyl Ether	[M+H]+	335.22144	C18H22O6	Biphenyls and derivatives	PKTVMNKLPFVXBH-UHFFFAOYSA-N	O(C1=CC(OC)=C(C(OC)=C1)C2=C(OC)C=C(OC)C=C2OC)C
2586	14.906	607.25281	Bilirubin	[M+H]+	607.25269	C33H36N4O6	Bilirubins	BPYKTIZUTYGOLE-IFADSCNNSA-N	O=C(O)CCC1=C(NC(C=C2N=C(O)C(=C2C=C)C)=C1C)CC=3NC(C=C4N=C(O)C(C=C)=C4C)=C(C3CCC(=O)O)C
2587	14.93	231.07939	Benzanthrone	[M+H]+	231.08044	C17H10O	Phenanthrenes and derivatives	HUKPVYBUJRAUAG-UHFFFAOYSA-N	C1=CC=C2C(=C1)C3=CC=CC=4C=CC=C(C43)C2=O
2588	14.934	413.3259	5-[(Z)-14-(3,5-dihydroxyphenyl)tetradec-8-enyl]benzene-1,3-diol	[M+H]+	413.32886	C26H36O4	Resorcinols	INRCFVYVWPWZJS-IWQZZHSRSA-N	OC=1C=C(O)C=C(C1)CCCCCC=CCCCCCCCC=2C=C(O)C=C(O)C2
2589	14.942	343.24689	4-[5-(4-hydroxy-3-methoxyphenyl)-3,4-dimethyloxolan-2-yl]-2-methoxyphenol	[M-H]-	343.24292	C20H24O5	7,7'-epoxylignans	GMXMKSFJQLFOSO-UHFFFAOYSA-N	OC1=CC=C(C=C1OC)C2OC(C3=CC=C(O)C(OC)=C3)C(C)C2C
2590	14.948	213.01645	2-Deoxyribose 5-phosphate	[M-H]-	213.01697	C5H11O7P	Pentose phosphates	KKZFLSZAWCYPOC-PYHARJCCSA-N	OC1C[C=H](O)[C=H](COP(O)(O)=O)O1
2591	14.95	243.02696	alpha-L-(-)-Fucose 1-phosphate bis(cyclohexylammonium) salt	[M-H]-	243.02753	C6H13O8P	Monosaccharide phosphates	PTVXQARCLQPGIR-KGJVWPDLSA-N	C[C=H]1O[C=H](OP(O)(O)=O)[C=H](O)[C=H](O)[C=H]1O
2592	14.95	245.02362	Prenyletin	[M-H]-	245.02171	C14H14O4	Hydroxycoumarins	AWEFUQDNSBBNCR-UHFFFAOYSA-N	O=C1OC=2C=C(OCC=C(C)C)C(O)=CC2C=C1
2593	14.951	455.20758	1,2Alpha-Epoxydeacetoxydihydrogedunin	[M-H]-	455.20752	C26H32O7	Limonoids	UIUKQADPLSFRBN-NIJWRPTQSA-N	O=C1OC(C2=COC=C2)C3(C)CCC4C5(C)C6OC6C(=O)C(C)(C)C5CC(O)C4(C)C73OC17
2594	14.961	467.23685	Bufotalin	[M+H]+	467.23999	C26H36O6	Bufanolides and derivatives	VOZHMAYHYHEWBW-NVOOAVKYSA-N	O=C1OC=C(C=C1)C2C(OC(=O)C)CC3(O)C4CCC5CC(O)CCC5(C)C4CCC23C
2595	14.961	429.19794	calycosin-7-O-beta-D-glucoside	[M-H]-	429.19568	C22H22O9	Isoflavonoid O-glycosides	MGJLSBDCWOSMHL-MIUGBVLSSA-N	O=C1C(=COC2=CC(OC3OC(CO)C(O)C(O)C3O)=CC=C12)C=4C=CC(OC)=CC4
2596	14.966	643.41455	Ginsenoside Rh4	[M+H]+	643.40997	C36H60O8	Triterpenoids	OZTXYFOXQFKYRP-TXRYYSRHSA-N	OCC1OC(OC2CC3(C)C(CC(O)C4C(C(=CCC=C(C)C)C)CCC43C)C5(C)CCC(O)C(C)(C)C25)C(O)C(O)C1O
2597	14.966	537.25385	Okaramine R_120153	[M+H]+	537.25201	C32H32N4O4	Alpha amino acids and derivatives	BUTLVTAFDGYVNP-UGHAWNMTSA-N	O=C1NC(C(=O)N2C=CC(C=3NC=4C=CC=CC4C3C=C12)(C)C)CC5(O)C(=O)N(C=6C=CC=CC65)C(C=C)(C)C
2598	14.973	300.28909	DErySphingosine	[M+H]+	300.2897	C18H37NO2	1,2-aminoalcohols	WWUZIQQURGPMPG-KRWOKUGFSA-N	CCCCCCCCCCCCC\C=C\[C=H](O)[C=H](N)CO
2599	14.981	236.13115	2-amino-9-methanesulfinylnonanoic acid	[M+2H]2+	236.1308	C10H21NO3S	Glucosinolate breakdown metabolites	PUZLVBQOZIVDKI-UHFFFAOYNA-N	O=C(O)C(N)CCCCCCCS(=O)C
2600	14.987	467.31229	methyl (2E,8E)-9-[3a-hydroxy-6,7-dimethyl-1-(2-methylpropyl)-3-oxo-2,4,7,7a-tetrahydro-1H-isoindol-4-yl]-4,5-dihydroxy-8-methylnona-2,8-	[M+H]+	467.31155	C25H39NO6	Isoindolones	YUXUTEAAGHFKKH-MKQCFFQWSA-N	O=C(OC)C=CC(O)C(O)CCC(=CC1C=C(C)C(C)C2C(N=C(O)C12O)CC(C)C)C
2601	15.001	703.47906	2,3,12-trihydroxy-4,6a,6b,11,12,14b-hexamethyl-8a-[3,4,5-trihydroxy-6-(hydroxymethyl)oxan-2-yl]oxycarbonyl-1,2,3,4a,5,6,7,8,9,10,11,12a,14,14a-	[M+H]+	703.47949	C36H56O12	Triterpene saponins	YXSQSVWHKZZWDD-UHFFFAOYSA-N	O=C(O)C1(C)C(O)C(O)CC2(C)C1CCC3(C)C2CC=C4C5C(O)(C)C(C)CCC5(C(=O)OC6OC(CO)C(O)C(O)C6O)CCC43C
2602	15.001	492.10486	Glucohirsutin	[M-H]-	492.10373	C16H31NO10S3	Alkylglucosinolates	GPMDJOOLATZDQL-SGBLMZTFSA-N	CS(=O)CCCCCCCC\C(S[C=H]1O[C=H](CO)[C=H](O)[C=H](O)[C=H]1O)=N/OS(O)(=O)=O
2603	15.003	685.16968	7-[(2S,3R,4S,5S,6R)-4,5-dihydroxy-3-[(2R,3R,4R,5R,6S)-3,4,5-trihydroxy-6-methyloxan-2-yl]oxy-6-[[(2R,3R,4R,5R,6S)-3,4,5-trihydroxy-6-methyloxan-2-	[M+H]+	685.17407	C28H38O17	Oligosaccharides	LLQBCHODNVGKSF-RVXDDWIJSA-N	O=C1OC=2C=C(OC3OC(COC4OC(C)C(O)C(O)C4O)C(O)C(O)C3OC5OC(C)C(O)C(O)C5O)C(OC)=CC2C=C1
2604	15.004	511.37311	Dehydroeburicoic acid monoacetate	[M+H]+	511.37	C33H50O4	Triterpenoids	AYHCPTDPDUADTK-UHFFFAOYSA-N	O=C(OC1CCC2(C3=CCC4(C)C(CCC4(C3=CCC2C1(C)C)C)C(C(=O)O)CCC(=C)C(C)C)C)C
2605	15.006	317.24826	Pregnenolone	[M+H]+	317.24799	C21H32O2	Gluco/mineralocorticoids, progestogins and	ORNBQBCIOKFOEO-SPKZCENISA-N	O=C(C)C1CCC2C3CC=C4CC(O)CCC4(C)C3CCC12C
2606	15.058	402.33209	N-(3-Methoxybenzyl)oleamide	[M+H]+	402.32999	C26H43NO2	Anisoles	ZMKZIKHBSPDWEF-KHPPLWFESA-N	O=C(NCC=1C=CC=C(OC)C1)CCCCCCCC=CCCCCCCCC
2607	15.066	119.03509	PURINE	[M-H]-	119.036	C5H4N4	Purines and purine derivatives	KDCGOANMDULRCW-UHFFFAOYSA-N	N=1C=NC=2N=CNC2C1
2608	15.073	637.42871	Ginsenoside F1	[M-H]-	637.43213	C36H62O9	Triterpenoids	XNGXWSFSJIQMNC-UHFFFAOYNA-N	CC(C)=CCCC(C)(OC1OC(CO)C(O)C(O)C1O)C1CCC2(C)C1C(O)CC1C3(C)CCC(O)C(C)(C)C3C(O)CC21C
2609	15.089	547.35889	13-Oxyingenol dodecanoat	[M+H]+	547.35999	C32H50O7	Tigliane and ingenane diterpenoids	FEZDDYPHEHMXLF-MTSAWMQWSA-N	O=C(OC12CC(C)C34C=C(C)C(O)C4(O)C(O)C(=CC(C3=O)C1C2(C)C)CO)CCCCCCCCCCC
2610	15.095	287.19968	4-Androstene-3,17-dione	[M+H]+	287.20056	C19H26O2	Androgens and derivatives	AEMFNILZOJDQLW-UHFFFAOYNA-N	CC12CCC(C=C2CCC3C4CCC(C4(C)CCC31)=O)=O
2611	15.117	354.2984	Oleoyl sarcosine	[M+H]+	354.30026	C21H39NO3	N-acyl-alpha amino acids	DIOYAVUHUXAUPX-ZHACJKMWSA-N	O=C(O)CN(C(=O)CCCCCCCC=CCCCCCCCC)C
2612	15.118	256.22589	jaeschkeanadiol	[M+H]+	256.22708	C15H26O2	Sesquiterpenoids	SUAPQGLGNKUSLY-LJISPDSOSA-N	OC1CC(=CCC2(C)CCC(O)(C(C)C)C12)C
2613	15.12	114.01618	2-Chloropyridine	[M+H]+	114.01051	C5H4ClN	2-halopyridines	OKDGRDCXVWSXDC-UHFFFAOYSA-N	ClC1=CC=CC=N1
2614	15.124	347.25494	(2R,4aR,7S,8S,8aR)-8-[(Z)-5-hydroxy-3-methylpent-3-enyl]-4,4,7,8a-tetramethyl-2,3,4a,5,6,8-hexahydro-1H-naphthalene-2,7-diol	[M+H]+	347.25565	C20H36O3	Diterpenoids	JNELTBHOLOQEJH-LPULDKDHSA-N	OCC=C(C)CCC1C(O)(C)CCC2C(C)(C)CC(O)CC12C
2615	15.127	254.21022	(4aR,5S,6S,8R,8aS)-6-hydroxy-3,8-dimethyl-5-propan-2-yl-4a,5,6,7,8,8a-hexahydro-1H-naphthalen-2-one	[M+H]+	254.21144	C15H24O2	Sesquiterpenoids	KLDSZMWMGCWFKH-GIEZYDSUSA-N	O=C1C(=CC2C(C1)C(C)CC(O)C2C(C)C)C
2616	15.198	593.27386	Pheophorbide A	[M+H]+	593.26898	C35H36N4O5	Chlorins	OINDWIFDMFYGDX-ZLNAAQIHSA-N	O=C(O)CCC1C=2N=C(C=C3NC(=CC=4NC(C=C5N=C6C(C(=O)C(C(=O)OC)C62)=C5C)=C(C4C)CC)C(C=C)=C3C)C1C
2617	15.212	507.34415	Dehydrotumulosic acid	[M+H]+	507.34	C31H48O4	Dihydroxy bile acids, alcohols and derivatives	LADJWZMBZBVBSB-YEXRKOARSA-N	O=C(O)C(CCC(=C)C(C)C)C1C(O)CC2(C3=CCC4C(C3=CCC12C)(C)CC(O)C4(C)C)C
2618	15.223	515.36755	Alisol B Acetate	[M+2H]2+	515.37	C32H50O5	Triterpenoids	NLOAQXKIIGTTRE-JSWHPQHOSA-N	O=C(OC(CC(C1=C2CC(O)C3C4(C)CCC(=O)C(C)(C)C4CCC3(C)C2(C)CC1)C)C5OC5(C)C)C
2619	15.229	249.13838	6,7-dihydroflavopereirine	[M+H]+	249.13901	C17H16N2	Quinolizines	DAGPUIPHACXCSU-UHFFFAOYSA-N	N1=C2C=CC=CC2=C3C1=C4C=CC(=CN4CC3)CC
2620	15.231	248.14099	12-Cytisineacetamide	[M+2H]2+	248.13936	C13H17N3O2	Cytisine and derivatives	MRVHCCZFXHOQOT-ZJUUUORDSA-N	OC(=N)CN1C[C=H]2C[C=H](C1)C1=CC=CC(=O)N1C2
2621	15.244	395.36502	CHOLESTANE	[M+H]+	395.36481	C27H48	Cholestane steroids	XIIAYQZJNBULGD-LDHZKLTISA-N	CC(C)CCCC(C)C1CCC2C3CCC4CCCCC4(C)C3CCC12C

2622	15.248	325.27295	(3S)-5-[(1R,2R,8aS)-2-hydroxy-2,5,5,8a-tetramethyl-3,4,4a,6,7,8-hexahydro-1H-naphthalen-1-yl]-3-methylpentanoic acid	[M+H]+	325.27371	C20H3603	Diterpenoids	KHCCSRVJJDOANA-ZOSGAXDSSA-N	O=C(O)CC(C)CCC1C(O)(C)CCC2C(C)(C)CCCC12C
2623	15.329	802.52032	Scytophycin B	[M+H]+	802.51599	C45H73NO12	Diterpene lactones	LGYQYCWMQRMLJJ-DAAXTDNESA-N	O=CN(C=CC(C)C(OC)C(C(=O)CCC(C)C(O)C(C)C1OC(=O)C=CC(=CC C(O)CC2OC(CC=C2)CC(OC)C3(OC3)C(OC)CC(OC)C1C)C)C)C
2624	15.386	372.28827	Buxtamine	[M+H]+	372.29001	C24H37NO2	Triterpenoids	KTASKRCJBXZGMH-WMYNZDBHSA-N	O=C(C)C1C(O)CC2(C)C3CCC4C(=C)C(NC)CCC54CC35CCC12C
2625	15.407	378.33545	Ursocholanic Acid	[M+H]+	378.33664	C24H4002	Bile acids, alcohols and derivatives	RPKLZQLYODPWTM-LVVAJZGHSA-N	O=C(O)CCC(C)C1CCC2C3CCC4CCCCC4(C)C3CCC12C
2626	15.418	363.28772	2-methoxy-4-pentadecylbenzoic acid	[M+H]+	363.289	C23H3803	O-methoxybenzoic acids and derivatives	HDGBHYXZLPCUJI-UHFFFAOYSA-N	O=C(O)C1=CC=C(C=C1OC)CCCCCCCCCCCCCCC
2627	15.423	397.34457	Ergosterol	[M+H]+	397.3465	C28H440	Ergosterols and derivatives	DNVPQKQSNYMLRS-UHFFFAOYNA-N	OC1CC2=CC=C3C(CCC4(C)C3CCC4C(C=CC(C)C(C)C)C)C2(C)CC1
2628	15.447	432.35431	korseveriline	[M+H]+	432.35001	C27H45NO3	Cerveratrum-type alkaloids	NQGYOUYEGZOZBN-CADHWWJYSA-N	OC1CCC2(C)C(C1)C(O)CC3C2CC4C5CN6CC(C)CCC6C(C)C5CCC430
2629	15.449	437.37778	(3beta)-stigmast-5-en-3-ol	[M+H]+	437.38	C29H500	Stigmastanes and derivatives	KZJWDPNRJALLNS-VJSFXXLFSA-N	OC1CC2=CCC3C(CCC4(C)C(CCC34)C(C)CCC(CC)C(C)C)C2(C)CC1
2630	15.45	607.25134	3-(2-((3-(2-carboxyethyl)-4-methyl-5-((Z)-(3-methyl-5-oxo-4-vinyl-1H-pyrrol-2(5H)-ylidene)methyl)-1H-pyrrol-2-yl)methyl)-4-methyl-5-((Z)-(4-	[M+H]+	607.25	C33H36N406	Bilirubins	BPYKTIZUTYGOLE-IFADSCNNSA-N	O=C(O)CCC1=C(NC(C=C2NC(=O)C(=C2C=C)C)=C1C)CC=3NC(C=C4N C(=O)C(C=C)=C4C)=C(C3CCC(=O)O)C
2631	15.491	398.34741	Solanidine (not validated)	[M+H]+	398.34091	C27H43NO	Steroidal saponins	JVKYZPBMZPJNAJ-UHFFFAOYNA-N	OC6CC5=CCC4C(CCC3(C)(C4(CC2N1CC(C)CCC1C(C)C23)))C5(C)C C6
2632	15.502	285.21609	Acacetin	[M+H]+	285.21265	C16H1205	4'-O-methylated flavonoids	DANYIYRPLHHOCZ-UHFFFAOYSA-N	O=C1C=C(OC=2C=C(O)C=C(O)C12)C=3C=CC(OC)=CC3
2633	15.541	917.53845	Notoginsenoside Ft1	[M+H]+	917.53998	C47H80017	Triterpenoids	LLXVPTXOKTYXHU-UGGLCNOCSA-N	OCC1OC(OC2CCC3(C)C(CCC4(C)C3CC(O)C5C(CCC54C)C(O)(C)CCC =C(C)C)C2(C)C)C(OC6OC(CO)C(O)C(O)C6OC7OC(CO)C(O)C7O)C(
2634	15.575	698.48297	10-[3-acetamido-4,5-dihydroxy-6-(hydroxymethyl)oxan-2-yl]oxy-5-hydroxy-2,2,6a,6b,9,9,12a-heptamethyl-1,3,4,5,6,6a,7,8,8a,10,11,12,13,14b-	[M+H]+	698.48602	C38H61NO9	Triterpene saponins	ZZVZUHRMTUSANL-UHFFFAOYSA-N	O=C(O)C12CCC(C)(C)CC2C3=CCC4C5(C)CCC(OC6OC(CO)C(O)C(O)C6N=C(O)C)C(C)(C)C5CCC4(C)C3(C)CC1O
2635	15.576	383.32977	Cholest-4,6-Dien-3-One	[M+H]+	383.33081	C27H420	Cholesterols and derivatives	XIWMRKFKSRYSIJ-GYKMGIIDSA-N	O=C1C=C2C=CC3C(CCC4(C)C3CCC4C(C)CCCC(C)C)C2(C)CC1
2636	15.58	321.21909	Bis(2-ethylhexyl)phosphate	[M-H]-	321.22003	C16H3504P	Dialkyl phosphates	SEGLCEQVOFDUPX-UHFFFAOYNA-N	CCCCC(CC)COP(O)(=O)OCC(CC)CCCC
2637	15.588	591.25671	3-[(3R,5S,10R,13R,14S,17R)-5,14-dihydroxy-10,13-dimethyl-3-[(2R,3R,4S,5S,6R)-3,4,5-trihydroxy-6-(hydroxymethyl)oxan-2-yl]oxy-	[M+H]+	591.25659	C29H44010	Cardenolide glycosides and derivatives	FECZMYDDHKVQIA-MHLHJPRMSA-N	O=C1OCC(=C1)C2CCC3(O)C4CCC5(O)CC(OC6OC(CO)C(O)C(O)C6O)CCC5(C)C4CCC23C
2638	15.589	398.23196	(3Z)-3-[[1,6-dimethyl-2-[(1E,3E)-penta-1,3-dienyl]-4a,5,6,7,8,8a-hexahydro-2H-naphthalen-1-yl]-hydroxymethylidene]-5-(1-	[M-H]-	398.23367	C24H33NO4	Pyrrolines	AIFXMSDWXBFQTF-SNXGECCISA-N	O=C1C(C(O)=NC1C(O)C)=C(O)C2(C)C(C=CC=CC)C=CC3CC(C)CCC3 2
2639	15.595	357.33517	conessine	[M+H]+	357.32999	C24H40N2	Conanine-type alkaloids	GPLGAQQQNWMVMM-CYFNKAHPSA-N	C1=C2CC(N(C)C)CCC2(C)C3CCC45CN(C)C(C)C5CCC4C3C1
2640	15.635	654.18976	7-chloro-6'-hydroxy-4,6-dimethoxy-1',3',8'-trimethyl-5'-(3,4,5-trimethoxyphenyl)-8',9'-dihydro-1'H,3H-spiro[benzofuran-2,7'-pyrimido[4,5-	[M+H]+	654.19	C32H32ClN3010	Phenylquinolines	BEAYLDNBJYAFTR-UHFFFAOYSA-N	O=C1C2=C(N=C3C(=C(O)C4(OC=5C(Cl)=C(OC)C=C(OC)C5C4=O)C(C)C3)C2C6=CC(OC)=C(OC)C(OC)=C6)N(C(=O)N1C)C
2641	15.637	653.17877	[2-[(2S,3R,4S,5S,6R)-3,4,5-trihydroxy-6-(hydroxymethyl)oxan-2-yl]oxyphenyl]methyl 6-hydroxy-2-methoxy-3-[3,4,5-trihydroxy-6-	[M+H]+	653.18365	C27H34016	Phenolic glycosides	WQQJTVODGXZMHF-XAYMNHNBSA-N	O=C(OCC=1C=CC=CC1OC2OC(CO)C(O)C(O)C2O)C=3C(O)=CC=C(OC4OC(CO)C(O)C(O)C4O)C3OC
2642	15.645	463.27945	Pristimerin derivative	[M+H]+	463.27701	C30H3804	Methyl esters	LQQBKDRWDYGCBI-RKHSXEAASA-N	O=C1C=C2C(=CC=C3C4(C=CC5(C)CCC(C(=O)OC)(C)CC5C4(C)CCC2 3C)C)C(=C1O)C
2643	15.646	479.25531	6-oxopristimerol derivative	[M+H]+	479.25	C30H3805	3-hydroxysteroids	LOTZFXULRMRODN-IUWOWYMDSA-N	O=C1C=C2C3=C(C)CC4(C)CCC(C(=O)OC)(C)CC4C3(C)CCC2(C5=CC(O)=C(O)C(=C15)C)C
2644	15.672	385.34454	Cholecalciferol	[M+H]+	385.3465	C27H440	Vitamin D and derivatives	QYSXJUFSXHHAJI-YRZJJWOYSA-N	CC(C)CCC[C=H](C)[C=H]1CC[C=H]2\C(CCC[C=]12C)=C\C=C1\C[C=H](O)CCC1=C
2645	15.706	577.242	"3_,7_-O-diacetoxy-5_-O-benzoyloxy-15_-hydroxy-17,13-oxy-14-oxopremyrsinol"	[M+H]+	577.24103	C31H3809	Bicyclic monoterpenoids	WSLFSVKKVVFSHV-VHZDBKMJSA-N	O=C(OC1C2C(OC(=O)C)C(C)CC2(O)C(=O)C3(OCC41C(OC(=O)C)CC5C(C34)C5(C)C)C)C=6C=CC=CC6
2646	15.715	645.43048	Ginsenoside Rh2	[M+H]+	645.42999	C36H6208	Triterpene saponins	CKUVNOCSBYYHIS-IRFFNABBSA-N	OCC1OC(OC2CCC3(C)C(CCC4(C)C3CC(O)C5C(CCC54C)C(O)(C)CCC=C(C)C)C2(C)C)C(O)C(O)C1O
2647	15.726	538.15131	N-[4,5-dihydroxy-2-[[4-[hydroxy(phenyl)methyl]-5-oxo-3-phenyl-2H-furan-2-yl]oxy]-6-(methoxymethyl)oxan-3-yl]acetamide	[M+H]+	538.1474	C26H29NO9	N-acyl-alpha-hexosamines	UCFGUMMLHWLLKJ-UHFFFAOYSA-N	O=C1OC(OC2OC(COC)C(O)C(O)C2N=C(O)C)C(C=3C=CC=CC3)=C1C(O)C=4C=CC=CC4
2648	15.729	338.34073	Erucamide	[M+H]+	338.34174	C22H43NO	Fatty amides	UAUDZVJPLUQNMU-UHFFFAOYSA-N	N=C(O)CCCCCCCCCCCC=CCCCCCCCC
2649	15.732	637.26135	5,6,7-triacetoxy-3-benzoyloxy-14,15-dihydroxy-9-oxojatropha-6(17),11E-diene (8)	[M+H]+	637.26202	C33H42011	Jatrophane and cyclojatrophane	IXGXYZXRENQKBA-QACYEURTSA-N	O=C(OC1C(C)CC2(O)C(O)C(C=CC(C(=O)C(OC(=O)C)C(OC(=O)C)C(=C)C(OC(=O)C)C12)(C)C)C)C=3C=CC=CC3
2650	15.741	548.23822	12-(3,4-diethoxyphenyl)-2,3-diethoxy-10-(prop-2-yn-1-yloxy)-5,6-dihydroindolo[2,1-a]isoquinoline	[M+H]+	548.23999	C33H35NO5	Phenylpyrroles	WVRAIABMMRFVCC-UHFFFAOYSA-N	C#CCOC1=CC=C2C(=C1)C(C3=CC=C(OCC)C(OCC)=C3)=C4C=5C=C(OCC)C(OCC)=CC5CCN24
2651	15.777	557.25812	(2S,3S)-8-[(2R,3S)-5,7-dihydroxy-2-(4-hydroxyphenyl)-4-oxo-2,3-dihydrochromen-3-yl]-3,5,7-trihydroxy-2-(4-hydroxyphenyl)-2,3-	[M-H]-	557.2561	C30H22011	Biflavonoids and polyflavonoids	AFDANKUHSLVEBJ-MBOHLOIDSA-N	O=C1C2=C(O)C=C(O)C(=C2OC(C3=CC=C(O)C=C3)C1O)C4C(=O)C=5C(O)=CC(O)=CC5OC4C6=CC=C(O)C=C6
2652	15.798	387.29108	(10R,13S,16R,17S)-17-acetyl-16-ethyl-10,13-dimethyl-2,3,4,7,8,9,10,11,12,13,14,15,16,17-tetradecahydro-1H-	[M+H]+	387.29001	C25H3803	Steroid esters	QBDHHZLEIBPNSS-FDGWVBKPSA-N	O=C(OC1CC2=CCC3C(CCC4(C)C(C(=O)C)C(CC)CC34)C2(C)CC1)C
2653	15.807	533.25305	ANTIMYCIN A (A1 shown)	[M-H]-	533.25043	C27H38N209	Acylaminobenzoic acid and derivatives	ZVYMCLIDNNTNCL-GFRRLMGDSA-N	O=C(OC1C(OC(=O)C(N=C(O)C=2C=CC=C(N=CO)C2O)C(OC(=O)C1CCCC)C)C)CC(C)C
2654	15.82	535.26947	Antimycin A2	[M+H]+	535.271	C27H38N209	Acylaminobenzoic acid and derivatives	LYDAGTPXPZARPR-UHFFFAOYSA-N	O=C(OC1C(OC(=O)C(N=C(O)C=2C=CC=C(N=CO)C2O)C(OC(=O)C1CCCCC)C)C)CCC
2655	15.84	379.28287	monoolein	[M+H]+	379.28189	C21H4004	1-monoacylglycerols	RZRNAYUHWVFMIP-KTKRTIGZSA-N	O=C(OCC(O)CO)CCCCCCCC=CCCCCCCCC
2656	15.848	600.41577	Auroxanthin	[M+H]+	600.4173	C40H5604	Tetraterpenoids	YLUSVJDFTAATNS-RFIAJWJGSA-N	C\C(\C=C\C=C(/C)C1O[C=]2(C)C[C=H](O)CC(C)(C)C2=C1)=C/C=C/C=C(\C)/C=C/C=C(\C)C1O[C=]2(C)C[C=H](O)CC(C)(C)C2=C
2657	15.881	669.42102	Monensin	[M-H]-	669.42194	C36H62011	Heterocyclic fatty acids	GAOZTHIDHYLHMS-UHFFFAOYNA-N	CCC1(CCC(C2(C)CCC3(CC(C(C)C(C(C)C(C(C)C(=O)O)OC)O3)O)O2)O1)C4C(C)CC(C5C(C)CC(C)C(CO)(O)O5)O4
2658	15.89	430.3299	peiminine	[M+H]+	430.32999	C27H43NO3	Cerveratrum-type alkaloids	IQDIERHFZVCNRZ-YUYPDVIUSA-N	O=C1CC2C3CCC4C(CN5CC(C)CCC5C4(O)C)C3CC2C6(C)CCC(O)CC16
2659	15.892	283.13647	5,7-dihydroxy-6-methoxy-2-phenylchromen-4-one	[M-H]-	283.1376	C16H1205	6-O-methylated flavonoids	LKOJGSWUMISDOF-UHFFFAOYSA-N	O=C1C=C(OC2=CC(O)=C(OC)C(O)=C12)C=3C=CC=CC3
2660	15.896	245.04285	2,2`,4,4`-Tetrahydroxybenzophenone	[M-H]-	245.04555	C13H1005	Benzophenones	WXNRYSGJLQFHBR-UHFFFAOYSA-N	C1=CC(=C(C=C1O)O)C(C=2C=CC(=CC2O)O)=O
2661	15.897	355.15955	rutamarin	[M-H]-	355.15509	C21H2405	Psoralens	AWMHMGFGCLBSAY-UHFFFAOYSA-N	O=C1OC=2C=C3OC(CC3=CC2C=C1C(C=C)(C)C)C(OC(=O)C)(C)C
2662	15.898	265.14694	(Dodecyloxy)sulfonic acid	[M-H]-	265.14661	C12H2604S	Others	MOTZDAYCYVMXPC-UHFFFAOYSA-N	O=S(=O)(O)OCCCCCCCCCCCC
2663	15.898	740.52325	Phosphatidylethanolamine 16	[M-H]-	740.52356	C41H76NO8P	Phosphatidylethanolamines	FOZMADPVSAYPHH-PNECARMWNA-N	CCCCCCCCCCCCCCCC(=O)OCC(COP(O)(=O)OCCN)OC(=O)CC\C=C/C\C=C/C\C=C/CCCCCCCCC
2664	15.9	271.078	1-methyl-9H-pyrido[3,4-b]indol-7-ol hydrochloride dihydrate	[M+H]+	271.07999	C12H15ClN2O3	Harmala alkaloids	UZTHVERXARKBAG-UHFFFAOYSA-N	Cl.O.O.OC=1C=CC2=C(C1)NC3=C2C=CN=C3C

2665	15.913	172.03557	Azaserine	[M-H]-	172.03638	C5H7N3O4	L-alpha-amino acids	AGNGYMCLFWQVGX-PEQLYWQKSA-N	N#[N+]C=C(O)OCC(N)C(=O)[O-]
2666	15.916	255.2318	Palmitic acid (NMR)	[M-H]-	255.23296	C16H32O2	Long-chain fatty acids	IPCSVZSSVZVIGE-UHFFFAOYSA-N	CCCCCCCCCCCCCCCC(O)=O
2667	15.917	627.42285	Ginsenoside Rh3	[M+H]+	627.42401	C36H60O7	Triterpenoids	PHLXREOMFNVWOH-YAGNRYSRSA-N	OCC1OC(OC2CCC3(C)C(CCC4(C)C3CC(O)C5C(C(=CCC=C(C)C)C)CCC54C)C2(C)C)C(O)C(O)C1O
2668	15.92	187.03702	1-Hydroxy-2-naphthoic acid	[M-H]-	187.04007	C11H8O3	Naphthalenecarboxylic acids	SJJCQDRGABAVBB-UHFFFAOYSA-N	O=C(O)C=1C=CC=2C=CC=CC2C1O
2669	15.92	313.23987	FA 18:1+2O	[M-H]-	313.23749	C18H34O4	Oxidized fatty acids	CQSLTKIXAJTQGA-UHFFFAOYNA-N	O=C(O)CCCCCCCC=CCC(O)C(O)CCCCC
2670	15.924	440.1842	FOLIC ACID	[M-H]-	440.18332	C19H19N7O6	Glutamic acid and derivatives	OVBPIULPVIDEAO-LBPRGKRZSA-N	O=C(O)CCC(NC(=O)C1=CC=C(C=C1)NCC2=NC=3C(O)=NC(=N)NC3N=C2)C(=O)O
2671	15.96	748.52954	Phosphatidylethanolamine alkenyl 16	[M-H]-	748.52869	C43H76NO7P	1-(1Z-alkenyl),2-acylglycerophosphoethano[illegible]	IRRKKHCAJLHSJE-NBLHLOSHNA-N	CCCCCCCCCCCCCC\C=C\OCC(COP(O)(=O)OCCN)OC(=O)CC\C=C/C\C=C/C\C=C/C\C=C/C\C=C/CCCCC
2672	15.971	430.87845	PFSA-Cl	[M-H]-	430.8775	C6HCl2F11O3S	PFSA	OLALPJBTHUZTEP-UHFFFAOYSA-N	O=S(=O)(O)C(F)(F)C(F)(F)C(F)(Cl)C(F)(F)C(F)(Cl)C(F)(F)F
2673	16	133.0136	(S)-MALATE	[M-H]-	133.01401	C4H6O5	Beta hydroxy acids and derivatives	BJEPYKJPYRNKOW-REOHCLBHSA-N	O=C(O)CC(O)C(=O)O
2674	16.02	547.33942	1-[2,4,6-trihydroxy-3-[7-hydroxy-2-(4-hydroxyphenyl)-3,4-dihydro-2H-chromen-4-yl]phenyl]dodecan-1-one	[M-H]-	547.34033	C33H40O7	7-hydroxyflavonoids	JGXZVDAPLSTBGZ-UHFFFAOYSA-N	O=C(C1=C(O)C=C(O)C(=C1O)C2C3=CC=C(O)C=C3OC(C4=CC=C(O)C=C4)C2)CCCCCCCCCCC
2675	16.054	769.46991	Ginsenoside F3	[M-H]-	769.47437	C41H70O13	Triterpenoids	HJRVLGWTJSLQIG-UHFFFAOYNA-N	CC(C)=CCCC(C)(OC1OC(COC2OCC(O)C(O)C2O)C(O)C(O)C1O)C1CCC2(C)C1C(O)CC1C3(C)CCC(O)C(C)(C)C3C(O)CC21C
2676	16.064	297.24451	FA 18:1+1O	[M-H]-	297.24399	C18H34O3	Oxidized fatty acids	IMYZYCNQZDBZBQ-UHFFFAOYNA-N	O=C(O)CCCCCCCC1OC1(CCCCCCCC)
2677	16.077	317.24756	5-{8(Z),11(Z)-pentadecadienyl}resorcinol	[M+H]+	317.2475	C21H32O2	Resorcinols	UFMJCOLGRWKUKO-UTOQUPLUSA-N	OC=1C=C(O)C=C(C1)CCCCCCCC=CCC=CCCC
2678	16.08	327.19388	Acitretin	[M+H]+	327.19501	C21H26O3	Retinoids	IHUNBGSDBOWDMA-AQFIFDHZSA-N	O=C(O)C=C(C=CC=C(C=CC=1C(=CC(OC)=C(C1C)C)C)C)C
2679	16.087	379.18048	methyl 4-(4,6-dihydroxy-5-methoxy-2,5-dimethyl-3-oxocyclohexen-1-yl)oxy-2-hydroxy-3,6-dimethylbenzoate	[M-H]-	379.1814	C19H24O8	o-Hydroxybenzoic acid esters	BXZJZDQDHHZIIB-UHFFFAOYSA-N	O=C(OC)C1=C(O)C(=C(OC2=C(C(=O)C(O)C(OC)(C)C2O)C)C=C1C)C
2680	16.087	383.11484	S-Adenosyl-homocysteine	[M-H]-	383.11432	C14H20N6O5S	5'-deoxy-5'-thionucleosides	ZJUKTBDSGOFHSH-UHFFFAOYNA-N	O=C(O)C(N)CCSCC1OC(N2C=NC=3C(=NC=NC32)N)C(O)C1O
2681	16.088	631.48914	Uvarigrin	[M+H]+	631.48999	C37H68O6	Annonaceous acetogenins	ADPAPZVYVYEBGS-UHFFFAOYSA-N	O=C1OC(C=C1CCCCCCCCCCCCC(O)CC(O)C2OC(CC2)C(O)CCCCCCCCCCC)C
2682	16.09	271.22717	16-Hydroxyhexadecanoic acid	[M-H]-	271.22787	C16H32O3	Long-chain fatty acids	UGAGPNKCDRTDHP-UHFFFAOYSA-N	OCCCCCCCCCCCCCCCC(O)=O
2683	16.091	165.01837	Phthalic acid	[M-H]-	165.01933	C8H6O4	Benzoic acids	XNGIFLGASWRNHJ-UHFFFAOYSA-N	O=C(O)C=1C=CC=CC1C(=O)O
2684	16.093	219.04987	PURPUROGALLIN-4-CARBOXYLIC ACID	[M-H]-	219.04782	C12H8O7	Tropolones	PJSKCCANFAFYHW-UHFFFAOYSA-N	O=C(O)C1=CC(=O)C(O)=C2C(O)=C(O)C(O)=CC2=C1
2685	16.096	191.01874	CITRATE	[M-H]-	191.019	C6H8O7	Tricarboxylic acids and derivatives	KRKNYBCHXYNGOX-UHFFFAOYSA-N	O=C(O)CC(O)(C(=O)O)CC(=O)O
2686	16.098	600.4162	Luteoxanthin	[M+H]+	600.4173	C40H56O4	Xanthophylls	YNNRPBRNWWIQPQ-MAAFUUECSA-N	C\C(\C=C\C=C(/C)\C=C\[C=]12O[C=]1(C)C[C=H](O)CC2(C)C)=C/C=C/C=C(\C)/C=C/C=C(\C)C1O[C=]2(C)C[C=H](O)CC(C)(C)C[illegible]
2687	16.103	284.29608	Stearamide	[M+H]+	284.29401	C18H37NO	Carboximidic acids	LYRFLYHAGKPMFH-UHFFFAOYSA-N	O=C(N)CCCCCCCCCCCCCCCCC
2688	16.107	281.24771	Oleic acid	[M-H]-	281.2486	C18H34O2	Long-chain fatty acids	ZQPPMHVWECSIRJ-UHFFFAOYSA-N	O=C(O)CCCCCCCC=CCCCCCCCC
2689	16.124	524.2793	Phosphatidylethanolamine lyso 22	[M-H]-	524.27826	C27H44NO7P	2-acyl-sn-glycero-3-phosphoethanolamines	TWBVHOYVCUOMJY-KUBAVDMBNA-N	CC\C=C/C\C=C/C\C=C/C\C=C/C\C=C/C\C=C/CCC(=O)OC(CO)COP(O)(=O)OCCN
2690	16.143	683.4361	Ginsenoside Rh1	[M-H]-	683.43756	C36H62O9	Triterpenoids	RAQNTCRNSXYLAH-UHFFFAOYNA-N	CC(C)=CCCC(C)(O)C1CCC2(C)C1C(O)CC1C3(C)CCC(O)C(C)(C)C3C(CC21C)OC1OC(CO)C(O)C(O)C1O
2691	16.146	281.24738	Linoleic acid	[M+H]+	281.2475	C18H32O2	Lineolic acids and derivatives	OYHQOLUKZRVURQ-HZJYTTRNSA-N	CCCCC\C=C/C\C=C/CCCCCCCC(O)=O
2692	16.161	421.32556	4-Methylabyssinone V	[M-H]-	421.32587	C26H30O5	3'-prenylated flavanones	JHEBMTRMMJXPTM-QHCPKHFHSA-N	O=C1C=2C(O)=CC(O)=CC2OC(C=3C=C(C(OC)=C(C3)CC=C(C)C)CC=C(C)C)C1
2693	16.169	623.28357	Ranaconitine	[M+H]+	623.28998	C32H44N2O9	Lappaconitine-type diterpenoid alkaloids	XTSVKUJYTUPYRJ-UHFFFAOYSA-N	O=C(OC12CN(CC)C3C4(O)CC1C3(C(OC)CC2)C5CC6C(OC)CC4(O)C5(O)C6OC)C=7C=CC=CC7NC(=O)C
2694	16.18	535.33789	1,2-Dilauroyl-sn-glycero-3-phosphate	[M-H]-	535.34052	C27H53O8P	1,2-diacylglycerol-3-phosphates	OKLASJZQBDJAPH-RUZDIDTESA-N	CCCCCCCCCCCC(=O)OC[C=H](COP(O)(O)=O)OC(=O)CCCCCCCCCCC
2695	16.244	207.01945	Methyl gallate	[M+H]+	207.02	C8H8O5	Galloyl esters	FBSFWRHWHYMIOG-UHFFFAOYSA-N	O=C(OC)C1=CC(O)=C(O)C(O)=C1
2696	16.3	503.39221	(1S,4aR,6aS,6bR,9R,10R,11R,12aR,14bS)-1,10,11-trihydroxy-9-(hydroxymethyl)-2,2,6a,6b,9,12a-hexamethyl-[illegible]	[M-H]-	503.38837	C30H48O6	Triterpenoids	IFIQVSCCFRXSJV-ZIZFEDMCSA-N	O=C(O)C12CCC3(C(=CCC4C5(C)CC(O)C(O)C(C)(CO)C5CCC43C)C2C(O)C(C)(C)CC1)C
2697	16.317	441.37103	Oleanane -4H, + 2O	[M+H]+	441.37241	C30H48O2	Triterpene saponins	VNGUCOGHCJHFID-UHFFFAOYNA-N	OCC2(C)(C(O)CCC3(C)(C4CC=C1C5CC(C=CC5(C)(CCC1(C)C4(C)(CCC23)))(C)C))
2698	16.321	367.35629	Nervonic Acid	[M+H]+	367.35001	C24H46O2	Very long-chain fatty acids	GWHCXVQVJPWHRF-KTKRTIGZSA-N	O=C(O)CCCCCCCCCCCCC=CCCCCCCCC
2699	16.324	398.33395	Palmitoylcarnitine	[M-H]-	398.32761	C23H45NO4	Acyl carnitines	XOMRRQXKHMYMOC-NRFANRHFSA-N	O=C([O-])CC(OC(=O)CCCCCCCCCCCCCCC)C[N+](C)(C)C
2700	16.346	559.89557	Hydroxylated and dihydrogenated 14-bromodiscorhabdin C (+2)	[M+H]+	559.90002	[C18H15Br3N3O3]+	Phenanthrolines	UDWHZZFAVRHFRN-UHFFFAOYSA-O	O=C1C=2NC(Br)=C3C2C(=[NH+]CC3)C4=C1NC(O)CC54C=C(Br)C(=O)C(Br)C5
2701	16.416	778.53717	Phosphatidylethanolamine 17	[M-H]-	778.53925	C44H78NO8P	Phosphatidylethanolamines	TZKBOGGOMRCSLO-RDCLIOPDNA-N	CCCCCCCCCCCCCCCCC(=O)OCC(COP(O)(=O)OCCN)OC(=O)CC\C=C/C\C=C/C\C=C/C\C=C/C\C=C/CCCCC
2702	16.42	365.30426	Colladine [M-H2O+H]+	[M+H]+	365.29999	C24H30O4	Coumarins and derivatives	FCWYNTDTQPCVPG-XLRKYUMYSA-N	O=C1OC=2C=C(OCC3C(=C)CCC4C(C)(C)C(O)CCC34C)C=CC2C=C1
2703	16.422	175.01489	Phenylphosphoric acid	[M+H]+	175.01547	C6H7O4P	Phenyl phosphates	CMPQUABWPXYYSH-UHFFFAOYSA-N	O=P(O)(O)OC=1C=CC=CC1
2704	16.435	786.57117	Phosphatidylcholine 14	[M-H]-	786.56543	C42H78NO8P	Phosphatidylcholines	BYFLSLXMNRHKEZ-YVCMCYLKNA-N	CCCCCCCCCCCCCC(=O)OCC(COP([O-])(=O)OCC[N+](C)(C)C)OC(=O)CC\C=C/C\C=C/C\C=C/CCCCCCCC[illegible]
2705	16.467	349.30911	henicosanoic acid	[M+H]+	349.31	C21H42O2	Long-chain fatty acids	CKDDRHZIAZRDBW-UHFFFAOYSA-N	O=C(O)CCCCCCCCCCCCCCCCCCCC
2706	16.514	447.28522	(3R,7R,8R,8aS)-3,4'-dihydroxy-7'-(2-hydroxyethyl)-4,4,7,8a-tetramethylspiro[2,3,4a,5,6,7-hexahydro-1H-naphthalene-8,2'-3,8-[illegible]	[M+H]+	447.28534	C25H35NO5	Isoindolones	ZHECNBLIOXZXBL-TVDJWTRXSA-N	O=C1C2=CC(O)=C3C(OC4(C3)C(C)CCC5C(C)(C)C(O)CCC54C)=C2CN1CCO
2707	16.53	653.29401	2-((7-acetamido-1,2,3-trimethoxy-9-oxo-5,6,7,9-tetrahydrobenzo[a]heptalen-10-yl)amino)-N-(3-acetamidophenyl)-3-methylpentanamide	[M+H]+	653.29999	C35H42N4O7	Isoleucine and derivatives	HPXCSOULTKVTSY-UHFFFAOYSA-N	O=C1C=C2C(=CC=C1NC(C(=O)NC=3C=CC=C(C3)NC(=O)C)C(C)CC)C4=C(OC)C(OC)=C(OC)C=C4CCC2NC(=O)C

2708	16.538	665.41907	(2S,3R,4S,5R)-2-(((4S,5aS,7S,11aR,12aS)-4,7-dihydroxy-3-((2R,5S)-5-(2-hydroxypropan-2-yl)-2-methyltetrahydrofuran-2-yl)-2a,5a,8,8-	[M+H]+	665.41998	C37H60O10	Cucurbitacin glycosides	KILZIJKCZIGLFJ-QSOLLAMVSA-N	O=C(OC1C(OCC(O)C1O)OC2CCC34CC54CCC6(C)C(C(O)CC6(C)C5CC(O)C3C2(C)C)C7(OC(CC7)C(O)(C)C)C)C
2709	16.544	120.02634	Bovinocidin (3-Nitropropionic Acid)	[M+H]+	120.02914	C3H5NO4	C-nitro compounds	WBLZUCOIBUDNBV-UHFFFAOYSA-N	O=C(O)CCN(=O)=O
2710	16.549	481.42374	Gedunin	[M-H]-	481.42529	C28H34O7	Limonoids	YJXDGWUNRYLINJ-POZBICLPSA-N	O=C(OC1CC2C(C=CC(=O)C2(C)C)(C)C3CCC4(C)C(OC(=O)C5OC54C13C)C6=COC=C6)C
2711	16.576	411.3287	gamma-Tocotrienol	[M+H]+	411.32574	C28H42O2	Tocotrienols	OTXNTMVVOOBZCV-WAZJVIJMSA-N	CC(C)=CCC\C(C)=C\CC\C(C)=C\CC[C@]1(C)CCC2=C(O1)C(C)=C(C)C(O)=C2
2712	16.623	667.44153	Ginsenoside Rh2 (S-FORM)	[M-H]-	667.44269	C36H62O8	Triterpenoids	CKUVNOCSBYYHIS-UHFFFAOYNA-N	CC(C)=CCCC(C)(O)C1CCC2(C)C1C(O)CC1C3(C)CCC(OC4OC(CO)C(O)C(O)C4O)C(C)(C)C3CCC21C
2713	16.633	551.42279	(R)-1-(4-benzylpiperazin-1-yl)-4-((3R,5R,8R,9S,10S,12S,13R,14S,17R)-3,12-dihydroxy-10,13-dimethylhexadecahydro-1H-cyclopenta[a]phenanthren-17-	[M+H]+	551.41998	C35H54N2O3	Dihydroxy bile acids, alcohols and derivatives	UTMFIKFVXBHDRL-GSUIVHTASA-N	O=C(N1CCN(CC=2C=CC=CC2)CC1)CCC(C)C3CCC4C5CCC6CC(O)CCC6(C)C5CC(O)C34C
2714	16.639	568.42535	Lutein	[M]+	568.42749	C40H56O2	Xanthophylls	KBPHJBAIARWVSC-RGZFRNHPSA-N	C\C(\C=C\C=C(/C)\C=C\[C@H]1C(C)=C[C@H](O)CC1(C)C)=C/C=C/C=C(\C)/C=C/C=C(\C)/C=C/C1=C(C)C[C@H](O)CC1(C)C
2715	16.649	249.14955	reynosin	[M+H]+	249.14999	C15H20O3	Eudesmanolides, secoeudesmanolides, and	FKBUODICGDOIGB-XOBFJNJYSA-N	O=C1OC2C(C1=C)CCC3(C)C(O)CCC(=C)C23
2716	16.654	359.31329	1-Monostearin	[M+H]+	359.31558	C21H42O4	1-monoacylglycerols	VBICKXHEKHSIBG-UHFFFAOYNA-N	O=C(OCC(O)CO)CCCCCCCCCCCCCCCCC
2717	16.659	569.42981	meso-Zeaxanthin	[M+H]+	569.42999	C40H56O2	Xanthophylls	JKQXZKUSFCKOGQ-YOPUJPICSA-N	OC1CC(=C(C=CC(=CC=CC(=CC=CC=C(C=CC=C(C=CC2=C(C)CC(O)CC2(C)C)C)C)C)C)C(C)(C)C1)C
2718	16.675	360.32504	phenylethylamide 359	[M+H]+	360.32599	C24H41NO	N-acyl amines	SBXFNAPGWOTRJF-UHFFFAOYSA-N	OC(=NCCC=1C=CC=CC1)CCCCCCCCCCCCCCC
2719	16.693	677.42322	Pseudoginsenoside RT5	[M+H]+	677.41998	C36H62O10	Triterpene saponins	PSOUXXNNRFNUAY-JLXGCTMESA-N	OCC1OC(OC2CC3(C)C(CC(O)C4C(CCC43C)C5(OC(CC5)C(O)(C)C)C)C6(C)CCC(O)C(C)(C)C26)C(O)C(O)C1O
2720	16.768	783.48492	(2S,3R,4S,5S,6R)-2-[(2R,3R,4S,5S,6R)-4,5-dihydroxy-2-[[(3S,5R,8R,10R,12R,13R,14R,17S)-12-hydroxy-17-(2-hydroxy-6-methylhept-5-	[M-H]-	783.48999	C42H72O13	Triterpene saponins	RWXIFXNRCLMQCD-XXYKJBRESA-N	OCC1OC(OC2C(O)C(O)C(OC2OC3CCC4(C)C(CCC5(C)C4CC(O)C6C(CC65C)C(O)(C)CCC=C(C)C)C3(C)C)CO)C(O)C(O)C1O
2721	16.804	422.30786	(9Z,12Z)-N-(3-Methoxybenzyl)octadeca-9,12-dienamide	[M+H]+	422.30301	C26H41NO2	Anisoles	BMQBTHWVNBJSPS-NQLNTKRDSA-N	O=C(NCC=1C=CC=C(OC)C1)CCCCCCCC=CCC=CCCCCC
2722	16.811	657.45343	(3S,10R,13R,17R)-10,13-dimethyl-17-((R)-6-methylheptan-2-yl)-2,3,4,7,8,9,10,11,12,13,14,15,16,17-tetradecahydro-1H-	[M+H]+	657.45001	C43H60O5	Cholesteryl esters	CGDPLLODVFIMRR-SWPIHIBISA-N	O=C1OC2=C(C=CC(OCC(=O)OC3CC4=CCC5C(CCC6(C)C(CCC56)C(C)CCCC(C)C)C4(C)CC3)=C2C)C7=C1CCCC7
2723	16.812	783.48621	Ginsenoside Rg2	[M-H]-	783.48999	C42H72O13	Triterpenoids	AGBCLJAHARWNLA-UHFFFAOYNA-N	CC1OC(OC2C(O)C(O)C(CO)OC2OC2CC3(C)C(CC(O)C4C(CCC34C)C(C)(O)CCC=C(C)C)C3(C)CCC(O)C(C)(C)C23)C(O)C(O)C1O
2724	16.815	633.1557	methyl (1S,4aS,7aS)-7-(hydroxymethyl)-1-[(2S,3R,4S,5S,6R)-3,4,5-trihydroxy-6-[[(E)-3-(4-hydroxy-3,5-dimethoxyphenyl)prop-2-	[M+H]+	633.15802	C28H34O14	Iridoid O-glycosides	YOFHAHMLGOFXAB-OBCDOPQVSA-N	O=C(OCC1OC(OC2OC=C(C(=O)OC)C3CC=C(CO)C23)C(O)C(O)C1O)C=CC4=CC(OC)=C(O)C(OC)=C4
2725	16.816	593.15967	methyl (4aS,5R,7S,7aS)-7-hydroxy-5-[3-(4-hydroxyphenyl)propanoyloxy]-7-methyl-1-[(2S,3R,4S,5S,6R)-3,4,5-trihydroxy-6-(hydroxymethyl)oxan-2-	[M+H]+	593.16309	C26H34O13	Iridoid O-glycosides	GODSXGJMZIRXOG-QAYRTFGPSA-N	O=C(OC)C1=COC(OC2OC(CO)C(O)C(O)C2O)C3C1C(OC(=O)CCC4=CC=C(O)C=C4)CC3(O)C
2726	16.829	611.17322	Etoposide	[M+H]+	611.17401	C29H32O13	Podophyllotoxins	VJJPUSNTGOMMGY-MRVIYFEKSA-N	O=C1OCC2C(OC3OC4COC(OC4C(O)C3O)C)C5=CC=6OCOC6C=C5C(C7=CC(OC)=C(O)C(OC)=C7)C12
2727	16.848	796.50897	Phosphatidylserine 17	[M-H]-	796.51343	C43H76NO10P	Phosphatidylserines	BPPNSMDUPJTVAF-VTJLAKARNA-N	CCCCCCCCCCCCCCCCC(=O)OCC(COP(O)(=O)OCC(N)C(O)=O)OC(=O)CC\C=C/C\C=C/C\C=C/C\C=C/CCCCCC
2728	16.88	700.49652	Soy bean phospholipid	[M+H]+	700.48999	C36H72NO8P	Phosphatidylcholines	CITHEXJVPOWHKC-UUWRZZSWSA-N	O=C(OCC(OC(=O)CCCCCCCCCCCCC)COP(=O)([O-])OCC[N+](C)(C)C)CCCCCCCCCCCCC
2729	16.885	818.52551	(2R,3R,4S,5S,6R)-2-[[(3S,6S,8R,10R,12R,13R,14R,17S)-3,12-dihydroxy-4,4,8,10,14-pentamethyl-17-[(2S)-6-methyl-2-[(2S,3R,4S,5S,6R)-3,4,5-	[M+H]+	818.526	C42H72O14	Triterpene saponins	YURJSTAIMNSZAE-ILDRPQKSSA-N	OCC1OC(OC2CC3(C)C(CC(O)C4C(CCC43C)C(OC5OC(CO)C(O)C(O)C5O)(C)CCC=C(C)C)C6(C)CCC(O)C(C)(C)C26)C(O)C(O)C1O
2730	16.913	174.12755	DEP_174.1277_10.3	[M+H]+	174.12773	C12H15N	Amphetamines and derivatives	UUFAJPMQSFXDFR-UHFFFAOYNA-N	C#CCNC(C)CC1=CC=CC=C1
2731	16.969	587.38898	Canthaxanthin (Euglenanone)	[M+H]+	587.38593	C40H52O2	Xanthophylls	FDSDTBUPSURDBL-DKLMTRRASA-N	O=C1C(=C(C=CC(=CC=CC(=CC=CC=C(C=CC=C(C=CC2=C(C(=O)CCC2(C)C)C)C)C)C)C(C)(C)CC1)C
2732	17.055	801.5506	1,2-O-Dilinoleoyl-3-O-beta-D-galactopyranosylracglycerol	[M+H]+	801.54999	C45H78O10	1,2-diacyl-3-O-beta-D-galactosyl-sn-glycerols	BROOMPUVDPTGEG-KWKASQHVSA-N	O=C(OCC(OC(=O)CCCCCCCC=CCC=CCCCCC)COC1OC(CO)C(O)C(O)C1O)CCCCCCCC=CCC=CCCCCC
2733	17.074	934.71783	(30E,34E)-2,6,10,14,18,22,26,30,34-nonamethyl-36-[(2R,3R,4S,5S,6R)-3,4,5-trihydroxy-6-(hydroxymethyl)oxan-2-yl]oxyhexatriaconta-2,30,34-triene-	[M+H]+	934.71887	C51H96O13	Polyterpenoids	MONOOAYGIBRELN-REQVTYDXSA-N	OCC1OC(OCC=C(C)CCC=C(C)CCC(O)C(O)(C)CCCC(O)(C)CCCC(O)(C)CCCC(O)(C)CCCC(O)(C)CCCC(O)(C)CCC=C(C)C)C(O)C(O)C1O
2734	17.089	709.42419	Spirostane -2H, + 1O, O-Pen-dHex	[M+H]+	709.42468	C38H60O12	Steroidal saponins	SKHJNNFXCKTDBG-UHFFFAOYNA-N	OC8CC5=CCC4C(CCC3(C)(C4(CC2OC1(OCC(C)CC1)C(C)C23)))C5(C)C(OC7OCC(O)C(O)C7(OC6OC(C)C(O)C(O)C6(O)))C8
2735	17.12	821.53479	Phosphatidylglyceride 18	[M-H]-	821.53381	C46H79O10P	Phosphatidylglycerols	SDJOKCPHTLSGSK-RRIMUHHXNA-N	CCCCCCCCCCCC\C=C/CCC(=O)OCC(COP(O)(=O)OCC(O)CO)OC(=O)CC\C=C/C\C=C/C\C=C/C\C=C/C\C=C/CCCCC
2736	17.144	586.43597	Ipomoeaxanthin C	[M+H]+	586.43805	C40H58O3	Xanthophylls	ZORQKKBTVVCHRR-FOTQGIOVSA-N	C\C(\C=C\C=C(/C)\C=C\[C@]1(O)C(C)(C)CCC[C@]1(C)O)=C/C=C/C=C(\C)/C=C/C=C(\C)C1O[C@]2(C)CCCC(C)(C)C2=C1
2737	17.152	459.38046	Soyasapogenol B	[M+H]+	459.38	C30H50O3	Triterpenoids	YOQAQNKGFOLRGT-UXXABWCISA-N	OCC1(C)C(O)CCC2(C)C3CC=C4C5CC(C)(C)CC(O)C5(C)CCC4(C)C3(C)CCC12
2738	17.178	625.41156	12-deoxy-5beta-hydroxy-phorbol-13-hexadecanoate	[M+H]+	625.40802	C36H58O6	Tigliane and ingenane diterpenoids	OVBUASHUXKNUMI-QQGRXWIWSA-N	O=C(OC12CC(C)C3C(C=C(CO)C(O)C4(O)C(=O)C(=CC34)C)C1C2(C)C)CCCCCCCCCCCCCCC
2739	17.193	721.35266	(4E)-4-[3-[4,5-dihydroxy-6-(hydroxymethyl)-3-[3,4,5-trihydroxy-6-(hydroxymethyl)oxan-2-yl]oxyoxan-2-yl]oxy-14-hydroxy-10,13-dimethyl-	[M+H]+	721.35187	C35H54O14	Steroidal glycosides	NRQCIBSGSINRCF-KNTRCKAVSA-N	O=C1OCC(=C2CCC3(O)C4CCC5CC(OC6OC(CO)C(O)C(O)C6OC7OC(CO)C(O)C(O)C7O)CCC5(C)C4CCC23C)C1
2740	17.2	719.51044	CUDA	[M+H]+	719.50439	C19H36N2O3	Medium-chain fatty acids	HPTJABJPZMULFH-UHFFFAOYSA-N	O=C(O)CCCCCCCCCCN=C(O)NC1CCCCC1
2741	17.215	167.06992	Paeonol	[M+H]+	167.07027	C9H10O3	Alkyl-phenylketones	UILPJVPSNHJFIK-UHFFFAOYSA-N	COC1=CC(O)=C(C=C1)C(C)=O
2742	17.222	343.22562	2-[(2'R,8R,8aS)-2',4,4,7,8a-pentamethylspiro[2,3,4a,5-tetrahydro-1H-naphthalene-8,5'-oxolane]-2'-yl]acetic acid	[M+H]+	343.22437	C20H32O3	Diterpenoids	XLWWERNKTLITEF-FOUFQHLJSA-N	O=C(O)CC1(OC2(C(=CCC3C(C)(C)CCCC32C)C)CC1)C
2743	17.222	207.10092	p-methoxycinnamic acid ethyl ester	[M+H]+	207.10001	C12H14O3	Cinnamic acid esters	DHNGCHLFKUPGPX-RMKNXTFCSA-N	O=C(OCC)C=CC1=CC=C(OC)C=C1
2744	17.261	483.35513	(R)-4-((3R,5R,8R,9S,10S,12S,13R,14S,17R)-3,12-dihydroxy-10,13-dimethylhexadecahydro-1H-cyclopenta[a]phenanthren-17-yl)-N-(pyridin-2-	[M+H]+	483.35001	C30H46N2O3	Dihydroxy bile acids, alcohols and derivatives	MQDDJIPAADLFAB-YAIFJCAJSA-N	O=C(NCC1=NC=CC=C1)CCC(C)C2CCC3C4CCC5CC(O)CCC5(C)C4CC(O)C23C
2745	17.321	712.49579	Phosphatidylethanolamine (16:0/16:1) Abbr: PPoPE or (14:0/18:1) Abbr: MOPE	[M+H]+	712.492	C37H72NO8P	Phosphatidylethanolamines	CZOSTDZGCCEZTJ-WMHOIYFHSA-N	O=C(OCC(OC(=O)CCCCCCCC=CCCCCCC)COP(=O)(O)OCCN)CCCCCCCCCCCCCCC
2746	17.332	493.38843	20(S),24(R)-Ocotillol	[M+H]+	493.39001	C30H52O5	Triterpene saponins	XIGBHUXQOPWGDK-IAFUCFMFSA-N	OC1CC2C3(C)CCC(O)C(C)(C)C3C(O)CC2(C)C4(C)CCC(C14)C5(OC(CC5)C(O)(C)C)C
2747	17.357	533.38629	Alisol A,24-acetate	[M+H]+	533.38	C32H52O6	Triterpenoids	WXHUQVMHWUQNTG-JSWHPQHOSA-N	O=C(OC(C(O)CC(C1=C2CC(O)C3C4(C)CCC(=O)C(C)(C)C4CCC3(C)C2(C)CC1)C)C(O)(C)C)C
2748	17.38	568.19189	ophiocordin	[M+H]+	568.19257	C28H26N2O10	Benzophenones	XYUFCXJZFZPEJD-XMSQKQJNSA-N	O=C(O)C=1C=CC=C(O)C1C(=O)C2=C(O)C=C(C=C2O)C(=O)OC3CCCNCC3NC(=O)C4=CC=C(O)C=C4
2749	17.387	491.37689	(Z)-(3S,10R,13R)-10,13-dimethyl-17-octyl-2,3,4,7,8,9,10,11,12,13,14,15,16,17-tetradecahydro-1H-	[M+H]+	491.38	C32H52O2	Steroid esters	WXCCRMLNFFAMJC-DIKJPAQWSA-N	O=C(OC1CC2=CCC3C(CCC4(C)C(CCCCCCCC)CCC34)C2(C)CC1)C(=CC)C
2750	17.387	470.19742	MK-886	[M-H]-	470.1926	C27H34ClNO2S	N-alkylindoles	QAOAOVKBIIKRNL-UHFFFAOYSA-N	O=C(O)C(C)(C)CC1=C(SC(C)(C)C)C=2C=C(C=CC2N1CC3=CC=C(Cl)C=C3)C(C)C

2751	17.406	213.01572	3:2 Fluorotelomer alcohol	[M-H]-	213.01559	C5H5F7O	PFSA	ROTSWXZIBBJEPH-UHFFFAOYSA-N	FC(F)(F)C(F)(F)C(F)(F)CCO
2752	17.408	217.04738	4-methylumbelliferyl acetate	[M-H]-	217.05063	C12H10O4	Coumarins and derivatives	HXVZGASCDAGAPS-UHFFFAOYSA-N	O=C1OC=2C=C(OC(=O)C)C=CC2C(=C1)C
2753	17.408	283.26352	Methyl Heptadecanoic acid	[M-H]-	283.26425	C18H36O2	Fatty acid methyl esters	HUEBIMLTDXKIPR-UHFFFAOYSA-N	O=C(OC)CCCCCCCCCCCCCCCC
2754	17.414	797.58899	IRGANOX 1330	[M+H]+	797.58429	C54H78O3	Linear diarylheptanoids	VSAWBBYYMBQKIK-UHFFFAOYSA-N	CC1=C(CC2=CC(=C(O)C(=C2)C(C)(C)C)C(C)(C)C)C(C)=C(CC2=C C(=C(O)C(=C2)C(C)(C)C)C(C)(C)C)C(C)=C1CC1=CC(=C(O)C(=C
2755	17.418	621.3042	"12b-O-[non-2Z,4E-dienoyl]-13_-isobutyroyloxy-7-oxo-5-ene-4b-phorbol"	[M+H]+	621.30402	C34H46O9	Phorbol esters	GOFSCWBZJWJLTF-STKJJOQCSA-N	O=C(OC1C(C)C2(O)C3C=C(C(=O)C3(O)C=C(C(=O)C2C4C1(OC(=O)C(C)C)C4(C)C)CO)C)C=CC=CCCCCC
2756	17.47	369.35422	Cholesterol	[M+H]+	369.35199	C27H46O	Cholesterols and derivatives	HVYWMOMLDIMFJA-UHFFFAOYSA-N	OC1CC2=CCC3C(CCC4(C)C(CCC34)C(C)CCCC(C)C)C2(C)CC1
2757	17.474	747.60223	Sphingomyelin d18	[M-H]-	747.60217	C40H81N2O6P	Phosphosphingolipids	YMQZQHIESOAPQH-QSTWNKESSA-N	CCCCCCCCCCCCCCCCC(O)=N[C=H](COP([O-])(=O)OCC[N+](C)(C)C)[C=H](O)\C=C\CCCCCCCCCCCCC
2758	17.532	299.11182	picein	[M+H]+	299.11252	C14H18O7	Phenolic glycosides	GOZCEKPKECLKNO-RKQHYHRCSA-N	O=C(C1=CC=C(OC2OC(CO)C(O)C(O)C2O)C=C1)C
2759	17.567	552.4317	beta-Carotene-5,6-epoxide	[M+H]+	552.43256	C40H56O	Xanthophylls	RVCRIPILOFSMFG-ASSOPURVSA-N	C\C(\C=C\C=C(/C)\C=C\C1=C(C)CCCC1(C)C)=C/C=C/C=C(\C)/C=C/C=C(\C)/C=C/[C=]12O[C=]1(C)CCCC2(C)C
2760	17.571	731.46729	Grisorixin	[M+H]+	731.46997	C40H68O10	Ketals	ZITSQIZMRMDQLE-FWKMWRKHSA-N	O=C(O)C(C)C1OC(CCC1C)CC2OC3(OC(C)(CC3C)C4OC(C)(CC4)C5OC(CC5C)C6OC(O)(C)C(C)CC6C)C(C)C(OC)C2
2761	17.59	479.36139	CRUSTECDYSONE	[M-H]-	479.36169	C27H44O7	Hydroxy bile acids, alcohols and derivatives	NKDFYOWSKOHCCO-YPVLXUMRSA-N	O=C1C=C2C(CCC3(C)C(CCC23O)C(O)(C)C(O)CCC(O)(C)C)C4(C)CC(O)C(O)CC14
2762	17.606	757.48541	(2S,3R,4R,5R,6S)-2-{[(2R,3R,4S,5S,6R)-4,5-dihydroxy-6-(hydroxymethyl)-2-{5',7,9,13-tetramethyl-5-	[M+H]+	757.4845	C39H65NO12	Steroidal saponins	RNUHPRWYDXEBOM-FXCXTKEDSA-N	OCC1OC(OC2CCC3(C)C(CCC4C3CCC5(C)C4C(O)C6OC7(NCC(C)CC7)C(C)C65)C2)C(OC8OC(C)C(O)C(O)C8O)C(O)C1O
2763	17.621	105.04296	3-Cyanopyridine	[M+H]+	105.04472	C6H4N2	3-pyridinecarbonitriles	GZPHSAQLYPIAIN-UHFFFAOYSA-N	N#CC1=CN=CC=C1
2764	17.641	104.10658	Choline	[M]+	104.10645	C5H14NO	Cholines	OEYIOHPDSNJKLS-UHFFFAOYSA-N	C[N+](C)(C)CCO
2765	17.643	515.33734	FUSIDIC ACID	[M-H]-	515.33783	C31H48O6	Steroid esters	IECPWNUMDGFDKC-MZJAQBGESA-N	O=C(O)C(=C1C(OC(=O)C)CC2(C)C1CC(O)C3C4(C)CCC(O)C(C)C4CC32C)CCC=C(C)C
2766	17.648	338.13989	cephalotaxine	[M+H]+	338.14001	C18H21NO4	Cephalotaxus alkaloids	YMNCVRSYJBNGLD-KZNAEPCWSA-N	OC1C(OC)=CC23N(CCC4=CC=5OCOC5C=C4C12)CCC3
2767	17.688	417.37399	gamma-Tocopherol	[M+H]+	417.37271	C28H48O2	Tocopherols	QUEDXNHFTDJVIY-DQCZWYHMSA-N	CC(C)CCC[C=H](C)CCC[C=H](C)CCC[C=]1(C)CCC2=C(O1)C(C)=C(C)C(O)=C2
2768	17.713	425.2222	Villosinol	[M-H]-	425.22635	C23H22O8	Rotenones	JCJPVNDLAAXNEX-UHFFFAOYSA-N	O=C1C2=C(O)C=C3OC(C(=C)C)CC3=C2OC4COC5=CC(OC)=C(OC)C=C5C14O
2769	17.746	584.42029	Loroxanthin	[M]+	584.42242	C40H56O3	Xanthophylls	ODGGKCNQKSEQNL-CWBMHJDKSA-N	C\C(\C=C\C=C(/C)\C=C\[C=H]1C(C)=C[C=H](O)CC1(C)C)=C/C=C/C=C(\C)/C=C/C=C(\CO)/C=C/C1=C(C)C[C=H](O)CC1(C)C
2770	17.751	566.40991	Diatoxanthin	[M+H]+	566.4118	C40H54O2	Triterpenoids	HNYJHQMUSVNWPV-DRCJTWAYSA-N	C\C(\C=C\C=C(/C)\C=C\C1=C(C)C[C=H](O)CC1(C)C)=C/C=C/C=C(\C)/C=C/C=C(\C)C#CC1=C(C)C[C=H](O)CC1(C)C
2771	17.794	879.74115	1-Palmitoyl-2-oleoyl-3-linoleoyl-rac-glycerol	[M+H]+	879.74121	C55H100O6	Triacylglycerols	KGLAHZTWGPHKFF-FBSASISJNA-N	CCCCCCCCCCCCCCCC(=O)OCC(COC(=O)CCCCCCC\C=C/C\C=C/CCCCC)OC(=O)CCCCCCC\C=C/CCCCCCCC
2772	17.846	907.77216	Triolein	[M+H]+	907.77252	C57H104O6	Triacylglycerols	PHYFQTYBJUILEZ-IUPFWZBJSA-N	CCCCCCCC\C=C/CCCCCCCC(=O)OCC(COC(=O)CCCCCCC\C=C/CCCCCCCC)OC(=O)CCCCCCC\C=C/CCCCCCCC
2773	17.875	663.36487	[(2R,3R,4S,5S,6R)-3-acetyloxy-2-(acetyloxymethyl)-5-butanoyloxy-6-[(2S,3R)-2,3,4-trihydroxybutoxy]oxan-4-yl] 14-hydroxytetradecanoate	[M-H]-	663.35974	C32H56O14	Saccharolipids	ORXMVTNKIKCWMQ-JBAQQFLCSA-N	O=C(OCC1OC(OCC(O)C(O)CO)C(OC(=O)CCC)C(OC(=O)CCCCCCCCCCCCCO)C1OC(=O)C)C
2774	17.876	563.30042	"12b-O-[octa-2Z,4E-dienoyl]-13_-isobutyroyloxy-4b-deoxyphorbol "	[M+H]+	563.29901	C32H44O7	Tigliane and ingenane diterpenoids	SVAPNKGLWRWWCI-GASJSDEUSA-N	O=C(OC1C(C)C2(O)C3C=C(C(=O)C3CC(=CC2C4C1(OC(=O)C(C)C)C4(C)C)CO)C)C=CC=CCCC
2775	17.877	688.16046	OLEANDOMYCIN	[M+H]+	688.16431	C35H61NO12	Aminoglycosides	RZPAKFUAFGMUPI-KGIGTXTPSA-N	O=C1OC(C)C(C)C(O)C(C(=O)C2(OC2)CC(C)C(OC3OC(C)CC(N(C)C)C3O)C(C)C(OC4OC(C)C(O)C(OC)C4)C1C)C
2776	17.887	571.4328	Podototarin	[M+2H]2+	571.42731	C40H58O2	Diterpenoids	RFUVOURUUJLQSB-QFFMKFBWSA-N	OC1=C(C=C2C(=C1C(C)C)CCC3C2(C)CCCC3(C)C)C=4C=C5C(=C(C4O)C(C)C)CCC6C5(C)CCCC6(C)C
2777	17.905	585.42694	Dereplicator Identification - Plectaniaxanthin;_1',2'-Dihydroxy-1',2'-	[M+H]+	585.42102	C40H56O3	Xanthophylls	JJPMVSRTRMLHST-BZLQNNQZSA-N	OC(C=CC(=CC=CC(=CC=CC(=CC=CC=C(C=CC=C(C=CC1=C(C)CC(O)CC1(C)C)C)C)C)C)C)C(O)(C)C
2778	17.92	544.50641	Phytoene	[M+H]+	544.50024	C40H64	Carotenes	YVLPJIGOMTXXLP-KEKOKYSKSA-N	CC(C)=CCC\C(C)=C\CC\C(C)=C\CC\C(C)=C\C=C\C=C(/C)CC\C=C(/C)CC\C=C(/C)CCC=C(C)C
2779	17.927	445.1221	[(2R,3R,4R,5R)-2-(2,4-dioxopyrimidin-1-yl)-4-hydroxy-5-(hydroxymethyl)oxolan-3-yl] 2,4-dimethoxy-6-methylbenzoate	[M+H]+	445.12173	C19H22N2O9	Pyrimidine nucleosides	DOVSRUBRIYLZCI-BASLNEPJSA-N	O=C(OC1C(O)C(OC1N2C=CC(O)=NC2=O)CO)C=3C(OC)=CC(OC)=CC3C
2780	17.928	401.34052	7-Oxocholesterol	[M+H]+	401.3414	C27H44O2	Cholesterols and derivatives	YIKKMWSQVKJCOP-ABXCMAEBSA-N	O=C1C=C2CC(O)CCC2(C)C3CCC4(C)C(CCC4C13)C(C)CCCC(C)C
2781	17.93	398.30365	N-(3-Methoxybenzyl)palmitamide	[M+H]+	398.30301	C24H41NO2	Anisoles	FIQGQTITXPTKIY-UHFFFAOYSA-N	O=C(NCC=1C=CC=C(OC)C1)CCCCCCCCCCCCCCC
2782	17.959	703.47876	((5aR,5bR,11aR)-9-(cinnamoyloxy)-5a,5b,8,8,11a-pentamethyl-1-(prop-1-en-2-yl)icosahydro-1H-cyclopenta[a]chrysen-3a-yl)methyl cinnamate	[M+H]+	703.46997	C48H62O4	Triterpenoids	OADZSFPWAGMTDV-QWRSHYLFSA-N	O=C(OCC12CCC(C(=C)C)C2C3CCC4C5(C)CCC(OC(=O)C=CC=6C=CC=CC6)C(C)(C)C5CCC4(C)C3(C)CC1)C=CC=7C=CC=CC7
2783	17.995	861.54639	Phosphatidylinositol 18	[M-H]-	861.54987	C45H83O13P	Phosphatidylinositols	DJIHJMQVAWUMQB-GRNFJTJUSA-N	CCCCCCCCCCCCCCCCCC(=O)OCC(COP(O)(=O)O[C=H]1[C=H](O)[C=H](O)[C=H](O)[C=H](O)[C=H]1O)OC(=O)CC\C=C/C\C=C/CCCCCC
2784	18.026	460.41733	betulin	[M+H]+	460.41489	C30H50O2	Triterpenoids	FVWJYYTZTCVBKE-ROUWMTJPSA-N	OCC12CCC(C(=C)C)C2C3CCC4C5(C)CCC(O)C(C)(C)C5CCC4(C)C3(C)CC1
2785	18.027	459.34845	4-[5-[[4-[5-[acetyl(hydroxy)amino]pentylamino]-4-oxobutanoyl]-hydroxyamino]pentylamino]-4-oxobutanoic acid	[M-H]-	459.34872	C20H36N4O8	N-acyl amines	VJQANBSDAGCDJE-UHFFFAOYSA-N	O=C(O)CCC(O)=NCCCCCN(O)C(=O)CCC(O)=NCCCCCN(O)C(=O)C
2786	18.053	712.53729	(2R,3S,4R,5R,6R)-5-acetamido-2-(acetoxymethyl)-6-((2-dodecyltetradecyl)oxy)tetrahydro-2H-pyran-3,4-diyl diacetate	[M+H]+	712.53998	C40H73NO9	N-acyl-alpha-hexosamines	QVWUTEYXUYBGKO-WKHSNOJSSA-N	O=C(OCC1OC(OCC(CCCCCCCCCCCC)CCCCCCCCCCCC)C(NC(=O)C)C(OC(=O)C)C1OC(=O)C)C
2787	18.083	727.4798	2-[[5-[[2,12-dihydroxy-4,4,10,13,14-pentamethyl-17-(1,5,6-trihydroxy-6-methylheptan-2-yl)-2,3,5,6,7,11,12,15,16,17-decahydro-1H-	[M+H]+	727.47394	C38H63NO11	Triterpenoids	IOMBMOCSWVAYQU-UHFFFAOYSA-N	O=C(O)CN=C(O)CC(O)(C)CC(=O)OC1C(O)CC2(C3=C(CCC2C1(C)C)C4(C)CCC(C(CO)CCC(O)C(O)(C)C)C4(C)C(O)C3)C
2788	18.212	760.586	1-palmitoyl-2-oleoyl-sn-glycero-3-phosphocholine	[M+H]+	760.58508	C42H82NO8P	Phosphatidylcholines	WTJKGGKOPKCXLL-VYOBOKEXSA-N	CCCCCCCCCCCCCCCC(=O)OC[C=H](COP([O-])(=O)OCC[N+](C)(C)C)OC(=O)CCCCCCC\C=C/CCCCCCCC
2789	18.226	431.39066	Alpha-Tocopherol	[M+H]+	431.39001	C29H50O2	Tocopherols	GVJHHUAWPYXKBD-UHFFFAOYSA-N	OC=1C(=C(C(=2OC(C)(CCC2C1C)CCCC(C)CCCC(C)CCCC(C)C)C)C
2790	18.248	666.63434	[(3S,10S,11S,12S,13S,14S)-17-acetyl-11-acetyloxy-14-hydroxy-3-[5-[5-[5-[3-hydroxy-4-methoxy-6-methyl-5-[3,4,5-trihydroxy-6-(hydroxymethyl)oxan-2-	[M+H]+	666.63196	C66H100O25	Steroidal glycosides	GOZAGCBVCYGQHP-PRJUEBITSA-N	O=C(OC1C(OC(=O)C)C2C(CCC3CC(OC4OC(C)C(OC5OC(C)C(OC6OC(C)C(OC7OC(C)C(OC8OC(CO)C(O)C(O)C8O)C(OC)C7O)C(OC)C6)C(
2791	18.252	800.58673	Phosphatidylcholine 17	[M-H]-	800.58112	C43H80NO8P	Phosphatidylcholines	FNRBLPPAKRRBCG-QGRKPBPINA-N	CCCCCCCCCCC\C=C/CCC(=O)OCC(COP([O-])(=O)OCC[N+](C)(C)C)OC(=O)CC\C=C/C\C=C/CCCCCCCCC
2792	18.431	693.36017	(2S,3R)-2-(((S)-7-acetamido-1,2,3-trimethoxy-9-oxo-5,6,7,9-tetrahydrobenzo[a]heptalen-10-yl)amino)-N-(1-benzylpiperidin-4-yl)-3-	[M+H]+	693.35999	C39H50N4O6	Isoleucine and derivatives	ZXPRMRWMMPZQED-AVFXGLFGSA-N	O=C1C=C2C(=CC=C1NC(C(=O)NC3CCN(CC=4C=CC=CC4)CC3)C(C)CC)C5=C(OC)C(OC)=C(OC)C=C5CCC2NC(=O)C
2793	18.487	612.16748	[6-[2-(3,4-dihydroxyphenyl)-8-hydroxy-4-oxochromen-7-yl]oxy-3,4,5-trihydroxyoxan-2-yl]methyl (E)-3-(4-hydroxyphenyl)prop-2-enoate	[M+H]+	612.17114	C30H26O13	Flavonoid-7-O-glycosides	VXAHTPDZSOTOPQ-XCVCLJGOSA-N	O=C(OCC1OC(OC2=CC=C3C(=O)C=C(OC3=C2O)C=4C=CC(O)=C(O)C4)C(O)C(O)C1O)C=CC5=CC=C(O)C=C5

2794	18.491	244.97466	Per?uoro-3,5-dioxahexanoic acid	[M-H]-	244.96902	C4HF7O4	PFSA	GCSHTDCBGCURHU-UHFFFAOYSA-N	O=C(O)C(F)(F)OC(F)(F)OC(F)(F)F
2795	18.499	587.43097	(1R,6R,10S,11R,13S,15R)-1,6-dihydroxy-8-(hydroxymethyl)-4,12,12,15-tetramethyl-5-oxotetracyclo[8.5.0.0?,?.0??,??]pentadeca-3,8-dien-13-yl	[M+H]+	587.4306	C36H58O6	Phorbol esters	QUVRUINWOFZNJL-QNMVXEMISA-N	O=C(OC12CC(C)C3(O)C(C=C(CO)CC4(O)C(=O)C(=CC43)C)C1C2(C)C)CCCCCCCCCCCCCCC
2796	18.611	659.32812	(2E,6E,10E)-14-[(2S,3R,4S,5S,6R)-4,5-dihydroxy-6-(hydroxymethyl)-3-[(2S,3R,4S,5S,6R)-3,4,5-trihydroxy-6-(hydroxymethyl)oxan-2-yl]oxyoxan-2-	[M-H]-	659.32843	C32H52O14	Sophorolipids	ISQUNAAALVXWGI-PGEVILJJSA-N	O=C(O)C(=CC(O)CC(=CCCC(=CCCC(OC1OC(CO)C(O)C(O)C1OC2OC(CO)C(O)C(O)C2O)(C=C)C)C)C)C
2797	18.614	675.39581	[(2R,3R,4S,5S,6R)-3-acetyloxy-2-(acetyloxymethyl)-5-hexanoyloxy-6-[(2S,3R)-2,3,4-trihydroxybutoxy]oxan-4-yl] tetradecanoate	[M-H]-	675.39612	C34H60O13	Saccharolipids	QGQSOVLOEOVNRW-KSHKLCIXSA-N	O=C(OCC1OC(OCC(O)C(O)CO)C(OC(=O)CCCCC)C(OC(=O)CCCCCCCCCCCCC)C1OC(=O)C)C
2798	18.74	591.32898	12-O-[deca-2Z,4E-dienoyl]-13-isobutyroyloxy-4-deoxyphorbol	[M+H]+	591.32898	C34H48O7	Tigliane and ingenane diterpenoids	MXLZHQOOQIUQGO-FGQBBZSKSA-N	O=C(OC1C(C)C2(O)C3C=C(C(=O)C3CC(=CC2C4C1(OC(=O)C(C)C)C4(C)C)CO)C)C=CC=CCCCCC
2799	18.763	441.39322	6,8-dihydroxy-2,2,4,4-tetramethyl-7-(3-methylbutanoyl)-9-(2-methylpropyl)-9H-xanthene-1,3-dione	[M-H]-	441.38669	C26H34O6	Xanthenes	TYDBFNAOFZIICW-UHFFFAOYSA-N	O=C(C1=C(O)C=C2OC3=C(C(=O)C(C(=O)C3(C)C)(C)C)C(C2=C1O)CC(C)C)CC(C)C
2800	19.116	684.20117	ACARBOSE	[M+H]+	684.21118	C25H43NO18	Aminocyclitol glycosides	CEMXHAPUFJOOSV-UHFFFAOYSA-N	O=CC(O)C(O)C(OC1OC(CO)C(OC2OC(C)C(NC3C=C(CO)C(O)C(O)C3O)C(O)C2O)C(O)C1O)C(O)CO
2801	19.153	597.45178	Ubiquinone 30	[M+H]+	597.44897	C39H58O4	Ubiquinones	GXNFPEOUKFOTKY-LPHQIWJTSA-N	COC1=C(OC)C(=O)C(C\C=C(/C)CC\C=C(/C)CC\C=C(/C)CC\C=C(/C)CC\C=C(/C)CCC=C(C)C)=C(C)C1=O
2802	19.204	418.3157	11,12,13-trihydroxy-4,5,8-trimethyl-3-(2-methylpropyl)-1H,2H,3H,4H,6aH,9H,10H,11H,12H,13H,14H,15H,15bH-cycloundeca[e]isoindole-	[M-H]-	418.3179	C24H37NO5	Aspochalasins	PLGNVFSTPJUFKJ-UHFFFAOYSA-N	O=C1CC(O)C(O)C(O)CCC(=CC2C=C(C)C(C)C3C(N=C(O)C123)CC(C)C)C
2803	19.224	451.39075	Epifriedelanol	[M+H]+	451.39001	C30H52O	Triterpenoids	XCDQFROEGGNAER-PFOIMGGJSA-N	OC1CCC2C(C)(CCC3C2(C)CCC4(C)C5CC(C)(C)CCC5(C)CCC34C)C1C
2804	19.261	430.37527	Sorbitane Monostearate - Polysorbate 60 in-source fragment	[M+H]+	430.38	C24H46O6	Fatty acid esters	HVUMOYIDDBPOLL-UHFFFAOYSA-N	O=C(OCC(O)C1OCC(O)C1O)CCCCCCCCCCCCCCCCC
2805	19.33	409.38287	beta-amyrin-H2O	[M+H]+	409.38199	C30H50O	Triterpenoids	JFSHUTJDVKUMTJ-QHPUVITPSA-N	OC1CCC2(C)C(CCC3(C)C2CC=C4C5CC(C)(C)CCC5(C)CCC43C)C1(C)C
2806	19.36	565.40094	Canthaxanthin	[M+H]+	565.40399	C40H52O2	Xanthophylls	FDSDTBUPSURDBL-DKLMTRRASA-N	C\C(\C=C\C=C(/C)\C=C\C1=C(C)C(=O)CCC1(C)C)=C/C=C/C=C(\C)/C=C/C=C(\C)/C=C/C1=C(C)C(=O)CCC1(C)C
2807	19.367	566.40961	Eschscholtzxanthin	[M+H]+	566.4118	C40H54O2	Xanthophylls	DHHWDJUUTBWANN-WUEUEEBUSA-N	C\C(\C=C\C=C\C(\C)=C\C=C\C(\C)=C\C=C1\C(C)=C[C=H](O)CC1(C)C)=C/C=C/C(/C)=C/C=C1/C(C)=C[C=H](O)CC1(C)C
2808	19.367	405.37463	pentacosanoic acid	[M+H]+	405.37	C25H50O2	Very long-chain fatty acids	MWMPEAHGUXCSMY-UHFFFAOYSA-N	O=C(O)CCCCCCCCCCCCCCCCCCCCCCCC
2809	19.368	550.41418	3,4-Dehydroxy-3'-deoxycapsanthin	[M+H]+	550.41693	C40H54O	Xanthophylls	ZFQAIKNKCQDPPB-BKGWKKLQSA-N	C\C(\C=C\C=C(/C)\C=C\C(=O)[C=]1(C)CCCC1(C)C)=C/C=C/C=C(\C)/C=C/C=C(\C)/C=C/C1=C(C)C=CCC1(C)C
2810	19.41	838.53687	Phosphatidylethanolamine 22	[M-H]-	838.53925	C49H78NO8P	Phosphatidylethanolamines	UFXLSRZZVFZURD-YECZQSHBNA-N	CCCCCCCC\C=C/C\C=C/C\C=C/C\C=C/CCC(=O)OCC(COP(O)(=O)OCCN)OC(=O)CC\C=C/C\C=C/C\C=C/C\C=C/C\C=C/C\C=C/CC
2811	19.556	304.02798	"CYTIDINE 2',3'-CYCLIC MONO-PHOS-PHATE"	[M-H]-	304.03299	C9H12N3O7P	2',3'-cyclic pyrimidine nucleotides	NMPZCCZXCOMSDQ-XVFCMESISA-N	O=C1N=C(N)C=CN1C2OC(CO)C3OP(=O)(O)OC23
2812	19.586	397.38235	beta-Sitosterol	[M+H]+	397.38287	C29H50O	Stigmastanes and derivatives	KZJWDPNRJALLNS-GDQWMHGZSA-N	CCC(CCC(C)C1CCC2C3CC=C4C[C=H](O)CCC4(C)C3CCC12C)C(C)C
2813	19.603	443.33218	"Cycloart-23-ene-3_, 25 diol"	[M+H]+	443.33401	C30H50O2	Cycloartanols and derivatives	JSPGKOBNHWTKNT-CPGXJQKCSA-N	OC1CCC23CC43CCC5(C)C(CCC5(C)C4CCC2C1(C)C)C(C)CC=CC(O)(C)C
2814	19.612	917.54559	Notoginsenoside Fe	[M+H]+	917.547	C47H80O17	Triterpenoids	MYBAONSAUGZRAX-RMLJSBQESA-N	OCC1OC(OCC2OC(OC(C)(CCC=C(C)C)C3CCC4(C)C3C(O)CC5C6(C)CCC(OC7OC(CO)C(O)C(O)C7O)C(C)(C)C6CCC54C)C(O)C(O)C2O)C(
2815	19.675	321.00656	Chloramphenicol	[M-H]-	321.0051	C11H12Cl2N2O5	Nitrobenzenes	WIIZWVCIJKGZOK-RKDXNWHRSA-N	O=N(=O)C1=CC=C(C=C1)C(O)C(N=C(O)C(Cl)Cl)CO
2816	19.688	648.91968	PFSA-perfluoroalkyl_branched_C3	[M-H]-	648.92065	C11HF23O3S	PFSA	HNPWOKHGYHBURV-UHFFFAOYSA-N	O=S(=O)(O)C(F)(F)C(F)(F)C(F)(C(F)(F)F)C(F)(F)C(F)(F)C(F)(F)C(F)(F)C(F)(F)C(F)(F)C(F)(F)F
2817	19.701	538.51947	N-Palmitoyl-D-erythro-Sphingosine	[M+H]+	538.51935	C34H67NO3	Ceramides	YDNKGFDKKRUKPY-TURZORIXSA-N	CCCCCCCCCCCCCCCC(=O)N[C=H](CO)[C=H](O)\C=C\CCCCCCCCCCCCC
2818	19.724	315.06842	Benzoic acid + 2O, O-Hex	[M-H]-	315.07001	C13H16O9	Phenolic glycosides	CBTFERBMQQAROP-UHFFFAOYNA-N	O=C(O)C=2C=C(OC1OC(CO)C(O)C(O)C1(O))C=CC=2(O)
2819	19.745	842.63379	Phosphatidylcholine 18	[M-H]-	842.62805	C46H86NO8P	Phosphatidylcholines	JKGNHUXTJMEAQU-YDXMYUDNNA-N	CCCCCCCCCCCCCCCCCC(=O)OCC(COP([O-])(=O)OCC[N+](C)(C)C)OC(=O)CC\C=C/C\C=C/C\C=C/CCCCCCCC
2820	19.769	136.06223	Adenine	[M+H]+	136.06177	C5H5N5	6-aminopurines	GFFGJBXGBJISGV-UHFFFAOYSA-N	c1nc2c(N)nc[nH]c2n1
2821	19.772	391.22421	Dehydrorotenone	[M-H]-	391.21799	C23H20O6	Rotenones	GFERNZCCTZEIET-UHFFFAOYSA-N	O=C1C2=CC=C3OC(C(=C)C)CC3=C2OC4=C1C=5C=C(OC)C(OC)=CC5OC4
2822	19.783	180.97241	4-O-Methylphloracetophenone	[M-H]-	180.9747	C9H10O4	Alkyl-phenylketones	GKSGTWUNURZTKD-UHFFFAOYSA-N	O=C(C1=C(O)C=C(OC)C=C1O)C
2823	19.786	318.29977	Phytosphingosine (not validated, isomer of 1697)	[M+H]+	318.2998	C18H39NO3	Lipids	AERBNCYCJBRYDG-UHFFFAOYNA-N	OCC(N)C(O)C(O)CCCCCCCCCCCCCC
2824	19.788	822.53961	Scytophycin E	[M+H]+	822.53998	C45H75NO12	Diterpene lactones	DSHVEBDLSYMWSX-MRGDGBMUSA-N	O=CN(C=CC(C)C(OC)C(C(=O)CCC(C)C(O)C(C)C1OC(=O)C=CC(=CC(O)CC2OC(CC=C2)CC(OC)C(C)C(CO)C(OC)CC(OC)C1C)C)C)C
2825	19.79	233.15536	(E)-5-(2,3-dimethyl-4,5,6,7-tetrahydro-1H-tricyclo[2.2.1.02,6]heptan-3-yl)-2-methylpent-2-enoic acid	[M-H]-	233.15469	C15H22O2	Sesquiterpenoids	NZSCHTYUGUVLHG-WEVVVXLNSA-N	O=C(O)C(=CCCC1(C)C2CC3C(C2)C31C)C
2826	19.795	714.51086	L-alpha-Phosphatidylethanolamine (Soy)	[M-H]-	714.50793	C39H74NO8P	Phosphatidylethanolamines	HBZNVZIRJWODIB-NHCUFCNUSA-N	CCCCCCCCCCCCCCCC(=O)OC[C=H](COP(O)(=O)OCCN)OC(=O)CCCCCCC\C=C/C\C=C/CCCCC
2827	19.795	789.54236	Scytophycin C	[M+H]+	789.53998	C45H75NO11	Diterpene lactones	PNDDYPOQKFXUHY-JOSACIENSA-N	O=CN(C=CC(C)C(OC)C(C(=O)CCC(C)C(O)C(C)C1OC(=O)C=CC(=CC(O)CC2OC(CC=C2)CC(OC)C(C)C(OC)CC(OC)C1C)C)C)C
2828	19.8	788.54205	2-amino-3-[hydroxy-[3-octadecanoyloxy-2-[octadec-11-enoyl]oxypropoxy]phosphoryl]oxypropanoic acid	[M-H]-	788.54401	C42H80NO10P	Phosphatidylserines	LDPJFEPYYCUUOO-JQIJEIRASA-N	O=C(OCC(OC(=O)CCCCCCCCCC=CCCCCCC)COP(=O)(O)OCC(N)C(=O)O)CCCCCCCCCCCCCCCCC
2829	19.812	857.51843	Phosphatidylinositol 16	[M-H]-	857.51855	C45H79O13P	Phosphatidylinositols	QAOYQEIOBPZFPB-VHZSRAIHSA-N	CCCCCCCCCCCCCCCC(=O)OCC(COP(O)(=O)O[C=H]1[C=H](O)[C=H](O)[C=H](O)[C=H](O)[C=H]1O)OC(=O)CC\C=C/C\C=C/C\C=C/C\
2830	19.819	194.92296	2,3,5-Trichlorphenol	[M-H]-	194.91768	C6H3Cl3O	O-chlorophenols	WWGQHTJIFOQAOC-UHFFFAOYSA-N	C1=C(C=C(C(=C1Cl)Cl)O)Cl
2831	19.822	230.00613	Dimethoate	[M+H]+	230.0069	C5H12NO3PS2	Dithiophosphate O-esters	MCWXGJITAZMZEV-UHFFFAOYSA-N	CN=C(CSP(OC)(OC)=S)O
2832	19.822	469.2558	Limonin	[M-H]-	469.25238	C26H30O8	Limonoids	KBDSLGBFQAGHBE-MSGMIQHVSA-N	O=C1OCC23C(OC(C)(C)C3CC(=O)C4(C)C2CCC5(C)C(OC(=O)C6OC645)C7=COC=C7)C1
2833	19.828	564.32751	LPC 18:2	[M-H]-	564.32727	C26H50NO7P	Lipids	SPJFYYJXNPEZDW-UHFFFAOYNA-N	O=C(OCC(O)COP(=O)([O-])OCC[N+](C)(C)C)CCCCCCCC=CCC=CCCCCC
2834	19.829	105.01875	D-GLYCERIC ACID	[M-H]-	105.019	C3H6O4	Sugar acids and derivatives	RBNPOMFGQQGHHO-UHFFFAOYSA-N	O=C(O)C(O)CO
2835	19.83	199.06007	N,N-Dimethyl-N'-phenylsulfamide	[M-H]-	199.05467	C8H12N2O2S	Sulfanilides	QCDQDISRALTLNQ-UHFFFAOYSA-N	CN(C)S(=O)(=O)NC1=CC=CC=C1
2836	19.833	179.0553	Galactose	[M-H]-	179.05611	C6H12O6	Hexoses	WQZGKKKJIJFFOK-UHFFFAOYNA-N	OCC1OC(O)C(O)C(O)C1O

2837	19.834	225.06013	1,8,9-Anthracenetriol	[M-H]-	225.05573	C14H10O3	Anthracenes	YUTJCNNFTOIOGT-UHFFFAOYSA-N	OC1=CC=CC2=CC3=CC=CC(O)=C3C(O)=C12
2838	19.835	370.72946	2-(hydroxymethyl)-6-[[(1R,4S)-2,2,4-trimethyl-3-oxabicyclo[2.2.2]octan-5-yl]oxy]oxane-3,4,5-triol	[M+H]+	370.72919	C16H28O7	O-glycosyl compounds	NWZYTZHMCGWGOF-HLIZFUEWSA-N	OCC1OC(OC2CC3CCC2(OC3(C)C)C)C(O)C(O)C1O
2839	19.836	742.53802	Phosphatidylethanolamine 18	[M-H]-	742.53925	C41H78NO8P	Phosphatidylethanolamines	CJVLBVKIFVTUPS-JSCMVKKRNA-N	CCCCCCCCCCCCCCCCCC(=O)OCC(COP(O)(=O)OCCN)OC(=O)CC\C=C/C\C=C/CCCCCCCCCC
2840	19.836	161.02267	Salicyclic acid	[M+H]+	161.02	C7H6O3	Salicylic acids	YGSDEFSMJLZEOE-UHFFFAOYSA-N	O=C(O)C=1C=CC=CC1O
2841	19.836	167.01692	URATE	[M-H]-	167.021	C5H4N4O3	Xanthines	LEHOTFFKMJEONL-UHFFFAOYSA-N	O=C1NC(=O)C=2NC(=O)NC2N1
2842	19.837	177.0397	D-GULONIC ACID GAMA-LACTONE	[M-H]-	177.03999	C6H12O7	Medium-chain hydroxy acids and derivatives	RGHNJXZEOKUKBD-QTBDOELSSA-N	O=C(O)C(O)C(O)C(O)C(O)CO
2843	19.837	267.07068	Norberto Lopes	[M-H]-	267.07001	C16H12O4	4'-O-methylisoflavones	HKQYGTCOTHHOMP-UHFFFAOYSA-N	O=C1C(=COC2=CC(O)=CC=C21)C=3C=CC(OC)=CC3
2844	19.837	117.0185	Succinic acid (not validated)	[M-H]-	117.0182	C4H6O4	Organic acids	KDYFGRWQOYBRFD-UHFFFAOYSA-N	O=C(O)CCC(=O)O
2845	19.841	134.03107	Thiazolidine-4-carboxylic acid	[M+H]+	134.02702	C4H7NO2S	L-alpha-amino acids	DZLNHFMRPBPULJ-UHFFFAOYNA-N	O=C(O)C1NCSC1
2846	19.842	264.08609	2,3',4,6-Tetrahydroxybenzophenone	[M+H]+	264.08664	C13H10O5	Benzophenones	QWRYPHZJTWQLFX-UHFFFAOYSA-N	O=C(C=1C=CC=C(O)C1)C=2C(O)=CC(O)=CC2O
2847	19.845	223.01393	(7R,8R)-7,8-dihydroxy-3,5,7-trimethyl-8H-isochromen-6-one	[M+H]+	223.01343	C12H14O4	Azaphilones	JVUYBLROBHJEJN-NEPJUHHUSA-N	O=C1C(=C2C=C(OC=C2C(O)C1(O)C)C)C
2848	19.845	184.01772	Pesticide1_Acephate_C4H10NO3PS_O,S-Dimethyl acetylphosphoramidothioate	[M+H]+	184.019	C4H10NO3PS	Organothiophosphorus compounds	YASYVMFAVPKPKE-UHFFFAOYSA-N	O=P(N=C(O)C)(OC)SC
2849	19.846	327.25266	1,2-dihydroxyheptadec-16-yn-4-yl acetate	[M+H]+	327.25296	C19H34O4	Long-chain fatty alcohols	FHGZOCAZNHYWAL-UHFFFAOYSA-N	O=C(OC(CCCCCCCCCCC#C)CC(O)CO)C
2850	19.848	101.02376	2-Oxobutyric acid	[M-H]-	101.02441	C4H6O3	Short-chain keto acids and derivatives	TYEYBOSBBBHJIV-UHFFFAOYSA-N	CCC(=O)C(O)=O
2851	19.848	193.03444	D-(+)-Galacturonic acid	[M-H]-	193.03537	C6H10O7	Glucuronic acid derivatives	AEMOLEFTQBMNLQ-YMDCURPLSA-N	OC1O[C=H]([C=H](O)[C=H](O)[C=H]1O)C(O)=O
2852	19.848	163.03859	Umbelliferone	[M+H]+	163.03897	C9H6O3	7-hydroxycoumarins	ORHBXUUXSCNDEV-UHFFFAOYSA-N	OC1=CC2=C(C=C1)C=CC(=O)O2
2853	19.851	160.04372	Brompheniramine	[M+H]+	160.04401	C20H23BrN2O4	Pheniramines	SRGKFVAASLQVBO-BTJKTKAUSA-N	O=C(O)C=CC(=O)O.BrC1=CC=C(C=C1)C(C2=NC=CC=C2)CCN(C)C
2854	19.854	158.11694	Homostachydrine	[M+H]+	158.11757	C8H15NO2	Alpha amino acids	XULZWQRXYTVUTE-ZETCQYMHSA-N	C[N+]1(C)CCCC[C=H]1C(=O)[O-]
2855	19.855	165.07521	1,5-Anhydrosorbitol	[M+H]+	165.07574	C6H12O5	Monosaccharides	MPCAJMNYNOGXPB-UHFFFAOYNA-N	OCC1OCC(O)C(O)C1O
2856	19.859	100.11179	Cyclohexylamine	[M+H]+	100.11208	C6H13N	Cyclohexylamines	PAFZNILMFXTMIY-UHFFFAOYSA-N	C1CCC(CC1)N
2857	19.861	478.29318	Phosphatidylethanolamine lyso 18	[M-H]-	478.29391	C23H46NO7P	2-acyl-sn-glycero-3-phosphoethanolamines	FEVAUEZKXSRQPS-PFONDFGANA-N	CCCCCCCCCCCCC\C=C/CCC(=O)OC(CO)COP(O)(=O)OCCN
2858	19.861	183.08594	Sorbitol	[M+H]+	183.08627	C6H14O6	Sugar alcohols	FBPFZTCFMRRESA-JGWLITMVSA-N	OCC(O)C(O)C(O)C(O)CO
2859	19.862	274.27481	Palmitic Acid	[M+H]+	274.27411	C16H32O2	Long-chain fatty acids	IPCSVZSSVZVIGE-UHFFFAOYSA-N	O=C(O)CCCCCCCCCCCCCCC
2860	19.862	301.23651	Roccellic Acid	[M+H]+	301.23734	C17H32O4	Long-chain fatty acids	CADNMISJDLVPCK-LSDHHAIUSA-N	O=C(O)C(C)C(C(=O)O)CCCCCCCCCCC
2861	19.864	199.01796	MCPA	[M-H]-	199.01674	C9H9ClO3	Chlorophenoxyacetates	WHKUVVPPKQRRBV-UHFFFAOYSA-N	CC1=CC(=CC=C1OCC(=O)O)Cl
2862	19.869	284.91183	Homopterocarpin	[M+H]+	284.91422	C17H16O4	1-benzopyrans	GIWAJLOUUBQKKA-UHFFFAOYSA-N	O(C1=CC=C2C(OCC3OC4=CC(OC)=CC=C4C23)=C1)C
2863	19.873	189.03371	4-nitroquinoline 1-oxide	[M-H]-	189.03056	C9H6N2O3	4-nitroquinoline N-oxides	YHQDZJICGQWFHK-UHFFFAOYSA-N	O=N(=O)C1=CC=N(=O)C=2C=CC=CC12
2864	19.892	167.99811	2-Mercaptobenzothiazole	[M+H]+	167.99362	C7H5NS2	Benzothiazoles	YXIWHUQXZSMYRE-UHFFFAOYSA-N	C1=CC=C2C(=C1)N=C(S)S2
2865	19.898	83.0601	3-Methylpyrazole	[M+H]+	83.06037	C4H6N2	Pyrazoles	XKVUYEYANWFIJX-UHFFFAOYSA-N	CC=1C=CNN1
2866	19.898	351.16913	hirsutanone	[M+H]+	351.16895	C19H20O5	Linear diarylheptanoids	VWHYFMQKJYFLCC-DUXPYHPUSA-N	O=C(C=CCC1=CC=C(O)C(O)=C1)CCC2=CC=C(O)C(O)=C2
2867	19.904	265.09631	D-Tryptophan	[M+H]+	265.09698	C11H12N2O2	Indolyl carboxylic acids and derivatives	QIVBCDIJIAJPQS-SECBINFHSA-N	O=C(O)C(N)CC1=CNC=2C=CC=CC21
2868	19.904	449.26535	Mappain	[M+H]+	449.26901	C29H36O4	Stilbenes	GJZKDVGLDGLPMB-ZVBRSKEYSA-N	OC=1C=C(C=CC2=CC(O)=C(C(O)=C2)CC=C(C)C)C=C(C1O)CC=C(C)CCC=C(C)C
2869	19.91	130.04935	Pyroglutamic acid	[M+H]+	130.04987	C5H7NO3	Alpha amino acids and derivatives	ODHCTXKNWHHXJC-UHFFFAOYNA-N	O=C(O)C1N=C(O)CC1
2870	19.92	87.04472	Isobutyric acid	[M-H]-	87.04515	C4H8O2	Carboxylic acids	KQNPFQTWMSNSAP-UHFFFAOYSA-N	O=C(O)C(C)C
2871	19.925	357.12521	Pioglitazone	[M+H]+	357.12674	C19H20N2O3S	Phenol ethers	HYAFETHFCAUJAY-UHFFFAOYNA-N	CCC=1C=CC(CCOC2=CC=C(C=C2)CC3C(=NC(=O)S3)O)=NC1
2872	19.927	224.04832	1-Hydroxyanthraquinone	[M-H]-	224.0479	C14H8O3	Anthraquinones	BTLXPCBPYBNQNR-UHFFFAOYSA-N	C1=CC=C2C(=C1)C(C=3C=CC=C(C3C2=O)O)=O
2873	19.928	199.02962	Herniarin	[M+H]+	199.03	C10H8O3	Coumarins and derivatives	LIIALPBMIOVAHH-UHFFFAOYSA-N	O=C1OC=2C=C(OC)C=CC2C=C1
2874	19.929	237.02075	6,7,8-trimethoxycoumarin	[M+H]+	237.02609	C12H12O5	Coumarins and derivatives	RAYQKHLZHPFYEJ-UHFFFAOYSA-N	O=C1OC=2C(OC)=C(OC)C(OC)=CC2C=C1
2875	19.932	134.05969	INDOXYL SULFATE	[M+H]+	134.06	C8H7NO4S	Arylsulfates	BXFFHSIDQOFMLE-UHFFFAOYSA-N	O=S(=O)(O)OC1=CNC=2C=CC=CC12
2876	19.933	316.25439	Decanoyl-L-carnitine	[M+H]+	316.24823	C17H33NO4	Acyl carnitines	LZOSYCMHQXPBFU-UHFFFAOYSA-N	O=C([O-])CC(OC(=O)CCCCCCCCC)C[N+](C)(C)C
2877	19.936	221.99419	HISTAMINE DIHYDROCHLORIDE	[M+H]+	221.99615	C5H11Cl2N3	2-arylethylamines	PPZMYIBUHIPZOS-UHFFFAOYSA-N	Cl.Cl.N1=CNC(=C1)CCN
2878	19.939	207.1373	Ibuprofen	[M+H]+	207.13699	C13H18O2	Phenylpropanoic acids	HEFNNWSXXWATRW-UHFFFAOYSA-N	O=C(O)C(C1=CC=C(C=C1)CC(C)C)C
2879	19.944	116.07018	Proline	[M+H]+	116.07	C5H9NO2	Proline and derivatives	ONIBWKKTOPOVIA-BYPYZUCNSA-N	O=C(O)C1NCCC1

2880	19.946	178.04254	Isoxanthopterin	[M-H]-	178.03705	C6H5N5O2	Pterins and derivatives	GLKCOBIIZKYKFN-UHFFFAOYSA-N	N=C1N=C(O)C=2N=CC(O)=NC2N1
2881	19.951	169.12199	Geranic acid	[M+H]+	169.1223	C10H16O2	Acyclic monoterpenoids	ZHYZQXUYZJNEHD-UHFFFAOYSA-N	O=C(O)C=C(C)CCC=C(C)C
2882	19.952	207.06438	7,8-dimethoxy-2H-chromen-2-one	[M+H]+	207.064	C11H10O4	Coumarins and derivatives	CHBBSMUTOCUVDW-UHFFFAOYSA-N	O=C1OC=2C(OC)=C(OC)C=CC2C=C1
2883	19.965	254.10156	10-Azabenzo[a]pyrene	[M+H]+	254.09642	C19H11N	Pyrenes	BYFIUFNRSNKODH-UHFFFAOYSA-N	C1=CC=2C=CC3=CC=4C=CC=NC4C=5C=CC(=C1)C2C35
2884	19.969	148.06073	L-Glutamic acid	[M+H]+	148.06044	C5H9NO4	Glutamic acid and derivatives	WHUUTDBJXJRKMK-VKHMYHEASA-N	C(CC(=O)O)[C=H](C(=O)O)N
2885	19.97	200.1277	Methylecgonine	[M+H]+	200.12801	C10H17NO3	Tropane alkaloids	QIQNNBXHAYSQRY-UHFFFAOYSA-N	O=C(OC)C1C(O)CC2N(C)C1CC2
2886	19.971	253.05798	rubiadin	[M-H]-	253.0596	C15H10O4	Hydroxyanthraquinones	IRZTUXPRIUZXMP-UHFFFAOYSA-N	O=C1C=2C=CC=CC2C(=O)C3=C(O)C(=C(O)C=C13)C
2887	19.972	149.04596	Arabinose	[M-H]-	149.04555	C5H10O5	Pentoses	PYMYPHUHKUWMLA-UHFFFAOYNA-N	O=CC(O)C(O)C(O)CO
2888	19.972	193.03604	D-sorbosonic acid	[M-H]-	193.03539	C6H10O7	Sugar acids and derivatives	VBUYCZFBVCCYFD-FLRLBIABSA-N	O=C(O)C(=O)C(O)C(O)C(O)CO
2889	19.972	135.0304	Threonic acid	[M-H]-	135.02989	C4H8O5	Sugar acids and derivatives	JPIJQSOTBSSVTP-UHFFFAOYNA-N	O=C(O)C(O)C(O)CO
2890	19.975	185.02164	m-Xylene-4-sulfonic acid	[M-H]-	185.02779	C8H10O3S	p-Methylbenzenesulfonates	CHZLVSBMXZSPNN-UHFFFAOYSA-N	CC=1C=CC(=C(C)C1)S(O)(=O)=O
2891	19.985	177.04108	Gluconic acid-gamma-lactone	[M-H]-	177.04047	C6H10O6	Gluconolactones	PHOQVHQSTUBQQK-UHFFFAOYNA-N	O=C1OC(CO)C(O)C(O)C1O
2892	19.989	145.04993	3-METHYGLUTARIC ACID	[M-H]-	145.05	C6H10O4	Methyl-branched fatty acids	XJMMNTGIMDZPMU-UHFFFAOYSA-N	O=C(O)CC(C)CC(=O)O
2893	19.989	117.01871	SUCCINATE	[M-H]-	117.019	C4H6O4	Dicarboxylic acids and derivatives	KDYFGRWQOYBRFD-UHFFFAOYSA-N	O=C(O)CCC(=O)O
2894	19.991	197.06747	Harmol	[M-H]-	197.07204	C12H10N2O	Alkaloids	SATMZMMKDDTOSQ-UHFFFAOYSA-N	CC1=C2NC3=C(C=CC(O)=C3)C2=CC=N1
2895	19.993	130.05026	Pyroglutamic acid (not validated, isomer of 89)	[M+H]+	130.0504	C5H7NO3	Amino acids	ODHCTXKNWHHXJC-UHFFFAOYNA-N	O=C(O)C1NC(=O)CC1
2896	19.998	146.02515	1H-Isoindole-1,3(2H)-dione	[M-H]-	146.02475	C8H5NO2	Phthalimides	XKJCHHZQLQNZHY-UHFFFAOYSA-N	C1=CC=C2C(=C1)C(=NC2=O)O
2897	19.998	217.03114	4,4`-Thiodiphenol	[M-H]-	217.03287	C12H10O2S	Diarylthioethers	VWGKEVWFBOUAND-UHFFFAOYSA-N	C=1C=C(C=CC1O)SC2=CC=C(C=C2)O
2898	19.998	138.02016	4-Nitrophenol	[M-H]-	138.01967	C6H5NO3	Nitrophenols	BTJIUGUIPKRLHP-UHFFFAOYSA-N	O=N(=O)C1=CC=C(O)C=C1
2899	19.999	129.01863	Itaconic acid (Not validated)	[M-H]-	129.0188	C5H6O4	Organic acids	LVHBHZANLOWSRM-UHFFFAOYSA-N	O=C(O)C(=C)CC(=O)O
2900	20	159.06561	3-Methyladipic acid	[M-H]-	159.06628	C7H12O4	Medium-chain fatty acids	SYEOWUNSTUDKGM-UHFFFAOYNA-N	O=C(O)CCC(C)CC(=O)O
2901	20.004	156.06519	Indole-3-acetonitrile	[M+H]+	156.06821	C10H8N2	3-alkylindoles	DMCPFOBLJMLSNX-UHFFFAOYSA-N	N#CCC1=CNC2=CC=CC=C12
2902	20.014	337.20325	Bavachinin A	[M-H]-	337.20111	C21H22O4	6-prenylated flavanones	VOCGSQHKPZSIKB-FQEVSTJZSA-N	O=C1C2=CC(=C(OC)C=C2OC(C3=CC=C(O)C=C3)C1)CC=C(C)C
2903	20.017	261.0162	isofraxidin	[M+H]+	261.01599	C11H10O5	7-hydroxycoumarins	HOEVRHHMDJKUMZ-UHFFFAOYSA-N	O=C1OC=2C(OC)=C(O)C(OC)=CC2C=C1
2904	20.019	296.1842	Esmolol	[M+H]+	296.18564	C16H25NO4	Phenol ethers	AQNDDEOPVVGCPG-UHFFFAOYNA-N	O=C(OC)CCC1=CC=C(OCC(O)CNC(C)C)C=C1
2905	20.02	297.11691	Enterolactone	[M-H]-	297.11322	C18H18O4	Lignols	HVDGDHBAMCBBLR-UHFFFAOYNA-N	OC1=CC(CC2COC(=O)C2CC2=CC(O)=CC=C2)=CC=C1
2906	20.024	249.99564	2-[(4,6-diamino-1,3,5-triazin-2-yl)thio]-Ethanesulfonic acid	[M-H]-	250.00101	C5H9N5O3S2	1,3,5-triazine-2,4-diamines	VOWJSEYJDXBICY-UHFFFAOYSA-N	O=S(=O)(O)CCSC1=NC(=N)NC(=N)N1
2907	20.024	164.06999	Clozapine (Clozaril)	[M+H]+	164.073	C18H19ClN4	Dibenzodiazepines	QZUDBNBUXVUHMW-UHFFFAOYSA-N	ClC1=CC=C2NC=3C=CC=CC3C(=NC2=C1)N4CCN(C)CC4
2908	20.03	306.97766	(2E)-2-[hydroxy(phenyl)methylidene]-6-methoxy-1-benzofuran-3-one	[M+H]+	306.97165	C16H12O4	Aurone flavonoids	TXVHSAPNXGBCKX-JQIJEIRASA-N	O=C1C(OC2=CC(OC)=CC=C21)=C(O)C=3C=CC=CC3
2909	20.031	228.98563	Hymecromone Methyl Ether	[M+H]+	228.98586	C11H10O3	Coumarins and derivatives	UDFPKNSWSYBIHO-UHFFFAOYSA-N	O=C1OC=2C=C(OC)C=CC2C(=C1)C
2910	20.037	169.08588	FA 9:2+1O	[M-H]-	169.08591	C9H14O3	Oxidized fatty acids	QHYBPAALYZNWPK-UHFFFAOYSA-N	O=C(O)C=CC(=O)CCCCC
2911	20.041	193.03444	D-GLUCURONIC ACID	[M-H]-	193.035	C6H10O7	Glucuronic acid derivatives	AEMOLEFTQBMNLQ-UHFFFAOYSA-N	O=C(O)C1OC(O)C(O)C(O)C1O
2912	20.042	179.05533	Psicose	[M-H]-	179.05611	C6H12O6	Monosaccharides	BJHIKXHVCXFQLS-UHFFFAOYNA-N	O=C(CO)C(O)C(O)C(O)CO
2913	20.043	133.04961	Dihydroxy-Valerate	[M-H]-	133.0497	C5H10O4	Fatty acids	JTEYKUFKXGDTEU-UHFFFAOYNA-N	O=C(O)C(O)C(O)(C)C
2914	20.044	122.09614	2,4,6-Trimethylpyridine	[M+H]+	122.09643	C8H11N	Methylpyridines	BWZVCCNYKMEVEX-UHFFFAOYSA-N	CC1=CC(C)=NC(C)=C1
2915	20.044	96.04408	3-Hydroxypyridine	[M+H]+	96.04439	C5H5NO	Hydroxypyridines	GRFNBEZIAWKNCO-UHFFFAOYSA-N	c1cc(cnc1)O
2916	20.045	177.0397	D-GLUCONATE	[M-H]-	177.03999	C6H12O7	Medium-chain hydroxy acids and derivatives	RGHNJXZEOKUKBD-UHFFFAOYSA-N	O=C(O)C(O)C(O)C(O)C(O)CO
2917	20.047	211.00325	Pesticide5_Acibenzolar-S-methyl_C8H6N2OS2_Actigard	[M+H]+	210.99899	C8H6N2OS2	Benzothiadiazoles	UELITFHSCLAHKR-UHFFFAOYSA-N	O=C(SC)C1=CC=CC=2N=NSC21
2918	20.051	163.039	2-Coumaric acid	[M-H]-	163.04007	C9H8O3	Hydroxycinnamic acids	PMOWTIHVNWZYFI-AATRIKPKSA-N	OC(=O)\C=C\C1=CC=CC=C1O
2919	20.052	125.02366	Benzene-1,2,4-triol	[M-H]-	125.02441	C6H6O3	Hydroxyquinols and derivatives	GGNQRNBDZQJCCN-UHFFFAOYSA-N	OC1=CC=C(O)C(O)=C1
2920	20.055	167.03416	Homogentisic acid	[M-H]-	167.03499	C8H8O4	2(hydroxyphenyl)acetic acids	IGMNYECMUMZDDF-UHFFFAOYSA-N	OC(=O)CC1=CC(O)=CC=C1O
2921	20.056	161.04457	Hydroxymethylglutaric acid	[M-H]-	161.0451	C6H10O5	Organic acids	NPOAOTPXWNWTSH-UHFFFAOYSA-N	O=C(O)CC(O)(C)CC(=O)O
2922	20.061	101.07047	Gyromitrin	[M+H]+	101.07094	C4H8N2O	N-alkylated hydrazones	IMAGWKUTFZRWSB-HWKANZROSA-N	O=CN(N=CC)C

2923	20.062	224.09085	Pesticide3_Dioxacarb_C11H13NO4_Phenol, 2-(1,3-dioxolan-2-yl)-, methylcarbamate	[M+H]+	224.091	C11H13NO4	Phenoxy compounds	SDKQRNRRDYRQKY-UHFFFAOYSA-N	OC(=NC)OC=1C=CC=CC1C2OCCO2
2924	20.063	195.05006	Xanthone	[M-H]-	195.0452	C13H8O2	Xanthones	JNELGWHKGNBSMD-UHFFFAOYSA-N	O=C1C=2C=CC=CC2OC=3C=CC=CC31
2925	20.069	146.05243	3-Formylindole	[M+H]+	146.06004	C9H7NO	Indoles	OLNJUISKUQQNIM-UHFFFAOYSA-N	O=CC1=CNC2=CC=CC=C12
2926	20.07	206.08853	1-phenylisoquinoline	[M+H]+	206.09	C15H11N	Phenylpyridines	LPCWDYWZIWDTCV-UHFFFAOYSA-N	N=1C=CC=2C=CC=CC2C1C=3C=CC=CC3
2927	20.07	108.08051	2,6-Dimethylpyridine	[M+H]+	108.08077	C7H9N	Methylpyridines	OISVCGZHLKNMSJ-UHFFFAOYSA-N	CC=1C=CC=C(C)N1
2928	20.071	183.101	Angelic anhydride	[M+H]+	183.10001	C10H14O3	Dicarboxylic acids and derivatives	LIHLSLRANKCHLV-SFECMWDFSA-N	O=C(OC(=O)C(=CC)C)C(=CC)C
2929	20.071	118.08569	Valine	[M+H]+	118.086	C5H11NO2	Valine and derivatives	KZSNJWFQEVHDMF-UHFFFAOYSA-N	O=C(O)C(N)C(C)C
2930	20.076	269.08661	7,3',4'-Trihydroxyflavone	[M-H]-	269.08456	C15H10O5	Flavones	PVFGJHYLIHMCQD-UHFFFAOYSA-N	O=C1C=C(OC2=CC(O)=CC=C12)C=3C=CC(O)=C(O)C3
2931	20.078	103.03949	3-HYDROXYBUTANOIC ACID	[M-H]-	103.04	C4H8O3	Beta hydroxy acids and derivatives	WHBMMWSBFZVSSR-GSVOUGTGSA-N	O=C(O)CC(O)C
2932	20.079	196.09613	Glucosaminic Acid	[M+H]+	196.09639	C6H13NO6	D-alpha-amino acids	UFYKDFXCZBTLOO-TXICZTDVSA-N	O=C(O)C(N)C(O)C(O)C(O)CO
2933	20.083	72.08051	Pyrrolidine	[M+H]+	72.08078	C4H9N	Pyrrolidines	RWRDLPDLKQPQOW-UHFFFAOYSA-N	C1CCNC1
2934	20.093	131.11752	N-Acetylputrescine	[M+H]+	131.11789	C6H14N2O	Carboximidic acids	KLZGKIDSEJWEDW-UHFFFAOYSA-N	CC(O)=NCCCCN
2935	20.094	147.06473	Phenyltetrazole	[M+H]+	147.06653	C7H6N4	Phenyltetrazoles and derivatives	MARUHZGHZWCEQU-UHFFFAOYSA-N	C1=CC=C(C=C1)C2=NNN=N2
2936	20.1	267.07315	INOSINE	[M-H]-	267.073	C10H12N4O5	Purine nucleosides	UGQMRVRMYYASKQ-UHFFFAOYSA-N	O=C1N=CNC2=C1N=CN2C3OC(CO)C(O)C3O
2937	20.118	180.10094	Phenacetin	[M+H]+	180.10201	C10H13NO2	Acetanilides	CPJSUEIXXCENMM-UHFFFAOYSA-N	O=C(NC1=CC=C(OCC)C=C1)C
2938	20.12	278.8667	3,5-dibromo-2-methoxyphenol	[M-H]-	278.866	C7H6Br2O2	Methoxyphenols	YFCZJZFKQUAEGT-UHFFFAOYSA-N	BrC=1C=C(Br)C(OC)=C(O)C1
2939	20.122	156.06592	3-Methylcrotonylglycine	[M-H]-	156.06662	C7H11NO3	N-acyl-alpha amino acids	PFWQSHXPNKRLIV-UHFFFAOYSA-N	O=C(O)CN=C(O)C=C(C)C
2940	20.123	74.05972	N,N-Dimethylformamide	[M+H]+	74.06004	C3H7NO	Tertiary carboxylic acid amides	ZMXDDKWLCZADIW-UHFFFAOYSA-N	O=CN(C)C
2941	20.124	90.0547	Alanine	[M+H]+	90.05495	C3H7NO2	Alanine and derivatives	QNAYBMKLOCPYGJ-UHFFFAOYNA-N	O=C(O)C(N)C
2942	20.127	167.03412	Vanillic acid	[M-H]-	167.03499	C8H8O4	M-methoxybenzoic acids and derivatives	WKOLLVMJNQIZCI-UHFFFAOYSA-N	COC1=CC(=CC=C1O)C(O)=O
2943	20.129	187.05923	Rhamnose	[M+H]+	187.05769	C6H12O5	Hexoses	PNNNRSAQSRJVSB-BXKVDMCESA-N	C[C=H](O)[C=H](O)[C=H](O)[C=H](O)C=O
2944	20.133	246.12245	4-Acetamidoantipyrine	[M+H]+	246.1237	C13H15N3O2	Phenylpyrazoles	OIAGWXKSCXPNNZ-UHFFFAOYSA-N	CN1N(C(=O)C(N=C(C)O)=C1C)C1=CC=CC=C1
2945	20.137	297.15091	ostruthin	[M-H]-	297.1496	C19H22O3	Terpene lactones	INBMTJJPUABOQJ-VGOFMYFVSA-N	O=C1OC=2C=C(O)C(=CC2C=C1)CC=C(C)CCC=C(C)C
2946	20.14	122.05975	Benzamide	[M+H]+	122.06004	C7H7NO	Benzamides	KXDAEFPNCMNJSK-UHFFFAOYSA-N	C1=CC=C(C=C1)C(=N)O
2947	20.141	170.08087	Pyridoxine	[M+H]+	170.08099	C8H11NO3	Pyridoxines	LXNHXLLTXMVWPM-UHFFFAOYSA-N	OC=1C(=NC=C(C1CO)CO)C
2948	20.152	169.08575	2-(3-oxobutyl)cyclopentane-1,3-dione	[M+H]+	169.09	C9H12O3	Beta-diketones	XRLGHWYZNGYZOW-UHFFFAOYSA-N	O=C(C)CCC1C(=O)CCC1=O
2949	20.152	397.07581	Propoxycarbazone	[M-H]-	397.08234	C15H18N4O7S	Benzoic acid esters	JTHMVYBOQLDDIY-UHFFFAOYSA-N	CCCOC1=NN(C(=NS(C2=CC=CC=C2C(=O)OC)(=O)=O)O)C(N1C)=O
2950	20.158	205.08662	1-[4-hydroxy-3-(3-methylbut-2-enyl)phenyl]ethanone	[M+H]+	205.08617	C13H16O2	Alkyl-phenylketones	QJAPFSSVKIZTMR-UHFFFAOYSA-N	O=C(C1=CC=C(O)C(=C1)CC=C(C)C)C
2951	20.167	182.08046	2-(4-aminotetrahydro-2H-pyran-4-yl)acetic acid	[M+H]+	182.08	C7H13NO3	Pyranoid amino acids and derivatives	QRIAWCUEIMQLGG-UHFFFAOYSA-N	O=C(O)CC1(N)CCOCC1
2952	20.169	241.07086	lusianthridin	[M-H]-	241.0719	C15H14O3	Hydrophenanthrenes	RDKDIPDDUFMMMT-UHFFFAOYSA-N	OC=1C=CC2=C(C1)CCC3=CC(OC)=CC(O)=C23
2953	20.173	132.10143	Leucine	[M+H]+	132.1019	C6H13NO2	Leucine and derivatives	ROHFNLRQFUQHCH-UHFFFAOYNA-N	O=C(O)C(N)CC(C)C
2954	20.177	169.04903	2,6-Dimethoxyquinone	[M+H]+	169.04953	C8H8O4	P-benzoquinones	OLBNOBQOQZRLMP-UHFFFAOYSA-N	O=C1C=C(OC)C(=O)C(OC)=C1
2955	20.179	76.039	Acetohydroxamic acid	[M+H]+	76.03931	C2H5NO2	Acetohydroxamic acids	RRUDCFGSUDOHDG-UHFFFAOYSA-N	ON=C(O)C
2956	20.181	166.04961	Phthalamic acid	[M+H]+	166.04987	C8H7NO3	Benzoic acids	CYMRPDYINXWJFU-UHFFFAOYSA-N	C1=CC=C(C(=C1)C(=N)O)C(=O)O
2957	20.189	182.11749	Homoveratrylamine	[M+H]+	182.11755	C10H15NO2	Dimethoxybenzenes	ANOUKFYBOAKOIR-UHFFFAOYSA-N	O(C1=CC=C(C=C1OC)CCN)C
2958	20.19	103.04034	2-Hydroxyisobutyric acid	[M-H]-	103.04007	C4H8O3	Alpha hydroxy acids and derivatives	BWLBGMIXKSTLSX-UHFFFAOYSA-N	O=C(O)C(O)(C)C
2959	20.191	202.09972	(1S,2S)-2-(methylamino)-1-phenylpropan-1-ol hydrochloride	[M+H]+	202.10001	C10H16ClNO	Phenylpropanes	BALXUFOVQVENIU-KXNXZCPBSA-N	Cl.OC(C=1C=CC=CC1)C(NC)C
2960	20.194	511.46915	Gallamine	[M+H]+	511.46909	C30H60N3O3	Phenol ethers	OZLPUNFFCJDMJD-UHFFFAOYSA-N	CC[N+](CC)(CC)CCOC1=CC=CC(OCC[N+](CC)(CC)CC)=C1OCC[N+](CC)(CC)CC
2961	20.194	89.0237	GLYCERALDEHYDE	[M-H]-	89.0239	C3H6O3	Monosaccharides	MNQZXJOMYWMBOU-UHFFFAOYSA-N	O=CC(O)CO
2962	20.201	253.1801	ilicic acid	[M+H]+	253.17999	C15H24O3	Eudesmane, isoeudesmane or cycloeudesmane	FXKCXGBBUBCRPU-QHSBEEBCSA-N	O=C(O)C(=C)C1CCC2(C)CCCC(O)(C)C2C1
2963	20.204	99.04379	alpha-Methylene-gamma-butyrolactone	[M+H]+	99.04405	C5H6O2	Gamma butyrolactones	GSLDEZOOOSBFGP-UHFFFAOYSA-N	O=C1OCCC1=C
2964	20.204	207.06506	Citropen	[M+H]+	207.065	C11H10O4	Coumarins and derivatives	NXJCRELRQHZBQA-UHFFFAOYSA-N	O=C1OC2=CC(OC)=CC(OC)=C2C=C1
2965	20.21	217.07001	Caffeine	[M+H]+	217.07001	C8H10N4O2	Xanthines	RYYVLZVUVIJVGH-UHFFFAOYSA-N	O=C1C2=C(N=CN2C)N(C(=O)N1C)C

2966	20.223	143.03363	cis,cis-Muconic acid	[M+H]+	143.03389	C6H6O4	Medium-chain fatty acids	TXXHDPDFNKHHGW-CCAGOZQPSA-N	OC(=O)\C=C/C=C\C(O)=O
2967	20.227	187.07864	1-Naphthoic acid methyl ester	[M+H]+	187.07536	C12H10O2	Naphthalenecarboxylic acids and derivatives	HMRROBKAACRWBP-UHFFFAOYSA-N	COC(C1=CC=CC2=CC=CC=C21)=O
2968	20.228	128.10646	N-Cyclohexylformamide	[M+H]+	128.10699	C7H13NO	Secondary carboxylic acid amides	SWGXDLRCJNEEGZ-UHFFFAOYSA-N	OC=NC1CCCCC1
2969	20.233	86.09613	PIPERIDINE	[M+H]+	86.09643	C5H11N	Piperidines	NQRYJNQNLNOLGT-UHFFFAOYSA-N	C1CCNCC1
2970	20.245	178.08586	PUTRESCINE DIHYDROCHLORIDE	[M+H]+	178.08722	C4H14Cl2N2	Monoalkylamines	XXWCODXIQWIHQN-UHFFFAOYSA-N	Cl.Cl.NCCCCN
2971	20.26	215.03214	5-Methoxypsoralen	[M-H]-	215.03499	C12H8O4	5-methoxypsoralens	BGEBZHIAGXMEMV-UHFFFAOYSA-N	O=C1OC=2C=C3OC=CC3=C(OC)C2C=C1
2972	20.265	195.17368	GERANYL ACETONE	[M+H]+	195.17435	C13H22O	Acyclic monoterpenoids	HNZUNIKWNYHEJJ-FMIVXFBMSA-N	CC(C)=CCC\C(C)=C\CCC(C)=O
2973	20.273	240.03497	Indole-3-Acetaldehyde	[M-H]-	240.0336	C10H11NO4S	3-alkylindoles	AGASQWULIFZUOK-UHFFFAOYNA-N	OC(CC1=CNC2=CC=CC=C12)S(O)(=O)=O
2974	20.277	188.10721	4-(1H-indol-3-yl)butan-2-one	[M+H]+	188.11	C12H13NO	3-alkylindoles	ZJCUUXGLZWBCIL-UHFFFAOYSA-N	O=C(C)CCC1=CNC=2C=CC=CC21
2975	20.291	310.85416	Ovalitenin B	[M+H]+	310.85498	C19H18O4	Butyrophenones	AADNEQWIZKTMBL-UHFFFAOYSA-N	O=C(C1=CC=C2OC=CC2=C1OC)CC(OC)C=3C=CC=CC3
2976	20.305	167.03374	"3,4-DIHYDROXYPHENYLACETATE"	[M-H]-	167.034	C8H8O4	Catechols	CFFZDZCDUFSOFZ-UHFFFAOYSA-N	O=C(O)CC1=CC=C(O)C(O)=C1
2977	20.308	112.98613	Trifluoroacetic acid	[M-H]-	112.98559	C2HF3O2	PFSA	DTQVDTLACAAQTR-UHFFFAOYSA-N	O=C(O)C(F)(F)F
2978	20.319	195.12218	Pelletierine Hydrochloride	[M+H]+	195.12585	C8H16ClNO	Piperidines	FMOKUKPRJSMPOI-UHFFFAOYSA-N	Cl.O=C(C)CC1NCCCC1
2979	20.328	124.03905	Nicotinic acid	[M+H]+	124.03931	C6H5NO2	Pyridinecarboxylic acids	PVNIIMVLHYAWGP-UHFFFAOYSA-N	c1cc(cnc1)C(=O)O
2980	20.33	241.00102	alizarin	[M+H]+	241.0043	C14H8O4	Hydroxyanthraquinones	RGCKGOZRHPZPFP-UHFFFAOYSA-N	O=C1C=2C=CC=CC2C(=O)C3=C(O)C(O)=CC=C13
2981	20.373	105.01872	GLYCERATE	[M-H]-	105.019	C3H6O4	Sugar acids and derivatives	RBNPOMFGQQGHHO-UHFFFAOYSA-N	O=C(O)C(O)CO
2982	20.381	131.03468	CITRACONIC ACID	[M+H]+	131.03389	C5H6O4	Methyl-branched fatty acids	HNEGQIOMVPPMNR-IHWYPQMZSA-N	C\C(=C\C(O)=O)C(O)=O
2983	20.413	410.19739	8-(azepan-1-ium-1-ylmethyl)-3-(2,4-dimethoxyphenyl)-2-oxo-2H-chromen-7-olate	[M+H]+	410.20001	C24H27NO5	Hydroxyisoflavonoids	VOBOQLHWHJWFCX-UHFFFAOYSA-N	O=C1OC2=C(C=CC([O-])=C2C[NH+]3CCCCCC3)C=C1C4=CC=C(OC)C=C4OC
2984	20.424	178.97702	Difluoro(perfluoromethoxy)acetic acid	[M-H]-	178.97731	C3HF5O3	PFSA	IHYMJUVQLSNPPP-UHFFFAOYSA-N	O=C(O)C(F)(F)OC(F)(F)F
2985	20.449	180.9895	kojic acid	[M+H]+	180.98976	C6H6O4	Pyranones and derivatives	BEJNERDRQOWKJM-UHFFFAOYSA-N	O=C1C=C(OC=C1O)CO
2986	20.455	213.98218	Chloroxine	[M+H]+	213.98199	C9H5Cl2NO	Chloroquinolines	WDFKMLRRRCGAKS-UHFFFAOYSA-N	ClC1=CC(Cl)=C2C=CC=NC2=C1O
2987	20.478	266.98193	Makaluvamine O	[M+H]+	266.98001	[C10H8BrN2O2]+	Pyrrolo[4,3,2-de]quinolines	FDGKQWLSJXEOPF-UHFFFAOYSA-O	O=C1C(=O)C=2NC=C3C2C(=C1Br)[NH2+]CC3
2988	20.484	282.02362	PRZ_M282	[M+H]+	282.02136	C11H14Cl3NO	Phenol ethers	CLFQSOIBYICELN-UHFFFAOYSA-N	CCCNCCOC1=C(C=C(C=C1Cl)Cl)Cl
2989	20.5	138.99495	Fumaric acid	[M+H]+	139	C4H4O4	Dicarboxylic acids and derivatives	VZCYOOQTPOCHFL-OWOJBTEDSA-N	O=C(O)C=CC(=O)O
2990	20.509	251.01062	Bauerine B	[M+H]+	251.01199	C12H8Cl2N2	Beta carbolines	XGTYSLIGRKUXED-UHFFFAOYSA-N	ClC=1C=CC=2C=3C=CN=CC3N(C2C1Cl)C
2991	20.512	110.02415	Pyrrole-2-carboxylic acid	[M-H]-	110.02475	C5H5NO2	Pyrrole 2-carboxylic acids	WRHZVMBBRYBTKZ-UHFFFAOYSA-N	O=C(O)C1=CC=CN1
2992	20.521	263.01022	L-Cystine	[M+H]+	263.01001	C6H12N2O4S2	L-cysteine-S-conjugates	LEVWYRKDKASIDU-IMJSIDKUSA-N	O=C(O)C(N)CSSCC(N)C(=O)O
2993	20.525	243.01859	Sodium Danshensu	[M+H]+	243.02	C9H9NaO5	Phenylpropanoic acids	ZMMKVDBZTXUHFO-DDWIOCJRSA-M	[Na+].O=C([O-])C(O)CC1=CC=C(O)C(O)=C1
2994	20.543	317.21625	Epiafzelechin Trimethyl Ether	[M+H]+	317.21445	C18H20O5	7-O-methylated flavonoids	NMLBREXWQRPOJH-CRAIPNDOSA-N	OC1CC=2C(OC)=CC(OC)=CC2OC1C3=CC=C(OC)C=C3
2995	20.552	123.00809	Propane sulfate	[M-H]-	123.0113	C3H8O3S	Sulfonic acids	HNDXKIMMSFCCFW-UHFFFAOYSA-N	O=S(=O)(O)C(C)C
2996	20.556	465.23865	(2E,6E,10E)-13-[(2R)-6-hydroxy-2,8-dimethyl-3,4-dihydrochromen-2-yl]-2,6,10-trimethyltrideca-2,6,10-trienoic acid	[M+H]+	465.24014	C27H38O4	Tocotrienols	QOFWRHWADNWKSU-LRXIOGKNSA-N	O=C(O)C(=CCCC(=CCCC(=CCCC1(OC=2C(=CC(O)=CC2CC1)C)C)C)C)C
2997	20.558	305.96692	PFSM-FSAA	[M-H]-	305.96765	C5H4F7NO4S	PFSA	MXFTXMQPMJCELP-UHFFFAOYSA-N	O=C(O)CNS(=O)(=O)C(F)(F)C(F)(F)C(F)(F)F
2998	20.58	200.99466	(4S,5Z,6S)-4-(2-methoxy-2-oxoethyl)-5-[2-[(E)-3-phenylprop-2-enoyl]oxyethylidene]-6-[(2S,3R,4S,5S,6R)-3,4,5-trihydroxy-6-	[M+H]+	200.99486	C9H6O3	7-hydroxycoumarins	ORHBXUUXSCNDEV-UHFFFAOYSA-N	O=C1OC=2C=C(O)C=CC2C=C1
2999	20.587	197.02109	Juglone	[M+H]+	197.0209	C10H6O3	Naphthoquinones	KQPYUDDGWXQXHS-UHFFFAOYSA-N	O=C1C=CC(=O)C=2C(O)=CC=CC12
3000	20.593	117.05611	2-Hydroxy-3-methylbutyric acid	[M-H]-	117.05572	C5H10O3	Hydroxy fatty acids	NGEWQZIDQIYUNV-UHFFFAOYNA-N	O=C(O)C(O)C(C)C
3001	20.593	168.98755	Phosphoenolpyruvic acid	[M+H]+	168.98965	C3H5O6P	Phosphate esters	DTBNBXWJWCWCIK-UHFFFAOYSA-N	O=C(O)C(OP(=O)(O)O)=C
3002	20.605	279.00589	danthron	[M+H]+	279.0054	C14H8O4	Anthraquinones	QBPFLULOKWLNNW-UHFFFAOYSA-N	O=C1C=2C=CC=C(O)C2C(=O)C=3C(O)=CC=CC13
3003	20.607	359.06995	Mycophenolic Acid	[M+H]+	359.06616	C17H20O6	Phthalides	HPNSFSBZBAHARI-RUDMXATFSA-N	O=C1OCC2=C1C(O)=C(C(OC)=C2C)CC=C(C)CCC(=O)O
3004	20.612	215.97879	2-Benzothiazolesulfonic acid	[M+H]+	215.97836	C7H5NO3S2	Benzothiazoles	ZCXGMSGCBDSEOY-UHFFFAOYSA-N	C1=CC=C2C(=C1)N=C(S2)S(O)(=O)=O
3005	20.632	208.07002	2,3-dihydro-1H-carbazol-4(9H)-one	[M+H]+	208.07001	C12H11NO	Carbazoles	DSXKDTZEIWTHRO-UHFFFAOYSA-N	O=C1C=2C=3C=CC=CC3NC2CCC1
3006	20.725	258.0051	Arecoline hydrobromide	[M+H]+	258.01001	C8H14BrNO2	Alkaloids and derivatives	AXOJRQLKMVSHHZ-UHFFFAOYSA-N	[Br-].O=C(OC)C1=CCC[NH+](C)C1
3007	20.727	122.02555	Cysteine	[M+H]+	122.02702	C3H7NO2S	Cysteine and derivatives	XUJNEKJLAYXESH-UHFFFAOYNA-N	O=C(O)C(N)CS
3008	20.762	88.07539	Morpholine	[M+H]+	88.07569	C4H9NO	Morpholines	YNAVUWVOSKDBBP-UHFFFAOYSA-N	C1COCCN1

3009	20.79	164.99457	maltol	[M+H]+	164.99486	C6H6O3	Pyranones and derivatives	XPCTZQVDEJYUGT-UHFFFAOYSA-N	O=C1C=COC(=C1O)C
3010	20.816	194.98166	Orotic Acid	[M+H]+	194.98027	C5H4N2O4	Pyrimidinecarboxylic acids	PXQPEWDEAKTCGB-UHFFFAOYSA-N	O=C(O)C=1N=C(O)N=C(O)C1
3011	20.832	379.08411	Justicidin G	[M+H]+	379.08124	C21H14O7	Lignols	VINGQMQXGDIELG-UHFFFAOYSA-N	COC1=C2OCOC2=CC2=C(C3=C(C=C12)C(=O)OC3)C1=CC2=C(OCO2)C=C1
3012	20.835	269.2471	methyl palmitoleate	[M+H]+	269.2475	C17H32O2	Fatty acid methyl esters	IZFGRAGOVZCUFB-HJWRWDBZSA-N	O=C(OC)CCCCCCCC=CCCCCCC
3013	20.838	148.03912	Isatin	[M+H]+	148.03931	C8H5NO2	Indolines	JXDYKVIHCLTXOP-UHFFFAOYSA-N	O=C1NC2=CC=CC=C2C1=O
3014	20.877	162.11195	L-Carnitine	[M+H]+	162.11247	C7H15NO3	Carnitines	PHIQHXFUZVPYII-ZCFIWIBFSA-N	C[N+](C)(C)C[C=H](CC(=O)[O-])O
3015	20.885	123.05493	Nicotinamide	[M+H]+	123.05529	C6H6N2O	Nicotinamides	DFPAKSUCGFBDDF-UHFFFAOYSA-N	N=C(O)C=1C=NC=CC1
3016	20.89	261.18573	falcarindiol	[M+H]+	261.18491	C17H24O2	Long-chain fatty alcohols	QWCNQXNAFCBLLV-RCQSYPNMSA-N	OC(C#CC#CC(O)C=CCCCCCCC)C=C
3017	20.894	236.01015	Pesticide1_Omethoate_C5H12NO4PS_	[M+H]+	236.011	C5H12NO4PS	Secondary carboxylic acid amides	PZXOQEXFMJCDPG-UHFFFAOYSA-N	O=C(NC)CSP(=O)(OC)OC
3018	20.914	130.0658	Indole-3-carbinol	[M+H]+	130.06512	C9H9NO	3-alkylindoles	IVYPNXXAYMYVSP-UHFFFAOYSA-N	OCC1=CNC2=CC=CC=C12
3019	20.951	251.99133	Pesticide1_Dimethoate_C5H12NO3PS2_Phosphorodithioic acid, O,O-dimethyl S-[2-(methylamino)-2-oxoethyl] ester	[M+H]+	251.989	C5H12NO3PS2	Dithiophosphate O-esters	MCWXGJITAZMZEV-UHFFFAOYSA-N	S=P(OC)(OC)SCC(O)=NC

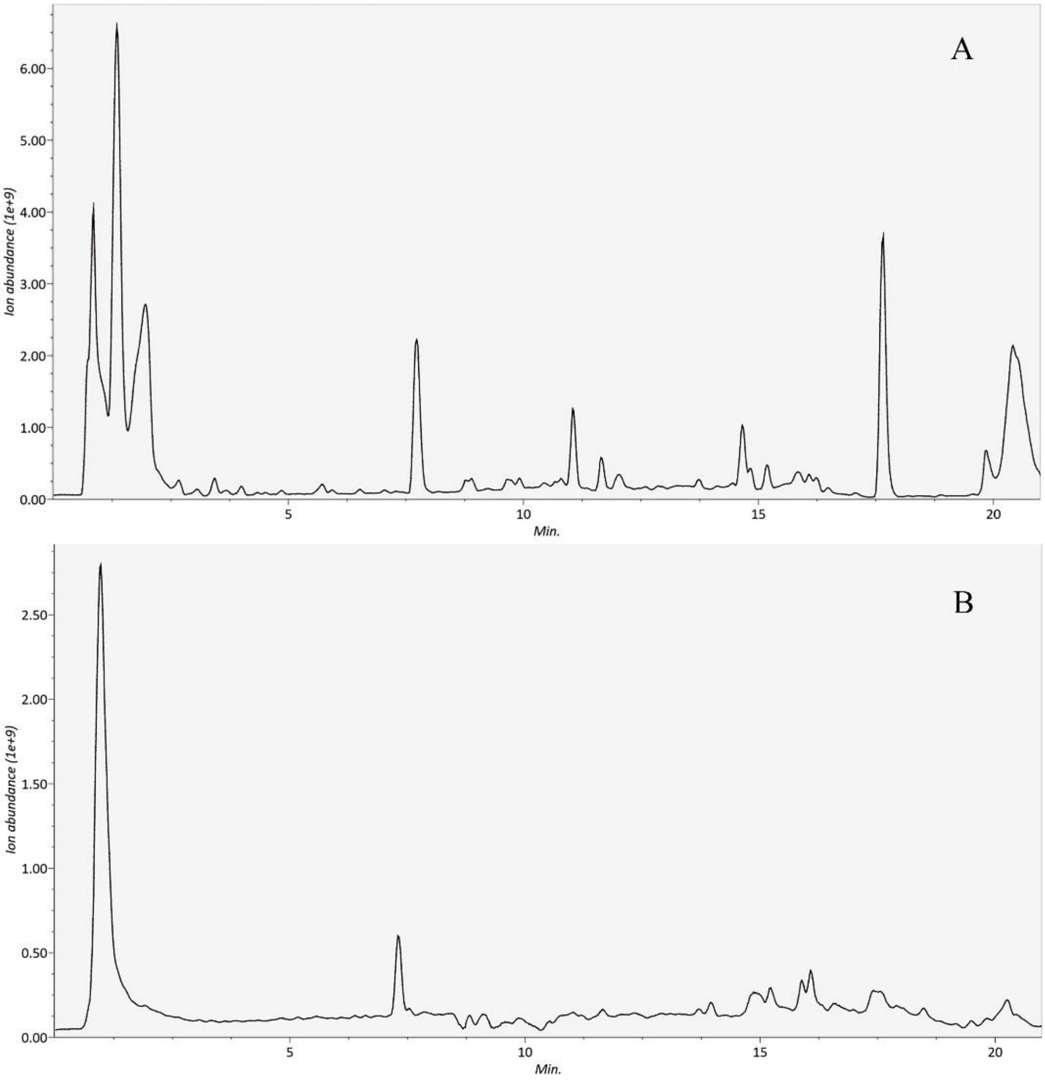

Figure S1. Total ion flow diagram in positive ion mode (**A**) and negative ion mode (**B**) of LC-MS/MS.
